
JOURNAL INFORMATION
==============================
NLM Title Abbreviation: JBMR Plus
Journal ID: JBMR Plus
NLM Journal ID: 101707013
Journal ID: jbmrplus

JBMR Plus

EISSN: 2473-4039
Publisher: Oxford University Press

ARTICLE INFORMATION
==============================
PMCID: PMC11103445
DOI: 10.1093/jbmrpl/ziae063
Article ID: ziae063
Article version: 1
Subjects: Abstracts, AcademicSubjects/MED00010, AcademicSubjects/MED00160, AcademicSubjects/MED00250

Abstracts of the ECTS 2024 Congress

Publication date: 2024 May
Electronic publication date: 2024 May 20
Volume: 8
Issue: Suppl 1
First page: i1
Last page: i340
Copyright: © The Author(s) 2024. Published by Oxford University Press on behalf of the American Society for Bone and Mineral Research.
Copyright year: 2024
License: This is an Open Access article distributed under the terms of the Creative Commons Attribution License (https://creativecommons.org/licenses/by/4.0/), which permits unrestricted reuse, distribution, and reproduction in any medium, provided the original work is properly cited.
License URL: https://creativecommons.org/licenses/by/4.0/

Page count: 1

==============================

### Abstracts of the ECTS 2024 Congress

### ECTS 2024 Congress

51st European Calcified Tissue Society Congress

25 – 28 May 2024

**Scientific Programme Committee**

**Chair:**
Peter Pietschmann (Vienna, Austria)

**Co-Chairs:**
Bo Abrahamsen (Holbæk, Denmark)
**Clinical Co-Chair:**
Nuria Guañabens (Barcelona, Spain)
**Basic Co-Chair:**
Philippe Zysset (Bern, Switzerland)
**Local Organizing Committee (LOC)-Chair:**
Martine Cohen-Solal (Paris, France)

**Members:**
Aline Bozec (Erlangen, Germany)
Eleni Douni (Athens, Greece)
Stella D'Oronzo (Bari, Italy)
Pawel Matusik (Katowice, Poland)
Graham Williams (London, United Kingdom)

**International Associate Members:**
Mary Bouxsein (Boston, USA)
Gustavo Duque (Montreal, Canada)

**Local Organising Committee:**
Roland Chapurlat (Lyon, France)
Anne-Laurence Baiada-Demoux (Marseille, France)
Thomas Funck-Brentano (Paris, France)
Marie-Eva Pickering (Clermont-Ferrand, France)
Federic Velard (Reims, France)

**Abstract Review Panel**
Each abstract was scored blind.

Bo Abrahamsen (Denmark)
Giovanni Adami (Italy)
Nerea Alonso (Austria)
Athanasios Anastasilakis (Greece)
Christina Andreasen (Denmark)
Natasha Appelman-Dijkstra (Netherlands)
Korhan Baklacı (Turkey)
Nicola Baldini (Italy)
Ulrike Baschant (Germany)
Duncan Bassett (United Kingdom)
Giulia Battafarano (Italy)
Sarah Beck-Cormier (France)
Zhanna Belaya (Russia)
Teresita Bellido (United States)
Francis Berenbaum (France)
Clemens Bergwitz (United States)
Claudine Blin (France)
Jean-Jacques Body (Belgium)
Jens Bollerslev (Norway)
Annemieke Boot (Netherlands)
Roger Bouillon (Belgium)
Mary Bouxsein (United States)
Aline Bozec (Germany)
Karine Briot (France)
Ludmila Brunerova (Czech Republic)
Björn Busse (Germany)
Natalie C Butterfield (United Kingdom)
M Leonor Cancela (Portugal)
John Carey (Ireland)
Geert Carmeliet (Belgium)
Mara Carsote (Romania)
Antonino Catalano (Italy)
Andrei Chagin (Sweden)
Roland Chapurlat (France)
Chantal Chenu (United Kingdom)
Roberto Civitelli (United States)
Emma Clark (United Kingdom)
Gavin Clunie (United Kingdom)
Martine Cohen-Solal (France)
Graziana Colaianni (Italy)
Luciano Colangelo (Italy)
Juliet Compston (United Kingdom)
Steven Cummings (United States)
Bess Dawson-Hughes (United States)
Teun De Vries (Netherlands)
Stella D'oronzo (Italy)
Patricia Ducy (United States)
Grahame Elder (Australia)
Florent Elefteriou (United States)

Ari Elson (Israel)
Karol Estrada (United States)
Vincent Everts (Netherlands)
Astrid Fahrleitner-Pammer (Austria)
Colin Farquharson (United Kingdom)
Verena Fischer (Germany)
Melissa M Formosa (Malta)
Morten Frost (Denmark)
Seiji Fukumoto (Japan)
Thomas Funck-Brentano (France)
Sonja Gamsjaeger (Austria)
Kaare Gautvik (Norway)
Luigi Gennari (Italy)
Valerie Geoffroy (France)
Jeroen Geurts (Switzerland)
Fernando Gianfrancesco (Italy)
Claus-Christian Glüer (Germany)
Mary Goldring (United States)
Roger Gomis (Spain)
Alanna Green (United Kingdom)
Jenny Gregory (United Kingdom)
Celia Gregson (United Kingdom)
Agi Grigoriadis (United Kingdom)
Daniel Grinberg (Spain)
Bettina Groetsch (Germany)
Núria Guañabens (Spain)
Melanie Haffner-Luntzer (Germany)
Morten Svarer Hansen (Denmark)
Barbara Hauser (United Kingdom)
Ursula Heilmeier (Germany)
Marietta Herrmann (Germany)
Eric Hesse (Germany)
Lorenz Hofbauer (Germany)
Wolfgang Högler (Austria)
Ingunn Holen (United Kingdom)
Donata Iandolo (France)
Anita Ignatius (Germany)
Yuuki Imai (Japan)
Alex Ireland (United Kingdom)
Abbas Jafari (Denmark)
Hervé Kempf (France)
Franziska Knopf (Germany)
Uwe Kornak (Germany)
Franziska Lademann (Germany)
Beate Lanske (United Kingdom)
Michaël Laurent (Belgium)
Laurence Legeai-Mallet (France)
Ulf Lerner (Sweden)
Thomas Levin Andersen (Denmark)
Mattias Lorentzon (Sweden)
Irma Machuca-Gayet (France)
Maria-Bernadette Madel (United States)
Christa Maes (Belgium)

Outi Mäkitie (Finland)
Polyzois Makras (Greece)
Janja Marc (Serbia)Radmila Matijevic (Slovenia)
Antonio Maurizi (Italy)
Michelle Mcdonald (Australia)
Carolina Medina-Gomez (Netherlands)
Ciro Menale (Italy)
Daniela Merlotti (Italy)
Petar Milovanovic (Serbia)
Salvatore Minisola (Italy)
Barbara Misof (Austria)
Ralph Müller (Switzerland)
Riko Nishimura (Japan)
George Nuki (United Kingdom)
Barbara Obermayer-Pietsch (Austria)
Ling Oei (Netherlands)
Richard Oreffo (United Kingdom)
Isabel Orriss (United Kingdom)
Julien Paccou (France)
Eleftherios Paschalis (Austria)
Pilar Peris (Spain)
Olivier Peyruchaud (France)
Peter Pietschmann (Austria)
Richard Pikner (Czech Republic)
Marco Ponzetti (Denmark)
Sylvain Provot (France)
Adalbert Raimann (Austria)
Saravana Ramasamy (United Kingdom)
Martina Rauner (Germany)
Lars Rejnmark (Denmark)
Mara Riminucci (Italy)
Fernando Rivadeneira (Netherlands)
Ilaria Roato (Italy)
Pamela Gehron Robey (United States)
Tim Rolvien (Germany)
Antonio Rossi (Italy)
Kenneth Saag (United States)
Syazrah Salam (United Kingdom)
Gonzalo Sanchez-Duffhues (Spain)
Arne Schaefer (Germany)
Martin Schepelmann (Austria)
Thorsten Schinke (Germany)
Felix Nikolai Schmidt (Germany)
Katharina Schmidt-Bleek (Germany)
Federica Scotto di Carlo (Italy)
Heide Siggelkow (Germany)
Cristina Sobacchi (Italy)
Kent Søe (Denmark)
Anne Sophie Sølling (Denmark)
Antonia Sophocleous (Cyprus)
Jakob Starup-Linde (Denmark)
Steve Stegen (Belgium)
Gudrun Stenbeck (United Kingdom)

Pawel Szulc (France)
Gaia Tabacco (Italy)
Hanna Taipaleenmaki (Germany)
Michaela Tencerova (Czech Republic)
Anna Maria Teti (Italy)
Jonathan Tobias (United Kingdom)
Drenka Trivanovic (Serbia)
Elena Tsourdi (Germany)
Jan Tuckermann (Germany)
Mustafa Unal (Turkey)
Jean Vacher (Canada)
Maria Teresa Valenti (Italy)
Jeroen van de Peppel (Netherlands)
Bram van der Eerden (Netherlands)
Marjolein Van Driel (Netherlands)
Wim Van Hul (Belgium)
Laurence Vico (France)
Anna Villa (Italy)
Ling Wang (China)
Ning Wang (United Kingdom)
Michael Whyte (United States)
Graham Williams (United Kingdom)
Elizabeth Winter (Netherlands)
Maria Yavropoulou (Greece)
Timur Yorgan (Germany)
Vit Zikan (Czech Republic)
Carola Zillikens (Netherlands)
Phillipe Zysset (Switzerland)

**Poster number abbreviations:**
PLOxx – Plenary Oral Presentation
COPxx – Concurrent Oral Presentation
Pxx – Poster
NDxx – New Data Poster

**Plenary Oral Presentations**

**Plenary Oral Presentations 1: Rare Bone Diseases and Cancer**

**PLO01**

**The Wnt1G177C mutation impairs load-induced bone formation via nuclear receptor 4a2**

*Mubashir Ahmad*[1], *Melanie Haffner-Luntzer*[1], *Astrid Schoppa*[1], *Timur Alexander Yorgan*[2], *Michael Amling*[2], *Thorsten Schinke*[2], *Anita Ignatius*[1]
[1]*Institute of Orthopedic Research and Biomechanics, University Medical Center Ulm, Ulm, Germany* ; [2]*Department of Osteology and Biomechanics, University Medical Center Hamburg-Eppendorf, Hamburg, Germany*

**Abstract Text**

**Introduction:** The Wnt signaling pathways plays a crucial role in the skeletal response to mechanical load, with recent emphasis on Wnt1 in load-induced bone formation. Despite its recognized importance, the precise mechanisms of Wnt1, especially in the context of the Wnt1 mutation linked to osteogenesis imperfecta type XV (OI-XV), remain elusive.

**Purpose:** To explore the impact of the $Wnt1^{G177C}$ loss-of-function mutation during mechanical loading and elucidate the signaling cascades governing Wnt1-mediated bone formation.

**Methods:** In vivo ulna loading, RNAseq, primary osteoblast isolation, in vitro mechanical stimulation using laminar fluid flow (LFF), siRNA transfection, qPCR, and statistical analysis.

**Results:** To understand the functional implications of the Wnt1 mutation ($Wnt1^{G177C}$) in the bone's anabolic response to loading, 12-week-old female mice from both $Wnt1^{+/+}$ and $Wnt1^{G177C}$ strains underwent ulnae loading (Fig. 1A). µCT analysis revealed that ulna loading substantially increased cortical thickness in $Wnt1^{+/+}$ control mice, whereas this effect was attenuated in $Wnt1^{G177C}$ mutant mice (Fig. 1B-C). Exploring pathways influenced by the $Wnt1^{G177C}$ mutation during mechanical loading through RNAseq analysis, we identified 1704 DEGs (Fig. 1D). Metascape analysis revealed several modulated pathways, including skeletal system development, and extracellular matrix organization. The comparison with RNAseq on primary osteoblasts, where *Wnt1* was deleted using siRNA, and which underwent LFF, highlighted 16 common genes, with nuclear receptor subfamily 4, group A, member 2 *(Nr4a2)* consistently dependent on both $Wnt1^{G177C}$ mutation and stimulation (Fig. 1E). Depleting *NR4A2* in human mesenchymal stem cells confirmed its positive role in promoting osteoblast differentiation. Our study demonstrates that the $Wnt1^{G177C}$ mutation impairs mechanical loading-induced bone formation through modulation of *Nr4a2* expression.

**Conclusion:** Our research highlights the significant impact of the $Wnt1^{G177C}$ mutation on impairing the bone's response to mechanical loading. These findings suggest potential therapeutic interventions targeting these pathways to improve bone formation in response to mechanical stimuli.

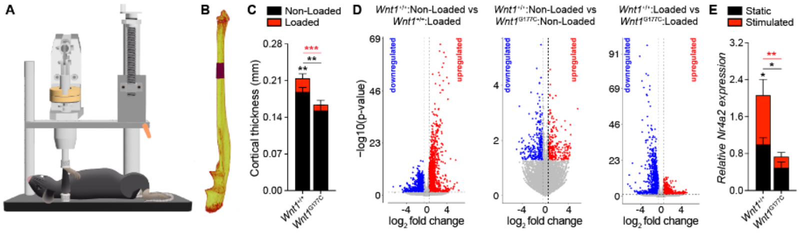

**Fig. 1: Impact of the *Wnt1*$^{G177C}$ loss-of-function mutation on mechanically induced bone formation via modulation of *Nr4a2* expression.** (A) Illustration depicting the methodology of ulnae loading in mice with *Wnt1*$^{+/+}$ and *Wnt1*$^{G177C}$ genotypes. (B) Representative micro-computed tomography (μCT) image of mouse ulnae, highlighting the 3 mm distal segement selected for subsequent μCT analysis post-loading. (C) Cortical thickness assessed by μCT analysis in female mice with *Wnt1*$^{+/+}$ and *Wnt1*$^{G177C}$ genotypes (n=4/group) following ulnae loading (day 16). (D) Volcano plots illustrating the differentially expressed genes (DEGs) with upregulated genes shown in red and downregulated genes shown in blue. DEGs significance criteria: log2 fold change (log2FC) ≤ -0.5 and ≥ 0.5; p-value < 0.05) (n=4/group).Non-DEGs are depicted in grey. (E) qPCR analysis of *Nr4a2* expression (0h after LFF) in primary murine osteoblasts isolated from mice with *Wnt1*$^{+/+}$ and *Wnt1*$^{G177C}$ genotypes (n=5/group).

**PLO02**

**Bi-allelic missense variants of ERI1 cause a new type of spondylo-epi-metaphyseal dysplasia**

*Long Guo*[1,2], *Rong Qiang*[3], *Shiro Ikegawa*[4]
[1]*The Affiliated Northwest Women's and Children's Hospital of Xi'an Jiaotong University Health Science Center, Center of Medical Genetics, Xi'an, China ;* [2]*School of Basic Medical Sciences of Xi'an Jiaotong University Health Science Center, Department of Laboratory Animal Science, Xi'an, China ;* [3]*The Affiliated Northwest Women's and Children's Hospital of Xi'an Jiaotong University Health Science Center, Center of Medical Genetics, Xi'an, China ;* [4]*RIKEN Center for Integrative Medical Sciences, Laboratory for Bone and Joint Diseases, Tokyo, Japan*

**Abstract Text**

**Background:** Spondylo-epi-metaphyseal dysplasia (SEMD) is a heterogeneous group of monogenic diseases characterized by the skeletal abnormalities that affects spine and epi- and meta-physes of long tubular bones. Identification of more than 10 causal genes for SEMD over the past few decades has not only improved genetic counseling of SEMD but also continuously contributed to the understanding of human skeletal development. However, there are still many SEMD whose disease genes are undetermined.

**Purpose:** To identify new disease genes for SEMD and clarify its phenotype and pathogenesis.

**Methods:** We recruited patients with unknown types of SEMD. The potential causal variants were identified by whole exome sequencing. The effects of these variants were analyzed using patient-derived fibroblasts and induced pluripotent stem cells (iPSCs). The gene function was evaluated by knock-out (KO) mice and *in vitro* chondrogenesis of patient-derived iPSCs.

**Results:** We discovered a new type of SEMD. Five affected individuals from four unrelated families are characterized by short stature, spine anomalies (platyspondyly, scoliosis and irregular endplates), epi-metaphyseal deformities of long bones, and dysplastic digits (clinodactyly, camptodactyly, brachydactyly, and oligosyndactyly) (Figure 1A). The affected individuals had bi-allelic missense variants of *ERI1*, which encodes a 3'-to-5' exoribonuclease involved in multiple RNA metabolic pathways (Figure 1B). These ERI1 missense variants cause a loss of the exoribonuclease activity, leading to defective trimming of the 5.8S rRNA 3' end and a decreased degradation of replication-dependent histone mRNAs ($P < 0.05$ for two-sided t test) (Figure 1C). *Eri1* KO mice showed similar features with the SEMD patients in spine and short bones (Figure 1D). Patient-specific iPSCs showed impaired *in vitro* chondrogenesis with down-regulation of genes regulating skeletal patterning ($P <$ 0.05 for two-sided t test) (Figure 1E).

**Conclusion:** We establish a novel SEMD entity in human (SEMD, Guo-Campeau type) and indicate that ERI1-mediated RNA metabolism regulates skeletal development.

**PLO03**

**A pathogenic role for brain-derived neurotrophic factor (BDNF) in Fibrous Dysplasia of bone**

*Biagio Palmisano*[1], *Giorgia Farinacci*[1], *Federica Campolo*[2], *Chiara Tavanti*[1], *Alessia Stefano*[1], *Samantha Donsante*[1], *Ernesto Ippolito*[3], *Giuseppe Giannicola*[4], *Mary Anna Venneri*[2], *Alessandro Corsi*[1], *Mara Riminucci*[1]
[1]*Sapienza University, Molecular Medicine, Rome, Italy ;* [2]*Sapienza University, Experimental Medicine, Rome, Italy ;* [3]*University of Rome Tor Vergata, Orthopaedic Surgery, Rome, Italy ;* [4]*Sapienza University, Anatomical- Histological- Medico Legal and Orthopaedic Sciences, Rome, Italy*

**Abstract Text**

Brain derived neurotrophic factor (BDNF) is a neurotrophin regulated by the Gsα/cAMP pathway that is expressed in the central nervous system and in multiple peripheral tissues. In bone, it enhances osteogenesis and, by stimulating RANKL secretion by stromal and osteoblast cells, it also increases osteoclastogenesis. Fibrous Dysplasia (FD) of bone is a rare genetic disease caused by gain-of-function mutations of Gsα in which RANKL-dependent enhanced bone resorption is a major cause of bone fragility and clinical morbidity. We previously observed that *BDNF* was highly expressed in osteoclast-rich human FD lesions. Therefore, we hypothesized that BDNF could play a role in the pathogenesis of FD by stimulating RANKL secretion and bone resorption.
We first performed immunolocalization studies on human FD bone biopsies and observed the expression of BDNF in osteoblasts and, to a lower extent, in the pathologic fibrous tissue that characterizes the disease. We then investigated the potential pathogenetic role of BDNF in FD by using the EF1α-GsαR201C mouse model of the disease (FD mice). Western blot analysis revealed a higher expression of BDNF in bone segments of FD compared to WT mice (proBDNF p=0.011, BDNF p=0.047). Moreover, immunohistochemistry on mouse FD lesions, revealed an expression pattern of BDNF similar to that observed in human FD biopsies. Treatment of FD mice with an anti-BDNF monoclonal antibody (aBDNF) reduced the fibrous tissue (Fig.1A,C; p=0.0848) along with the number of osteoclasts (Fig.1B,D; p=0.0018) and osteoblasts (p=0.006) within mouse femoral lesions. *In vitro*, aBDNF markedly decreased the expression of osteogenic markers in bone marrow stromal cells from FD mice, suggesting that BDNF may contribute also to the abnormal osteogenesis observed in FD.
These results reveal BDNF as a new player in the pathogenesis of FD and a potential new target to reduce the abnormal bone remodeling associated with the disease.

Figure 1.

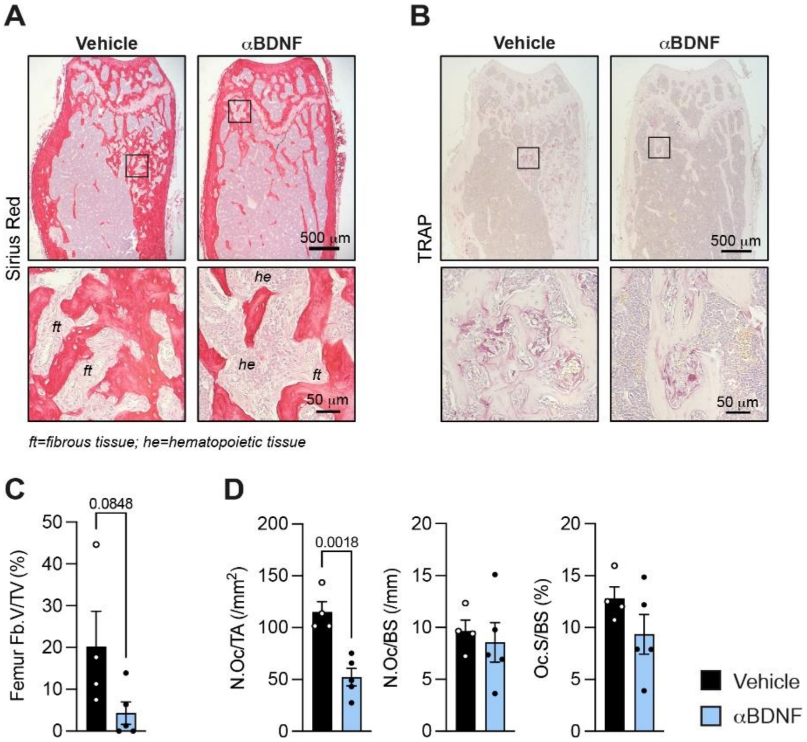

ft=fibrous tissue; he=hematopoietic tissue

**PLO04**

**Mucopolysaccharidosis type I Hurler skeletal alterations and cross-correction in an ex vivo gene therapy approach**

*Ludovica Santi[1], Stefania Crippa[1], Valentina Capo[1], Sara Penna[1], Evelyn Savoia[1], Giada De Ponti[1], Gaia Alberti[1], Margherita Berti[1], Selene Ceriotti[1], Giulia Consiglieri[2], Francesca Tucci[2], Mara Riminucci[3], Alessandro Corsi[3], Andrea Barbero[4], Ivan Martin[4], Francesca Sanvito[5], Rossana Norata[5], Martina Rocchi[5], Simona Bolamperti[6], Isabella Villa[6], Alessandro Aiuti[1], Anna Villa[1], Maria Ester Bernardo[1]*
*[1]IRCSS San Raffaele Scientific Institute, San Raffaele TIGET, Milan, Italy ; [2]IRCSS San Raffaele Scientific Institute, Pediatric Immunohematology and Bone Marrow Transplantation Unit, Milan, Italy ; [3]Sapienza University, Department of Molecular Medicine, Rome, Italy ; [4]University Hospital Basel, Tissue Engineering Department of Biomedicine, Basel, Switzerland ; [5]IRCCS San Raffaele Scientific Institute, Pathology Unit- Department of Experimental Oncology, Milan, Italy ; [6]IRCCS San Raffaele Scientific Institute, Osteoporosis and bone and mineral metabolism Unit, Milan, Italy*

**Abstract Text**

Skeletal dysplasia is an unmet clinical need in Mucopolysaccharidosis type I Hurler (MPSIH) patients as it is not fully addressed by hematopoietic stem cell transplantation (HSCT), the standard of care. Supported by preclinical findings in the MPSI murine model, 8 MPSIH patients were treated in a phase I/II clinical trial (NCT03488394) with an *ex vivo* lentiviral vector (LVV) based autologous hematopoietic stem cell gene therapy (HSC-GT) approach. As pathophysiological mechanisms of skeletal damage and cross-correction after GT are not completely understood, we investigated key players of skeletal remodeling, such as mesenchymal stromal cells (MSCs), chondrocytes (CHs), osteoblasts (OBs), and osteoclasts (OCs) obtained from MPSIH patients enrolled in the trial before and after GT. Patient-derived MSCs normally underwent intramembraneous ossification *in vitro*, although patient-derived OBs showed intracellular GAGs accumulation. Cross-correction of patient-derived OBs was feasible due to their ability to uptake IDUA enzyme when exposed to the supernatant of gene-corrected OCs. The *in vitro* scaffold-free 3D model, used to generate hypertrophic CHs, revealed a reduced expression of Collagen type X, BMP6 and Sox9 in patient-derived CHs as compared to healthy donors. The skeletal disease and correction were investigated in the MPSI murine model treated with LVV-GT. A rescue of disease-associated alterations, such as osteocytes and periosteum vacuolation, was achieved, whereas vacuolation of chondrocytes of the growth plate and the articular cartilage was improved. Trabecular bone mineral density, cortical area and strength strain index, evaluated by pQCT, were ameliorated in GT-treated mice. The istomorphometric analyses, in terms of OBs and OCs counts, the bone formation rate and the evaluation of the Achilles tendon are under evaluation. Finally, osteomedullary biopsies of MPSIH patients before GT showed alterations of the enthesis tissue which were rescued after GT. These preliminary findings suggest an early beneficial effect of HSC-GT on MPSI typical skeletal features.

**PLO05**

**Spine deformity in mice lacking Nf1 gene in boundary cap-derived osteoblasts**

*Franceska Kovaci*[1]*, Simon Perrin*[1]*, Cassandre Goachet*[1]*, Lotfi Slimani*[2]*, Françoise Tilotta*[2]*, Piotr Topilko*[1]*, Celine Colnot*[1]
[1]*INSERM- IMRB- Université Paris-Est Créteil, Biology of the Neuro-Muscular System, 94000 Creteil, France ;* [2]*Université de Paris- Life Imaging Facility- UR 2496, Pathologies- Imagerie et Biothérapies Orofaciales, 92120 Montrouge, France*

**Abstract Text**

Neurofibromatosis type 1 (NF1) is a disorder affecting 1 in 3000 people due to heterozygous mutations in the *NF1* gene. NF1 patients manifest bone abnormalities, including spine deformities, in addition to other symptoms, such as cutaneous and plexiform neurofibromas (cNFBs and pNFBs). Neurofibromas result from a second *NF1* hit mutation in Schwann cells. $Prss56^{Cre}$; $R26^{tdTom}$; $Nf1^{fl}$ (*Prss56-Nf1 KO*) mice, lacking *Nf1* gene in Prss56-expressing boundary cap (BC) cells and Schwann cell derivatives, faithfully recapitulate cNFBs, and pNFBs symptoms. Although a second *NF1* hit mutation has been reported in NF1 patients with spine deformity, the affected cell type(s) and the mechanism remain unknown. To elucidate the molecular and cellular bases in NF1 spine deformities, we explored the *Prss56-Nf1 KO* mouse model.

We used in vivo micro-CT and lineage tracing analyses from 3 until 16 months of age to characterize the spine phenotype in *Prss56-Nf1 KO* mice and the affected cell types. Micro-CT analyses showed that *Prss56-Nf1 KO* mice recapitulate scoliosis and kyphosis at 12 and 16 months of age (Figure). Spine deformity in *Prss56-Nf1 KO* mice was associated with vertebral anomalies (fusion, scalloping, wedging) as observed in NF1 patients. In vivo, fate mapping of BC-derived (tdTom+) cells revealed a significant increase of tdTom+ cells in the vertebrae of *Prss56-Nf1 KO* mice compared to control at 16 months of age and corresponding to an increase of tdtom+ osteoblasts. The increase of tdtom+ signal correlated with increased bone volume and with kyphosis spine curvature, indicating that an increase in *Nf1*-deficient osteoblasts in mutant vertebrae can lead to changes in bone parameters and subsequently to kyphosis spine deformity (Figure). Overall, our results establish a new mouse model of NF1 spine deformity, suggesting the role of Prss56+ BC cells as the cell of origin in NF1 spine deformity.

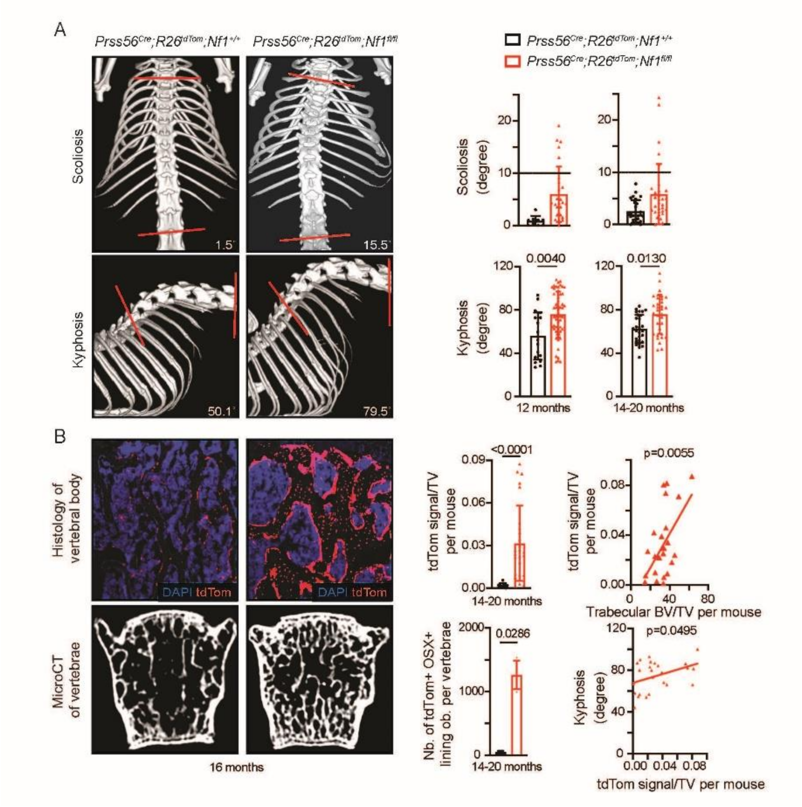

**PLO06**

**A brief vitamin B6 supplementation to individuals with low alkaline phosphatase is useful to identify patients with ALPL mutations**

*Sofía Álvarez Villalobos*[1], *Patricia Caso*[1], *Álvaro Del Real*[2,3,4], *María Teresa García-Unzueta*[2,3,4], *Gema Martín*[1], *Leyre Riancho*[5,6], *Ana Isabel Vega*[7], *José Antonio Riancho*[1,8,9,10]
*[1]Hospital Universitario Marqués de Valdecilla, Internal Medicine, Santander, Spain ; [2]Hospital Universitario Marqués de Valdecilla, Biochemistry, Santander, Spain ; [3]IDIVAL, Biochemistry, Santander, Spain ; [4]Universidad de Cantabria, Biochemistry, Santander, Spain ; [5]IDIVAL, Reumatology, Santander, Spain ; [6]Universidad de Cantabria, Reumatology, Santander, Spain ; [7]IDIVAL, Genetics, Santander, Spain ; [8]CIBERER, Internal Medicine, Santander, Spain ; [9]Universidad de Cantabria, Internal Medicine, Santander, Spain ; [10]IDIVAL, Internal Medicine, Santander, Spain*

**Abstract Text**

**Introduction:** Hypophosphatasia is a rare disorder caused by mutations in the ALPL gene, which encodes the tissue non-specific alkaline phosphatase (AP). It results in reduced serum AP activity and accumulation of its substrates, such as pyridoxal phosphate (PLP).  Diagnosis, combining biochemical assays and DNA sequencing, is sometimes complex because some patients have decreased serum AP without detectable mutations. Moreover, PLP levels can overlap between patients and normal individuals, particularly in case of limited intake of vitamin B6.

**Purpose:** To assess the PLP response to vitamin B6 supplementation in both healthy subjects and patients with low AP and suspected hypophosphatasia.

**Methods:** Serum PLP was measured with a chromatographic method (Reference Laboratory) at baseline and after a 6-day, 20 mg/day, vitamin B6 supplementation. Three groups were included: 1) healthy controls (n=15); 2) patients with hypophosphatasia and mutation in the ALPL gene (n=13); and 3) patients with low AP without detected mutations (n=12).

**Results:** Baseline PLP levels were 59±26, 200±118, and 70±36 nmol/l in groups 1, 2 and 3, respectively ($p<0.0001$). Six patients with ALPL mutations (46%) had PLP levels within the control range.
After supplementation, PLP levels were 246±87, 817±350, and 294±72 nmol/l in groups 1, 2 and 3, respectively ($p<0.0001$), with significant differences between group 2 and the other two groups ($p<0.0001$). However, there were no significant differences between groups 1 and 3. Notably, none of the individuals in these two groups exceeded 450 nmol/l after supplementation, while 85% of patients with ALPL mutations reached levels above that threshold.

**Conclusion:** The measurement of PLP levels after oral vitamin B6 supplementation emerges as a valuable tool for identifying patients with ALPL mutations. A cutoff of 450 nmol/l exhibits high sensitivity (85%) and specificity (100%), positioning it as a potential alternative to genetic testing, particularly in situations where ALPL gene sequencing is impractical.

### Plenary Oral Presentations 2: Treatment and Fracture Repair

### PLO07

### Single nuclei analyses of immune cells during bone repair reveal a crucial role of macrophages in musculoskeletal trauma

*Yasmine Hachemi[1], Simon Perrin[1], Anais Julien[2], Maria Ethel[1], Julia Vettese[1], Blandine Geisler[1], Christian Göritz[2], Céline Colnot[1]*
*[1]INSERM, Mondor institute for Biomedical research, Créteil, France ; [2]Karolinska Institutet, Department of Cell and Molecular Biology, Stockholm, Sweden*

**Abstract Text**

Musculoskeletal traumatic injuries (MTI) involve soft tissue lesions adjacent to a bone fracture leading to bone non-union and fibrotic accumulation. Successful bone repair is tied to an efficient inflammatory response, but the impact of MTI on bone regeneration and on the inflammatory response to fracture remains unknown. Using single-nuclei transcriptomic analyses, flow cytometry and immunolabeling, we described an immune cell atlas of bone regeneration and identified distinct macrophage subsets with successive pro-inflammatory, pro-repair and anti-inflammatory profiles. In a murine model of MTI, where tibia fracture is associated to adjacent muscle injury, macrophages accumulate in the fibrous tissue. Single nuclei RNAseq of the fracture callus at day 5 post-MTI showed a disruption of the immune response to fracture leading to a prolonged pro-inflammatory phase and delayed resolution of inflammation. Using constitutive (CCR2 KO) and inducible ($LysM^{Cre}$ ; $R26^{tdTom/IDTR}$) genetic approaches to deplete macrophages, we observed improved bone union in the MTI mouse model and a significant reduction in bone fibrosis demonstrating macrophage involvement in fibrous non-union. These findings reveal the crucial role of macrophages in fracture repair and trauma depicting them as a promising target for the treatment of trauma-associated fibrosis.

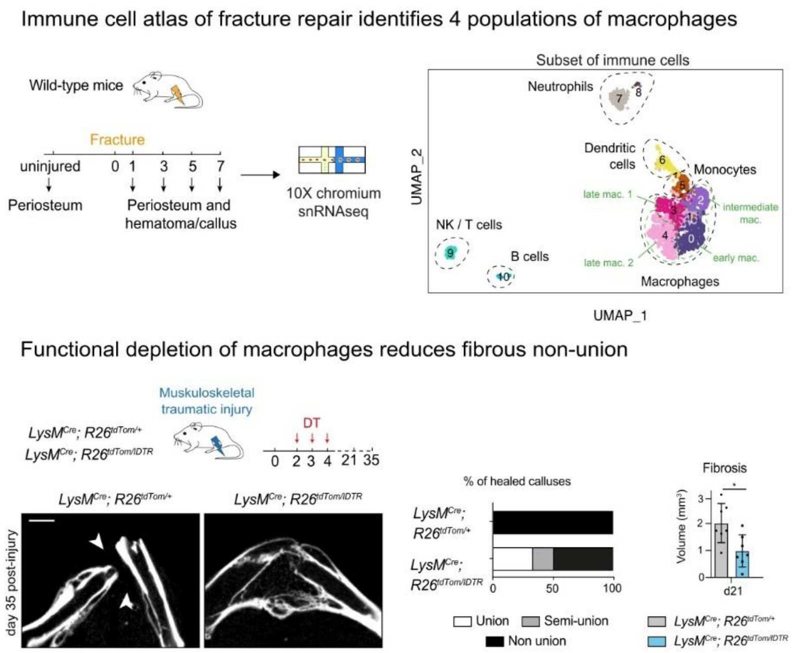

**PLO08**

**Revolutionizing Fracture Risk Prediction: Machine-Learning Synergy with Genetic Insights**

*<u>Qing Wu</u>[1], Jingyuan Dai[1]*
*[1]Ohio State University, Biomedical Informatics, Columbus, United States*

**Abstract Text**

**Introduction:** Accurate osteoporotic fracture risk prediction in postmenopausal women is crucial for optimal clinical management. Literature reports indicate the limitations of existing tools, like FRAX, for long-term risk assessment, necessitating more advanced solutions to improve patient outcomes.

**Purpose:** This study aims to revolutionize 10-year fracture risk prediction by integrating advanced machine-learning (ML) models with genetic risk scores (GRS) and FRAX clinical risk factors (CRFs), using Bayesian Optimization to set a new standard in personalized fracture risk assessment.

**Methods:** We analyzed data from 25,772 postmenopausal women in the Women's Health Initiative, dividing it into 80% for model development and 20% for validation. We developed and validated SVM, Random Forest, XGBoost, and ANN models, incorporating GRS from 1,103 SNPs and FRAX CRFs, using Bayesian Optimization and grid search. Our focus was on time-to-event data, taking into account competing risks.

**Results:** Among all ML models evaluated, the GRS-enhanced XGBoost model, optimized via Bayesian methods, emerged as the top performer in predicting 10-year fracture risk. For Major Osteoporotic Fractures (MOF), it achieved a C-index of 0.795 (95% CI: 0.783-0.806) and a mean dynamic AUC of 0.799 (95% CI: 0.788-0.809); for hip fracture, it attained a C-index of 0.699 (95% CI: 0.686-0.711) and a mean dynamic AUC of 0.703 (95% CI: 0.690-0.715), significantly surpassing the baseline Cox model (all p-values<0.0001). This novel genetics-enhanced XGBoost model, outperforming the FRAX with a 22.6% NRI in MOF predictions (p=0.004), is validated by sensitivity analysis with bone density and portability tests across diverse populations, including Asians and African Americans, underscoring its precision and broad applicability in fracture risk assessment.
**Conclusion:** Integrating ML models with genetic and clinical data significantly advances 10-year fracture risk prediction. The genetics-enhanced XGBoost model, optimized via Bayesian methods, represents a breakthrough in personalized osteoporosis management, potentially reshaping clinical guidelines and patient care strategies.

**PLO09**

**Periosteal response to bone fracture at single nuclei resolution**

*Cassandre Goachet[1], Simon Perrin[1], Maria Ethel[1], Vincent Bretegnier[1], Cécile-Aurore Wotawa[1], Marine Luka[2], Fanny Coulpier[1], Cécile Masson[3], Mickael Ménager[2], Céline Colnot[1]*
*[1]INSERM, IMRB, Créteil, France ; [2]Imagine Institue, Laboratory of Inflammatory Responses and Transcriptomic Networks in Diseases, Paris, France ; [3]Institut Imagine-Structure Fédérative de Recherche Necker, Bioinformatics Core Facility, Paris, France*

**Abstract Text**

Bone regeneration is a highly efficient repair process allowing bones to consolidate following injury. It is mediated by skeletal stem/progenitor cells (SSPCs) that are mainly recruited from the periosteum, the thin outer layer of bones. The cellular composition of the periosteum and the steps of periosteal SSPCs differentiation after bone fracture remain poorly understood. In this study, we generated single-nuclei RNAseq datasets from mouse periosteum at steady state and during the early stages of bone repair at days 3, 5 and 7 post-fracture providing an atlas of the periosteal response to bone fracture. In the uninjured periosteum, we identified periosteal SSPCs expressing stemness markers (Pi16 and Sca1). In response to fracture, periosteal SSPCs adopt an injury-induced fibrogenic cell (IIFC) fate, marked by intensive extracellular matrix production (*Col3a1, Postn, Aspn),* prior to undergoing either osteogenesis or chondrogenesis (Figure). Through gene regulatory network analysis, we identified distinct sets of regulatory genes associated with the IIFC state involving Notch, with the transition from IIFC state towards osteogenesis involving Wnt, and with the transition from IIFC state towards chondrogenesis involving the circadian clock signaling. Finally, using cell interaction analysis, we show that IIFCs are the main source of paracrine signals in the fracture environment, suggesting a crucial paracrine role of this transient IIFC population during fracture healing. Overall, our study provides a complete temporal topography of the fracture healing stages and identifies IIFCs as a new transient fibrogenic population and an essential component of the dynamic response of periosteal SSPCs to injury.

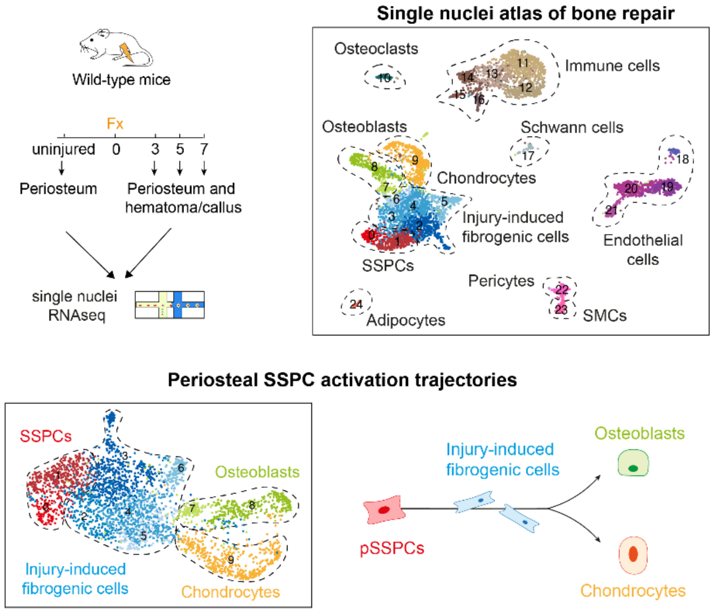

**PLO10**

**Compound heterozygous TNSALP mutations cause poorly hydrated, densely-packed mineral platelets, explaining spontaneous, osteopetrosis-like femoral fractures in hypophosphatasia**

*Scott Dillon[1], Linda Skingle[2], Adrian Murgoci[1], Mark Garton[3], Stephen McDonell[4], Phillip Johnston[5], Melinda Duer[1], Ken Poole[6]*
*[1]University of Cambridge, Chemistry, Cambridge, United Kingdom ; [2]University of Cambridge, Medicine, Cambridge, United Kingdom ; [3]Wrexham Hospital, Medicine, Wrexham, United Kingdom ; [4]University of Cambridge, Orthopaedics, Cambridge, United Kingdom ; [5]Addenbrooke's Hospital, Orthopaedics, Cambridge, United Kingdom ; [6]University of Cambridge, Department of Medicine, Cambridge, United Kingdom*

**Abstract Text**

Hypophosphatasia (HPP) is caused by genetic mutations encoding the enzyme alkaline phosphatase (Tissue Non-Specific, TNSALP). HPP is classically thought of as an ‘osteomalacia’-causing disease, due to failure to adequately mineralise bone osteoid. IIn contast, we find that patchy mineralisation defects are only part of a spectrum of bone tissue anomalies in HPP, that we explore using NMR imaging methods. We biopsied bone from unrelated HPP patients with strikingly similar ‘osteopetrotic’ femoral fracture phenotypes caused by compound heterozygous mutations in HPP. Their BMD Z-scores were >5, and both presented with bilateral spontaneous tensile mid-femoral femur fractures in early post-menopause, having never received bisphosphonates.

Trans iliac bone biopsies were collected from the compound heterozygous HPP patients, X-linked hypophosphatemia (XLH) patients and controls, and embedded in LR White resin. Backscattered scanning electron microscopy (BSE-SEM) was used to investigate. Serial sections were cut from biopsies and subjected to solid state nuclear magnetic resonance (ssNMR) experiments. Ultrathin sections were examined by high-resolution transmission electron microscopy (HR-TEM) to investigate bone mineral nanostructure.

BSE-SEM revealed large accumulations of unmineralised osteoid on bone surfaces accompanied by islands of calcification remote from the surface, along with a reduction in the number of osteocyte lacunae in HPP patients compared to controls. 1D $^{31}P$ and 2D $^{1}H$-$^{31}P$ ssNMR spectra identified an extremely crystalline mineral phase in HPP patients, with a loss of the inter-platelet hydrated phase present in healthy controls, as compared to XLH samples which retained this layer but exhibited an accumulation of amorphous $HPO_4$ phases (Figure 1). This was confirmed by HR-TEM, revealing extremely large and densely packed mineral platelets in HPP samples compared to controls.

High bone density and propensity for brittle fracture in compound heterozygous HPP patients is driven by dysregulated nanoscale bone mineral chemistry, with patients exhibiting significantly purer and more crystalline bone mineral with impaired mechanical properties.

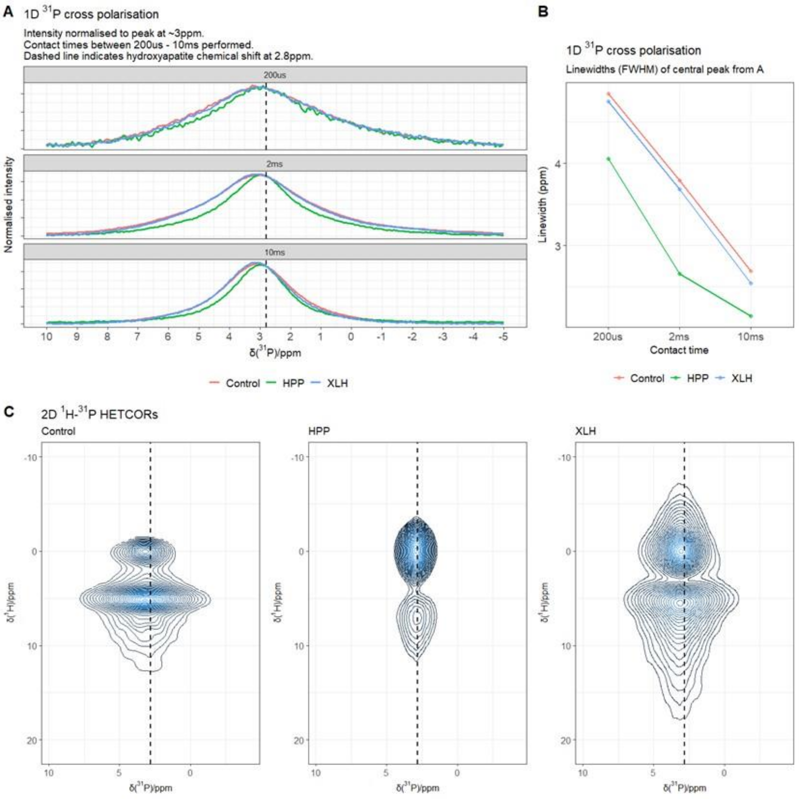

**PLO11**

**Spatial transcriptomics reveal distinct gene expression patterns in non-union and union bone fractures in mice**

*Maria Schröder[1], Nico Giger[1], Jan Barcik[2], Lena Gens[3], Daniel Arens[3], Dominic Gehweiler[2], Peter Varga[2], Stephan Zeiter[3], Martin Stoddart[1], Esther Wehrle[1]*
*[1]AO Research Institute Davos, Regenerative Orthopaedics, Davos Platz, Switzerland ; [2]AO Research Institute Davos, Biomedical Development, Davos Platz, Switzerland ; [3]AO Research Institute Davos, Preclinical Services, Davos Platz, Switzerland*

**Abstract Text**

**Background/Introduction:** Non-union bone fractures remain a challenging clinical problem. Combining novel omics technologies with established .and mechanically-controlled preclinical animal models have the potential to capture and understand underlying biological changes during fracture healing.

**Purpose:** This study applies spatial transcriptomics (ST) to characterize musculoskeletal gene expression patterns in non-union and union fractures in mice.

**Methods:** Female 20-week old C57BL/6J mice received a femur osteotomy (0.7mm) or a segmental defect (2.4mm) stabilized with an external fixator (n=14/group; license 22/2022, Grisons CH). To monitor healing progression, *in vivo* micro-CT and *in vivo* stiffness measurements were performed on days 0, 3, 7, 10, 14, 21, 28 and 49 (statistics with GraphPadPrism10). Mice were euthanized at postoperative days 3, 7, 14, 21 (all n=3/group) and 49 (n=2/group). ST was performed from formalin-fixed paraffin-embedded tissue samples on day 14 (n=2 per group; Visium CytAssist FFPE; 10x Genomics). Sequencing data was processed in SpaceRanger with downstream statistical analyses/visualization in R using Seurat and Bioconductor packages.

**Results:** *In vivo* monitoring via micro-CT and stiffness measurements indicated bony bridging of osteotomies by d21 (0.029N/µm±0.004) whereas segmental defects (0.005N/µm±0.009) remained unbridged throughout the study ($p<0.01$, unpaired t-test+Welch's correction). Histology and ST (d14) substantiated this finding with distinct healing-associated gene expression patterns (Fig.1). In unions, strong expression of genes involved endochondral ossification, mineralization and remodelling were seen in the callus region (cluster 5; e.g. *Dmp1*, *Acp5*). Non-unions displayed high marrow adiposity adjacent to the defect site (cluster 9) largely displacing the bone marrow clusters seen in unions (clusters 4+8). Interestingly, ST captured markers predominantly expressed in non-unions, in the defect region (cluster 2; e.g. *Postn, Fstl1, Sfrp2*) and the adjacent bone marrow (cluster 9; e.g. *Scd1*), indicating potential treatment targets.

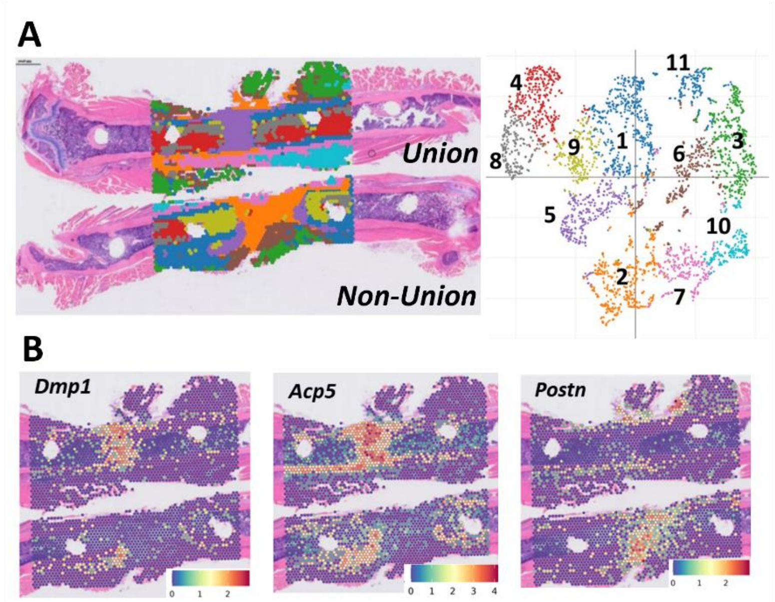

**Figure 1.** Spatial transcriptomics of bone unions and non-unions in mouse femur defect models. A: H&E and clustering, t-SNE projection. B: Spatial distribution of molecular markers.

**Conclusion(s):** ST comprehensively characterizes musculoskeletal gene expression patterns in non-union and union fractures relevant for identification of targets for individualized treatments to improve healing.

**PLO12**

### Identification of a rare heterozygous variant in WDR5 associated with high bone mass

*Neelam Hassan[1], Marc Van der Kamp[2], Celia Gregson[1], Emma Duncan[3], Jon Tobias[1]*
*[1]University of Bristol, Musculoskeletal Research Unit, Bristol, United Kingdom ; [2]University of Bristol, School of Biochemistry, Bristol, United Kingdom ; [3]King's College London, Department of Twin Research & Genetic Epidemiology, London, United Kingdom*

### Abstract Text

**Introduction:** We aimed to identify novel monogenic causes of unexplained high bone mass (HBM).

**Methods:** Whole exome sequencing (WES) was undertaken in 23 UK HBM pedigrees (proband: Z-score of ≥+3.2 at total hip (TH) or first lumbar vertebra ($L_1$); affection status for other individuals: summed Z-score (TH + $L_1$) of ≥+3.2) Data were analysed for carriage of novel or rare (MAF<0.005) nonsynonymous single nucleotide variants or indels in highly conserved gene regions predicted to be damaging by at least one protein prediction algorithm, segregating with HBM. WES data from 493 low bone mass cases in the Anglo-Australasian Osteoporosis Genetics Consortium (hip Z-Score <-1.5) were analysed to ensure absence of variants. Protein homology modelling was conducted using the SwissModel server.

**Results:** A rare (MAF 0.000008) heterozygous missense variant in *WDR5* (GRCh37/hg19 NM_017588.3:c.854T>C, p.Ile285Thr) segregated with HBM in five affected family members (four affected siblings and child of one affected person; probability of random segregation with affection status = 0.03125).

In addition to HBM (Table 1), no individual had ever fractured; four reported difficulty floating when swimming; and two had tori.

Data from animal models are limited but have previously indicated that WDR5 regulates osteoblast differentiation. Protein modelling showed that p.Ile285 is located in a hydrophobic pocket, close to the WDR5-binding motif (WBM) site (Figure 1). Substituting hydrophobic isoleucine with hydrophilic threonine is predicted to cause instability, indirectly affecting substrate binding to the WBM site.

**Conclusion:** *WDR5* variants may cause autosomal dominant HBM.

**Table 1**

| | HBM pedigree, WDR5 c.854T>C p.Ile285Thr | | | | |
|---|---|---|---|---|---|
| | **P1** | **P2** | **P3** | **P4** | **P5** |
| Age | 76 | 74 | 70 | 68 | 43 |
| Sex | Male | Female | Male | Female | Female |
| Height (cm) | 171 | 164 | 173 | 158 | 165 |
| Weight (kg) | 96 | 80 | 96 | 76 | 78 |
| BMI (kg/m²) | 33 | 30 | 32 | 30 | 29 |
| L1 Z-score | +5.2 | +3.5 | +3.0 | +5.5 | +1.7 |
| TH Z-score | +2.3 | +3.4 | +2.2 | +1.3 | +1.8 |

Figure 1: (A) Overview of the WDR5 structure in complex with dimethylated H3K4 peptide in cyan, bound to the WBM site (PDB ID: 2O9K). The Ile285 residue is highlighted in orange. (B) Ile285 residue within the hydrophobic pocket (grey surface) formed by the surrounding residues.

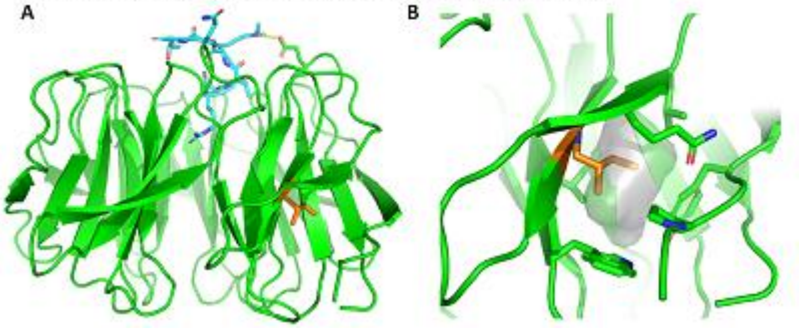

**Plenary Oral Presentations 3: Innovative Concepts**

**PLO13**

**CRISPR-toolbox for identification and validation of bone-specific enhancers**

*<u>Regina Margarete Grün</u>[1], Salaheddine Ali[2], Uwe Kornak[3]*
*[1]University Medical Center Göttingen, Institut of Human Genetics, Göttingen, Germany ; [2]Universitätsmedizin Berlin, Institute for Medical Genetics and Human Genetics- Charité, Berlin, Germany ; [3]University Medical Center Göttingen, Institute of Human Genetics, Göttingen, Germany*

**Abstract Text**

**Background:** Only 1.5 % of the human genome encodes for proteins. The remaining 98.5 % contains important regulatory elements which are still mostly uncharacterized. Enhancers spatiotemporally control gene expression over large genomic distances and thereby regulate cell differentiation and tissue development. Due to the regulation over large genomic distances interpretation of the role of enhancers is challenging. We developed a database by analyzing enhancer patterns of osteoclasts, osteoblasts and their respective precursors using ATAC-seq, HiC-seq and ChIP-seq and found that around 2 % of the genome comprise potential regulatory sequences.

**Purpose:** We developed a CRISPR/Cas based iPSC (induced pluripotent stem cells) model for characterization and functional analyses of putative bone-specific enhancers.

**Methods:** We targeted putative enhancer regions using CRISPRa-iPSCs, a CRISPR gene activation system in iPSCs (Fig. B). Cells were transfected by nucleofection with in average three sgRNAs (single guide RNAs) per region. Effects on gene expression were evaluated by qRT-PCR.

**Results:** We identified an up to 100-fold increase in *SOST* gene expression by targeting three putative *SOST* enhancers (Fig. C top, p-value=0.0013). Whereby, the most distal region Enh5 (approx. 60 kb distal) had the most impact on *SOST* expression compared to the more proximal regions Enh4 and proximal Enh. Expression of the neighboring gene *DUSP3* was not changed upon targeting any of the selected regions (Fig. C bottom).

**Conclusion:** Our model is a powerful tool for characterization and validation of bone-specific enhancers. Deletion of region Enh5, which had the highest impact on *SOST* gene expression in our experiments, is known to cause Van Buchem-disease, a sclerosing bone dysplasia characterized by osteoblast-driven progressive bone growth. Identifying the regulatory networks of bone cells is not only important for a better understanding of bone cell differentiation and maturation but is also crucial for genome-wide genetic diagnostics.

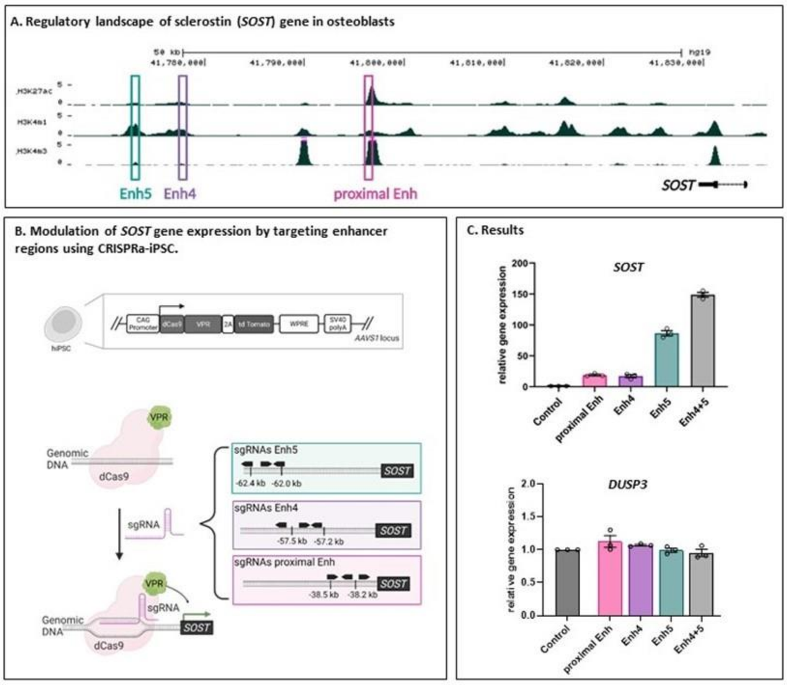

**PLO14**

**Improved skeletal dynamics in adults treated with palopegteriparatide (TransCon PTH) for hypoparathyroidism: 52-week analysis of phase 3 PaTHway trial**

*Aliya Khan[1], Mishaela R. Rubin[2], Dolores M. Shoback[3], Peter Schwarz[4], Lynn Kohlmeier[5], Andrea Palermo[6], Bart L. Clarke[7], Erik Eriksen[8], Elena Tsourdi[9], Filomena Cetani[10], Rajesh Jain[11], Carol Zhao[12], Bryant Lai[13], Jenny Ukena[13], Christopher T. Sibley[13], Michael Ominsky[14], Aimee D. Shu[13], Lars Rejnmark[15]*
*[1]McMaster University, Divisions of Endocrinology and Metabolism and Geriatrics, Hamilton, Canada ; [2]Columbia University, Endocrinology, New York, United States ; [3]UCSF/VA Medical Center, School of Medicine, San Francisco, United States ; [4]Rigshospitalet, Department of Endocrinology, Copenhagen, Denmark ; [5]Endocrinology and Spokane Osteoporosis, Endocrinology, Spokane, United States ; [6]Fondazione Policlinico Campus Bio-medico and Campus Bio-medico University, Unit of Endocrinology and Diabetes- Department of Medicine, Rome, Italy ; [7]Mayo Clinic E18-A, Endocrinology, Rochester, United States ; [8]Oslo University Hospital, Endocrinology, Oslo, Norway ; [9]Technische Universität Dresden Medical Center, Department of Medicine III and Center for Healthy Aging, Dresden, Germany ; [10]University Hospital of Pisa, Endocrinology, Pisa, Italy ; [11]University of Chicago, Endocrinology, Chicago, United States ; [12]Ascendis Pharma Inc, Medical Affairs Statistical Sciences, Palo Alto, United States ; [13]Ascendis Pharma Inc, Clinical Development- Endocrine Medical Sciences, Palo Alto, United States ; [14]Ascendis Pharma Inc, Skeletal Sciences- Clinical Development, Palo Alto, United States ; [15]Aarhus University Hospital, Department of Clinical Medicine - The Department of Endocrinology and Diabetes, Aarhus, Denmark*

**Abstract Text**

**Background:** Hypoparathyroidism is associated with reduced bone turnover and high bone mineral density (BMD).

**Purpose:** Evaluate the temporal changes in and relationship between skeletal endpoints with palopegteriparatide (TransCon PTH), a prodrug with sustained release of PTH(1-34), in individuals with chronic hypoparathyroidism.

**Methods:** PaTHway is a phase 3 trial of palopegteriparatide with a randomized, placebo-controlled 26-week blinded period and 156-week open-label extension. Serum bone turnover markers procollagen type 1 N-terminal propeptide (P1NP) and C-telopeptide (CTx) were assessed at baseline and weeks 12, 26, 38, and 52. BMD was assessed by DXA at the lumbar spine, total hip, and distal 1/3 radius at baseline, weeks 26 and 52. Results are reported for participants randomized to palopegteriparatide with week 52 data (n=59).

**Results:** At baseline, participants had a mean (SD) age of 49 (13) years; 75% were female, 41% of whom were postmenopausal. Through week 52, 81% (48/59) maintained normocalcemia (8.3-10.6 mg/dL) and independence from conventional therapy (≤600 mg/day elemental calcium and no active vitamin D). Palopegteriparatide treatment initially increased CTx and P1NP; responses peaked by week 12 and 26, respectively, then declined through week 52 (Figure). With 52 weeks of palopegteriparatide treatment, elevated mean baseline BMD Z-scores and T-scores declined toward age- and/or sex-matched norms at the lumbar spine and total hip, with a smaller magnitude of change from week 26 to 52 (Figure). Changes in BMD at the spine and hip were consistent across age, sex, and postmenopausal status, and at week 26 were inversely correlated with changes in P1NP and CTx at week 12 (R: -0.23 to -0.48).

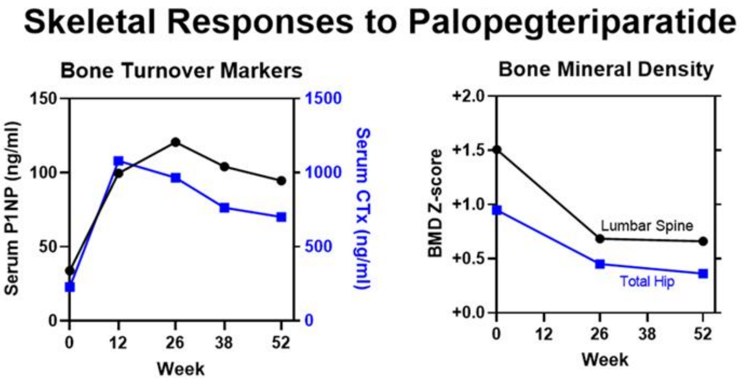

**Conclusions:** Palopegteriparatide treatment resulted in mobilization of calcium from the low bone turnover state in adults with hypoparathyroidism. These 52-week results reflect temporal changes trending toward a new skeletal steady state closer to age-appropriate norms with continued use of palopegteriparatide in hypoparathyroidism.

**PLO15**

**Mapping osteoblastic bone formation in vivo at the single organelle resolution**

Kai Chen[1], Jinyu Guo[2], Xiaojun Chen[1], Nathan Pavlos[3], Haibo Jiang[4]
[1]The University of Western Australia, School of Molecular Sciences, Perth, Australia ; [2]The University of Hong Kong, Department of Chemistry, Hong Kong, China ; [3]The University of Western Australia, School of Biomedical Sciences, Perth, Australia ; [4]University of Hong Kong, Department of Chemistry, Hong Kong, China

**Abstract Text**

**Background:** Amino acids are "building blocks" for osteoblasts to synthesize new bone. Understanding osteoblast biology requires the spatial mapping of how amino acids are incorporated into intracellular organelles and subsequent extracellular matrix (ECM) across time scales, in their native microenvironment. Therefore, spatial technology enabling the *in vivo*, organelle-level metabolic profiling is essential but remains a long-standing technical challenge.

**Methods:** To achieve this, stable isotope-labeled amino acids (i.e.,$^{15}N$-) were first orally given to the young and aging mice (4- and 18-month-old). Bone samples were collected at different time points spanning from weeks to mere minutes. Electron microscopy (EM) provided the ultrastructure of cells and ECM. The same area of interest was sequentially imaged by ion microscopy (IM), using the nanoscale secondary ion mass spectrometry (NanoSIMS) technique, creating images of secondary ions of biological elements and isotopes (e.g., $^{14}N^-$, $^{15}N^-$) at ~40 nm lateral resolution. Quantitative analysis of $^{15}N/^{14}N$ ratios mapped the dynamic amino acid distributions and accumulations in subcellular compartments and ECM.

**Results:** By spatiotemporally tracing the metabolic flux of amino acids, we profiled the osteoblastic bone formation *in vivo*. Within just 10 mins of oral intake, dietary $^{15}N$-amino acids were observed in osteoblast endoplasmic reticulum, Golgi apparatus, and secretory granules, with the latter exhibiting the highest $^{15}N/^{14}N$ ratio. Osteoblasts rapidly secreted the new bone (osteoid) within 30 minutes. By 2 h, most dietary amino acids were converted to osteoid. Following 1 day, 7 days, and 3 weeks of ingestion, amino acids were incorporated into region-specific matrix renewal. Moreover, we demonstrated the decline of amino acid metabolism and ECM renewal during the aging process.

**Conclusion:** We have developed a first-in-kind metabolic imaging platform, allowing the quantitative visualization of intra- and extracellular amino acid handling by bone-resident osteoblasts at the nanoscale, providing metabolic insights for the musculoskeletal community and beyond.

**PLO16**

**Acute effects of GLP-1 and GIP on human bone turnover**

Nina Hansen[1], Morten Svarer Hansen[1], Bolette Hartmann[2], Morten Frost[1,3]
[1]University of Southern Denmark, Molecular Endocrinology Department- KMEB, Odense, Denmark ; [2]Department of Biomedical science, University of Copenhagen, Denmark ; [3]Steno Diabetes Centre Odense, Denmark,

**Abstract Text**

**Background:** Incretin hormones are widely and increasingly used to treat obesity and T2D, both associated with changes in fracture pattern and fracture risk. While short-term infusion of GLP-1 and GIP decreases biochemical markers of bone resorption in peripheral blood (PB), GIP is suggested to transiently increase bone formation markers whereas GLP-1 has neutral effects on bone formation markers. However, the effect of GIP and GLP-1 in bone marrow (BM) is unknown.

**Purpose:** We hypothesized that a short-term infusion of GIP and GLP-1 a) would demonstrate that changes in bone resorption measured in plasma from PB reflects changes in the BM, b) would show differential effects on bone formation markers, without c) affecting differentiation capacity *in vitro*.

**Methods:** Following ethical approval 12 healthy male subjects (22-43 years) underwent 2-hour infusions of either GIP, GLP-1 or the combination on three separate study days. Glucose was infused to mimic a postprandial glucose level of 8 mM. PB samples were collected every 30 minutes (-30, 0, 30, 60, 90, 120 min.). Iliac crest BM-aspirates were collected at baseline and post-intervention. Bone turnover markers, P1NP and CTX were measured in BM-plasma and in PB. Skeletal stem cells were isolated and differentiated to osteoblast and adipocytes to study differentiation capacity.

**Results:** GIP and GLP-1 potently decreased CTX both in PB (38% and 34%, respectively (Fig.2A)) and BM (84% and 78%, respectively (Fig. 1A)). P1NP increased (10%) transiently after 30 minutes in the PB (Fig.2B) and was (34%) higher after 2 hours in the BM (Fig.1B). GLP-1 exerted no effect on P1NP. Neither GIP nor GLP-1 affected cellular differentiation measured by Alp-activity and by Nile-Red (Fig.3).

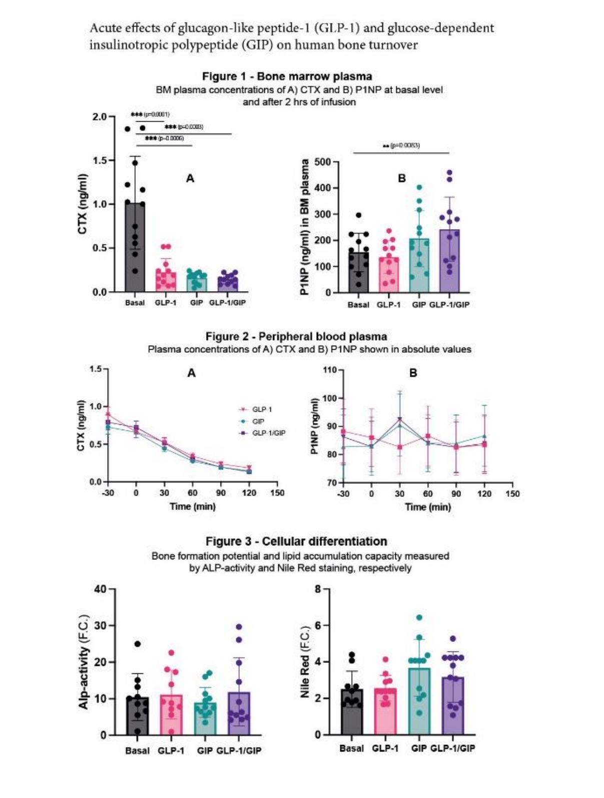

**Conclusion:** Incretin hormones have differential effects on bone turnover markers. Both exert acute anti-resorptive effects, while GIP demonstrate a transient effect on bone formation. Mechanisms behind differential effects of incretin hormones on bone turnover remain to be established.

### PLO17

### Single cell sequencing reveals a novel osteoblast-derived cell population supporting breast cancer bone metastasis

*Jennifer Zarrer[1,2,3], Marie-Therese Haider[3], Nicole Ridlmaier[3], Rafael Preuer[3], Nichole Pederson[4,5], Daniel J. Smit[6], Hiroaki Saito[1,2], Christina M. Andreasen[4,5], Eric Hesse[1,2], Hanna Taipaleenmäki[1,2,3]*
*[1]University Hospital- LMU Munich, Institute of Musculoskeletal Medicine, Munich, Germany*
*; [2]University Hospital- LMU Munich, Musculoskeletal University Center Munich, Munich, Germany*
*; [3]University Medical Center Hamburg-Eppendorf, Molecular Skeletal Biology Laboratory- Department of Trauma- Hand and Reconstructive Surgery, Hamburg, Germany ; [4]University of Southern Denmark, Molecular Bone Histology lab- Research Unit of Pathology, Odense, Denmark ; [5]Odense University Hospital, Department of Pathology, Odense, Denmark ; [6]University Medical Center Hamburg-Eppendorf, Institute of Biochemistry and Signal Transduction, Hamburg, Germany*

**Abstract Text**

In breast cancer bone metastases, the abundance of osteoblasts is diminished. To determine the fate of osteoblasts, we combined lineage-tracing (*Osx-cre-ERt⁺;Ai9*) with intracardiac injection of breast cancer cells and performed single cell RNA sequencing (scRNAseq) of tdTomato-positive (tdTom⁺) osteoblasts in metastases-bearing and healthy animals. Bioinformatic analysis revealed a distinct cluster of tdTom⁺ osteoblasts in metastases-bearing mice expressing genes related to tumor microenvironment and cancer-associated fibroblast (CAF) proliferation (e.g. *Cxcl12, Pdgfr-α,-β*). CAFs reside inside tumors providing a micro-milieu that supports tumor growth. However, little is known about metastasis-associated fibroblasts, particularly in bone metastases. Based on our findings, we hypothesized that osteoblasts might abandon bone surfaces and become bone metastasis-associated fibroblasts (BMAFs). Indeed, histological analysis demonstrated elongated tdTom⁺ osteoblasts within metastases ($p<0.05$), while only few osteoblasts occupied nearby bone surfaces. Consistently, in human bone metastases biopsies immunohistochemistry and *in situ* hybridization revealed elongated Runx2-positive osteoblasts within tumors. *In vitro*, MC3T3-E1- and primary osteoblasts acquired a spindle shape morphology upon stimulation with 4T1 or MDA-MB-231 breast cancer cell conditioned medium (CCM), which also increased osteoblast migration and osteoblast-induced collagen remodeling ($p<0.05$). Immunoblot, qRT-PCR, scRNAseq and histological analyses revealed a reduction of osteoblast markers (*Runx2, Osx*) and an increase of BMAF-associated genes (e.g. *Pdgfr-α,-β*) in CCM-stimulated- and cancer-associated tdTom⁺ osteoblasts ($p<0.05$). To elucidate the mechanisms transitioning osteoblasts to BMAFs, we performed RNA sequencing and identified IL-17A signaling activation in CCM-stimulated osteoblasts, which was confirmed by immunoblot and qRT-PCR analyses of downstream targets (*C/ebp, Ccl2*). Furthermore, recombinant IL-17A promoted osteoblast migration and acquisition of a BMAF phenotype while anti-IL-17 antibody restored the CCM-induced morphological changes and osteoblast migration ($p<0.05$). *In vivo*, anti-IL-17A reduced the number and growth of bone metastases determined by bioluminescence imaging and histology ($p<0.05$). Together, these results demonstrate that osteoblasts become BMAFs at least in part through IL-17A, contributing to metastatic growth.

**PLO18**

**Zoledronate after denosumab discontinuation: is repeated administrations more effective than single infusion in preserving bone mineral density?**

*Giorgia Grassi[1], Alberto Ghielmetti[2], Marta Zampogna[2], Iacopo Chiodini[3], Maura Arosio[2], Giovanna Mantovani[2], Cristina Eller Vainicher[4]*
*[1]Fondazione IRCCS Ca' Granda-Ospedale Maggiore Policlinico, Endocrine Unit, Milano, Italy*
*; [2]University of Milan, Department of Clinical Sciences and Community Health, Milan, Italy*
*; [3]University of Milan, Department of Medical Biotechnology and Translational Medicine, Milan, Italy*
*; [4]Fondazione IRCCS Ca' Granda-Ospedale Maggiore Policlinico, Endocrine Unit, Milan, Italy*

**Abstract Text**

**Background:** After Denosumab (Dmab) discontinuation the C-terminal telopeptide (CTX) levels increase, the bone mineral density (BMD) is lost and multiple vertebral fractures (FX) may occur. A sequential therapy with bisphosphonates is recommended to mitigate this so called "rebound phenomenon". In 2020, the European Calcified Tissue Society (ECTS) proposed to repeat zoledronate (ZOL) administration in patients with persistently high CTX, although the efficacy of this schedule has not been clearly proved.

**Purpose:** In this retrospective study we describe the BMD changes and the incidence of FX in 52 patients discontinuing Dmab, who have been managed according to the ECTS recommendations. Ethical approval was obtained from the ethic committee Lombardia 3, Milan.

**Methods:** CTX levels were measured and ZOL was administrated after one month of Dmab washout (t0). We reassessed CTX 6 months later (t1) and patients with CTX levels ≥280 ng/L underwent a second ZOL infusion. The BMD changes and the incidence of FX were assessed after 17 months on average.

**Results:** 75% of patients had to repeat ZOL. In this group spine BMD declined significantly (-5.5±5.6%) whereas it remained stable in the group with CTX levels at t1 <280 ng/L (-0.1±5.5%, p=0.008) (Figure 1). The overall FX rate was 9.6%. All fractured patients had been treated with >5 Dmab injections and received two ZOL infusions. The BMD worsening after Dmab withdrawal was significantly associated with CTX t1 (OR 2.9, IQR 1.3-6.6, p=0.009) and spine BMD gain during Dmab therapy corrected for the number of Dmab injections (OR 3.0, IQR 1.2-7.2, p=0.014). A CTX level at t1 >212 ng/L had 100% sensitivity in predicting the BMD loss.

**Conclusions:** In patients discontinuing Dmab who have uncontrolled CTX levels at 6 months after the first ZOL infusion, a second ZOL dose does not completely prevent BMD loss and rebound FX.

**Figure 1. The BMD changes during Denosumab therapy and after discontinuation in all patients and in the group treated with singlezoledronate or in patients who underwent a second zoledronatedose 6 months later.**

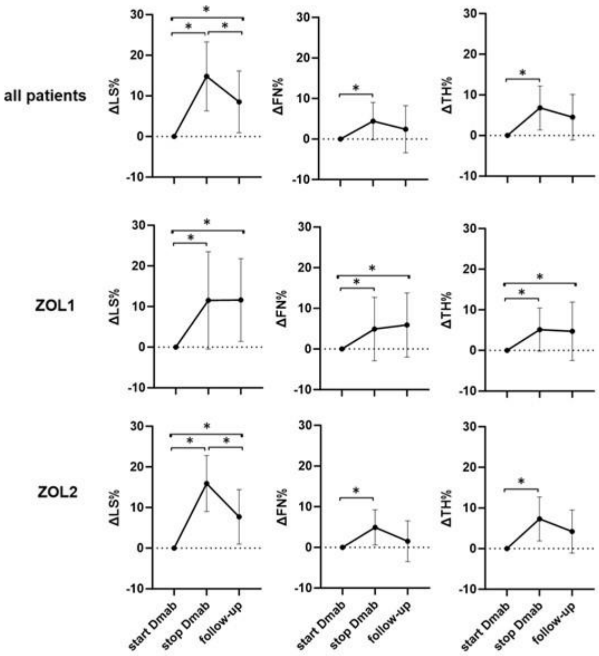

*Percent changes (mean ± S.D.) during denosumab treatment and after denosumab discontinuation in lumbar spine (LS), femoral neck (FN), total hip (TH). ZOL 1 = patients treated with single ZOL infusion after Dmab. ZOL 2 = patients treated with 2 ZOL infusion after Dmab. *ΔBMD>LSC.*

**Concurrent Oral Presentations**

**Concurrent Oral Presentations 1: Rare Bone Diseases**

**COP01**

**Possible role of bone turnover markers in the diagnosis of Adult Hypophosphatasia**

*Marco Onofrio Torres*[1], *Gaetano Paride Arcidiacono*[1], *Francesco Bertoldo*[2], *Giovanni Giovanni Tripepi*[3], *Martina Zaninotto*[4], *Mario Plebani*[4], *Alfredo Scillitani*[5], *Massimo Varenna*[6], *Chiara Crotti*[6], *Cristiana Cipriani*[7], *Jessica Pepe*[7], *Salvatore Minisola*[7], *Flavia Pugliese*[5], *Vito Guarnieri*[8], *Valeria Baffa*[1], *Francesca Zanchetta*[1], *Maria Fusaro*[9], *Maurizio Rossini*[10], *Maria Luisa Brandi*[11], *Stefania Sella*[1], *Paolo Simioni*[1], *Sandro Giannini*[1]

*[1]University of Padua, Clinica Medica 1- Department of Medicine, Padua, Italy ; [2]University of Verona, Bone Metabolism and Osteoncology Unit, Verona, Italy ; [3]Ospedali Riuniti, National Research Council CNR- Institute of Clinical Physiology IFC- Clinical Epidemiology of Renal Diseases and Hypertension, Reggio Calabria, Italy ; [4]University of Padua, Laboratory Medicine Unit- Department of Medicine, Padua, Italy ; [5]"Casa Sollievo della Sofferenza" Hospital- IRCCS, Unit of Endocrinology and Diabetology, San Giovanni Rotondo, Italy ; [6]"Gaetano Pini" Institute, Bone Diseases Unit- Department of Rheumatology, Milan, Italy ; [7]Sapienza University of Rome, Department of Clinical- Internal- Anaesthesiology- and Cardiovascular Sciences, Rome, Italy ; [8]"Casa Sollievo della Sofferenza" Hospital- IRCCS, Division of Medical Genetics, San Giovanni Rotondo, Italy ; [9]National Research Council CNR, Institute of Clinical Physiology IFC, Pisa, Italy ; [10]University of Verona, Rheumatology Unit- Department of Medicine, Verona, Italy ; [11]F.I.R.M.O. Foundation, Italian Foundation for the Research on Bone Diseases, Florence, Italy*

**Abstract Text**

**Introduction**: Hypophosphatasia (HPP) is a rare inherited disorder of bone metabolism, characterized by low serum alkaline phosphatase (ALP). However, comparable ALP levels may be observed in osteoporotic patients previously treated with potent antiresorptive drugs.

**Purpose**: the aim of this study was to verify if differences in well-defined bone turnover markers (BTM) may help to distinguish patients with Adult HPP from osteoporotic patients already undergoing antiresorptive therapy.

**Methods**: In this multicenter observational retrospective study, we enrolled adult patients with a confirmed diagnosis of HPP (Group 1). Thereafter, we collected a pool of osteoporotic patients with serum ALP < 50 U/L, documented within 12 months after the use of intravenous zoledronic acid 5 mg or 6 months after subcutaneous denosumab 60 mg (Group 2). BTM, such as C-terminal telopeptide of Type I collagen (CTx), procollagen type 1 amino-terminal propeptide (P1NP), total ALP and bone ALP isoenzyme (bALP), were assayed in all patients.

**Results**: A total of 92 patients were included; 23 of them were classified in Group 1 and 69 in Group 2. We observed that HPP patients compared with controls had higher CTx ($p<0.001$) and P1NP ($p<0.001$) levels and lower ALP activity ($p<0.001$). All the following BTM ratios were significantly lower in HPP patients as compared to control subjects: ALP/P1NP ratio ($0.62\pm0.46$ vs $2.86\pm1.35$, $p<0.001$), bALP/P1NP ratio ($0.11\pm0.07$ vs $0.44\pm0.28$, $p<0.001$), ALP/CTx ratio ($0.19\pm0.18$ vs $0.88\pm0.54$, $p<0.001$) and bALP/CTx ratio ($0.04\pm0.03$ vs $0.12\pm0.08$, $p<0.001$). In addition, in a ROC curve analysis, we observed that ALP/P1NP best performed in discriminating between HPP and control patients (AUC 0.977, $p<0.001$), with an optimal cut-off value of 1.114.

**Conclusions**: In the setting of low serum ALP levels, BTM ratios may be useful to distinguish between subjects with HPP and osteoporotic patients treated with antiresorptive drugs, thus improving the diagnostic process for HPP.

**COP02**

**Exploring the interplay of genetics, epigenetics, gene expression, and biochemistry in individuals with suspected adult hypophosphatasia.**

*Alvaro Del Real[1], Sofía Álvarez-Villalobos[2], Carolina Sañudo[1], Ana I. Vega[3], Joan Canal[2], Juan Ansede[4], Leyre Riancho-Zarrabeitia[5], Jose A Riancho[6]*
*[1]Universidad de Cantabria- IDIVAL, Medicina y Psiquiatría, Santander, Spain ; [2]Hospital U.M. Valdecilla, Servicio de Medicina Interna, Santander, Spain ; [3]Hospital U.M. Valdecilla, Servicio de Genética, Santander, Spain ; [4]Centro Nacional de Genotipado CEGEN- Universidade de Santiago de Compostela, Genética, Santiago de Compostela, Spain ; [5]Hospital U Sierrallana- IDIVAL, Servicio de Reumatología, Torrelavega, Spain ; [6]Hospital U.M. Valdecilla- Universidad de Cantabria- IDIVAL-CIBERER, Servicio de Medicina Interna, Santander, Spain*

**Abstract Text**

**Background:** Hypophosphatasia (HPP) manifests as reduced alkaline phosphatase (ALP) levels in serum and various abnormalities in mineralized tissues. In cases with unexplained low ALP levels and no identified pathogenic variants in ALPL, the gene encoding ALP, the diagnosis becomes uncertain.

**Purpose:** To search deeper into the mechanisms behind low ALP in such cases, we conducted a study involving 26 patients exhibiting low ALP levels of presumably genetic origin, and a control group of 15 healthy individuals.

**Methods:** Peripheral blood samples were collected. We used NGS to analyze ALPL gene. Promoter regions were studied by Sanger sequencing; gene expression, by RT-PCR with Taqman probes; and promoter methylation by MALDI-TOF (Epityper).

**Results:** NGS revealed pathogenic/likely pathogenic variants in 14 patients, while 12 showed no mutations. Carriers of genetic variants exhibited significantly lower serum ALP levels than non-carriers (20.5±5.6 and 31.2±5.9, respectively; p=0.0001). However, bone-specific ALP (BSALP) levels were similar in the two patient groups (8.2±4.3 and 10.2±3.1, respectively; p=0.19). Intriguingly, total ALP and BSALP levels were correlated in controls and in patients with pathogenic variants (p=0.0002 and p=0.006, respectively), but not in non-carriers (p=0.79). Sequencing the promoter region yielded no relevant variants, and methylation analysis indicated near-complete unmethylation, without between-group differences. While ALPL transcript abundance was slightly higher in the non-carrier group, no significant differences were observed among the three groups (p=0.13). Additionally, no apparent correlation existed between gene expression levels and serum ALP levels in any group.

**Conclusions:** Our findings eliminate proximal promoter region variants, changes in promoter methylation, and reduced transcription as causes for low ALP levels in patients without ALPL coding region mutations. The absence of significant differences in BSALP between carriers and non-carriers, along with the lack of correlation between total ALP and BSALP in non-carriers, would be consistent with an ALPL/ALP abnormality limited to bone in the latter group.

**COP03**

**Mineral homeostasis in adults with suspected hypophosphatasia: data from the FAME study**

*Tatiane Vilaca*[1], *Meena Balasubramanian*[1], *Richard Eastell*[1]
[1]*University Of Sheffield, Division of Clinical Medicine, Sheffield, United Kingdom*

**Abstract Text**

**Background:** Hypophosphatasia (HPP) is caused by variants in the *ALPL* gene, which encodes the tissue non-specific isoform of alkaline phosphatase. The deficient enzyme activity disrupts the mineralization process and mineral metabolism. In children, hypercalcemia and hyperphosphatemia have been reported, but less is known about mineral metabolism in adults.

**Purpose:** We aim to characterise mineral metabolism in a cohort of adults in a family mapping study (FAME) of HPP who carried a pathogenic (or likely pathogenic) variant of *ALPL*.

**Methods:** We recruited probands with HPP and investigated their first- or second-degree relatives. We tested for the familiar *ALPL* variant, and measured calcium (Ca), phosphate (P), parathyroid hormone (PTH), 25OH-vitamin D (25OHD), and creatinine using auto analysers, and pyridoxal 5'-phosphate (PLP) using HPLC in fasting blood samples. We used ANOVA or Kruskal Wallis test followed by Dunnett's or Mann-Whitney analysis where appropriate.

**Results:** We recruited 70 participants: 26 probands (PROB) for *ALPL* variants, 20 positive relatives (POS) and 24 negative relatives (NEG). PROB and POS participants had lower ALP and PTH and higher P and PLP than NEG (table). There was no difference in Ca, 25OHD, creatinine or eGFR. PTH correlated negatively with P, r -0.326, p = 0.007.

Table Mineral homeostasis by groups

| | Proband | Relative Positive | Relative negative |
|---|---|---|---|
| Ca (nmol/L) | 2.34 (0.08) | 2.34 (0.08) | 2.34 (0.07) |
| P (nmol/L) | 1.22 (0.18)** | 1.36 (0.18)*** | 1.04 (0.20) |
| ALP (IU/L) | 26.0 (21-35)*** | 28.5 (24.5-40.5)*** | 81 (60.5–108.5) |
| 25OHD (nmol/L) | 60.9 (20.6) | 50.8 (19.1) | 50.9 (20.8) |
| Creatinine (umol/L) | 72.2 (11.1) | 70.3 (10.5) | 79.8 (19.5) |
| eGFR (ml/min.1.73m2) | 90 (81-90) | 90 (81-90) | 88 (67-90) |
| PTH (pmol/L) | 4.1 (3.5-5.2)* | 3.7 (2.7-4.9)* | 4.9 (4.0-6.1) |
| PLP (nmol/L) | 137 (81-345)*** | 204 (108-315)*** | 37.5 (18.5-60.0) |

Variables are shown as mean (standard deviation) or median (interquartile range)

*p<0.05 **p=<0.01 ***p=<0.001

**Conclusion:** Pathogenic variations in *ALP* are associated with higher phosphate and lower PTH levels. Lower PTH might cause an increase in P levels.

**COP04**

**Dosing studies to determine the efficacy and safety of recombinant AAV type 8 vector encoding TNAP-D10 in late-onset hypophosphatasia**

*Flavia Amadeu De Oliveira*[1], *Cintia Kazuko Tokuhara*[1], *Fatma Faiez A Mohamed*[2], *Sonoko Narisawa*[1], *Koichi Miyake*[3], *Brian L. Foster*[4], *José Luis Millán*[1]
[1]*Sanford Burnham Prebys Medical Discovery Institute, Human Genetics Program, La Jolla, United States ;* [2]*University of Michigan School of Dentistry, Biologic and Materials Sciences & Prosthodontics, Ann Arbor- MI, United States ;* [3]*Nippon Medical School, Department of Gene Therapy, Tokyo, Japan ;* [4]*The Ohio State University, College of Dentistry, Columbus, United States*

**Abstract Text**

We documented the successful resolution of the skeletal and dental defects of infantile and adult hypophosphatasia (HPP) after a single injection of AAV8 vector encoding TNAP-$D_{10}$. This was a significant advance since patients receiving daily injections of asfotase alfa often develop lipodystrophy sometimes severe enough to force discontinuation of treatment, followed by rapid deterioration of their clinical condition. Having a viral vector deliver the therapeutic mineral-targeted TNAP in a single or limited number of injections could alleviate this concern. We conducted dosing studies in a late-onset HPP model, to assess efficacy of AAV8-TNAP-$D_{10}$, to help move this therapy to the clinic. A single dose of $4x10^6$ up to $4x10^9$ (vg/b) was intramuscularly injected into 8-week-old $Alpl^{Prx1/Prx1}$ and WT and evaluated up to 70 days post-treatment. Serum alkaline phosphatase activity was higher and $PP_i$ levels were decreased in a dose-dependent manner in treated HPP mice compared to the lowest doses and the untreated mice. ALT and BUN levels were normal in treated HPP mice, indicating the treatment did not cause any toxicity to the liver or kidney. Radiographic and micro-CT analysis of long bones of female $Alpl^{Prx1/Prx1}$ mice revealed full correction of the bone phenotype at $4x10^8$ and $4x10^9$, but bones remained hypomineralized at $4x10^6$ and $4x10^7$ (vg/b) after 70 days. In male $Alpl^{Prx1/Prx1}$, we observed skeletal improvements using $4x10^9$, but the phenotype was not fully corrected. Moreover, even the highest dose did not promote unwanted calcification of soft organs. Overall, the AAV8-TNAP-$D_{10}$ at the highest examined dose improved the bone phenotype in the late-onset HPP mice, without apparent side effects in soft organs, indicating it to be a safe dose in this study. This pre-clinical work may contribute to the development of gene therapy as an effective treatment option for the improvement of the bone phenotype in adult HPP patients.

**COP05**

**Impact of INZ-701 on Bone and Mineral Metabolism Biomarkers and Clinical Outcomes in Adults with ENPP1 Deficiency- Results from 48-week Phase 1/2 Open Label Study**

*Yves Sabbagh*[1], *Robert Wermers*[2], *Rainard Fuhr*[3], *Dirk Schnabel*[4], *Alix Besancon*[5], *Munier Nour*[6], *Deborah Wenkert*[7], *Kurt Gunter*[1]
[1]*Inozyme Pharma, Research and Development, Boston- MA, United States ;* [2]*Mayo Clinic, Division of Endocrinology- Diabetes- Metabolism- and Nutrition and Department of Medicine, Rochester, United States ;* [3]*Parexel International GmbH, Early Phase Clinical Unit Berlin, Berlin, Germany ;* [4]*Charitè-Universitätsmedizin, Center for Chronic Sick Children- Pediatric Endocrinology, Berlin, Germany ;* [5]*Hôpital Universitaire Necker Enfants Malades, Endocrino-diabetologie pediatrique, Paris, France ;* [6]*University of Saskatchewan, Pediatrics, Saskatoon, Canada ;* [7]*Wenkert & Young- LLC, N/a, Thousand Oaks- CA, United States*

**Abstract Text**

**Background:** Inactivating mutations in the *ENPP1* gene (ENPP1 Deficiency) result in low levels of inorganic pyrophosphate (PPi), a critical regulator of mineralization. Subsequent cardiovascular calcification leads to ~50% infant mortality. By early adolescence patients develop FGF-23 mediated hypophosphatemic rickets, characterized by bone deformities, short stature, joint/ligament calcification and musculoskeletal pain. INZ-701 is a recombinant ENPP1-Fc investigational product.

**Purpose:** Describe the safety and exploratory efficacy of INZ-701 in adults with ENPP1 Deficiency through the end of the phase 2 study period (week 48).

**Methods:** Phase 1/2, multicenter, open-label, multiple ascending dose study including three adults in each of three dose cohorts (0.2, 0.6 and 1.8 mg/kg; total N=9), with *ENPP1* variants and PPi <1300 nM (NCT04686175). Participants were dosed subcutaneously at Day 1, then twice weekly from Day 8 to end of study. Data as of July 6 2023 are included; topline week 48 data will be presented.

**Results:** Rapid increase in mean PPi from baseline of426±407 nM was noted in all patients, reaching normal range within 6 hours of first dose and with sustained elevation through week 48 (Fig 1). There were improvements from baseline to week 48 in FGF-23 and phosphate (Fig 2) with dose-dependent changes in BSAP consistent with bone healing. There were trends towards improvement in 6MWT (Fig 3) and physician and patient GIC. Across cohorts, INZ-701 was well-tolerated with no drug-related serious or severe (> grade 2) adverse events. Low titers of non-neutralizing ADA (≤160) were observed in 7/9 patients. Long half-life of approximately 126 hours suggests potential for once-weekly dosing.

**Conclusions:** In adults with ENPP1 Deficiency, INZ-701 was well-tolerated with no related serious or severe adverse events. INZ-701 demonstrated a rapid and sustained increase in PPi levels from baseline to week 48 with improvements in bone mineral biomarkers, functional performance, and patient and physician-reported outcomes.

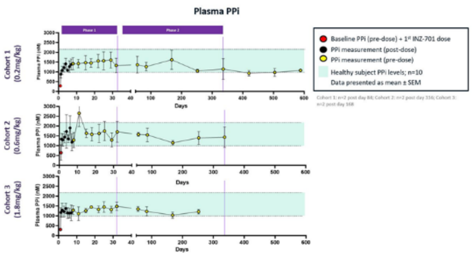

**Figure 1.** Mean plasma pyrophosphate (PPi) across dosing cohorts at each collected time point. Not all patients had reached week 48 as of July 6, 2023 data cut.

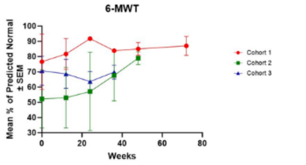

**Figure 3.** Mean % of predicted normal 6-MWT adjusted for subject age, gender, height and weight as of July 6, 2023.
*one patient in cohort 2 did not have baseline value; week 12 value substituted

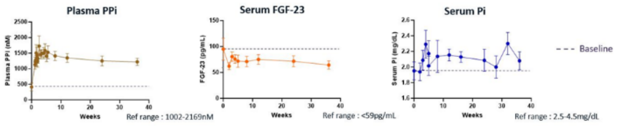

**Figure 2.** Pooled cohorts 1-3 mean baseline vs. post-treatment PPi, FGF-23, and phosphorous (Pi) levels as of July 6, 2023.

### COP06

### Noninvasive personalized therapeutic approach for the treatment of cranial bone defects in Crouzon syndrome using ferritin nanoparticles-mediated siRNA delivery

*Federica Tiberio*[1], *Martina Salvati*[2], *Luca Polito*[2], *Lorena Di Pietro*[2], *Elisabetta Falvo*[3], *Gianpiero Tamburrini*[4], *Giada Tisci*[5], *Pierpaolo Ceci*[3], *Luca Massimi*[4], *Ornella Parolini*[2], *Alessandro Arcovito*[6], *Wanda Lattanzi*[2]
[1]*Università Cattolica del Sacro Cuore, Department of Life Science and Public Health, Seleziona, Italy* ; [2]*Università Cattolica del Sacro Cuore, Department of Life Science and Public Health, Rome,*

*Italy; [3]Istitute of Molecular Biology and Pathology - National Research Council, Department of Biochemical Sciences - A. Rossi Fanelli, Rome, Italy ; [4]Fondazione Policlinico Universitario A. Gemelli IRCCS, Department of Pediatric Neurosurgery, Rome, Italy ; [5]Dipartimento di Scienze Biochimiche, Università La Sapienza, Rome, Italy ; [6]Università Cattolica del Sacro Cuore, Dipartimento di Scienze biotecnologiche di base- cliniche intensivologiche e perioperatorie, Rome, Italy*

**Abstract Text**

**Background**: Crouzon syndrome (CS) is a rare genetic craniofacial malformation caused by heterozygous “gain-of-function” mutations in the Fibroblast growth factor receptor 2 (*FGFR2*) gene. The hyperactivation of *FGFR2* receptor increases osteogenic differentiation of calviarial mesenchymal stromal cells (CMSC) within skull sutures, resulting in premature ossification and fusion (i.e. craniosynostosis) during prenatal or early postnatal stages. Surgical decompressive craniectomy is the gold standard treatment, being inherently burdened by severe complications.

**Purpose**: The aim of this study is to develop a personalized *FGFR2* knock-down strategy using allele-specific siRNAs, delivered by highly biocompatible nanoparticles (NPs) based on human recombinant ferritin (hFt).

**Methods**: CMSC were isolated from cranial tissue fragments collected as surgical waste from *FGFR2* mutation-positive CS patients (CE approved protocol #4876). siRNA sequences targeting mutant *FGFR2* have been designed and tested in CMSC. The efficiency of silencing and allele-discrimination of siRNA were analyzed through expression analysis (qPCR). The intracellular biodistribution of functionalized fluorescently-labeled hFt constructs was evaluated in CMSC cultures by Live-cells analysis microscopy. siRNA targeting *FGFR2* were conjugated to hFT surface and tested in *in vitro* culture of CMSC. siRNA delivery and release from hFt NPs were investigated through qPCR.

**Results:** For each enrolled patient, we selected specific siRNAs that reduced the expression of *FGFR2* mutant allele by at least 50% without affecting the wild-type allele expression. Expression analysis revealed that ferritin receptor *CD71* was up-regulated during osteogenic differentiation of CMSC. The intracellular trafficking analysis showed an efficient internalization of hFt, distributing within CMSC cytoplasm after as early as 10 minutes of incubation. Our data demonstrated an efficient delivery and release of siRNA from hFt within cells, reducing *FGFR2* expression levels by 40%.

**Conclusion**: Here, we show that hFt-mediated allele-specific *FGFR2* knock-down by siRNA may represent a suitable strategy to dampen the constitutive expression of FGFR2 in CS patients’ cells.

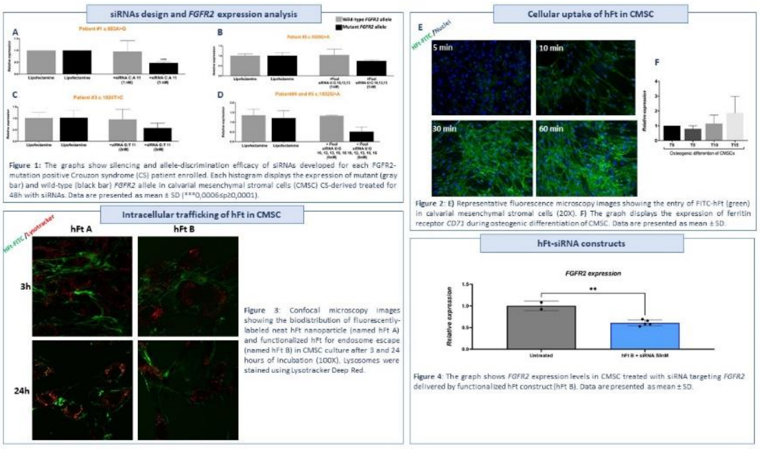

**Concurrent Oral Presentations 1: Pathophysiology and Treatment of Bone Diseases**

**COP07**

**Preferential editing of the mutated CLCN7 allele as therapeutic approach for autosomal dominant osteopetrosis**

*Dario Gajewski*[1], *Svenja Vishnolia*[1], *Anna Floriane Hennig*[1], *Regina Grün*[1], *Uwe Kornak*[1]
[1]*University Medical Center Göttingen, Institute of Human Genetics, Göttingen, Germany*

**Abstract Text**

**Introduction:** Autosomal dominant osteopetrosis type 2 (ADO2) is a hereditary bone disease caused by dysfunction of osteoclasts. ADO2 is characterized by osteosclerosis, increased fracture risk, and, in severe cases, blindness, deafness, and bone marrow insufficiency. ADO2 is exclusively caused by heterozygous mutations in *CLCN7*, which encodes the dimeric $Cl^-/H^+$ exchanger ClC-7. ClC-7 is crucial for extracellular acidification by osteoclasts important for degradation of the mineralized bone matrix. Mutations can cause dysfunction, instability or mislocalization of ClC-7 dimers in a dominant negative manner. The p.Gly215Arg mutation is one of the most frequent pathogenic variants in Europe and causes retention in the endoplasmatic reticulum.

With an incidence of 1 in 20000 births, ADO2 is among the most abundant skeletal dysplasias, still there is no curative treatment yet.

**Purpose:** Using CRISPR/Cas9, we developed a strategy to selectively inactivate the p.Gly215Arg mutated allele in peripheral blood monocytes to ameliorate dominant negative effects and increase bone resorption.

**Methods:** Monocytes were isolated from blood of an individual with ADO2. The cells were transfected by nucleofection with Cas9/sgRNA complexes targeting the p.Gly215Arg mutation. Efficiency was assessed by Deep Sequencing. Functional effects were evaluated by differentiation of monocytes into osteoclasts on bone chips *in vitro*.

**Results:** In four independent experiments we achieved knockout of 95 % mutated alleles while retaining 75 % unedited wildtype alleles (Fig.A). On cDNA level, this resulted in 72 % wildtype reads in osteoclasts (Fig.B). The mutation-specific knockout caused a 2.4 fold increase ($p=0.0002$, Mann-Whitney Test) of bone resorption in *CLCN7*-edited cells compared to cells edited in the *B2M* locus as control (Fig.C).

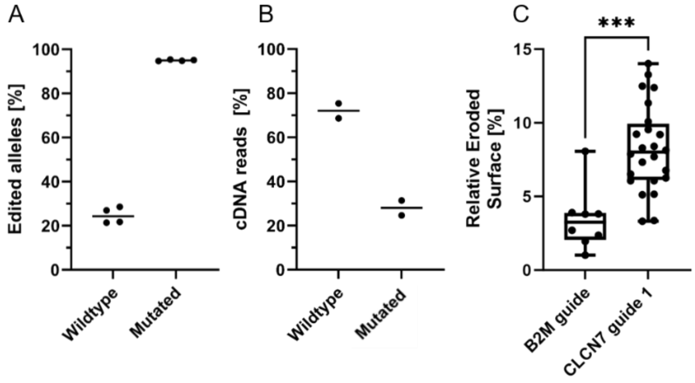

**Conclusions:** Specific inactivation of the mutated *CLCN7* allele is a promising strategy for an ADO2 gene therapy that may complement currently tested RNAi-based approaches.

**COP08**

**CFTR modulators treatments (ETI) partially restore osteoclasts phenotype in cystic fibrosis-related bone disease**

*Johan Sergheraert[1,2,3], Christine Guillaume[1], Claire Dumortier[1], Julien Braux[1,2,3], Muriel Griffon[4], Bruno Ravoninjatovo[4], Pierre-Régis Burgel[5], Loïc Pierrard[2,3,6], Sophie C Gangloff[1,7], Marie-Laure Jourdain[1,2,3], Frédéric Velard[1]*
*[1]Université de Reims Champagne-Ardenne, Biomatériaux et Inflammation en Site Osseux- BIOS EA 4691, Reims, France ; [2]Centre Hospitalo-Universitaire de Reims, Pôle de Médecine Bucco-Dentaire, Reims, France ; [3]Université de Reims Champagne-Ardenne, UFR d'Odontologie, Reims, France ; [4]Centre Hospitalo-Universitaire de Reims, Service de pneumologie- CRCM Reims, Reims, France ; [5]AP-HP- Hôpital Cochin, Service de Pneumologie- CRCM, Paris, France ; [6]Université de Reims Champagne-Ardenne, Vieillissement- Fragilité- VieFra- EA 3795, Reims, France ; [7]Université de Reims Champagne-Ardenne, UFR de Pharmacie, Reims, France*

**Abstract Text**

Cystic fibrosis-related bone disease (CFBD) is a frequent comorbidity in CF patients. CFBD is characterized by low bone density and increased risk of fracture, even during childhood. However, mechanisms explaining the impact of defective CFTR function on bone homeostasis remain not fully elucidated. Previous data demonstrated that loss of CFTR function led to impaired osteoblastogenesis as well as a rise in the number of circulating pre-osteoclastic $RANK^+MCSFR^+$ monocytes. We aimed to study the impact of CFTR channel loss of activity and its correction on osteoclast (OC) phenotype. Circulating pre-OC monocytes were isolated from 33 patients bearing CFTR Class II mutations (NCT04877223) and 23 healthy controls (ALC/PIL/DIR/AJR/FO/606). Membrane RANK and MCSFR receptors were labelled for flow cytometry study. After 14 days, OC were plated on dentin slices for 7 days to evaluate resorption activity. After 21 days of differentiation, OC were stained (Phalloïdin-AlexaFluor488®, Vinculin-immunofluorescent staining, DAPI) to determine osteoclasts number, size and evaluate focal adhesion complexes. Pre-OC monocytes were treated with prolonged exposure to Elexacaftor, Tezacaftor and Ivacaftor (ETI:3µM/3µM/1µM). We evidenced an increased proportion of $RANK^+MCSFR^+$ cells in CF patients' blood (2.3fold) compared to controls. OC exhibiting CFTR mutations were less numerous but larger than non-CF ones (1.5fold). ETI treatment did not restore number of OC but significantly decreased (-70%) their size towards non-CF ones. Resorption activity that was strongly reduced in CF cultures (40fold), was enhanced in treated culture compared to untreated (12fold) without reaching non-CF levels. Vinculin staining was thinner and less co-localized with actin ring in OC bearing-CFTR Class II mutation than in control. ETI treatment partially restored cytoskeleton organization. Our results tend to demonstrate defective OC differentiation and resorption process in OC bearing class II CFTR mutations, which are partially restored by CFTR modulator treatment. This could explain disruption in bone homeostasis observed in CF patients.

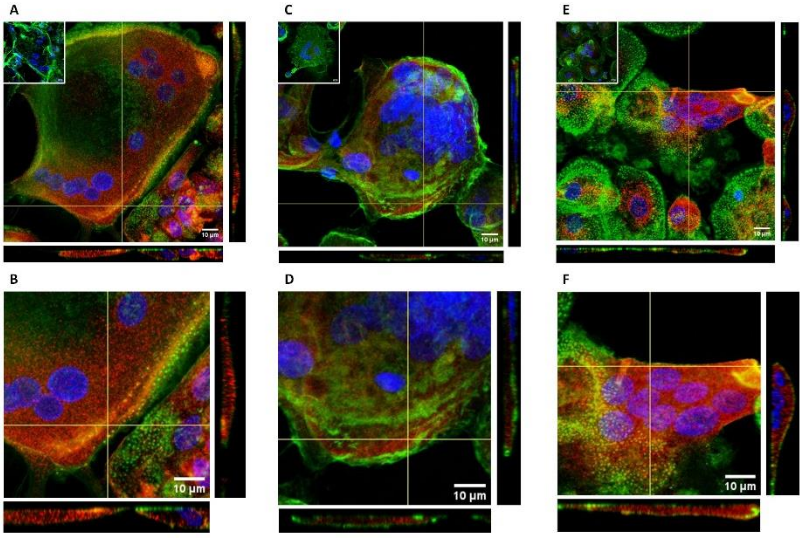

*Figure : CF osteoclasts present an altered podosomes belt partially restored after treatment. Representative photographs of non-CF (A,B, CF (C,D) and treated-CF (E,F) osteoclast culture. Colorized images : Phalloidin-AlexaFluor®488 / Vinculin (AlexaFluor®568) / nucleus (DAPI).*
*Scale bar = 10µm. Non-CF culture : n=23 ; CF culture : n=33 ; treated-CF culture : n=16*

**COP09**

**Anti-myostatin treatment effectively ameliorates bone metastasis in a xenograft mouse model of breast cancer**

*Julia Reinhardt[1], Fabienne Geers[1], Sarah Boedecker[1], Christiane Geyer[2], Thomas Pap[1], Berno Dankbar[1], Corinna Wehmeyer[1]*
*[1]University of Muenster, Institute of Musculoskeletal Medicine, Muenster, Germany ; [2]University of Muenster, Translational Radiology, Muenster, Germany*

**Abstract Text**

**Background:** Breast cancer (BC)-derived bone metastases colonize to bone and induce severe bone degradation by a complex interaction with osteoclasts (OC). In turn, resorbed bone releases many soluble factors such as TGF-β and calcium from matrix stores that stimulates tumor growth that further triggers bone resorption. Myostatin, a member of the TGF-β superfamily, highly promotes bone destruction by enhancing osteoclast development in mouse models of musculoskeletal diseases. However, the impact of myostatin on bone lesions and metastases in BC is completely unknown.

**Aim:** Analysis the impact of myostatin on tumor-cell OC interaction promoting bone metastases mediated bone loss in a xenograft BC mouse model.

**Methods**: Murine bone marrow macrophages (BMM) were differentiated into OC in co-culture experiments with MDA-MB-231 conditioned media (CM) or cells and co-incubated with anti-Myostatin antibodies. OC formation was determined using TRAP-staining and OC resorption by evaluating resorption pits on CaP-coated plates. A xenograft MDA-MB-231 BC mouse model was treated with neutralizing myostatin antibodies (RK-35 by Pfizer). Distribution of MDA-MB-231-luc+ cells were analyzed by bioluminescence imaging and bone lesions by µCT. OC formation, metastatic spread and myostatin expression were evaluated by TRAP-, H&E- and immunohistology on tibiae sections.

**Results:** OC precursors co-cultured with MDA-MB-231 cells or CM showed a significantly accelerated OC formation (cells: 1.6-fold, *p*=0.0348; CM: 2.3-fold, *p*=0.0197*)* and increased resorption area (cells: 3.7-fold, *p*=0,0132; CM: 4-fold, *p*=0.0011) *in vitro* that was completely prevented by anti-Myostatin antibodies. *In vivo*, anti-Myostatin treated MDA-MB-231-luc+-bearing tumor mice revealed reduced bioluminescence intensity, elevated trabecular bone density parameters (BV/TV: 4-fold, *p*=0,0014; Tb.Th: 2.1-fold, *p*=0,0001; Tb.N: 3.9-fold ,*p*=0.0003) and reduced TRAP$^{+}$-OC (-57%, *p*=0.0342) compared to vehicle controls.

**Conclusion:** Our results strongly indicate that neutralizing Myostatin decrease the development of metastases, osteoclasts and bone damage. Therefore, blocking Myostatin may be a promising treatment option for osteolytic bone metastasis.

**COP10**

**Mitochondrial E3 Ubiquitin Ligase MUL1 reduces proliferation and increases cell death in metastatic osteosarcoma cells**

*<u>Jacopo Di Gregorio</u>[1], Sara Terreri[2], Michela Rossi[2], Giulia Battafarano[2], Laura Di Giuseppe[3], Olivia Pagliarosi[2], Antonis S Zervos[4], Vincenzo Flati[1], Andrea Del Fattore[2]*
*[1]University of L'Aquila, Department of Biotechnological and Applied Clinical Sciences, L'Aquila, Italy ; [2]Bambino Gesù Children's Hospital- IRCCS, Bone Physiopathology Research Unit- Translational Pediatrics and Clinical Genetics Research Division, Rome, Italy ; [3]Sapienza University, Department of Clinical- Internal- Anaesthesiological and Cardiovascular Sciences, Rome, Italy ; [4]University of Central Florida, Burnett School of Biomedical Sciences- College of Medicine- University of Central Florida, Orlando, United States*

**Abstract Text**

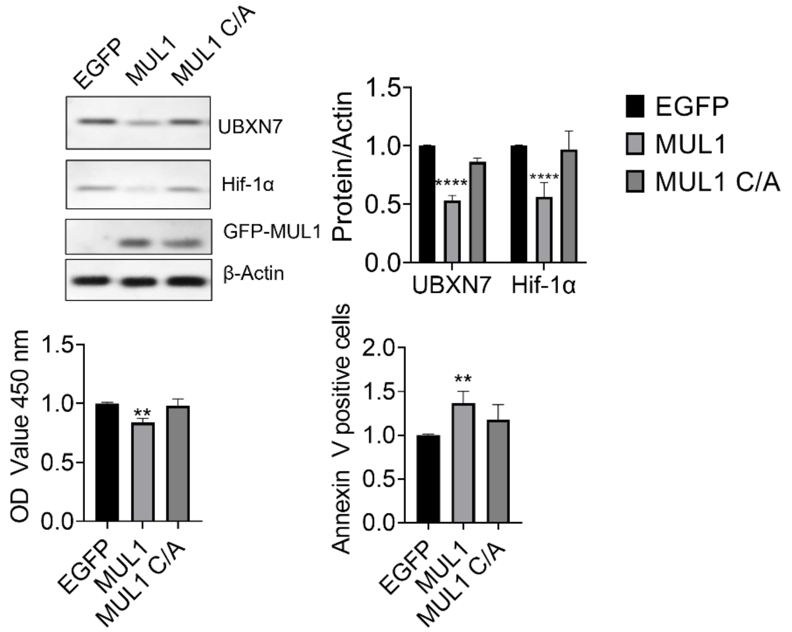

Osteosarcoma is a highly aggressive and metastatic bone cancer. Identification of new therapeutic targets is important for effective treatments. A possible strategy leverages the inhibition of the hypoxia-inducible factor HIF-1α, upregulated in metastatic osteosarcoma.
This study aims to evaluate the effects of HIF-1α downregulation by the mitochondrial E3 ubiquitin ligase MUL1 and the cofactor protein UBXN7 pathway in osteosarcoma cells.

Firstly, we observed that MUL1 protein levels inversely correlate with tumor aggressiveness; it was expressed in the osteoblast-like osteosarcoma Saos-2 cells, while barely detected in the highly aggressive and metastatic 143B cells [Arbitrary Unit (AU), Saos-2: 0.86±0.15, 143B: 0.24±0.07, p<0.01); accordingly, the expression of UBXN7 (AU, Saos-2: 0.14±0.04, 143B: 1.06±0.05, p<0.01) and HIF-1α (AU, Saos-2:0.12±0.03, 143B:0.90±0.17, p<0.01) was higher in 143B cells.

MUL1 overexpression in 143B cells causes a 50% reduction of UBXN7 (AU, pEmpty-143B: 1.01±0.08 pMUL1-143B: 0.50±0.03, p<0.0001) and HIF-1α protein levels (pEmpty-143B: 0.79±0.10, pMUL1-143B: 0.43±0.07, p<0.008). Most importantly, MUL1 induction in 143B cells reduces the proliferative rate of 143B cells (AU, pEmtpy-143B: 1.01±0.04 , pMUL1-143B: 0.79±0.02, p<0.001) coupled with induction of apoptosis, as shown by the increase of Annexin V positive cells (AU, pEmtpy-143B: 1.01±0.02, pMUL1-143B: 1.36±0.01, p<0.01) and of the cleaved isoforms of PARP (AU, pEmpty-143B: 1.01±0.05 pMUL1-143B: 2.65±0.06, p<0.02) and Caspase 3 (AU, pEmpty-143B: 1.01±0.05 pMUL1-143B: 3.36±0.09, p<0.04).

Induction of MUL1 treatment with Ucf-101, an inhibitor of the upstream mediator of MUL1 HTRA2, achieves similar results. Moreover, overexpression of an inactive MUL1 mutant fails to induce HIF-1α downregulation and cell death.

Our results show that the MUL1/UBXN7 pathway is dysregulated in metastatic osteosarcoma cells, and that restoring MUL1 levels in that scenario results not only in HIF-1α downregulation, but also in reduced cell proliferation and increased cell death.

**COP11**

**Glucocorticoid-induced osteoporosis and the osteohematopoietic niche**

*Heidi Lunze[1], Holger Henneicke[2], Lorenz C. Hofbauer[1], Martina Rauner[1], Ulrike Baschant[1]*
*[1]University Hospital Carl Gustav Carus- Dresden University of Technology, Department of Medicine III & Center for Healthy Aging- Medical Faculty, Dresden, Germany ; [2]University Hospital Carl Gustav Carus- Dresden University of Technology, Department of Medicine III & Center for Healthy Aging- Medical Faculty and Center for Regenerative Therapies, Dresden, Germany*

**Abstract Text**

**Background:** Glucocorticoids (GCs) are potent immunosuppressive drugs exerting anti-inflammatory effects through complex actions on circulating immune cells as well as tissue-specific cells. However, long-term GC treatment causes severe side effects such as glucocorticoid-induced osteoporosis due to adverse actions of GCs in bone. Despite the known effects of GCs on immune and bone cells, GC actions in the osteohematopoietic niche remain ill-defined. Thus, we aimed to explore the effects of long-term GC treatment on hematopoietic stem cell (HSC) function.

**Methods:** 16-week-old conditional GR deficient mice ($GR^{GtRosaERTCre}$, $GR^{OsxCre}$ and $GR^{VavCre}$) lacking GR expression ubiquitously, in osteoprogenitor cells or HSCs were treated with 75 mg/L corticosterone (4 weeks via the drinking water). HSC numbers and function were examined by flow cytometry analysis and competitive bone marrow transplantation. Bone turnover was measured by µCT and bone histomorphometry analysis.

**Results:** GC treatment reduced bone formation (BFR/BS, -65%, p<0.05) in a GR-dependent manner and diminished the number of short-term (LSK $CD34^{+}CD150^{-}CD135^{+}$) (-50%, p=<0.05) HSCs as well as primitive, long-term (LSK $CD34^{-}CD150^{+}CD135^{-}$) HSCs (60%, p<0.05) in $GR^{flox}$ control and $GR^{OsxCre}$ but not in $GR^{GtRosaCre}$ and $GR^{VavCre}$ mice. Whereas cell cycle analysis of HSC subtypes revealed no differences, we observed an increased apoptosis rate in LSK cells (+30%, p<0.01) upon GC treatment.

Interestingly, the multi-lineage reconstitution ability of hematopoiesis of HSCs was significantly increased (+50%, *p*<0.05) after GC exposure. Especially, cells of the lymphoid lineage showed a higher ability for multi-lineage differentiation potential (CD3: +80%, p<0.05; CD19: +55%, p<0.05) 12 weeks after bone marrow transplantation. The increase of repopulation ability was abrogated in mice with GR-deficiency in HSCs and partially in mice lacking the GR in osteoprogenitor cells.

**Conclusion:** Taken together, our data suggest that long-term GC exposure reduces HSC numbers in a HSC-GR-dependent manner, but improves HSC function in a HSC- and osteoblast-specific GR-dependent manner.

**COP12**

**Lrp5 p.Val667Met variant does not alter the response to anti-sclerostin antibodies**

*Stéphanie Fabre[1,2], Morgane Bourmaud[1], Agnès Ostertag[1], Thomas Funck-Brentano[1,2], Corinne Collet[1,3], Martine Cohen-Solal[1,2]*
*[1]INSERM, U1132 Bioscar- Université Paris Cité, Paris, France ; [2]Lariboisière hospital AP-HP, Rheumatology department, Paris, France ; [3]Robert Debré hospital AP-HP, Molecular Genetic department, Paris, France*

**Abstract Text**

**Background:** Early-onset osteoporosis has been associated to LRP5, coding for a co-receptor in the Wnt pathway. Genome wide-association studies showed that LRP5 p.Val667Met (V667M) variant is associated with low bone mineral density (BMD) and increased fractures. However, the efficacy of anti-sclerostin treatment, also acting on the Wnt pathway, remains unknown in presence of the V667M variant.

**Purpose:** Here, we aimed to evaluate the response to anti-sclerostin antibodies (Scl-Ab), with or without corticosteroid treatment, in the *Lrp5*$^{V667M}$ mouse model, carrying the equivalent of the human V667M variant and having low BMD compared to control (CTL) mice.

**Methods:** Scl-Ab or vehicle (Veh) was administered during 4 weeks to *Lrp5*$^{V667M}$ and CTL 3-month-old mice, or 4.5-month-old mice in conjunction to corticosteroid pellets or placebo for 4 weeks.

**Results:** Scl-Ab was efficient in increasing BMD compared to Veh, as expected in CTL mice (BMD increase during treatment (ΔBMD) femur 29.71±10.29 vs 13.31±8.61mg/cm², p=0.0098 and lumbar spine 17.22±6.24 vs 3.08±5.83mg/cm², p=0.0141) but also in *Lrp5*$^{V667M}$ mice (ΔBMD femur 37.72±11.03 vs 11.65±11.68g/cm², p=0.0004 and lumbar spine 17.47±9.17 vs 4.29±11.31g/cm², p=0.0101). Scl-Ab also improved cortical and trabecular microarchitecture compared to Veh in *Lrp5*$^{V667M}$ mice (Ct.Th 217.1±20.7 vs 174.9±11.0µm, p<0.001; BV/TV 21.04±5.43 vs 12.76±2.78%, p=0.0017 ; Tb.Th 74.12±7.00 vs 52.7±4.65µm, p<0.001). Scl-Ab was also efficient to prevent corticosteroid-induced osteoporosis, as BMD in *Lrp5*$^{V667M}$ mice treated with corticosteroid and Scl-Ab compared to corticosteroid and Veh was 111.9±8.0 vs 94.91±7.1mg/cm² (p=0.0014) for femur and 83.7±6.1 vs 68.7±3.8mg/cm² for lumbar spine (p=0.0012). Trabecular microarchitecture, but not cortical, was also improved when *Lrp5*$^{V667M}$ mice receiving corticosteroid were treated with Scl-Ab compared to Veh (BV/TV 12.46±1.08 vs 7.30±3.20% , p=0.0058).

**Conclusion:** Scl-Ab is efficient in increasing BMD and improving bone microarchitecture in the presence of the V667M variant. Therefore, such anabolic agent will be valuable for patients with early onset osteoporosis.

### Concurrent Oral Presentations 2: Osteoporosis

### COP13

### Analysis of the relationship between baseline BMD and fracture incidence in the placebo groups using individual patient data from the FNIH-ASBMR-SABRE Project

*Marian Schini*[1], *Tatiane Vilaca*[1], *Eric Vittinghoff*[2], *Li-Yung Lui*[3], *Susan Ewing*[2], *Austin Thompson*[2], *Douglas Bauer*[2], *Mary Bouxsein*[4], *Dennis Black*[2], *Richard Eastell*[5]
*[1]University of Sheffield, Division of Clinical Medicine- School of Medicine and Population Health, Sheffield, United Kingdom ; [2]University of California, Department of Epidemiology & Biostatistics, San Francisco, United States ; [3]California Pacific Medical Center, Research institute, San Francisco, United States ; [4]Harvard Medical School, Center for Advanced Orthopedic Studies- Beth Israel Deaconess Medical Center and Department of Orthopedic Surgery-, Boston, United States ; [5]The University of Sheffield, Division of Clinical Medicine- School of Medicine and Population Health, Sheffield, United Kingdom*

**Abstract Text**

**Introduction:** We have proposed that treatment-related increases in total hip (TH) BMD at two years could be a surrogate for fracture risk reduction in clinical trials. The validation of a surrogate includes the association of the surrogate with the clinical outcome. We have compiled a large database of individual patient data (IPD) through the FNIH-ASBMR-SABRE project, and this study aimed to assess the relationship between BMD and fracture risk in the placebo groups.

**Methods:** We estimated the association between a) baseline THBMD and b) baseline femoral neck (FNBMD) and fracture risk using IPD from the combined placebo groups. We included data from 25 randomised controlled trials (RCTs). We standardized the BMD data for our analyses. We estimated the relative risk of fracture per standard deviation (SD) decrease in baseline BMD using logistic regression models for radiographic vertebral fractures and proportional hazards models for hip, non-vertebral, "all" and "all clinical" fractures.

**Results:** The number of placebo participants included in THBMD analyses was 33,916 for vertebral fractures and 42,847 for the other fracture types. The respective numbers for the FNBMD analyses were 36,176 and 46,125. We observed significant relationships between baseline BMD and fracture risk within the combined placebo groups for all five fracture types (**Table**). These relationships were similar for age, race and study. Plotting fracture incidence as a function of the THBMD quintile confirms the strong association between BMD and fracture risk.

**Conclusion:** These data support the very strong relationship between BMD and fracture risk and provide supporting rationale for change in BMD as a surrogate for fracture risk reduction in future RCTS. A unique aspect of our study is that we used selected patients with high fracture risk, and the results might differ from the population-based studies.

***Table*** *Association between baseline hip BMD and incident fracture in the placebo group from combined studies: odds ratio or hazard ratio (95% CI) per 1 standard deviation (SD) decrement*

| Fracture outcome | TH sBMD *(per 1 SD (110 mg/cm²) decrease)* N = 42,847* Unadjusted | Adjusted** | FN sBMD *(per 1 SD (89 mg/cm²) decrease)* N = 46,125* Unadjusted | Adjusted** |
|---|---|---|---|---|
| Vertebral | 1.55 (1.48, 1.62) | 1.54 (1.46, 1.62) | 1.46 (1.40, 1.53) | 1.47 (1.39, 1.54) |
| Hip | 2.27 (2.09, 2.47) | 1.94 (1.78, 2.12) | 2.20 (2.01, 2.40) | 1.94 (1.77, 2.13) |
| Non-Vertebral | 1.31 (1.27, 1.36) | 1.32 (1.28, 1.37) | 1.29 (1.25, 1.33) | 1.32 (1.28, 1.37) |
| All | 1.43 (1.39, 1.47) | 1.39 (1.35, 1.44) | 1.40 (1.36, 1.44) | 1.37 (1.33, 1.41) |
| All Clinical | 1.35 (1.31, 1.39) | 1.34 (1.30, 1.39) | 1.31 (1.27, 1.35) | 1.33 (1.29, 1.38) |

sBMD = standardized bone mineral density; TH = Total hip; FN = Femoral neck
*N=33,916 for TH and N=36,176 for FN for the subset of participants in the vertebral fracture analysis
**adjusted for age, race (white vs. others), and study
SDs determined from all placebo participants

**COP14**

**A Randomized, Double-blind, Phase III Study to Compare SB16 (Proposed Denosumab Biosimilar) to Reference Denosumab in Patients with Postmenopausal Osteoporosis: 18-Month Results**

*Richard Eastell[1], Bente Langdahl[2], Yoon-Sok Chung[3,4], Rafal Plebanski[5], Edward Czerwinski[6], Eva Dokoupilova[7], Jerzy Supronik[8], Jan Rosa[9], Andrzej Mydlak[10], Anna Rowinska-Osuch[11], Ki-Hyun Baek[12,13], Audrone Urboniene[14], Robert Mordaka[15], Sohui Ahn[16], Young Hee Rho[17], Jisuk Ban[17]*
*[1]University of Sheffield, Division of Clinical Medicine, Sheffield, United Kingdom ; [2]Aarhus University Hospital and Aarhus University, Department of Endocrinology, Aarhus, Denmark ; [3]Ajou University School of Medicine, Department of Endocrinology and Metabolism, Suwon, Korea- South ; [4]Ajou University Medical Center, Institute on Aging, Suwon, Korea- South ; [5]Klinika Zdrowej Kosci, Clinic of Healthy Bone, Lodz, Poland ; [6]Krakowskie Centrum Medyczne sp z oo, Clinical Trial Center, Krakow, Poland ; [7]MEDICAL PLUS sro, Rheumatology, Uherske Hradiste, Czech Republic ; [8]OsteoMedic sc A Racewicz J Supronik, Rheumatology, Bialystok, Poland ; [9]Affidea Praha- s.r.o., Osteocentre, Praha, Czech Republic ; [10]ETG Siedlce, Medical, Siedlce, Poland ; [11]ETG Warszawa, Clinical Trials, Warszawa, Poland ; [12]Yeouido St Mary's Hospital, Division of Endocrinology, Seoul, Korea- South ; [13]Yeouido St Mary's Hospital, Department of Internal Medicine, Seoul, Korea- South ; [14]JSC Saules seimos medicinos centras, Family Medicine, Kaunas, Lithuania ; [15]Santa Sp. z o.o., Santa Familia PTG Lodz, Lodz, Poland ; [16]Samsung Bioepis, Biometrics, Incheon, Korea- South ; [17]Samsung Bioepis, Clinical Development, Incheon, Korea- South*

**Abstract Text**

**Background:** SB16 has been developed as a proposed biosimilar to reference denosumab (Prolia, DEN hereafter).

**Purpose:** We have previously reported the biosimilarity of SB16 to DEN in terms of efficacy, pharmacokinetics (PK), pharmacodynamics (PD), safety, and immunogenicity up to 12 months.[1] Here we present the switching period results up to Month 18.

**Methods:** Postmenopausal osteoporosis patients were randomized in a 1:1 ratio to receive either 60 mg of SB16 or DEN subcutaneously at Month 0 and Month 6. At Month 12, patients were rerandomized into switching period to continue their treatment (SB16+SB16 or DEN+DEN) or switch from DEN to SB16 (DEN+SB16). Efficacy, PK, PD, safety, and immunogenicity were assessed up to Month 18. The study received ethical approval, and all participating patients gave informed consent.

**Results:** Among 457 patients, 407 patients were re-randomized at Month 12 and entered the switching period; SB16+SB16 (n=206), DEN+DEN (n=101), DEN+SB16 (n=100). A total of 404 patients completed the switching period.

The mean %change from baseline in lumbar spine bone mineral density (BMD) at Month 18 was comparable between treatment groups. The least squares mean (LSM) of %change from baseline in lumbar spine BMD at Month 18 was 6.77 (standard error: 0.286) for SB16+SB16. The LSM difference in the lumbar spine BMD change for the full analysis set was –0.03% (90% CI [–0.85, 0.79]) between SB16+SB16 and DEN+DEN, and –0.52 (90% CI [–1.48, 0.43]) between DEN+SB16 and DEN+DEN.

The mean %change from baseline in total hip BMD and femoral neck BMD at Month 18 were comparable between treatment groups. The PK, PD, safety, and immunogenicity profiles were also comparable up to Month 18.

**Conclusion:** The efficacy, PK, PD, safety, and immunogenicity among the treatment groups (SB16+SB16, DEN+DEN, and DEN+SB16) were comparable up to Month 18.

**Reference:** [1]Eastell R et al., ASBMR 2023, SUN-432

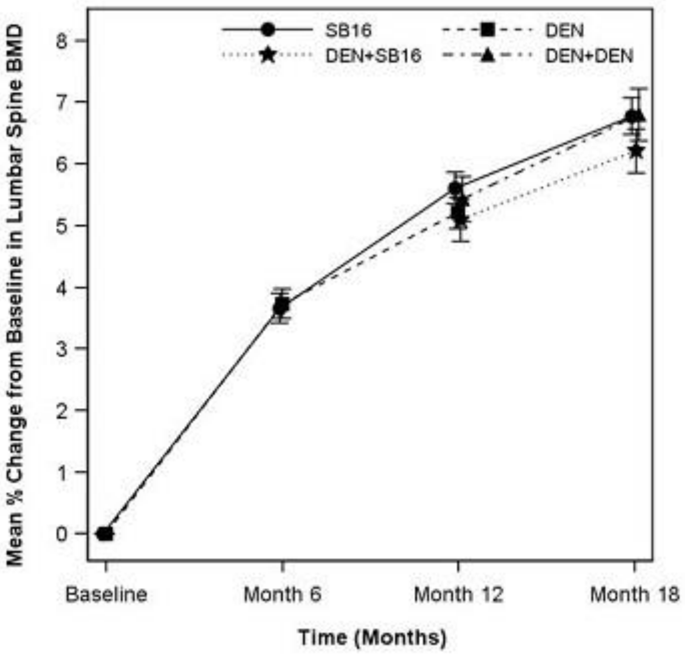

**Figure 1. Mean Percent (%) Change from Baseline in Lumbar Spine BMD up to Month 18 (Full Analysis Set)**

**COP15**

**Clinical Experience with Denosumab Discontinuation**

Natasha Laursen[1], Anne Sophie Sølling[1], Torben Harsløf[1], Bente Langdahl[1]
[1]Department of Endocrinology and Internal Medicine, Aarhus University Hospital, Aarhus, Denmark

**Abstract Text**

**Background:** Discontinuing denosumab (DMAb) rapidly decreases bone mineral density (BMD) and increases the risk of multiple vertebral fractures. We examined if treatment with zoledronate (ZOL) administrated when bone turnover was increased could prevent the bone loss.

**Methods:** We conducted a retrospective cohort study based on medical records of 63 patients referred for DMAb discontinuation. We retrieved information on biochemistry and DXA performed prior to DMAb initiation, at the last injection, and 12 and 24 months after discontinuation. We administered ZOL 6 months after the last DMAb injection and 3, 6, 12 and 24 months thereafter if p-C-terminal collagen crosslinks (CTX) increased above 0.5 µg/l or BMD decreased (≥5% at the hip, ≥3% at the spine) at month 12 and 24.

**Results:** We included 63 men and women discontinuing DMAb after a mean treatment duration of 6.7±2.7 years resulting in mean increases in BMD of 5.9±4.9% at TH and 14.0±6.0% at LS. A total of 9, 32, 29 and 12 patients fulfilled the p-CTX and/or BMD criteria for re-treatment with ZOL at month 3, 6, 12 and 24, respectively, with a mean number of injections of 3 (range 1–5).
BMD decreased significantly 12 months after the initial ZOL treatment by -1.9±2.5% and -2.5±4.2% at the TH and LS, respectively (n=44) (p≤0.005 for all) but was maintained from month 12 to 24 (n=12) (p>0.05 for all).
Furthermore, an increase in BMD above the mean during DMAb treatment was associated with a greater bone loss at M12 (TH: -2.9±2.5% vs. -0.6±2.1%, LS: -3.9±3.6% vs. -0.4±4.2%, p<0.02 for both).

**Conclusion:** In this retrospective cohort study of 63 patients discontinuing long-term DMAb, a mean of 3 infusions of ZOL limited the bone loss 12 and 24 months after DMAb discontinuation and thereby preserving most of the BMD gain during DMAb treatment.

### COP16

### Romosozumab and denosumab combination therapy in postmenopausal osteoporosis

Giovanni Adami[1], Elisa Pedrollo[1], Maurizio Rossini[1], Angelo Fassio[1], Vania Braga[1], Camilla Benini[1], Ombretta Viapiana[1], Gatti Davide[1]
[1]University of Verona, Rheumatology Unit, Verona, Italy

**Abstract Text**

**Background:** Optimization of sequential and combination treatment is crucial in shaping long-term management of postmenopausal osteoporosis (OP).

**Purpose:**
To investigate the effectiveness of romosozumab added to ongoing denosumab treatment in postmenopausal OP.

**Methods:** We conducted a 6-month prospective observational study on postmenopausal women with severe OP receiving treatment with romosozumab either alone or in combination with ongoing long-term denosumab (>2 years) or continuing denosumab alone (>2 years). We collected serum samples for bone turnover markers (P1nP and CTX) and bone modulators (sclerostin and Dkk1) at month 3, and month 6. Bone mineral density was assessed at baseline and after 6 months.

**Results:** Fifty-two postmenopausal women with OP were included in the study. Nineteen received romosozumab alone, 11 received romosozumab combined with ongoing denosumab, and 22 continued denosumab alone. BMD increased significantly at all sites at 6 months of follow-up in the romosozumab naive group (femoral neck +8.1% SD ±10.3, total hip +6.8% SD ±6.5 and lumbar spine +7.9% SD ±11.6; p 0.034, p 0.009 and p 0.037, respectively - **figure 1A**). In contrast, in the combination group and the denosumab group, BMD increased significantly only at the lumbar spine (+7.2% SD ±4.5, p 0.027 and +1.5% SD ±2.7, p 0.022; respectively). Regarding BTMs, P1nP increased significantly with romosozumab at month 3 (+70.4% in romosozumab alone group and +99.1% in the combination group, **figure 1B**). Sclerostin levels increased steeply in both romosozumab groups at month 3 and month 6 (greater than +2000% in both groups) and Dkk1 did not change.

**Figure 1.**

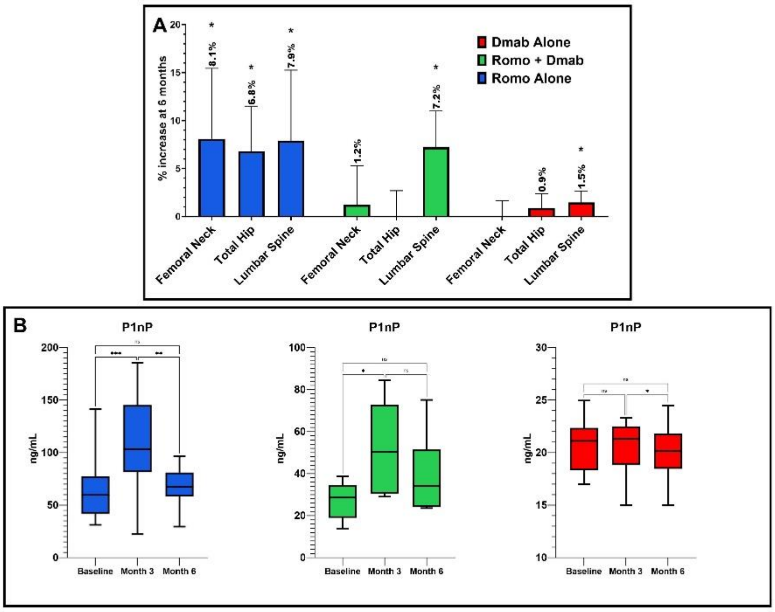

**Conclusion:** Romosozumab added to ongoing denosumab resulted in an increase in P1nP and lumbar spine BMD, but not in femoral neck BMD, the increase was greater than denosumab alone. For the uncommon cases of patients who suffer a vertebral fracture while being treated with denosumab, using romosozumab as an additional treatment might be effective.

### COP17

### Effectiveness of conservative treatment in the management of acute painful vertebral compression fractures: Systematic review and network meta-analysis

*Tim Rolvien[1], Athanasios D. Anastasilakis[2], John J. Carey[3], Stella D'Oronzo[4], Anda M. Naciu[5], Julien Paccou[6], Maria P. Yavropoulou[7], Willem F. Lems[8], Assil-Ramin Alimy[1], Ects Clinical Practice Action Group[9]*
*[1]University Medical Center Hamburg-Eppendorf, Department of Trauma and Orthopaedic Surgery, Hamburg, Germany ; [2]424 General Military Hospital, Department of Endocrinology, Thessaloniki, Greece ; [3]Galway University Hospitals, Department of Rheumatology, Galway, Ireland ; [4]University of Bari Aldo Moro, Interdisciplinary Department of Medicine, Bari, Italy ; [5]Fondazione Policlinico Universitario Campus Bio-Medico, Unit of Metabolic Bone and Thyroid Diseases, Rome, Italy ; [6]Univ.*

*Lille-CHU Lille, MABLab ULR 4490-Department of Rheumatology, Lille, France ; [7]Medical School-National and Kapodistrian University of Athens, 1st Department of Propaedeutic and Internal Medicine Center of Expertise for Rare Endocrine Diseases C.E.R.E.D, Athens, Greece ; [8]Amsterdam University Medical Center, Department of Rheumatology, Amsterdam, Netherlands ; [9]Clinical Practice Action Group, Ects, n/a, Germany*

**Abstract Text**

**Background:** Acute vertebral compression fractures (VFs) substantially impair quality of life due to pain and reduced mobility. Effective pain management is crucial for improving functionality and patient outcomes. While there is a lack of consistent evidence for pain relief with surgical procedures such as vertebroplasty and kyphoplasty, the potential of optimized conservative pain management is receiving increasing attention. However, the effectiveness of conservative treatments remains unclear.

**Purpose:** To evaluate the effectiveness of various conservative treatments in managing pain and improving quality of life in patients with acute painful VFs.

**Methods:** A systematic review and network meta-analysis was conducted according to the Cochrane Handbook and PRISMA guidelines. Eligible studies were randomized controlled trials (RCTs) and prospective comparative studies comparing conservative treatments for acute painful osteoporotic VFs to each other and against no treatment or placebo.

**Results:** Twenty studies (16 RCTs, four prospective comparative studies) from a total of 15,770 retrieved reports met our criteria, which included 1,788 patients. Treatments ranged from braces to bone-modifying agents including bisphosphonates, calcitonin and teriparatide. Braces showed no benefit in pain reduction compared to no braces. Teriparatide was more effective in pain management (standardized mean difference (SMD) -1.22, [95%-CI -2.32 to -0.12]) and functional outcomes (SMD -4.88, [95%-CI -6.06 to -3.69]) compared to bisphosphonates (*Figure 1A, B*). Calcitonin proved more effective than bisphosphonates (SMD -3.28, [95%-CI -4.14 to -2.43]) and teriparatide (SMD -3.19, [95%-CI -4.13 to -2.25]) regarding pain reduction.

**Conclusion:** Teriparatide appears superior to bisphosphonates in managing pain and improving functional outcomes. Calcitonin was also effective; however, approval restrictions limit its clinical use. Importantly, braces did not reduce pain, questioning their role in conservative management of acute painful VFs. Finally, only few high-quality studies that investigated analgesics (NSAIDs, opioids) were available, highlighting the urgent need for further research.

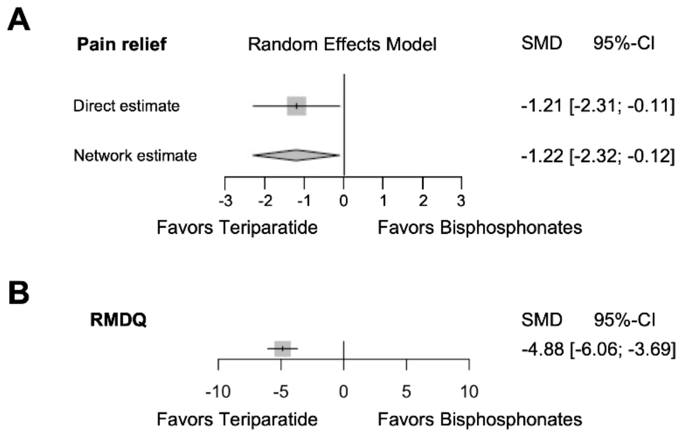

**COP18**

**Bone material properties in male patients with "idiopathic osteoporosis"**

Markus Hartmann[1], Stéphane Blouin[1], Daniel A. Kraus[1], Martina Behanova[1], Sonja Lueger[1], Petra Keplinger[1], Judith Haschka[1], Jochen Zwerina[1], Roland Kocijan[1]
[1]Ludwig Boltzmann Institute of Osteology at Hanusch Hospital of OEGK and AUVA Trauma Centre Meidling, 1st Med. Dept. Hanusch Hospital, Vienna, Austria

**Abstract Text**

**Introduction:** Male idiopathic osteoporosis (MIO) refers to primary osteoporosis with unclear etiology. Clinical characteristics of MIO are a reduced bone mass and/or the occurrence of fragility fractures.

**Purpose:** We study histomorphometry, bone matrix mineralization and osteocyte lacunae characteristics in bone samples from patients with MIO to detect similarities in bone material properties in this inhomogeneous group.

**Methods:** Transiliac bone biopsy samples were analyzed from 27 male patients with osteoporosis after exclusion of secondary reasons for fragility fractures (mean age 46±13 years). Undecalcified samples were embedded in PMMA. 3 µm thick sections were stained with Goldner’s trichrome for histomorphometrical analysis to obtain structural parameters and static parameters of bone formation and resorption. Twenty of the patients received double tetracycline labelling prior to the biopsy for assessment of dynamic parameters of bone formation. Bone mineralization density distribution and osteocyte lacunae sections (OLS) were assessed on the residual sample block using quantitative backscattered electron imaging.

**Results:** The MIO group showed reduced bone volume per tissue volume ($p=0.002$), reduced trabecular thickness ($p<0.001$) and reduced trabecular number ($p=0.03$) compared to age matched controls. Reductions in osteoid volume ($p=0.003$), osteoid thickness ($p<0.001$) and osteoblast surface were observed ($p<0.001$). The double labeling showed a decrease in adjusted apposition rate ($p<0.001$) and an increase in mineralization lag time ($p=0.001$). We found hypomineralization of trabecular bone of the MIO group compared to the reference group. In particular, the mean calcium content was reduced (-3 %, $p=0.001$). No difference in mineralization was detected in the cortical compartment. Osteocyte lacunar density and area were not significantly different between MIO and control group neither in trabecular nor in cortical compartment.

**Conclusions:** Bone material in patients with MIO is characterized by an altered bone structure due to a decrease in turnover leading also to hypomineralization of the bone matrix, explaining the high fracture risk in MIO patients.

**Concurrent Oral Presentations 2: Bone Biology and Biomechanics**

**COP19**

**The beta2-adrenoreceptor is essential for bone repair in adult mice.**

Paul-Richard Knapstein[1], Denise Jahn[2], Ellen Otto[2], Paul Köhli[2], Jan Sevecke[1], Shan Jiang[1], Weixin Xie[1], Anke Baranowsky[1], Johannes Keller[1]

*[1]University Medical Center Hamburg-Eppendorf, Department of Trauma and Orthopedic Surgery, Hamburg, Germany ; [2]Charité – Universitätsmedizin Berlin, Center for Musculoskeletal Surgery, Berlin, Germany*

**Abstract Text**

**Background:** Impaired fracture healing is a major public health concern as the number of age-related fractures continues to rise. The beta2-adrenergic receptor (Adrb2), a major component of the sympathetic nervous system, has previously been shown to suppress bone formation and promote bone resorption in adults, where the sympathetic tone is higher than in the young organism. In this context, genetic or pharmacological inactivation of Adrb2 resulted in high bone mass, identifying this G-Protein coupled receptor as a promising target for the treatment of bone loss disorders. However, the role of Adrb2 in fracture healing remained largely unknown.

**Purpose:** The study was designed to test a potential function of Adrb2 in fracture healing in young and adult mice.

**Methods:** Adrb2-deficient mice and littermate controls were subjected to femoral osteotomy (Fx), stabilized with an external fixator, at 12 (young) or 30 (adult) weeks of age. Radiological and histological analyses were performed at early (day 7), intermediate (day 14), and late (day 21) stages of bone regeneration. Expression of the pro-angiogenic factor vascular endothelial growth factor A (Vegfa) and the markers of osteogenic type H vessels Cd31 and Endomucin were assessed by qRT-PCR and immunofluorescence.

**Results:** Young Adrb2-deficient mice with low sympathetic tone showed normal bone architecture and only minor alterations in fracture healing. In contrast, adult Adrb2-deficient mice with elevated sympathetic tone showed increased trabecular bone mass in the spine and femur, whereas fracture repair was severely impaired and characterized by atrophic nonunion with diminished cellular callus remodeling. As an explanation, a strong reduction of Vegfa expression was observed in the periosteum of adult Adrb2-deficient mice, correlating with a decreased formation of osteogenic type H vessels.

**Conclusions:** While only of minor importance in the young organism with low sympathetic tone, Adrb2 plays a pivotal role in bone regeneration in adult mice.

### Figure 1

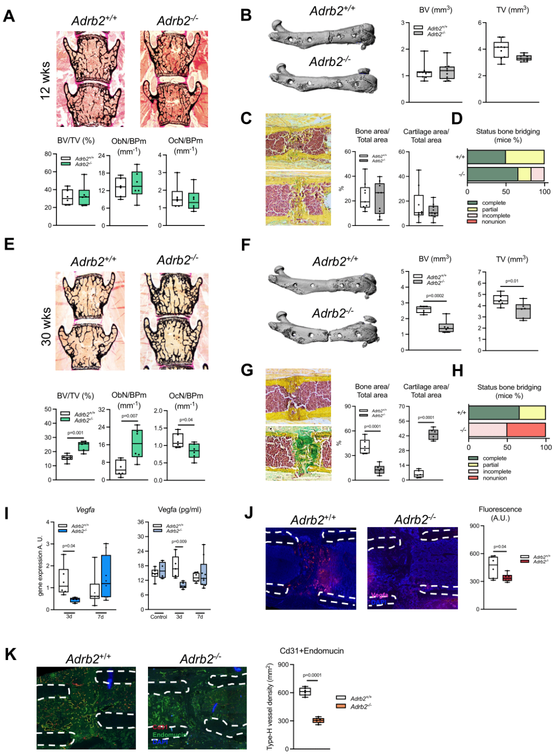

**Figure 1**. The beta2-adrenergic receptor is essential for bone healing in adult mice. **A)** Von Kossa/van Gieson staining of undecalcified vertebra sections (L3 and L4) and histomorphometry of bone volume per tissue volume (BV/TV), osteoblast numbers/bone perimeter (ObN/BPm), and osteoclast numbers/bone perimeter (OcN/BPm) in 12-week-old mice of the indicated genotypes. Scale bar=500 µm. **B)** Representative µCT images of the fractured femora in 12-week-old mice of the indicated genotypes 21 days following fracture and quantification of callus bone volume (BV) and tissue volume (TV). Scale bar=3 mm. **C)** Representative callus sections (Movat Pentachrome staining) in the same mice and quantification of bone area/total area and cartilage area/total area. Scale bar=500 µm. **D)** Semiquantitative evaluation of osseous callus bridging in the same mice. **E)** Von Kossa/van Gieson staining of undecalcified vertebra sections (L3 and L4) and histomorphometry of BV/TV, ObN/BPm, and OcN/BPm in 30-week-old mice of the indicated genotypes. Scale bar=500 µm. **F)** Representative µCT images of the fractured femora in 30-week-old mice of the indicated genotypes 21 days following fracture and quantification of callus BV and TV. Scale bar=5 mm. **G)** Representative callus sections in the same mice and quantification of bone area/total area and cartilage area/total area. Scale bar=500 µm. **H)** Semiquantitative evaluation of osseous callus bridging in the same mice. **I)** Expression of Vegfa in the callus 3 and 7 days after osteotomy in 30-week-old mice of the indicated genotypes. Serum Vegfa levels in the same mice and untreated controls at the indicated time points. **J)** Representative immunofluorescent stainings using a Vegfa-specific antibody in the fracture callus of 30-week-old mice of the indicated genotypes 7 days after osteotomy, and quantification of signal intensity in the ROI. Scale bar=300 µm. **K)** Quantification of type H vessel density and representative immunofluorescent co-stainings using Cd31- and Endomucin-specific antibodies in the fracture callus of 30-week-old mice of the indicated genotypes 14 days after osteotomy. Scale bar=400 µm. Two-way ANOVA followed by Tukey's post-hoc-test and two-tailed Student's t-test were performed for data analysis. All experiments were approved by the local animal care committee.

### COP20

### Lysosomal enzymes in mature osteoblasts and osteocytes limit collagen width and mineral accrual in bone tissue

*Martha Blank[1], Blessing Crimeen-Irwin[1], Cameron J Nowell[2], T John Martin[1,3], Laura E Edgington-Mitchell[4], Natalie A Sims[1,3]*
*[1]St. Vincent's Institute of Medical Research, Bone Biology and Disease Unit, Melbourne, Australia ; [2]Drug Discovery Biology Theme, Monash Institute of Pharmaceutical Sciences, Melbourne, Australia ; [3]St. Vincent's Hospital, Department of Medicine- The University of Melbourne, Melbourne, Australia ; [4]Bio21 Molecular Science and Biotechnology Institute, Department of Biochemistry and Molecular Biology- The University of Melbourne, Melbourne, Australia*

### Abstract Text

Lysosomes are acidic vesicles containing enzymes, including cysteine proteinases. Both acid and enzymes degrade cytoplasmic contents and mediate degradation of collagen and mineral by osteoclasts, but their function in the osteoblast lineage is unknown. We sought to determine whether lysosome deficiency in osteoblasts and osteocytes leads to collagen and mineral accumulation *in vivo* and *in vitro*. To do this, we used EphrinB2 (*Efnb2*)-deficient Ocy454 osteocyte-like cells which have a low lysosome content, and tibiae from mice with targeted *Efnb2*-deficiency in osteocytes, which have bone fragility due to high mineral and collagen levels.

In tibiae, immunohistochemistry for Lysosomal Associated Membrane Protein 1 (LAMP1) revealed that *Efnb2*-deficient osteocytes had 34% fewer LAMP1-positive osteocytes compared to controls (p=0.0246). Second harmonic generation microscopy revealed that collagen fibres in *Efnb2*-deficient bones had significantly greater fibre width than controls (p=0.0424), indicating that the greater collagen levels includes formation of thicker fibers. Wide angle X-ray scattering analysis on bones from control and *Efnb2*-deficient mice did not detect an effect of *Efnb2*-deficiency on mineral crystal length. This confirmed an association between EphrinB2 and lysosome deficiencies in osteocytes *in vivo,* with formation of thicker collagen fibres, and mineral accumulation in bones of *Efnb2*-deficient mice.

In the Ocy454 osteocyte-like cell line, 7 days of differentiation was sufficient to express osteoblast (*Collagen1a1, Osterix*) but not osteocyte markers (*Sclerostin*). Treatment with a broad lysosomal enzyme inhibitor (bafilomycin A1) or with a specific cysteine proteinase inhibitor (E-64) led to increased mineral accumulation over 48 hours (by 58%-p=0.0002 and 74%-p=0.0025, respectively), suggesting that lysosomal enzymes limit mineral accumulation mediated by osteoblasts.

These data suggest that EphrinB2 in osteoblasts and osteocytes limits mineral accrual and collagen fibre width through lysosomal enzymes, including cysteine proteinases. This suggests osteoblast lineage-derived lysosomes function to preserve bone strength by limiting aggregation of collagen and mineral within the bone matrix.

**COP21**

**Unveiling Age- and Sex-Specific Impairments in Mechanical Adaptation of Prematurely Aged PolgA Mice**

*Dilara Yilmaz[1], Francisco Marques[1], Boaretti Daniele[1], Gian Schaedli[1], Danielle Whittier[1], Neashan Mathavan[1], Amit Singh[1], Esther Wehrle[2], Gisela Anna Kuhn[1], Ralph Mueller[1]*
*[1]ETH Zurich, Institute for Biomechanics, Zurich, Switzerland ; [2]AO Research Institute, Davos Platz, Davos, Switzerland*

**Abstract Text**

**Introduction:** Age-related osteoporosis results from an imbalance between bone formation and resorption. Our study, utilizing *in vivo* mechanical loading, showcased enhanced bone adaptation in wild-type mice. However, in the prematurely aging PolgA$^{D257A/D257A}$ (PolgA) mouse model displaying musculoskeletal aging phenotypes such as frailty and osteosarcopenia, it remains unclear how aging bone responds to changes in the mechanical environment and whether these changes are sex-dependent.

**Purpose:** We investigate the age- and sex-specific responses to *in vivo* mechanical loading in PolgA mice for both sexes.

**Methods:** Stainless steel pins were inserted into the 5$^{th}$ and 7$^{th}$ caudal vertebrae of young (12 weeks) and aged (34 weeks) PolgA mice. A 4-week loading regime (8-10N, 5 min, 3x/week) was applied to the 6$^{th}$ caudal vertebra of female (n = 14, 18) and male (n=19, 24) young and aged PolgA mice, comparing to the sham group (license ZH036/2022). Time-lapsed *in vivo* micro-CT imaging was performed on CV6 (10.5 µm resolution) from which morphometric parameters were evaluated.

**Results:** Cyclic mechanical loading significantly enhanced osteogenic responses in young PolgA mice, particularly in females (Fig 1A). ΔBV/TV, the absolute difference between the last and the initial time point, increased with loading in young males (5.43±2.09%, p=0.0198) and females (16.11±3.56%, p=0.0003), while loading in aged PolgA mice showed no significant changes (males: 0.92±1.13%; females: 2.35±4.48%). Net remodeling rate, the difference between bone formation rate (BFR) and bone resorption rate (BRR), notably increased with loading in young males (p<0.01) and females (p<0.001) indicating positive remodeling, while loading in aged PolgA mice resulted in negative remodeling associated with bone loss (Fig 1B).

**Conclusion:** Our findings demonstrate that cyclic mechanical loading significantly enhances bone adaptation in young PolgA mice, with more pronounced effects in females. However, as the mice age, the adaptive response of bone to mechanical stimuli is impaired similarly for both sexes.

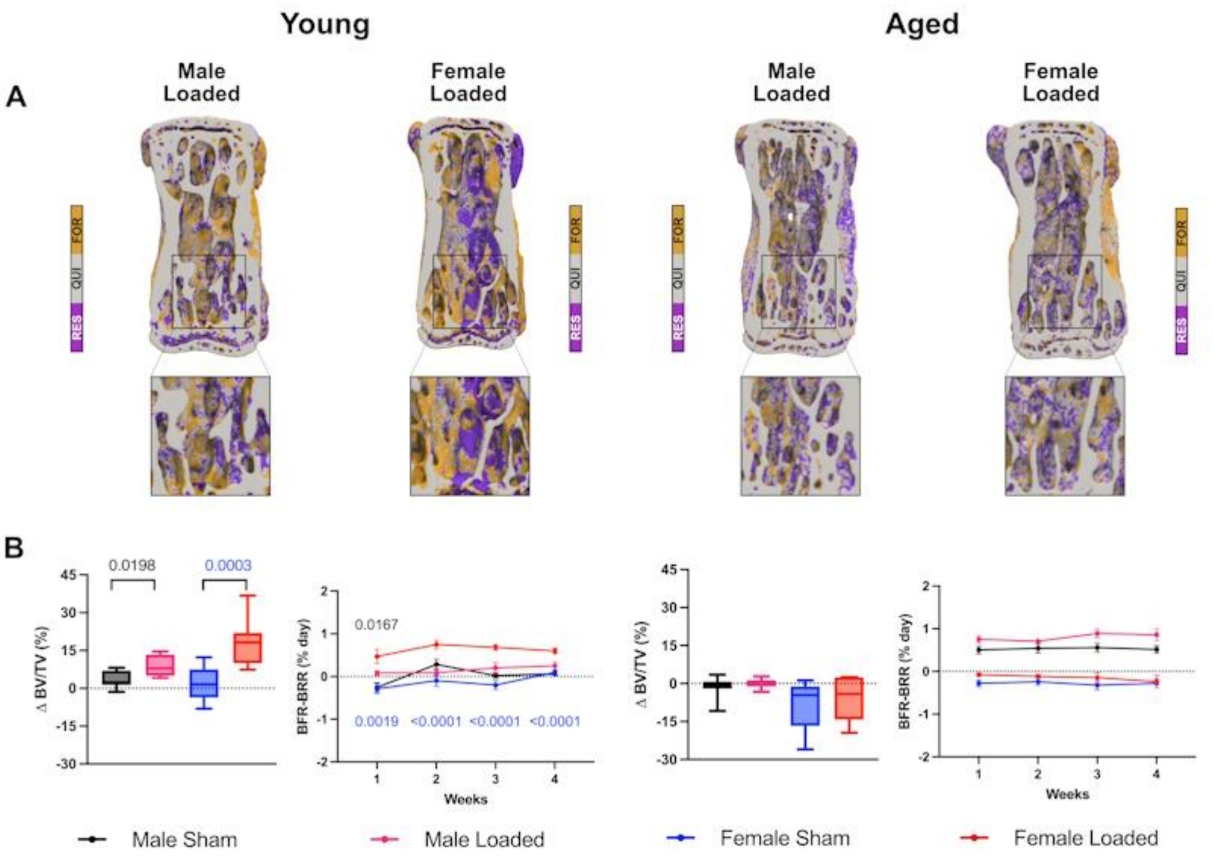

**Figure 1:** A) Visualization of bone remodeling sites (formation, quiescence, and resorption) in the loaded caudal vertebra in vivo. Overlay of time-lapsed micro-CT image showing sites of bone formation (orange), quiescence (grey), and resorption (purple) at week 2. B) Changes in the bone volume fraction from week 0-4 and net remodeling rate over the 4-week loading period. Mann Whitney and 2way ANOVA with Šidák's multiple comparison test are used. Data are presented as mean ± s.e.m. Exact p values are indicated as black for males, blue for females directly in the figure .

### COP22

### Neutrophil extracellular traps are targetable mediators of impaired bone healing in mice

*Lilly-Charlotte Albertsen[1], Weixin Xie[1], Shan Jiang[1], Jan Sevecke[1], Ruben Augustin[1], Annika Heuer[1], Anke Baranowsky[1], Johannes Keller[1]*
*[1]University Medical Center Hamburg-Eppendorf, Department of Trauma and Orthopedic Surgery, Hamburg, Germany*

**Abstract Text**

**Background:** Impaired fracture healing is observed in up to 15% of trauma patients, necessitating the identification of novel therapeutic approaches. After injury, the fracture hematoma is rapidly infiltrated by neutrophils, which are capable of extruding neutrophil extracellular traps (NETs). This process of NETosis is dependent on the NETs generator PAD4 and can be resolved by DNAse1- and DNAse1-like3, together representing an evolutionarily highly conserved mechanism of the innate immune system aimed at killing bacteria. Although NETosis can also be triggered by trauma and NETs components may affect bone cell function, a role for NETs in fracture healing remains unknown.

**Objectives:** To define the role of NETs in a murine fracture and/or nonunion model using genetic and pharmacological means that alter NETosis.

**Methods:** WT, combined DNAse1- and DNAse1-like3-deficient (hereafter referred to as DNAse1/3), and combined DNAse1/3;PAD4-deficient mice were subjected to femoral osteotomy stabilized by an external fixator to assess physiologic fracture healing (Fx). To model pathologic Fx, the periosteum of the fracture ends was cauterized in the same model to induce nonunion. Bone healing in both models was monitored by qRT-PCR, ELISA, micro-CT and undecalcified histology.

**Results:** In WT mice, Fx was associated with increased expression of the NETs generator PAD4 in callus tissue and increased serum levels of released NETs components. Mice lacking DNAse1/3 showed impaired fracture healing and high rates of fracture nonunion, which were completely abolished by additional deletion of PAD4. In the nonunion model, DNAse1/3-deficiency impaired fracture consolidation, which was rescued and even significantly improved in DNAse1/3;PAD4-deficient mice compared to WT mice. Finally, pharmacological targeting of NETs resolution by FDA-approved recombinant DNAse1 improved fracture consolidation in both normal Fx and nonunion models.

**Conclusion:** The release of neutrophil extracellular traps impairs fracture union in both physiologic and pathologic healing states and can be targeted pharmacologically to improve bone healing in mice.

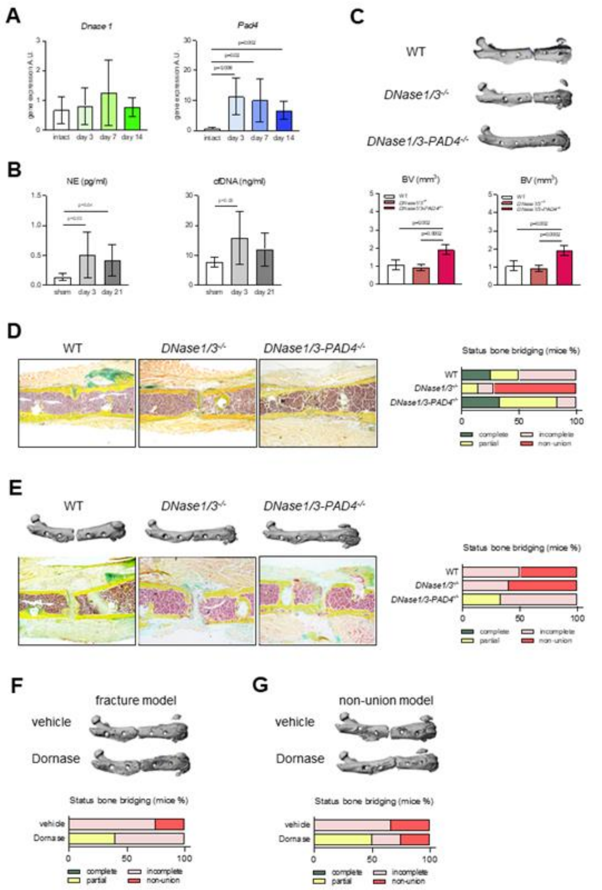

**Figure 1.** NETs impair bone healing in mice. **A)** Gene expression (qRT-PCR) of the indicated genes in uninjured bone (intact)and the callus at the indicated time points after surgery in WT mice. AU=arbitrary units. **B)** Serum levels of the NETs components neutrophil elastase (NE) and cell-free DNA (cfDNA) in the indicated groups. **C)** Representative µCT images of the fractured femora 21 days following surgery in the indicated mice and and quantification of bone volume (BV) and tissue volume (TV) in the callus. **D)** Representative callus sections (Movat Pentachrome staining) 21 days after surgery (yellow=mineralized bone; green=cartilage) and semiquantitative evaluation of osseous callus bridging in the same mice. **E)** Representative µCT images and callus sections (Movat Pentachrome staining) 28 days after femoral osteotomy and cauterization of fracture ends (nonunion model) in the indicated mice. The semiquantitative evaluation of osseous callus bridging in the same mice is shown on the right. **F)** Representative µCT images and semiquantitative evaluation of osseous callus bridging 14 days after osteotomy and **G)** 28 days after osteotomy and additional cauterization of fracture ends in WT mice treated daily with Dornase alpha. One-way ANOVA followed by Tukey's post-hoc-test was performed for data analysis. For all experiments, n = 5-6 mice per group. All experiments were approved by the local animal care committee.

### COP23

### Family-based whole-exome sequencing implicates a variant in lysyl oxidase like 4 in atypical femur fractures

*Wei Zhou[1], Denise M. Van de Laarschot[1], Jeroen G.J. Van Rooij[1], Marijke Koedam[1], Hanh H Nguyen[2], André G. Uitterlinden[1], Peter R. Ebeling[2], Rajesh V Thakker[3], Piet Geusens[4], Bram C.J. Van der Eerden[1], Annemieke J.M.H. Verkerk[1], M. Carola Zillikens[1]*

*[1]Erasmus Medical Center, Internal Medicine, Rotterdam, Netherlands ; [2]School of Clinical Sciences-Monash University, Department of Medicine, Clayton, Australia ; [3]Academic Endocrine Unit University of Oxford, Radcliffe Department of Medicine, Oxford, United Kingdom ; [4]Maastricht University, Department of Internal Medicine, Maastricht, Netherlands*

### Abstract Text

Atypical femur fractures (AFFs) are rare adverse events associated with bisphosphonate use, having unclear pathophysiology. AFFs also cluster in families and have occurred in patients with monogenetic bone diseases sometimes without bisphosphonate use, suggesting an underlying genetic susceptibility. Our aim was to identify a genetic cause for AFF in a Caucasian family with seven members affected by osteoporosis, including three siblings with bisphosphonate-associated AFFs. Using whole-exome sequencing, we identified a rare pathogenic variant c.G1063A (p.Gly355Ser) in lysyl oxidase like 4 (*LOXL4*) among 64 heterozygous rare, protein-altering variants shared by the three siblings with AFFs. The same variant was also found in a fourth sibling with a low-trauma femur fracture above the knee, not fulfilling all the ASBMR criteria of AFF and in one of 73 unrelated European AFF patients. LOXL4 is involved in collagen cross-linking and is likely to be relevant for bone repair mechanisms. Skin fibroblast-derived osteoblasts from the unrelated patient with the *LOXL4* variant expressed less collagen type I and elastin, while osteogenic differentiation and mineralization were enhanced compared with controls. In conclusion, this *LOXL4* variant may underlie AFF susceptibility possibly due to abnormal collagen metabolism leading to compromised healing of microcracks in the femur.

**COP24**

**GWAS meta-analysis of bone stiffness indices in 544,864 individuals identifies new candidate genes that may control the structural integrity of bone and susceptibility to fracture.**

*Kaitlyn Flynn[1], Yu-Ting Yan[2], Dorothy Hsia[2], Ryan Chai[3], Yuandan Zhang[1], Shih-Jen Tsai[4], Fernando Rivadeneira[5], Carolina Medina-Gomez[5], Robert Blank[6], Duncan Basset[7], Graham Williams[7], Cheryl Ackert-Bicknell[8], David Evans[9], Jonathan Tobias[10], Peter Croucher[3], Douglas Kiel[11], Loic Yengo[9], Nicole Warrington[9], Po-Hsiu Kuo[2], John Kemp[1]*
*[1]University of Queensland, Mater Research Institute, Brisbane, Australia ; [2]National Taiwan University, Institute of Epidemiology and Preventative Medicine, Taipei, Taiwan R.O.C. ; [3]Garvan Institute of Medical Research, Bone Biology, Sydney, Australia ; [4]National Yang Ming Chiao Tung University, Division of Psychiatry, Taipei, Taiwan R.O.C. ; [5]Erasmus MC, Department of Internal Medicine, Rotterdam, Netherlands ; [6]Garvan Institute of Medical Research, Immunometabolism Laboratory, Sydney, Australia ; [7]Imperial College London, Molecular Endocrinology Laboratory, London, United Kingdom ; [8]University of Colorado, Department of Orthopedics, Aurora, United States ; [9]University of Queensland, Institute for Molecular Bioscience, Brisbane, Australia ; [10]University of Bristol, Musculoskeletal Research Unit, Bristol, United Kingdom ; [11]Hebrew SeniorLife Marcus Institute for Aging Research, Harvard Medical School, Boston, United States*

**Abstract Text**

**Background:** Quantitative ultrasound (QUS) evaluates bone structural integrity by measuring changes in the speed and attenuation of sound waves. These parameters are combined to form indices that predict bone fractures independent of BMD and FRAX. Indices include a "stiffness index'' (SI) for Lunar devices and a "quantitative ultrasound index" (QUI) for Hologic systems. SI and QUI are heritable, yet the underlying genetic determinants remain uncharacterised.

**Purpose:** To quantify the genetic similarity of SI and QUI, identify shared genetic determinants, and evaluate their relationship with fractures.

**Methods:** Genome-wide association studies (GWAS) were conducted on standardised measures of QUI in the UK-Biobank (N=447,873) and SI in the Taiwan Biobank (N=96,991). Pairwise genetic correlations correcting for genetic ancestry were estimated. QUI and SI GWAS were meta-analysed using a random effects method and clumped with ancestry-matched data. Associated variants were followed-up in GWAS of hip and forearm fractures. The closest protein coding gene to each associated variant was followed up in a single-cell transcriptomics dataset of murine bone and marrow cells, and the MGI mutant mouse database.

**Results:** QUI and SI were highly genetically correlated rg=0.83($CI_{95}$:0.73–0.92). 1,637 associated variants were identified ($p_{meta}<5\times10^{-8}$). 31 were robustly associated with forearm fractures ($p<5\times10^{-8}$), but not with hip fractures (p>0.05, Figure 1). 58 were located >1mb away from known DXA/QUS GWAS loci and deemed novel. Four novel variants were associated with forearm fractures [*RYBP, CACNB2, IGF1,* and *HMGA1* (p<0.05)], and 2 with hip fractures (*DRG1* and *HMGA1*). Genes closest to novel variants were differentially expressed in osteoblasts (e.g., *CDH11*), osteoclasts (*S100A10*), endothelial (*FKBP1C*), and vascular cells (*CRIM1*). *CRIM1* resulted in abnormal skeletal phenotypes when mutated in mice.

**Conclusions:** Genetic determinants of QUI and SI are largely shared and highlight that such phenotypes enable the discovery of genes that may control bone structural integrity and fracture susceptibility.

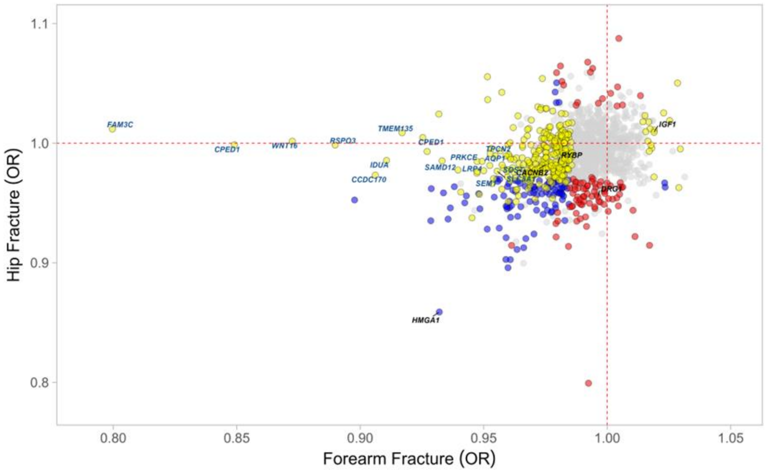

**Figure 1.** Scatter plot contrasting the genetic effect of QUI/SI increasing variants on risk of hip fractures (~11k cases/723k controls) and forearm fractures (~50k cases/ 969k controls).

Each circle corresponds to an independent QUI/SI associated genetic variant. Blue circles correspond to variants associated with hip and forearm fractures (p < 0.05). Yellow circles correspond to variants associated with forearm fractures (p < 0.05) but not hip fractures (p > 0.05). Red circles correspond to variants associated hip fractures (p < 0.05), but not forearm fractures (p > 0.05). Grey circles correspond to variants that are not associated with hip or forearm fractures (p > 0.05). Variants associated with forearm fracture ($p < 5\times10^{-8}$), but not hip fracture (p > 0.05) are annotated with the name of the closest gene in blue. Novel variant associations that are also associated with hip or forearm fracture are annotated with the name of the closest gene in black.

### Concurrent Oral Presentations 3: Disease Trajectories and Assessment

### COP25

### Abdominal aortic calcification assessed in DXA-derived images using machine learning predicts increased injurious falls risk in older adults: the Manitoba Bone Mineral Density Registry

*Marc Sim[1,2], Abadi Gebre[1], Jack Dalla Via[1], Cassandra Smith[1], Siobhan Reid[3], Mohammad J Jozani[4], Douglas Kimelman[5], Barret A Monchka[6], Syed Z Gilani[7], Zaid Ilyas[7], John T Schousboe[8,9], Joshua R Lewis[1,2], William D Leslie[10]*
*[1]Edith Cowan University, Nutrition & Health Innovation Research Institute, Perth, Australia ; [2]The University of Western Australia, Medical School, Perth, Australia ; [3]University of Manitoba, Department of Electrical and Computer Engineering, Winnipeg, Canada ; [4]University of Manitoba, Department of Statistics, Winnipeg, Canada ; [5]University of Manitoba, Department of Radiology-Rady Faculty of Health Sciences, Winnipeg, Canada ; [6]University of Manitoba, George and Fay Yee Centre for Healthcare Innovation, Winnipeg, Canada ; [7]Edith Cowan University, Centre for AI&ML-School of Science, Perth, Australia ; [8]HealthPartners, Park Nicollet Clinic and HealthPartners Institute, Minneapolis, United States ; [9]University of Minnesota, Division of Health Policy and Management,*

*Minneapolis, United States ; [10]University of Manitoba, Departments of Medicine and Radiology, Winnipeg, Canada*

**Abstract Text**

**Background:** Abdominal aortic calcification (AAC), a subclinical measure of vascular disease that can be assessed during vertebral fracture assessment (VFA), has been identified as a falls risk factor. A major clinical limitation is that the 24-point AAC scoring (AAC24) requires trained experts and is time consuming.

**Purpose:** Determine if our machine learning AAC algorithm (AAC24-ML) from DXA-derived VFA images is associated with higher incident hospitalised falls risk in the Manitoba Bone Mineral Density Registry.

**Methods:** 8565 individuals (91.2% female, mean±SD 75.7±6.7 years) who had a bone mineral density and VFA test between March 2010 and December 2017 were included. Our AAC24-ML algorithm has been validated for cardiovascular outcomes in three categories (AAC24-ML= Low <2; Moderate 2-<6; Severe ≥6). Cox-proportional hazards models assessed the relationship between AAC24-ML and incident fall-related hospitalisations obtained from linked health records over a mean±SD follow-up of 3.9±2.2 years.

**Results:** Individuals with Severe-AAC24-ML recorded the highest proportion (11.7%) of fall-related hospitalisations (**Table**). Compared to Low-AAC24-ML, individuals with Moderate- (HR=1.49) and Severe-AAC24-ML (HR=1.89) had greater hazards for a fall-related hospitalisation. Point estimates were slightly lower (HR=1.39 and 1.63, respectively) after multivariable adjustment. Wald statistics indicated the top five contributors in our multivariable-adjusted model were age ($\chi^2$=116.9), Severe-AAC24-ML ($\chi^2$=21.4), loop diuretics ($\chi^2$=15.6), anti-coagulant ($\chi^2$=12.0) and tobacco use ($\chi^2$=11.3).

**Conclusion:** AAC24-ML can efficiently screen for injurious falls risk among patients undergoing VFA during routine osteoporosis assessments.

**Table.** Hazard ratios (95%CI) for incident fall-related hospitalisations by predicted AAC severity.

| AAC24-ML severity | *Falls, n(%)* | *Age + sex adjusted* | *Multivariable-adjusted[1]* |
|---|---|---|---|
| *Low- n=3400* | 205 (6.0) | Ref (1.0) | Ref (1.0) |
| *Moderate- n=2840* | 272 (9.6) | 1.49 (1.24-1.79)* | 1.39 (1.15-1.67)* |
| *Severe- n=2325* | 273 (11.7) | 1.89 (1.56-2.28)* | 1.63 (1.34-1.99)* |

[1]adjusted for age, sex, ethnicity, BMI, tobacco use, alcohol intake, diabetes, hypertension, socioeconomic status, glucocorticoids, numerous cardiovascular medications and falls in the last year. *indicates p<0.05.

**COP26**

**Dissecting the genetic architecture of trabecular bone score: a genome-wide association study in 45 thousand individuals.**

*Haojie Lu[1], John P.Kemp[2], Griffin Tibbitts[3], Monika Frysz[4], Gloria Hoi-Yee Li[5], Ching-Lung Cheung[6], Christine Lary[3], Douglas P. Kiel[7], Jonathan H. Tobias[4], Fernando Rivadeneira[1], Carolina Medina-Gomez[1], On behalf of the GEFOS/CHARGE Consortium[1]*
*[1]Erasmus MC, Internal Medicine, Rotterdam, Netherlands ; [2]University of Queensland- University of Bristol, Institute for Molecular Bioscience- University of Queensland- Diamantina Institute- University of Queensland- Medical Research Council Integrative Epidemiology Unit- University of Bristol, Brisbane, Aust ; [3]Northeastern University, Roux Institute, Boston, United States ; [4]University of Bristol, Medical Research Council Integrative Epidemiology Unit- University of Bristol- Musculoskeletal Research Unit- University of Bristol, Bristol, United Kingdom ; [5]The Hong Kong Polytechnic University, Department of Health Technology and Informatics, Hong Kong, China ; [6]the University of Hong Kong, Department of Pharmacology and Pharmacy-, Hong Kong, China ; [7]Hebrew Senior Life Boston-Harvard Medical School, Hinda and Arthur Marcus Institute for Aging Research- Hebrew Senior Life Boston- Department of Medicine- Beth Israel Deaconess Medical Center- Harvard Medical School, Boston, United States*

**Abstract Text**

**Background:** Trabecular bone score (TBS) is a texture-based technique evaluating the pixel gray-level variations in lumbar spine DXA scans. It is correlated with trabecular number and connectivity in CT scans and shown to be associated with vertebral fracture risk independent of bone mineral density (BMD). While genetic determinants of BMD have been largely explored, the factors underlying TBS variation remain to be elucidated.

**Aim:** To conduct a large genome-wide association study (GWAS) of TBS to identify genes influencing lumbar spine microarchitecture.

**Methods:** We performed the GWAS analysis (n=44,767 from 8 studies, 95% of European background) adjusted by sex, age, and genomic principal components and meta-analysis using an inverse variance weighted model. We conducted conditional analyses using GCTA. LD-score regression was performed to calculate TBS SNP-based heritability and evaluate genetic correlations ($r_g$).

**Results:** We identified a total of 33 genome-wide conditionally independent variants, which explained 4.1% of the TBS-variance with the SNP-heritability of 0.20 (se: 0.02). All signals were mapped to BMD-associated loci and were not independent. TBS was genetically correlated with lumbar spine ($r_g$: 0.90, P-value: $9.3 \times 10^{-50}$) and femoral neck (gr: 0.81, P-value: $4.79 \times 10^{-43}$) BMD, and negatively correlated with hip ($r_g$: -0.46, P-value: $1.23 \times 10^{-8}$), forearm ($r_g$: -0.42, P-value: $2.17 \times 10^{-12}$) and any-type ($r_g$: -0.46, P-value: $6.70 \times 10^{-16}$) of fracture risk. Sex-specific analysis did not identify variants with sex-differential effects.

**Conclusion:** We identified variants mapping to 33 loci associated with TBS, all of which are also associated with BMD. TBS is genetically correlated with BMD and fracture risk demonstrating its utility to assess skeletal fragility. Larger studies are required to identify any TBS-specific loci that can provide insight into the biological mechanisms underlying fracture risk and the identification of potential drug targets to improve bone microarchitecture.

**COP27**

**Assessment of Romosozumab (Romo) Treatment on Spine Bone Strength Using Biomechanical Computed Tomography (CT) Virtual Stress Tests**

*Tony M. Keaveny[1], Mary Oates[2], Donald Betah[2], Yifei Shi[2], Jen Timoshanko[3], Corinna C. Zygourakis[4], Mohamad Bydon[5], Kelly Krohn[6], Koji Ishikawa[7]*
*[1]University of California at Berkeley, Departments of Mechanical Engineering and Bioengineering, Berkeley, United States ; [2]Amgen Inc., Bone Franchise, Thousand Oaks, United States ; [3]UCB Pharma, Bone Health, Slough, United Kingdom ; [4]Stanford University School Medicine, Department of Neurosurgery, Palo Alto, United States ; [5]Mayo Clinic, Mayo Clinic Neuro-Informatics Laboratory, Rochester, United States ; [6]The CORE Institute, The CORE Institute, Phoenix, United States ; [7]Showa University School of Medicine, School of Medicine, Tokyo, Japan*

**Abstract Text**

**Background**: Osteoporosis (OP) is a significant risk factor for post-surgical complications in patients undergoing spinal fusion. Romo, a sclerostin inhibitor, has been shown to increase bone mineral density (BMD) and vertebral strength and reduce fracture risk.

**Purpose:** Assess pedicle screw-bone connection in pullout tests and physiological spinal loads using Finite Element (FE) analysis with different OP treatments.

**Methods**: This retrospective analysis used lumbar spine CT data from postmenopausal women with low BMD or OP in the imaging substudies of the Phase 2 (Ph2; NCT00896532) and Phase 3 ARCH (NCT01631214) trials. In the Ph2 study, patients received Romo 210mg monthly, placebo [PBO], or teriparatide [TPTD] for 12 months (M). In ARCH, patients received Romo or alendronate [ALN] for 12M, then ALN for a second 12M. CT scans at baseline (BL) and follow-up (M12 for Ph2, M6, M12 and M24 for ARCH) were used to create a FE model at each timepoint for the L1 vertebra, virtually implanted with a pedicle screw, in a "shear pullout" configuration. Percent changes from BL in shear bone strength, volume of failed tissue ($cm^3$) associated with shear bone loading related to weak bone, and periprosthetic BMD ($mg/cm^3$) were assessed.

**Results**: 79 patients from each study were analysed (Ph2: Romo=24;PBO=27;TPTD=28. ARCH: Romo/ALN=44;ALN/ALN=35). Within-study BL characteristics were similar across treatment groups. At M12 vs BL [Ph2] and M6/M12 vs BL [ARCH], Romo significantly increased shear bone strength, decreased volume of failed tissue and increased periprosthetic BMD vs comparators (**Figure**). In ARCH, changes were maintained at M24 after switching from Romo to ALN (**Figure**).

**Conclusion**: Romo significantly increased shear bone strength by FE analysis and periprosthetic BMD and lowered the amount of failed tissue vs comparators, demonstrating the efficacy of Romo for improving lumbar spine BMD and strength associated with pedicle screw fixation.

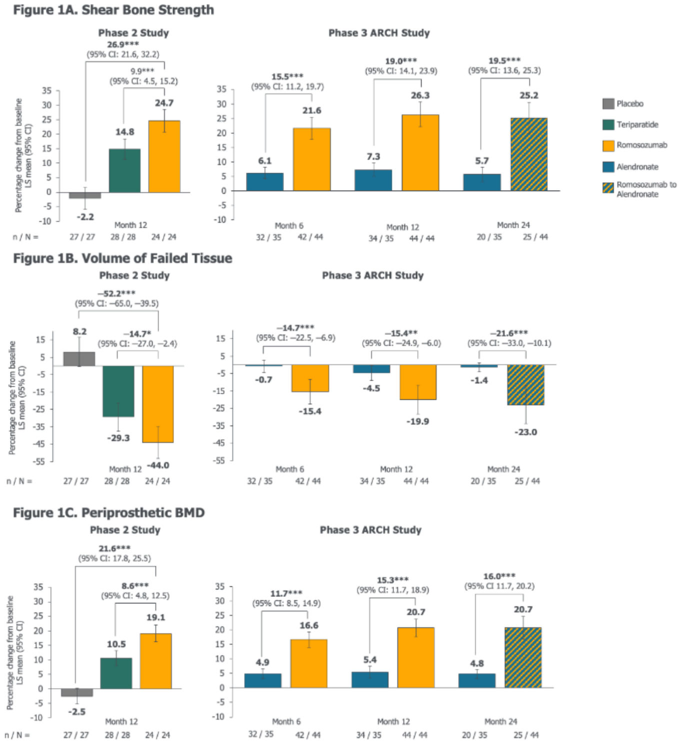

***p<0.001, **p<0.01, *p<0.05. N=Number of subjects with values at baseline and at-least one post-baseline visit. n=Number of subjects with evaluable data at the time point of interest. BMD: bone mineral density; CI: confidence interval; LS: least squares. Percentage changes from baseline were assessed by analysis of covariance model adjusting for [Phase 2 study] treatment, geographic region, and baseline value of the measure; [Phase 3 ARCH study] treatment, presence of severe vertebral fracture at baseline, and baseline value of the measure. For Phase 3 ARCH, missing values were imputed by carrying forward the last non-missing post-baseline value prior to the missing value and within the treatment period.

**Funding:** UCB Pharma and Amgen Inc.

### COP28

### Longitudinal course of volumetric hip BMD and macrostructure in postmenopausal T2D women with and without diabetic retinopathy versus controls - a prospective 5-year-quantitative computed tomography-(qCT)-study

*<u>Ursula Heilmeier</u>[1,2], Sigurdur Sigurdsson[3], Elias Gudmundsson[3], Thor Aspelund[3], Thomas Lang[2], Vilmundur Gudnason[3,4], Thomas M. Link[2], Ann V. Schwartz[5]*
*[1]ECTS Academy, Department of Rheumatology and Clinical Immunology- University Medical Center Freiburg, Freiburg, Germany ; [2]University of California San Francisco, Musculoskeletal Quantitative Imaging Research Group- Department of Radiology & Biomedical Imaging, San Francisco, United States ; [3]The Icelandic Heart Association, AGES-Reykjavik Study Coordinating Center, Kopavogur, Iceland ; [4]University of Iceland, Faculty of Medicine, Reykjavik, Iceland ; [5]University of California San Francisco, Department of Epidemiology and Biostatistics, San Francisco, United States*

### Abstract Text

**Background:** Type 2 diabetes (T2D) is characterized by an increased skeletal fragility partly attributed to microvascular disease. Diabetic retinopathy (RT) is a common microvascular complication and a potential clinical proxy of T2D bone microvascular disease. To date, clinical studies on BMD and RT were mainly cross-sectional, contradictory and limited to lower-resolution imaging-techniques such as DXA. Using qCT, we investigated prospectively the 5-year changes in qCT-derived volumetric and structural/geometrical hip bone parameters in T2D postmenopausal women with RT (DM-RT:n=16) relative to their retinopathy-free non-diabetic (Co:n=108) and T2D-peers (DM:n=54).

**Material and Methods:** 178 postmenopausal T2D and non-diabetic women of the AGES-Reykjavik Study without bone-impacting medications/conditions were included. All had proximal femoral qCT-scans (BL; 5-year-FU) and BL retinopathy-readings of each eye available. BL, FU, and absolute 5-year changes of cortical, trabecular and integral volumetric BMD (vBMD) were calculated for the three hip ROIs: entire proximal femur, trochanter and neck, along with the proximal femoral bone volume (FIVL) and the minimal cross-sectional neck area (MNCS). Ancovas were run for intergroup comparisons.

**Results:** All 3 groups showed similar age. DM-RT women had a higher BMI (p=0.022) and HbA1c than DMs (p=0.011), but a similar diabetes duration. While DM exhibited significantly higher integral BL vBMD then Co at all three hip ROIs (0.017<p<0.051), baseline cortical, trabecular and integral vBMDs were comparable between DM-RT and DM (p>0.05) at all three sites, except for MNCS (p=0.033). Longitudinally, DM-RT and DM demonstrated similar rates of vBMD change at all compartments and ROIs (p>0.05).

**Conclusion:** Our data suggest a comparable baseline vBMD-hip-status and longitudinal change in qCT-derived volumetric and structural/geometrical hip bone parameters between T2D postmenopausal women with and without diabetic retinopathy indicating that diabetic retinopathy alone may not be a suitable proxy of T2D bone microvascular disease and reinforcing the potential impact of retinopathy/microvascular-independent pathomechanisms on T2D bone fragility.

### COP29

### Accumulation of Advanced Glycation End products and Microarchitecture of Bone Tissue in Subjects with or without Type 2 Diabetes Mellitus

*Helena Bardenfleth[1,2], Jakob Starup-Linde[1,2], Katrine Hygum[1,2], Anastasiia Sadetskaia[3,4], Henrik Birkedal[3,4], Torben Harsløf[1,2], Bente Langdahl[1,2]*
*[1]Aarhus University Hospital, Dept. of Endocrinology and Internal Medicine, Aarhus, Denmark ; [2]Faculty of Health- Aarhus University, Dept. of Clinical Medicine, Aarhus, Denmark ; [3]Faculty of Natural Sciences, The Interdisciplinary Nanoscience Center iNANO, Aarhus, Denmark ; [4]Faculty of Natural Sciences, Dept. of Chemistry, Aarhus, Denmark*

### Abstract Text

**Background:** Despite comparable or increased bone mineral density (BMD) in patients with type 2 diabetes mellitus (T2DM), T2DM is associated with an increased risk of fragility fractures. Advanced glycation end products (AGEs) are proteins or lipids that accumulate in the organic matrix of bone and are increasingly present in T2DM, strongly driven by disease duration and poor glycemic control. This study aims to examine levels of AGEs in T2DM and healthy controls and evaluate the effect of the disease on trabecular bone thickness.

**Methods:** Randomly selected eligible males (>40 years, BMI<35kg/m2) were invited to participate. Disease duration$^{3}$5 years, HbA1c$^{3}$48mmol/mol throughout the last 24 months. Controls with lifelong HbA1c<45mmol/mol. Users of anti-osteoporotic drugs and systemic glucocorticoids were excluded. Skin AGE (SAF-AGE) accumulation was quantified using autofluorescence (AGE Reader™). Regional and whole-body BMD was measured using DXA. Iliac crest biopsies were obtained using Jimshidi™ and trabecular bone thickness (Tr.Th.) was measured with X-ray microtomography (µCT) and Dragonfly processing software.

**Results:** Twenty-six male T2DM (HbA1c 60.2mmol/mol [57.4-62.9]) and 26 sex-, age- (67.1yrs±6.7 vs. 67.4yrs±7.3), BMI- (28.7kg/$m^2$±4.6 vs. 28.2kg/$m^2$±3.5), and fat%- (from DXA;33.0%±4.8 vs. 33.0%±4.4) comparable nondiabetic controls were included. Mean diabetes duration was 15.4±8.0yrs. BMD showed no significant difference in regional and whole-body T-scores. T2DM had significantly higher SAF-AGE values (2.9±0.5AU vs. 2.3±0.5AU,p<0.05). No significant association was found between SAF-AGE and HbA1c or between SAF-AGE and age, diabetes duration, BMD, whole body fat percentage, BMI, and Tr. Th., respectively. No significant difference in Tr.Th. (150.1μm±45.3 vs 139.0μm±38.5).

**Conclusions:** Elevated AGEs in T2DM may explain the increased risk of fragility fractures, independent of BMD and bone structure, that is found in patients with T2DM. Skin AGE was not associated with HbA1c and may represent longstanding glycemic excursions or a longer duration of hyperglycemia than two years.

**COP30**

**A 5-yr longitudinal study of body composition tissue-specific change with aging and the association with loss of muscle strength and physical performance**

_Yi Yuan_[1], *Wen-shuang Zhang*[1], *Yandong Liu*[1], *Ling Wang*[1], *Xiaoguang Cheng*[1]
[1]*Peking University Fourth School of Clinical Medicine- Beijing Jishuitan Hospital, Department of Radiology, Beijing, China*

**Abstract Text**

**Background:** The amount and distribution of fat and the amount and composition of muscle mass are associated with health outcomes.

**Purpose:** Our objective was to examine the 5-yr changes in body composition in older individuals and explore the longitudinal associations of changes in body composition with changes in handgrip strength (HGS) and the Timed Up and Go test (TUG).

**Methods:** 120 older individuals performed baseline and follow-up HGS, TUG and quantitative CT (QCT) scans 5 years apart. Via QCT images, changes in muscle size and muscle density of the paraspinals, psoas major muscles, the pelvic muscles (extensors, external rotators, adductors, flexors and abductors), thigh muscles (anterior compartment, medial compartment and posterior compartment), changes in adipose area (subcutaneous adipose area [SAA], visceral adipose area, [VAA], and total adipose area [TAA]) and changes in liver fat accumulation from baseline to the 5-yr follow-up were compared.

**Results:** The magnitude of psoas major, abductors and posterior compartment of thigh muscles atrophy were greater in individuals over 70 years (P<0.05). In females over 70 years, VAA hypertrophied lesser (8.5 vs. 16.3, P=0.03) while liver fat content declined greater (-17.4 vs. -5.8, P<0.01). Among females, after adjustment, there were significant associations of higher changes in anterior compartment of thigh muscle size with higher changes in HGS (OR, 1.10; P=0.02), and higher changes in anterior compartment of thigh muscle size with higher changes in TUG (OR, 1.11; P=0.01). Among males, higher changes in anterior and medial compartment of thigh muscle size were associated with higher changes in HGS (OR, 1.15, 1.12; P=0.02, 0.02, respectively), and higher changes in anterior compartment of thigh muscle density were associated with higher changes in TUG (OR, 1.44; P<0.01).

**Conclusion:** CT-based body composition is associated with loss of muscle strength and physical performance, especially atrophy of thigh muscles.

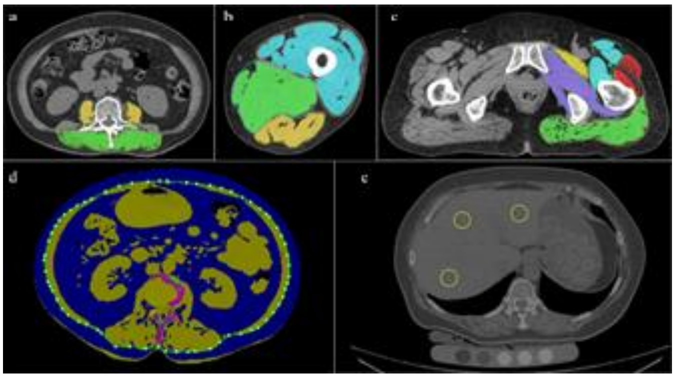

**Concurrent Oral Presentations 3: Osteoclast Biology, Osteoarthritis**

**COP31**

**Zfp687 deficiency causes altered skeletal growth due to impaired osteoclast differentiation**

*Sharon Russo*[1], *Maria Rosaria D'Angelo*[1], *Federica Scotto di Carlo*[1], *Fernando Gianfrancesco*[1]
[1]*Institute of Genetics and Biophysics, National Research Council of Italy, Naples, Italy*

**Abstract Text**

A specific gain-of-function mutation in the *ZNF687* gene was recently described as responsible for a severe form of Paget's disease of bone associated with a susceptibility to the development of Giant Cell Tumor (GCT). Mice harbouring this mutation recapitulate the human phenotype through an increase in osteoclast activity and formation. To expand the current knowledge about the mechanistic role of ZNF687 in bone metabolism, we generated the *Zfp687*-knock out (*Zfp687*$^{-/-}$) mouse model. As early as 1 month of age, we noticed that *Zfp687*$^{-/-}$ mice were visibly smaller than wild-type littermates, and such significant level of difference was maintained in 3-month-old mutant mice. We measured lengths of femurs and tibiae and found that *Zfp687*$^{-/-}$ bones were significantly reduced compared to controls (p=0.0076 and 0.0014). Since osteoclasts contribute to bone elongation, we histologically determined whether the observed skeletal phenotype was caused by an osteoclast failure. TRAP-stained femur sections revealed decreased osteoclast surface and number per bone surface in 3-month-old Zfp687$^{-/-}$ mice (p=0.010; p=0.028, respectively) (**Fig.1a-b**). To verify if the reduced osteoclastogenesis observed *in vivo* was a result of a lower availability of osteoclast precursors, we evaluated bone marrow-derived osteoclast progenitors through flow cytometry. Interestingly, preliminary results unveiled a remarkable deficiency of osteoclast progenitors in 3-month-old *Zfp687*$^{-/-}$ marrows (4.39% vs 3.03%; p=0.0006). Osteoclast activity contributes to growth plate (GP) regulation, which in turn positively governs bone elongation. Therefore, we sought to investigate whether the compromised osteoclast differentiation affects bone elongation via an impaired GP formation. Interestingly, our histological examination revealed that GPs were less wide than expected, thus reducing the positive correlation between GP and bone length ($R^2$=0.86 and $R^2$=0.69, respectively; p=0.0086). In conclusion, these data provide a clue about the role of ZNF687 in the pathophysiology of bone biology.

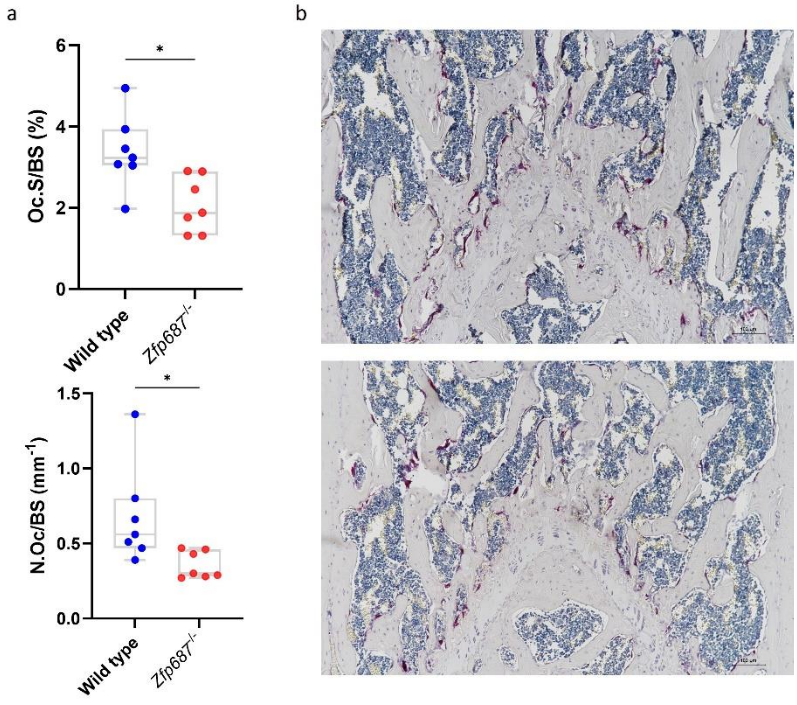

**COP32**

**Cell-autonomous osteoclast iron deficiency leads to osteopenia**

*Tiago Carvalho Oliveira*[1], *Martina Rauner*[2], *Martina U. Muckenthaler*[1], *Lorenz C. Hofbauer*[2], *Sandro Altamura*[1]
[1]*University Hospital Heidelberg, Department of Pediatric Oncology- Hematology and Immunology, Heidelberg, Germany ;* [2]*TU Dresden, Department of Medicine III & Center for Healthy Aging, Dresden, Germany*

**Abstract Text**

Maintaining optimal bone health requires a proper balance of iron levels. In fact, conditions characterized by excessive iron, such as hereditary hemochromatosis and beta-thalassemia, often lead to complications such as osteoporotic fragility fractures. Despite the clinical significance, it remains unclear whether impaired bone remodeling arises from cell-autonomous variations in bone iron content, alterations in blood iron status, or iron-related endocrine deficiencies, i.e. hypogonadism. In this study, we have generated a mouse model with iron deficiency specifically in myeloid-derived cells, including osteoclasts ($FPN_{C326S}^{LysMCre+}$), due to the presence of the Fpn p.C326S gain of function mutation in the iron exporter Ferroportin (Fpn).

$FPN_{C326S}^{LysMCre+}$ mice, analyzed at 30 weeks of age, maintain balanced hepatic and systemic iron levels despite a reduction in splenic iron content ($P<0.001$). Bone analysis revealed severe iron deficiency ($P<0.001$), with significant architectural changes ($P<0.05$): a decrease in trabecular volume and thickness and an increase in trabecular space. Additionally, dynamic histomorphometric analysis of the bone revealed an increased number of osteoclasts per total bone perimeter (N.Oc./B.Pm.) ($P<0.001$), without alterations in bone formation rate per bone surface (BFR/BS).

To understand the molecular alterations associated with the observed bone phenotype, an ex-vivo osteoclast cell culture was established. Osteoclasts derived from $FPN_{C326S}$ mice show an increase in bone resorption capacity (P<0.001) without alterations in osteoclast formation. A similar phenotype was observed in wild-type osteoclasts treated with the iron chelator DFO, indicating that the phenotype may arise from intracellular iron deficiency.

In summary, our findings demonstrate that osteoclastic iron deficiency, even in the presence of balanced systemic iron levels, is sufficient to lead to significant alterations in bone microarchitecture due to increased osteoclast activity. This work holds promising translational implications for individuals affected by iron disorders, suggesting that modulating iron levels could serve as an adjunct to available antiresorptive therapies.

**COP33**

**bone marrow adipose tissue-mediated lipolysis regulates abberant bone formation in hand Osteoarthritis**

*Léa Loisay[1], Mauro Maniglio[2], Elke Moradpour[1], Thomas Hügle[1], Jeroen Geurts[1]*
*[1]Lausanne University Hospital, Department of Rheumatology, Lausanne, Switzerland ; [2]Lausanne University Hospital, Plastic & Hand Surgery, Lausanne, Switzerland*

**Abstract Text**

**Purpose:** Bone marrow adipose tissue (BMAT) and adipocytes (BMAds) are emerging as regulators of bone formation by releasing free fatty acids and glycerol through lipolysis. However, the influence of BMAT on subchondral bone remodeling in osteoarthritis (OA) remains unclear. This study aims to explore the association between BMAT lipolysis and bone remodeling in hand OA.

**Methods:** Resection specimens were collected from patients with base of the thumb (CMC1, *n* = 20, 13 female) or distal interphalangeal OA (*n* = 4, 2 female) undergoing joint replacement. Average age and BMI were 66 ± 10 years and 25.5 ± 5.5 kg/$m^2$. Specimens were processed for histology (*n* = 13) or explant culture (*n* = 11). Bone markers (ALP, pro-collagen-Iα) and BMAd-derived factors (free fatty acids, glycerol) were measured in supernatant. BMAd size and osteoblasts were quantified in ROIs displaying low or high subchondral bone (sclerosis) and BMAT remodeling. Expression of adipose triglyceride lipase (ATGL), phosphorylated hormone-sensitive lipase (p-HSL) and monoacylglycerol lipase (MGLL) was assessed by immunohistochemistry.

**Results:** BMAd size was associated with BMI in low remodeling ROIs (Figure 1A). In sclerotic ROIs (Figure1B), BMAd size (Figure 1C) was reduced and osteoblast numbers (Figure 1D) were increased. Relative changes in BMAd size and osteoblast numbers were correlated (Figure 1E). Sclerotic ROIs displayed increased expression of lipolysis enzymes ATGL (1.7-fold), p-HSL (2-fold) and MGLL (2.9-fold) in both BMAd and bone-lining cells. Bone formation markers pro-collagen-Iα ($r$ = 0.90) and ALP ($r$ = 0.76) were correlated with secreted glycerol levels.

**Conclusions:** These findings suggest that BMAT plays a role in hand OA through increased lipolysis contributing to bone remodeling and potentially regulating OA progression. BMAd properties in non-weight-bearing finger joints appear to be regulated by BMI, offering a novel hypothesis for the heightened risk of hand OA in obesity.

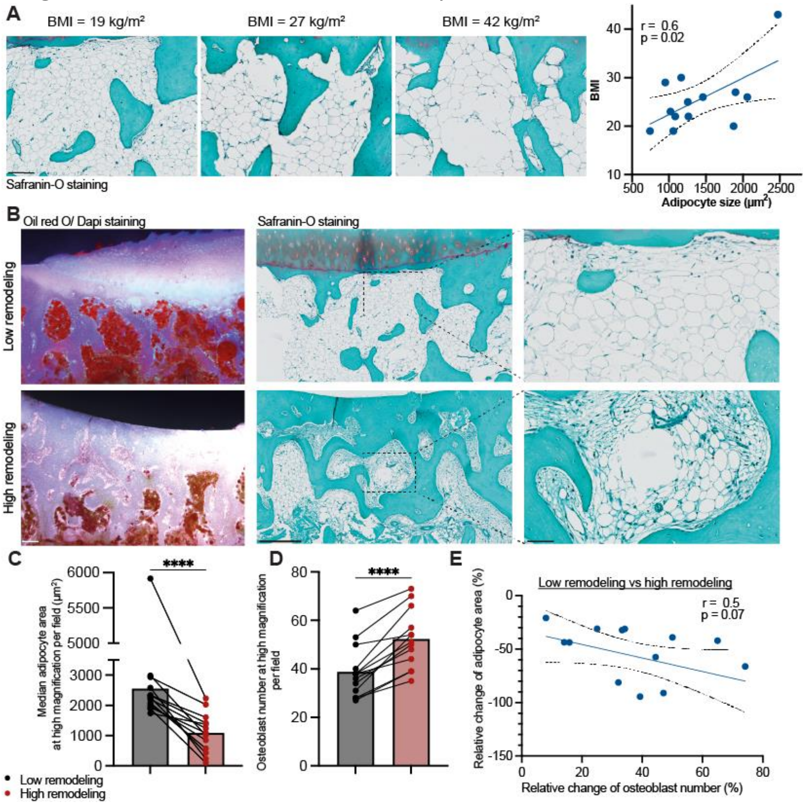

### COP34

### Genetic modulation of WNT1 expression alters subchondral bone remodeling and osteophyte formation during post-traumatic osteoarthritis in mice

*__Weixin Xie__[1], Antonia Donat[1], Shan Jiang[1], Ruben Augustin[1], Jan Sevecke[1], Thorsten Schinke[2], Timur Alexander Yorgan[2], Anke Baranowsky[1], Johannes Keller[1]*
*[1]University Medical Center Hamburg-Eppendorf, Department of Trauma and Orthopedic Surgery, Hamburg, Germany ; [2]University Medical Center Hamburg-Eppendorf, Department of Osteology and Biomechanics, Hamburg, Germany*

**Abstract Text**

**Background:** WNT1 mutations in humans have been shown to cause osteogenesis imperfecta (OI) or early onset osteoporosis, establishing an essential role for WNT1 as a key regulator of skeletal integrity. In addition, WNT1 has been implicated in the pathogenesis of osteoarthritis, although a causal relationship has not been established.

**Objective:** To evaluate the effect of WNT1 on the progression of post-traumatic osteoarthritis of the knee (PTOA) in mice with inactivation, overexpression, or a patient-relevant mutation of WNT1.

**Methods:** Twelve-week-old female mice underwent anterior cruciate ligament transection (ACLT) of the right knee to induce PTOA. Bilateral knee joints were harvested 4 weeks after surgery. Osteoblast-specific WNT1 knockout (Runx2-Cre;Wnt1$^{fl/fl}$) mice and mice carrying the homozygous OI-causing WNT1-G177C mutation (G177C) were used. In addition, an osteoblast-specific inducible WNT1 transgenic (Col1a1$^{tTA}$/pTet$^{Wnt1}$) mouse model was employed, in which Wnt1 overexpression was induced in the first 2 weeks (Early-Tg) or last 2 weeks (Late-Tg) after ACLT. Littermate controls were used for each mouse strain. Subchondral bone microstructure and osteophyte volume were quantified by micro-CT scanning.

**Results:** Compared to littermate controls, Runx2-Cre;Wnt1$^{fl/fl}$ mice showed a similar degree of subchondral trabecular bone loss in OA knees relative to the contralateral knees. Early-Tg and, unexpectedly, G177C mice showed protection against ACLT-induced subchondral bone loss. Late-Tg mice exhibited a sclerotic bone phenotype with higher subchondral bone mass in OA knees than in contralateral knees. Osteophyte formation in OA knees was significantly increased in Late-Tg mice, whereas there was no difference in Early-Tg, Runx2-Cre;Wnt1$^{fl/fl}$ and G177C mice compared to the respective controls.

**Conclusions:** Late-phase osteoblast-specific WNT1 overexpression promotes subchondral bone sclerosis and exacerbates osteophyte formation in mice with PTOA, whereas early-phase WNT1 overexpression and the G177C mutation protect subchondral bone from ACLT-induced bone loss. However, osteoblasts appear not to be the pathophysiological relevant source of WNT1.

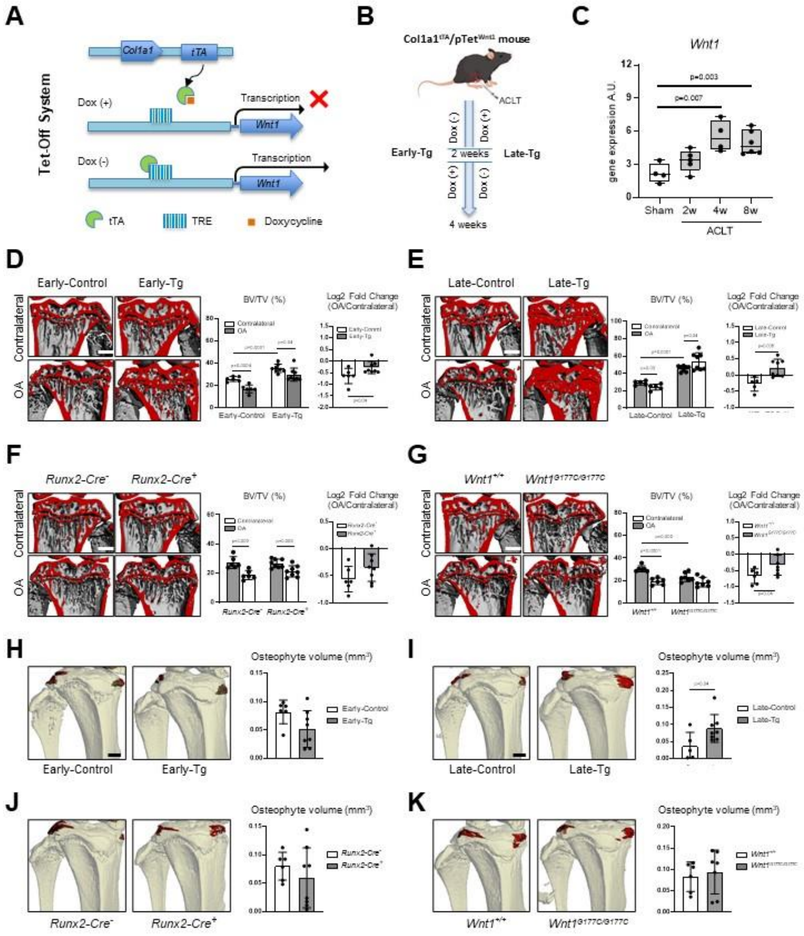

**Figure 1. Modulation of WNT1 expression alters subchondral bone remodeling and osteophyte formation after ACLT. A)** Schematic representation of the Tet-off system used to induce *Wnt1* expression in osteoblastogenic lineage by *Col1a1-tTA*. **B)** Schematic diagram showing the time points of doxycycline removal in Col1a1$^{tTA}$/pTet$^{Wnt1}$ mice after ACLT for the induction of Wnt1 expression in the early and late phases. **C)** *Wnt1* gene expression in knee joints of WT mice receiving sham or ACLT surgery at indicated time points. **D-G)** Representative µCT images of the tibial subchondral bone in mice of the indicated genotypes 4 weeks after ACLT and quantification of bone volume/tissue volume (BV/TV) with comparison of fold change (OA knee to contralateral knee). Scale bar = 500µm. **H-K)** Representative µCT images of the tibial osteophyte formation in mice of the indicated genotypes 4 weeks after ACLT and quantification of osteophyte volume. Osteophytes are shown in red. Scale bar = 500µm. The data are expressed as means ± SD, n = 5-8 per group as indicated. One-way or two-way ANOVA followed by Tukey's post-hoc test and two-tailed Student's t-test were used for data analysis. All experiments were approved by the local animal care committee.

**COP35**

### Inactivation of Cxcl9 protects from cartilage deterioration and osteophyte formation in experimental osteoarthritis

*Antonia Donat[1], Weixin Xie[1], Shan Jiang[1], Paul Knapstein[1], Lilly-Charlotte Albertsen[1], Anke Baranowsky[1], Johannes Keller[1]*
*[1]University Medical Center Hamburg-Eppendorf, Department of Trauma and Orthopedic Surgery, Hamburg, Germany*

### Abstract Text

**Background:** Patients with osteoarthritis (OA) suffer from chronic pain, immobility, and reduced quality of life. As there is no cure, joint replacement surgery is often the only treatment option. Previous studies have shown that the chemokine CXC ligand 9 (CXCL9) plays a critical role in angiogenesis and bone metabolism. However, it remains unclear whether CXCL9 influences the progression and outcome of OA.

**Objectives:** This study was designed to evaluate the effects of genetic ablation of *Cxcl9* in a standardized OA mouse model.

**Methods:** Female WT and *Cxcl9*-KO mice with a C57BL/6J genetic background received a unilateral anterior cruciate ligament dissection (ACLT) at the age of 12-14 weeks. To evaluate the progression of OA over time, analysis of the operated and contralateral knees was carried out 4 and 8 weeks postoperatively. Cartilage degeneration and synovial inflammation were evaluated histologically. Osteophyte formation and subchondral bone parameters were analyzed using µCT. Furthermore, gene expression analysis of specific bone and inflammatory parameters was assessed via qRT-PCR.

**Results:** In early stages of OA, cartilage degeneration and synovitis scores did not differ between WT and *Cxcl9*-KO mice. After 8 weeks however, histological evaluation of the OARSI score, synovitis and cartilage degeneration indicated diminished progression of OA in *Cxcl9*-KO mice. While morphometric analysis using µCT revealed no difference in subchondral bone loss in the femur of *Cxcl9*-KO mice compared to WT mice, the formation of osteophytes was reduced in *Cxcl9*-KO mice in early and late-stage OA. This was accompanied by an increase in the cartilage markers *Acan* and *Col2a1* on a transcriptional level, whereas inflammatory markers were decreased by tendency.

**Conclusion:** Our results suggest a protective effect of CXCL9 against cartilage degradation and joint inflammation. Therefore, CXCL9 must be considered as an important mediator in the progression of OA and could potentially represent a target for new treatment options.

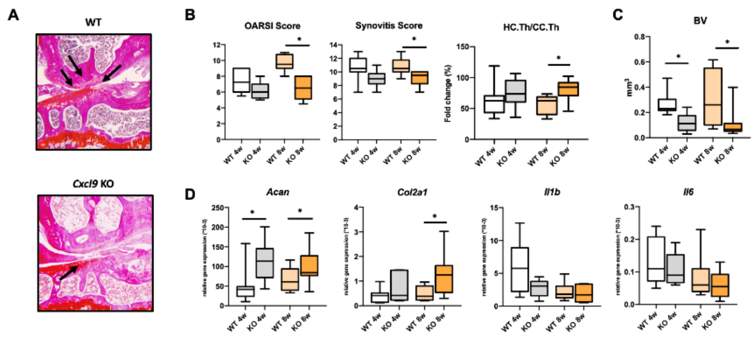

**Figure 1.** Attenuation of OA progression in mice lacking *Cxcl9*. **A)** Representative H&E-stained sections of the medial knee compartment 8 weeks after ACLT operation. Black arrows indicate the observed cartilage erosions. **B)** OARSI score, synovitis score and cartilage destruction at the indicated time points. Values are presented as mean values derived by evaluation of all four quadrants. HC.Th/CC.Th = hyaline cartilage thickness / calcified cartilage thickness. **B)** Micro-computed tomographic evaluation of osteophytes at the indicated timepoints. BV = bone volume. **C)** Expression of chondrocyte and inflammatory markers in early and late-stage OA. *Acan* = Aggrecan. *Col2a1* = Collagen type II alpha 1 chain. *Il1b* = Interleukin-1 beta. *Il6* = Interleukin-6. For all experiments, n = 6-8 mice per group. Two-tailed Student's t-test were performed for data analysis. All experiments were approved by the local animal care committee.

### COP36

### Chondrocyte specific inactivation of Piezo1 attenuates osteoarthritis development

*Assil-Ramin Alimy[1], Laura J. Brylka[2], Shan Jiang[1], Tobias Malte Ballhause[1], Anke Baranowsky[1], Simon Von Kroge[1,2], Frank Timo Beil[1], Michael Amling[2], Johannes Keller[1], Thorsten Schinke[2], Timur A. Yorgan[2], Tim Rolvien[1]*
*[1]University Medical Center Hamburg-Eppendorf, Department of Trauma and Orthopaedic Surgery, Hamburg, Germany ; [2]University Medical Center Hamburg-Eppendorf, Department of Osteology and Biomechanics, Hamburg, Germany*

### Abstract Text

**Background:** Piezo proteins are mechanically activated ion channels required for mechanosensing functions in various cell types. While it was previously shown that Piezo1 expression in osteoblast lineage cells is crucial for bone formation, there is limited evidence regarding a potential role of Piezo1 and/or Piezo2 in physiological and pathological endochondral ossification processes. The latter includes, for example, the formation of osteophytes during the development of osteoarthritis (OA), the most common joint disorder worldwide.

**Purpose:** We aimed to address the question if chondrocyte expression of the mechanosensitive Piezo proteins affects OA development, focusing on osteophyte formation.

**Methods:** We generated mice with chondrocyte-specific deletion of Piezo1 and Piezo2 for in-depth characterization of their osteochondral phenotype compared to control mice. Anterior cruciate ligament transection (ACLT) was performed in 12-week-old mice on the right side whereas the left knee served as non-operated control. The induced OA phenotype was analyzed 8 weeks after surgery by micro-CT and histomorphometry. Immunohistochemistry was applied on osteophyte specimens to assess the expression of Piezo1.

**Results:** Mice with chondrocyte-specific inactivation of Piezo1, but not Piezo2, exhibited a reduction in articular cartilage degeneration, as indicated by lower OARSI scores post-ACLT (control: 7.3 vs $Piezo1^{Col2a1cre}$: 3.7, p=0.03; vs $Piezo2^{Col2a1cre}$: 8.6, p=0.56). Importantly, chondrocyte-specific inactivation of Piezo1 also resulted in decreased osteophyte volume (Figure 1A, B) and maturation (Figure 1C, D). In line with these findings, we observed high Piezo1 expression in human osteophyte specimens, particularly in zones of endochondral ossification, compared to osseus, fibrotic, and cartilaginous areas (Figure 1 E, F).

**Conclusion:** Our data establish Piezo1 as a crucial regulator in the development of OA, prominently in osteophyte formation and maturation. Consequently, our findings also imply that Piezo1 antagonists could potentially be established as innovative disease-modifying osteoarthritis drugs.

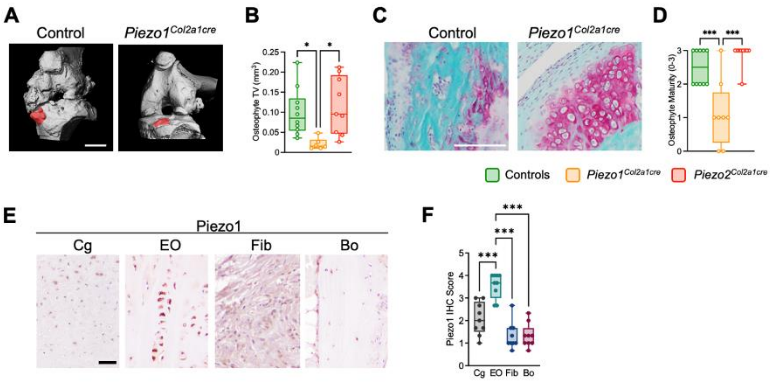

### Poster Sessions

### Poster Sessions: Biomarkers and biochemical testing

### P001

### Bone turnover biomarkers predict one-year all-cause mortality and walking ability in geriatric hip fracture patients

_Guangtao Fu_[1]
[1]*Guangdong Provincial Peoples' Hospital, Orthopedics, Guangzhou, China*

### Abstract Text

**Purpose:** To investigate the utility of serum C-terminal cross-linking telopeptides (β-CTX) and procollagen type I N propeptide (PINP) for predicting one-year mortality and walking ability in Chinese geriatric hip fracture patients who underwent surgical interventions.

**Method:** Elderly patients (≥ 60 years) who underwent surgical interventions for unilateral low-energy hip fractures from 2015 to 2020 in our center were included. Demographic data was retrospectively retrieved from the electronic medical database. The PINP and β-CTX concentrations were measured before the surgery. The patients were divided into two groups according to the outcome of mortality and walking ability after hip surgery, respectively. β-CTX and PINP were divided into four grades based on quartiles [Quartile(Q)1–4] for further analysis. All the variables with $p < 0.1$ in univariable analysis were included in a multivariable model.

**Results:** In univariable analysis, the levels of serum β-CTX (p =0.007) and PINP (p =0.025) were associated with one-year mortality, while the association between levels of serum β-CTX (p =0.072) or PINP (p =0.055) with one-year disability was marginally significant. After adjustment for confounders, the relative risk [OR (95 % CI), Q4 v sQ1, p-value] of one-year mortality and one-year disability were 7.28 (2.08–29.78, p =0.003) and 3.97 (1.44–11.69, p =0.009) for β-CTX and 5.87 (1.70–23.80, p =0.008) and 3.48 (1.30–9.93, p =0.016) for PINP, respectively. The coefficient of determination, AUC and bias-corrected C-index of predictive models based on previously reported predictors were significantly improved after integrating β-CTX or PINP.

**Conclusion:** Higher serum β-CTX and PINP are independently associated with an increased risk of one-year mortality and disability in patients with hip fractures. The application of BTMs improves the performance of currently available predictive models.

**P002**

**Arctiin suppresses ferroptosis and apoptosis of chondrocyte induced by accumulated iron via AKT/NRF2/HO-1 signaling pathway**

*Junzheng Yang*[1]*, He Qi*[1]*, Chen Baihao*[1]*, Chen Peng*[2]
[1]*Guangzhou University of Chinese Medicine, First school of clinical medicine, Guangzhou, China ;* [2]*The First Affiliated Hospital- Guangzhou University of Chinese Medicine, Department of Orthopaedics, Guangzhou, China*

**Abstract Text**

**Background:** With the development of the aging society, more and more knee osteoarthritis (KOA) patients will be diagnosed. Iron overload (IO) was considered to be a risk factor for cartilage degradation in KOA advancement. However, few drugs were found to improve cartilage degeneration by alleviating multiple cell death induced by the impaired iron level of the knee joints.

**Purpose:** We aimed to elucidate that Arctiin (ARC), a natural compound extracted from Fructus Arctii, plays a role in managing KOA caused by accumulated iron levels by restoring chondrocyte apoptosis and ferroptosis.

**Methods:** Single-cell RNA sequencing (scRNA-seq) analysis was used to discover the disparities in chondrocytes between KOA patients and non-KOA people. CCK-8 assay was performed to detect chondrocyte viability. Annexin V-FITC/PI staining determined the cell apoptosis rate. The fluorescence density reflected the iron content, reactive oxygen species (ROS), lipid-ROS, and mitochondrial membrane potential. RT-qPCR and Western Blotting (WB) were used to detect the expression levels of genes and proteins expression. Micro-CT and Safranin O-Fast Green staining were used to detect the phenotype of the knee joints.

**Results:** ARC can increase cell viability and inhibit chondrocyte apoptosis. Further, ARC acts as an anti-ferroptosis effect by reducing the intracellular iron, ROS, and lipid-ROS content and restoring mitochondrial damage. Based on the results of scRNA-seq, we found that ARC can play a role by activating AKT/NRF2/HO-1 signaling pathway. In vivo, ARC can significantly improve the severity of KOA caused by IO.

**Conclusion:** We indicated that ARC alleviates apoptosis and ferroptosis in chondrocytes via the AKT/NRF2/HO-1 signaling pathway which provides ARC a promising role in treating KOA.

**P003**

**The Polymorphism of rs6086746 may be Susceptible to Osteoporosis: from Inference of Bioinformatics to Validation in Case-Control Study**

*Yen Chun Chou*[1], *Sui-Lung Su*[1]
[1]*National Defense Medical Center, School of Public Health, Taipei, Taiwan R.O.C.*

**Abstract Text**

**Background/Introduction:** Osteoporosis (OP) possesses notable prevalence in postmenopausal women. Genome-wide association studies (GWAS) have revealed a multitude of single nucleotide polymorphisms (SNPs) linked to the risk of osteoporosis. However, the linkage of most SNPs to OP from GWAS remains controversial.

**Purpose:** We aimed to develop a method integrated GWAS and chromatin immunoprecipitation sequencing (ChIP-seq) to infer OP-related SNPs and to confirm our discovery in case-control study.

**Methods:** We integrated whole genome sequencing about SNPs from the Taiwan Biobank database and ChIP-seq from JASPAR database to infer OP-related SNPs. A case-control study was used to validate the inference from bioinformatics with 107 OP patients and 254 controls recruited at Tri-Service General Hospital between 2015 and 2019. OP diagnosis adhered to the World Health Organization (WHO) criteria, where a T score of bone mineral density (BMD) ≤ -2.5 indicated osteoporosis, while a T score of BMD ≥ -1 signified normal bone density. Statistical assessment involved the application of Chi-square tests and logistic regression.

**Results:** A novel SNP, rs6086746, within the PLCB4 promoter was discovered to exhibit an association with OP in Chinese populations. Individuals carrying the AA allele demonstrated a significantly elevated risk of OP compared to those with GG or AG genotypes (adjusted odds ratio [OR] = 6.89; 95% confidence intervals: 2.23–21.31, p = 0.001) in our case-control study.

**Conclusions:** Our novel method, integrated conventional GWAS and ChIP-seq, and a case-control study prove that rs6086746 may be susceptible to OP. However, functional analysis is needed to further confirm the causal relationship between rs6086746 and OP.

**P004**

**IGF-1 rs35767 is susceptible to osteoporosis in Asian postmenopausal females**

*Yi Yun Chen*[1], *Sui-Lung Su*[1]
[1]*National Defense Medical Center, School of Public Health, Taipei, Taiwan R.O.C.*

**Abstract Text**

**Background:** Most postmenopausal women suffer from Osteoporosis (OP). However, the issue about the relationship between IGF-1 polymorphisms and OP is pending, particularly rs35767.

**Purpose:** To investigate whether the *IGF-1* rs35767 were associated with OP in postmenopausal females.

**Methods:** We used meta-analysis which related studies derived from PubMed, Embase, Cochrane, and Web of science up until November 2020 and case-control study which recruited 95 OP cases and 222 healthy controls between March 2015 and July 2019. OP was diagnosed based on WHO criteria for diagnosis of OP as T score of bone mineral density (BMD) $\leq -2.5$; normal, as T score of BMD $\geq -1$. *IGF-1* SNPs were genotyped by iPLEX Gold SNP genotyping. Our case-control study was incorporated into a meta-analysis with criteria of significance using odds ratios (ORs) with corresponding 95% confidence intervals (CI) to evaluate risk factor of SNPs on OP. TSA was used to estimate the sample sizes required to achieve a conclusion.

**Results:** In our case-control study, we found nonsignificant association of rs35767 [Adj-OR: 0.95 (95% CI: 0.56-1.60)] with OP in postmenopausal females. However, integration of our case-control study and 3 published studies, rs35767 [OR: 1.24 (95% CI: 1.05-1.47)] showed a conclusively risk association with OP in postmenopausal females judged by TSA with 2267 Asians.

**Conclusions:** This study concludes that IGF-1 rs35767 is susceptible to osteoporosis in Asian postmenopausal females.

**P005**

**Diabetes and bones: Unraveling the Biomarker Symphony and Density Dance**

*Nicklas Rasmussen*[1], *Annika Vestergren Kvist*[2], *Simon Lykkeboe*[3], *Joop Van den Bergh*[4], *Peter Vestergaard*[5]
[1]*Aalborg University Hospital- Denmark, Steno Diabetes Center North Denmark, Aalborg, Denmark ;* [2]*Aalborg University Hospital- Denmark, Steno Diabetes Center North, Aalborg, Denmark ;* [3]*Aalborg University Hospital, Department of clinical Biochemistry, Aalborg, Denmark ;* [4]*School for Nutrition and Translational Research in Metabolism NUTRIM- Maastricht University- Maastricht-, Department of Internal Medicine- Division of Rheumatology- Maastricht University Medical Center+- Maastricht- The Netherlands- Department of Inter, ;* [5]*Aalborg University Hospital and Steno Diabetes Center North, Endocrinology and clinical Institute, Aalborg, Denmark*

**Abstract Text**

**Introduction:**
Individuals with diabetes face an increased susceptibility to fractures; however, the pathogenesis of this phenomenon remains poorly understood at a biochemical level.

**Purpose:** Therefore, this study delved into essential bone biomarkers in T1D and T2D, investigating their connections with areal bone mineral density (aBMD) and volumetric BMD (vBMD).

**Methods:** In a cross-sectional study, 543 adults participated, including 111 with T1D, 106 with T2D, and 328 controls. Employing DXA and HR-pQCT scans, a range of biochemical bone biomarkers (CTX, P1NP, sclerostin, osteopontin, OCN, and IGF-1) were analyzed. Group comparisons and regression models, adjusting for relevant factors, were executed. Correlation analyses, utilizing Spearman with Bonferroni correction, explored associations between bone parameters and biomarkers for intergroup comparison.

**Results:** Results revealed significantly reduced CTX1, P1NP, IGF-1, OCN, and osteopontin in T1D and T2D ($p<0.05$), with T2D having the lowest ($p=0.01$). Conversely, sclerostin was higher in both T1D and T2D than controls ($p=0.001$). T2D showed elevated neck aBMD (0.85g/cm2, $p=0.011$) and tibial vBMD (300.6HA/cm3, $p=0.004$) in contrast to T1D and controls. In T2D, neck aBMD correlated negatively with CTX1 (r=-0.332,p=0.001), P1NP (r=-0.278, p=0.005), OCN (r=-0.397, p=0.001), and positively with sclerostin (r=0.229, p=0.041). In T1D, CTX1 correlated negatively, while IGF-1 correlated positively with neck aBMD (r=-0.301,p=0.002, r=0.329,p=0.001, resp). Controls exhibited positive correlations of IGF-1 (r=0.321,p=0.001) and negative correlations of sclerostin (r=-0.151,p=0.008) with neck aBMD. Tibial vBMD was negatively correlated with P1NP in T2D (r=-0.231,p=0.024) and positively correlated with IGF-1 in T1D and controls (p<0.011). Sclerostin strongly correlated with diabetes duration in both T1D and T2D (r=0.491,p=0.001, r=0.342, p=0.003, respectively). IGF-1 had a negative correlation in T1D (p=0.001).

**Conclusion:** Individuals with T1D and T2D present distinctive bone biomarker profiles and associations with aBMD and vBMD, emphasizing the intricate interplay between diabetes and bone health.

**P006**

**Correlation of bone turnover markers, vitamin D levels and steroidal hormones with bone density in healthy males**

*Panagiotis Kokkoris*[1]
[1]*HELLENIC AIR FORCE GENERAL HOSPITAL, ENDOCRINOLOGY, Athens, Greece*

**Abstract Text**

**Purpose:** The aim of the study is to find any possible factors which may influence bone density in otherwise healthy males.

**Methods:** We examined 300 men. They all had a DXA scan in the lumbar spine and the femoral neck. Additionally we measured the following: Bone turnover markers (osteocalcin, alkaline phosphatase and P1NP for bone formation, and CTX for bone resorption), 25(OH) Vitamin D levels, steroidal hormones (testosterone and estradiol), and calcium and phosphorus levels. Finally we examined if there is any correlation between all the above measurements with the bone density.

**Results:** The mean age of the population studied was 33,9±7,7 years, mean weight 84,1±10,8 kg, and mean body mass index 26,1±4,2 kg/m². The mean bone mineral density was 1,26±0,15 g/cm² for the lumbar spine and 1,08±0,14 g/cm² for the femoral neck. The mean values for the markers that were described above were as following: Osteocalcin 18,3±7 ng/ml, alkaline phosphatase 63±17,2 IU/l, P1NP 51,7±25 ng/ml, CTX 0,36±0,18 ng/ml, 25(OH) Vitamin D 21,7±9,2 ng/ml, testosterone 5,5±2,3 ng/ml, estradiol 43,6±17,3 pg/ml, calcium 9,5±0,6 mg/dl and phosphorus 3,2±0,5 mg/dl. As for the correlations we found the following: Positive correlation between bone mineral density (BMD) and osteocalcin, P1NP and CTX respectively (p<0,05), negative correlation between BMD and alkaline phosphatase (p<0,05). We didn’t find any correlation between BMD and 25(OH) Vitamin, testosterone, estradiol, calcium and phosphorus. Additionally mineral density (BMD) (p<0,05), and negative correlation between BMI and 25(OH) Vitamin and testosterone, while we found no correlation between BMI and estradiol.

**Conclusion:** Our study showed that in young men, bone turnover markers (apart from the alkaline phosphatase) are positively correlated with bone mineral density, while steroidal hormones, Vitamin D and calcium and phosphorus are not correlated with BMD. Additionally, body weight is positively correlated with BMD and negatively correlated with Vitamin D and testosterone.

**P007**

**Bone turnover markers and their correlations with parameters of bone quality from HRpQCT measurements of the femoral head**

*Ines Foessl*[1], *Uldis Berzins*[2], *Gloria Hohenberger*[2], *Barbara Obermayer-Pietsch*[1]
[1]*Medical University of Graz, Department of Internal Medicine- Division of Endocrinology and Diabetology, Graz, Austria ;* [2]*Medical University of Graz, Department of Orthopaedics and Trauma, Graz, Austria*

**Abstract Text**

Bone density and quality decrease with age, eventually enabling low impact fractures. Femoral neck fractures are among the most threatened in the elderly, because they often result in morbidity or immobility as well as early mortality. Laboratory characteristics are easily accessible bone turnover markers (BTM). In this study, we analysed bone turnover markers for their correlation with bone density and trabecular parameters of the femoral head by measuring surgery explants with high-resolution quantitative CT (HRpQCT) in patients after acute fractures.

41 patients (30 women and 11 men) were included in this study. Blood was drawn preoperatively within 12 hours after administration to the emergency room and analysed for a BTM-panel. Femoral heads were collected during surgery, and a calotte of the femoral head (164 slices) was scanned by XtremeCTII (Scanco, Brüttisellen, CH) and evaluated for bone trabecular morphometry. Values of men and women were compared with t-test, correlations were calculated with Spearman correlation (GraphPad Prism 8). This study was conducted under the premises of the Declaration of Helsinki (Ethics Committee approval 28-003 ex 15/16).

Mean age of the participants was 82 years. Male femoral head calottes were larger (p=0,009) with higher bone volume (p=0,021) than in females, with no differences in any other measured laboratory characteristic or bone trabecular morphometry between males and females in our cohort. Interestingly, osteoprotegerin correlated with trabecular number (r=0,5854, p=0,0053), trabecular spacing (r=0,7154, p=0,0133) and the connectivity density (r=-0,7198, p=0,0125) in women but not in men. Other than that, BTMs did not correlate with trabecular parameters.

Our results showed no correlation between BTM and HRpQCT-based parameters of femoral head explants. While this skeletal site might undergo specific geometrical and strain conditions not being representative for other bone sites, the connection between osteoprotegerin and trabecular integrity highlights a relationship, which might be explained by further investigations.

**P008**

**Calcium Isotope Ratio in Patients with Mongenic Bone Diseases – a prospective, cross sectional, single center Study**

*Robert Munzinger*[1], *Felix Von Brackel*[1], *Florian Barvencik*[1], *Ralf Oheim*[1], *Michael Amling*[1]
[1]*University Medical Center Hamburg-Eppendorf, Institute for Osteology and Biomechanics, Hamburg, Germany*

**Abstract Text**

In the field of genetic bone diseases, malformations or mineralization deficits do often restrict the reliability of bone density for diagnosis. Therefore, a comprehensive assessment of serum and urine parameters plays a major role. The proportion of calcium isotopes $^{44}Ca$ and $^{42}Ca$ and their kinetic fractionation is topic to research since the 1970s. The premise posits that lighter isotopes ($^{42}Ca$) play a more prominent role in metabolism, hence $\delta^{44/42}Ca$ is higher for bone formation and lower for resorption phases. The perception exists, that the $\delta^{44/42}Ca$ concentration in blood and urine serves as a reflective indicator of bone metabolism and thus bone density.

We have employed elaborated clinical characterization by means of full laboratory assessment, bone densitometry, high resolution quantitative computed tomography and isotope analysis to test for the hypothesis.

We included 40 subjects with genetically caused bone diseases, such as early-onset osteoporosis (7), hypophosphatasia (12), osteogenesis imperfecta (12) and X-linked hypophosphatemia (9) and controls (17). Regression analyses revealed a significant correlation of $\delta^{44/42}Ca$ with ca/creatinine ($\delta^{44/42}Ca_{serum}$: $r^2$=0.5727, p<0.; $\delta^{44/42}Ca_{urine}$: $r^2$=0.4226, p<0.001). Furthermore, bone densitometric parameters were significantly correlated with $\delta^{44/42}Ca$ (BMC: serum $\delta^{44/42}Ca$ $r^2$=0.4029, p<0.001; urine $\delta^{44/42}Ca$ $r^2$=0.6063, p<0.001). However, XLH became apparent to solely drive the correlations with higher bone density than the rest of the cohort. Correlations significantly dropped after the exclusion of XLH (max. $r^2$=0.29).

Our results suggest that $\delta^{44/42}Ca$ is mainly linked to calcium excretion in patients with genetic bone diseases. Furthermore, increased osteoid volume in XLH patients and thus increased calcium need, may result in higher $\delta^{44/42}Ca$. Given weak correlations of bone mineral density and $\delta^{44/42}Ca$ upon exclusion of XLH, $\delta^{44/42}Ca$ does not provide valuable information regarding bone density in genetic patients.

**ND-P001**

**Clinical characterization of iron transfusion induced hypophosphatemic osteomalacia in 13 patients including recovery data**

*Felix Nikolai Schmidt[1], Jonathan Grambeck[1], Florian Barvencik[1], Michael Amling[1], Ralf Oheim[1]*
*[1]University Medical Center Hamburg-Eppendorf, Department of Osteology and Biomechanics, Hamburg, Germany*

**Abstract Text**

**Background/Introduction:** Iron deficiency is treated by iron supplementation. Intravenous iron infusion is the treatment of choice. It is now thought that the carbohydrate components of iron infusions cause hypophosphatemia and hence osteomalacia due to excess iFGF23. Patients present with fractures and low bone density, which is often misinterpreted as osteoporosis in clinical practice.

**Purpose:** We performed a detailed characterisation of patients with iron infusion-induced osteomalacia.

**Methods:** Laboratory assessment, densitometry, muscle strength testing and HR-pQCT imaging were used to characterise and monitor the treatment effect by stopping the infusions. In addition, we describe a clinical case in which the infusions had to be continued and were therefore treated with burosumab (FGF23 antibody).

**Results:** All 13 patients received ferric carboxymaltose (FCM) infusions and had low blood phosphate levels. A rapid normalisation of phosphate levels (pre: 0.50 mmol/l ± 0.23 mmol/l, post: 0.93 mmol/l ± 0.32 mmol/l, $p<0.001$) was observed after stopping the FCM infusion. In addition, bone density increased significantly (pre: -1.85 ± 1.84, post: -0.91 ± 2.13, $p<0.05$) and muscle strength increased significantly over the course of therapy, with pain scores decreasing from 7.31 ± 1.22 to 2.71 ± 1.27 ($p<0.0001$). Therapy included discontinuation of FCM infusions, temporary phosphate and calcium substitution, and supplemental vitamin D and active vitamin D substitution. One patient requiring continuous FCM infusion showed significant improvement with supportive therapy and additional benefit with burosumab treatment.

**Conclusions:** Our study highlights the importance of monitoring phosphate during iron infusion and to distinguish iron infusion induced osteomalacia from osteoporosis. Withdrawal of iron infusions is effective in counteracting iFGF23-induced phosphate wasting. Supplementation allows a rapid recovery of the musculoskeletal system and improves the pain level of the patients.

**ND-P002**

**Vitamin D and K measurements in postmenopausal women with different bone mineral density status**

*<u>Sieglinde Zelzer</u>[1], Meinitzer Andreas[1], Enko Dietmar[2], Makris Konstantinos[3], Tournis Symeon[4], Trironidi Ioulia[5], Spanou Loukia[5], Alonso Nerea[1], Cavalier Etienne[6], Herrmann Markus[1]*
*[1]Clinical Institute of Medical and Chemical Laboratory Diagnostics, Medical University of Graz, Graz, Austria ; [2]Clinical Institute of Medical and Chemical Laboratory Diagnostics, Medical University of Graz, 'Graz, Austria ; [3]Director of Clinical Biochemistry Department, KAT General Hospital, Athens, Greece ; [4]Laboratory for Research of the Musculoskeletal System "Th. > Garofalidis"- Medical School, University of Athens, Athens, Greece ; [5]Clinical Biochemistry Department, KAT General Hospital, Athens, Greece ; [6]Department of Clinical Chemistry, University of Liege, Liege, Belgium*

**Abstract Text**

**Objectives:** Both vitamins, namely D and K, are known to promote bone metabolism. This study aimed at measuring several metabolites of these vitamns by liquid chromatography tandem mass spectrometry (LC-MS/MS) in a cohort of postmenopausal women with normal and low bone mineral density (BMD).

**Methods:** Overall, 131 postmenopausal women were included for vitamin metabolite determination (serum 25-hydroxyvitamin D (25[OH]D), 24,25-dihydroxyvitamin D (24,25[OH]$_2$D), Phylloquinone (K1), menaquinone-4 (MK-4) and MK-7determination) in this study. The vitamin D metabolite ratio (VMR) was calculated: VMR (%) = (24,25[OH]$_2$D/25[OH]D)*100. Additionally, parathyroid hormone (PTH), type I procollagen-N-terminal-Peptide (PINP) and C-terminal telopeptides of type I collagen (CTX) were performed with immunoassay.

**Results:** The mean age of 68 women with normal BMD and 63 women with low BMD was 58.2 ± 8.5 years. Median (interquartile range) for 25(OH)D and total vitamin K concentrations were 53.5 (39.6 – 65.9) nmol/L and 1.33 (0.99 – 2.39) nmol/L, respectively. All vitamin metabolites were comparable in both BMD groups. Furthermore, BMD was comparable in participants with adequate and inadequate vitamin status (at least one criterion was met: 25(OH)D < 50 nmol/L, 24,25(OH)$_2$D < 3 nmol/L, VMR < 4 %, total vitamin K < 0.90 nmol/L). PTH, but not PINP or CTX, was inversely correlated with 25(OH)D, 24,25(OH)2D and VMR.

**Conclusions:** In postmenopausal women vitamin D and K measurements were not related to BMD. An inverse association was observed between vitamin D metabolites and PTH.

**ND-P003**

**Correlation between chronological age and P16INK4a expression in circulating T-cells as biomarker of human senescent cell burden.**

*Shakespeare Jeromdesella*[1], *Moustapha Kassem*[1]
[1]*Clinical institute- university of southern Denmark, Department of Endocrinology- Odense University Hospital- Denmark, Odense, Denmark*

**Abstract Text**

**Background:** Primary osteoporosis is considered a condition occurring as response to loss of bone mass due to aging. One of the hallmarks of aging is the accumulation of cellular senescence. Studies in murine models showed that reducing cellular senescence increased bone mineral density and bone quality.

**Purpose:** This pilot study aimed to measure the marker of cellular senescence, p16ink4a in CD3+ lymphocytes, in young (age 20-35) and older (age 65-90) individuals, to confirm age-dependent expression in a Danish cohort. Furthermore, in 5 individuals we compared the expression of p16ink4a in blood and bone marrow to evaluate any difference in the compartments.

**Methods:** Peripheral whole blood was collected in EDTA tubes from 85 individuals. Using magnetic-activated cell sorting CD3+ lymphocytes were isolated and lysed. RNA was isolated for measurement of p16ink4a expression by real-time qPCR. Bone marrow aspirates from 5 individuals were collected in EDTA tubes and followed the same isolation and measurement protocol as for the whole blood.

**Results:** The calculated Pearson correlation coefficient showed a strong positive linear correlation ($r=0.7113$) for chronological age and expression of p16ink4a in CD3+ lymphocytes with a p-value $< 0.0001$. In the bone marrow, we observed a higher expression of p16ink4a than in blood. Results of the paired t-test indicated that there is a non-significant small difference between the expression of p16ink4a in the blood ($M = 2.1$, $SD = 2.4$) and bone marrow ($M = 2.8$, $SD = 3.4$), $t(9) = 1$, $p = .354$.

**Conclusion:** The expression of p16ink4s indicating the burden of cellular senescence, is associated with chronological aging. This marker could be used to evaluate the effect of treatment targeting cellular senescence.

### Poster Sessions: Bone bioengineering, regeneration and implants

**P009**

**Chemical composition determines the resorbability of ceramics after a prolonged follow-up period in the rabbit spinal fusion model**

*Nikola Štoković[1], Natalia Ivanjko[1], Marko Pecin[2], Ana Smajlovic[2], Hrvoje Capak[3], Zoran Vrbanac[3], Drazen Vnuk[2], Drazen Maticic[2], Slobodan Vukicevic[1]*
*[1]University of Zagreb School of Medicine, Laboratory for Mineralized Tissues, Zagreb, Croatia ; [2]University of Zagreb Faculty of Veterinary Medicine, Clinics for Surgery- Orthopedics and Ophthalmology, Zagreb, Croatia ; [3]University of Zagreb Faculty of Veterinary Medicine, Department of Radiology- Ultrasound Diagnostics and Physical Therapy, Zagreb, Croatia*

**Abstract Text**

**Background/Introduction:** Osteogrow-C is a novel autologous bone graft substitute comprised of recombinant human Bone Morphogenetic Protein 6 (rhBMP6) delivered within autologous blood coagulum with the addition of calcium phosphate (CaP) synthetic ceramic particles. CaP particles are available as tricalcium-phosphate (TCP), hydroxyapatite (HA) as well as biphasic CaP containing TCP and HA in different ratios.

**Purpose:** The objective of this study was to evaluate Osteogrow-C with CaP particles in a broad range of different chemical compositions in the rabbit posterolateral spinal fusion model.

**Methods:** rhBMP6 was added to autologous blood and mixed with CaP particles (74-420µm) of four different chemical compositions (TCP, TCP(80%)/HA(20%), HA(80%)/TCP(20%) and HA). Following blood coagulation, Osteogrow-C devices were implanted between L6-L7 lumbar transverse processes in twelve New Zealand White Rabbits. At the end of the follow-up period of one year spinal fusion was evaluated using microCT and histological analyses as well as biomechanical testing employing a three-point bending test. Animal studies were approved by the National Ethics Committee.

**Results:** MicroCT analyses revealed that Osteogrow-C implants with different ceramic particles induced spinal fusion between transverse processes that was maintained after a prolonged, one-year follow-up period. Furthermore, histological analyses revealed that newly formed bone was completely osseointegrated with adjacent transverse processes. Both microCT and histological analyses showed that the chemical composition of the ceramics determined the amount of residual ceramics: TCP was completely resorbed, TCP(80%)/HA(20%) partially while HA and HA(80%/TCP(20%) were almost completely unresorbed. Finally, biomechanical testing (three-point bending test) confirmed that fusion in all experimental groups was biomechanically competent.

**Conclusions:** Osteogrow-C is a novel therapeutic solution in regenerative medicine of bone while the chemical composition of ceramic particles enables tailoring resorbability and amount of residual ceramics incorporated in bone.

**P010**

### rhBMP6 delivered in autologous blood coagulum with addition of ceramics promotes healing of segmental defect in rabbits

*Natalia Ivanjko[1], Nikola Štoković[1], Marko Pećin[2], Ana Smajlović[2], Hrvoje Capak[3], Zoran Vrbanac[3], Ana Javor[3], Dražen Matičić[2], Slobodan Vukičević[1]*
*[1]University of Zagreb School of Medicine, Laboratory for Mineralized Tissues, Zagreb, Croatia ; [2]University of Zagreb Faculty of Veterinary Medicine, Clinics for Surgery- Orthopedics and Ophthalmology, Zagreb, Croatia ; [3]University of Zagreb Faculty of Veterinary Medicine, Department of Radiology- Ultrasound Diagnostics and Physical Therapy, Zagreb, Croatia*

**Abstract Text**

**Purpose/Objective:** Segmental defects of the long bones are among the most complicated conditions in clinical medicine. This study aimed to evaluate Osteogrow-C, a novel osteoinductive device containing recombinant human Bone Morphogenetic Protein 6 (rhBMP6) delivered in autologous blood coagulum (ABC) with synthetic ceramic particles in rabbit ulnar segmental defect model.

**Material and Methods:** Segmental defect (15 mm) was created in the rabbit ulna and filled with Osteogrow-C containing tricalcium-phosphate (TCP; n=5) or biphasic (BCP; composed of 40% TCP and 60% hydroxyapatite-HA; n=5) 500-1700 µm granulate ceramics as compression resistant matrix (CRM). ABC containing TCP or BCP ceramics without rhBMP6 (n=2) were used as a control group. Segmental defect healing was followed by *in vivo* x-ray images (4 and 8 weeks after surgery) and analyzed post-mortem employing micro CT and histological analyses (17 weeks after surgery).

**Results:** Both formulations of Osteogrow-C implants achieved rebridgement of segmental defect 8 weeks following surgery as observed by in vivo x-ray images. Moreover, ex vivo microCT analyses confirmed these findings and revealed that TCP particles were significantly more resorbed than BCP particles. Furthermore, bone formation and defect rebridgement by Osteogrow-C implants was confirmed on histological analyses. On the other hand, ABC with ceramic particles without rhBMP6 did not promote the rebridgement of the bone defect.

**Conclusions:** Different formulations of Osteogrow-C implants achieved rebridgement of ulnar segmental defect in rabbits suggesting that Osteogrow-C might be a therapeutic solution for this complex clinical condition.

**P011**

**PLGA-PEG-COOH Microparticles Covalent Functionalization of MEW PCL 3D Printed mimetic Periosteum Autografts**

*Juan Antonio Romero Torrecilla*[1], *Miguel Echanove-González de Anleo*[1], *Tania López-Martínez*[1], *Purificación Ripalda-Cemboráin*[1], *María José Blanco-Prieto*[2], *Felipe Prósper*[3], *Elisa Garbayo*[2], *Emma Muiños-López*[1], *Froilán Granero-Moltó*[1]
[1]*Clínica Universidad de Navarra, Cell Therapy/Orthopedic Surgery and Traumatology, Pamplona, Spain ;* [2]*Universidad de Navarra, Department of Pharmaceutical Technology and Chemistry, Pamplona, Spain ;* [3]*Clínica Universidad de Navarra, Department of Hematology, Pamplona, Spain*

**Abstract Text**

**Background and Purpose:** Despite bone unique regenerative capacity, fracture healing may be drastically delayed or even impeded, leading to fracture non-union. Autograft treatment as the gold standard offers limited amount of tissue whereas, the use of rhBMP-2 needs to be optimized to avoid undesirable adverse effects derived from supraphysiological dosing. Our aim is to present cutting-edge tissue engineering strategies that combine key factors for bone regeneration.

**Methods:** We manufactured PCL mimetic periosteum scaffolds by 3D melt electrowriting printing that serve as backbone for further functionalization with microparticles (MPs) encapsulating rhBMP-2. MPs containing rhBMP-2 were synthesized by double emulsion. Next, PCL scaffolds underwent aminolysis and PLGA-PEG-COOH MPs were covalently bond to the membrane. We assessed the therapeutic potential of these constructs using critical size femoral defects in rats. Regenerated bone was determined by micro computed tomography (mCT).

**Results:** MPs presented and average size of 10 microns with encapsulation efficacy of 65 % and kinetic release profile for rhBMP-2 reaching plateau at day 1. Amidation reaction assisted by NHS/EDC was optimized up to a 68.8 % yield. Grading doses of rhBMP-2 (200 to 1200 ng rhBMP-2) were encapsulated and their therapeutic potential for bone regeneration assessed in a critical size femoral defect in SD rats. Results showed that functionalization with 600 and 1200 ng presented significantly higher regenerated tissue than nontreated CTL (** p < 0.01 and **** p < 0.0001 respectively) and even than lower doses of 200 and 400 ng rhBMP-2 (**** p < 0.0001) (Fig. 1).

**Conclusions:** Here we present a novel and extremely versatile approach for functionaliing PCL scaffolds using tunable PLGA MPs. Covalent functionalization ensures in situ drug release. This system can be used to encapsulate a wide range of molecules and showed ability to preserve therapeutic potential after lyophilization, facilitating clinical translation and personalized medicine.

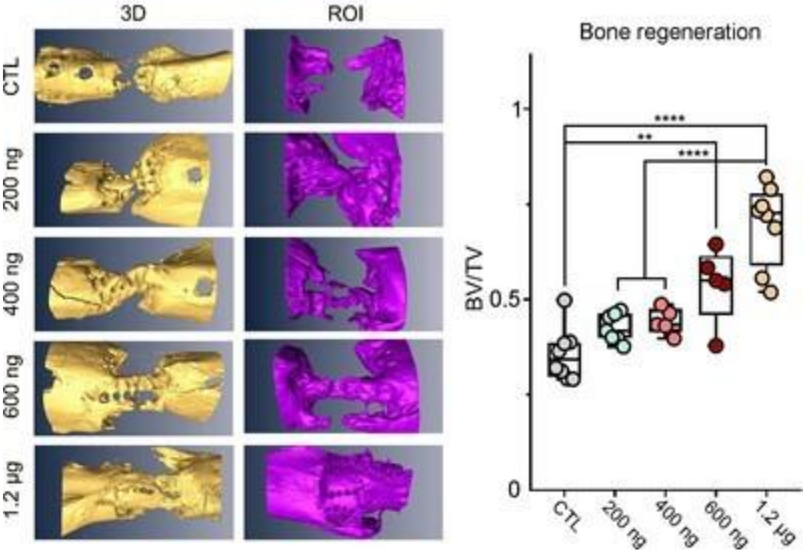

**P012**

**Research on the mechanism of Black phosphorus quantum dots promoting osteogenic differentiation of Human apidose-derived stem cells**

*Renxian Wang[1], Xueshan Bai[2], Dingding Wang[3], Wenkai Wu[3], Anyi Guo[3], Hui Qi[3], Quanzhong Ren[3], Songxia Xia[2], Liya Yang[2], Li Teng[2], Dafu Chen[4], Xieyuan Jiang[3], Yajun Liu[3]*

*[1]JST Sarcopenia Research Centre- National Center for Orthopaedics, Beijing Research Institute of Traumatology and Orthopaedics- Beijing Jishuitan Hospital- Capital Medical University, Beijing, China ; [2]Plastic Surgery Hospital- Chinese Academy of Medical Sciences & Peking Union Medical College, Cranio-Maxillo-Facial Surgery Department, Beijing, China ; [3]Beijing Research Institute of Traumatology and Orthopaedics- Beijing Jishuitan Hospital- Capital Medical University, JST Sarcopenia Research Centre- National Center for Orthopaedics, Beijing, China ; [4]Beijing Research Institute of Traumatology and Orthopaedics- Beijing Jishuitan Hospital- Capital Medical University, Laboratory of Bone Tissue Engineering- Beijing Laboratory of Biomedical Materials- National Center for Orthopaedics, Beijing, China*

**Abstract Text**

**Background:** There are increasing reports on promoting osteogenic differentiation by BP quantum dots (BPQDs). Nanomaterials can promote osteogenic differentiation of mesenchymal stem cells through epigenetic regulation, but the specific regulatory mechanism is not yet clear.

**Purpose:** To investigate whether BPQDs have epigenetic bio-effects on the osteogenic differentiation of hADSCs.

**Method:** Compared with wild type hADSCs, we pre-cocultured hADSCs with BPQDs for osteogenic induction. First, we applied the ALP staining, Alizarin red staining and the sub-renal capsule transplantation model to testify the promoting bio-effects of BPQDs in vitro and in vivo. Next, we performed RNA-seq, direct-DIA Phosphoproteome, Histone modification proteome, and ATAC-seq analysis to explore the epigenetic mechanism of BPQDs' regulation of hADSCs' differentiation fate in depth.

**Results:** We found that the osteogenic capacity of hADSCs was significantly enhanced even without the subsequent addition of BPQDs. We found that a 3-day pre-coculture of BPQDs facilitates the expression of osteogenic genes in hADSCs at 4 and 7 days and the formation of calcium nodules at 14 days. Also, the sub-renal capsule transplantation model showed that the pre-coculture of BPQDs enhanced the osteogenic potential of hADSCs in vivo. Compared with the WT group, hADSCs pre-cultured with BPQDs were sequenced to activate osteogenesis-related genes earlier during subsequent osteogenic induction. Molecular mechanistically, BPQDs affected the chromatin accessibility in hADSCs. The histone modification analysis revealed that BPQDs up-regulated the expression of epigenetically activated marker histones. Moreover, co-analysis of RNA-seq, proteomic, and ATAC-seq reveals that BPQDs can enhance the stemness of hADSCs by up-regulating nuclear skeletal proteins, Histone centromeric protein, and DNA topoisomerases.

**Conclusion:** Thus, when ADSCs are exposed to osteogenesis-inducing signals, they can respond to the stimuli faster and open the chromatin structure related to osteogenic genes. Collectively, these results indicate that BPQDs modulate hADSCs osteogenic differentiation at the epigenetic regulatory level.

**P013**

**Evaluation of the applicative potential of biopolymers produced by microalgae in bone regeneration**

*Arnaud Dulom[1], Céline Laroche[2], Didier Lecerf[3], Pierre Weiss[1], Olivier Gonçalves[4], Valérie Trichet[1]*
*[1]UMR_S 1229 RMeS, Nantes Université- Ecole Nationale Vétérinaire de l'Alimentation et de l'Agroalimentaire- Nantes Atlantique - ONIRIS- route de Gachet- CS 40706- 44307 Nantes Cedex 03- France- CHU d'Angers- Université d'Angers- INSERM, Nantes, France ; [2]UMR 6602 Institut Pascal, Centre National de la Recherche Scientifique- Université Clermont Auvergne- Institut national polytechnique Clermont Auvergne, Clermont-Ferrand, France ; [3]UMR 6270 Polymères Biopolymères Surfaces PBS, Université de Rouen Normandie- Institut national des sciences appliquées Rouen Normandie- Institut Normand de Chimie Moléculaire Médicinale et Macromoléculaire- Institut de Chimie - CNRS Chimie- Centre Nati, ; [4]UMR 6144 Génie des Procédés Environnement - Agroalimentaire GEPEA, Ecole Nationale Vétérinaire- Agroalimentaire et de l'alimentation Nantes-Atlantique- Centre National de la Recherche Scientifique- IMT Atlantique- Nantes université - UFR des Sciences e,*

**Abstract Text**

There is growing interest in the use of marine biopolymers as biomaterials, carriers, delivery systems or bioactive compounds for bone regeneration. According to the strain, macromolecules excreted by microalgae have a wide range of physicochemical and biological properties that may be similar to certain components of skeletal tissue. The aim of this research was to evaluate the potential and safety of biopolymers derived from microalgae in bone healing.

Immune response to an implanted material plays a critical role in all clinical application. For this reason, an identification of immunomodulatory properties has been performed by a quantification of pro-inflammatory cytokines produced by human monocytes treated with our molecular extracts. Analysis indicated a pro-inflammatory profile of the biopolymer related to lipopolysaccharides (LPS) quantified in this one. Then, LPS were totally removed following an alkaline treatment and molecules were characterized by size exclusion and ions exchange chromatography. Results showed that LPS-depleted molecular extracts lost their pro-inflammatory capacity. However, the alkaline treatment induced a denaturation of the macromolecules, with a loss of 2-fold both initial molecular weight and sulphated compounds. Then, we tested osteogenic properties of the different extracts by measuring alkaline phosphatase (ALP) activity during the differentiation of mesenchymal stem cells into osteoblasts secreting bone matrix. Results showed that untreated extracts promoted osteoblastic differentiation through a significant increase in ALP activity, 2-fold higher than LPS control. However, LPS-depleted extracts lost osteogenic potential due to structural modification that lead to a loss of interaction with the differentiation factor BMP-2 measured by surface plasmon resonance. This work has shown that microalgal biopolymers may have osteogenic potential, but GRAM-negative bacterial contaminants present in microalgal culture, must be absent for any clinical application. Ideally, bacterial contamination should be avoided during microalgal growth, as alkaline treatment used to remove LPS induces macromolecule size and sulphation reduction.

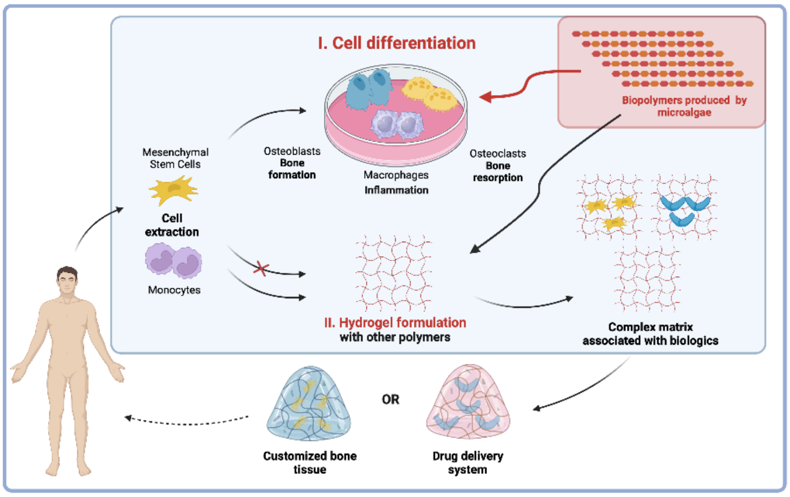

**P014**

**Allografts promote skeletal regeneration of periprosthetic femoral bone loss**

*Simon Von Kroge[1,2], Constantin Schmidt[1], Sebastian Butscheidt[1], Frank Timo Beil[1], Thorsten Gehrke[3], Klaus Püschel[4], Michael Amling[2], Michael Hahn[2], Tim Rolvien[1]*
*[1]University Medical Center Hamburg-Eppendorf, Department of Trauma and Orthopaedic Surgery-Division of Orthopaedics, Hamburg, Germany ; [2]University Medical Center Hamburg-Eppendorf, Department of Osteology and Biomechanics, Hamburg, Germany ; [3]Helios ENDO-Klinik, Department of Joint Surgery, Hamburg, Germany ; [4]University Medical Center Hamburg-Eppendorf, Department of Forensic Medicine, Hamburg, Germany*

**Abstract Text**

**Background:** Periprosthetic bone loss is a common clinical issue that needs to be addressed during revision arthroplasty. Although bone allografts represent the standard among the substitute materials used, evidence of their regenerative potential at the microstructural, cellular, and compositional level is lacking.

**Purpose:** Aim of this study was to evaluate the determinants of allograft incorporation in the femur. For this purpose, a multiscale imaging analysis of human specimens obtained postmortem and subjected to varying follow-up periods (*i.e.,* time in situ) after successful revision surgery was performed.

**Methods:** We analyzed the allograft-host bone interface in seven human femoral proximal diaphyses, applying an experimental imaging setup comprising digital contact radiography, histological analysis, cellular histomorphometry, scanning electron microscopy, and osteocyte lacunocanalicular network characterization.

**Results**: The degree of skeletal regeneration through allograft incorporation between the host bone and allograft bone (i.e., overlap) was higher compared to analyzed specimens obtained from the acetabulum (4.0±2.9 mm *vs*. 2.2±1.0 mm, $p$=0.037). While the overlap was found to be strongly associated with the defect depth ($R^2$=0.94, $p$<0.001), no association between the time in situ and incorporation was observed. The overlap region presented with a higher bone volume fraction (BV/TV) compared to the host bone and showed accumulation of osteoid (OV/BV, 9.8±15.8 % *vs.* 0.4±0.8 %, $p$=0.019) compared to allograft bone. Furthermore, the number of osteoblasts per bone perimeter in the overlap region (15.5±6.2 $mm^{-1}$) was not different compared to host bone (6.8±5.1 $mm^{-1}$, $p$=0.280) and elevated compared to allograft bone (0.1±0.2 $mm^{-1}$, $p$<0.001). While bone cement limited the incorporation process, we further demonstrated that the osteocytic canalicular system of host bone reached the allograft interface without penetrating allograft bone.

**Conclusion:** Our results provide the first systematic histological evaluation of bone allografts used during revision surgery for femoral bone loss, showing that they adequately facilitate skeletal regeneration through osteoconduction and remodeling.

**P015**

**A murine sub-renal capsule transplatation model: a novel approach to evalaute the osteogenic potency of stem cells and biomaterials**

*Xueshan Bai*[1]*, Renxian Wang*[2]
[1]*Plastic Surgery Hospital- Chinese Academy of Medical Sciences & Peking Union Medical College, Cranio-Maxillo-Facial Surgery Department, Beijing, China ;* [2]*JST Sarcopenia Research Centre- National Center for Orthopaedics, Beijing Research Institute of Traumatology and Orthopaedics- Beijing Jishuitan Hospital- Capital Medical University, Beijing, China*

**Abstract Text**

**Background:** Human adipose-derived stem cells (hADSCs) have great potential as important seed cells for applications in bone regenerative medicine. Moreover, bone tissue engineering research focuses on efficiently and stably evaluating the osteogenic potential of hADSCs in vivo and their interaction with biomaterials. Now, the in situ osteogenic animal models have the disadvantages of severe trauma and a long postoperative observation period. In contrast, the traditional subcutaneous allogeneic osteogenesis has the shortcomings of being unstable and prone to non-specific calcified nodules. Thus, we provide a protocol of a murine sub-renal transplantation model for osteogenic potential evaluation of hADSCs and biomaterials.

**Methods:** We performed experiments on 48 BALB/c mice. Among them, 24 mice underwent sub-renal capsule transplantation surgery with different cell count of hADSCs and different concentration of BMP-2. The other 24 mice were injected subcutaneously with same subgrouped. The mice were observed for 4-8 weeks, recording their body weight, subcutaneous nodule size in the subcutaneous group, and wound healing in the sub-renal capsule group. After 4-8 weeks of sampling, gross photographic measurements, H&E staining, Movat staining, IF staining were performed to evaluate and calculate the modeling success rate.

**Results:** After 4 weeks, ossicles could be found in the sub-renal capsule(5/6), while nodes rarely formed subcutaneously (1/6). H&E staining, Movat staining, and IF staining confirms ectopic bone-forming. At 8 weeks,  the success rate of modeling in sub-renal capsule group is higher than subcutaneous group, especially in low concentration of BMP group. No mice died during the observation period of 8 weeks, and the weight loss of both groups was not statistically significant.

**Conclusions:** We describe a novel and efficient murine sub-renal capsule transplantation model to investigate the osteogenic potential of hADSCs and biomaterials. This mini-invasive model allows for a shorter postoperative observation period with more stable than traditional subcutaneous ectopic osteogenesis.

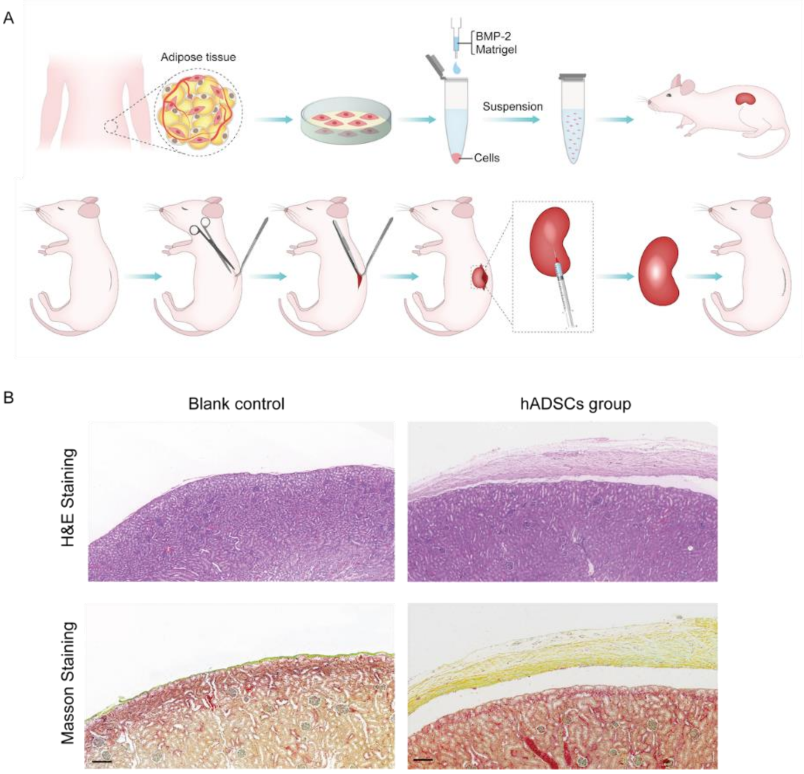

**P016**

**Osteogenic potential of canine periodontal ligament-derived cells: Unveiling insights for scientific advancements**

*<u>Laura Pinho</u>[1,2,3,4,5], André Queirós[6,7], Catarina Ferreira dos Santos[2,8], Bruno Colaço[3,5,7,9], Maria Helena Fernandes[3,4]*
*[1]University of Trás-os-Montes e Alto Douro, Veterinary Sciences, Vila Real, Portugal ; [2]Politechnic Institute of Setúbal, EST Setúbal - CDP2T, Setúbal, Portugal ; [3]University of Porto, Requimte/LAQV, Porto, Portugal ; [4]Faculty of Dental Medicine of University of Porto, BoneLab - Laboratory for Bone Metabolism and Regeneration, Porto, Portugal ; [5]University of Trás-os-Montes e Alto Douro, CITAB - Centre for the Research and Technology of Agro-Environmental and Biological Sciences, Vila Real, Portugal ; [6]SCIVET, Breed, Paredes, Portugal ; [7]University of Trás-os-Montes e Alto Douro, CECAV -*

*Animal and Veterinary Research Centre UTAD, Vila Real, Portugal ; [8]Higher Technical Institute, Centre for Structural Chemistry, Lisbon, Portugal ; [9]University of Trás-os-Montes e Alto Douro, AL4AnimalS - Associate Laboratory for Animal and Veterinary Sciences, Vila Real, Portugal*

**Abstract Text**

**Background/Introduction:** Periodontal disease is highly prevalent in companion animals, with dogs experiencing approximately 80% prevalence by the age of 2 years. The substantial incidence underscores the importance of addressing periodontitis in veterinary and dental care. The periodontal tissue is an intricate connective tissue comprised of various cell types that collaborate to uphold the integrity of all structures within the periodontium. Periodontal ligament (PDL)-derived cells are widely used in dental research and can be easily isolated and differentiated into various cell types, including osteogenic cells. This characteristic adds significant value, making them ideal for *in vitro* studies focused on understanding periodontal tissue.

**Purpose:** Develop a suitable *in vitro* model for canine periodontal studies.

**Methods:** PDL was isolated from freshly extracted teeth from dog maxilla in a routine procedure, and explants were cultured in standard conditions. PDL-derived cells were cultured for up to 21 days in basal or osteogenic medium and characterized for cell viability/proliferation, alkaline phosphatase (ALP) activity, Collagen, ALP and Von Kossa histochemical staining and Osteopontin (SPP1) immunostaining. Comparison of experimental conditions was assessed using the t-test and one-way analysis of variance (ANOVA), followed by the post-hoc Tukey.

**Results**: In both conditions, cell viability/proliferation and ALP activity increased throughout the culture time. Of note, PDL-derived cells were sensitive to the culture conditions and osteogenic-induced cultures exhibited a cell layer organized in cellular aggregates that stained intensely for collagen and ALP, distinct from the homogeneous cell layer observed in basal conditions. Furthermore, the presence of SPP1 protein and deposition of calcium phosphate were observed in osteogenic cultures.

**Conclusion(s)**: Osteogenic induction of canine PDL-derived cells suggests its potential to be used as an *in vitro* cell model for periodontal studies expecting to bring awareness to reduce animal use.

**P017**

**Persistent mineralisation defects in the medial malleolus after resorption of Mg implants for fracture fixation: lateral comparison with the unfractured side**

*Ines Foessl[1], Viktor Labmayr[2], Annelie Martina Weinberg[2], Patrick Holweg[2], Barbara Obermayer-Pietsch[1]*
*[1]Medical University of Graz, Department of Internal Medicine- Division of Endocrinology and Diabetology, Graz, Austria ; [2]Medical University of Graz, Department of Orthopaedics and Trauma, Graz, Austria*

**Abstract Text**

**Background:** Bioresorbable implants ought to provide stability for fracture healing and resorb thereafter, rendering implant removal obsolete. We follow up on our long-term results of the first in human study in 5 patients with medial malleolus fractures treated with bioresorbable magnesium screws.

**Purpose**: Comparison of the implantation site with the corresponding contralateral part as well as implant resorption and assessment of the implantation site of 9 implants in 5 patients with HRpQCT (high resolution peripheral quantitative computed tomography).

**Methods:** Isolated bimalleolar or trimalleolar ankle fractures were surgically treated with bioresorbable screws (Magnesium 99.1%, Calcium 0.45%, Zinc 0.45%). 20 patients were assessed clinically and HRpQCT scans were performed after at least 2.5 years in 5 patients. The implantation site was compared to the contralateral ankle. Evaluations included total volume, bone volume, and volume of the trabeculae-free space.

**Results:** All patients showed clinical evidence of fracture healing already twelve weeks after the surgery without clinical complications, full weight bearing and absence of pain in the fractured ankle. All observed implants were resorbed for the most part after 2.5 years. Persistent mineralization defects at the surgical site were recorded at all implantation sites studied, with apparent secondary enlargements of the drilling channel in patients with low bone mineral density. The trabeculae-free cavities were 596.1 $mm^3$ in mean size (239.2 –1109.1$mm^3$), with a screw volume before implantation of 243.3 $mm^3$.

**Conclusion:** Bioresorbable magnesium screws for fracture fixation provide satisfactory fracture healing and clinical outcome after one year. Surgical removal is not necessary. We provide long-term results in paired patient investigations. Especially in patients with decreased bone mineral density, the dynamic of implant resorption needs to be studied further.

**P018**

**Insights into surface topography: Coupled osteogenesis and osteoclastogenesis on metallic bone implants**

*Sanjana Vig[1], Lamborghini Sotelo[2], Maria Helena Fernandes[3], Silke Christiansen[4]*
*[1]University of Porto, Faculty of Dental Medicine, Porto, Portugal ; [2]Friedrich–Alexander University Erlangen–Nürnberg- Staudtstraße 7- 91058 Erlangen- Germany, Friedrich–Alexander University Erlangen–Nürnberg- Staudtstraße 7- 91058 Erlangen- Germany, Forchheim, Germany ; [3]Universidade do Porto- Rua Dr. Manuel Pereira da Silva- Porto- 4200-393 Portugal, Faculdade de Medicina Dentaria-, Porto, Portugal ; [4]Institute for Nanotechnology and Correlative Microscopy, Institute for Nanotechnology and Correlative Microscopy eV INAM- Äußere Nürnberger Str. 62- 91301 Forchheim-Germany, Forchheim, Germany*

**Abstract Text**

**Background:** Human bone undergoes a constant remodeling cycle involving bone resorption by osteoclasts followed by bone formation by osteoblasts. Successful osseointegration of orthopedic implants at bone-implant site requires a balance between the bone formation and resorption processes. While several studies on prospective bone grafts focus on bone formation potential, there is a dearth of studies evaluating simultaneous osteoclastogenic and osteogenic activity of the implant post transplantation. Metals like Titanium alloys, Titanium and stainless steel have been popular choice of bone substitutes due to their excellent mechanical properties and cytocompatibility.

**Purpose:** This study aims to evaluate the influence of surface topography, specifically through laser modifications and polishing, in regulating osteogenic and osteoclastic differentiation over metallic bone substitutes. This will contribute towards delineating optimal surface properties for successful osseointegration of implants.

**Method**: We established in vitro human osteoclasts and osteoblastcultures over polished and unpolished surfaces of laser modified metallic plates. Cultured osteoblasts and osteoclasts were characterized for proliferation/metabolic activity, tartrate-resistant acid phosphatase (TRAP)/ alkaline phosphatase (ALP) activity, morphology (SEM/confocal microscopy) and specific gene expression. Osteoblasts were further subjected to EDX analysis and Raman spectroscopy.

**Results:** Laser modified surfaces demonstrated increased ALP activity of osteoblasts. SEM/EDX and Raman analysis further revealed that laser modified surfaces showed significantly enhanced mineralization potential, specifically calcium and phosphate deposition, compared to unlasered surfaces. In contrast, TRAP activity and osteoclastic gene expression was lower on the lasered modified surfaces compared to unlasered materials. These outcomes were corroborated by SEM/confocal analysis. Large and well spread osteoclast-like cells were observed on non-lasered surfaces, undergoing fusion and F-actin ring formation. These interactions were absent on lasered surfaces exhibiting significantly lower surface area and multinucleation.

**Conclusion:** Osteoblasts and osteoclasts are highly responsive to surface topography and roughness. Laser modified metallic surfaces demonstrated enhanced osteogenesis and mineralization while showing lower potential for osteoclastogenesis.

**ND-P004**

**Combining cell therapy and therapeutic mRNAs for the treatment of bone fractures**

*Amelie Frischer*[1]*, Oona Jung*[1]*, Johannes Oesterreicher*[1]*, Magda Tyszkiewicz*[1]*, Madhusudhan Bobbili*[1]*, Heinz Redl*[2]*, Johannes Grillari*[1]
[1]*Ludwig Boltzmann Institute for Traumatology, Tissue Regeneration and Ageing, Vienna, Austria ;* [2]*Ludwig Boltzmann Institute for Traumatology, Senescence and Healing of Wounds, Vienna, Austria*

**Abstract Text**

**Introduction:** Several types of bone fractures require enhanced therapeutic treatment and can be a substantial psychological and physiological burden for the patient. Regarding non-union fractures, common surgical interventions such as autologous bone grafts and allografts are confronted with drawbacks such as restricted supply of donor tissue, donor site morbidity, and rejection of the implant by the host. Consequently, new strategies are necessary to complement conventional therapy.
Combinations of pro-regenerative materials and growth factors can be applied locally to the defect site and are expected to boost the regeneration process. Bone morphogenetic proteins (BMPs) and vascular endothelial growth factor A (VEGF-A) have been reported to act in this direction. More recently, WNT1 has come into focus for its osteo-anabolic activity. Direct growth factor administration is confronted with limitations such as short half-lives and the need for supraphysiological dosages *in vivo*. This can be circumvented by using stromal cells as delivery vehicles which produce the therapeutic proteins on site. The inherent pro-regenerative features of stromal cells represent an additional bonus of cell therapy approaches.

**Aim:** Here we use human adipose-derived stromal cells (ASC/TERT300) transfected with therapeutic *in vitro* transcribed (IVT) mRNA coding for human BMP-2, VEGF-A, and WNT1 to improve the regenerative processes underlying bone formation *in vitro* and *in vivo*.

**Methods:** The therapeutic factors are synthesized as IVT mRNA. ASC/TERT300 are transfected using Lipofection. Expression of the factors is monitored via ELISA and Western Blot. Functionality is confirmed via induction of HUVEC-sprouting, canonical WNT-pathway activation and ALP activity, respectively.

**Results and conclusion:** Expression of the therapeutic factors produced by ASC/TERT300 was successfully confirmed *in vitro*. As a next step, transfected cells will be tested in an ectopic bone model.

**ND-P005**

**Monitoring cell fate in vitro**

*<u>Donata Iandolo</u>[1,2], Suraj Pavagada[3], Sofia Herrero Barros[4], Fu Ying[5,6], Henry Lu[6], Ciro Menale[7], Laurence Vico[8], Róisín M. Owens[6]*
*[1]Inserm, SAINBIOSE U1059- INSERM- Université Jean Monnet- Mines Saint-Etienne Campus Santé - 10- rue de la Marandière- F-42270, Saint-Priest-en-Jarez, France ; [2]University of Cambridge, Department of Chemical Engineering and Biotechnology-, Cambridge, United Kingdom ; [3]University of Cambridge, Department of Chemical Engineering and Biotechnology, University of Cambridge, United Kingdom ; [4]University of Glasgow, Department of Biomedical Engineering, Glasgow, United Kingdom ; [5]University of Strathclyde, Department of Chemistry, Glasgow, United Kingdom ; [6]University of Cambridge, Department of Chemical Engineering and Biotechnology, Cambridge, United Kingdom ; [7]University of Naples Federico II, Department of Clinical Medicine and Surgery, Naples, Italy ; [8]INSERM, Sainbiose, Saint-Priest-en-Jarez, France*

**Abstract Text**

How can we monitor cells in vitro? The last years have seen an increase in the number of techniques to monitor cells. Microphysiological systems do benefit from the possibility of integrating devices to monitor cell fate continuously. Our work focuses on the development of tools to monitor stem cell fate using both bioelectronic devices in vitro with a focus on bone physiology and tissue engineering. Osteoporosis is a skeletal disease characterized by bone loss and bone microarchitectural deterioration. The increasing life expectancy calls for innovative and effective approaches to compensate for bone loss [1]. Osteoporosis was reported to affect 32 million individuals aged 50+ across Europe in 2019 [2]. 4.28 million so-called osteoporotic fractures were reported in 2019, and they are projected to increase to 5.34 million by 2034. Due to their well-documented regenerative and anti-inflammatory potential, stem cells represent a promising option. We investigate the effect of physical stimuli (e.g., shear stress, electrical/electromagnetic stimulation) on stem cells affecting their differentiation into bone-forming cells to both understand physiological processes, for future tissue regeneration application and to develop a robust 3D in vitro system. Stem cell differentiation into bone-forming cells is associated with the secretion of several proteins such as collagen type I, osteopontin, osteonectin, osteocalcin and BMP-2. To be able to online monitor stem cell differentiation we selected various biomarkers for the different stages of cell differentiation. Here we present some of the work done targeting both the protein BMP-2 [3] and RANKL. We were able to detect both biomarkers at physiologically relevant levels. Sensor integration within the culture system is the next challenge to effectively achieve continuous stem cell differentiation.

**References**

1. 1. https://www.iofbonehealth.org/global-policy-initiatives
2. *Arch Osteoporos,* 16(1):82. doi: 10.1007/s11657-020-00871-9
3. *Flex. Print. Electron.* 4 044006. doi: 10.1088/2752-5724/ace3dd

**Acknowledgment:** D.I. would like to acknowledge the financial support by ESA contract 4000128599/19/NL/PG, Bonus project.

**ND-P006**

**Analysis of the Multi-Differentiation Induction Potential of Pro-Osteogenic Human Dental Pulp Stem Cell Spheroid's Secretome**

*Daniel Ferreira[1], Icaro Ribeiro[1], Jose Granjeiro[2], Katiucia Paiva[1]*
*[1]University of São Paulo, Anatomy, São Paulo, Brazil ; [2]The National Institute of Metrology-Standardization and Industrial Quality Inmetro, Bioengineering, Xerem, Brazil*

**Abstract Text**

**Background:** It is known that the cell culture models an oxygen tension may modulates the secretome.

**Purpose:** The objective was evaluate the monolayer/spheroid, spheroid size and oxygen tension on DPSCs secretome when cultured under osteogenic medium.

**Methods:** DPSCs were isolated from human adult dental pulps collected from third molars. DPSCs were used at passage 6 to fabricate 500 µm and 1,000 µm spheroids or monolayers. Cells/spheroids were kept in a basic medium/BM or induced by an osteogenic medium/OM. The spheroids/monolayers were cultured for 14 days in an OM (generators) under normoxia (20% O2) or hypoxia (2% O2). Conditioned medium (CM) was produced by fresh serum-free medium for four days, generating basic CM (bnCM or bhCM) or osteogenic CM (onCM or ohCM). Spheroids CM was incubated on undifferentiated monolayers and the CM of the monolayers on spheroids (undifferentiated) for four days (recipients). We analysed the gene expression related to differentiation (osteogenic, endothelial and adipogenic), hypoxia and cytoskeleton.

**Results:** For monolayers that received 1,000 µm/onCM, there was a more significant upregulation of β-actin/HIF-1α genes when compared to controls. In monolayers that received 500 µm/onCM, there was substantial variation only in β-actin, where the group that received bnCM had a decrease, or bhCM had upregulation. In 500 µm spheroids induced by monolayer, the group that received onCM showed an increase of up to 11 times in endothelial markers expression concerning the other 500 µm experimental groups, but osteoblastic genes had downregulated. For the monolayer induced by 1,000 µm/onCM, the main results are drastically reduced genes related to endothelial cells, hypoxic stress, and adipogenic differentiation. Conclusion(s): It shows the increase in multidifferentiation is linked to the 3D conformation/spheroid size/osteogenic stimulus, the larger size being more prone to multidifferentiation.

**Poster Sessions: Bone biomechanics and quality**

**P019**

**How Does Subchondral Bone Density Distribution of the Distal Humerus Change Between Early and Advanced Stages of Osteoarthritis?**

Hyun Sik Gong[1], Ji Sup Hwang[2]
[1]Seoul National University Bundang Hospital, Orthopedic Surgery, Seongnam, Korea- South ; [2]Seoul National University Hospital, Orthopedic Surgery, Seoul, Korea- South

**Abstract Text**

**Background:** The distribution of subchondral bone density (SBD) within a joint represents stress distribution applied to the joint. In the elbow joint, this has not been studied much in the context of osteoarthritis (OA) initiation or progression. We asked whether SBD is different between early and advanced stages of elbow OA, and determined factors associated with the shift in SBD distribution.

**Methods:** We reviewed 126 patients (90 men and 36 women; mean age, 60 years) with elbow OA who underwent plain radiographs and conventional CT scans. We measured OA grade, osteophyte severity, and alignment (carrying angle) in the radiographs. Three-dimensional distal humerus subchondral bone models were derived from the CT images, and we measured the SBD in two different sites: where it articulates with radius (SBDrad), and with ulna (SBDulna). We further defined SBDratio as ratio of SBDrad to SBDulna. Then, we compared the SBD parameters between early and advanced OA and performed multivariate analysis of the factors associated the SBD parameters, including age, sex, osteophyte severity, and carrying angle.

**Results:** There were 42 patients (33%) with early OA and 84 patients (67%) with advanced OA. Compared to early OA, the SBDratio was significantly higher in advanced OA (1.18 vs. 1.09, $p = 0.008$). The large SBDratio, indicative of radial deviation in SBD distribution, was independently associated with a large carrying angle ($\beta = 0.005$, $p = 0.04$) and severe osteophytes at the radiocapitellar joint ($\beta = 0.095$, $p = 0.002$), regardless of age, sex, or osteophyte at the ulnotrochlear joint.

**Conclusions:** Stress concentration was more radially deviated in the advanced stages of elbow OA than in the early stages. Increase in carrying angle was associated with radial deviation of the stress. This study gives some insight into potential pathophysiology of elbow OA in relation to elbow alignment.

**P020**

### Enzymatic and non-enzymatic collagen cross-links and fracture occurrence in type 1 diabetes patients

Eleftherios Paschalis[1], Sonja Gamsjaeger[1], Laura Graeff-Armas[2], Susan Bare[2], Robert Recker[3], Mohammed Akhter[3]
[1]Ludwig Boltzmann Institute for Osteology, Organic Matrix, Vienna, Austria ; [2]University of Nebraska, Medical Center, Omaha, United States ; [3]Creighton University, Osteoporosis Research Center, Omaha, United States

**Abstract Text**

**Background / Introduction:** Increased fracture risk in type 1 diabetes (T1D) patients is not fully captured by bone mineral density (BMD) considerations. Elevated non-enzymatic cross-links (advance glycation endproducts; AGEs) are suspected to be responsible, yet, a recent review has questioned whether this is causative or associative.

**Purpose & Methods:** To test the hypothesis that enzymatic collagen cross-links rather than AGEs correlate with fracture incidence in T1D, we analyzed iliac crest biopsies from age- and sex-matched, fracturing T1D patients (N = 5; T1DFx), 6 nonfracturing T1D patients (T1DNoFx), and 6 healthy subjects (HC), by Raman microspectroscopy (spectra were acquired at: i) osteoid: surface with evident tetracycline labels, 1 µm distance from the mineralizing front, ii) TA1: mid-distance between the second fluorescent label and the mineralizing front, iii) TA2: mid-distance between the two fluorescent labels, iv) TA3: 1 µm behind the first fluorescent label, v) Interstitial bone: geometrical centers in cortical and cancellous compartment, away from fluorescent labels), in intracortical and trabecular bone, to determine pyridinoline (Pyd), ε-N-Carboxymethyl-L-lysine (CML), and pentosidine (PEN)).

**Results:** There were no differences in the clinical characteristics between the T1DFx and T1DNoFx groups. At trabecular forming surfaces, T1DFx patients had higher PEN and Pyd content compared to T1DNoFx ones.

**Discussion & Conclusions:** Previous studies have shown that elevated PEN does not necessarily correlate with fracture incidence in postmenopausal, long-term T1D patients. On the other hand, the elevated Pyd content in the T1DFx patients would be consistent with published studies showing a significant correlation between elevated Pyd collagen cross-links and fracture occurrence independent of BMD. Collagen fibers with high Pyd content are more brittle. Thus, a plausible suggestion is that it is the enzymatic collagen cross-links that either by themselves or in combination with the adverse effects of increased AGEs accumulation, that result in fragility fracture in T1D.

**P021**

**Bone loss occurs at the radius in adult patients with osteogenesis imperfecta**

*<u>Camille Blandin</u>[1], Agnes Ostertag[2], Martine Cohen-Solal[1]*
*[1]Lariboisière hospital, rheumatology, Paris, France ; [2]Lariboisière hospital, inserm U1132, Paris, France*
**Abstract Text**

**Introduction:** Osteogenesis imperfecta (OI) is a rare genetic bone disorder characterized by recurrent fractures. Although low bone density (BMD) has been reported, prospective studies on BMD change overtime in OI are missing.

**Purpose:** We aimed to describe the natural history of BMD in adults with OI in a Reference Center of Rare Bone Diseases.

**Methods:** Among the 106 patients, we included the patients who underwent BMD measurements (DEXA) from 2000 to 2023. We selected those with available BMD at least at one skeletal site (hip, lumbar spine (L1-L4) or radius) and no bone treatment at baseline. Data are analyzed as Z-scores and T-scores at baseline and BMD after a mean follow-up of 5.05 years (3.18–8.75y, 161 visits). Longitudinal BMD change was analyzed in linear mixed effects models.

**Results:** We included 71 patients at the initial visit (44 women including 8 postmenopausals, 27 men) with a median age 41.4±13.7 years. Serum levels of calcium, 25OHD, alkaline phosphatase and CTX were unremarkable. At baseline, Z-score revealed no low BMD at any site except in men who had a lumbar Z-score of -3SD (table). After a mean 5 years follow-up, mixed effect models adjusted for age, gender, BMI and treatment showed no significant bone loss at the lumbar spine (p=0.14), total hip (p=0.30), femoral neck (p=0.12) and UD radius (p=0.68). Bone loss was borderline at the one-third radius (p=0.05).

**Conclusion:** Our cohort showed low lumbar Z-score BMD in men, but not in women with OI. No change in BMD was observed after 5 years of follow-up, except at the radius site. Therefore, a longitudinal monitoring of BMD should include the radius in OI.

**Table I : Baseline BMD measurements**

| Skeletal sites | | Total<br>n=67<br>*mean ± SD* | Women<br>n=43<br>*mean ± SD* | Men<br>n=24<br>*mean ± SD* |
|---|---|---|---|---|
| **Total Hip** | Z-score | -1.2 ± 1.0 | -1.0 ± 1.0 | -1.6 ± 1.0 |
| | T-score | -1.6 ± 1.1 | -1.4 ± 1.1 | -1.9 ± 1.0 |
| **Femoral neck** | Z-score | -1.0 ± 1.0 | -0.9 ± 1.0 | -1.4 ± 1.0 |
| | T-score | -1.5 ± 1.1 | -1.4 ± 1.1 | -1.7 ± 1.1 |
| **Lumbar spine** | Z-score | -2.3 ± 1.5 | -1.9 ± 1.4 | -3.0 ± 1.6 |
| | T-score | -2.7 ± 1.5 | -2.4 ± 1.4 | -3.2 ± 1.6 |
| **One-third Radius** | Z-score | -0.8 ± 1.4 | -0.5 ± 1.4 | -1.2 ± 1.4 |
| | T-score | -0.9 ± 1.4 | -0.5 ± 1.4 | -1.3 ± 1.3 |
| **Ultra-distal Radius** | Z-score | -1.6 ± 2.0 | -1.7 ± 1.9 | -1.5 ± 2.1 |
| | T-score | -1.7 ± 1.9 | -1.7 ± 1.8 | -1.7 ± 2.0 |

**P022**

**Glucagon-like peptide-1 (GLP-1) controls bone strength and material properties of the appendicular skeleton through a central relay**

*Guillaume Mabilleau[1], Jessica Denom[2], Aleksandra Mieczkowska[1], Christophe Magnan[2], Celine Cruciani - Guglielmacci[2]*
*[1]University of Angers, Inserm UMR_S 1229 - REGOS team, Angers, France ; [2]Université Paris Cité, CNRS UMR 8251 BFA- REGLYS team, Paris, France*

**Abstract Text**

**Background/Introduction:** GLP-1, a gut hormone produced by L-cells of the intestinal epithelium, has previously been implicated in controlling bone strength. However, the exact mechanism by which it exerts its activity remains to be elucidated.

**Purpose:** Here we hypothesized that GLP-1 acts in the central nervous system (CNS) to control bone material properties and strength.

**Methods:** Expression of Glp1r was investigated by qPCR and fluorescent in situ hybridization (FISH). A total of 88 female BALB/c mice, sham or ovariectomized, received either the stable GLP-1 analogue exendin-4 (Ex4) through subcutaneous (25 nmoles/kg/day) or intracerebroventricular (icv, 70 pmoles/day) administration, or subcutaneous administration of the blood-to-brain barrier impermeant exendin-4-BSA (25 nmoles/kg/day). Bone strength was evaluated at the femur midshaft by 3-point bending. Cortical bone microstructure was evaluated at the femur mid-diaphysis by microCT with a 4µm isotropic voxel size. Bone material properties were investigated at bone formation site by Fourier transform infrared microspectroscopy. Differences between groups were investigated by one-way ANOVA with Dunnett’s post-hoc comparison and considered significant at $p<0.05$.

**Results:** *Glp1r* expression was undetectable in bone by qPCR or FISH. Subcutaneous administration of Ex4 resulted in a 23% enhancement of energy-to-fracture ($p=0.023$) in the absence of any modifications in cortical bone microstructure. At the bone material level, collagen maturity was significantly improved by 22% (p=0.004). Icv administration of Ex4 led to significant 5% augmentation of energy-to-fracture (p=0.009) in the absence of any modifications in cortical bone microstructure. Here again, collagen maturity was increased by 22% (p<0.001). However, subcutaneous administration of Ex4-BSA, that cannot cross the blood-brain barrier, did not result in ameliorations in neither energy-to-fracture nor collagen maturity. This is in agreement with the absence of GLP1r detection in bone.

**Conclusions:** Taken together, these data suggest that GLP-1 controls bone strength and material properties through a central relay that remains to be elucidated.

**P023**

**A Novel Bone Microstructure Analysis Revealed Critical Regions for Early Stability in Total Hip Arthroplasty**

*Jing Xu*[1], *Changchuan Li*[1], *Yue Ding*[1]
[1]*Sun Yat-Sen Memorial Hospital- Sun Yat-Sen University, Department of Orthopaedic Surgery, Guangzhou, China*

**Abstract Text**

**Background/Introduction:** Periprosthetic bone quality is crucial for arthroplasty implant stability. While numerous studies have discovered treatments to enhance bone quality to prevent implant loosening, the focus has largely been on BMD, with a notable lack of studies addressing periprosthetic bone microstructure.

**Purpose:** This study proposed a novel method for periprosthetic bone microstructure analysis, based on standardized zones and strut analysis (Fig 1A). The purpose was to investigate its performance and superiority in assessing periprosthetic bone microstructure in relation to implant stability

**Methods:** This retrospective study analyzed periprosthetic femur bone microstructure in 91 patients who underwent uncemented total hip arthroplasty. The periprosthetic bone microstructure was denoted by strut parameters. The top 5% of standard zones most associated with implant stability (regarded as implant migration measured by Radiostereometric Analysis) were identified as the most informative regions, and were compared with conventional Gruen zones for predicting 1-year implant migration.

**Results:** The porosity change of periprosthetic bone microstructure was observed during 1-year follow-up (high density area: -0.25±0.25%/month, number of struts: -0.94±1.10/zone·month). A significant correlation was found between the strut-based density and BMD (Pearson's r=0.36-0.56, P≤0.05). The proposed method further revealed a thin bone region adjacent to the implant surface was highly correlated with implant migration (Fig 1B, MSE=0.81, Pearson's r=0.35, p<0.01), and was confirmed as an independent predictor of 1-year migration.

**Conclusion:** The proposed method allowed to access periprosthetic bone microstructure, and enabled a more fine-scale identification of critical bone regions associated with early implant stability.

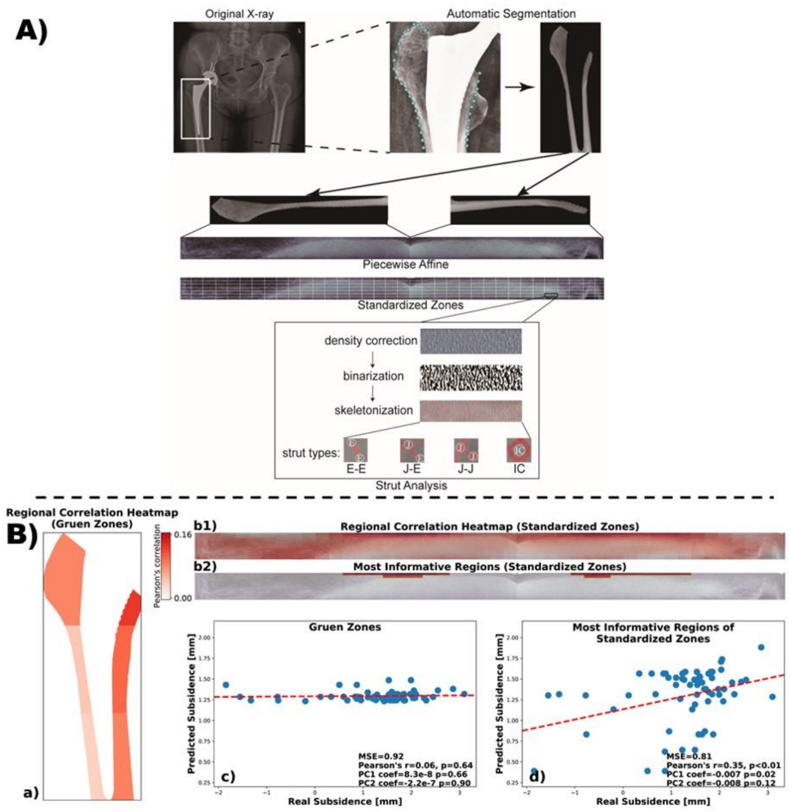

Fig 1: A): Flowchart of standardized zones and strut analysis. B): The correlation between 1-year implant subsidence and strut parameters of each Gruen Zones (B-a), or standardized zones (B-b1), scaled by red. The most informative regions (B-b2). Performance of the strut parameters in predicting 1-year implant subsidence (B-c&d).

**P024**

### Alcohol-associated liver disease–induced changes in osteocyte lacunar network and bone mineral density distribution of the femoral neck: postmortem assessment using quantitative backscattered electron imaging

*Jelena Jadzic*[1], *Christine Plumeyer*[2], *Danica Djukic*[3], *Vladimir Zivkovic*[3], *Slobodan Nikolic*[3], *Marija Djuric*[1], *Petar Milovanovic*[1], *Bjorn Busse*[2], *Danijela Djonic*[1]
*[1]University of Belgrade- Faculty of Medicine, Center of Bone Biology, Belgrade, Serbia ; [2]University Medical Center Hamburg-Eppendorf, Department of Osteology and Biomechanics, Hamburg, Germany ; [3]University of Belgrade- Faculty of Medicine, Institute of Forensic Medicine, Belgrade, Serbia*

**Abstract Text**

**Introduction:** Although previous studies suggested that disruptions in bone mineral density distribution (BMDD) and osteocyte lacunar network morphology could play a role in increased bone fragility associated with aging and diseases, these bone properties have not been thoroughly investigated in individuals with *alcohol-associated liver disease* (AALD).

**Purpose:** We conducted an *ex vivo* assessment of osteocyte lacunar network morphology and BMDD in the superolateral femoral neck (SLN) of adult male individuals with AALD.

**Methods:** With the institutional review board approval (No. 1322/V-1), 24 SLN samples were collected during routine autopsies (12 individuals with pathohistological confirmation of AALD [60±12 years] and 12 individuals without pathohistological features of liver disease [59±12 years]), embedded in resin, and scanned using backscattered electron microscopy.

**Results:** Lower mean calcium content and lower peak calcium content were found in the trabecular and cortical compartments of SLN samples obtained from the AALD group compared to the control group (Figure, $p<0.05$). The AALD group showed a significantly higher density of mineralized osteocyte lacunae solely in the trabecular compartment (Figure, $p=0.001$), while none of the parameters of osteocyte lacunar network morphology were significantly altered in the femoral cortex of the AALD group ($p>0.05$).

**Conclusions:** Our findings revealed reduced bone mineralization and an increased proportion of mineralized osteocyte lacunae in SLN of male individuals with AALD, which may contribute to the increased bone fragility in these individuals.

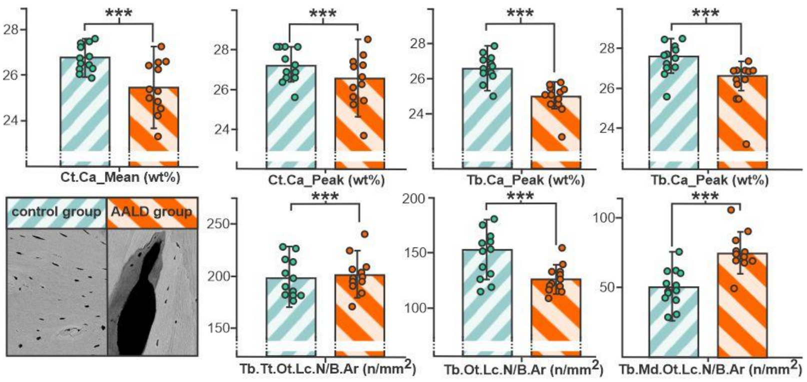

**P025**

**Deteriorated trabecular micro-architecture as an indicator of decreased bone strength in lumbar vertebrae of subjects with intravenous heroin addiction: postmortem micro-computed tomography assessment**

*Jelena Jadzic[1], Nada Tomanovic[2], Aleksa Lekovic[3], Slobodan Nikolic[3], Marija Djuric[1], Danijela Djonic[1]*
*[1]University of Belgrade- Faculty of Medicine, Center of Bone Biology, Belgrade, Serbia ; [2]University of Belgrade- Faculty of Medicine, Institute of Pathology, Belgrade, Serbia ; [3]University of Belgrade- Faculty of Medicine, Institute of Forensic Medicine, Belgrade, Serbia*

**Abstract Text**

**Background:** Previous studies suggested that heroin abuse could be associated with various bone-affecting conditions, including low bone mineral density and, subsequently, potential susceptibility to bone fractures. Still, micro-scale bone features were not previously analyzed in individuals prone to intravenous heroin abuse.

**Purpose:** We aimed to conduct *postmortem* assessment of trabecular and cortical micro-architecture in the anterior mid-transverse portion of the first lumbar vertebral body collected from adult male cadavers with intravenous heroin addiction (HA) and age-matched control group.

**Methods:** With the institutional review board approval (no. 1322/VII-17), 1 cm-thick bone samples were collected during autopsies from 36 male individuals. Based on macroscopic stigmata of intravenous heroin abuse and pathohistological analysis of liver tissue, individuals were divided into the HA group (n=22, age range: 26-49 years) and a control group (n=14, age range: 23-45 years). The trabecular and cortical micro-architectural parameters were analyzed using the Skyscan 1172 micro-CT system (Bruker, Belgium) operated under 10 µm spatial resolution.

**Results:** A declining trend in trabecular bone volume fraction, trabecular thickness, and fractal dimension was noted in the HA group (Figure, $p<0.05$), while cortical micro-architectural parameters were not significantly affected in these individuals ($p>0.05$).

**Conclusions:** Our data revealed dominant trabecular micro-architectural impairment in the vertebral body of individuals with HA, while the cortical bone was not severely affected. Further studies with a more detailed analysis of bone quality features are necessary to fully understand bone alterations in individuals prone to heroin abuse.

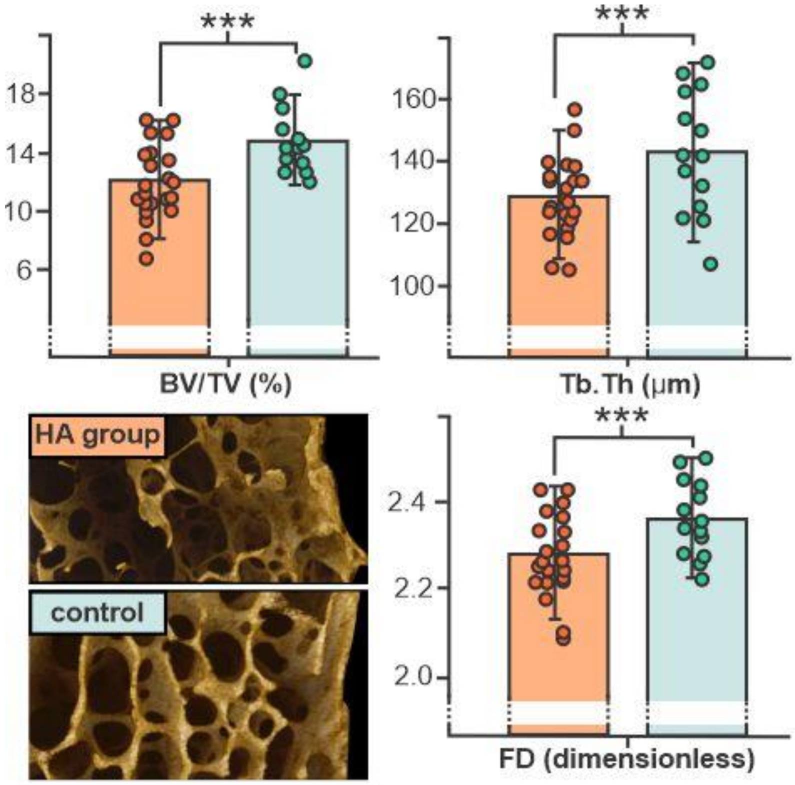

**P026**

**Dual-energy x-ray absorptiometry (DXA) differentiates body composition and bone mineral density in different mouse strains**

*Tiina Kähkönen[1], Jussi Halleen[1]*
*[1]OncoBone Ltd, Oulu, Finland*

**Abstract Text**

Imaging methods in bone research include Dual-energy X-ray Absorptiometry (DXA), peripheral quantitative computed tomography (pQCT) and micro computed tomography (µCT), and DXA and nuclear magnetic resonance (NMR) in metabolic research. These methods have different advantages, and it can be challenging to choose the right methods for a study. DXA technology utilizes variable absorption of X-rays by different body components that allows reliable analysis of body composition and bone mineral density (BMD). DXA is the gold-standard bone analysis method in humans, but its full potential has not been exploited in preclinical studies.

DXA and pQCT are the best methods to analyze BMD in bone research. pQCT allows separate analysis of cortical and trabecular bone areas of imaged bones, while DXA allows whole-body imaging and analysis from selected Regions of Interest (ROIs). µCT in turn allows analyzing total bone volume from trabecular and cortical bone compartments separately. DXA and pQCT allow repeated measurements in longitudinal studies, while µCT is commonly an endpoint measurement. In metabolic research, both DXA and NMR can analyze fat and lean mass. The benefit of DXA is the analysis of specific ROIs from whole-body images, whereas NMR only provides results per animal.

In validation of small-animal DXA iNSiGHT, body composition and BMD were analyzed in three different mouse strains. C3H mice had the highest body weight and the highest fat and lean mass, followed by C57BL6 and BALB/c mice. BMD values demonstrated similar findings, suggesting that C3H mice with highest body mass had also highest BMD values, followed by C57BL6 and BALB/c mice. The DXA results correlated with more detailed bone analysis with µCT and bone histomorphometry.

We conclude that rapid whole-body imaging and ROI analysis are the main advantages of DXA. Mouse strains have variable body composition and BMD, which can affect the study outcome.

**P027**

**Micro-architectural differences in the proximal tibia and distal femur of patients treated with total knee arthroplasty due to primary knee osteoarthritis: a pilot study**

*Andreja Baljozovic[1], Jelena Jadzic[2], Aleksa Lekovic[3], Slobodan Nikolic[3], Zoran Bascarevic[1], Marija Djuric[2], Danijela Djonic[2]*
*[1]University of Belgrade- Faculty of Medicine, Institute for Orthopedic Surgery "Banjica", Belgrade, Serbia ; [2]University of Belgrade- Faculty of Medicine, Center of Bone Biology, Belgrade, Serbia ; [3]University of Belgrade- Faculty of Medicine, Institute of Forensic Medicine, Belgrade, Serbia*

**Abstract Text**

**Background:** Current data regarding subchondral trabecular micro-architecture in individuals with primary knee osteoarthritis (OA) is conflicting due to the various study designs and methodological approaches applied in previous studies.

**Purpose:** We applied high-resolution micro-CT imaging to analyze the subchondral trabecular micro-architecture in individuals with primary knee OA, which could contribute to the understanding of the reasons for various effects of surgical treatment reported in these patients.

**Methods:** With the institutional review board approval, this study encompassed a total of 240 bone samples collected during surgery and autopsy (Figure). Patients who were subjected to total knee arthroplasty due to primary knee OA were included in the OA group (n=20, age range: 60-84 years), while sex-matched cadavers without macroscopic signs of knee OA were included in a control group (n=20, age range: 54-85 years). The subchondral bone micro-architectural parameters of proximal tibia (Figure, n=160) and distal femora samples (Figure, n=80) were analyzed using micro-CT system operated under 10 µm spatial resolution.

**Results:** Our data revealed a site-specific alterations in subchondral bone micro-architecture, predominately due to higher bone volume fraction and trabecular thickness of the OA group (Figure, p<0.05), indicating that OA may reflect differences in the medial-to-lateral load distribution in the knee joint.

**Conclusions:** Observed site-specificity of subchondral trabecular micro-architectural alterations could be morphological factors contributing to different effects of surgical treatment in individuals with primary knee OA, warranting further research.

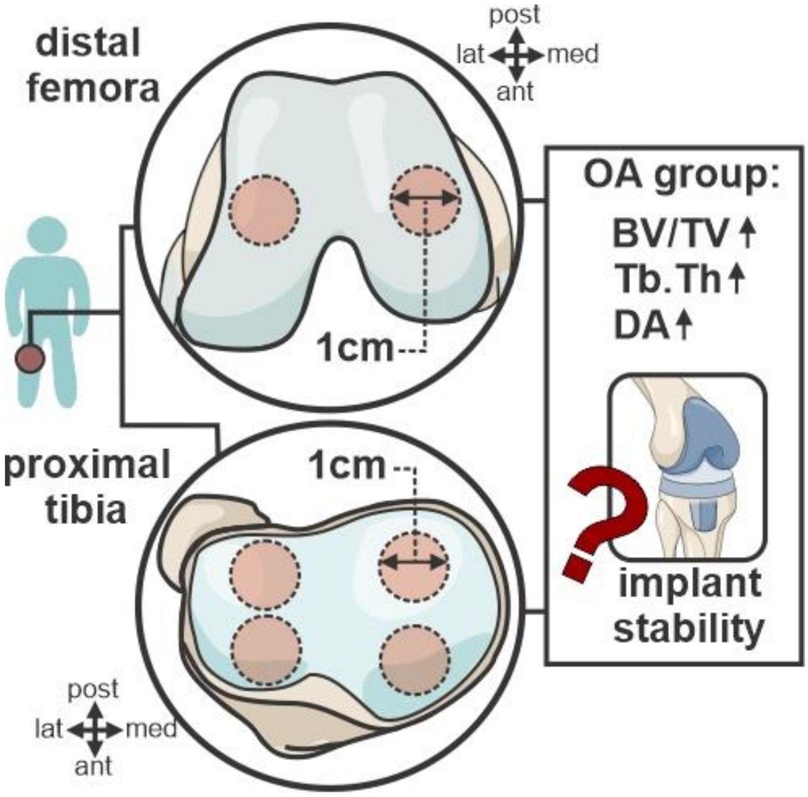

**P028**

**Donepezil and memantine did not affect the musculoskeletal system in an experimental model of sporadic Alzheimer's disease in estrogen-deficient rats**

*Piotr Londzin[1], Joanna Folwarczna[1]*
*[1]Medical University of Silesia - Katowice, Department of Pharmacology - Faculty of Pharmaceutical Sciences in Sosnowiec, Sosnowiec, Poland*

**Abstract Text**

Alzheimer's disease (AD) and osteoporosis are diseases of old age occurring more frequently in women. The data on the effects of donepezil and memantine, drugs used in the treatment of AD, on the musculoskeletal system are very scarce and inconsistent. We have recently reported slight unfavorable effects of donepezil or memantine administration on the skeletal system in healthy non-ovariectomized (NOVX) rats. The aim of the present study was to investigate the effects of donepezil or memantine administration on the musculoskeletal system in estrogen-deficient rats with streptozotocin (STZ)-induced sporadic AD.

The experiments were performed on mature female Wistar rats, divided into 5 groups (*n*=10-12): sham-operated control rats, ovariectomized (OVX) control rats, OVX rats with experimental AD, OVX rats with experimental AD treated with donepezil, and OVX rats with experimental AD treated with memantine. Experimental AD was induced by a single *i.c.v.* injection of STZ one week after the bilateral ovariectomy. Administration of donepezil (1 mg/kg *p.o.*, once daily) or memantine (2 mg/kg *p.o.*, once daily) started four weeks after the STZ injection and lasted four weeks. Bone mass, density, composition and mechanical properties, and skeletal muscle mass and strength were determined.

Estrogen deficiency strongly worsened cancellous bone mechanical properties and increased skeletal muscle mass in the OVX control rats. There was no significant effect of *i.c.v.* STZ on the musculoskeletal system compared to the OVX control rats. Administration of donepezil or memantine did not significantly affect any of the investigated parameters in relation to the untreated OVX rats with experimental AD.

In conclusion, results of the present study indicate no significant effects of donepezil or memantine on the musculoskeletal system in an experimental model of sporadic AD induced by *i.c.v.* injection of STZ in estrogen-deficient rats.

**P029**

**Metal(loid) profiling in femoral neck trabeculae: a comparative study between individuals with fractures and healthy controls, including correlation analysis with bone microarchitecture**

*Aleksandar Cirovic[1], Danijela Djonic[2], Vladimir Zivkovic[3], Slobodan Nikolic[3], Marija Djuric[4], Petar Milovanovic[1]*
*[1]University of Belgrade - Faculty of Medicine, Center of Bone Biology- Dr Subotica 4/2- Belgrade, Belgrade, Serbia ; [2]University of Belgrade - Faculty of Medicine-, Center of Bone Biology- Dr Subotica 4/2- Belgrade, Belgrade, Serbia ; [3]University of Belgrade - Faculty of Medicine, Institute of Forensic Medicine- Deligradska 31a- Belgrade, Belgrade, Serbia ; [4]University of Belgrade - Faculty of Medicine, Center of Bone Biology- Dr Subotica 4/2- Belgrade-, Belgrade, Serbia*

**Abstract Text**

**Background**: Increased urine/blood concentrations of heavy metals are linked to an elevated risk of femoral neck fractures, but studies dedicated to directly measuring metal(loid) concentrations in the femoral neck are limited.

**Purpose**: The aims of this study were to investigate whether individuals who experienced fractures exhibit a different pattern of metal(loid) bioaccumulation in the femoral neck and to examine potential correlations between concentrations of various metal(loid)s and microarchitecture of the femoral neck trabeculae.

**Methods**: We collected the femoral neck specimens from 23 individuals, 11 individuals with a positive history of femoral neck fractures (9 women and 2 men, mean age 77.7±8.1 years) and 12 individuals without fractures (n=12, 10 women and 2 men, mean age 79.5±5.6 years). All samples were subject to microcomputed tomography (micro-CT) to evaluate bone microarchitecture and inductively coupled plasma–mass spectrometry to determine tissue concentrations of metal(loid)s. The research was approved by the Ethics Committee of the Faculty of Medicine.

**Results:** The groups were similar in age (p>0.05). Individuals with fracture had lower concentrations of vanadium and rubidium (p<0.001, p=0.042) and a higher concentration of magnesium (p=0.031) in the femoral neck relative to controls. BV/TV inversely correlated with manganese, cadmium, lead, and zinc (r=-0.441, r=-0.436, r=-0.428, r=-0.563, respectively; $p_{all}$< 0.05). Tb.Th inversely correlated with aluminum, zinc, and magnesium (p<0.05). Higher aluminum was associated with lower trabecular connectivity (higher Tb.Pf) (p=0.041). Manganese, strontium, cadmium, and lead correlated positively with Tb.Sp and inversely with Tb.N (p<0.05).

**Conclusion(s)**: Rubidium, vanadium, and magnesium differed between the groups. While the tissue concentration of magnesium was partly associated with bone microarchitecture, the mechanisms by which the differences in these three elements deteriorated bone quality cannot be fully explained by micro-CT findings alone. In contrast, cadmium, lead, manganese, and aluminum demonstrated clear adverse effects on the femoral neck trabecular microarchitecture in humans.

**P030**

**Detailed mapping of cortical and trabecular mineralization, osteocyte lacunar parameters, and microarchitecture along the superolateral femoral neck in subjects with type 2 diabetes mellitus**

*Aleksandar Cirovic[1], Jelena Jadzic[2], Danica Djukic[3], Christine Plumeyer[4], Vladimir Zivkovic[3], Slobodan Nikolic[3], Danijela Djonic[2], Marija Djuric[2], Björn Busse[4], Petar Milovanovic[2]*
*[1]University of Belgrade Faculty of Medicine, Center of Bone Biology- Dr Subotica 4/2, Belgrade, Serbia ; [2]University of Belgrade Faculty of Medicine-, Center of Bone Biology- Dr Subotica 4/2, Belgrade, Serbia ; [3]University of Belgrade Faculty of Medicine-, Institute of Forensic Medicine- Deligradska 31a, Belgrade, Serbia ; [4]University Medical Center Hamburg-Eppendorf-, Department of Osteology and Biomechanics- Lottestrasse 55a, Hamburg, Germany*

**Abstract Text**

**Background:** The prevalence and costs of fractures related to type 2 diabetes mellitus (T2DM) are on the rise, while the complex interaction between T2DM and bone quality necessitates further investigation.

**Purpose:** To explore how T2DM affects matrix mineralization, osteocyte lacunar characteristics, and microarchitecture of the superolateral femoral neck—the typical fracture-initiating site.

**Methods:** We collected femoral neck specimens post-mortem from elderly men with T2DM and non-DM controls (n=16). Micro-CT was performed to evaluate bone microarchitecture, while backscattered-electron microscopy was used to determine mineralization parameters and osteocyte lacunar characteristics. The research was approved by the institutional review board.

**Results:** The groups did not significantly differ in age, body mass index, and bone mineral density (p>0.05). In the endocortical region, T2DM was associated with lower mineralization degree (lower CaMean: p=0.04), higher proportion of extremely low–mineralized areas (higher CaLow: p=0.027), and higher mineralization heterogeneity (higher CaWidth: p=0.003) relative to controls, whereas there were no significant intergroup differences in the periosteal region. In the trabecular compartment, T2DM was associated with lower mineral content (lower CaMean: p=0.048; higher CaLow: p=0.012) and higher heterogeneity (higher CaWidth: p=0.007) compared with controls. Cortical porosity was insignificantly higher in the T2DM group (34% vs. 27%, p=0.118), and none of the trabecular microarchitectural parameters (BV/TV, Tb.Th, Tb.N, SMI, Tb.Pf, Tb.Sp) varied significantly between the groups (p>0.05). Osteocyte lacunar density was significantly lower in T2DM

in the endocortical and periosteal regions ($p<0.05$), along with significantly increased percentage of mineralized osteocyte lacunae in the endocortical region ($p<0.05$) and no intergroup differences in the trabecular bone ($p>0.05$).

**Conclusions:** Reduced degree of mineralization along with higher mineralization heterogeneity, lower osteocyte lacunar density, and trends to higher percentage of mineralized lacunae and higher cortical porosity are hallmarks of bone alterations in T2DM that could be associated with increased bone fragility.

**P031**

**Effect of sleeve gastrectomy in bone metabolism in a rat model**

*Malory Couchot[1], Françoise Schimtt[2], Celine Fassot[3], Guillaume Mabilleau[4]*
*[1]University of Angers, Inserm UMR_S 1229 - REGOS team, Angers, France ; [2]University of Angers, hifih UPRES EA 3859, Angers, France ; [3]University of Angers, Inserm U1083 - CNRS 6015, Angers, France ; [4]University of Angers, Inserm UMR_S1229 - REGOS team, Angers, France*

**Abstract Text**

**Background:** Sleeve gastrectomy (SG) is an effective treatment for obesity mostly performed in young women of childbearing age. However, there is now a growing body of evidence that bariatric surgery leads to a higher fracture risk although the mechanisms remain to be elucidated. Furthermore, bone consequences of pregnancy and lactation in SG-women are presently not known.

**Purpose:** Goals of this study were to assess whether bone loss following pregnancy and lactation were reversible, and to better understand the determinants of bone fragility after SG.

**Methods:** Twenty-five Sprague-Dawley females were randomly allocated into 3 groups corresponding to sham/non-obese (n=8), sham/obese (n=7), SG/obese (n=10). All animals were matted with controls male SD rats. 9 weeks after delivery all animals were sacrificed. Longitudinal evaluation of BMD and microarchitecture was performed at the right tibia proximal metaphysis by in vivo microCT. Bone strength was evaluated by 3-point bending. Bone material properties were evaluated by Raman microspectroscopy. ANOVA with Tukey's multiple comparisons test was used.

**Results:** Compared to sham, SG led up to 80% of trabecular bone loss ($p<0.0001$). Physiological bone recovery after pregnancy and lactation was observed in all groups. However, tissue strength was lower in obese animals independently of surgical conditions and associated with lower heterogenicity of the degree of mineralization of the bone matrix ($p=0.0087$).

**Conclusion:** This study demonstrated that SG resulted in a dramatic irreversible trabecular bone loss. The cortical fragility observed in animals on obese diet, with or without surgery, was due to alterations of bone material properties. Further studies are required to ascertain whether such phenomenon is occurring in humans.

**P033**

**Trabecularization of the femoral neck has potential bone quality implications relevant for bone fragility**

*<u>Philipp Thurner</u>[1], Martin Bittner-Frank[1], Andreas Reisinger[2], Orestis Andriotis[1], Dieter Pahr[3]*
*[1]TU Wien, Institute of Lightweight Design and Structural Biomechanics, Vienna, Austria ; [2]Karl Landsteiner University of Health Sciences, Department of Anatomy and Biomechanics- Division Biomechanics, Krems, Austria ; [3]TU Wien / Karl Landsteiner University of Health Sciences, Institute of Lightweight Design and Structural Biomechanics / Department of Anatomy and Biomechanics- Division Biomechanics, Vienna / Krems, Austria*

**Abstract Text**

**Introduction:** Diagnosis of osteoporosis in patients prior to a fragility fracture remains a challenge. Here, knowledge of bone quality and in particular bone material properties might improve diagnostic prediction. In particular, the putative change of bone properties with age and disease is of interest.

**Purpose:** To answer whether osteoporosis affects bone material properties and hence whole bone mechanics we conducted mechanical tests at the meso- and micro scale comparing trabecular and cortical bone from osteoporotic and healthy donors.

**Methods:** We conducted mechanical tensile tests on isolated trabeculae as well as cortical bone from the femoral neck from 10 (OP) osteoporotic donors (fragility fracture) and 10 (CTRL) previously healthy individuals with no prior diagnosed bone disease. Tests were conducted with partial unloading cycles to assess also viscous and plastic behaviour, and fitted using a rheological model consisting of a Maxwell- and a Prandtl-layer in parallel arrangement. Envelope curves were assessed similar to quasistatic tests.

**Results:** While OP bone was not found to differ from CTRL bone in both compartments, trabecular and cortical bone tissue were found to differ significantly. Cortical bone showed significantly higher tensile moduli both for rheological model and envelope analysis for cortical (17.2±6.1) GPa compared to trabecular (8.8±5.0) GPa bone ($p<0.001$). Qualitatively, cortical bone specimens showed an almost linear elastic behaviour at low strains, followed by a short stress-hardening phase. In contrast, trabecular bone specimens showed a rather non-linear phase at small strains and a much longer post-yield hardening (cf. Table for values and statistics).

**Conclusion:** Material and mechanical properties of cortical and trabecular bone tissue of the proximal femur are not significantly changed in patients sustaining low-trauma fracture.
However, trabecular and cortical bone tissue at the femoral neck are distinctly different tissues. With trabecularization of the endosteal femoral neck in ageing and osteoporosis, whole bone mechanics might be weakened by local changes in material properties.

Table: Rheological material (top), apparent mechanical (middle), and tissue mineral properties (bottom) for grouping based on tissue type (trabecular vs. cortical) for a subset of identical donors. Mean values ± std. p: p values determined with a Linear Mixed Effects Model with the effect of age and gender.

| Parameter | trabecular | cortical | p |
|---|---|---|---|
| $E_{\infty}$, GPa | 4.9 ± 2.3 | 13.7 ± 3.5 | **< 0.001** [a] |
| $E_0$, GPa | 8.3 ± 3.5 | 23.0 ± 9.5 | **< 0.001** [a] |
| $\sigma_y$, MPa | 30 ± 15 | 50 ± 9 | **< 0.001** [a,b] |
| $p$ | 41 ± 30 | 184 ± 115 | **< 0.001** [a] |
| $\sigma_u$, MPa | 91 ± 35 | 68 ± 16 | **< 0.001** [a] |
| $\eta$, GPas | 9.3 ± 5.1 | 17.9 ± 10.9 | **< 0.001** [a] |
| $\tan\delta$ | 0.021 ± 0.014 | 0.070 ± 0.087 | 0.059 [c] |
| $D_{max}$ | 0.46 ± 0.26 | 0.61 ± 0.19 | **0.005** [a] |
| $D_{end}$ | 0.60 ± 0.18 | 0.73 ± 0.12 | **0.004** [a] |
| $\hat{E}$, GPa | 8.8 ± 5.0 | 17.2 ± 6.1 | **< 0.001** [a] |
| $\hat{\varepsilon}_y$, % | 0.22 ± 0.16 | 0.25 ± 0.28 | 0.870 |
| $\hat{\varepsilon}_u$, % | 5.1 ± 2.2 | 2.6 ± 1.3 | **< 0.001** [a] |
| $\hat{\sigma}_{max}$, MPa | 88 ± 27 | 73 ± 15 | **< 0.001** [a,b] |
| $\hat{W}_{el}$, MJ/m$^3$ | 0.017 ± 0.017 | 0.128 ± 0.215 | **0.005** [a] |
| $\hat{W}_{py}$, MJ/m$^3$ | 3.2 ± 1.9 | 1.6 ± 1.2 | **< 0.001** [a] |
| $TMD_{mean}$, mgHA/cm$^3$ | 966 ± 37 | 1040 ± 21 | **< 0.001** [a] |
| $TMD_{std}$, mgHA/cm$^3$ | 175 ± 10 | 151 ± 10 | **< 0.001** [a] |

[a] significant p values (< 0.05), marked bold.
[b] Reduced model (low trauma fracture and sex without age as grouping variable) if original model did not converge.
[c] p -values indicating a tendency (0.05 < p < 0.07).

**ND-P007**

**Areal bone mineral density (aBMD) increases and trabecular bone score (TBS) decreases with vertebral compression**

*Sofía Aparicio-Ródenas[1], Daniela Frauchiger[2], Mathieu Simon[1], Stefan Bracher[1], Kurt Lippuner[2], Philippe Zysset[1]*
*[1]University of Bern, ARTORG Centre, Bern, Switzerland ; [2]Bern University Hospital, Policlinic for Osteoporosis, Bern, Switzerland*

**Abstract Text**

Trabecular bone score (TBS) based on DXA contributes moderately to vertebral fracture prediction, but previous *ex vivo* work suggested that TBS of dissected vertebrae does correlates poorly or not at all with architectural parameters or vertebral strength. Accordingly, the physical mechanism underlying the contribution of TBS to fracture risk remains obscure. The aim of this study is to investigate aBMD and TBS in an *ex vivo* model of progressive vertebral compression fracture. Fresh-frozen human lumbar spines (T12-L5) were obtained from five donors (3F & 2M, age 76-88) with informed consent. Both aBMD and TBS (Medimaps 2.1.2.0) of individual vertebra were measured by DXA (Discovery A, Hologic) in the intact spine (*in situ*). The vertebral bodies (L1-L4, n=20) were dissected out, and their posterior elements and endplates were removed. The resulting plano-parallel vertebral sections were scanned (*ex situ*) with DXA and HR-pQCT to retrieve trabecular bone architectural parameters. The vertebral sections were then compressed stepwise in a testing machine to 2, 4, 8, 16 and 32% axial strain. After each step, the residual height was measured and a DXA scan was repeated. As a first observation, *in situ* vertebral aBMD correlated moderately with *ex situ* aBMD ($R^2_{adj}$=0.42, p=0.001). Furthermore, *in situ* TBS was only weakly correlated to *ex situ* TBS ($R^2_{adj}$=0.19, p=0.030). Only weak relationships were found between *in situ* aBMD and architectural parameters ($R^2_{adj}$=0.12-0.27, p>0.011), but stronger and significant correlations ($R^2_{adj}$=0.32-0.62, p<0.006) were obtained for *ex situ* aBMD. As shown below, aBMD increased strongly and significantly ($R^2_{adj}$=0.88, p<0.001), while TBS decreased weakly but significantly ($R^2_{adj}$=0.05, p=0.013) with vertebral compression.

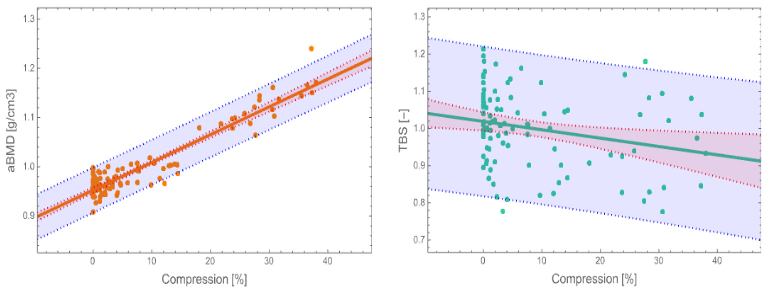

In conclusion, aBMD and especially TBS are sensitive to extraction of the vertebral sections. Compression of the vertebral sections *ex situ* not only increases aBMD, but also decreases TBS, which may potentially explain the contribution of TBS in predicting future fractures.

**ND-P008**

**Relating bone fracture properties across scales from molecular to macro**

*Richard Stavri*[1], *Ulrich Hansen*[2], *<u>Richard L Abel</u>*[1]
[1]*Imperial College London, Surgery and Cancer, London, United Kingdom ;* [2]*Imperial College London, Mechanical Engineering, London, United Kingdom*

**Abstract Text**

The nanoscale mechanics of bone are not well characterized, even though tissue-level properties are well understood. We pursued an investigation into the connections between bone nanomechanics and tissue-level mechanics using an experimental approach. Our central hypotheses were that: (1) nanoscale strain would be reduced in hip fracture patients compared to controls; and (2) nanoscale mineral and fibril strains would negatively correlate with aging and fracture. We examined a cross-sectional sample of trabecular bone sections from the proximal femur of two donor groups: aging controls without fracture (n=17) and hip fracture cases (n=20), aged 44-94 years. Employing synchrotron X-ray diffraction, we simultaneously quantified tissue, fibril, and mineral strain during tensile loading to failure. We then compared strains between groups utilizing unpaired t-tests and correlated strains with age through Pearson analysis. Controls showed significantly higher peak tissue, mineral, and fibril strains versus hip fracture patients (all $p<0.05$). Age correlated with decreased peak tissue ($p=0.099$) and mineral ($p=0.004$) strains, but not fibril strain ($p=0.260$). Overall, hip fracture and aging were linked to alterations in nanoscale strain corresponding to changes at the tissue level. While causation cannot be determined due to the cross-sectional design, we propose two new hypotheses about the importance of bone nanomechanics based on the data. (1) Hip fracture risk rises with low tissue strain, potentially attributable to low collagen or mineral strain. (2) Age-dependent loss of tissue strain relies upon declining mineral strain but not fibril strain. These initial findings provide new perspectives on multi-scale skeletal mechanics that could eventually enable diagnostic and preventative innovations grounded in failure mechanisms originating from the nanoscale.

**ND-P009**

**BMD changes in kidney transplant recipients treated with denosumab: a quasi-experimental explorative study**

*<u>Francesco Pollastri</u>*[1], *Giovanni Adami*[1], *Stefano Andreola*[2], *Matteo Gatti*[1], *Davide Gatti*[1], *Maurizio Rossini*[1], *Ombretta Viapiana*[1], *Giovanni Gambaro*[2], *Riccardo Bixio*[1], *Angelo Fassio*[1]

[1]Azienda Ospedaliera Universitaria di Verona, Rheumatology Unit, Verona, Italy ; [2]Azienda Ospedaliera Universitaria di Verona, Nephrology Unit, Verona, Italy

**Abstract Text**

**Background:** denosumab is a monoclonal antibody without safety limitations in subjects with impaired kidney function; data in kidney transplant recipients (KTR) are scarce.

**Purpose:** we investigated the effects on bone mineral density (BMD) of five years of denosumab treatment in KTR subjects and compared them to a matched untreated cohort.

**Methods:** we longitudinally followed KTR-subjects starting treatment with denosumab. We included a sex-age-matched control arm of untreated KTR-subjects. Clinical and biochemical data were recorded and a DXA scan was performed at baseline and after five years (lumbar spine, total hip, femoral neck) for both groups. One-way ANOVA was used to test for baseline differences between the two arms at baseline, chi-squared for proportions, one-way ANOVA for repeated measurements to analyse BMD changes over time in the two arms, mixed-design ANOVA to test for differences in the BMD changes over time between the two groups. Written informed consent was obtained from all participants included (protocol 1483 CESC).

**Results:** 23 kidney transplant recipients were enrolled and 23 age and sex-matched controls. Significant baseline differences were observed for BMI and BMD. The changes in BMD are depicted in figure 1. The treated arm showed a significant increase in BMD at all sites, the control arm showed a significant decrease, at all sites. The Z-score in the treated arm significantly increased, while it decreased at the femoral neck and total hip in the untreated group. The between-groups p for interaction for denosumab treatment was statistically significant at all sites. No differences over time in serum creatinine were documented.

**Conclusions:** in KTR subjects, denosumab is associated with a significant increase in BMD at all sites. In the untreated population, a significant decrease was shown, greater than what expected for aging, especially at the femoral sites.

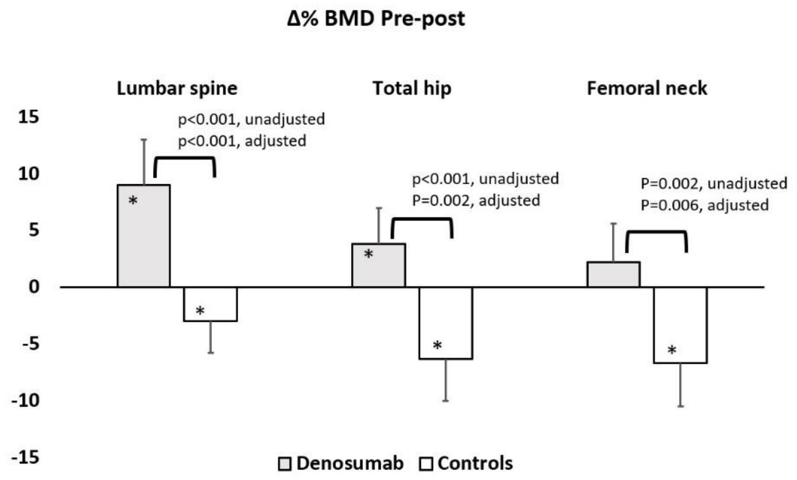

**ND-P010**

**Long-term femoral neck structural changes in kidney transplant recipients treated with denosumab: a quasi-experimental study**

*Francesco Pollastri*[1]*, Giovanni Adami*[1]*, Stefano Andreola*[2]*, Davide Gatti*[1]*, Ombretta Viapiana*[1]*, Matteo Gatti*[3]*, Riccardo Bixio*[1]*, Giovanni Gambaro*[2]*, Maurizio Rossini*[1]*, Chiara Caletti*[1]*, Paolo Fabbrini*[3]*, Angelo Fassio*[1]

[1]*Azienda Ospedaliera Universitaria di Verona, Rheumatology Unit, Verona, Italy ;* [2]*Azienda Ospedaliera Universitaria di Verona, Nephrology Unit, Verona, Italy ;* [3]*ASST Nord Milano, Nephrology Unit, Milano, Italy*

**Abstract Text**

**Background and Purpose:**
denosumab is a monoclonal antibody without safety limitations in subjects with impaired kidney function, we investigated the effects of five-years denosumab therapy on kidney transplant recipients (KTR) and compared them to a matched untreated cohort.

**Methods:**
we longitudinally followed KTR treated with denosumab and sex-age-matched control arm of untreated KTR-subjects, collecting clinical, biochemical and DXA scans at baseline and after five years for both groups. We used the 3D-Shaper software on DXA scans at femoral site. One-way ANOVA was used to test for differences between the two arms at baseline, chi-squared for proportions, one-way ANOVA for repeated measurements to analyse changes in cortical and trabecular bone and strenght indices over time in the two arms. Written informed consent was obtained from all participants included (protocol 1483 CESC).

**Results:** Twenty-three denosumab-treated KTRs were enrolled, as well as twenty-three controls.
The mean time from transplant to the start of denosumab therapy was 7.8±7.6 years.
The treated arm showed a significant increase from baseline in femoral neck cortical thickness (1.75 [1.60;1.82] - 1.80 [1.73;1.88], p= 0.049).
The control arm showed a significant decrease in femoral neck cortical volumetric bone mineral density (729 [687;771] - 690 [649;732], p <0.001), femoral neck cortical surface bone mineral density (134 [122;145] - 124 [112;135], p<0.001), and in cortical surface buckling ratio (11.4 [10.7; 12.0] - 12.2 [11.5; 12.8], p <0.001), cross-sectional area (0.889 [0.723; 0.934] - 0.789 [0.745; 0.959], p<0.001), and cross-sectional moment of inertia (1.34 [1.09; 1.58] - 1.20 [0.94; 1.45], p=0.002).
The between-groups p for interaction for denosumab treatment was statistically significant at all sites.
No significant changes in serum creatinine or cases of hypocalcemia were observed.

**Conclusions:** the whole area of the hip region improved in the denosumab treated group and similar trajectories were found in the estimated strength areas.

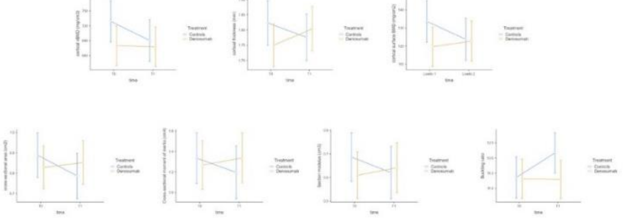

**Poster Sessions: Bone development/growth and fracture repair**

**P034**

**Endothelial YAP/TAZ controls bone marrow fibrosis and Osteosclerosis**

*Kishor Kumar Sivaraj*[1], *Paul-Georg Majev*[1], *Backialakshmi Dharmalingam*[1], *Bella Banjanin*[2], *Rebekka K. Schneider*[2], *Ralf H. Adams*[1]
[1]*Max Planck Institute for Molecular Biomedicine, Tissue Morphogenesis, Münster, Germany*
*;* [2]*Erasmus Medical Center Cancer Institute, Developmental Biology, Rotterdam, Netherlands*

**Abstract Text**

**Background**: Myelofibrosis and osteosclerosis are fibrotic diseases disrupting bone marrow function, which occur in various leukemias but also in response to non-malignant alterations in hematopoietic cells.

**Purpose**: Here, we show that Yap/Taz regulates the plasticity of endothelial cells in an organ-specific manner. During bone remodeling, Yap/Taz activation in endothelial cells promotes bone marrow fibrosis and osteosclerosis.

**Methods:** We used mouse genetic to activate *Yap1*, and inactivate *Yap1/Taz*, *Lats2*, and *Srf* in endothelial cells. In addition, we performed lineage tracing, high-resolution imaging, single-cell RNA-sequencing (scRNA-seq) and computation analysis to show Endothelial mediated signals control bone remodeling.

**Results**: Here, we show that endothelial cell-specific inactivation of the *Lats2* gene, encoding Hippo kinase large tumor suppressor kinase 2, or overexpression of the downstream effector YAP1 induce myofibroblast formation and lead to extensive fibrosis and osteosclerosis, which impair bone marrow function and cause extramedullary hematopoiesis. Mechanistically, loss of LATS2 induces endothelial-to-mesenchymal transition, resulting in increased expression of extracellular matrix and secreted signaling molecules. Changes in endothelial cells involve increased expression of serum response factor target genes and, strikingly, major aspects of the LATS2 mutant phenotype are rescued by inactivation of the *Srf* gene.

**Conclusion**: Our findings identify the endothelium as a driver of bone marrow fibrosis, which improves our understanding of myelofibrotic and osteosclerotic diseases for which we currently lack drug therapies.

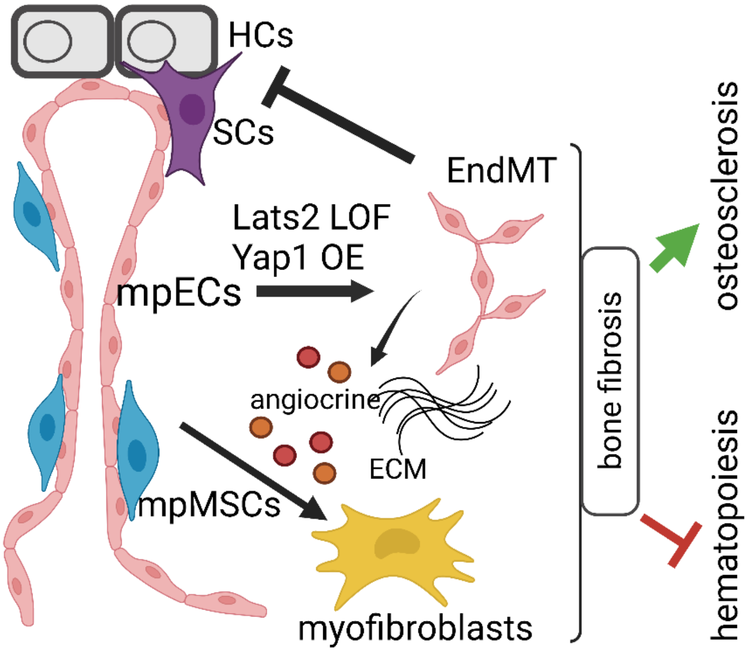

**Figure**: Proposed model of YAP1/TAZ-induced endothelial-to-mesenchymal transition (EndMT) as a driver of myelofibrosis and osteosclerosis. mp: Metaphysis; ECs; Endothelial cells; MSCs: Mesenchymal stromal cells; SCs: Septoclasts; HCs: hypertrophic chondrocytes; ECM: Extracellular matrix

**P035**

**A Molecular Mechanism for Fracture Nonunion**

*José Valdés-Fernández[1], Miguel Echanove-González de Anleo[1], Tania López-Martínez[1], Purificación Ripalda-Cemboráin[1], Juan Antonio Romero-Torrecilla[1], Emma Muiños-López[1], Froilán Granero-Moltó[1]*
*[1]Clínica Universidad de Navarra, Cell Therapy/Orthopedic Surgery and Traumatology, Pamplona, Spain*

**Abstract Text**

**Background**: After injury, bone tissue initiates a reparative response that recovers its structure and function. Failure or delay during early reparative stages results in fracture nonunion. The molecular mechanisms underlying fracture nonunion appearance are unknown. Previous results suggest that the signaling pathways derived from hypoxia signaling and reactive oxygen species (ROS) homeostasis control *Bmp2* expression and fracture initiation, having potential to induce fracture nonunion when disturbed.

**Purpose**: To determine how fracture healing is affected when *Apex1*, a nuclear factor and ROS signaling effector, is silenced.

**Methods**: All animal procedures were approved by our institutional committee for animal use in research. A closed fracture was performed in the tibia of Prrx1Cre;Apex1$^{C/C}$ (Apex1$^{KO}$) and WT mice littermates. The repair process was evaluated along the reparative phase (3-, 6-, 10- 14- and 21-dpf) by histology, immunohistochemistry, transcriptomic analysis (RNA-seq) and microcomputed tomography (mCT).

**Results:** mCT analysis showed that Apex$^{KO}$ mice presented an impaired healing response. Apex1$^{KO}$ mice develop a significant reduced callus when compared with their WT littermates (WT, 32.04±10.50 mm$^3$; Apex1$^{KO}$, 14,45±9,35 mm$^3$; p<0.0001), presenting a significative volume reduction of both soft (WT, 16.63±6.47 mm$^3$; Apex1$^{KO}$, 6.47±5.46 mm$^3$; p<0.0001) and stiff (WT, 15.41±4.68 mm$^3$; Apex1$^{KO}$, 7.97±5.345 mm$^3$; p=0.0006) tissues. At 21-dpf, radiographic analysis showed that Apex1$^{KO}$ mice present a persistent fracture line and sclerotic bone, defining a nonunion phenotype (Figure 1). Transcriptomic and histologic analysis showed that periosteal response to injury at 3-dpf was impaired in Apex1$^{KO}$ mice, resulting in reduced chondrogenesis and osteogenesis during the reparative phase at 6-, 10- and 14-dpf.

In conclusion, silencing *Apex1* in mesenchyme progenitors results in delayed fracture response, impaired endochondral ossification and nonunion-like phenotype. Thus, hypoxia signaling and ROS have a major role in fracture healing initiation identifying a molecular pathway for fracture nonunion appearance.

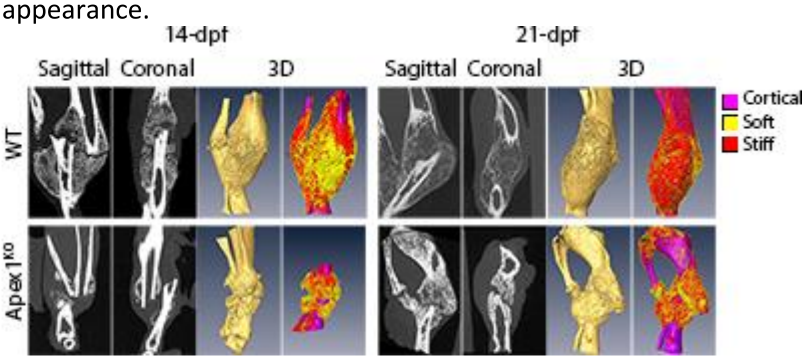

**P036**

**Spatial distribution and role of PDGFRα+ skeletal stem/progenitor cells in bone regeneration**

*Maria Ethel*[1], *Cassandre Goachet*[1], *Simon Perrin*[1], *Salma Lakhlifi*[1], *Céline Colnot*[1]
[1]*Institut Mondor de recherche biomédicale, Inserm, Creteil, France*

**Abstract Text**

Bone regeneration relies on the activation and recruitment of skeletal stem/progenitor cells (SSPCs) forming bone and cartilage after injury. SSPCs represent a diversity of cell populations residing in bone (i.e., periosteum and bone marrow) and adjacent skeletal muscle. Understanding the relative contribution and fate of SSPCs from different origins remains challenging due to the lack of tissue-specific marker. Here, we show that Platelet-derived growth factor receptor alpha (PDGFRα) is a pan marker of all SSPC populations located in bone marrow, periosteum and skeletal muscle. Using Pdgfra$^{CreERT2}$ inducible mouse model, we developed a local tamoxifen metabolite delivery approach (endoxifen gel) to specifically target PDGFRα+ SSPCs in periosteum and skeletal muscle in vivo (figure). Lineage tracing and local depletion of this population demonstrate that PDGFRα+ SSPCs in periosteum and muscle are the main contributors to bone repair compared to bone marrow-derived SSPCs (figure). Inactivation of *Sox9*, the master regulator of chondrogenesis, in PDGFRα+ SSPCs localized in periosteum and skeletal muscle prevents callus formation and bone bridging revealing the requirement of these SSPC populations to bone healing via endochondral ossification. However, single cell RNAseq of periosteum and skeletal muscle SSPCs show that these 2 populations exhibit distinct fates in response to bone fracture. These results elucidate the diverse roles of PDGFRα+ SSPCs in bone regeneration and highlights the central role of periosteum and skeletal muscle SSPCs during bone repair.

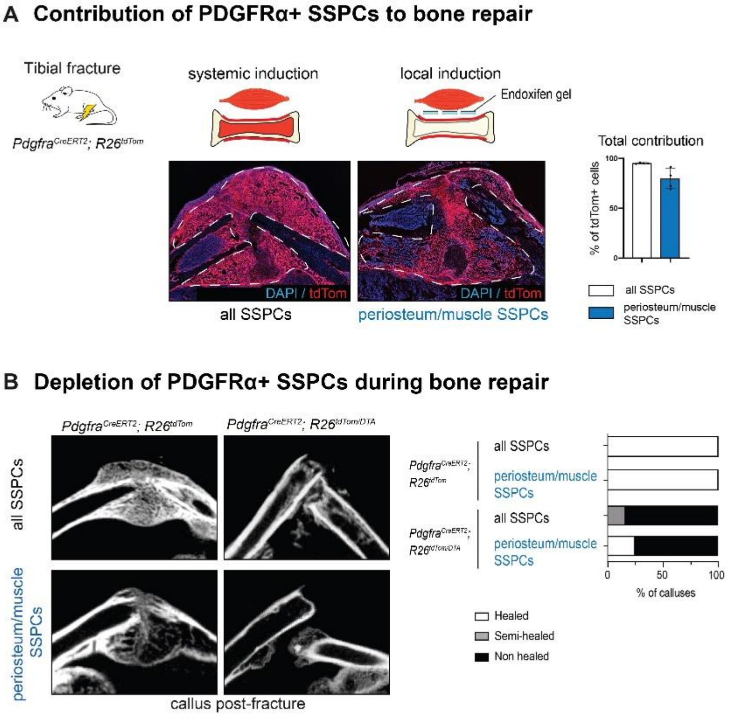

**P037**

**Staphylococcus epidermidis peri-implant infections - an experimental pilot study in mice**

*Christine Linda Dowling*[1], *Samira Weisselberg*[2], *Tobias Malte Ballhause*[1], *Anke Baranowsky*[1], *Holger Rohde*[2], *Johannes Keller*[1]

[1]*University Medical Center Hamburg-Eppendorf, Trauma and Orthopedic Surgery, Hamburg, Germany ;* [2]*University Medical Center Hamburg-Eppendorf, Medical Microbiology- Virology and Hygiene, Hamburg, Germany*

**Abstract Text**

**Introduction:** Arthroplasty and osteosynthesis are two of the most common orthopedic procedures. In this regard, peri-implant infections remain one of the most serious complications with high morbidity, often caused by biofilm (BF)-forming bacteria including *Staphylococcus epidermidis*. However, the underlying in vivo mechanisms have not been fully elucidated due to a lack of suitable in vivo models that allow bacterial and host factors to be studied at the molecular level.

**Purpose:** To establish a novel mouse model to study the interactions between bone tissue and *S. epidermidis* during peri-implant infection in vivo.

**Methods:** Clinical, BF-forming *S. epidermidis* wild type 1457 and a corresponding, isogenic BF-negative mutant were employed in infection experiments. Peri-implant infection was achieved in WT mice (C57Bl/6) by drill opening of the distal femur, introducing a standardized volume of bacterial suspension into the medullary cavity, and retrograde insertion of a titanium pin pre-coated with bacteria. Animals (n=3 per group including a NaCl control) were sacrificed after 14 days for microbiological and histological evaluation.

**Results:** Successful peri-implant infection was confirmed microbiologically by detection of viable *S. epidermidis* in the femoral shaft, condyles, and pin surface 14 days after surgery. Histologic evaluation of ground bone samples with the pin in situ revealed thickening and excessive porosity of the cortical bone in infections using BF-positive *S. epidermidis*. Both BF-positive wild type 1457 and the BF-negative mutant caused sclerosis of the lateral femoral condyle, while degradation of the growth plate was observed primarily in animals infected with the BF-positive *S. epidermidis* 1457.

**Conclusion:** This novel in vivo model provides a platform to study different bone infection scenarios, e.g. using different mouse genotypes, *S. epidermidis* strains and/or pharmacological interventions. We propose that this model may be useful to better understand the biological interactions between biofilm-forming bacteria, implant material and cells of the bone microenvironment.

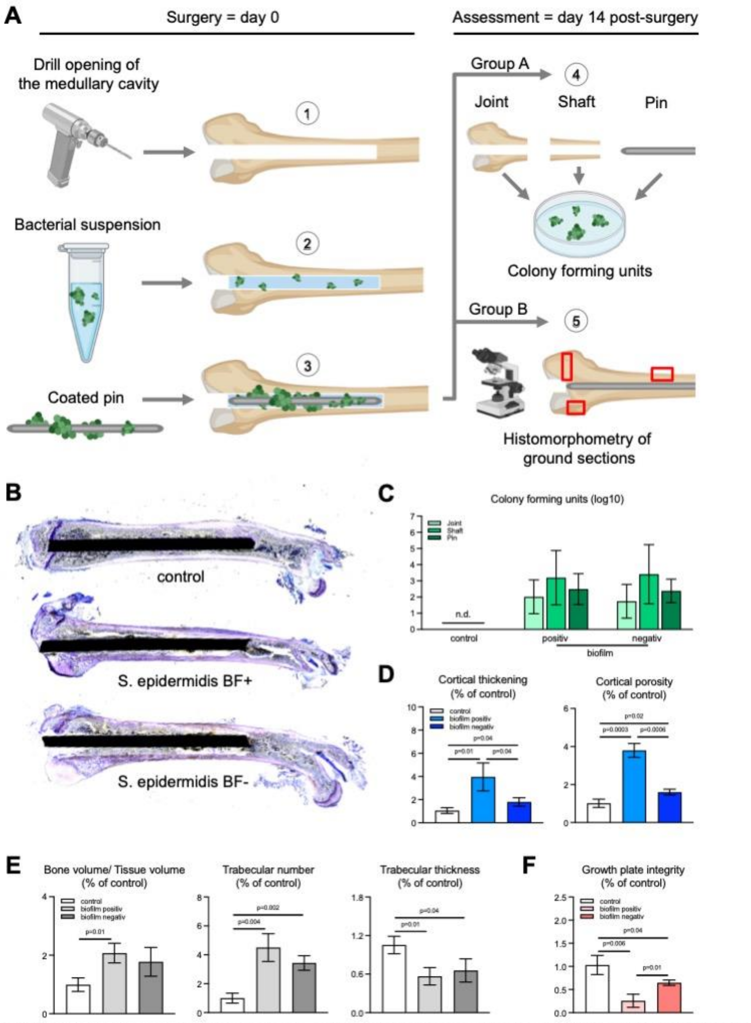

Peri-implant infection with Staphylococcus epidermidis in mice. **A)** Workflow to achieve persistent peri-implant infection with Biofilm (BF)-positive and BF-negative S. epidermidis in the femur of WT mice, including (1) drill opening of the distal femur, (2) placement of a pre-defined volume of bacterial suspension into the medullary cavity, and (3) installation of a bacteria-coated titanium pin. After sacrifice, group A was processed for microbiological assessment of bacterial load in respective compartments (4), while the femur of group B was subjected to ground processing for non-decalcified histological analysis of the three regions of interest marked in red boxes (cortical bone, lateral condyle, growth plate). **B)** Representative toluidine blue-stained ground samples of the femur with the pin in situ. **C)** Determination of bacterial load 14 days after induction of peri-implant infection. nd=nondetectable. **D)** Histomorphometric assessment of cortical bone pathology, **E)** changes in trabecular bone in the lateral condyle, and **F)** degradation of the cartilaginous growth plate. n=3 mice per group. The data are expressed as means ± SD. Mann-Whitney-U test was used for data analysis. All experiments were approved by the local animal care committee.

**P038**

**Bone matrix dependent strength features of the tibia in diabetic rats with repeated fracture after systemic and local calcium administration**

*Nadezhda Mosyagina*[1]
[1]*FSBEI HI ST. LUKA LSMU of MOH of Russia, Human anatomy department, Lugansk, Russia C.I.S.*

**Abstract Text**

**Introduction:** Diabetes specifically affects bone matrix quality via collagen glycation. This in turn may affect bone strength and thus increase risk of fracture. Repeated fractures may require a special attention once they feature more severe osteoporosis resulting from both diabetes and previous fracture. Treatment of fractures and diabetic osteoporosis includes calcium administration. Many studies focus on the effects of calcium on bone strength yet strength behavior after repeated fracture in diabetes and combined systemic and local administration of calcium is not clear.

**Purpose:** To test Young's modulus and specific sag changes of the tibia in rats with repeated fracture after administration of Calcemin Advance and implantation of hydroxyapatite (HA) material into fracture area.

**Methods:** 24 diabetic female rats with repeated fracture modeled as consecutive fractures of the femur and the tibia with 60-day space for the first fracture healing were selected for further manipulations. In these animals, fracture area in the tibia was filled with hydroxyapatite material. Intragastric calcium started next day after intervention. Observation terms were 7, 15, 30, and 60 days. Comparison group included diabetic animals with repeated fractures and HA implants in the tibiae. Strength testing of the tibia employed bending technique.

**Results:** Values of specific sag in the experimental group as compared to the animals with sole HA implants decreased in all observation terms by 6.30%, 12.05%, 13,27%, and 15,59%, which testifies for greater stiffness of the whole organ. Young's modulus in the same terms increased by 13,68%, 8,80%, 4,86%, and 3,80%, which stands for improvement of tissue elasticity.

**Conclusions:** Combined action of HA implants and Calcemin Advance results in marked improvement of stiffness and tissue elasticity that both depend on collagen quality. This may be due to improved bone regeneration under both local and systemic effects of calcium.

**P039**

**TYRA-300 Demonstrates Significant Increases in Bone Length and foramen magnum area in a Mouse Model of FGFR3-Related Skeletal Dysplasia**

*Laurence Legeai-Mallet*[1]*, Clara Lemoine*[2]*, Matthias Guillo*[2]*, Nabil Kaci*[3]*, Jacqueline H. Starrett*[4]*, Ronald V. Swanson*[5]
[1]*Université de Paris Cité- Imagine Institute-, Laboratory of Molecular and Physiopathological Bases of Osteochondrodysplasia- INSERM UMR1163-, Paris, France ;* [2]*Université de Paris Cité- Imagine Institute, Laboratory of Molecular and Physiopathological Bases of Osteochondrodysplasia. INSERM UMR1163, Paris, France ;* [3]*Université de Paris Cité- Imagine Institute, Laboratory of Molecular and Physiopathological Bases of Osteochondrodysplasia. INSERM UMR1163-, Paris, France ;* [4]*Tyra Biosciences-, Tyra, Carlsbad, United States ;* [5]*Tyra Biosciences, Tyra, Carlsbad-, United States*

**Abstract Text**

Achondroplasia (ACH) is the most common human skeletal dysplasia affecting ~1 in 25,000 births. Infants with ACH have an increased risk for death related to critical foramen magnum stenosis leading to cervicomedullary compression. A specific mutation in *FGFR3*, G380R, causes over 99% of achondroplasia. FGFR3 is expressed in growth plate chondrocytes where it functions to slow endochondral bone formation. The G380R mutation, as well as other activating mutations, results in increased FGFR3 activity, which suppresses chondrogenesis in the growth plate, disturbing long bone elongation. Vosoritide, a C-natriuretic peptide analogue, acting exclusively on the MAP kinase pathway, was recently approved as a daily injection to increase annual growth velocity in children with open growth plates. While an important breakthrough, long-term effects on ACH-associated comorbidities are not yet known. TYRA-300 is an oral, highly selective FGFR3 tyrosine kinase inhibitor currently undergoing a Phase 1/2 clinical trial, SURF301 (Study in Untreated and Resistant FGFR3+ Advanced Solid Tumors). To explore the effectiveness of TYRA-300 in FGFR3-related chondrodysplasias, TYRA-300 was evaluated in $Fgfr3^{Y367C/+}$ micemimicking ACH. TYRA-300 administered daily at a 1.2 mg/kg dose for 15 days in the $Fgfr3^{Y367C/+}$ mouse model significantly increased body length in mice by 17.9% compared to the vehicle (p<0.0001) and increased the length of the femur (+22.6%), tibia (+33.0%) and L4-L6 (+23.5%) in mice (p<0.0001). mCT analyses showed a significant increase of the foramen magnum area (+15.08%) in $Fgfr3^{Y367C/+}$ compared to untreated mice (p<0.0001). Histological staining of the femurs revealed restoration of the hypertrophic zone and secondary ossification center within the epiphysis after TYRA-300 treatment. Collagen X staining also indicated that TYRA-300 improved the structure of the trabecular bone and increased chondrocyte differentiation. These data indicate that inhibiting FGFR3 directly leads to highly increased bone length, as well as foramen magnum diameter, in this preclinical model.

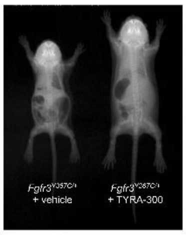

Figure. Impressive growth improvement

**P040**

**Sodium intake as a risk factor for osteoporosis: a systematic review and narrative synthesis of observational studies of adults**

*Mina Nicole Händel[1], Camilla Paludan[2], Isabel Cardoso[2], Sofus Christian Larsen[2], Berit Heitmann[2], Bo Abrahamsen[1]*
*[1]University of Southern Denmark, Research unit OPEN- Department of Clinical Research, Odense, Denmark ; [2]Frederiksberg Hospital, Parker Institute, Copenhagen, Denmark*

**Abstract Text**

**Background:** Understanding the possible role of sodium intake on the aetiology of osteoporosis can be relevant when developing and implementing prevention strategies for osteoporosis and its associated fractures.

**Purpose:** to examine the association between sodium intake/excretion and bone health by summarizing evidence of observational studies

**Methods:** A literature search was performed in 5 electronic databases from inception to August 2023. The inclusion criteria were non-institutionalised adults with measured sodium intake or excretion. Outcomes were any bone indices. A narrative synthesis was undertaken. The certainty of the evidence was evaluated using GRADE and risk of bias was assessed using the quality criteria checklist for primary research of the Academy of Nutrition and Dietetics. PROSPERO registration: CRD42017065360.

**Results:** 34 unique studies met our inclusion criteria (n>160,000): 30 cross-sectional studies and 4 cohort studies. The study populations consisted of either only women or men and women combined, aged 18 to 89 years. 9 cross-sectional studies and 1 cohort study showed no association, and the remaining studies indicated that both low or high sodium intake/excretion was associated with decreased bone mineral density, higher risk of fractures or higher risk of osteoporosis. The certainty in the evidence is very low due to serious risk of bias, risk of inconsistency and risk of publication bias.

**Conclusion(s):** This narrative synthesis confirms how challenging it is to study the association between sodium and bone health. Most of the studies available are cross-sectional, which gives uncertain direction of causality, even though they included large study populations. The methodological limitations in the primary studies - such as lack of adjustment for relevant confounders, misclassification of exposure, missing data, selective reporting bias, and heterogeneous and inconsistent outcome reporting across studies - prohibits meta-analysis. Randomised controlled trials or prospective cohort studies using standardised set of outcomes are needed to establish dietary guidelines.

**P041**

**Chondrocyte-specific Deletion of the β2 Adrenoceptor impairs Bone Growth and Fracture Healing**

*Melanie R. Kuhn[1], Melanie Haffner-Luntzer[1], Sandra Dieterich[1], Anita Ignatius[1], Stefan O. Reber[2], Miriam E. A. Tschaffon-Müller[1]*
*[1]Institute of Orthopaedic Research and Biomechanics, Ulm University Medical Center, Ulm, Germany ; [2]Laboratory for Molecular Psychosomatics- Department of Psychosomatic Medicine and Psychotherapy, Ulm University Medical Center, Ulm, Germany*

**Abstract Text**

**Introduction:** $\beta_2$-adrenoceptor ($\beta_2$-AR) signaling has been shown to influence bone homeostasis. Mouse models with systemic or osteoblast-specific knockout suggest a negative effect of the $\beta_2$-AR on bone. However, the role of $\beta_2$-AR signaling in chondrocytes and its effect on endochondral ossification is still unknown.

**Purpose:** The aim of our study was to determine the role of chondrocyte $\beta_2$-AR signaling in endochondral ossification during bone growth and fracture healing.

**Methods:** 12-week-old male Adrb2$^{fl/fl}$/Col2a1-Cre$^{+}$ mice lacking the $\beta_2$-AR in chondrocytes and Cre$^{-}$ littermates were euthanized and femur and tibia length were measured. Femora were scanned by µCT and subjected to histology and immunohistochemistry to analyze the growth plates. Another group of male Adrb2$^{fl/fl}$/Col2a1-Cre mice underwent a femur osteotomy (approved by the local ethical committee) to evaluate fracture healing after 10 and 21 days.

**Results:** Adrb2$^{fl/fl}$/Col2a1-Cre$^{+}$ vs. Cre$^{-}$ mice displayed shorter femora (14.6 vs. 15.7 mm, $p<0.0001$) and tibiae (Fig. 1A). Immunostainings revealed enlarged hypertrophic zones in growth plates of Adrb2$^{fl/fl}$/Col2a1-Cre$^{+}$ mice with increased numbers of Col10a1$^{+}$ cells (Fig. 1 B, C). No differences in bone mass were observed. 21 days after fracture, Adrb2$^{fl/fl}$/Col2a1-Cre$^{+}$ mice displayed a reduced bending stiffness of the fractured femur (Fig. 1D) as well as a decreased mineralization (746.1 vs. 783.8 mg HA/cm$^3$; $p=0.0252$) and BV/TV (6.5 vs. 13.7%; $p=0.0043$) in the fracture callus, indicating disturbed fracture healing. 10 days post-fracture, facture calli of Cre$^{+}$ mice showed a reduced cartilage (20.5 vs. 46.3%; $p=0.0365$) and increased fibrous tissue area (79.5 vs. 53.5%; $p=0.0359$).

**Conclusion:** Our data indicate a critical role of $\beta_2$-AR signaling in chondrocytes during bone growth and fracture healing. The impaired bone growth of Adrb2$^{fl/fl}$/Col2a1-Cre$^{+}$ mice could possibly be mediated by an accelerated differentiation of proliferative towards hypertrophic chondrocytes, limiting chondrocyte proliferation. Fracture healing could be disturbed by the same mechanism due to impaired cartilage formation in the fracture callus.

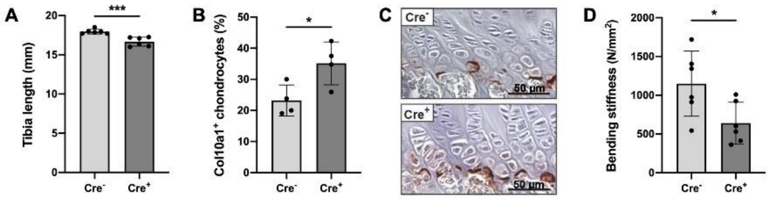

**P042**

**Influence of the exogenous insulin on bone formation**

*Nadezhda Mosyagina[1], Olena Skriabina[1], Larisa Sankova[2]*
*[1]Lugansk State Medical University, Human anatomy and physiology department, Lugansk, Ukraine ; [2]Kyiv medical university, Human anatomy department, Kyiv, Ukraine*

**Abstract Text**

**Introduction:** Bone is always influenced by numerous exo- and endogenous factors. Exogenous insulin is a conventional drug for diabetes mellitus type 1 so many people use it every day in different dosage. Although there are numerous data describing the effects of insulin on the morphogenesis of skeletal bones yet they are controversial and highlight mostly the initial stage of osteogenesis.

**Purpose:** To test the bone growth processes in rats under the influence of exogenous insulin at various stages of osteogenesis.

**Material and methods:** Male rats (n=96) were divided into two groups. The animals of the 1st (control) group received subcutaneous injections with sterile water in volume equal to insulin injection. The animals of the 2nd group received subcutaneous insulin in dose of 3 U/kg per day. Observation terms constituted 7, 14, 30, and 60 days. By the end of observation terms, histological study of the proximal epiphyses and midshaft sections of the tibia was performed.

**Results:** Hyperinsulinemia resulted in increase of bone length on the 7th day by 1.54% as compared to the 1st group. Epiphyseal cartilage exhibited widening, with maximum values found by the 14th day. On the 30th day, length of the tibia decreased by 1.92%. and epiphyseal cartilage narrowed by 16.1% as well. Changes in the transverse dimensions of the bones were more pronounced and were observed throughout the whole experiment. Thus, by the 60th day, the width of the tibia increased by 5.26%.

**Conclusions:** During the first week of insulin administration to rats, activity of the tibial epiphyseal cartilage increased, as well as the rate of their longitudinal and appositional growth. Long-term administration of insulin leads to a decrease of epiphyseal cartilage activities in the tibia. At the same time, longitudinal growth rate of the tibia reduces while appositional growth remained higher.

**P043**

**Syndecan-1-deficiency impairs neoangiogenesis at the chondro-osseous interface in bone and fracture healing**

*Melanie Timmen[1], Christian Arras[2], Nils Roters[1], Daniel Kronenberg[1], M. Gabriele Bixel[3], Ralf H. Adams[3], Richard Stange[1]*
*[1]Institute of Musculoskeletal Medicine- University of Muenster, Dept. of Regenerative Musculoskeletal Medicine, Muenster, Germany ; [2]University Medical Centre Hamburg-Eppendorf, Dept. of Trauma- Hand and Reconstructive Surgery, Hamburg, Germany ; [3]Max Planck Institute for Molecular Biomedicine, Dept. of Tissue Morphogenesis, Muenster, Germany*

**Abstract Text**

**Background**: Neoangiogenesis drives the replacement of mineralized cartilage by trabecular bone during bone growth regulated by molecules like e.g. VEGF, OPG and RANKL. The Heparan-sulfate-proteoglycan Syndecan-1 (Sdc1) while interacting with VEGF and OPG, plays a role in the communication of osteoclasts and osteoblasts and in the development of blood vessels.

**Purpose**: To understand the function of Sdc1 in enchondral ossification we analysed bone structure and vessel development in bone growth and fracture healing in mice deficient in Sdc1.

**Methods**: Femora of WT (n=11) and Sdc1-/- (n=13) mice (male/female) were used for bone analysis. Female mice (WT n=6-14, Sdc1-/- n=6-8, per time point) underwent midshaft femur fracture intramedullary stabilized (Ethic-app: 81-02.04.2019.A164). Bone structure was analysed using microCT. Callus composition was quantified as callus area, fibrous tissue, cartilage and trabecular bone. Vascularisation was visualised by anti-Endomucin antibody. Bone marrow was used for isolation of endothelial progenitor cells and microvessel development was analysed.

**Results**: Bone structure in 4-month-old male Sdc1-deficient mice was significantly reduced compare to age matched male WT. Sdc1-deficient mice showed a significant decreased number of vessel buds at the chondro-osseous border (growth plate) compared to WT (in both male and female mice). After fracture, callus development was delayed with regard to cartilage area at day 7 and trabecular bone area at day 14. A decreased number of vessel buds invading at the borderline of cartilage to bone in the callus were counted in Sdc1-deficient callus tissue. Microvessel outgrowth of endothelial cells from bone marrow revealed a decreased amount of sprouting, but increased length of microvessels of Sdc1-/- cells compared to WT.

**Conclusion**: Syndecan-1 has a significant impact on neoangiogenesis at the chondro-osseous border in bone as well as during bone healing. This emphasises the importance to further characterise the mechanism, how Syndecan-1 regulates the process of endothelial invasion during enchondral ossification.

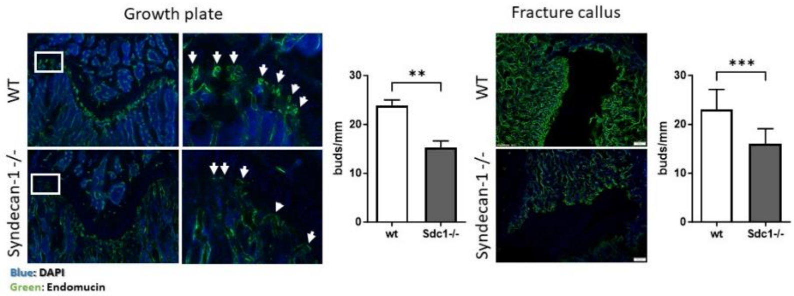

Figure: Neoangiogenesis at the chondro-osseous border of the femur growth plate or in the fracture callus of femur fractures. Syndecan-1 deficient and WT mice were sacrificed at the age of 14 weeks, after 14days of fracture healing (femur midshaft fracture stabilized with an intramedullary nail). Vessel buds were stained using anti-Endomucin-antibody in green (DAPI as nucleus staining) and quantified as buds/mm of the chondro-osseous borderline. Syndecan-1 deficient mice showed decreased number of vessel buds pointing towards an impaired neoangiogenesis

**P044**

**Improvement of matrix dependent strength features of tibia in diabetic rats with repeated fracture after oral calcium intake**

*Nadezhda Mosyagina*[1], *Dmitrii Astrakhantsev*[1]
*[1]FSBEI HI ST. LUKA LSMU of MOH of Russia, Human anatomy department, Lugansk, Russia C.I.S.*

**Abstract Text**

**Introduction:** Fracture healing in diabetes is affected by numerous factors including poor calcium turnover and impaired matrix synthesis. Calcium replenishment is always required for fracture treatment although strength of bone matrix itself is not properly studied. Therefore our study is aimed at testing of bone matrix strength features in diabetic rats with repeated fracture after administration of oral calcium drugs.

**Purpose:** To test the improvement of matrix dependent strength features of tibia in diabetic rats with repeated fracture after oral calcium intake

**Methods:** 60 female rats were separated into three groups. Group 1 comprised intact animals, groups 2 and 3 include animals with hyperglycemia and obesity caused by adipogenic diet. Fractures in these animals were modeled as 2 mm holes in femurs and tibiae. Animals of the group 3 received CA. Strength testing of the tibiae employed three-point bending technique. Strength features obtained were Young's modulus (YM) and specific sag (SS).

**Results:** In diabetic animals in comparison with intact animals YM decreased in all observation terms and SS increased insignificantly. In animals of group 3 SS in comparison with those of the group 2 increased by the 30$^{th}$ day – by 9.24%. YM in these animals increased by the 15$^{th}$ and the 30$^{th}$ days – by 24.28% and 26.69% ($p \leq 0.05$ in all cases). This may testify for improvement of bone matrix synthesis.

**Conclusions:** After repeated fracture in diabetic animals YM decreases and SS insignificantly increases. These changes testify for increased bone fragility possibly coming from deranged bone matrix synthesis. In animals that received CA SS changed in the same way as in untreated animals.

YM on the contrary increased, which is the sign of improvement of bone tissue quality.

**P045**

**The direct healthcare costs of fractures in T1D**

*Annika Kvist[1], Morten Frost[2], Andrea M. Burden[3], Peter Vestergaard[1], Troels Kristensen[4]*
*[1]Aalborg University Hospital, Steno Diabetes Centre North Denmark, Aalborg, Denmark ; [2]Molecular Endocrinology Stem Cell Research Unit KMEB, Endocrinology and Metabolism, Odense, Denmark ; [3]Pharmaceutical Sciences, Chemistry and Applied Biosciences, Zurich, Switzerland ; [4]Public Health, DaCHE, Odense, Denmark*

**Abstract Text**

**Background:** Patients with type 1 diabetes (T1D) have an increased risk of sustaining a fracture, and post-fracture complications are higher in T1D compared to persons without diabetes. It is largely unknown how bone health and fracture-related complications affects the cost of fractures in T1D.

**Aim:** This study aims to estimate the direct healthcare cost of fractures in T1D compared to controls.

**Methods:** This study analyses data about patients aged 18 years or above in Denmark with a hip, humerus, forearm, foot, or ankle fracture from 2011 to 2015. Patients with T1D were identified using ICD-10 codes and prescription medication. The direct healthcare costs were calculated within one year after the fracture. The treatment group of patients with T1D were matched with non-diabetic individuals based on fracture site, sex, age, and CCI using coarsened exact matching.

**Results:** In this study 302, 203, 346, 290, and 149 cases of hip, humerus, forearm, foot, and ankle fractures were matched with controls. The direct healthcare cost within one year after fracture was significantly higher among patients with T1D compared to controls for humerus (€1,689 vs. €810, p=0.02), forearm (€842 vs. €575, p=0.002), foot (€402 vs. €320, p=0.007), and ankle fractures (€1,395 vs. €806, p=0.03). By contrast, the direct healthcare cost of hip fractures was similar in patients with T1D and controls (€10,483 vs. €10,795, p=0.37).

**Conclusion:** The direct healthcare cost of humerus, forearm, foot, and ankle fracture treatment was higher among individuals with T1D, although no difference was identified for hip fractures between T1D and controls. Understanding the differences in healthcare costs of fractures among T1D and controls is necessary to address optimization of care and prevention of complications, which could lead to lower treatment costs.

**P046**

**Immunomodulatory functions of mesenchymal stromal cells correlate with fracture healing outcome**

*Katrin Übelmesser[1], Eva Kupczyk[2], Sebastian Häusner[1], Drenka Trivanovic[3], Stefanie Hölscher-Doht[2], Marietta Herrmann[1]*
*[1]University Hospital Wuerzburg, IZKF Group Tissue Regeneration in Musculoskeletal Diseases, Wuerzburg, Germany ; [2]University Hospital Wuerzburg, Deartment of Trauma- Hand- Plastic and*

*Reconstructive Surgery, Wuerzburg, Germany ; [3]University of Belgrade, Group for Hematology and Stem Cells- Institute for Medical Research, Belgrade, Serbia*

**Abstract Text**

**Introduction:** The initial formation of a platelet-rich fracture haematoma and the coordination of the inflammatory response is a critical step in fracture healing. Bone marrow mesenchymal stromal cells (MSCs) are known for their immunoregulatory properties.

**Purpose:** Here, we investigated a possible correlation between the healing outcome and the immunomodulatory properties of MSCs.

**Methods**: MSCs were isolated from bone marrow-rich drilled dust of the fracture region of nine patients undergoing intramedullary nailing of femoral shaft fractures with informed consent and ethical approval. Routine X-ray of the femur after six month was used to assess the healing outcome. MSCs were expanded following standard protocols and stimulated with INF- γ and TNF- α to enhance their immunomodulatory properties. Viability was assessed using the MTT assay. Expression of immunomodulatory genes (VCAM1, IDO1, IL-6) was tested by qPCR. Functional immunomodulatory properties of MSCs were analysed by flow cytometry based on changes in proliferation and phenotype of activated lymphocytes after five days of co-culture with MSCs.

**Results**: Stimulation with INF-γ and TNF-α showed no significant effect on the viability of MSCs. MSCs from patients with impaired healing were not able to inhibit lymphocyte proliferation in co-culture (124.54±36.03% proliferating cells compared to lymphocyte single cultures), which was in contrast to MSC from patients with normal healing (76.58±14.30%, $p = 0.034$). The relative proportion of regulatory T cells was slightly lower in co-cultures with MSCs from the impaired healing group. Pretreatment with INF-γ and TNF-α improved the immunosuppressive effects in the impaired healing group.

**Conclusions:** Our results provide initial evidence that MSCs from patients with impaired fracture healing have reduced immunomodulatory capabilities compared to MSCs from patients with normal healing. These immunoregulatory properties of MSCs could be enhanced by cytokines, which needs to be further investigated as part of a potential therapeutic approach.

**P047**

**Impact of BMP-1 modulation on early fracture repair**

*<u>Daniel Kronenberg</u>[1], Jens Everding[2], Catherine Moali[3], Sandrine Vadon-LeGoff[3], Melanie Timmen[1], Richard Stange[1]*
*[1]University of Muenster, Department of Regenerative Musculoskeletal Medicine- Institute of Musculoskeletal Medicine IMM, Muenster, Germany ; [2]University Hospital Münster, Department of Trauma-- Hand-- and Reconstructive Surgery, Muenster, Germany ; [3]CNRS - University of Lyon 1, Tissue Biology and Therapeutic Engineering Unit- UMR 5305, Lyon, France*

**Abstract Text**

**Objective:** BMP-1 is the major procollagen-C-peptidase activating fibrillary collagens type I-III as well as several enzymes and growth factors involved in the generation of extracellular matrix in bone. This study investigated the effect of adding and inhibiting BMP1 directly post fracture with the aim to accelerate fracture repair by increasing the availability of mature collagen and osteoblastic growth factors.

**Methods**: Standardised murine femoral shaft fractures were stabilised by an intramedullary nail in 12 week old female C57Bl/6J mice (animal experiment licence 84-02.04.2015.A035). We injected either 20 µL BMP-1, activity buffer or the BMP-1 inhibitor "sizzled". After 7, 14 and 28 days mice are sacrificed. Femurs were dissected and paraffin slides were prepared. Callus composition was divided into soft tissue, mineralised and cartilaginous callus. Murine primary isolated pre-osteoblastic cells were kept in culture 40 ng/ml BMP-1 and 50ng/ml sizzled was added under serum-free conditions. Pro-osteogenic BMP-2 and anti-osteogenic myostatin protein quantity was determined using in-cell western (Li-Cor) and normalized to total cell number(cell tag) control signal.

**Results:** Adding BMP-1 displayed only a weak osteogenic effect on the outcome. After 7 days more mineralised callus was present and afterwards the cartilaginous callus was apparently remodelled at higher rate. For inhibition of BMP-1, we observed more cartilaginous callus, which may indicate reduced stability. In cell culture we could detect increased myostatin and decreased BMP-2 deposition on pre-osteoblast under BMP-1 addition a slightly increased BMP-2 and decreased myostatin deposition.

**Conclusion:** Interfering BMP-1 homeostasis at this early stage of fracture repair seemed to have no severe effects on the healing outcome. Inhibition yields apparently lower callus quality, addition of BMP-1 did not accelerate the healing outcome significantly. Cell culture experiments showed that BMP-1 application decreased Myostatin and increased pro-osteogenic BMP-2 deposition, for BMP-1 inhibition this finding was reversed. Further experiments to elucidate involved BMP-1 substrates are ongoing.

**P048**

**Nacre organic compounds stimulate endochondral ossification**

*Sarah Nahle[1], Capucine Jourdain de Muizon[2,3], Ganggang Zhang[4], Solène Viallon[1], Arnaud Bianchi[5], Ali Al-Mourabit[2], Marthe Rousseau[1,6]*
*[1]Université Jean Monnet Saint-Étienne- INSERM- Mines Saint Etienne- SAINBIOSE U1059, Campus Santé- faculté de médecine, Saint-Etienne, France ; [2]CNRS- Institut de Chimie des Substances Naturelles, Université Paris-Saclay, Gif-sur-Yvette, France ; [3]CNRS- ISCR-UMR 6226- Ecole Nationale Supérieure de Chimie de Rennes, Université de Rennes, Rennes, France ; [4]The First Affiliated Hospital of Zhengzhou University, Department of Orthopedics, Zhengzhou, China ; [5]UMR 7365 CNRS Université de Lorraine, Ingénierie Moléculaire et Physiopathologie Articulaire IMoPA, Vandœuvre-lès-Nancy, France ; [6]UMR5510 MATEIS CNRS/ Lyon University, Lyon University/INSA-Lyon, Lyon, France*

**Abstract Text**

Nacre organic matrix has gained huge attention for its effect on bone, especially in promoting intramembranous ossification. Herein, we aimed to extend the understanding of the osteogenic potential of this matrix by investigating its influence on endochondral ossification using a micromass 3D-culture model of the pre-chondrogenic ATDC5 cell line. Our results indicated that the nacre ethanol-soluble matrix (ESM) stimulates the differentiation and the mineralization of the pre-chondrocytes. Mechanistically, the ESM stimulates the phosphorylation of Akt, p38, JNK, GSK-3, and CREB proteins at the early stages of differentiation and p70S6, TOR, and GSK-3β in the later stages. Knowing that these pathways are linked to the endochondral ossification process, these data suggest that the ESM could be promising in bone-related diseases. The analysis of ESM composition unveiled the presence of osteogenic peptides. Using a cell-based screening model representing Collagen X (Col X) transcriptional activity, we identified a novel small nacre-derived-peptide TY6, as an efficient up-regulator of chondrogenesis. TY6 increased the expression of hypertrophic chondrogenic marker genes as well as the mineralization of the extracellular matrix in ATDC5 cells. Overall, our results suggest that TY6 deserves further in vitro and in vivo studies to uncover its mechanisms of action on the first step of endochondral ossification.

**P049**

**Clinical and molecular genetic predictors of bone formation in young people**

*Karin Akhiiarova*[1], *Anton Tyurin*[1], *Khusainova Rita*[2]
[1]*Bashkir State Medical University, Department of Internal Medicine, Ufa, Russia C.I.S. ;* [2]*Bashkir State Medical University- Department of Medical Genetics, The National Medical Research Center for Endocrinology- Laboratory of Genomic Medicine, Ufa- Moscow, Russia C.I.S.*

**Abstract Text**

**Introduction:** The maximum amount of bone mass at a young age forms the peak bone mass (PBM), which gradually decreases after the plateau period. Suboptimal values of PBM lead to the development of osteopenia and early osteoporosis.

**Purpose of the study:** was to evaluate clinical and molecular genetic factors that can have a negative impact on aBMD (bone mineral density) in young people.

**Methods:** A single-stage noninvasive study was performed (N=84), the average age was 26.04±1.34 years. aBMD was evaluated by X-ray absorptiometry with determination of absolute values and Z-criteria and body composition, joint hypermobility (JH) by the Beighton score (1998). A molecular genetic study of the SNP gene loci was performed: *ADAMTS5 rs9978597* and *rs229069*, *TRPM6 rs3824347*, *BMP5 rs3734444*. Quantitative features were compared by using the Mann-Whitney U-test or the Student's t-test, qualitative features were compared by using the Fisher criterion ($\chi 2$) with an estimate (OR) at $p<0.05$. The correction for plurality was made by Benjamin-Hochberg (FDR).

**Results:** At the first stage, the participants were divided into two groups: with fragility fractures N= 14 and control (N=40). Groups were compared on aBMD, no statistically significant differences were found. With a comparable body mass index in group 1, the amount of adipose tissue in absolute and percentage terms was statistically significantly less (15.66±1.74kg vs. 20.10±0.76 kg, p=0.013), and lean weight prevailed only in percentage terms (70.16±1.75% vs. 65.43±0.86%, p=0.015). At the second stage, no associations of JH, SNP *ADAMTS5*, *TRPM6, BMP5*, body composition with aBMD were detected either in absolute value or in the values of the Z-criterion.

**Conclusions:** The distribution of fat and lean masses can affect the accumulation of bone tissue at a young age, in particular, adipose tissue can play a protective role with respect to fractures at a young age.

**P050**

**A 2'-Fucosyllactose + B infantis synbiotic improves the effect of vitamins K2, A, and D on bone quality and strength.**

*Michael Baruchet[1], Florac De Bruyn[2], Nancy Frank[3], Alessandro Turcato[4], Esther Campos-Gimenez[3], Yves Henrotin[5], Marie Noelle Horcajada[1], Nicolas Bonnet[1]*
*[1]Nestlé Institute of Health Sciences- Nestlé Research- EPFL innovation Park, Physical health, Lausanne, Switzerland ; [2]Société des Produits Nestlé S.A.- Nestlé Research & Development, Product Technology & Development, Konolfingen, Switzerland ; [3]Nestlé Institute of Health Sciences- Nestlé Research- EPFL innovation Park, Analytical science, lausanne, Switzerland ; [4]Nestlé Institute of Health Sciences- Nestlé Research- EPFL innovation Park, Manager, lausanne, Switzerland ; [5]Center for Interdisciplinary Research on Medicines CIRM, Pathology, Liege, Belgium*

**Abstract Text**

Vitamins K2, A and D (K2AD) are key for calcium metabolism. We previously reported that human milk oligosaccharides (HMO) supplementation stimulates bone formation through microbial metabolites production. We hypothesized that a specific synbiotic, 2'-Fucosyllactose (2FL)+*B infantis* combined with vitamins K2AD could maximize osteoblastogenesis and lead to improved bone quality and strength during growth. To determine the impact of the symbiotic on microbial metabolites, we performed an *in vitro* fermentation with infant feces and measured SCFA and vitamin K2 production We cultured MC3T3 cells for 7 days with vitamins K2AD +/- SCFAs (obtained after fermentation) and monitored ALP. In addition, growing mice aged of 18 days received, standard diet (placebo) supplemented with vitamins K2AD or with vitamins K2AD+[2FL+*B infantis*] for 4 weeks. Femoral cartilage growth plate, microstructure and strength (3 points bending / axial compression) were evaluated. 2FL+*B infantis* increased vitamin K2 and total SCFAs levels (127% & 200% vs blank; 121% & 149% vs 2FL; and 114% & 163% vs *B infantis;* p<0.01). MC3T3 cultured with vitamins K2AD +/-SCFAs exhibited an increase of ALP by 146% and 216% vs control, p<0.01. *In vivo*, vitamins K2AD+[2FL+B infantis] increased maturing chondrocytes and decreased apoptotic areas (+23% & -8.8% vs placebo, p<0.01). It also increased trabecular connection, cortical vBMD (+24% & +1% vs placebo, p<0.05) whereas vitamins K2AD only significantly increase cortical vBMD (+1% vs placebo, p<0.05). Accordingly, vitamins K2AD+[2FL+B infantis] increased both bending and compression strength, in contrast vitamins K2AD alone increased only bending strength. In conclusion, we demonstrated that a [2FL + *B infantis*] synbiotic increases the production of K2 & SCFAs compared to HMO or probiotic alone with a significant impact on osteoblastogenesis. Hence, it strengthened the effect of vitamins K2AD on bone growth and quality. These findings highlight the potential of synbiotics in early life for bone development.

**P051**

**Exploring the Nexus of Postural Control in Diabetes: A Cross-Sectional Investigation of New Strategies for Fracture Preventions.**

*Asger Krohn Jespersen1[1], Aksayan Arunanthy Mahalingasivam1[2], Niels Ejskjær[3], Dan Dupont Hougaard[4], Peter Vestergaard[2], Johan Møller Røikjer[3], Nicklas Rasmussen[5]*
*[1]Aalborg University Hospital, Department of Clinical Medicine- Faculty of Medicine- Aalborg University- Aalborg- Denmark, Aalborg, Denmark ; [2]Aalborg University Hospital- Denmark, Department of Clinical Medicine- Faculty of Medicine- Aalborg University- Aalborg- Denmark, aalborg, Denmark ; [3]Aalborg University Hospital- Denmark and Steno Diabetes Center, Department of Clinical Medicine- Faculty of Medicine- Aalborg University- Aalborg- Denmark, Aalborg, Denmark ; [4]Aalborg University Hospital- Denmark, Balance & Dizziness Centre- Department of Otolaryngology-Head & Neck Surgery and Audiology- Aalborg University Hospital- Aalborg- Denmark, Aalborg, Denmark ; [5]Aalborg University Hospital- Denmark, Steno Diabetes Center North Denmark, Aalborg, Denmark*

**Abstract Text**

**Aim and introduction**: In individuals with type 1 diabetes (T1D) and type 2 diabetes (T2D), impaired postural control poses an elevated risk of falls and fractures, with neuropathy potentially contributing. This study employs Computerized Dynamic Posturography (CDP) to assess the impact of diabetes and diabetic neuropathy on postural control.

**Results:** Participants with T1D and T2D exhibited significantly poorer performance in the majority of SOT parameters (T1D: 9 of 11, T2D: 8 of 11, $p < 0.05$), with lower vestibular ratios in both diabetes types. Visual ratios were reduced only in T1D. T2D participants swayed more in the dynamic VR environment compared to eyes closed. Equilibrium scores were notably lower in dynamic surface conditions, indicating challenges in integrating somatosensory information for balance control.
MCT results revealed deteriorated performance in both diabetes types (T1D: 5 of 7, T2D: 5 of 7, $p < 0.05$), with significantly higher composite translation latency scores in forward, backward, and mean directions. This suggests slower reaction times and an increased risk of falls. The presence of neuropathy had limited influence on performance, except for one SOT measurement and MCT forward medium translation, where participants with T1D without neuropathy outperformed those with T2D with neuropathy."

**Methods:** In a cross-sectional study with 94 participants (T1D: n=49, T2D: n=45), matched with healthy controls, postural control was evaluated through CDP using Sensory Organization Test (SOT) protocols and Motor Control Test (MCT). Diabetic neuropathy was assessed through sural nerve conduction and hand grip strength. Statistical analyses included Kruskal-Wallis H tests, Bonferroni-corrected Mann-Whitney U tests, and Chi-squared tests, with correlations examined using Spearman rank correlation.

**In conclusion,** diabetes compromises postural control, while diabetic neuropathy's impact is limited. CDP emerges as a valuable tool for exploring factors influencing postural control, with implications for fall and fracture prevention in diabetes.

**P052**

**Functional outcomes six months after fragility hip fracture**

*<u>Inês Genrinho</u>[1,2], Catia Machado[3], Susana P Silva[1], Sofia F. Azevedo[1], Oliveira Cláudia[1], Vilafanha Carolina[1], Pedro M. Teixeira[1], Nuno Pais[3], Anabela Barcelos[1]*
*[1]Centro Hospitalar Baixo Vouga, Rheumatology, Aveiro, Portugal ; [2]Centro Hospitalar Tondela Viseu, Rheumatology, Viseu, Portugal ; [3]Centro Hospitalar Baixo Vouga, Orthopaedic, Aveiro, Portugal*

**Abstract Text**

**Background**: Hip fragility fractures represent a significant health concern and often lead to a substantial decline in activities of daily living (ADLs), walking capacity, and functional status.

**Purpose**: To determine the functional outcomes after the treatment of hip fracture.

**Methods:** A retrospective study involved patients $^{3}$ 50 years old with fragility hip fractures admitted to our Fracture Liaison Service between 2019 and 2023. Katz score of Independence in ADLs was used to determine the dependence status before (Group A) and 6 months after (Group B) the hip fracture. Demographic, type of hip fracture, length of stay (LOS), walking aids, and mortality data were collected. Data was analysed using SPSS.

**Results:** A total of 327 hip fracture patients were included. The mean age was 81.0 (SD 9.2) years old and 83.8% were women. 29.1% had previous fragility fractures. The most frequent hip fracture was the intertrochanteric (51.4%), followed by the neck (36.1%) and subtrochanteric (11.9%). The mean LOS was 8.5 (SD 5.2) days. In Group A, 74.3% of the patients were autonomous, 20.5% and 4.9% were partially and dependent on ADLs respectively. In Group B, patients were partially or dependent in 25.4% and 19%, respectively. 51.1% maintained their previous autonomy level. 24.8% presented autonomous gait, while 71.3% needed any type of walking aid (24.2% walking frames, 21.7% walking sticks, 19.6% wheelchairs, and 5.5% crutches).

The in-hospital mortality rate was 0.6% and the mortality rate at 6 months was 3.7%. In Group B, Katz's score was lower ($p < 0.0001$), had a higher dependence status associated with the female gender ($p<0.008$), older age ($p<0.001$), previous fragility fractures ($p<0.028$), longer LOS ($p<0.016$) and type of fracture ($p<0.035$).

**Conclusion:** 27.5% of fragility hip fracture patients presented a substantial decrease in Katz score, being more dependent on ADLs 6 months after fracture.

**P053**

**Nf1 loss in skeletal progenitors induces cellular senescence and an inhibitory bystander effect on the osteogenic differentiation of normal osteoprogenitors**

*Florent Elefteriou*[1], *Efrosini Cuko*[2]
[1]*Baylor College of Medicine, Molecular and Human Genetics- Orthopedic Surgery, Houston, United States ;* [2]*Baylor College of Medicine, Orthopedic Surgery, Houston, United States*

**Abstract Text**

Neurofibromatosis type I (NF1) is a common genetic disorder that arises from mutations in *NF1*, a gene encoding a negative regulator of RAS signaling. Although cutaneous/plexiform neurofibromas are common in patients with NF1, musculoskeletal conditions can also be found. NF1 congenital pseudarthrosis is a rare but challenging skeletal manifestation in children with NF1, often presenting as unilateral tibia bowing, followed by fracture and recalcitrant healing. Previous research demonstrated that *Nf1* inactivation in bone marrow stromal cells (BMSCs) impairs their osteogenic differentiation and mineralization. We serendipitously observed that $Nf1^{Osx\text{-}creTetOff}$-null BMSCs were large and flat, and found in these cultures a higher proportion of Senescence-Associated beta-galactosidase-positive cells (+60%), γH2AX foci (x2), cells arrested in G2/M (x2.5) and higher expression of the cell cycle inhibitor *p16Ink4a* (+ 60%, $p<0.05$ for all outcomes). Most importantly, the conditioned medium (CM) of *Nf1* cKO BMSCs inhibited the osteogenic differentiation of WT BMSC cultures, as determined by the expression of *Alpl* and *Ibsp*, and treatment of CM-producing cells with the JAK inhibitor Ruxolitinib inhibited this effect ($p<0.05$). As observed *in vitro* in cKO BMSCs, we detected a higher expression of *p16Ink4a* in the bones of $Nf1^{Osx\text{-}creTetOff}$ cKO mice (2x, $p<0.05$), whereas p21 expression was normal. These results suggest that senescence in *Nf1*-deficient BMSCs is a protective mechanism against transformation and propagation promoted by uncontrolled RAS activation, that *Nf1* cKO BMSCs produce a senescence associated secretory phenotype (SASP) whose components have an inhibitory bystander effect on normal osteoblast progenitors of the bone microenvironment, and open the possibility of using senolytics, a class of drug that sensitize senescent cells to apoptosis, to rid *Nf1*-deficient osteoprogenitors from the skeleton of patients with NF1 and prevent the formation of dysplastic bones and/or promote bone union after fracture.

**P054**

**CXXC Finger Protein 1 is Required for Osteoblast Differentiation**

*Ahmed Al Saedi*[1,2], *Joshua Mayers*[3], *Vicki Rosen*[4], *Diana Carlone*[2,5]
[1]*Boston children's Hospital, Department of Endorinology, Boston, United States ;* [2]*Harvard Medical School, Pediatrics, Boston, United States ;* [3]*Boston Children's Hospital, Endocrinology, Boston, United States ;* [4]*Harvard Dental School, Biology, Boston, United States ;* [5]*Boston Children's Hospital, Endorinology, Boston, United States*

**Abstract Text**

Skeletal development, remodeling, and regeneration are intricately tied to the activity of mesenchymal progenitor cells (MPCs). Recent evidence highlights the role of epigenetics in regulating MPC function, impacting bone formation in both mice and humans. Despite this, the precise mechanisms through which epigenetics, particularly the action of CXXC Finger Protein 1 (CFP1), governs MPC activity and their involvement in bone formation remain unclear. CFP1, identified as an epigenetic regulatory factor facilitating H3-Lys4 trimethylation for transcriptional activation, has shown regulatory roles in progenitor cell differentiation in various systems. However, its specific function in bone formation has yet to be defined. To address this gap, we used *Prx1*-Cre to delete *Cxxc1*, the gene encoding CFP1, in MPCs ($cKO^{Prx1}$). This deletion led to a disruption in chondrocyte differentiation, maturation, and primary ossification, indicating CFP1's critical role in endochondral ossification. Moreover, mutant mice exhibited reduced intramembranous ossification within the calvaria, suggesting CFP1's direct influence on osteoblast differentiation. Further investigations using adenoviral deletion of *Cxxc1* in bone marrow stromal cells (BMSCs) and quantitative RT-PCR analysis demonstrated a significant decrease in the expression of osteoblast markers (*Runx2*, *Osx*, *Col1a1*, *Ocn*), underscoring CFP1's involvement in bone formation. To delve deeper into its role in osteoblast differentiation, CFP1 knockout MC3T3-E1 and W20 cells were generated using CRISPR/Cas9. Consistent with our earlier findings, mutant cells fail to properly differentiate. Preliminary studies link CFP1 to several signaling pathways including BMP implicating a possible mechanism by which this epigenetic factor controls osteoblast differentiation. Additionally, *Osx1*-Cre ($cKO^{Osx1}$) was utilized in mice. In summary, our recent findings establish a connection between CFP1 and both endochondral and intramembranous bone formation. This research sheds light on the crucial regulatory role of CFP1 in MPCs, emphasizing its impact on skeletal development and osteoblast differentiation.

**ND-P011**

**BMP3 affects chondroblast-to-osteoblast transition in ectopic bone**

*Viktorija Rumenović*[1], *Slobodan Vukičević*[1], *Igor Erjavec*[1]
[1]*School of Medicine- University of Zagreb, Department of Anatomy- Laboratory for Mineralized Tissue, Zagreb, Croatia*

**Abstract Text**

**Introduction:** BMP3 is a member of the bone morphogenetic family of proteins that has been shown to act as an antagonist to other BMPs. The skeletal effects of BMP3 knock-out have been widely studied, while the underlying mechanism and the effect on cartilage and the transition to bone have not been explored.

**Purpose:** Aims of this research are to explore the effect of removal of BMP3 on bone remodeling and formation processes.

**Methods:** For *in vitro*, the effect of BMP3 was studied in MC3T3 cell line. For *in vivo* the gene expression analysis of Bmp3$^{-/-}$ mice was done using RNAseq. Using FACS osteoprogenitor markers were characterized in bone marrow stromal cells (BMSC). Ectopic bone assay was used to assess *de novo* bone formation. All experiments were approved by institutional and national Ethics comitee.

**Results:** In MC3T3-E1 preosteoblast cell line displayed a dose-dependent inhibitory effect of BMP3 on osteogenic potential of BMP6. Gene expression analysis found 5 genes differing significantly. Bmp3$^{-/-}$ mice displayed higher count of skeletal stem cells and a deficit in cartilage. During BMSC differentiation, BMP3 added to cells from WT mice resulted in a 10-fold decrease of alkaline phosphatase production. Autologous blood coagulum with BMP6 implanted in axillary region showed increased ectopic bone formation in *Bmp3*$^{-/-}$ mice. Prevalent bone phenotype in ectopic bone was observed in *Bmp3*$^{-/-}$, as opposed to a more cartilaginous phenotype in WT mice.

**Conclusion:** These findings further corroborate that BMP3 mediates differentiation and maturation of bone tissue. Interestingly, BMP3 potentially plays a pivotal role in the transition from chondrogenic to osteogenic phenotype during ectopic bone formation.

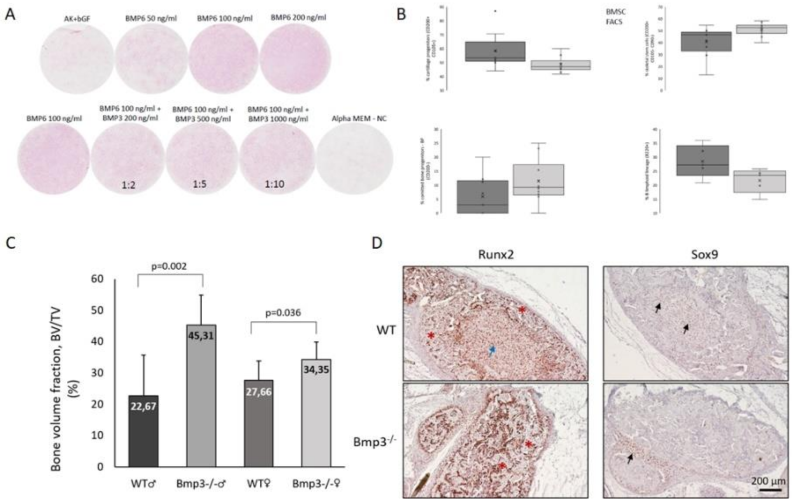

**Figure1.** BMP3 inhibition of BMP6 osteogenic effect on MC3T3 cells (A). Bone marrow stromal cells (BMSC) characterization by Flow cytometry (B). Ectopic bone volume analysis by micro-CT (C). Phenotype of ectopic bone analyzed by immunohistochemistry (D).

### Poster Sessions: Cancer and bone

### P055

### Irisin prevents trabecular bone loss in a mouse model of Multiple Myeloma

*Roberta Zerlotin*[1], *Graziana Colaianni*[1], *Patrizia Pignataro*[2], *Clelia Suriano*[1], *Manuela Dicarlo*[1], *Silvia Colucci*[2], *Angela Oranger*[1], *Maria Grano*[1]
*[1]University of Bari, Department of Precision and Regenerative Medicine and Ionian Area - DiMePRe-J, Bari, Italy ; [2]University of Bari, Department of Translational Biomedicine and Neuroscience - DiBraiN, Bari, Italy*

**Abstract Text**

**Background:** Multiple myeloma-associated bone disease (MMBD) is characterized by osteolytic lesions and pathologic fractures, which remain a therapeutic priority despite new drugs improving the survival of patients with MM. Antiresorptive molecules, such as bisphosphonates and denosumab, are the main option for treating MMBD, although they are only partially effective in repairing prior bone lesions.

**Purpose:** The search for pharmacological agents for the treatment of MMBD is currently focused on the study of osteoanabolic molecules. Among them, we investigated the effects of myokine irisin, which has been shown to increase bone mass in healthy mice, prevent bone loss in mouse models of disuse-induced osteoporosis, and accelerate fracture healing.

**Methods:** Ex-vivo MicroCT and flow cytometry analyses were used to evaluate the effect of irisin treatment in a mouse model of induced multiple myeloma (MM) (C57BL/KaLwRij mice) by intratibial injection of green, fluorescent tumor cells (5TGM1-eGFP cells), followed by intraperitoneal administration of vehicle or 100μg/Kg/weekly of rec-irisin for 6 weeks (approved experimental protocol 12-2022-PR).

**Results:** Micro-CT analysis showed that irisin treatment ameliorates MM-induced trabecular bone damage by preventing the reduction of femoral trabecular bone volume (Tb. BV/TV, p=0.0141) and trabecular number (Tb. N, p=0.034), as well as by improving fractal dimension, an indicator of proper trabecular microarchitecture (Tb. FD, p=0.0008). We also showed that irisin downregulates the expression of sclerostin, a known inhibitor of bone formation. In parallel, by flow cytometry, we found that MM cell invasion was significantly decreased in the femoral bone marrow of irisin-treated mice, consistent with the significantly reduced viability of MM cells detected after 48 hours of stimulation with 200 ng/ml and 500 ng/ml and after 72 hours already with 100 ng/ml of irisin.

**Conclusions:** Although further studies are needed, our results suggest that irisin may be a promising new strategy to counteract MMBD and tumor burden.

**P056**

**Characterization of Key Cells in the Bone Microenvironment of Metastatic Breast Cancer Patients.**

*Melisa K. Uguz[1], Emma K.E. Bengtsson[1], Malene H. Nielsen[1], Bilal El-Masri[1], Thomas L. Andersen[1], Anne Marie Bak Jylling[2], Christina Andreasen[1]*
*[1]University of Southern Denmark, Molecular Bone Histology lab- Research Unit of Pathology, Odense, Denmark ; [2]Odense University Hospital, Clinical Pathology, Odense, Denmark*

**Abstract Text**

**Background:** Bone is one of the major targets for breast cancer metastases and the interaction between cancer cells and stromal cells is crucial for establishment of the tumor supportive microenvironment.

**Purpose:** To quantify the density of key cells and blood vessels near the bone surface in biopsies from metastatic breast cancer patients (MBC) in relation to remodeling activity.

**Methods:** Bone marrow biopsies from 10 MBC-treated (MBC-T: radiation + chemotherapy, zoledronic acid, aged 51–92) and 10 MBC-untreated (MBC-U: newly diagnosed or cancer free >2 years, aged 38–87) were included. Bone remodeling activity was assessed on Masson Trichrome stained sections, while neighboring sections were multiplex immunofluorescent stained for CK7/19, TRAcP, perilipin, CD34, and DAPI to identify cancer cells, osteoclasts, adipocytes, blood vessels, and nuclei, respectively. Densities were quantified in a zone of 0–100 µm from the bone surface using HALO software.

**Results:** MBC-U patients had higher bone volume ($p<0.025$), but fewer cancer cells ($p<0.047$) than MBC-T. Both groups had more eroded than quiescent bone surfaces (MBC-T: $p<0.02$; MBC-U: $p<0.002$), with no evidence of bone formation. MBC-T patients had more cancer cells above eroded surfaces than MBC-U ($p<0.005$), mainly in the 25–100 µm marrow zone. Blood vessel density was higher in MBC-U near the bone surface (0–25 µm), but similar in the 25–100 µm zone irrespectively of remodeling stage. MBC-U patients tended to have more adipocytes, however not significantly different from MBC-T. Both groups had osteoclasts at the bone surface (0–25 µm), with no significant difference. Notably, some patients had a high osteoclast density in the marrow distant from bone surfaces.

**Conclusion:** Despite anti-resorptive therapy, MBC-T patients had more eroded surfaces and cancer cells than MBC-U patients. Furthermore, ongoing bone formation was absent in all patients. Interestingly, a subset of the treated MBC patients displayed a significant number of osteoclasts, even distant from bone surfaces.

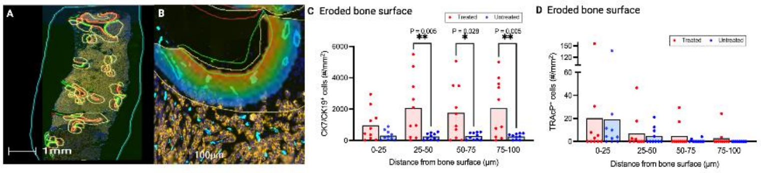

**P057**

**Head and Neck Cancer induces bone deterioration differently in young and mature mice**

*Alexander Jones[1], Joshua R. Huot[2], Michael G. Moore[1], Lynda F. Bonewald[3], Fabrizio Pin[4]*
*[1]Indiana University- School of Medicine, Otolaryngology-Head & Neck Surgery, Indianapolis, United States ; [2]Indiana University- School of Medicine, Anatomy- Cell Biology & Physiology, Indianapolis, United States ; [3]Indiana University- Indiana Center for Musculoskeletal Health, Anatomy- Cell Biology & Physiology, Indianapolis, United States ; [4]Indiana University- School of Medicine, Anatomy- Cell Biology and Physiology, Indianapolis, United States*

**Abstract Text**

It is estimated that 660,000 people will be diagnosed with Head and Neck Cancer (HNC) worldwide in 2023. Notably, 40% of HNC patients will develop debilitant musculoskeletal alterations that reduce QoL and survival. Bone deterioration can occur in HNC patients in the absence of bone metastasis. Unfortunately, the mechanism(s) and the consequence of these bone alterations are unknown. This study is aimed to elucidate the bone deterioration in a model of non-bone metastatic HNC.

C57BL/6J male mice (2-and-13-month-old) were subcutaneously injected with 3×106 MLM3 and MLM5 HNC cells. Only the MLM3 growth caused severe loss of trabecular and cortical bone as suggested by the reduction of the Tb.BV/TV, Tb.Th, Tb.N. and Ct.BV/TV in both age. The increased bone resorption was suggested by the high levels of circulating CTX-I detected in the MLM3 bearers. The assessment of mechanical properties of the femur reveled that HNC bearers have weaker bones. The measurement of the tibia length and the proximal growth plate suggest that bone loss in young HNC mice is also due to growth failure. We then measure the circulating levels of pro-resorptive factors. High circulation IL-6 was detected in all groups, TNFα instead was found elevated in the old mice only. RANKL was unchanged whereas OPG was surprisingly elevated in all HNC bearers. As suggested by the analysis of the MLM3&5 conditioned media, HNC cells can produce OPG. Further analysis reveals an unexpected negative correlation between OPG levels and body weight, muscle mass and Tb.BV/TV.

Our study shows that non-bone metastatic HNC has severe and age-dependent detrimental effects on bone. Further studies will be performed to understand the inability of OPG to counteract bone loss in an HNC setting. Co-treatments that maintain bone health should be used in non-bone metastatic HNC patients.

**P058**

**Vitamin D regulates extracellular vesicle communication of bone metastatic prostate cancer cells**

*Joelle A Z Klazen[1], Iris J Robbesom - van den Berge[1], Martin E Van Royen[2], Andre J Van Wijnen[3], Johannes P T M Van Leeuwen[1], Marjolein Van Driel[1]*
*[1]Erasmus MC, Internal medicine, Rotterdam, Netherlands ; [2]Erasmus MC, Pathology, Rotterdam, Netherlands ; [3]University of Vermont, Biochemistry, Burlington, United States*

**Abstract Text**

Bone is a preferential site for metastatic invasion by cancer cells. To unravel underlying mechanisms, we studied the role of extracellular vesicles (EVs). EVs are nano-sized particles that transfer information from cell to cell and thereby support pre-metastatic niche formation. This makes EVs an interesting target for new therapies. As a bone anabolic therapeutic also known for its inhibitory effects on cancer cell growth, we studied vitamin D, aiming to unravel its role in human bone metastatic cancer EV communication with osteoblasts.

EVs from vitamin D ($10^{-7}$ M $1\alpha,25\text{-}OH_2D_3$) treated and untreated GFP-expressing human bone metastatic prostate cancer cells (PC-3) were isolated by ultracentrifugation (20.000 g and 100.000 g fractions) after one week of culture. EVs were counted by high-resolution flow cytometry (BD influx) and microscopy-image-guided counting (EVQuant). PC-3 EV mRNA content was analyzed by high-throughput next generation sequencing and confirmed by qPCR. PC-3 EV uptake by human osteoblasts (SV-HFO) was measured by flow cytometry (BD Accuri C6) and qPCR.

Vitamin D treatment significantly decreased PC-3 cell number by 68% ($p< 0.0001$). The surviving cancer cells produced a significantly higher number of EVs per cell (100,000 g fraction: 82% increase, $p=0.0008$). These EVs contained a different set of preferential mRNA cargo (based on EV/cell expression ratio) and were less efficiently internalized (100.000 g: 15% reduction ($p=0.013$), 20.000 g: 43% reduction ($p=0.0260$) by human osteoblasts compared to the EVs from untreated cells. Remarkably, despite this decreased EV uptake by osteoblasts, gene expression levels of the preferentially packaged EV mRNAs of the vitamin D treated PC-3 cells were highly upregulated in the recipient osteoblasts (PI3 and SLPI ($p=0.0286$)).

In conclusion vitamin D negatively affects EV communication of bone metastatic prostate cancer cells, but surviving cancer cells have an increased capacity to set their landmarks for metastatic outgrowth in bone via EVs.

**P059**

**Biological Potential Of Synergistic Effect Of Taspine Derivative With Other Phytochemical For The Treatment Of T-Cell Acute Lymphoblastic Leukemia With Their Molecular Mechanism**

*Kanika Patel*[1], *Dinesh Kumar Patel*[2]
[1]*SHUATS, Department of Pharmaceutical Sciences, Allahabad, India ;* [2]*Sam Higginbottom University of Agriculture- Technology and Sciences, Department of Pharmaceutical Sciences, Prayagraj, India*

**Abstract Text**

**Backgrounds:** Plants derived secondary metabolites have been used in medicine for the treatment of numerous human disorders. Phytochemicals have been used as vital components in drug discovery for the treatment of both communicable and non-communicable diseases. Taspine is a bioactive aporphine alkaloid isolated from *Leontice ewersmannii*, which has many potent pharmacological effects, including leukemia. Acute lymphocytic leukemia is a type of cancer of the blood and bone marrow. Taspine has many pharmacologic activities and found that taspine has an effect on anti-tumor and inhibits angiogenesis of chicken chorioallantoic membrane.

**Purpose:** Biological potential of synergistic effect of taspine derivative with other phytochemical for the treatment of T-cell acute lymphoblastic leukemia has been investigated in the present work.

**Methods:** Present work investigated the therapeutic potential of taspine derivative with berberine for their effectiveness on T-cell acute lymphoblastic leukemia cell growth. Scientific data of the taspine derivative and other phytochemical have been analyzed in the present work in order to know the biological potential of taspine for their effectiveness on lymphoblastic leukemia. Other pharmacological activity of taspine has also been analyzed in the present work in order to signify the therapeutic effectiveness of taspine in medicine.

**Results:** Present work signified the biological potential and therapeutic effectiveness of taspine in medicine. Combination of taspine derivative and berberine on the growth of T-cell acute lymphoblastic leukemia cell has been investigated and found to significantly inhibit the proliferation of Jurkat cells. Further, combination of taspine derivative and berberine induced G1 -phase cell-cycle arrest and enhanced apoptosis in Jurkat cells. However taspine derivative and berberine combination also regulated the ephrin-B2 and VEGFR2 signaling signified its therapeutic effectiveness for the treatment of T-cell acute lymphoblastic leukemia.

**Conclusion(s)**: Present work signified the biological potential of taspine derivative and berberine combination for the treatment of T-cell acute lymphoblastic leukemia.

**P060**

**Investigating the role of RANKL in a breast cancer mouse model of bone metastasis**

*Evi Gkikopoulou*[1,2], *Christos-Chrysovalantis Syrigos*[1,2], *Chrysa-Eleni Petraki*[1,2], *Ioanna Mantogiannakou*[1], *Melina Dragolia*[3], *Vasileios Ntafis*[3], *Martina Rauner*[4], *Eleni Douni*[1,2]

[1]Biomedical Sciences Research Center "Alexander Fleming", Institute for Bioinnovation, Athens, Greece ; [2]Agricultural University of Athens, Department of Biotechnology, Athens, Greece ; [3]Biomedical Sciences Research Center "Alexander Fleming", Institute for Fundamental Biomedical Research, Athens, Greece ; [4]Faculty of Medicine, Department of Medicine III, Dresden, Greece

**Abstract Text**

**Background:** Receptor activator of nuclear factor-κB ligand (RANKL) despite of being the main mediator of osteoclastogenesis and bone resorption, it has been also associated with hormone-driven breast carcinogenesis and bone metastasis. Denosumab a monoclonal antibody targeting RANKL and bisphosphonates such as Zoledronic acid that inhibit osteoclasts are medications used to treat and prevent bone-related issues, including osteoporosis and bone metastasis by reducing bone destruction.

**Purpose:** Establishment of a breast cancer derived bone metastasis model in osteoporotic mice overexpressing human RANKL (TgRANKL) and investigation of bone metastasis development upon anti-resorptive therapies.

**Methods:** EO771 mouse breast cancer cells were injected systemically to recapitulate bone metastasis in the hind limbs. Denosumab (Dmab) or Zoledronic acid (ZA) were administered as prophylactic treatments, whereas the therapeutic effect of Dmab was also evaluated.

**Results:** *In vivo* imaging at 19 days post-injection of EO771 cells revealed that 66% of TgRANKL (Tg) mice had a positive metastatic signal in their bones in contrast to their WT littermates or treated mice (% of mice with positive BL signal <30%). Moreover, TgRANKL mice exhibited increased tumor burden and severe cortical bone loss, whereas prophylactic treatments inhibited metastasis (%Tumor area/bone: WT=1.99±3.44; Tg=50.38±18.25; Tg+Dmab=11.95±12.85; Tg+ZA=7.22±12.20; $p<0.001$) and restored cortical bone volume at the baseline level (Ct.BV/mm3: WT=1.42±0.11; Tg=0.69±0.19; Tg+Dmab=2.35±0.17; Tg+ZA=2.09±0.21, $p<0.001$). In contrast, therapeutic Dmab administration did not reduce metastasis in the TgRANKL mice however it minimized metastasis-induced bone loss and osteolysis.

**Conclusions:** Collectively, our results demonstrated that prophylactic administration of anti-resorptive drugs, Denosumab and Zoledronic acid, effectively reduced bone metastasis and prevented cortical bone loss in TgRANKL mice, while Denosumab as a therapy protected from secondary skeletal related events.

*The research work was supported by the Hellenic Foundation for Research and Innovation (HFRI) under the 3rd Call for HFRI PhD Fellowships (Fellowship Number: 05346).*

**P061**

**Studying RANKL in mammary and prostate gland pathophysiology in an osteoporotic TgRANKL mouse model**

*Christos-Chrysovalantis Syrigos[1,2], Evi Gkikopoulou[1,2], Dimitrios Lymperopoulos[1,3], Anthi Kolokotroni[1,2], Eleni Douni[1,2]*
[1]Biomedical Sciences Research Center "Alexander Fleming", Institute for Bioinnovation, Athens, Greece ; [2]Agricultural University of Athens, Department of Biotechnology, Athens, Greece ; [3]European University Cyprus, School of Medicine, Nicosia, Cyprus

**Abstract Text**

**Background:** Receptor activator of nuclear factor-κB ligand (RANKL) is the main regulator of bone loss through the induction of osteoclast differentiation. Although it has been associated with hormone-induced carcinogenesis in the mammary gland, the mechanisms of carcinogenesis and metastasis involving RANKL remain unclear. Bone metastasis is a common phenomenon in advanced stages of several types of cancer, including breast and prostate cancer which are leading causes of cancer-related death.

**Purpose:** In the present study, we examined the mammary and prostate glands of osteoporotic TgRANKL mice that overexpress human RANKL (huRANKL).

**Methods:** Gene expression levels in mammary and prostate gland were measured by qPCR and morphology of the glands was analyzed by whole-mount carmine staining and histology with hematoxylin/eosin staining.

**Results:** Our results showed that the luminal epithelial cells of the mammary glands of TgRANKL mice express huRANKL and display increased proliferation as shown by the CyclinD1 marker. Analysis of the TgRANKL mammary glands demonstrated increased density in the network of mammary ducts, especially secondary and tertiary branches when compared to wild-type (WT) mice, that was prevented by the monoclonal antibody Denosumab against huRANKL. Moreover, qPCR results revealed a significant increase in the expression of breast cancer-associated genes such as Akt1, Vegfa, Stat5a, Ccnd1, Adam9 and Marco in the mammary glands of TgRANKL mice compared to those of WT. As regards the prostate gland, qPCR analysis showed that huRANKL was expressed locally at the prostate gland of TgRANKL mice, whereas histological analysis did not reveal gross structural differences in the four types of paired prostatic lobes between WT and TgRANKL mice.

**Conclusions:** Collectively, our results demonstrate that increased expression of RANKL in the mammary glands of osteoporotic TgRANKL mice is correlated with enhanced proliferation of mammary epithelial cells and increased mammary density, while the prostate gland of TgRANKL mice seems physiological.

**P062**

**Multilayered control of inflammation/immunity in osteosarcomas: contribution of cancer stem cells**

*Minh-Anh Huynh[1], Ostertag Agnès[2], Camille Péron[3], Martine Cohen-Solal[3], Dominique Modrowski[4]*
*[1]Institut de la santé et de la recherche médicale, U1132- BIOSCAR- Biologie de l'os et du cartilage, paris, France ; [2]Institut de la santé et de la recherche médicale INSERM, U1132- BIOSCAR- Biiologie de l'os et du cartilage, paris, France ; [3]Institut de la santé et de la recherche médicale- INSERM, U1132- BIOSCAR- Biologie de l'os et du cartilage, paris, France ; [4]U1132 INSERM, BIOSCAR- Biologie de l'os et du cartilage, Paris, France*

**Abstract Text**

In osteosarcomas, the richness of the immune compartment was associated with better survival rates. We explored the inflammation status of bone tumors to highlight mechanisms regulating this compartment. We used transcriptomic data sets and build an inflammation index with the HALLMARK_OF_INFLAMMATORY_RESPONSE signature (INFIND). Patients with low INFIND had lower immune score and worse 5-year survival rate. Analyses of differentially expressed genes indicated that hypoxia, autophagy, cytokine activity were down-regulated and that ribosomal machinery and ATP synthesis were up-regulated in low compared to high INFIND tumors. Genes involved in cell fate specification/commitment during development were also altered. This suggested a possible relation between tumor stemness and the control of immunity. We previously showed that Calpain-6 is a biomarker of sarcoma stem cells. CAPN6 knockdown in cultured osteosarcoma cells resulted in downregulation of immune suppressors (CD73, CD39, PD-L1, IL10…). In vitro, osteosarcoma cells induced M2 polarization of macrophages. This effect was dependent on Calpain-6 expression. In the human tumors with high INFIND the expression of CAPN6 was associated with lower immune scores, decreased cytokines activity, antigen binding, Ig production and up-regulation of genes of hypoxia and glycolysis and smaller patient survival rate. At the opposite, patients with low INFIND and no CAPN6 expression had the worse survival rate. CAPN6 expression in bone tumors with low INFIND was associated with good survival rate, re-organization of the cytoskeleton, ion transport and extracellular matrix, with decreased oxidative phosphorylation and ribosomal activity.

In conclusion, considering the inflammation status and CAPN6 expression allowed prediction of patient outcome and highlighted possible mechanisms that control the immune compartment of osteosarcoma tissues and thereby could interfere with immunotherapy. Our results unveiled an immunosuppressive function for sarcomas stem cells especially in high inflammation contexts. Deciphering the CAPN6-dependent mechanisms will help to determine new therapeutic targets for the patients with worse outcome.

**P063**

**A microfluidic bone-on-a-chip platform to study the role of citrate in prostate carcinoma tropism to bone**

*<u>Sofia Avnet</u>[1], Mariaveronica Lipreri[2], Elena Cavassi[1], Elisa Boanini[3], Gemma Di Pompo[4], Nicola Baldini[1]*
*[1]University of Bologna, Department of Biomedical and Neuromotor Sciences, Bologna, Italy ; [2]IRCCS-Istituto Ortopedico Rizzoli, Biomedical Science- Technologies- and Nanobiotecnologiy Lab, Bologna, Italy ; [3]University of Bologna, Department of Chemistry 'Giacomo Ciamician', Bologna, Italy ; [4]IRCCS Istituto Ortopedico Rizzoli, Biomedical Science- Technologies- and Nanobiotecnologiy Lab, Bologna, Italy*

**Abstract Text**

**Background:** Prostate cancer exhibits distinct citrate metabolism and bone tropism. Nutrient availability influences organotropism. Like the prostate, bone contains high citrate levels within its mineralized matrix and secreted by osteogenic cells. Despite frequent bone metastasis, PCa's bone behaviour and citrate dependence remains understudied. Flat biology models overlook 3D stromal interactions and concentrated tumor microenvironment metabolites. Our bone-on-a-chip model recapitulating osteocytes and bone minerals provided a 3D system to study citrate's role in PCa's bone tropism [1].

**Purpose:** Using a 3D bone-on-a-chip model recapitulating the bone microenvironment's metabolic complexity, we aimed to elucidate PCa's pronounced bone tropism and identify new metabolic therapeutic targets for bone metastatic PCa, focusing on citrate metabolism.

**Methods:** We developed hydroxyapatite nanoparticles (HA-NP) with/without citrate and loaded them into a collagen matrix with MLO-Y4 osteocytic cells. This engineered bone matrix injected into a microfluidic platform created a chip modeling PCa bone metastasis (Fig. 1a). Seeded PCa cells were evaluated for viability and clonogenicity with/without citrate. Roles of citrate pathways in PCa activities were also investigated using molecular analysis and advanced imaging.

**Results:** We successfully obtained citrate-enriched HA-NP and mixed them with the collagen-, osteocyte- and HA-NP-enriched extracellular matrix. After mixing with PCa cells, the engineered hydrogel was loaded into the microfluidic chips. As expected, we observed formation of the osteocytic dendrite network. Furthermore, despite unchanged viability (Fig. 1b), increasing citrate concentrations caused increased PCa spheroid formation.

**Conclusion:** Our advanced 3D bone metastasis model elucidated citrate's role in PCa's pronounced bone tropism. Results will improve 3D PCa bone metastasis models and identification of novel therapeutic targets based on PCa's unique citrate metabolism.

Lipreri MV, Di Pompo G, Boanini E, Graziani G, Sassoni E, Baldini N, Avnet S. Bone on-a-chip: a 3D dendritic network in a screening platform for osteocyte-targeted drugs. Biofabrication. 2023;15(4).

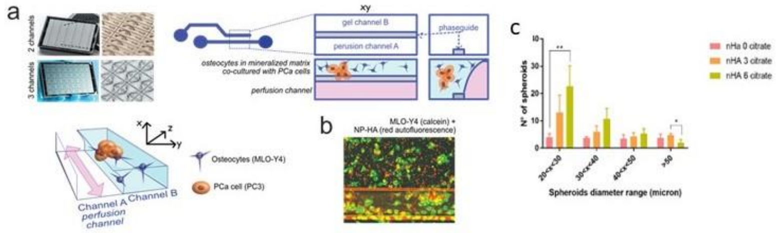

**P064**

### Characterization of circulating and tumor infiltrating neutrophils in a murine model of osteosarcoma

*Diane Ji Yun Yoon[1], Juliette Samaniego[1], Mylène Zarka[1], Maria Eugenia Marques Da Costa[2], Dominique Modrowski[1], Martine Cohen Solal[1], Antonin Marchais[2], Guillaume Odri[1]*
*[1]INSERM, u1132 BIOSCAR, Paris, France ; [2]INSERM, u1015 ITIC, Paris, France*

**Abstract Text**

Osteosarcoma, the most frequent primary high-grade sarcoma of skeleton, remains a health challenge despite a highly aggressive treatment. Neutrophils are innate immune cells involved in various cancers, but their role in osteosarcoma is largely unknown. Our hypothesis was that infiltrating neutrophils (called Tumor Associated Neutrophils, TAN) and circulating neutrophils (CN) influence tumor development. We aimed to identify and characterize neutrophils in order to better understand their role in a murine intratibial model of osteosarcoma.

We first confirmed their presence by identifying and counting neutrophils in blood, bone marrow and tumor, using an anti-Ly6G immunohistochemistry and FACS. We observed that TAN represented 16,6% of living cells and that higher proportions of neutrophils were found in tumor-bearing (TB) versus sham mice in blood (54,4% (95% CI 42-67) versus 21,4% (95% CI 9-34), p=0,0023) and in bone marrow (49% (95% CI 44-54%) versus 36% (95% CI 29-43), p=0.0084).

Then we performed a transcriptomic analysis on 33 943 neutrophils from blood, bone marrow and tumor and observed that CN of TB mice had a different transcriptomic profile than those of sham mice, and that TAN had a unique transcriptomic signature. After enrichment, TAN had an activated cytokine-mediated response but inhibited mRNA process and cellular organization pathways.

Finally, immunohistochemistry and immunofluorescent analysis revealed a co-localization of Ly6G, myeloperoxidase, histone H3 and cleaved caspase 3 stainings on necrotic areas, suggesting that the presence of TAN was associated to cell apoptosis, as well as the Neutrophil Extracellular Trap (NET) formation.

We explored the role of neutrophils in intratibial osteosarcoma development, from maturation stage to TAN, which were associated to an activated cytokine-mediated response and to cell necrosis and apoptosis through NETosis. Our results provide insights for further studies on the interactions between neutrophils, osteosarcoma cells, and the tumor microenvironment, as well as potential therapeutic perspectives.

**ND-P012**

**Raman Spectroscopy Coupled with Machine Learning Unveils Temporal Cell-Associated ECM Signatures for Early Classification of Osteosarcoma Cell Lines**

*<u>Theodore Hornsey</u>[1], Aikta Sharma[2], Lysanne Michels[1], Jemma Kerns[3], Claire Clarkin[1]*
*[1]University of Southampton, School of Biological Sciences, Southampton, United Kingdom ; [2]University College London, Department of Mechanical Engineering, London, United Kingdom ; [3]Lancaster University, Lancaster Medical School- Faculty of Health and Medicine, Lancaster, United Kingdom*

**Abstract Text**

**Introduction:** Osteosarcoma (OS) is the most common and deadly malignant bone tumour (1). Its lethality is largely due to the slow and difficult diagnostic process, involving both biopsy and imaging (2). New approaches are required to improve diagnostics and patient outcomes. OS is characterised by an altered extracellular matrix (ECM) and vasculature for tumour progression into metastasis. We previously found that human OS cell lines can be distinguished from murine osteoblast (OB) cells using Raman Spectroscopy (RS).

**Purpose:** We now aim to combine RS with machine learning (ML) to predict the different OS grades via their ECM signatures.

**Methods:** Human Soas-2 (low-grade), human MG63 (medium-grade) and human 143B (high-grade) OS cell lines were cultured *in-vitro* for up to 14 days; as were Murine OB cells (MC3T3). Raman spectra of individual cells (n=15 per subgroup) were collected at day 1,4 and 14. Random Forest ML models of spectra were developed in Python to classify a cell as OS or OB, including pathological grading. Feature importance from each peak and model accuracy was determined to identify the underlying biological components contributing to the model's predictive accuracy.

**Results:** OS phenotypes were predicted with an average 79.87% accuracy both within and between timepoints, indicating that each cell produced a unique and temporally distinct cell-associated ECM as early as day 1 in development. Multiple ECM components including collagen, lipids, amide I and III were identified as important features for prediction between cell lines.

**Conclusion:** Using machine learning on Raman spectra of the extracellular matrix, we can accurately predict the different grades of murine OS cells. This suggests that our developed technique may be used to improve osteosarcoma diagnosis, thereby leading to better patient prognoses.

**References:**
1. Marulanda et al. DOI:10.1177/107327480801500103
2. Hayashi et al. DOI: 10.1007/s10147-013-0592-z

**Poster Sessions: Osteoblasts and bone formation**

**P065**

**Hematopoietic autophagy defect contributes bone loss**

*Ye Yuan*[1]
[1]*Peking University People's Hospital, Arthritis Clinic & Research Center, Beijing, China*

**Abstract Text**

**Objective:** Hematopoietic disorders are known to increase the risk of complications such as osteoporosis.

**Methods:**
（1） Physical examination results of 4979 healthy individuals were analyzied for blood count and BMD. Bone marrow were obtained from young normal BMD patient and aged osteoporosis patients. Bone marrow monocytes were measured by flowcytometry.

（2） Ovariectomized mice bone marrow hematopoietic stem and progenitor cells autophagy function were measured.

（3） Mice deleted Atg7 gene, using an Vav-Cre transgene, which targets cells at bone marrow hematopoietic cells (Atg7f/f;Vav-Cre); Bone histomorphology was detected.

（4） Atg7f/f;Vav-iCre bone tissue was collected for TMT labeled proteome analysis. Western blot was used to verifified the proteome results.

（5） Atg7f/f;Vav-iCre tibia CD31hiEMCNhi vessel was observed by immunoflfluorescence. mRNA of vessels metablism was measured.

**Results:**
（1） Red blood cell count was reduced in both male and female osteoporotic patients. Human hematopoietic stem progenitor cells (CD34+CD45+) LC3 protein was inhibited in aged people.

（2） The proportion of hematopoietic stem and progenitor cells was increased in ovariectomized mice. Bone marrow hematopoietic stem cells displayed a reduced basal autophagy activity in ovariectomized mice.

（3） Deletion of Atg7 in hematopoietic system caused low bone mass and bone formation ratio. The H＆E staining showed Atg7f/f;VaviCre mice trabecular degeneration and more fat tissue. The TRAP staining showed there was more abundance and activity of osteoclasts in Atg7f/f;Vav-iCre mice. Scanning electron microscope depicted trabecular microstructure destruction. The absence of Atg7 in hematopoietic resulted in weak bone biomechanical strength properties.

（4）Integrative proteomics functional enrichment of diffferentially quantifified proteins showed Atg7f/f;Vav-Cre mice skeletal system morphogenesis and development down-regulated. Collagen1 in ECM-Receptor interaction was down-regulated, which was verified by immunohistochemical.

（5）Bone CD31hiEMCNhi vessel was inhibited in Atg7-/- mice. RT-PCR showed Vegfa was down-regulated in Atg7-/- mice.

**Conclusions:** Hematopoietic autophagy defect causes disruption of CD31hiEMCNhi vessel via inhibition of Vegfa, resulting in bone loss.

**P066**

**Extracellular phosphate control of PHOSPHO1 and alkaline phosphatase expression by osteoblasts.**

*Soher Jayash*[1], *Qaisar Tanveer*[1], *Colin Farquharson*[1]
[1]*University of Edinburgh, The Roslin Institute, Edinburgh, United Kingdom*

**Abstract Text**

**Background/Introduction:** Biomineralisation of the skeleton is essential for skeletal health but the ability of the osteoblast to sense extracellular phosphate and control the timely deposition of mineral within the collagen matrix is poorly understood.

**Purpose:** The project explored the ability of osteoblasts to sense extracellular phosphate concentrations and control the local production of phosphatases such as tissue-nonspecific alkaline phosphatase (TNAP) and PHOSPHO1.

**Methods:** Human primary osteoblasts were cultured with varying phosphate concentrations (0, 1mM, 3mM & 5mM), and matrix mineralisation (alizarin red staining) and PHOSPHO1, TNAP, *SLC20A1*/PiT1, and *SLC20A2/*PiT2 expression (RT-qPCR and immunoblotting) were examined. Immunoblotting with antibodies to total and phosphorylated forms of ERK1/2, AKT, p38, and JNK1/2 was employed to detect intracellular signalling pathways. Additionally, specific inhibitors of MEK 1/2 (U0126) and Pit1/Pit2 (phosphonoformic acid) were introduced to the cultures to explore the pathways activated by extracellular phosphate.

**Results:** Increasing extracellular phosphate promoted osteoblast mineralisation whereas PHOSPHO1 and TNAP expression was down-regulated in response to increasing phosphate concentrations at both the mRNA and protein levels ($p<0.05$). Extracellular phosphate also downregulated PiT-1 and PiT-2 expression at the mRNA level ($p<0.05$), but enhanced the phosphorylation of ERK1/2 but not AKT, JNK, or P38. The phosphorylation of ERK 1/2 by extracellular phosphate was suppressed by phosphonoformic acid. Co-treatment with U0126 or phosphonoformic acid abolished the decrease in *PHOSPHO1 and ALPL* expression by elevated extracellular phosphate.

**Conclusions:** These results demonstrate that extracellular phosphate is sensed by osteoblasts and promotes matrix mineralisation but down-regulates the expression of TNAP and PHOSPHO1 via type III Na/Pi co-transporters, PiT-1 and PiT-2, and ERK 1/2 signalling. These findings contribute to a better understanding of the molecular events associated with the control of osteoblast matrix mineralisation and suggest that the local production of phosphate by bone forming osteoblasts may

"fine-tune" or be "rate-limiting" in physiological bone mineralisation.

**P067**

**Delineating the role of Fshr in Bone Remodeling Using Novel Genetic Mouse Models**

*Mingxin Sun[1], Hongqian Chen[1], Li Xing[1], Liben Cheng[1], Zemin Liu[1], Wenhuan Chai[1], Zhenwei Yang[1], Clifford Rosen[2], Peng Liu[1]*
*[1]Shanxi Medical University, Lab of Bone and Adipose Biology, Taiyuan, China ; [2]Maine Medical Center Research Institute, Maine Medical Center Research Institute, Scarborough, United States*

**Abstract Text**

Fsh, as a gonadotropin hormone, regulates reproductive organs through its receptor Fshr. However, increasing evidence indicates that the Fsh-Fshr axis also plays a role in extragonadal organs, such as bone, fat and vasculature, although the extragonadal expression and function of Fshr continue to be debated. To address this challenge, we created an Fshr promoter-driven ZsGreen reporter line for more precisely defining the localization of Fshr expression. Interestingly, in the skeleton, we observed Fshr in osteoclasts, and most importantly, expressed in osteoblasts, bone lining cells and osteocytes. To understand the role of Fshr in bone formation, we also developed Fshr cKO mice and bred them with a 10kb DMP1-CreERT2 line to investigate the role of Fshr in bone remodeling. We used micro-CT to sequentially assess bone and adipose compartments, and found significant increases in trabecular and cortical bone mass in both male and female tamoxifen-induced Fshr knockout (FshrKO) mice, compared to corresponding controls. In addition, we also noted an increase in serum markers for osteocalcin, PINP and TRACP5b, and histomorphometric parameters of bone resorption, indicating a high remodeling in the Fshr cKO mice. We also examined ovariectomized Fshr cKO mice and found similar findings in bone mass; i.e. there was a significant increase in trabecular bone mass in the Fshr cKO after ovariectomy, compared to loss of trabecular bone volume fraction in the control mice. Furthermore, we recently observed a significant increase in fracture healing in the Fshr cKO mice and a substantial Fsh inhibition of mineralization in osteoblast cultures. These data provide exceptional evidence that Fshr is expressed in several cells of the bone remodeling unit and that Fshr cKO in osteocytes can dramatically impact bone remodeling. Our results suggest that removing the inhibitory action on bone formation by the Fsh-Fshr complex could have valuable therapeutic implications.

**P068**

**Prdm16, A Key Regulator of Energy Metabolism, Regulates Bone Remodeling**

*Liben Cheng[1], Li Xing[1], Mingxin Sun[1], Hongqian Chen[1], Peng Liu[1]*
*[1]Shanxi Medical University, Laboratory of Bone and Adipose Biology, Taiyuan- Shanxi, China*

**Abstract Text**

Bone is the second most dynamic organ in metabolism in human beings. However, a mechanism underlying regulation of energy metabolism of bone remodeling is largely unknown. To explore it, we aimed to investigate a role of Prdm16 -a key regulator in control of energy metabolism, in bone remodeling. Because Prdm16 knockout in adipose tissue caused obesity in mice, we therefore hypothesized that the deficiency of Prdm16 in bone would lead to osteoporosis, presumably due to a deficit of energy supply to drive bone remodeling. To address it, we employed a 10kb DMP1-CreERT$^2$ line to specifically delete Prdm16 in osteocytes. When mice aged at 2 months, we injected tamoxifen (TMX) to induce cKO of Prdm16 in osteocytes. After 2 months of TMX administration, we found a profound increase of bone mass in both cortical and trabecular bones in the cKO mice, compared to the control mice. In addition, we noted an increase in serum markers for both bone formation and resorption, indicating a high bone turnover in the cKO mice. Similarly, we also observed that the cKO protected mice from bone loss and even gained more bone in ovariectomized cKO mice than the sham mice and enhanced fracturre healing. Intriguingly, we also observed an impressive reduction in both body weights and adipose tissues in the cKO mice and tolerated high glucose/insulin challenges, compared to the corresponding controls, when fed *ad lib* with either normal chow or high feed diet. To probe for the mechanism responsible for this change, we further performed femoral RNA-seq analysis, indicating that at least these two factors (Sost and Lcn2) secreted from bone are responsible for the interaction of bone with adipose when Prdm16 was deleted in osteocytes. Overall, our compelling data uncover that Prdm16 in osteocytes plays a significant role in bone remodeling and fat metabolism.

**P069**

**The human amniotic mesenchymal stromal cells' secretome modulates osteogenic differentiation of mesenchymal stromal cells in Craniosynostosis patients**

*Alessia Vita[1], Federica Tiberio[2], Lorena Di Pietro[2], Andrea Papait[2], Antonietta Silini[3], Pietro Romele[3], Gianpiero Tamburrini[4], Ornella Parolini[2], Wanda Lattanzi[2]*
*[1]Università Cattolica del Sacro Cuore, Dipartimento di Scienze della Vita e Sanità Pubblica, ROME, Italy ; [2]Università Cattolica del Sacro Cuore- Fondazione Policlinico Universitario A. Gemelli IRCCS, Dipartimento di Scienze della Vita e Sanità Pubblica, Rome, Italy ; [3]Fondazione Poliambulanza-Istituto Ospedaliero, Centro di Ricerca E. Menni CREM, Brescia, Italy ; [4]Università Cattolica del Sacro Cuore-Fondazione Policlinico Universitario A. Gemelli IRCCS, Dipartimento Scienze dell'invecchiamento-neurologiche- ortopediche e della testa-collo, Rome, Italy*

**Abstract Text**

**Background:** Skull flat bones and sutures house multipotent calvarial mesenchymal stromal cells (CMSCs) that contribute to perinatal skull morphogenesis and growth. The disruption of the homeostatic balance between growth and osteogenic differentiation in the calvarial stem cell niche, due to genetic and environmental factors, results in accelerated osteogenic differentiation of CMSC leading to the premature fusion of cranial sutures (i.e. craniosynostosis, CS). Human amniotic mesenchymal stromal cells (hAMSC) have remarkable trophic and regenerative properties exerted via the release of paracrine factors in their secretome. They are being extensively studied as a therapeutic strategy in tissue regeneration. Compared with MSCs from other tissues, hAMSC display a reduced osteogenic potential.

**Purpose:** This study aimed to assess whether the hAMSCs secretome can modulate the proliferation and osteogenic differentiation of CMSCs. This potential strategy could be translated into a treatment to hamper the aberrant ossification of calvarial sutures in CS.

**Methods:** CMSCs were isolated from the cranial suture samples collected from 6 CS patients (CE approved protocol #4876). CMSCs were pretreated with hAMSC-derived conditioned medium (hAMSC-CM) for 72 hours, then induced to osteogenic differentiation using hAMSC-CM supplemented with osteoinductive factors in time course experiments. The expression of proliferative and osteogenic genes was investigated by qPCR, while mineralized matrix deposition through Alizarin Red S (ARS) staining and quantification.

**Results:** Gene expression analysis revealed that the treatment with hAMSC-CM leads to a delay of the osteogenic differentiation of CMSCs reducing levels of osteogenic genes as *ALP*, *COL1A1*, *BGLAP*. ARS confirmed the reduced deposition of bone mineralized matrix in treated CMSCs. The expression of cell cycle-maker genes *CCNB1 and KI67* was regulated by hAMSC-CM treatment.

**Conclusions:** Our data suggest that hAMSC-CM may slow down the osteogenic differentiation of suture-derived CMSCs. Future studies are needed to characterize the biological effects of hAMSC-CM and its soluble factors on CMSCs fate.

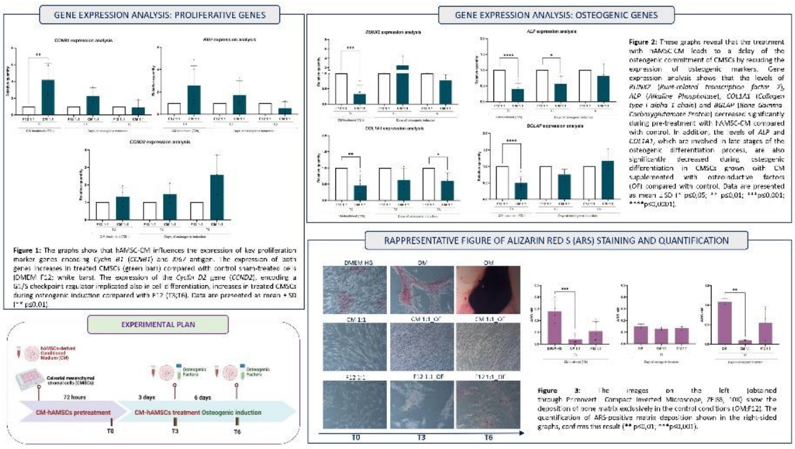

**P070**

**Defining a therapeutic window to treat osteopathia striata with cranial sclerosis in a mouse model**

*Clément Nachef*[1], *Arnaud Vanjak*[1,2], *Sylvie Thomasseau*[1], *Badre Rabhi*[1], *François Brial*[1,3], *Martine Cohen-Solal*[1,2,3], *Amélie Coudert*[1,4], *Thomas Funck-Brentano*[1,2,3]
[1]*BIOSCAR, Inserm U1132, Paris, France ;* [2]*Service de Rhumatologie, Hôpital Lariboisière, Paris, France ;* [3]*UFR de Médecine, Université de Paris, Paris, France ;* [4]*UFR d'Odontologie, Université de Paris, Paris, France*

**Abstract Text**

Osteopathia striata with cranial sclerosis (OSCS, OMIM#300373) is a rare genetic osteosclerotic disease caused by *WTX* mutations, a repressor of Wnt signaling pathway, leading to Wnt activation in osteoblasts, and therefore to increased bone formation. Currently lacking specific treatment, OSCS poses a challenge in clinical management.
In order to treat OSCS patients, we want to test pharmaceutical therapies, hence we needed to define a treatment window in a mouse model previously generated. We followed the bone phenotype from 4 to 16 weeks of age both in hemizygous male and heterozygous females of the Prx1-Cre; $Wtx^{flox/flox}$ mice.
As early as 4 weeks, the mutant male mice showed a significant increase of the femur BMD (+19%, p=0.0010) that goes up to +35% at 11 weeks (p<0.001) in comparison to the control mice. At 8 weeks, hemizygous males Prx1-Cre; $Wtx^{flox/Y}$ presented, compared to WT mice, an increased BV/TV, Tb.Th and Tb.N (p<0.001), and a decreased of Tb.Sp (p<0.0001). Ct.BV was also increased (p<0.0001). Bone histomorphometry analyses showed an increase of osteoid surface (OS), osteoblast number (Ob), bone formation rate (BFR), and an increase of TRAP positive cells (p<0.01). At 11 weeks, hemizygous males, compared to WT mice, an increased BV/TV, Tb.Th and Tb.N (p<0.001), and a decreased of Tb.Sp (p<0.0001). Ct.BV was also increased (p<0.0001). The heterozygous female mice Prx1-Cre; $Wtx^{flox/wt}$ showed an increased cortical and trabecular bone mass phenotype but in a less extend.
The analysis of the bone phenotype in the OSCS mouse model allowed us to define the therapeutic window between 8 and 11 weeks. The next steps will be to test targeted therapies in order to blunt the excessive bone formation characterizing the OSCS. Our final goal is to reposition the successful tested drugs for the management of patients affected by this orphan disease.

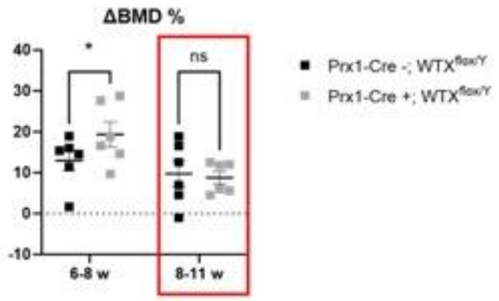

Figure : Definition of the therapeutic window

**P071**

**Proteomic profile of osteoblasts induced by adipocytes**

*Mariana Sanches[1], Cintia Tokuhara[2], Flávia Oliveira[2], Talita Ventura[1], Adriano Pessoa[1], Flávia Iano[3], Emanuelle Carvalho[1], Marília Buzalaf[1], Rodrigo Oliveira[1]*
*[1]Faculdade de Odontologia de Bauru - Universidade de São Paulo, Ciências Biológicas, Bauru, Brazil ; [2]Sanford Children's Health Research Center, Human Genetics Program, San Diego, United States ; [3]Faculdade de Odontologia de Araçatuba - UNESP, Departamento de Odontologia Preventiva e Restauradora, Araçatuba, Brazil*

**Abstract Text**

Introduction: Bone cells and adipocytes have a dynamic relationship within the bone marrow, playing crucial roles in bone metabolism and overall homeostasis of the skeletal system. It is known that osteoblasts and adipocytes share a mesenchymal progenitor, and osteo-adipogenic dysregulation can trigger pathological conditions such as osteoporosis. Purpose: We sought to study the proteomic profile and function of osteoblasts in the presence of adipogenic stimulation. Methods: Thus, adipocytes conditioned media (CM) were collected at 12 days of differentiation. Osteoblasts were treated with CM for up to 21 days, and mineralization assay, gene expression, and proteomic analysis were performed. Results: Our results showed that adipocytes CM treatment inhibited the formation of calcium deposition in osteoblasts, followed by inhibition of Alpl and Bglap gene expression in all time points. In regard to the proteomic profile, several proteins were regulated, such as fibronectin, histone subunits, hemoglobin, asporin, and vimentin. The imbalance of these proteins modulates inflammatory responses, metabolic dysregulation, and the cell cycle, thus determining whether or not bone formation occurs. Proteomic analysis showed a downregulation of proteins related to bone formation when treated with adipocytes CM. Conclusions: Therefore, our results indicate that important factors were secreted by adipocytes that directly influenced the homeostasis of osteoblasts, promoting a non-mineralization pattern and consequently, negatively modulating the protein profile of these cells. Future studies are needed to identify and characterize the adipogenic factors with inhibitory mineralization potential in osteoblasts. These results may have significant implications for future research and the development of therapeutic strategies for conditions related to bone disorders.

**P072**

**Effect of ginsenoside Rb1 and Rb2 on proliferation and osteogenic differentiation of C2C12 cells**

*Heesu Lee[1], Seong-Hee Ko[2]*
*[1]College of Dentistry and Research Institute of Oral Science- Gangneung-Wonju National University, Dept of Oral anatomy, Gangneung, Korea- South ; [2]College of Dentistry and Research Institute of Oral Science- Gangneung-Wonju National University, Dept of Pharmacology, Gangneung, Korea- South*

**Abstract Text**

**Introduction/Purpose:** The current treatment for osteoporosis mainly involves the use of drugs that suppress bone resorption, but their use is limited due to various adverse effects. Therefore, there is a need for the development of new bone-forming agents with lower risks compared to conventional treatments. Ginseng is one of the most commonly used herbal remedies known for its various therapeutic effects and it is also believed to have therapeutic effects on osteoporosis. This study aimed to develop safer anabolic agents for treating osteoporosis by exploring the effects of ginsenosides, active compounds found in Panax Ginseng, on bone formation. Specifically, the effect of ginsenoside Rb1 (G-Rb1) and ginsenoside Rb2 (G-Rb2) on cell proliferation and osteogenic differentiation of C2C12 cells, multipotent mesenchymal progenitor cells, were investigated.

**Methods:** The effects of Ginsenosides' on C2C12 cell proliferation were tested via a viability assay using Calcein AM. The study analyzed whether ginsenoside Rb1 and Rb2 enhanced osteogenic differentiation by examining mRNA expression levels of runt-related transcription factor-2 (Runx2), a key regulator of osteogenesis. Additionally, the effects of G-Rb2 on the mRNA expression of estrogen receptor α and β were examined.

**Results:** Our results showed that G-Rb1, G-Rb2, G-Rc and G-Rd significantly enhanced the proliferation of C2C12 cells, whereas G-Rg3 reduced it at higher concentrations. Moreover, G-Rb2 notably upregulated the gene expression of Runx2, suggesting its potential to enhance osteogenic differentiation. However, when treated with G-Rb1, there was no effect on the gene expression of Runx2. Futhermore, G-Rb2 upregulated the mRNA expression of both estrogen receptor α and β in C2C12 cells.

**Conclusion:** The observed results suggest that G-Rb2 have the potential as a promising treatment for osteoporosis due to its ability to stimulate the cell proliferation and the commitment of undifferentiated non-osteogenic cells into osteoblasts.

**P073**

**Quantitative three-dimensional and multiplexed analysis of human bone marrow reveals spatial heterogeneity of the stromal niche.**

*Tsz Long Chu[1], Emil Krukov[2], Yu-WEi Chang[3], Ostap Dregval[3], Dana Drompet[4], Shuo Misty Zhang[2], Emiliano Gomez Ruiz[3], Björn Barenius[5], Lars Sävendahl[6], Giovanni Volpe[7], Andrei S Chagin[8]*
*[1]Gothenburg University, The Institute of Medicine, Stockholm, Sweden ; [2]Karolinska Institutet, Pharmacology and Physiology, Stockholm, Sweden ; [3]University of Gothenburg, The Insitute of Medicine, Gothenburg, Sweden ; [4]The Insitute of Medicine, University of Gothenburg, Gothenburg, Sweden ; [5]Södersjukhuset, Karolinska Institutet, Stockholm, Sweden ; [6]Karolinska Institutet, Department of Women's and Children's Health, Stockholm, Sweden ; [7]University of Gothenburg,*

*Department of Physics, Gothenburg, Sweden ; [8]University of Gothenburg, The Insitutet of Medicine, Gothenburg, Sweden*

**Abstract Text**

CXCL12-abundant reticular (CAR) cells in bone marrow support hematopoiesis, encompass skeletal stem cells and form an extensive cellular network (ref. #1, 3, 4, 5, 6, 49). Depending on their location and surroundings, CAR cells form distinct niches for hematopoietic stem cells (ref. 2). However, literally nothing is known about spatial arrangement of CAR cells in humans. To fill this gap on knowledge, we first developed a tissue-clearing protocol allowing detection and spatial quantitative analysis of multiple mRNAs and proteins in large human bone biopsies, named *DeepBone*, and further combined it with an atlas of bone marrow single-cell sequences and with an unsupervised deep learning algorithm for microenvironment analysis. It revealed that in humans CAR cells are transcriptionally identical to bone marrow mesenchymal stromal cells (BM-MSCs), and all abundantly express CXCL12 chemokine. The niches in which CXCL12-expressing cells reside were formed by a complex composition of capillaries, sinusoids, adipocytes, and bony trabeculae, and the niche landscape drastically changed with age. The niche composition was heterogenic but formed a continuum in a latent space. The CD146-positive and CD271-positive subpopulations of human stroma, reported to be skeletal stem and progenitor cells, changed differently with advancing age. Novel CD271-positive membranes and sinusoidal-bone composites were also revealed. This is the first comprehensive spatial characterization of bone microenvironment in humans, which improves our understanding of the mechanisms underlying numerous hematological and bone diseases.

**P074**

**Viability and morphology of osteoblasts cultured on three calcium silicate-based root canal sealers**

*Caroline Arruda[1], Elisangela Chinen[1], Victor Elias Arana-Chavez[1]*
*[1]University of São Paulo, Biomaterials and Oral Biology, São Paulo, Brazil*

**Abstract Text**

**Introduction:** Mineral trioxide aggregate (MTA) is often used in endodontic treatments due to its bioactive properties and ability to promote bone regeneration. However, its solid consistency limits its use as a root canal sealer. To overcome this limitation, new sealers were developed to mimic the properties of MTA while maintaining a fluid consistency.

**Purpose:** This in vitro study aimed to assess the biocompatibility of three different calcium silicate-based root canal sealers, in comparison with MTA by evaluating their effects on osteoblast cell cultures.

**Methods:** MC3T3-E1 osteoblasts were cultured in plates. The test groups were exposed to: BioC Sealer (G1), Endosequence BC Sealer (G2), BioC Sealer Ion (G3). The positive control received MTA (PC), while control (C) and negative control (N) groups were included. The sealers were placed at the bottom of the culture plates. After the sealers had set, the cells were added, allowing the diffusion of the medium. Evaluations were performed at 24, 48, and 72 hours. Cell viability was determined through direct cytotoxicity analysis using the MTT method. The control groups were cultured using plates without sealers. The negative control group received phenol treatment. Additionally, scanning electron microscopy was employed to examine the morphological features of the cells.

**Results:** The results showed that all tested sealers were biocompatible and supported cell growth. BioC Sealer, BioC Sealer Ion, and MTA exhibited higher cell growth and adhesion, while Endosequence showed fewer cells. BioC Sealer Ion demonstrated enhanced cell growth, adhesion, and higher cell viability compared to the other groups.

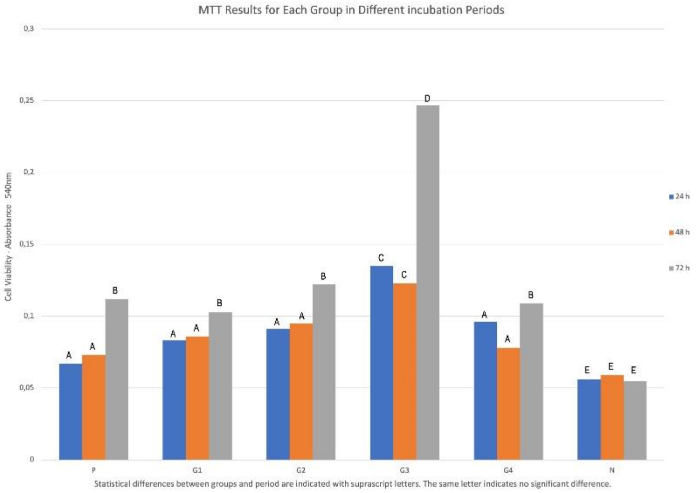

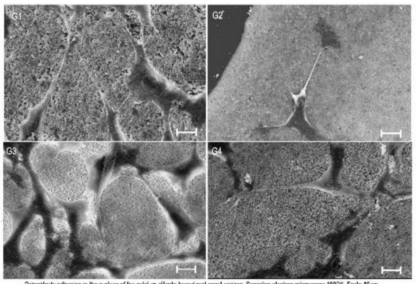

Osteoblasts adhesion to the surface of the calcium silicate-based root canal sealers. Scanning electron microscopy 1000X. Scale 10µm

**Conclusions:** These findings suggest that the BioC Sealer Ion may have potential advantages for clinical use, as it promotes favorable interactions with osteoblast cells. Further research is needed to explore the underlying mechanisms responsible for these observed effects.

**P075**

### ADRB2/aCGRP-dependent induction of pro-angiogenic factors in periosteal cells by norepinephrine

*Jan Sevecke[1], Paul Knapstein[1], Ruben Augustin[1], Anke Baranowsky[1], Johannes Keller[1]*
*[1]University Medical Center Hamburg-Eppendorf, Department of Trauma Surgery and Orthopedics-Experimental Trauma Surgery, Hamburg, Germany*

### Abstract Text

**Background:** The periosteum is richly vascularized and innervated by both the sympathetic nervous system and pain fibers, releasing Norepinephrine (NE) and calcitonin gene-related peptide alpha (aCGRP), respectively. In bone, NE signals primarily through the beta2-adrenergic receptor (ADRB2), which regulates osteoblast function. However, the precise impact of NE and its receptor, ADRB2, on periosteal cell function in relation to aCGRP signaling remains unclear.

**Objectives:** To assess the impact of NE signaling and its interaction with aCGRP on periosteal cells in vitro.

**Methods:** Primary periosteal cells and bone marrow-derived osteoblasts as controls were generated from femur bones of adult WT, ADRB2-/- and aCGRP-/- mice by enzymatical digestion of periosteum and flushed bone marrow, respectively. Both cell types were then differentiated in osteogenic medium with or without supplementation of NE, aCGRP and the aCGRP antagonist BIBN for short- or long-term stimulation. Alizarin red staining was used to assess matrix mineralization. The expression of osteoblast and angiogenic marker genes was monitored by qRT-PCR and/or ELISA.

**Results:** NE significantly reduced matrix mineralization of bone marrow-derived osteoblasts, whereas osteogenic differentiation and expression of osteoblast markers in WT periosteal cells remained normal. However, in periosteal cells, NE induced the expression of the pro-angiogenic factors hypoxia-inducible factor 1-alpha (HIF1A), vascular endothelial growth factor A (VEGFA), and aCGRP in an ADRB2-dependent manner. In aCGRP-deficient periosteal cells, NE treatment failed to induce HIF1A and VEGFA, which was also observed when cultures were co-stimulated with the aCGRP antagonist BIBN. In turn, aCGRP synergistically enhanced the stimulatory effect of NE on the expression of HIF1A and VEGFA.

**Conclusions:** The findings collectively indicate that NE/aCGRP signaling plays an important role in regulating the angiogenic properties of periosteal cells, which may be necessary for proper callus vascularization.

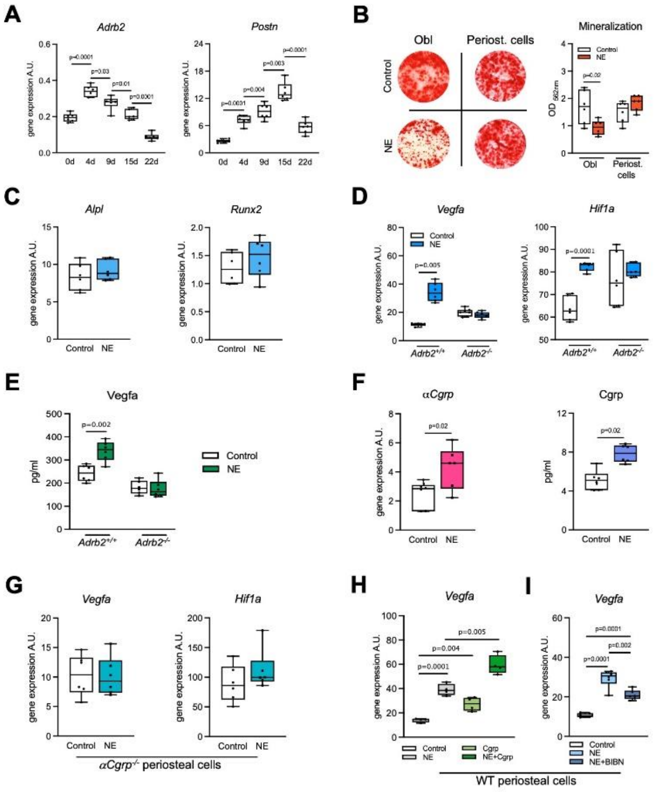

**Figure 1.** ADRB2/aCGRP signaling induces pro-angiogenic factors in periosteal cells. **A)** Expression of *ADRB2* and the periosteal marker periostin (*POSTN*) in primary periosteal cells at the indicated time points of differentiation. **B)** Representative alizarin red stained images of bone marrow-derived osteoblasts or primary periosteal cells and quantification of extracellular matrix mineralization following 15 days of osteogenic differentiation with/without norepinephrine (NE). **C)** Expression of the indicated genes in WT periosteal cells derived from femur at day 15 of differentiation stimulated with/without NE for 6 hours. **D)** Expression of the indicated genes in WT or *ADRB2*-deficient periosteal cells at day 15 of differentiation stimulated with/without NE for 6 hours. **E)** ELISA of VEGFA levels in the supernatant of the same cultures. **F)** Expression of *aCGRP* by qRT-PCR in WT periosteal cells derived from femur after stimulation with NE for 6 hours (left) and CGRP protein concentrations in the supernatant of the same cultures measured by ELISA (right). **G)** Expression of *VEGFA* and *HIF1A* in *aCGRP*-deficient periosteal cells stimulated with NE for 6 hours. **H)** Expression of *VEGFA* in WT periosteal cells stimulated with NE, CGRP, or both, for 6 hours. **I)** Expression of *VEGFA* in WT periosteal cells stimulated with NE and/or the CGRP receptor antagonist BIBN for 6 hours. One or two-way ANOVA followed by Tukey's post-hoc-test and two-tailed Student's t-test were performed for data analysis. All experiments were approved by the local animal care committee.

**P076**

### Biological Potential And Therapeutic Effectiveness Of Ginkgetin On Osteoporosis And Related Complications Through Its Effects On Osteoblast Differentiation And Other Molecular Mechanism

Dinesh Kumar Patel[1], Kanika Patel[2]
[1]Sam Higginbottom University of Agriculture- Technology And Sciences, Faculty of Health Sciences, Prayagraj, India ; [2]Department of Pharmaceutical Sciences- SHUATS, Faculty of Health Sciences, Prayagraj, India

**Abstract Text**

**Background: H**erbal medicines have been used for the treatment of human disorders, and globally it is accepted for the treatment of human disorders due to its numerous health beneficial aspects potential in medicine, including anti-bacterial, anti-fungal, anti-cancer, anti-viral, anti-parasitic, and anti-inflammatory activity. Herbal medicines have been used in the world for the treatment of human ailments since very old time.

**Purpose:** Scientific research work data signified the biological potential of ginkgetin in medicine for the treatment of different types of inflammatory disorders.

**Methods:** In order to investigate the biological potential of ginkgetin in medicine, here in the present work scientific data have been analyzed. Biological potential of ginkgetin in medicine have been investigated for their therapeutic effectiveness on osteoporosis and related complications. Other pharmacological activities of ginkgetin have also been investigated in medicine for their effectiveness against bone disorders.

**Results:** Scientific data analysis of different research work revealed the biological importance of ginkgetin in medicine. Scientific data signified the therapeutic potential of ginkgetin in medicine, as ginkgetin increased osteoblast differentiation in the scientific investigation assessed by alkaline phosphatase activity, collagen synthesis, and mineralization. Further, ginkgetin also revealed significant biological potential in medicine for the treatment of osteoporosis. **Conclusion(s):** Scientific data analysis of the present work revealed the therapeutic potential of ginkgetin on bone diseases.

**P077**

**Detection and characterization of osteoblasts in fresh and deep frozen bone fragments and their potential for bone regeneration**

Ivana Zimić[1], Marija Zekušić[1], Jelena Martinčić[2], Iva M. Tolić[2], Marina Bujić Mihica[1], Dominik Puljić[1], Slaven Babić[3], Tiha Vučemilo[1], Zlata Sošić[4], Beata Hallasy[5], Adela Štimac[5], Snježana Ramić[6], Tamara Dolenec[1], Dinko Vidović[7]
[1]Sestre milosrdnice University Hospital Center, Department of Transfusion and Regenerative Medicine, Zagreb, Croatia ; [2]Ruđer Bošković Institute, Division of Molecular Biology, Zagreb, Croatia ; [3]Sestre milosrdnice University Hospital Center, Institute of General Traumatology and Pelvic Fractures, Zagreb, Croatia ; [4]University of Turin, Department of Clinical and Biological Sciences, Turin, Italy ; [5]University of Zagreb- Centre for Research and Knowledge Transfer in Biotechnology, Laboratory for Immunochemistry and Biochemistry, Zagreb, Croatia ; [6]University Hospital Center Sestre Milosrdnice, Department of Oncological Pathology and Clinical Cytology 'Ljudevit Jurak', Zagreb, Croatia ; [7]Sestre milosrdnice University Hospital Center, Department of Sports Traumatology, Zagreb, Croatia

**Abstract Text**

**Introduction:** Osteoblasts, originating from mesenchymal cells, possess proliferative, endocrine and mineralizing capabilities, playing a crucial role in the development, remodeling and regeneration of bone tissue. Identity, purity, potency and safety are crucial aspects in the detection and characterization of osteoblasts from both fresh and deep-frozen bone fragments.

**Purpose:** The purpose of this research was to compare the characteristics of isolated osteoblasts from fresh and frozen bone fragments (with and without dimethyl sulfoxide, DMSO). The aim was to identify osteoblasts through the expression of the Runx2 antibody, determine purity through the analysis of DMSO residues, proliferative potential using the Ki67 marker and finally, verify safety through cellular cytogenetic analysis.

**Methods:** Cell culture was enzymatically derived from fresh femoral head fragments and those frozen at -80 °C for 15 and 30 days, with or without DMSO. Detection and characterization of osteoblasts were conducted using the following methods: immunofluorescence (Runx2 and cytogenesis), immunohistochemistry (Ki67) and high-performance liquid chromatography (DMSO residues).

**Results:** Osteoblasts were successfully isolated from both fresh and frozen bone fragments. In all cases, the cells exhibited positive results for Runx2 and mitotic processes were observed with high fidelity. Characteristics of isolated cells are presented in the table below.

| | *Bone samples* | *Cultivation period (days)* | *Number of isolated cells* |
|---|---|---|---|
| bone | Fresh | 7 | 1545000 |
| | 15 days + DMSO | 24 | 970000 |
| | 15 days - DMSO | 31 | 1025000 |
| | 30 days + DMSO | 22 | 1611000 |
| | 30 days - DMSO | 30 | 937500 |

The results of quadruple rinsing indicate that reduction of 10% DMSO in bone grafts reached 0,0033% DMSO. The cells demonstrated positive expression of the proliferation marker Ki67 within the range of 42-69 %.

Conclusion: Despite varying times needed to achieve 80% confluence, osteoblasts isolated from fresh and frozen bone can be successfully detected and characterized. It is presumed that the bone matrix protects osteoblasts from extremely low temperatures, enabling their survival.

**P078**

**Treatment with Orally-Active NOTUM Inhibitor LP-862993 Increases Cortical Bone Thickness in Mouse Midshaft Femurs and Lumbar Vertebrae**

*Robert Brommage[1], Melanie K. Shadoan[1], Jeff Liu[1], Sabrina Jeter-Jones[1], David R. Powell[1], Vikki K. Lombardo[2]*
*[1]Lexicon Pharmaceuticals, Metabolism, The Woodlands, United States ; [2]Lexicon Pharmaceuticals, Medicinal Chemistry, The Woodlands, United States*

**Abstract Text**

The secreted enzyme NOTUM inactivates WNTs by removing palmitoyl lipid moieties required for Frizzled receptor binding and activation. *Notum* gene KO mice have elevated cortical bone thickness and strength without trabecular bone changes. Treating young adult mice with NOTUM small molecule inhibitors or neutralizing antibodies increases cortical bone thickness and strength by stimulating endocortical bone formation (Brommage et al., *Bone Research* 7:2; 2019).

Here, we describe the effects of LP-862993, a novel orally-active small molecule NOTUM inhibitor, delivered to healthy male mice starting at 12 weeks of age. Studies were approved by Lexicon's IACUC.

<u>Study 1</u>: After 8 days of 100 mg/kg treatment by once-daily oral gavage, serum PINP levels (a biomarker of bone formation) were elevated 40% (P < 0.01).

<u>Study 2</u>: After 8 weeks of 30 or 100 mg/kg given by once-daily oral gavage, midshaft femur cortical thickness, measured by microCT, was increased by 10% and 13%, respectively (P < 0.001 for each dose, compared to the control group). MicroCT analyses of the fifth lumbar vertebral body showed no changes in total volume or trabecular BV/TV. However, treatment increased LV5 cortical bone BV/TV by 7% (low dose, P = 0.11) and 10% (high dose (P = 0.02) compared to the control group.

<u>Study 3</u>: After 4 weeks of 100 mg/kg given by either once-daily oral gavage or by admixture into purified mouse diet (food consumption was measured 3 times each week), midshaft femur cortical thickness increased by 12% in both groups (P < 0.01 for each dosing regimen, compared to the control group). For LV5, treatment increased cortical bone BV/TV by 7% (gavage dosing, P = 0.01) and 5% (diet dosing, P = 0.04). Treatment did not affect LV5 trabecular BV/TV (P = 0.98).

LP-862993 (figure) is a novel NOTUM inhibitor from a novel chemotype.

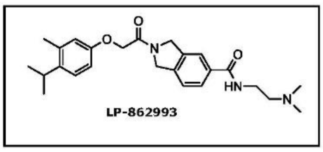

**P079**

**Modulating the osteogenic potential of falciform ligament-derived mesenchymal stromal cells**

*<u>Carla Ferreira-Baptista</u>[1,2,3,4], André Queirós[5], Rita Ferreira[3], Maria Helena Fernandes[1,4], Pedro Sousa Gomes[1,4], Bruno Colaço[2,4,6,7]*
*[1]Faculty of Dental Medicine- University of Porto, BoneLab - Laboratory for Bone Metabolism and Regeneration, Porto, Portugal ; [2]University of Trás-os-Montes e Alto Douro UTAD, Centre for the Research and Technology of Agro-Environmental and Biological Sciences CITAB, Vila Real, Portugal ; [3]University of Aveiro, REQUIMTE/LAQV- Department of Chemistry, Aveiro, Portugal ; [4]University of Porto, Requimte/LAQV, Porto, Portugal ; [5]SCIVET, Grupo Breed, Paredes, Portugal ; [6]University of Trás-os-Montes and Alto Douro UTAD, Associate Laboratory for Animal and Veterinary Sciences AL4AnimalS, Vila Real, Portugal ; [7]University of Trás-os-Montes and Alto Douro UTAD, CECAV—Animal and Veterinary Research Centre UTAD, Vila Real, Portugal*

**Abstract Text**

**Introduction:** Adipose tissue-derived stromal cells (ADSCs) are a versatile population of multipotent cells with a variety of promising characteristics, such as accessibility, abundance, rapid proliferation, low immunogenicity, and differentiation capacity. These cells can be obtained from different anatomical locations, such as subcutaneous, omentum, perirenal, periovarian and mesenteric depots. Notably, the falciform ligament has recently emerged as a potential depot for ADSCs' isolation. However, the osteogenic capacity of falciform ligament-derived ADSCs (FL-ADSCs) remains unexplored and has not been previously characterized.

**Purpose:** The aim of this study is to characterize the functionality of this cell population as it undergoes osteogenic induction via a classical method with dexamethasone, β-glycerophosphate and ascorbic acid (DEX), or retinoic acid (RA). The research employs a comparative approach, contrasting the outcomes with those of ADSCs isolated from a representative visceral region, as a control. Canine cells were used within this experimental research.

**Methods:** All experimental procedures were approved by the institutional ethics committee [15-CE-UTAD-2021]. Adipose tissue from FL and periovarian visceral region (control) was collected from 6 healthy dogs. ADSCs were isolated through an enzymatic dissociation process and cultured in DEX and RA conditions. The obtained cultures were characterized at different periods for proliferation, morphology, and osteogenic activity. Comparison between groups was performed using analysis of variance and Tukey post-hoc test ($p < 0.05$).

**Results:** Our results demonstrated a significantly higher expression of osteogenic genes such as RUNX2 (≈10 fold), COL1A1 (≈4 fold), SP7 (≈4 fold) and BGLAP, as well as cytochemical markers in cultures of ADSCs isolated from the falciform ligament. This heightened expression, particularly evident under dexamethasone (DEX) induction, significantly surpassed that observed in the control group.

**Conclusion:** It can thus be concluded, that FL-ADSCs are a relevant cell source for bone-related applications, given their high osteogenic capacity and responsiveness to inductive agents.

**P080**

**Comparative analysis of haemostatic agents' biological functionality on alveolar bone**

*Maria Guerra Gomes*[1,2,3,4,5]*, Liliana Grenho*[1,2]*, Maria Helena Fernandes*[1,2]*, Bruno Colaço*[2,3,4,6]*, Joana Barros*[5]*, Pedro Gomes*[1,2]
*[1]Faculty of Dental Medicine of University of Porto, BoneLab - Laboratory for Bone Metabolism and Regeneration, Porto, Portugal ; [2]University of Porto, Requimte/LAQV, Porto, Portugal ; [3]University of Trás-os-Montes e Alto Douro, Al4Animals - Associate Laboratory for Animal and Veterinary Sciences, Vila Real, Portugal ; [4]University of Trás-os-Montes e Alto Douro, CECAV - Animal and Veterinary Research Centre, Vila Real, Portugal ; [5]I3S - Institute for Research and Innovation in Health, Biocomposites, Porto, Portugal ; [6]University of Trás-os-Montes e Alto Douro, CITAB - Centre for the Research and Technology of Agro-Environmental and Biological Sciences, Vila Real, Portugal*

**Abstract Text**

**Background/Introduction:** Dental practitioners encounter diverse scenarios necessitating meticulous bleeding control, ranging from routine dental extractions to more complex oral surgeries. Achieving effective haemostasis is integral to the success of dental procedures, ensuring optimal clinical outcomes and patient comfort. Among the crucial therapeutic options for managing bleeding is the utilization of topical haemostatic sponges. These agents can persist at the site of application for up to 8 weeks, which could affect osteogenesis. Consequently, it is imperative to scrutinize their potential modulatory effects on the bone tissue, to ensure a comprehensive understanding of their full biological impact.

**Purpose:** As such, the aim of this study was to conduct an *in vitro* characterization of various commercially available gelatine-based haemostatic agents, specifically, Hemospon®, Roeko® and Octocolagen®, focusing on their impact on human osteoblastic cells.

**Methods:** Haemostatic sponges' leachables were prepared in Minimum Essential Medium (MEM), per the ISO EN 10993-17 standard. The human osteoblastic MG-63 cells were cultured in growth medium for 24 hours, subsequently allocated into experimental groups exposed to leachables, at concentrations of 50% and 12,5% and a control group without exposure. The cell cultures were then assessed for cell viability and proliferation (metabolic activity (MTT) and DNA quantification), alkaline phosphatase (ALP) activity, mitochondrial and cellular morphology, as well as ALP histochemical staining and collagen content, at different timepoints.

**Results and conclusion:** Upon data analysis, cultures exposed to haemostatic agents' leachabels appeared to proliferate similarly to the control, displaying minimal cell death and unaffected activity. Moreover, normal cellular morphology, and collagen production were observed across all groups. Notably, Roeko® leachables' induced significantly higher ALP activity compared to the other agents, suggesting an increased osteogenic potential and a prospective application to prime bone tissue healing in clinical applications.

**P081**

**Phosphate Signaling Molecule and Trps1 Transcription Factor: Regulatory Feedback Loop in Cells Producing Mineralized Extracellular Matrix (ECM)**

*Mairobys Socorro[1], Kaoru Fujikawa[1], Nadine Robert[1], DOBRAWA NAPIERALA[1]*
*[1]University of Pittsburgh, Oral and Craniofacial Sciences- Center for Craniofacial Regeneration, Pittsburgh, United States*

**Abstract Text**

Phosphate ($P_i$) is a signaling molecule regulating differentiation and function of cells producing mineralizing ECM. Studies have shown that promoters of $P_i$-responsive genes are enriched in GATA binding sites. Trps1 is a GATA-type transcription factor involved in development and homeostasis of skeletal and dental tissues. We have demonstrated that overexpression of Trps1 in odontoblasts (*Col1a1-Trps1* transgenic mice) represses one of the $P_i$-activated genes – *SerpinB2*, while the binding of Trps1 to the *SerpinB2* promoter differs depending on the extracellular $P_i$ levels. We hypothesize that $P_i$ regulates the activity of Trps1. We used 17IIA11 osteogenic cell line that express high levels of osteogenic transcription factors (Runx2 and Osx) as an *in vitro* model of mineralization, *Col1a1-Trps1* transgenic mice, and $Trps1^{Col1a1}$ conditional knockout (cKO) mice with postnatal deletion of Trps1 in osteoblasts, odontoblasts and cementoblasts. Using proximity ligation assay in *Col1a1-Trps1* P0 mouse mandible and co-immunoprecipitation in 17IIA11 cells, we have uncovered that Trps1 directly interacts with Osx transcription factor under normophosphatemic conditions (0.8mM $P_i$). However, this interaction is diminished under hyperphosphatemic conditions (5mM $P_i$) *in vitro*. $P_i$ levels had no effect on *Trps1* expression. Interestingly, *Trps1* deletion results in decreased expression of *Osx in vitro* and *in vivo*, and decreased expression of several $P_i$-activated genes, including *Dmp1*, which is also regulated by Osx. Restoring Osx expression in Trps1-deficient cells resulted in overactivation of *Dmp1* expression as compared to unmodified 17IIA11 cells, suggesting that Trps1 prevents the overactivation of the Osx-mediated transcription. In addition, Trps1-deficient cells lost their ability to respond to extracellular $P_i$, as demonstrated by reduced levels of pErk1/2 and expression of several $P_i$-stimulated genes. Together, these data demonstrate that $P_i$ regulates the activity of the Trps1 transcription factor, which controls the cellular responses to $P_i$ at the gene expression level as well as cellular ability to recognize available $P_i$.

**P082**

**ISWI-mediated chromatin remodeling directing stromal cell differentiation**

*Kaja Madsen*[1], *Alexander Rauch*[1]
[1]*University of Southern Denmark, Department of Clinical Research, Odense, Denmark*

**Abstract Text**

**Background and aim:** Linage determination of stromal progenitor cells is a complex process involving activation of gene programs that drive differentiation and repression of genes maintaining multipotency. Rewiring of lineage specific gene expression is controlled by multiple mechanisms such as chromatin accessibility. The latter is modulated by ATP-dependent chromatin remodeling complexes. Some chromatin remodeling complexes have shown to be important for determining stromal progenitor fate, with SWI/SNF being reported to be pro-adipogenic and INO80 to be pro-osteogenic. However, the effect of ISWI on stromal progenitor fate has not been described, why I in this study aim to elucidate the function of ISWI during osteogenic and adipogenic differentiation.

**Material and methods:** I compared *in vitro* differentiation potential of human stromal progenitors exposed to pharmacologic or genetic inhibition of SMARCA5, the ATPase subunit of ISWI, using ED2-AD101, siRNA mediated knockdown, and stable CRISPR-Cas9 knockout. *De novo* bone formation of control cells and CRISPR-Cas9 mediated SMARCA5 knockout cells was tested in ectopic bone formation assays. Furthermore, RNA-seq, DNase-seq and ChIP-seq of ISWI, H3K27ac and MED1 were performed to test genome wide association of ISWI with lineage-selective change in gene expression and enhancer activity.

**Results and conclusion**: *In vivo* experiments showed decreased osteogenic potential for stromal progenitor cells upon CRISPR-Cas9 mediated knockout (*FoldChange:0.7±0.1, p=0.003),* transient siRNA knockdown (*FoldChange: 0.3±0.13, p = 0.00005*) and pharmacological inhibition (*FoldChange: 0.3±0.04, p = 0.00007* ) of SMARCA5. Furthermore, a correlation between change in ISWI binding and accessibility of chromatin (DNase-seq) during differentiation was found (*Correlation:0.7,* $p=2.2*10^{-16}$ *for osteogenesis and correlation:0.9,* $p=2.2*10^{-16}$ *for adipogenesis*). Enrichment analysis showed that gene induction during osteoblast differentiation ($p = 9.9*10^{-13}$) as well as adipocyte differentiation ($p = 9.5*10^{-33}$) was associated with nearby gain in ISWI binding (+/- 50,000 bp from TSS).

**P083**

**Connecting HIPPO signaling to chromatin action during early osteoblast and adipocyte differentiation**

*Atenisa Caci*[1], *Alexander Rauch*[1]
[1]*University of Southern Denmark, Department of Clinical Research, Odense C, Denmark*

**Abstract Text**

**Background**: Cellular differentiation of bone marrow stromal cells (BM-MSCs) into osteoblasts or adipocytes is directed by lineage-selective transcription factor networks that control the transition from progenitor gene program to the differentiated cell type. A network of about 200 transcription factors (including TEAD1) is pivotal for osteoblast while efficiently inhibiting fat cell differentiation.

**Purpose:** Identify transcriptional mechanisms of the HIPPO signaling contributing to lineage commitment of BM-MSCs.

**Methods:** Enhancer activity, gene expression and binding profiles of YAP. TEAD1 and RUNX2 during early differentiation of BM-MSCs have been established using ChIP and RNA-seq. Quantification of the impact of genetic and pharmacological disruption of HIPPO components on osteoblast and adipocyte differentiation in BM-MSCs.

**Results:** Putative active enhancers in BM-MSCs are associated with YAP chromatin binding and YAP binding dynamics resemble the changes in enhancer activity during early differentiation (FigureA). YAP binds stronger to the chromatin during osteoblast differentiation and its occupancy is reduced during adipocyte differentiation. Changes in TEAD1 and RUNX2 binding intensity as predictors for gain or loss of YAP recruitment, pointed out TEAD1 binding dynamics as the strongest predictor for enhancers gain activity during osteoblast differentiation as well as for enhancers restrained during adipogenic differentiation (pvalue:Obup<2.2e-16,Obdown=1.806e-13,Adup<2.2e-16,Addown<2.2e-16) (FigureB).Immunostaining showed clear lineage selective dynamics in nuclear localization of YAP(Cohen's estimation: 1dAd= -0.1; 1dOb = 1.31(large) (FigureC) and reduction in nuclear YAP protein levels correlate with reduced genome-wide occupancy of both YAP1 and TEAD1 upon adipogenic stimulation, indicating cofactor-dependent dissociation. YAP binding in cells exposed to osteogenic condition was associated with WNT-signaling, while loss of YAP binding upon adipogenic stimulation correlates with repression of those genes. Pharmacological inhibition and genetic ablation of YAP-TEAD1 strongly reduces mineralization.

**Conclusion:** From our genome wide binding pattern, we suggest TEAD1 as the major sequence specific rheostat of Hippo signaling that is implicated in lineage determination of BM-MSCs.

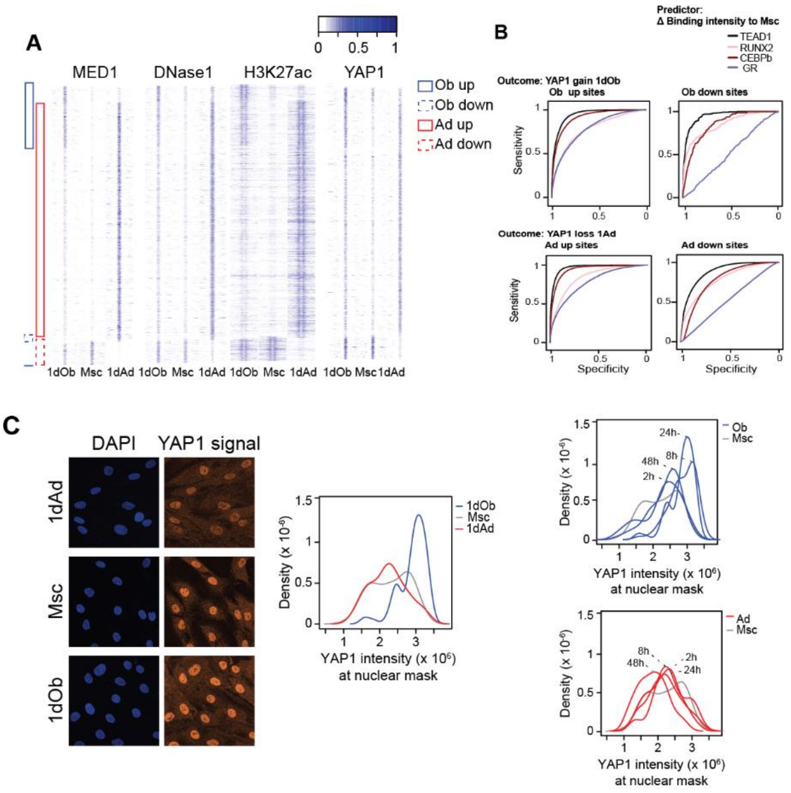

**ND-P013**

**3D bone-like constructs without artificial scaffolds can be analysed by non-destructive methods**

*Arooj Munir[1], Lene B Solberg[2], Torben Hildebrand[1], Janne E Reseland[1,3], Liebert P Nogueira[3]*
*[1]University of Oslo, Department of Biomaterials- Institute of Clinical Dentistry, Oslo, Norway ; [2]Oslo University Hospital, Division of Orthopaedic Surgery, Oslo, Norway ; [3]University of Oslo, Oral Research Laboratory- Institute of Clinical Dentistry- Faculty of Dentistry, Oslo, Norway*

**Abstract Text**

In rotary cell culture (RCC) systems primary human osteoblasts (hOBs) can form three-dimensional (3D) spheroids with bone-like structure without the addition of artificial scaffolds, generating dynamic in vitro models of artificial tissue. RCC has shown to enhance cell aggregation, extracellular matrix formation, cell-cell, and cell-extracellular matrix (ECM) interaction, as well as osteoblast differentiation to osteocyte-like cells. These artificial bone tissue models with complex 3D cellular structures are useful tools in many applications, like in vitro drug screening and basic research.

Non-destructive analyses of these spheroids by e.g. X-ray microtomography (micro-CT) or synchrotron imaging cannot yet rival traditional histology for differentiation of tissue types and cell components due to lack of contrast. Therefore, contrast in osteospheroids were enhanced using lead(II) acetate (pbII) and phosphotungstic acid (PTA), followed by comprehensive investigation using contrast enhanced synchrotron radiation micro-CT (SRµCT) scanning and compared to histological staining and stacking of 2D images.

SRµCT imaging revealed exquisite 3D details of ECM and the arrangement of cells throughout the osteospheroid. The artificial histological colouring, mimicking the HE staining, allowed visualization of cellular density, and cells and ECM distribution in the osteospheroids, which were confirmed by histological staining. Additionally, contrast enhancement and scanning had no negative effect on histological staining enabling additive analyses, like 3D quantification of cellular structures followed by a detailed identification of molecular mechanisms by immunohistology of sections.

In conclusion, non-destructive 3D visualization allowing virtual histology of osteospheroids by contrast enhanced SRµCT is a potential tool for better visualization and assessment of ultra-structures in osteospheroid including cells and ECM.

**ND-P014**

**Enhancing human osteoblast to osteocyte-like cell differentiation using 25-hydroxyvitamin D3 and All-trans retinoic acid combination in-vitro**

*Arooj Munir[1], Anne E Agger[1], Athina Samara[1], Janne E Reseland[1], Lene B Solberg[2]*
*[1]University of Oslo, Department of Biomaterials- Institute of Clinical Dentistry, Oslo, Norway ; [2]Oslo University Hospital, Division of Orthopaedic Surgery, Oslo, Norway*

**Abstract Text**

The combined effects of 25-hydroxyvitamin D3 (25(OH)D3) and all-trans retinoic acid (ATRA) on the differentiation of primary human osteoblasts (hOBs) to osteocyte-like cells remains largely unexplored. We therefore compared the effect of various dosage of 25(OH)D3 and ATRA, alone and in combinations, on hOBs differentiation for up to 21 days. hOBs growth, alkaline phosphatase (ALP)

activity (cell bound and secreted), along with the expression of bone-related markers at both genetic level (RT-PCR) and protein level (multiplex immunoassays) were evaluated. 25(OH)D3 and ATRA enhanced the differentiation of hOBs, however, the combination of $10^{-6}$ M 25(OH)D3 and $10^{-5}$ M ATRA was found to have the most profound effect by reducing the growth and enhancing the differentiation. This was evident from an increase in the relative expression of osteoblast markers (ALP, collagen type I alpha 1 (COL1A1), osteopontin, and osteocalcin), and upregulation of osteocyte markers such as podoplanin (E11), dentin matrix acidic phosphoprotein 1 (DMP1), sclerostin (SOST), and fibroblast growth factor 23 (FGF23). Furthermore, 25(OH)D3 and ATRA regulated the expression of retinoic acid receptor alpha (RARα), retinoid X receptor beta (RXRβ), and vitamin D receptor (VDR) in osteoblasts during differentiation. In conclusion, the present study demonstrates that a combination of $10^{-6}$ M 25(OH)D3 and $10^{-5}$ M ATRA enhanced the differentiation of hOBs to osteocyte-like cells in vitro.

**Poster Sessions: Osteoclasts and bone resorption**

**P084**

**Histological analysis of the influence of acute and chronic stressors in spontaneously hypertensive rats on the morphofunctional characteristics of long bones**

*João Paulo Mardegan Issa*[1], *Marina Paulini*[2], *Dimitrius Pitol*[3], *Glauce Crivelaro*[3], *Ana Paula Macedo*[4]
[1]*Faculdade de Odontologia de Ribeirão Preto- Usp, Departamento de Biologia Básica e Oral, Ribeirão Preto, Brazil ;* [2]*Faculdade de odontologia de Bauru, Prótese e materiais dentários, Ribeirão Preto, Brazil ;* [3]*Faculdade de odontologia de Ribeirão Preto, Biologia Oral, Ribeirão Preto, Brazil ;* [4]*Faculdade de odontologia de Ribeirão Preto, Prótese e materiais dentários, Ribeirão Preto, Brazil*

**Abstract Text**

Arterial Hypertension (AH) is part of the group of cardiovascular diseases that represent the highest percentage of causes of mortality. Evidence suggests that psychosocial factors may play an important role in the development of high blood pressure. Exposure to chronic stress has been considered a risk factor for this chronic disease. The literature also indicates that hypertension is associated with increased bone mineral loss, although this requires further investigation. Due to the relevance of the topic, much has been studied on the subject, although little attention has been given to the association of emotional factors with blood pressure. Therefore, this study aimed to investigate the effect of varied acute and chronic stress associated with hypertension on the skeletal system, through morphofunctional and metabolic assessments (Histology). Male Hannover rats (n=40) and SHR rats (spontaneous hypertensive rats) (n=40) were used, randomly divided into three large groups: control group without stress (S); group with acute stress (A); group with varied chronic stress (C). There were the following subgroups in groups A and C: control group (n=8) and those subjected to stress (n=8). The rats were euthanized on the 10th day after the start of the experiment, to obtain the right tibia for histological analysis. Kruskal-Wallis was used to compare controls for the same type of animal. No significant differences were found: Normal (p=0.077) and SHR (=0.123). The Wald Test was performed in a generalized linear model to compare groups acute and chronic stress, in which no significant differences were identified between the types of rats (p=0.203) and stress/handling (p=0.157) and no interaction Type of rat vs stress/ manipulation (p=0.420). When checking the graph, there is a tendency towards an increase in the area in the manipulated control groups, for both types of rats, showing that the stress protocol generates bone resorption.

**P085**

**Rankl Contributes to Early Bone Development via the Wnt Signaling Pathway in Zebrafish**

*Yu Jiang*[1]
[1]*Jiangnan University Medical Centre, Department of General Practice, WuXi, China*

**Abstract Text**

Rankl is a key regulator of osteoclastogenesis, mainly produced by osteoblast and osteocyte cells. While previous studies have shown that rankl is the main factor inducing osteoclast differentiation, its role in early bone development is unclear. In this study, the conservation of zebrafish rankl and human homologous genes were analyzed. The effects of rankl on zebrafish osteogenic differentiation, osteoclastic differentiation, and bone development were studied using a zebrafish rankl mutant (rankl-/-) constructed with CRISPR/Cas9. The observed rankl homology in zebrafish, humans, and mice was very high. Additionally, bone, alizarin red, and tartaric acid phosphatase staining showed that osteogenic ability was weakened in rankl-/-, while bone mineralization and osteoclasts were reduced during development. In adult zebrafish, rankl-/- bone was enhanced and bone mass was increased compared to the wild type. Moreover, regenerated fins of rankl-/- grew faster and osteoclasts decreased, while rankl-capped mRNA could rescue the disorder caused by rankl deletion. Transcriptome sequencing further showed that the Wnt signaling pathway was substantially enriched, and its inhibitor could save the bone retardation phenotype after rankl deletion. This study provides a new theoretical basis for deeply understanding the role of rankl in early bone development and for the clinical treatment of bone-related diseases.

**P086**

**Identification of a novel osteoclast progenitor cell type with higher multinuclear capacity**

*Erika Yamashita*[1], *Ishii Masaru*[1]
[1]*Osaka university, Immunology and Cell Biology, Suita, Japan*

**Abstract Text**

**Background:** Osteoclasts are multinucleated giant cells characterized by their bone-resorbing activity, and they have been shown to differentiate from hematopoietic stem cells via granulocyte-monocyte progenitor cells (GMPs), monocyte-dendric cell progenitor cells (MDPs) and monocyte. Recent accumulating evidence suggesting the plasticity of hematopoietic lineage cells toward fate determination may indicate that MDPs or monocytes may not be the exclusive OC progenitors. In this study, we hypothesize that the OC differentiation pathway might be more complicated than shown in conventional studies.

**Purpose:** We comprehensively searched the myeloid progenitors having osteoclastogenic properties to clarify the whole picture of the OC differentiation pathway.

**Methods and Results:** The limiting dilution assay showed that a bone marrow cell population corresponding to basophil mast cell progenitor cells (BMCPs) had sufficient osteoclastogenic properties under the RANKL administration similar to GMPs and MDPs. Furthermore, intravital bone marrow imaging revealed that BMCPs can indeed differentiate into OCs with bone-resorbing ability under biological conditions. In addition, *in vitro* time-lapse imaging showed that the BMCPs can generate enlarged multinuclear OCs with a higher bone-resorbing activity than the conventional MDP-derived OCs (see the figure for numerical data and statistical evaluation). Furthermore, RNA sequencing detected the genes related to fusion or multinucleation (*Marco*, *Cav1*, *Cd81*, *Aif1l etc.*) were significantly increased in BMCPs compared to MDPs and GMPs.

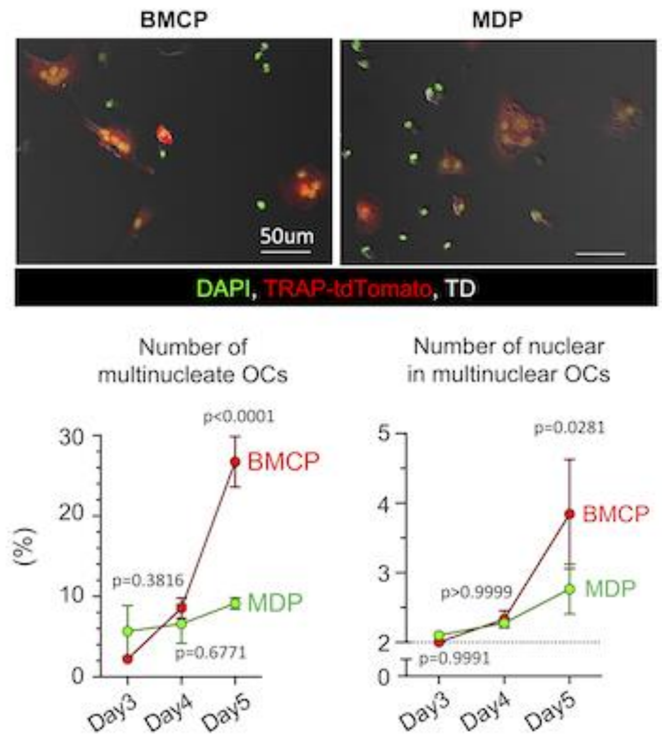

**P087**

**The anti-diabetic drug pramlintide protects against ovariectomy-induced osteoporosis by inhibiting bone resorption in a calcitonin receptor-dependent manner.**

*Shan Jiang[1], Weixin Xie[1], Judith Kokot[1], Paul Knapstein[1], Antonia Donat[1], Anke Baranowsky[1], Johannes Keller[1]*
*[1]University Medical Center Hamburg-Eppendorf, Department of Trauma and Orthopedic Surgery, Hamburg, Germany*

**Abstract Text**

**Background:** Pramlintide represents a synthetic analogue of pancreas-derived Amylin and is used clinically to treat type 2 diabetes. It is widely accepted that the calcitonin receptor (CTR) is an indispensable component of the Amylin-specific receptors, which is highly expressed on osteoclasts and mediates the inhibitory effect of calcitonin on bone resorption. However, this view was challenged in a previous study, in which Amylin and CTR-deficient mice displayed distinct bone phenotypes, indicating that Amylin acts through an unidentified receptor other than CTR.

**Purpose:** First, to investigate the ability of pramlintide to limit bone resorption in an osteoporosis model. Second, to delineate whether CTR is the key receptor mediating the skeletal effects of pramlintide.

**Methods:** 8-week-old female WT and CTR-deficient mice were ovariectomized (OVX) and treated with pramlintide for 4 weeks. The spine and femur were harvested for micro-CT and non-decalcified histology. Bone marrow-derived osteoclasts were generated and treated with pramlintide for in vitro experiments.

**Results:** In WT mice, OVX-induced bone loss was completely restored in the vertebral column and partially blunted in the distal femur with pramlintide treatment. In contrast, the beneficial effects of pramlintide were completely abolished in CTR-deficient mice. Cellular histomorphometry revealed significantly elevated osteoclast parameters in WT OVX mice, which were normalized by pramlintide in a CTR-dependent manner, while osteoblast numbers were not affected. In vitro, pramlintide potently inhibited the formation of WT but not CTR-deficient osteoclasts. Genome-wide transcriptome profiling showed that the expression of key genes associated with osteoclastogenesis was regulated by pramlintide-CTR signaling.

**Conclusion:** Our results provide first evidence that the anti-diabetic drug pramlintide is effective to prevent bone loss in OVX-induced osteoporosis via a robust suppression of osteoclastogenesis in a CTR-dependent manner. Together, these findings indicate that in addition to its beneficial effects on glucose metabolism, pramlintide may also preserve bone integrity in patients with type 2 diabetes and postmenopausal osteoporosis.

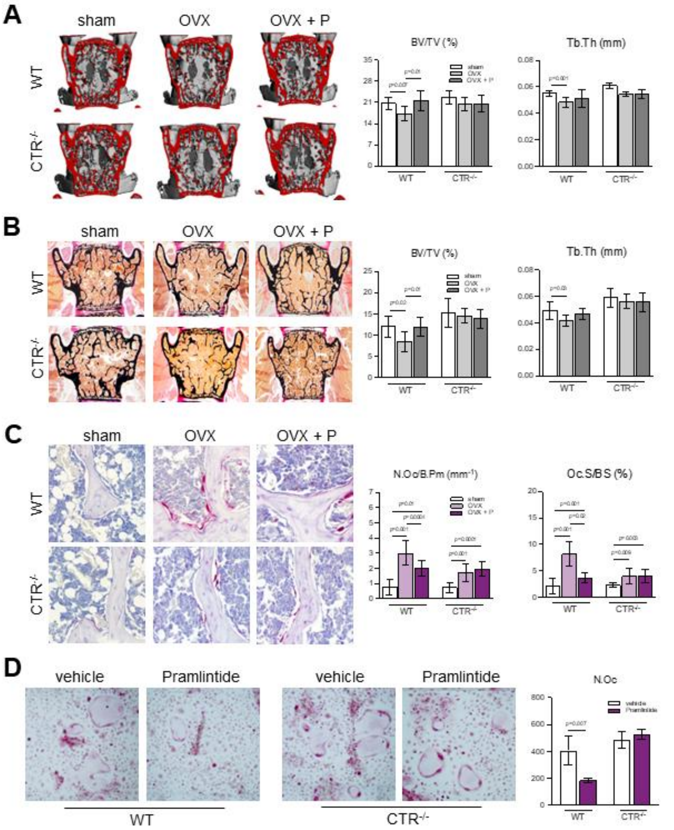

**Figure 1.** Pramlintide inhibits bone resorption in a CTR-dependent manner. **A)** Representative µCT images of the L3 vertebrae in the indicated mice 4 weeks after OVX and daily treatment with pramlintide or vehicle. The quantification of bone volume per tissue volume (BV/TV) and trabecular thickness (Tb.Th) is indicated on the right. **B)** Von Kossa/van Gieson staining of undecalcified vertebra sections (L4) and histomorphometric evaluation of BV/TV and Tb.Th in the same mice. **C)** Cellular histomorphometry of bone resorption markers in the same mice. Osteoclast numbers/bone perimeter (N.Oc/B.Pm), osteoclast surface/bone surface (OcS/BS). For A-C, n = 6-8 mice per group. **D)** Trap-activity staining of bone marrow cells of the indicated genotypes undergoing osteoclastic differentiation with/without continouspramlintide supplementation. The quantification of TRAP-positive, multinucleated (n>3 nuclei) osteoclasts after 6 days of differentiation is indicated on the right. Two-way ANOVA followed by Tukey's post-hoc-test and two-tailed Student's t-test were performed for data analysis. All experiments were approved by the local animal care committee.

**P088**

### Investigating miR-Mediated Interactions Among SERPINB1 and NFATc1 in Postmenopausal Osteoporosis Pathogenesis.

*Simran Kaur[1], Vandana Dhiman[1], Mani Sangar[1], Ravi Pratap Barnwal[2], Naresh Sachdeva[1], Gurpal Singh[3], Sanjay Kumar Bhadada[1]*
*[1]Postgraduate Institute of Medical Education and Research, Endocrinology, Chandigarh, India ; [2]Panjab University, Biophysics, Chandigarh, India ; [3]Panjab University, University Institute of Pharmaceutical Sciences, Chandigarh, India*

**Abstract Text**

**Background/Introduction:** Postmenopausal osteoporosis (PMO), a pervasive health challenge, demands precise markers due to limitations in traditional BMD and bone turnover markers. Addressing this gap, our study focuses on microRNAs (miRs), particularly hsa-miR-335-5p, as potential tools for improved understanding and management of PMO.

**Purpose:** Our investigation centres on hsa-miR-335-5p's role in downregulating SERPINB1, a crucial osteoclast differentiation regulator. Utilizing robust bioinformatics tools, we generate a target list, prominently featuring SERPINB1. The broader regulatory network involving hsa-miR-335-5p, SERPINB1, and NFAT1c is systematically explored for enhanced comprehension and its effect on osteoclast cells. Additionally, we delve into the interplay between miR-335-3p and RUNX2, unravelling its effects on osteoblast cells.

**Methods:** We conducted a microarray analysis on postmenopausal osteoporosis, validating identified miRNAs in a large cohort. A prior written informed consent approved by IEC was taken. After validation, MiR-335-5p was selected for in silico analysis using miRwalk, miRTarBase, miRDB, miR-pathDB, miRPath, and Human Protein Atlas. Venn diagrams refined targets, cross-referencing with bone-related genes. MG63 cell line experiments validated SERPINB1 and RUNX2 expressions. Functional enrichment and tissue-specific analyses contextualized hsa-miR-335-5p's pivotal role in bone metabolism.

**Results:** SERPINB1 and RUNX2 emerge as pivotal regulators in PMO, with SERPINB1 consistently expressed in the bone marrow. miRDB and tissue-specific analyses reveal hsa-miR-335-5p's extensive influence beyond bone tissue, supported by the Pharos database's insights into broader protein interactions. Intriguingly, SOX2's interaction with SERPINB1 is highlighted, contributing to the inhibition of osteoclast formation by downregulating NFAT1c. Our study aims to validate this interaction in MG63 cells through miR-335-5p mimic and inhibitor transfections. Overexpression of miR-335-5p suppresses SERPINB1, elevates NFATc1, indicating increased bone resorption, and significantly downregulates RUNX2, correlating with reduced bone formation.
**Conclusion:**Our study conclusively underscores the significance of hsa-miR-335-5p, SERPINB1 and NFATc1 in PMO. Proposed regulatory pathways offer potential interventions for effective management, with complex interactions providing insights for targeted validations and refined PMO management strategies.

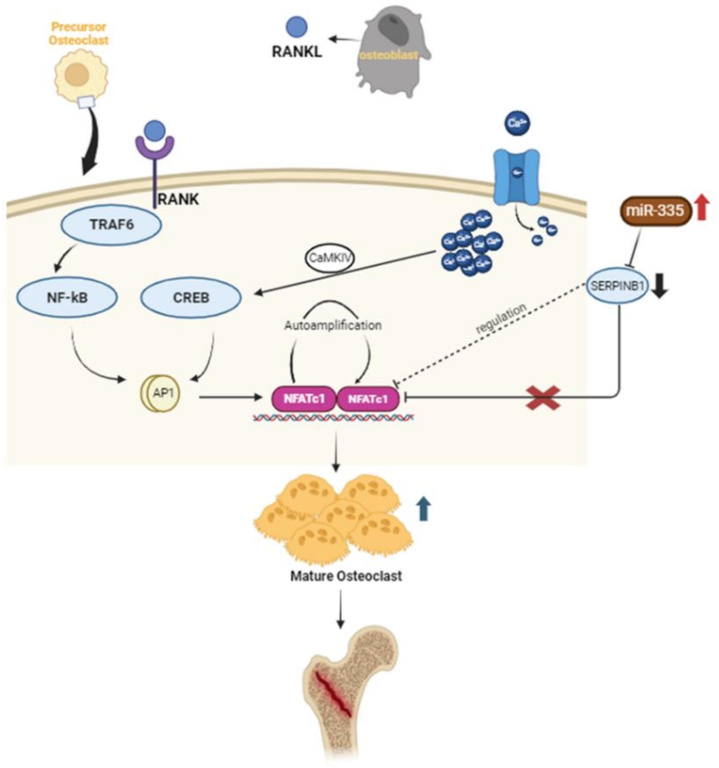

**P089**

**Osteogenic Differentiation inhibits Osteoclast Formation**

*Caya Prins[1], Merve Ceylan[2], Jolanda Hogevorst[3], Ineke Jansen[2], Irene Schimmel[4], Ton Schoenmaker[2], Teun De Vries[2]*
*[1]Academic Centre for Dentistry Amsterdam- University of Amsterdam and Vrije Universiteit, Periodontology, Amsterdam, Netherlands ; [2]Academic Centre for Dentistry Amsterdam- University of Amsterdam and Vrije Universiteit Amsterdam, Periodontology, Amsterdam, Netherlands ; [3]Academic Centre for Dentistry Amsterdam- University of Amsterdam and Vrije Universiteit Amsterdam, Oral Cell Biology, Amsterdam, Netherlands ; [4]Amsterdam University Medical Centre, Medical Biology, Amsterdam, Netherlands*

**Abstract Text**

Although it has been established that periodontal ligament fibroblasts (PLFs) play a role in both osteogenesis and osteoclastogenesis, it remains unclear how the two bone remodelling processes interact. Here, the osteogenic differentiation of PLFs and the effects of this process on the formation of osteoclasts were investigated. PLFs were obtained from extracted teeth and cultured for 21 days, of which in the presence of osteogenic medium for 0 or 21 days, or for the last 14 or 7 days of culture. After this 21 days of culturing, the cells were co-cultured with peripheral blood mononuclear cells (PBMCs) for an additional 21 days to study osteoclast formation. Alkaline phosphatase (ALP) activity, calcium concentration, and gene expression of osteogenic markers were assessed at day 21 to evaluate the different stages of osteogenic differentiation. Alizarin red staining and scanning electron microscopy were used to visualise mineralisation. Tartrate-resistant acid phosphatase (TRAcP) activity, TRAcP staining, multinuclearity, the expression of osteoclastogenesis-related genes, and TNF-α and IL-1β protein levels were assessed to evaluate osteoclastogenesis. The osteogenesis assays revealed that PLFs became more differentiated as they were exposed to osteogenic medium for a longer period of time. Mineralisation by these osteogenic cells increased with the progression of differentiation. Culturing PLFs in osteogenic medium before co-culturing them with PMBCs led to a significant decrease in osteoclast formation. qPCR revealed significantly lower DC-STAMP expression in cultures that had been supplemented with osteogenic medium. In parallel to these findings: protein levels osteoclastogenesis stimulator TNF-α were lower in cultures that were exposed to osteogenic medium. The present study shows that the osteogenic differentiation of PLFs reduces the osteoclastogenic potential of these cells. Therefore, mature and immature osteogenic cells may have different roles in maintaining bone homeostasis.

**P090**

**Dysregulated myeloid differentiation in colitis is induced by inflammatory osteoclasts in a TNFa-dependent manner**

*Maria-Bernadette Madel[1], Lidia Ibáñez[2], Thomas Ciucci[1], Julia Halper[1], Matthieu Rouleau[1], Christopher Mueller[3], Laurent Peyrin-Biroulet[4], David Moulin[5], Claudine Blin-Wakkach[1], Abdelilah Wakkach[1]*
*[1]CNRS Universite Cote d'Azur, Lp2m, Nice, France ; [2]CNRS Uniiversite Cote d'Azur, Lp2m, Nice, France ; [3]CNRS, Ibmc, Strasbourg, France ; [4]INFINY institute, Department of gastroenterology, Nancy, France ; [5]CNRS, IMoPA, Nancy, France*

**Abstract Text**

**Introduction:** Osteoporosis is a frequent complication in patients with Crohn's disease. We and other have demonstrated in murine models that colitis results in bone loss due to a dramatic increase of osteoclast activity. Besides bone resorption, osteoclasts are known to control hematopoietic niches in vivo and modulate inflammatory responses in vitro, suggesting their possible role in inflammation in vivo.

**Aim:** Therefore, we investigated whether osteoclasts participate in intestinal inflammation in chronic colitis.

**Methods:** Using 2 classical models of colitis induced by Dextran Sulfate Sodium administration in normal mice or by transfer of naïve CD4+ T-cells in Rag1-/- mice, we combined RNAseq analysis of osteoclasts, in vivo osteoclast inhibition and in vitro experiments to evaluate by cytometry and functional assays the role of osteoclasts in colitis.

**Results:** In colitic mice, osteoclast inhibition by Zoledronate or Calcitonin significantly reduced colitis severity (pVal<0.0001), and stimulation of osteoclastogenesis by RANKL worsened colitis (pVal<0.01). We found a direct link between osteoclast activity and myeloid cell accumulation in the colitic intestine ($R^2$=0.92). RNAseq analysis revealed overexpression of genes involved in the remodeling of hematopoietic stem cell niches in colitic osteoclasts compared to controls (pVal<0.01). We also demonstrated in vivo and in vitro that osteoclasts induced hematopoietic progenitor proliferation and a myeloid skewing (pVal<0.01) in the early phases of colitis, which was confirmed in a model of RANKL-induced osteoclastogenesis. Inhibition of TNF-a reduced the myeloid skewing by OCL both in vitro and in vivo in colitis (p-val=0.001). Lastly, we observed a positive correlation between osteoclast activity and the proportion of myeloid cells in the blood in Crohn's disease patients ($R^2$=0.79).

**Conclusion:** Our results shed light on a new role of osteoclasts in colitis in vivo, demonstrating they exert an inflammatory activity through an early action on hematopoiesis, leading to an increase in myelopoiesis sustaining gut inflammation.

**P091**

**Even in the absence of RANKL and serum, osteoclasts from female donors aged 60-80 years are more active than osteoclasts from females aged 25-35 years**

*Neha Sharma[1], Jacob B. Olesen[2], Pernille Hermann[3], Line S Andersen[4], Julie Therese Skaugen[4], Torben Barington[4], Megan M Weivoda[5], Kent Søe[6]*
*[1]University of Southern Denmark, Department of Clinical Research, Odense, Denmark ; [2]Odense University Hospital, Department of Pathology, Odense, Denmark ; [3]Odense University Hospital, Department of Endocrinology, Odense, Denmark ; [4]Odense University Hospital, Department of Immunology, Odense, Denmark ; [5]Mayo Clinic, Department of Endocrinology- Diabetes- Metabolism and Nutrition, Rochester, United States ; [6]University of Southern Denmark, Department of Clinical Research, Odense M, Denmark*

**Abstract Text**

**Background:** Previously, we reported that the bone resorptive activity of human osteoclasts increases with age of the donor, especially due to more osteoclasts making trenches.

**Purpose:** In this study, we aim to explore if the donor's age continues to exert a significant influence on in vitro bone-resorbing osteoclasts, even in the absence of fetal bovine serum (FBS) and RANKL.

**Methods:** 15 female donors were recruited, Group 1 (25-35 years, n=6) and Group 2 (60-80 years, n=9). $CD14^+$ monocytes were differentiated into osteoclasts over 9 days. Osteoclasts were detached, split in two, and reseeded on bone slices and cultured with 1) M-CSF, RANKL, and FBS or 2) only M-CSF for 72h. Bone resorption was either visualized by toluidine blue and analyzed blinded for pits and trenches or analyzed by 72h time-lapse.

**Results:** In the presence of FBS and RANKL: trench surface(TS)/BS, G1 0.8% vs G2 2.8%, p=0.004; pit surface(PS)/BS, G1 1.2% vs G2 2.8%, p=ns; TS/ES, G1 40.77% vs G2 59.16%, p=0.0007. TRAcP activity, 9.7 times higher in G2 vs G1 (p=0.002).

In the absence of FBS and RANKL: TS/BS, G1 0.3% vs G2 0.8%, p=0.0004; PS/BS, G1 0.4% vs G2 1.2%, p=0.007; TS/ES, G1 41% vs G2 58%, p=0.17). TRAcP activity, 4.9 times higher in G2 vs G1 (p=0.01).

Time-lapse with FBS and RANKL; G2 (n=3) showed 2.7 times faster resorption speed with respect to cavity length (p=0.02) and 7.3 times faster resorption speed times concerning cavity area (p=0.02) compared to G1 (n=3).

Time-lapse without FBS and RANKL: G2 (n=3) showed 4 times faster resorption speed with respect to cavity length (p=0.05) and 12.5 times faster resorption speed times concerning cavity area (p=0.05) compared to G1 (n=3).

**Conclusion:** In the absence of FBS/RANKL, the donor's age still exerts a significant influence on bone-resorbing osteoclasts, rendering them more aggressive when generated from older compared to younger females.

**P092**

**Protein Kinase A activity regulates osteoclastogenesis**

*Carole Le Henaff*[1], *Yongli Qin*[2], *Lawrence Kirschner*[3], *Baohong Zhao*[2], *Nicola Partridge*[1]
[1]*New York University, Molecular Pathobiology, New York, United States ;* [2]*Hospital for Special Surgery, Weill Cornell Medical College, New York, United States ;* [3]*The Ohio State University Wexner Medical Center, Departments of Cancer Biology and Genetics and the Division of Endocrinology, Columbus, United States*

**Abstract Text**

Protein Kinase A action in osteoclasts has not been clearly investigated. Previous studies were mainly done *in vitro* without clear results yielding controversies in PKA activity in osteoclastogenesis or osteoclast activity. A few studies, such as with calcitonin, associated PKA activation to an inhibition of osteoclastogenesis or osteoclast activity whereas other studies, such as with dopamine showed the opposite. To elucidate the role of PKA activity during osteoclastogenesis, we constitutively modulated this enzyme activity in osteoclastic lineages (from monocytes to mature osteoclasts) using *LysM*-Cre in mice: first, we deleted the PKA regulatory subunit 1A (*Prkar1a*) to increase PKA activity ($PKA^{highOC}$) and secondly, we blocked PKA activation using a dominant negative mouse model ($PKA^{lowOC}$) which results in a mutation in the cAMP binding site of the regulatory subunit and blocks the release of the active catalytic subunit. At 2 months of age, in our mouse models, hyperactive osteoclastic PKA caused a decrease in trabecular bone volume (-23% in males to -33 in females), thickness (-12%) and number (-15% to -24%) and an increase in trabecular separation (14%), suggesting an increased osteoclastogenesis. This was confirmed, *in vitro*, with primary osteoclastic cell cultures which showed a doubling in osteoclast numbers. The cortical thickness was decreased in hyperactive mice in the metaphysis (-10% to -14%) but no changes were observed in the diaphysis. Conversely, hypoactive osteoclastic PKA increased trabecular bone volume (18%) with increased trabecular thickness (9% to 12%) and separation (23% to 45%). Interestingly, the cortical thickness was only reduced in the diaphysis with hypoactive PKA (-7%). The cortical bone phenotype was more pronounced in females compared with males. These results collectively suggest that PKA activity positively regulates osteoclastogenesis. These genetic data, for the first time, corroborate that PKA is a key positive regulator in osteoclastogenesis.

**Poster Sessions: Osteocytes and mechanobiology**

**P093**

**Bone-derived extracellular vesicles (EVs) cargos: new players in osteoblast differentiation and function**

*Laura Entz*[1], *Floriane Binet*[1], *Meggane Amiot*[1], *Solène Tessier*[1], *Joëlle Véziers*[2], *Laurence De Beaurepaire*[3], *Gwennan André-Grégoire*[4], *Florent Dingli*[5], *Damarys Loew*[5], *Pierre Weiss*[6], *Angélique Galvani*[1], *Valérie Geoffroy*[1]
[1]*Nantes Université- Oniris- Univ Angers- Inserm, Regenerative Medicine and Skeleton- RMeS- UMR 1229, Nantes, France ;* [2]*Nantes Université- Oniris- CHU Nantes- Inserm, Regenerative Medicine and Skeleton- RMeS- UMR 1229, Nantes, France ;* [3]*Oniris- INRAE, Immuno-Endocrinologie Cellulaire et Moléculaire IECM- USC1383, Nantes, France ;* [4]*Nantes Université- Inserm- CNRS- Univ Angers, Centre de Recherche en Cancérologie et Immunologie Intégrée Nantes Angers CRCI2NA, Nantes, France*

; [5]Institut Curie- PSL Research University, CurieCoreTech Mass Spectrometry Proteomics, Paris, France ; [6]Nantes Université- Oniris- Univ Angers- CHU Nantes- Inserm, Regenerative Medicine and Skeleton- RMeS- UMR 1229, Nantes, France

**Abstract Text**

Osteocytes are known to regulate bone remodeling by secreting paracrine factors that target osteoblasts, osteoclasts and their precursors, but they can also communicate via extracellular vesicles (EVs). Only a few studies have described EVs in osteocytes, and mainly *in vitro*.

The main objective of this work was to collect, characterize and evaluate *in vitro* the potential osteogenic properties of bone-derived EVs.

Two subpopulations of EVs, large EVs (lEVs) and small EVs (sEVs), were isolated from the long bones of adult Swiss male mice by differential centrifugation and characterized physicochemically and functionally. This study was performed according to the French and European regulations on care and protection of laboratory animals. Protein and miRNA cargos associated with lEVs and sEVs were identified by proteomics and small RNA-Seq, respectively.

We demonstrated that osteoblast and osteocyte markers were among the cargos identified in bone-derived lEVs and sEVs (Phex, Sclerostin, RANK-L). We have also shown that lEVs can be taken up by stromal cell and increase their commitment to the osteoblastic lineage, for example by increasing Alpl activity ($p<0.0001$) and osx gene expression, and that sEVs can stimulate their activity, for example by increasing ocn gene expression and mineralized nodule formation. Cargos present in lEVs such as unique proteins (Fhl2, Ptn) and several miRNAs (miR-22-3p, x0.4, $p=7.25x10^{-05}$ ; miR-378b, x2.2, $p=8.18x10^{-06}$ ; miR-128-3p, x2.3, $p=7.25x10^{-05}$) were associated with the regulation of early osteoblastic pathways (Hippo, TGF-β, Wnt). In addition, regulators of extracellular matrix mineralization such as Alpl (x4.07, $p=4.7x10^{-14}$) and miR-125b (x1.26, $p=5.09x10^{-15}$) were enriched in sEVs.

This work demonstrates for the first time that osteocytes are capable of secreting EVs *in vivo*: osteocyte EVs can be extracted from bone tissue, and some of their cargos could in part recapitulate the osteocytes' ability to regulate bone formation.

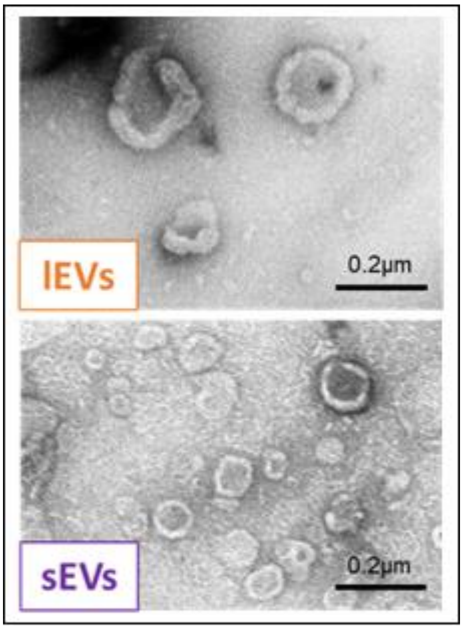

**P094**

### An automated image pre-processing pipeline to identify lacunar-canalicular networks from FITC-stained confocal microscopy images

*Francisco Correia Marques[1], Dilara Yilmaz[1], Jerome Schlatter[1], Christian Gehre[1], Xiao-Hua Qin[1], Esther Wehrle[1,2], Gisela Kuhn[1], Ralph Müller[1]*
*[1]Institute for Biomechanics, ETH Zurich, Zurich, Switzerland ; [2]AO Research Institute, AO Foundation, Davos, Switzerland*

**Abstract Text**

**Background/Introduction:** The osteocyte network is vital to maintain bone homeostasis. Previous research has associated progressive osteocyte degeneration with bone loss, as demonstrated in a premature ageing mouse model (PolgA). As lacunar-canalicular networks (LCN) represent osteocyte network architectures, LCN morphometric analysis can help our understanding of degenerative bone conditions. However, existing evaluation methods were designed for specific LCN imaging protocols, preventing their use on datasets obtained with alternative well-established staining protocols.

**Purpose:** This study introduces an automated preprocessing pipeline for FITC-stained images of cortical bone that enables a reproducible segmentation of the LCN, whose morphometry can be analysed with existing evaluation methods (e.g., cschurm/ocy_connectomics_updated).

**Methods:** Fluorescein isothiocyanate (FITC)-stained undecalcified femur sections (50 µm thick, n=20) from 20-week-old female PolgA mice and their wildtype (WT) littermates were imaged with 3D confocal microscopy (pixel size: 0.2 µm). The brightness of each section in a z-stack was adjusted (sliding-window approach with kernel: 50 µm, stride: 8 µm), followed by Gauss-filtering (sigma: 0.65 µm). After intensity normalisation, contrast-limited adaptive histogram equalisation (CLAHE) was applied (kernel: 6 µm) to enhance lacunae and canaliculi details. Sigmoid correction was performed with a sample-specific cut-off value that scaled the range of the intensity histogram to the interval (0, 1). Images were binarised (threshold: 0.6) and the LCN was analysed with ocy_connectomics_updated.

**Results:** Scale-dependent calibration of all parameters enabled reliable identification of the LCN, verified by visual inspection (Figures 1A-C). Subsequent LCN analysis overestimated lacunae numbers compared to manual counting (average 50±8 vs 41±6 lacunae/image, respectively; p=0.001, Student's t-test) but was still sensitive to differences between PolgA and WT groups in LCN morphometric parameters, such as "canalicular density" (Figure 1D).

**Conclusion(s):** Our pipeline enables automated high-throughput preprocessing of FITC-stained confocal images of LCN, expanding the range of datasets compatible with established tools for evaluating LCN morphology towards the characterisation of osteocyte degenerative conditions.

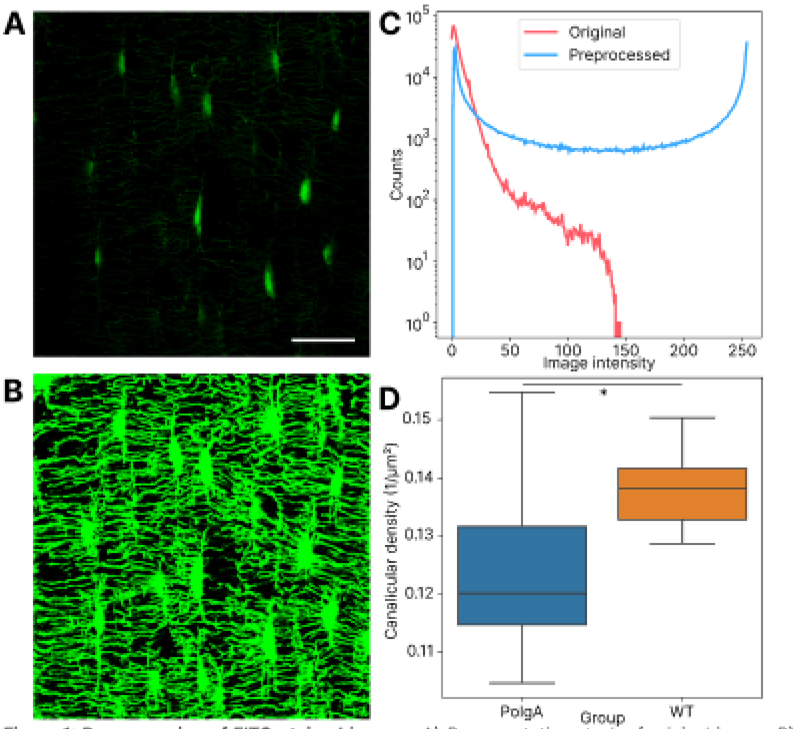

**Figure 1: Preprocessing of FITC-stained images.** A) Representative stack of original image. B) Image after CLAHE and sigmoid correction, with sharper details around the LCN. C) Comparison of the histogram of the original and preprocessed images. D) Comparison of the canalicular density between 20-week female PolgA and WT mice (p=0.02, Student's t-test).

**P095**

**Osteocytes Response to exercise-induced secretion of myokines**

*Eirini Giannousi[1], Polyzois Makras[2], Grigorios Papadopoulos[3], Elena Tsourdi[4], Antonios Chatzigeorgiou[5], Maria Yavropoulou[6]*
*[1]Medical School- National and Kapodistrian University of Athens-, Department of Physiology, Athens, Greece ; [2]251 Hellenic Air Force & VA General Hospital, Department of Medical Research-, Athens, Greece ; [3]Medical School- National and Kapodistrian University of Athens, Department of Physiology, Athens, Greece ; [4]Technische Universität Dresden, Department of Medicine III and Center for Healthy Aging, Athens, Greece ; [5]Medical School- National and Kapodistrian University of Athens- LAIKO University Hospital of Athens, Department of Physiology, Athens, Greece ; [6]Medical School- National and Kapodistrian University of Athens- LAIKO University Hospital of Athens, First Department of Propaedeutic and Internal Medicine, Athens, Greece*

**Abstract Text**

**Background:** In the state of exercise, osteocytes are able to sense variations in mechanical stress, respond and send signals to the surrounding cells. However, data are scarce regarding a direct effect of exercise -produced myokines in bone cells.

**Purpose:** In this study we investigated the potential direct effect of myokines derived by stressed-myoblasts in cell culture of osteocytes**.**

**Materials and Methods:** C2C12 myoblasts were cultured in elastic membranes until the 10th day of their differentiation. Flexcell® FX-5000™ Tension System was used to apply tension load to C2C12 myoblasts cell culture, so as to mimic the loading pattern of mature skeletal muscle and the supernatant was collected. Proteomic characterization of the culture supernatant and statistical processing was performed by PERSEUS software, Max Quant (v.16.10.14). Osteocytes-like cells MLO-Y4 were cultured in the presence of the stressed-C2C12 myoblasts supernatant followed by gene expression analysis (qPCR).

**Results:** Proteomic analysis revealed up 30 proteins related to bone metabolism, of which 15 were overexpressed in the supernatant of the stressed-C2C12 myoblasts (Figure 1a). Treatment of MLOY-4 with the stressed-C2C12 myoblasts' supernatant increased expression of genes related to mechanosensing (prostaglandins E2), osteoclastogenesis (M-CSF, RANKL, osteoprotegerin) and osteocyte's maturation (Connexin 43, podoplanin) (Figure 1b-g).

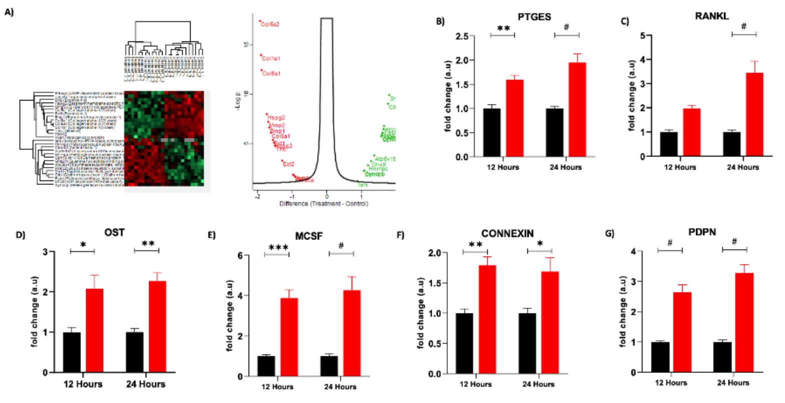

**Figure 1.** A) Proteomic characterization of stressed-C2C12 supernatant. Respective Gene Names list of Over- or Under- expressed gene products in the treatment group. B-G) Gene expression analysis of MLOY4 treated with stressed-C2C12 supernatant. ΔΔCT method (Relative to Average Control), Multiple Comparison: 2 way-ANOVA.
PTGEs, prostanglandines; RANKL, Receptor activator of nuclear factor kappa-B ligand; OST, osteoprotegerin; MCSF, Macrophage colony-stimulating factor ; PDPN, podoplanin ; a.u., arbitrary units

**Conclusion:** Exercise-induced mechanical loading may affect bone remodeling through a direct effect of secreted myokines in osteocytes.

**P096**

### Decoding Osteoarthritis: Comparative Analysis of Osteocyte Lacunar Morphological Parameters in Subchondral Bone Plate and Trabeculae

*Dzenita Muratovic[1], David Findlay[1], Gerald Atkins[1]*
*[1]The University of Adelaide, Centre for Orthopaedic and Trauma Research, Adelaide, Australia*

**Abstract Text**

**Background:** Osteoarthritis (OA) manifests intricate changes in the subchondral bone. Despite its acknowledged significance, a substantial research gap exists in understanding the specific features within the subchondral bone plate (SBC plate) and subchondral bone trabeculae (SBC trabeculae). This study aims to determine the osteoarthritis-related morphological characteristics of osteocyte lacunae in the SBC plate and SBC trabeculae and their relationship with clinical factors and cartilage degeneration.

**Methods:** Bone biopsies were obtained from 12 patients (six males and six females, aged 70±8 years) undergoing knee arthroplasty for advanced OA. The biopsies were imaged using synchrotron radiation micro-computer tomography to obtain subchondral bone structural characteristics and histologically processed to obtain Osteoarthritis Research Society International (OARSI) cartilage degenerative grades and osteocyte cellular characteristics.

**Results:** Compared to SBC trabeculae, the SBC plate is characterised by higher bone mineral density (BMD) (p=0.04) but a lower density of viable osteocytes (p=0.04) that are more apoptotic (p=0.005) and more senescent (p=0.005). In addition, the SBC plate is associated with a higher density of osteocyte lacune (p=0.0006) that are smaller (p=0.002, less spheric (p=0.0003), and flatter (p<0.0001) compared to SBC trabeculae. Pearson correlation analyses indicated that Body mass index is positively associated with the lacunar volume (r=0.86, p=0.006) and Sauter diameter (r=0.26, p=0.007) in SBC plate and negatively associated with the lacunar volume (r=-0.67, p=0.03) and lacunar sphericity (r=-0.81, p=0.004) in SBC trabeculae. OARSI grade is positively associated with BMD (r=0.50, p=0.04) in SBC plate and lacunar density (r=0.47, p=0.03) in SBC trabeculae.

**Conclusions:** Our study has revealed significant heterogeneity within subchondral bone compartments and underscores that the hyper-mineralised bone matrix of the subchondral plate is associated with abnormal bone cell metabolism and might directly impact the mechanical competence of both compartments. These insights advance our understanding of OA-related subchondral alterations, paving the way for targeted interventions to suppress disease progression.

**Poster Sessions: Chondrocytes and cartilage**

**P097**

**RNA sequencing uncovers key players of cartilage calcification in knee osteoarthritis**

*<u>Sonia Nasi</u>[1], Ilaria Bernabei[1], Elodie Faure[2], Julien Wegrzyn[2], Nathalie Busso[2]*
*[1]CHUV, Musculoskeletal Medicine, Epalinges, Switzerland ; [2]CHUV, Musculoskeletal Medicine, Lausanne, Switzerland*

**Abstract Text**

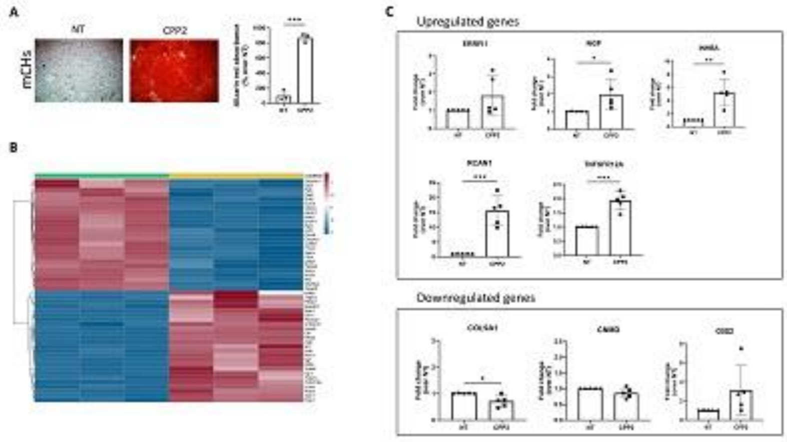

**Background:** Osteoarthritis (OA) is a joint disease characterized by pathologic calcification, a process involving calcium-containing crystals deposition by chondrocytes. The precise mechanisms driving calcification remain elusive, posing a significant challenge for therapeutic interventions.

**Purpose:** To identify pivotal players in knee OA cartilage calcification, offering insights for targeted interventions.

**Methods:** Primary murine chondrocytes were stimulated with secondary calciprotein particles (CPP2) or left untreated (NT) in DMEM high glucose for 6 hours. Lysates were prepared according to MERCURIUS™ Extraction-free DRUG-seq kit and submitted to Alithea Genomics SA (Lausanne, Switzerland) for Bulk RNA sequencing on an Illumina Novaseq 6000. STARsolo v2.7.9a aligned and quantified reads against the mouse (GRCm38.102) genome. DESeq2 identified significantly modulated genes (abs(LogFC)>1, FDR < 0.05), and the top 50 differentially expressed (DE) genes were cross-referenced with human OA cartilage datasets from previous studies (*i.e.* OA *vs* healthy, or damaged *vs* undamaged). Finally, we stimulated human primary chondrocytes obtained from 5 osteoarthritis patients with CPP2 to validate the modulation of some of these genes by qRT-PCR.

**Results:** We first confirmed that CPP2 induce calcification in primary murine chondrocytes (Figure panel A). By Bulk RNA sequencing we identified 1466 genes (668 up- and 798 downregulated) differentially expressed between CPP and NT murine chondrocytes. Out of the top 50 DE genes (Figure panel B), 18 were significantly upregulated and 4 downregulated in at least 1/7 OA cartilage datasets. Notably, previously studied genes in osteoarthritis (ERRFI1, NGF, INHBA, RCAN1, and GREM1) were found and new candidates (S100A10, EIF5A, and TNFRSF12A) emerged. In primary human chondrocytes, we confirmed upregulation of NGF, INHBA, RCAN1 and TNFSFR12A by CPP2, and downregulation of COL9A1 (Figure panel C).

**Conclusion:** Our study unveils NGF, INHBA, RCAN1 and TNFSFR12A as key players in cartilage calcification, presenting promising avenues for therapeutic interventions in a domain where effective treatments are currently limited.

**P098**

**Mechanisms of NADPH oxidase 4 induced osteoarthritis**

Elodie Zocola[1], Felix Renaudin[1], Cynthia Diabanza[1], Thomas Laumonier[1], Didier Hannouche[1]
[1]University Hospital and Faculty of Medicine, Cell Therapy & Musculoskeletal Disorders Lab-Department of Orthopedic Surgery, Genève, Switzerland

**Abstract Text**

**Introduction:** In Osteoarthritis (OA), cartilage undergoes progressive degeneration, where low-grade inflammation plays a pivotal role through reactive oxygen species (ROS). Our previous research showed that deletion of NADPH oxidase 4 (NOX4), a major ROS producer in chondrocytes, reduces cartilage degradation.

**Purpose:** This study evaluated the mechanisms of NOX4-induced OA.

**Methods:** After ethical approval, in-vivo OA was induced via destabilization of the medial meniscus (DMM) in WT and NOX4$^{-/-}$ mice. After eight weeks, mice were sacrificed for immuno-histo-chimistry (IHC) analysis. Femoral heads from 9-week-old mice (WT and NOX4$^{-/-}$) were cultured for 72 hours with PBS or IL-1β. Primary chondrocytes extracted from 4/6-day-old pups femoral heads and knees (WT and NOX4$^{-/-}$) were cultured for 24 hours with PBS or IL-1β. Genes expression were quantified by RTqPCR. Proteins were quantified in supernatants of chondrocytes and femoral heads using ELISA and colorimetric dosage.

**Results:** IHC revealed stronger IL-1β and NLRP3 expression after DMM surgery in WT mice, as compared to NOX4 $^{-/-}$ mice.
In-vitro, IL-1β stimulation increased IL-18 secretion in WT but not in NOX4$^{-/-}$ native chondrocytes. In

WT cells, anabolic (AGG, COL2, SOX9) and NOX4 genes expression was decreased and catabolic (MMP3, MMP13, ADAMTS4, ADAMTS5) and inflammation (IL1, IL6, TNF, COX2) genes expression was increased post IL-1β stimulation. We observed lower ADAMTS4, ADAMTS5 and IL1-β and higher COX2 and IL6 genes expression in NOX4$^{-/-}$ chondrocytes as compared to WT.
IL-1β treatment increased MMP3, IL6 and proteoglycan secretion in chondrocytes and femoral heads. Surprisingly we didn't observe any difference between WT and NOX4$^{-/-}$ for these parameters.

**Conclusion:** Our results demonstrate that NOX4 deletion inhibits NLRP3 inflammasome activation and IL-18 secretion. We showed that NOX4 deficiency inhibits ADAMTS gene expression. These results underline that NOX4 could aggravate OA by activation of ADAMTS enzymes via the NLRP3/IL-18 pathway.

**P099**

**Lin28a induces chondrocyte energy metabolism reprogramming to regenerate cartilage**

*Eric Hay[1,2], Zohra Bouchemla[1], Inès Slitine Inès Slitine[1], Valentin Duong[1], Lucie Orliaguet[3], Gwenaelle Jayat[2], Fawaz Alzaid[3], Augustin Latourte[1,4], Pascal Richette[4,5], Hang-Korng Ea[2,4,5], Martine Cohen-solal[2,6]*
*[1]Bioscar INSERM 1132, cartilage team, Paris, France ; [2]université Paris Cité, medical Faculty, Paris, France ; [3]Cordeliers Research Center, Immediab, Paris, France ; [4]Lariboisière APHP hospital, rhumatology, Paris, France ; [5]Bioscar INSERM 1132, inflammation, Paris, France ; [6]Bioscar INSERM 1132, bone fragility team, Paris, France*

**Abstract Text**

Osteoarthritis drive to irreversible articular cartilage degradation, which lacks regenerative capacity and without curative treatment. Our Preview publication have demonstrated that Lin28a over expression is associated with chondrocyte reprogramming and cartilage protection through let7a/HMGA axes.

Osteoarthritis progression is associated with chondrocyte energetic metabolism dysfunction, for that our focus here is on examining the impact of Lin28a on this process and understanding how energetic metabolism plays a role in chondrocyte reprogramming.

In vitro Data was obtained from primary murine chondrocyte (Rnaseq, RtqPCR, WB IF, seahorse), ex vivo data from human cartilage explant (immunohistology, SafraninO staining) and in vivo data from menisectomized mice (WT/Lin28TG$^{col2er}$), (immunohistology, SafraninO staining, RTqPCR)

RNAseq analysis show that OA chondrocytes (Wnt3a treatment) an enrichment of genes associated with oxidative stress and mitochondrial impairment (+53% q<0.05). This drive to a significant mitochondrial failure (Spare Respiratory Capacity 0.015fold). Conversely, Lin28a protect mitochondria increase glycolysis (SRC 1.8fold ECAR 2.7fold). SDHA (TCA enzyme), expression is increased in OA condition in vitro (2.1 fold p<0.05), ex vivo (4;1 fold) and in vivo (10.4 fold) this increases is diminished in presence of Lin28a (p<0.05). SDHA inhibitor. Aptenin5 inhibited the increased expression of mmp13 and adamts5 induced in OA condition in vitro, ex vivo. (12,4fold VS 3,1fold p<0.05). Because SDHA catalyse succinate/fumarate conversion, we tested succinate accumulation on chondrocytes. succinate counteracts the pro-catabolic and anti-anabolic effects of Wnt3a in vitro and ex vivo (9.83fold,4.12fold p<0.05). succinate accumulation, as Lin28a overexpression, stabilized HIF-1α, (respectively 3.2 and 4.3fold) which plays a role in increased glycolytic function ang glucose uptake (3.2fold p<0.05)

our data suggest that the energetic metabolism reprogrammed by Lin28a overexpression contributes to cartilage protection during osteoarthritis through the Succinate-HIF-1α axis. Indicating that exploring chondrocyte energy metabolism could offer new insights into osteoarthritis therapeutics.

**P100**

**Kashin-Beck disease : a case of "Big bone disease"**

*Anne-Cecile Debrach*[1], *Andrea Trombetti*[1]
[1]*Geneva University Hospitals and Faculty of Medicine, Division of Bone Diseases- Department of Medicine, Geneva, Switzerland*

**Abstract Text**

**Introduction:** Kashin-Beck disease (KBD) is a rare chronic osteoarthropathy which is endemic in Tibet, China, North Korea and Eastern Siberia. The epiphyseal plate and the articular cartilage are most commonly affected, with focal chondronecrosis and impaired endochondral ossification, leading to growth retardation, brachydactyly and secondary degenerative changes. The exact cause is poorly understood; however, selenium deficiency and fungal contamination of water have been suggested as environmental causes of KBD.

**Case:** We present the case of a 51-year-old patient who attended the outpatient clinic in Geneva with mechanical polyarticular joint pain and hand deformities. The patient was originally from Heilong Jiang, in the North-East of China. Two out of his three siblings had similar symptoms. Both his children, who grew up in the Shandong area, were asymptomatic.

On clinical examination, his height was 1.58 m. Deformities of the hands could be observed, with slight brachydactyly, campodactyly and widened proximal interphalangeal joints without synovitis. Range of motion was significantly decreased at the wrists and ankles. The rest of the musculoskeletal examination as well as the neurological and dermatological examinations were unremarkable.

Radiographs showed wide osteophytes of the proximal and distal interphalangeal joints as well as degenerative changes at the wrists, hips, elbows and ankles. Blood tests, including inflammatory markers, full blood count, rheumatoid factor, anti-CCP, calcium, phosphate, urate, liver function tests, creatinine and transferrin saturation coefficient were unremarkable.

The clinical and radiological investigations correlated with a diagnosis of KBD. The patient was given chondroitin and non-steroidal anti-inflammatory drugs. Whilst selenium replacement seems beneficial in children, the mainstay of treatment in adults remains conservative.

**Conclusion:** Whilst a well-known pathology in Asia, KBD is rather unknown in Europe. We aim to highlight the characteristics of this disease, to help clinicians from other parts of the world to recognize and treat it more efficiently.

**P101**

**Impaired ossification of the femoral head in a mouse model of mucolipidosis type III gamma**

Assil-Ramin Alimy[1], Thorsten Schinke[2], Sandra Pohl[2], Tim Rolvien[1]
[1]University Medical Center Hamburg-Eppendorf, Department of Trauma and Orthopaedic Surgery, Hamburg, Germany ; [2]University Medical Center Hamburg-Eppendorf, Department of Osteology and Biomechanics, Hamburg, Germany

**Abstract Text**

**Background:** Mucolipidosis type III (MLIII) gamma is a rare lysosomal storage disorder caused by mutations in *GNPTG* encoding the γ-subunit of GlcNAc-1-phosphotransferase. One of the leading clinical problems in affected patients is early-onset hip degeneration (i.e., osteoarthritis) associated with femoral head collapse. The pathophysiological basis of hip pathologies in ML III gamma is not well understood.

**Purpose:** This study aimed to elucidate the micromorphology of the hip joint in a mouse model of MLIII gamma (*Gnptg*$^{ko}$).

**Methods:** We analyzed hip micromorphology of 4-week and 25-week-old *Gnptg*$^{ko}$ mice, compared with age-matched wildtype (WT) controls (n≥4 per age and genotype). Micro-CT was used for microstructural analysis, while joint phenotyping was performed by undecalcified histology and histomorphometry.

**Results:** There were no differences in acetabular or femoral three-dimensional configuration or skeletal microstructure between *Gnptg*$^{ko}$ mice and WT. While the growth plate thickness in 4-week-old *Gnptg*$^{ko}$ mice (155.6 µm vs 167.1 µm, p=0.75) was similar compared to WT, 25-week-old *Gnptg*$^{ko}$ mice showed thicker growth plates (110.9 µm vs 47.0 µm, p=0.047). Importantly, ossification of the femoral head was markedly impaired in *Gnptg*$^{ko}$ mice, indicated by remaining epiphyseal cartilage with increased thickness in 25-week-old *Gnptg*$^{ko}$ mice compared to WT (256.9 µm vs 0 µm, p=0.006) (Fig. 1A, B). Articular cartilage integrity, assessed by OARSI scores, was not different between *Gnptg*$^{ko}$ and WT across both age groups (Fig. 1C). However, articular cartilage thickness was reduced in 25-week-old *Gnptg*$^{ko}$ mice (26.47 µm vs 51.81 µm, p=0.013) (Fig. 1D).

**Conclusion:** *Gnptg*$^{ko}$ mice displayed a specific osteochondral phenotype in articular and epiphyseal cartilage of the femoral head, primarily defined by impaired ossification. These results provide an explanatory basis for hip pathologies in MLIII gamma. Further investigations are underway to determine whether dysfunctional chondrocyte homeostasis or other mechanisms are decisive for the observed pathology.

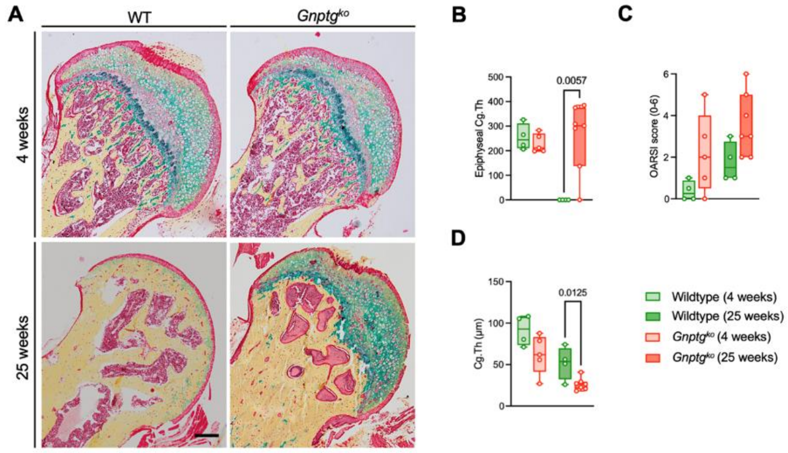

**P102**

**Effect of caspase-1 inhibitors VX-740 and VX-765 on murine chondrogenic cultures**

*Barbora Vesela[1], Kateřina Holomková[1], Corina Blecha[1], Natalia Obratilova[1], Jian Mei[2], Anja Pasoldt[2], Susanne Grässel[2], Eva Matalova[1]*
*[1]Academy of Sciences, Institute of Animal Physiology and Genetics, Brno, Czech Republic ; [2]University of Regensburg, Dept. of Orthopedic Surgery- Experimental Orthopedics, Regensburg, Germany*

**Abstract Text**

In osteoarthritis, TNFα and IL-1β are the key cytokines driving catabolic pathways in affected joints. Caspase-1 is the central pro-inflammatory caspase working as IL-1β converting enzyme. Several caspase inhibitors have been tested for therapies, in the case of caspase-1 inhibition this applies for VX-740 (Pralnacasan) and VX-765 (Belnacasan). Recently, caspases are considered to have several important roles beyond promoting apoptosis and inflammation.

To investigate such functions of caspase-1 and impact of its inhibitors VX-740 and VX-765, murine chondrogenic 2D/3D? cultures were challenged. The inflammatory-like conditions were achieved by addition of IL-1β for?h. We investigated four parallel cell culture regimen: 1) control, 2) IL-1β treatedcells, 3) IL-1β + VX-740 treated cells, 4) IL-1β + VX-765 treated cells. Samples were collected after 7 days of cultivation. Morphologically, there was an apparent difference in number of nodules formed in individual groups. While IL-1β caused a dramatic decrease, there was a rescue effect in the case of both inhibitors. Additionally, the samples were analysed by PCR Arrays focused on autophagic and chondro/osteogenic factors. The results pointed to several candidate genes being affected by VX caspase-1 inhibitors. The affected genes among the chondro/osteogenic factors include Nog, Sp7, Cd36, within the autophagic array, the most significant change was registered for Snca. There are on-going further analyses.

Caspase inhibitors were shown to reduce the severity of cartilage lesions in experimental models of osteoarthritis. Current challenges toward their therapeutic applications include understanding of various caspase functions. This work points to engagement of caspase-1 in chondrogenesis and impact of its pre-clinically tested inhibitors on autophagy and differentiation under inflammatory-like conditions.

*Supported by the Ministry of Education, Youth and Sports, Czech Republic, Inter-Excellence/Inter-Action programme, project LUABA22019 and the Bayhost programme, Bavaria, Germany, project BTHA-JC-2022-36.*

**Poster Sessions: Endocrine factors**

**P103**

**Evidence of a Link Between Grip Strength and Type 2 Diabetes in Menopausal Women in Korea: the Korea National Health and Nutrition Examination Survey (2014-2019)**

*Yunsoo Chung[1], Jin Kyung Back[1], Jae Kyung Lee[1], Euna Choi[1], Seok Kyo Seo[1]*
*[1]Yonsei University College of Medicine, Department of Obstetrics and Gynecology, Seoul, Korea- South*

**Abstract Text**

**Introduction**: Multiple studies are on-going about aging and sarcopenia. As aging, the risk of sarcopenia increases and sarcopenia could be diagnosed based on muscle strength, muscle mass and physical performance. Since hand grip strength (HGS) is relatively easy to measure, it is used to evaluate sarcopenia in multiple studies. Diabetes is highly related to cardiovascular disease, chronic renal disease, and metabolic syndromes. To prevent developing diabetes or diagnosis in early stage, effort to find proper diagnostic biomarkers is on-going. Some studies demonstrate that people with strong HGS are less likely to be diagnosed diabetes, since they have more skeletal muscle mass and efficiently control the blood glucose level.

**Purpose**: The relation between HGS and diabetes is not well studied in Korean postmenopausal population. Through this study our team want to investigate the relation between them.

**Methods**: Korea National Health and Nutrition Examination Survey from 2014 to 2019 were used to analyze data of Korean menopausal women aged from 45 to 65 years old. HGS were stratified into two groups and two groups were compared based on diabetic history, HbA1c, and fasting glucose levels.

**Results**: The odds ratio (OR) for diagnosed diabetes is lower in HGS of 18kg or more compared to those in HGS of less than 18kg (OR = 0.7, p-value = 0.004). Among those who have not been diagnosed diabetes, the OR of those who either have fasting glucose level greater than 125 or HbA1C level greater than 6.4 are lower in HGS of 18kg or more compared to those in HGS of less than 18kg (OR = 0.95, p-value=0.8022; OR = 0.83, p-value=0.2709).

**Conclusions**: Among postmenopausal women, low HGS showed high risk of diabetes. However, among those who are not diagnosed diabetes, HGS cannot be a biomarker of assessing the risk of diabetes.

**P104**

**Irisin, osteoporosis and endothelial function**

*Marie-Eva Pickering[1], Julie Durif[2], Nicolas Macian[3], Veronique Morel[4], Charlotte Oris[2]*
*[1]CHU Gabriel Montpied, Rheumatology Department, Clermont-Ferrand, France ; [2]University Hospital Clermont-Fd, Biochemistry department, Clermont-ferrand, France ; [3]University Hospital Clermont-Fd, Clinical Investigation Platform/CIC, Clermont-Fd, France ; [4]University Hospital, Clinical Investigation platform PIC/CIC, Clermont-Fd, France*

**Abstract Text**

**Introduction**. Osteoporosis and cardiovascular disease are common comorbidities in aging: arterial calcification and bone mass/mineralization share a number of features. Among potential biomarkers and targets for prediction and treatment, irisin, a hormone, may be a crucial regulator. Lower circulating irisin levels and irisin dysfunction have been suggested to play an essential role in cardiovascular disease and endothelial damage as well as in bone pathologies like osteoporosis.

**Purpose**. The main aim of the study is to evaluate if circulating irisin levels correlate with endothelial function and with bone mass/density in osteoporotic post-menopausal women.

**Methods**. This cross-sectional study included the first 70 women with post-menopausal osteoporosis who took part to the VASCO cohort (NCT05228262) in Clermont-Fd University Hospital, France (150

inclusions in March 2024). Demographic criteria were age, body mass index (BMI), bone mineral density (BMD, g/cm2) and T-score. Serum bone markers were measured including irisin (Elisa test), Carboxy terminal collagen crosslinks (CTx) and serum Propeptide N-terminal procollagen type 1 (P1NP). Endothelial function was assessed non-invasively using the flow-mediated dilation (FMD) technique (in %). FMD is a tool for examining the pathophysiology of cardiovascular disease and a rigorous standardized protocol was followed to validate its reproducibility. Data are expressed in means ± SD.

**Results**: Women were 69,4 ± 7,8 years old, BMI : 24,6 ± 9,2; T score ; -2,6 ± 1, BMD:0.587±0.078 g/cm2; Serum irisin levels were 64.12 ± 16.42 ng/mL, P1NP : 46.79 ± 38.28 µg/L; CTx:0.458±0.382 µg/L. FMD : 2.85 ± 2.53%. No correlation was shown between irisin (and the other markers) and FMD.

**Conclusions**: Preliminary findings underline a dysfunctional endothelial function with pathological cardiovascular risk, impaired bone markers and low irisin levels in women with postmenopausal osteoporosis. Correlations between parameters was however not shown at this study mid-point and needs to be confirmed on the whole population.

**P105**

**PTH-induced osteoblast differentiation of marrow stromal cells involves the Src-dependent tyrosine phosphorylation of YAP at Y428**

*<u>Sara Monaci</u>[1], Yongxing Zhang[1], Mengrui Wu[1], Maisa Monseff[2], Hiroyuki Okada[1], Clifford Rosen[3], Francesca Gori[1], Roland Baron[4]*
*[1]Harvard School of Dental Medicine, Oral Medicine- Infection and Immunity, Boston, United States ; [2]Harvard Medical School, Medicine, Boston, United States ; [3]Maine Medical Center, Research Institute, Portland, United States ; [4]Harvard Medical School and Harvard School of Dental Medicine, Medicine and Oral Medicine- Infection and Immunity, Boston, United States*

**Abstract Text**

**Background:** PTH targets osteoblasts and osteocytes to increase bone formation and mesenchymal stem cells to favor their differentiation into osteoblasts. PTH acts through the PTH1R, a GPCR. PTH signaling also cross-talks with WNT and/or TGFb signaling, with synergistic or antagonistic effects, respectively. Whether PTH interacts with Hippo signaling, for which no receptor(s) or ligand(s) have been identified, has not been explored.

The Hippo pathway comprises two kinases, MST1 and LATS, which regulate the transcriptional coactivators YAP and TAZ. In the active state, LATS1/2 phosphorylates YAP/TAZ at serine residues, promoting degradation.

**Purpose**: Investigate whether PTH/PTH1R regulates Hippo signaling, and whether this regulation contributes to PTH effects on bone cells.

**Methods**: Using a murine marrow stromal cell line (W20), we tested whether PTH alters LATS regulation of YAP, exploring both serine and tyrosine phosphorylation by western blot and immunoprecipitation, and determined the effects of YAP1 phosphorylation on W20 differentiation into osteoblasts.

**Results**: As expected, PTH led to W20 osteoblast differentiation. Importantly PTH treatment stabilized YAP by decreasing Serine 127 phosphorylation, increasing YAP target gene expression and showing for the first time that PTH affects the Hippo pathway. PTH also induced an increase in YAP tyrosine phosphorylation and we identified Y375 and Y428 as two Src targets. Mutation of Y428, but not Y375, into non-phosphorylatable residues led to YAP instability. Whereas PTH stabilized YAP in WT cells, PTH led to YAP degradation, decreasing YAP target genes, ALP activity, and osteoblast differentiation in $YAP^{Y428F}$mutant cells. Importantly, PTH activated Src Kinase and Src-dependent phosphorylation of YAP Y428 with YAP nuclear translocation, increased YAP target genes and osteoblast differentiation.

**Conclusions**: Through Src-mediated phosphorylation of YAP Y428, PTH protects YAP from degradation, increasing its transcriptional activity, which contributes to PTH-mediated osteoblast differentiation. Thus, the Hippo pathway plays a role in PTH-induced differentiation of mesenchymal precursors into osteoblasts.

**P106**

**Characteristics of primary hyperparathyroidism in young adults: a retrospective study**

*Hanne Goeminne[1], Sarah Barbullushi[1], Lapauw Bruno[2], Charlotte Verroken[2]*
*[1]Ghent University, Department of Endocrinology / Unit for Osteoporosis and Metabolic Bone Diseases, Gent, Belgium ; [2]Ghent University / Ghent University Hospital, Department of Endocrinology / Unit for Osteoporosis and Metabolic Bone Diseases, Gent, Belgium*

**Abstract Text**

**Background**: Primary hyperparathyroidism (PHPT) has been extensively described in elderly populations, but its characteristics in young individuals are understudied.

**Purpose**: We aimed to investigate differences in PHPT characteristics in patients aged ≤40 vs. >40 years at diagnosis.

**Methods**: A retrospective case-control study was performed in a single tertiary center. Relevant laboratory results and information on symptoms, medical history, family history, and treatment were retrieved from electronic patient files. Ethical approval was obtained from the local Ethics Committee.

**Results**: 34 patients aged ≤40 years (range 16-40, 59% women) were gender-matched with 68 patients aged >40 years (range 41-84). Median serum calcium concentration at diagnosis was higher (2.96 vs. 2.81 mmol/L; p=0.008) and serum phosphorus concentration was lower (0.73 vs. 0.86 mmol/L; p=0.031) in young vs. older patients. Young patients more often presented with headache (12/34 vs. 10/68; p=0.017) and mood disorders including depression (15/34 vs. 17/68; p=0.05). Furthermore, median bone mineral density Z-scores at the lumbar spine (-1.7 vs. -0.6; p=0.002) and femoral neck (-1.2 vs. -0.6; p=0.016) were lower in young vs. older patients. In turn, hypertension (7/34 vs. 33/68; p=0.006) and renal insufficiency (2/34 vs. 13/68; p=0.075) were more prevalent in the older group. No differences were observed in other laboratory parameters (PTH, calciuria, 25(OH)vitamin D), presence of risk factors (prior radiotherapy in 2/34 young and 2/68 older patients, lithium use in 1 older patient), relevant family history, gastro-intestinal symptoms, prevalence of nephrolithiasis/nephrocalcinosis, total hip and forearm Z-scores, frequency of surgical treatment, or cure and recurrence rate. A hereditary form of PHPT was present in 3/34 young and 4/68 older patients, all diagnosed with MEN-1 syndrome (p>0.05).

**Conclusions**: Higher calcium and lower phosphate concentrations as well as more pronounced neurological symptoms and bone deficit in patients aged ≤40 years at PHPT diagnosis may indicate a more active disease.

**P108**

**Aged bone marrow mesenchymal stem cells-derived extracellular vesicles as a messenger for calcification paradox**

*Yali Ling*[1], *Feng Xu*[2]
[1]*The Second Xiangya Hospital of Central South University, Department of Metabolism and Endocrinology, Changsha- Hunan, China ;* [2]*The Second Xiangya Hospital- Central South University, Department of Metabolism and Endocrinology, changsha, China*

**Abstract Text**

Osteoporosis is a bone disease associated with aging. The main features of senile osteoporosis are decreased bone formation and increased fat accumulation in the bone marrow with decreased osteogenic differentiation and increased adipogenic differentiation of bone marrow mesenchymal stem cell cells (BMSCs). Vascular calcification often occurs with osteoporosis, a contradictory association called "calcification paradox". In this study, we have shown that Aged BMSCs-derived extracellular vesicles (AB-EVs) aggravate vascular calcification, and the underlying mechanism involved. Here we found that AB-EVs could be uptaken by vascular smooth muscle cells (VSMCs) and aggravated the osteogenic differentiation of VSMCs. Using vitamin $D_3$-induced vascular calcification model mice, tail intravenous injection of AB-EVs exacerbates aortic vascular calcification in mice. We also found exosomal miR-125b-5p and miR-133a-3p mediated the paracrine effect of extracellular vesicles secreted by BMSCs. MiR-125b-5p and miR-133a-3p are enriched in AB-EVs and essential for the AB-EVs-induced exacerbation of vascular calcification. In summary, our present study uncovered that the role of AB-EVs as a messenger for calcification paradox in a paracrine manner by transferring miR-125b-5p and miR-133a-3p.

**ND-P015**

**The role of C-type natriuretic peptide on bone metabolism and fracture healing in mice**

*Eri Kondo*[1]
[1]*Kondo Orthopaedic Clinic, Endocrinology, Ikoma, Japan*

**Abstract Text**

**Background/Purpose:** Recent studies have revealed that C-type natriuretic peptide (CNP) is a potent stimulator of endochondral bone growth. CNP analog therapy is effective for children with achondroplasia. Nevertheless, the effect of CNP on bone turnover has not yet been well studied.

**Methods:** In order to elucidate this issue, I investigated the bone phenotype of a mouse model with elevated plasma CNP concentrations (*CNP-Tg* mice). Next, I performed an open and stabilized femoral fracture using 8-week-old *CNP-Tg* mice and compared the healing process with age-matched wild type mice.

**Results:** Micro-CT analysis revealed less bone in femurs, but not in lumber vertebrae, of young adult *CNP-Tg* mice than that of wild-type mice. Bone histomorphometry of the tibiae from 8-week-old *CNP-Tg* mice showed enhanced osteoblastic and osteoclastic activities, in accordance with elevated serum levels of osteocalcin and TRAP5b, respectively. During the fracture healing process of mice, CNP and its receptors, natriuretic peptide receptor-B (NPR-B) and natriuretic peptide clearance receptor are immunohistochemically expressed in hard calluses of wild-type mice, suggesting possible role of CNP/NPR-B signaling in fracture repair, especially in bone remodeling stage. On micro-CT analysis, rapid decrease in callus volume was observed in *CNP-Tg* mice, followed by generation of significantly higher new bone volume with a tendency of increased bone strength. In addition, micro- CT analysis also showed that bone remodeling was accelerated in *CNP-Tg* mice, which was also evident from increased serum osteocalcin and TRAP5b levels in *CNP-Tg* mice at remodeling stage of fracture repair.

**Conclusion:** These results indicate that CNP activates bone turnover and remodeling *in vivo* and possibly accelerates fracture healing in mice.

### Poster Sessions: Energy metabolism, fat, diabetes, nutrition

### P109

### Low bone mineral density and nutritional status in women with rheumatoid arthritis

*Olga Dobrovolskaya[1], Alexey Feklistov[1], Nikolay Demin[1], Natalia Toroptsova[1]*
*[1]V.A. Nasonova Research Institute of Rheumatology, Department of Osteoporosis, Moscow, Russia C.I.S.*

**Abstract Text**

Low bone mineral density and low-energy fractures are the common complications of rheumatoid arthritis (RA).

**Aim:** to assess the relationship between low bone mineral density and nutritional status in women with RA.

**Material and methods:** 104 women aged 40-75 years with RA (ACR/EULAR criteria 2010) were examined. Anthropometry, dual-energy X-ray absorptiometry of the lumbar spine, femur neck, total hip and total body were done. Mini Nutritional Assessment-Short Form (MNA-SF) and questionnaire on frequency of dairy and protein-containing products and calcium intake were used to assess nutritional status. The study was approved by the local ethics committee.

**Results:** Osteoporosis (OP) was found in 40 (38.5%) and osteopenia - in 54 (51.9%) patients. Malnutrition and risk of malnutrition was detected in 4 (3.8%) and 47 (45.2%) women, respectively. Patients with OP were older, had BMI and MNA-SF score less than persons without OP ($p<0.05$). There were no differences between the groups depending on the presence of OP in the frequency of intake of dairy and protein-containing products. There was also no difference in calcium intake. Patients with OP had mid-upper arm circumferences, appendicular muscle mass and appendicular muscle index significantly less than women without OP. Women with and without OP didn’t differ in the fat mass and obesity frequency. Multivariate logistic regression analysis revealed that OP was related to a low nutritional status by MNA-SF (odds ratio (OR) 1.79, $p<0.01$), low blood serum albumin level (OR 1.14, $p<0.05$), and low creatinine clearance (OR 1.05, $p<0.01$).

**Conclusion:** Low nutritional status was found out in 49% RA patients. There was no relationship between OP and the frequency of dairy and protein-containing products intake. OP was associated with low nutritional status by MNA-SF, low blood serum albumin level, and low creatinine clearance.

**P110**

**Assessment of motor activity of patients undergoing health resort treatment and medical rehabilitation**

*Valeriia Vasileva*[1], *Larisa Marchenkova*[2], *Anatoliy Fesyun*[3]
[1]*National Medical Research Center of Rehabilitation and Balneology, Department of Somatic Rehabilitation of Reproductive Health and Active Longevity, Moscow, Russia C.I.S. ;* [2]*Federal State Budgetary Institution "National Medical Research Center of Rehabilitation and Balneology" of the Ministry of Health of the Russian Federation, Department of Somatic Rehabilitation of Reproductive Health and Active Longevity, Moscow, Russi ;* [3]*Federal State Budgetary Institution "National Medical Research Center of Rehabilitation and Balneology" of the Ministry of Health of the Russian Federation, Department of Healthcare Organization and Health Resorts- Acting Director of the National Medic,*

**Abstract Text**

**Aim:** To study the nature and degree of disorders of muscle strength, motor and coordination functions in patients undergoing medical rehabilitation and sanatorium treatment.

**Material and Methods:** Single cross-sectional study included 160 patients aged 40 to 65 years with normal and overweight. The complex of the study included: functional tests and assess muscle strength and balance.

**Results:** In patients with obesity, compared with persons with normal body weight of the same age, significantly ($p < 0.05$) lower indicators of muscle strength of the right and left arms, strength of the abdominal and back muscles, lower endurance of the abdominal muscles and back muscles and longer time to complete the "Get up and walk" test. Also, in obesity, it turned out to be significantly less time to maintain balance in the “Stand on one leg” tests on the right and left legs with open eyes. A statistically significant direct relationship was found between the level of back muscle strength ($\gamma = –0.82$, $p = 0.0038$) and body weight. A significant relationship was also found between the level of endurance of the back muscles to physical activity and BMI, $\gamma = -0.79$, $p = 0.01$. At the same time, there was no relationship between age and the level of endurance of the back muscles to physical activity ($\gamma = 0.107$, $p = 0.36$).

**Conclusion:** In patients with obesity at the age of 40–65 years, compared with persons with normal body weight of the same age and gender, there is a statistically significant decrease in the muscle strength of the arms, abdomen and back, a longer time to complete the "Get up" test and walk”, as well as deterioration in the function of static balance according to the results of the “Stand on one leg” test.

**P111**

**Effectiveness of complex medical rehabilitation of patients with postthrombophlebitic syndrome of lower extremities and obesity**

*Valeriia Vasileva*[1]*, Tatiana Konchugova*[2]*, Anatoliy Fesyun*[3]*, Tatiana Apkhanova*[4]*, Olga Yurova*[5]
[1]*National Medical Research Center of Rehabilitation and Balneology, Department of Somatic Rehabilitation of Reproductive Health and Active Longevity, Moscow, Russia C.I.S. ;* [2]*Federal State Budgetary Institution "National Medical Research Center of Rehabilitation and Balneology" of the Ministry of Health of the Russian Federation, Department of Rehabilitation Medicine- Physical Therapy and Medical Rehabilitation, Moscow, Rus ;* [3]*Federal State Budgetary Institution "National Medical Research Center of Rehabilitation and Balneology" of the Ministry of Health of the Russian Federation, Department of Healthcare Organization and Health Resorts, Moscow, Russia C.I.S. ;* [4]*Federal State Budgetary Institution "National Medical Research Center of Rehabilitation and Balneology" of the Ministry of Health of the Russian Federation, Department of Physiotherapy and Reflexotherapy, Moscow, Russia C.I.S. ;* [5]*Federal State Budgetary Institution "National Medical Research Center of Rehabilitation and Balneology" of the Ministry of Health of the Russian Federation, Department of Somatic Rehabilitation of Reproductive Health and Active Longevity, Moscow, Russi*

**Abstract Text**

**Aim:** Comparative study of the influence of complex methods of medical rehabilitation, including various methods of laser blood irradiation, pulse magnetotherapy and dry carbon baths, on the dynamics of indicators of composite body composition according to bioimpedance measurements in patients with postthrombophlebitic (PTS) syndrome of the lower extremities and obesity.

**Material and Methods:** The study included 40 patients with PTS of the lower extremities and accompanying obesity, the average age of which was 58.3 [51.5; 68.0] year, randomized to two groups. The patients of the 1st group received Intravenous laser blood irradiation (ILIB), impulse magnetotherapy and dry carbon baths, as well as therapeutic gymnastics in the hall. The patients of the 2nd group received a complex that included supravascular laser irradiation of blood, pulse magnetic therapy, and dry carbon baths, as well as therapeutic gymnastics in the gym. Anthropometric measurements were used for the evaluation of anti-edema and lipolytic effects after a comprehensive course of rehabilitation, as well as bioimpedance measurement of body composition.

**Results:** In patients with PTS of the lower extremities and obesity, after carrying out a course of medical rehabilitation using preformed physical factors, including various methods of laser irradiation of the blood, a significant decrease in body weight indicators was observed ($p < 0.001$), a decrease in BMI ($p < 0.001$), total and extracellular fluid ($p < 0.001$) without significant differences between groups. A significant decrease in the index of fat mass (kg) ($p = 0.007$) was found in patients of the main group.

**Conclusions:** The method of bioimpedance measurement, used for the evaluation of the composite composition of the body, showed high sensitivity and allowed to establish the greater effectiveness of the developed complex, which includes ILIB with a wavelength of low-intensity laser radiation of 635 nm in reducing fat mass.

**P112**

**The differential effect of metformin on osteocytes, osteoblasts and osteoclasts**

Ton Schoenmaker[1], Teun Vries de[1], Jianfeng Jing[2], Antonella Kleemann[1]
[1]ACTA, Department of periodontology, Amsterdam, Netherlands ; [2]ACTA, Department of oral cell biology, Amsterdam, Netherlands

**Abstract Text**

**Purpose of the review:** Metformin is an anti-glycemic agent, which is widely prescribed to diabetes patients. Although its alleged role on bone strength has been reported for some time, this review focuses primarily on the recent mechanistical insights of metformin on osteocytes, osteoblasts and osteoclasts.

**Recent findings**: Overall, metformin contributed to steering anabolic activity in osteocytes. It caused lower expression in osteocytes of the negative regulators of bone formation sclerostin and DKK1. Likewise, the osteoclastogenesis function of osteoblasts was also skewed towards lower RANKL and higher OPG expressions. Osteoblast lineage cells generally responded to metformin by activating bone formation parameters, such as alkaline phosphatase activity, higher expression of anabolic members of the Wnt pathway, transcription factor Runx2, bone matrix protein proteins, and subsequent mineralization. Metformin affected osteoclast formation and activity in a negative way: reducing the number of multinucleated cells in association with lower expression of typical osteoclast markers and with inhibited resorption. A common denominator studied in all three cell types is its beneficial effect on activating phosphorylated AMP kinase (AMPK) which is associated with the coordination of energy metabolism.

**Summary:** Metformin differentially affects bone cells, shifting the balance to more bone formation. Although metformin is a drug prescribed for diabetic patients, the overall bone anabolic effects on osteocytes and osteoblasts and the anti-catabolic effect on osteoclast suggest that metformin could be seen as a promising drug in bone diseases.

**P113**

**In otherwise healthy young men, Trabecular Bone Score (TBS) is significantly lower in case of insulin resistance: an analysis of the SIBLOS study.**

Steven Van Offel[1], Charlotte Verroken[1], Stefan Goemaere[1], Bruno Lapauw[1]
[1]Ghent University Hospital, Endocrinology Department, Ghent, Belgium

**Abstract Text**

**Background**: Type 2 diabetes mellitus (T2DM) increases fracture risk, despite a preserved or even increased bone mineral density (BMD). The dual energy X-ray absorptiometry (DXA)-derived trabecular bone score (TBS) is considered to be a proxy of bone quality and is lower in people with T2DM as compared to controls. Whether this is also the case in otherwise healthy, insulin resistant people has not been investigated.

**Purpose**: To assess whether insulin resistance already associates with TBS in young healthy men.

**Methods**: 999 young healthy men were recruited in the region of Ghent and surrounding communities and were cross-sectionally assessed for multiple bone, muscle and metabolic parameters as part of the SIBLOS study. Of 459 participants, aged 35±5.4 years, data on both TBS, fasting glucose and insulin were available. Insulin resistance was defined by a Homeostatic Model Assessment of Insulin Resistance (HOMA-IR, which is the product of fasting glucose with fasting insulin, divided by a constant) of ≥2.17. Differences in DXA-derived parameters between the insulin resistant and insulin sensitive group were assessed using t-tests.

**Results**: Of the 459 participants, 89 (19.4%) were insulin resistant. Mean TBS ± standard deviation in this group was 1.42±0.08, which was significantly lower compared to the insulin sensitive group (1.46±0.08; p=0.002). Quantitatively, BMD was higher in the insulin resistant group in all measurement regions, but this only reached statistical significance at the femoral neck and total hip BMD (0.87±0.13 g/cm² vs 0.91±0.14 g/cm², p=0.031 and 1.07±0.13 g/cm² vs 1.11±0.16 g/cm², p=0.009 respectively).

**Conclusion**: Despite being years ahead of established T2DM, TBS is lower in insulin resistant as compared to insulin sensitive young men. However, because of the small order of magnitude, no relevant statements about bone quality on the level of the individual patient can be made based upon TBS alone.

**P114**

**Relationship between handgrip strength and metabolic syndrome in Korean postmenopausal women: the Korea National Health and Nutrition Examination Survey (2014-2019)**

*Eun Jin Lee*[1]*, Jin Kyung Back*[1]*, Hee Yon Kim*[1]*, Jae Kyung Lee*[1]*, Euna Choi*[1]*, Seok Kyo Seo*[1]
[1]*Yonsei University College of Medicine, Department of Obstetrics and Gynecology, Seoul, Korea- South*

**Abstract Text**

In women, body component repositioning occurs as a result of aging and menopause, leading to increase in fat and decrease in muscle mass and strength. It has been thought that maintaining or improving muscle strength may reduce the risk of metabolic syndrome (MetS), and handgrip strength (HGS) has been used as a tool to assess muscle strength. However, the relationship between HGS and MetS remains unclear. Therefore, this study aims to investigate the relationship among relative HGS and MetS in Korean postmenopausal women.

We analyzed data from Korean menopausal women aged 45-65 years (N=4102) who participated in the Korea National Health and Nutrition Examination Survey. Participant's HGS, BMI, MetS, and five criteria were measured. Subjects were categorized into four groups based on RHGS, and stratified by age at menopause. Analyses were divided into unadjusted model, model 1 adjusted by age, and model 2 adjusted by age at menopause, dyslipidemia, contraceptive use, alcohol use, smoke, exercise and age.

In women aged 45-65 years, higher RHGS was associated with lower OR of MetS in all models, and all five MetS criteria showed similar trends in the unadjusted model and model 2. Higher RHGS was associated with lower OR of MetS in all models in the within 10 years of menopause group, and in the after 10 years of menopause group, the trend was similar in the unadjusted model and model 2. For each MetS criterion, both groups showed a trend toward lower OR with higher RHGS in the unadjusted model and model 2, but this was not statistically significant for blood pressure ≥ 135/85 and in the after 10 years of menopause group.

HGS showed a highly significant inverse association with the risk of MetS in Korean menopausal women, and it can be a novel biomarker for assessing the risk of MetS.

**P115**

**Semaglutide increases bone resorption but not formation and causes bone loss in adult men and women with increased fracture risk**

*Morten Svarer Hansen*[1], *Eva Wölfel*[2], *Niklas Rye Jørgensen*[3], *Richard Eastell*[4], *Stinus Hansen*[5], *Morten Frost*[1]
[1]*Odense University Hospital, Department of Endocrinology, Odense, Denmark ;* [2]*University of Southern Denmark, Department of Clinical Research, Odense, Denmark ;* [3]*Rigshospitalet, Department of Biochemistry, Copenhagen, Denmark ;* [4]*University of Sheffield, Division of Clinical Medicine, Sheffield, United Kingdom ;* [5]*Hospital of South West Jutland, Department of Diabetes and Endocrinology, Esbjerg, Denmark*

**Abstract Text**

**Background:** Glucagon-like peptide 1 (GLP-1) receptor agonists (GLP-1Ras), including semaglutide, are extensively used to treat obesity and type 2 diabetes, which are associated with changes in fracture pattern and higher fracture risk. Rodent and early clinical studies indicate that GLP-1Ras may advance bone formation.

**Purpose:** We hypothesised that semaglutide treatment increases bone formation in adults with increased fracture risk.

**Methods:** This randomized, placebo-controlled, double-blinded clinical trial included adult male and female subjects (without diabetes) with a total hip or lumbar spine T-score <-1.0 and/or history of low-energy fracture. Participants were randomized to receive subcutaneous semaglutide 1.0 mg or placebo once-weekly for 52 weeks. The primary outcome was change in the bone formation marker P-Procollagen type I N-terminal propeptide (P-PINP) after 52 weeks. Additional outcomes included changes in bone resorption marker P-Collagen type I cross-linked C-terminal telopeptide (P-CTX), areal BMD by DXA, and volumetric BMD (vBMD) by HR-pQCT.

**Results:** Sixty-four subjects (86% female) with a mean age of 63 years (SD 5.5) and BMI ranging from 21 to 39 kg/m$^2$ were enrolled. Assessed as the effect of semaglutide *vs*. placebo from baseline to week 52, there was no change in P-PINP (p=0.19). By contrast, P-CTX increased by 55% (p=0.005) (Figure 1). Furthermore, lumbar spine BMD decreased by 2.1% (p=0.01), and total hip BMD decreased by 2.6% (p=0.001). Femoral neck BMD was unchanged (p=0.24). Tibial vBMD decreased by 1.7% (p=0.01), and tibial cortical thickness decreased by 1.8% (p=0.01), while radial vBMD (p=0.27) and radial cortical thickness (p=0.27) were unchanged. Body weight decreased by 8.8% (p<0.001). There were no dropouts and no serious adverse events related to semaglutide.

**Conclusions:** In contrast to what was anticipated, semaglutide did not increase bone formation. Rather, semaglutide increased bone resorption, resulting in significant bone loss. These data raise concerns about the use of GLP-1Ras in patients with increased fracture risk.

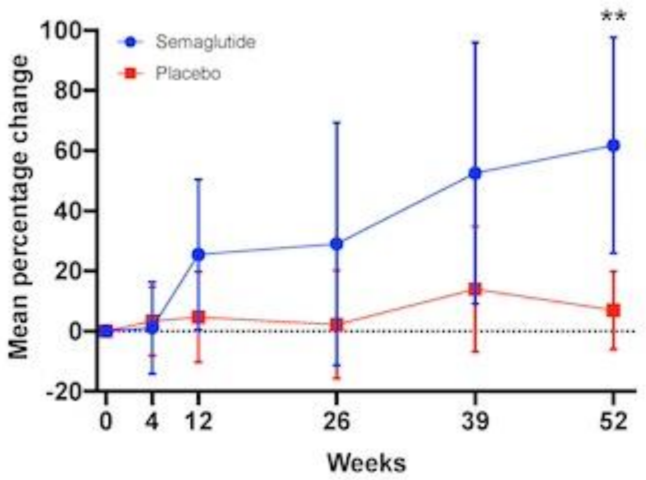

**Figure 1.** Mean percentage change from baseline for the bone resorption marker P-CTX. Semaglutide increased P-CTX by 55% compared to placebo from baseline to week 52. Data shown as mean with 95% CI. **, p<0.01

**P116**

**High-fat diet-treated C57BL/6J and A/J mice manifest different impact on bone and metabolic parameters**

*Michaela Ferencakova[1], Andrea Benova[1,2], Martina Dzubanova[1,2], Jan Prochazka[3], Frantisek Spoutil[3], Kristina Bardova[4], Martin Rossmeisl[4], Jan Kopecky[4], Michaela Tencerova[1]*
*[1]Institute of Physiology of the Czech Academy of Sciences, Laboratory of Molecular Physiology of Bone, Prague, Czech Republic ; [2]Charles University, Faculty of Science, Prague, Czech Republic ; [3]Institute of Molecular Genetics of the Czech Academy of Sciences, Czech Centre for Phenogenomics & Laboratory of Transgenic Models of Diseases, Prague, Czech Republic ; [4]Institute of Physiology of the Czech Academy of Sciences, Laboratory of Adipose Tissue Biology, Prague, Czech Republic*

**Abstract Text**

**Introduction:** Obesity-associated bone fractures are not manifested in all obese individuals and they are classified as metabolically healthy obese (MHO). MHO represents 10-30% of obese individuals as a transient phenotype to the development of metabolic diseases, including bone fractures and diabetes. However, the molecular mechanisms behind a link between obesity and bone diseases are not well described. Thus, we investigated the impact of obesity on bone and bone marrow stromal cells (BMSCs) metabolism in obesity-prone C57BL/6J (B6) mice and obesity-resistant A/J mice.

**Methods:** Eight-weeks-old B6 and A/J male mice were fed chow or high-fat-diet HFD (60 % kcal fat) (n=12/ group) for 3 months and examined for metabolic (glucose tolerance, body composition) and bone parameters (uCT, BMAT evaluation). Lipidomic analysis in bone marrow (BM) was performed by LC-MS. In addition, bioenergetic profile of mouse BMSCs was performed.

**Results:** After HFD treatment, B6 mice gained more weight and had impaired glucose tolerance ($p<0.001$), while A/J mice did not worsen their glucose metabolism ($p<0.05$). Further, µCT and histology analysis showed trabecular bone loss and increased bone marrow adiposity in tibia of B6 vs. A/J mice ($p<0.01$) . Metabolomic analysis of BM revealed HFD-induced increased levels of Cholesterol Esters and decreased levels of N-Acetylcytidine and Sphingomyelins in B6 vs. A/J mice ($p<0.001$), suggesting a worse impact on bone metabolism in B6 mice. Moreover, measurement of oxygen consumption rate of primary BMSCs showed increased maximal respiration in HFD B6 mice vs. A/J mice ($p<0.01$).

**Conclusion:** Our findings show that obesity induces an opposite impact on bone and fat metabolism in B6 and A/J mice. Moreover, decreased maximal respiration in A/J BMSCs suggests slowing down of their metabolic activity, which may contribute to prevention of HFD-induced bone loss in A/J mice. However, further analyses are needed to uncover the mechanism behind this phenomenon.

**P117**

### Glutamine supplementation in high fat diet improves bone and metabolic parameters in obese mice

*<u>Martina Džubanová</u>[1,2], Andrea Benova[1,2], Michaela Ferencakova[1], Michaela Vodickova[1], Dung Kim Nguyen[1], Heleen Fehervary[3], Harry Van Lenthe[3], Michaela Tencerova[1]*
*[1]Institute of Physiology of the Czech Academy of Sciences, Laboratory of Molecular Physiology of Bone, Prague, Czech Republic ; [2]Charles University, Faculty of Sciences, Prague, Czech Republic ; [3]KU Leuven, Department of Mechanical Engineering, Leuven, Belgium*

**Abstract Text**

**Background and purpose**: Glutamine plays an important role in the regulation of cellular metabolism and tissue homeostasis. Obesity reduces circulating glutamine levels and impairs peripheral adipose tissue (AT) function, leading to ectopic fat accumulation in bones and increasing fracture risk. Glutamine supplementation has shown positive effects on immune system and metabolism. However, the underlying mechanisms improving bone and fat metabolism remain unclear. Thus, this study investigates the impact of a glutamine-enriched high-fat diet (HFD+Gln) on bone and fat metabolism in mice.

**Methods**: Eight-week-old C57BL/6J male mice were fed 60% HFD, HFD supplemented with 4% glutamine (HFD+Gln), and a 10% low-fat diet (LFD) as a control for two months (n=11-14 per group). Metabolic parameters, body composition, and bone parameters were assessed, alongside with gene expression analysis. Primary adipose tissue-derived mesenchymal stem cells (AT-MSCs) isolated from treated mice were subjected to bioenergetic assays, senescence, and reactive oxygen species (ROS) measurement.

**Results**: HFD+Gln-treated mice gained less body weight (~27%, $p<0.01$), decreased fat mass (~8%, $p<0.05$), and improved glucose tolerance (~15%, $p<0.05$) compared to HFD mice. Inflammatory gene expression in AT (IL1-β ~40%; TNFα ~35%, $p< 0.05$) and adipogenic gene expression (Cidea ~77%, $p<0.05$) in liver were lowered in the HFD+Gln vs. HFD group. Additionally, primary AT-MSCs showed decreased senescence and ROS production in HFD+Gln vs. HFD group (~40%, $p<0.01$; ~25%, $p< 0.001$, respectively). Total body bone mineral content was improved in HFD+Gln group (~15%, $p<0.05$) accompanied by higher bone volume in L5 vertebrae in HFD+Gln vs. HFD group (6%, $p< 0.05$). Further, glutamine supplementation increased ultimate bone strength and fracture load (~15%, $p<0.01$; 21% $p<0.05$, respectively).

**Conclusion**: This study suggests that glutamine-enriched diet positively influences metabolic and bone parameters along with reduced AT inflammation and senescence in mice. These findings highlight the potential use of glutamine in the treatment of metabolic bone diseases.

**P118**

**In SOPF conditions, female BALBc mice have greater bone toughness and altered ovariectomy-induced bone fragility**

*<u>Hélène Racapé</u>*[1]*, Béatrice Bouvard*[1]*, Guillaume Mabilleau*[1]
[1]*University of Angers, Inserm UMR_S 1229 - REGOS team, Angers, France*

**Abstract Text**

**Background:** Evaluation of the role of gut microbiome on bone homeostasis or induced osteoporosis has been explored both in specific pathogen-free (SPF) conditions and germ-free conditions. However, the gut microbiota of mice raised under SPF or conventional conditions differs, and few studies compare bone health and ovariectomy-induced bone loss between these two sanitary conditions.

**Purpose:** To evaluate the effects of specific opportunistic pathogen-free (SOPF) conditions in bone health and ovariectomy-induced bone loss in mice.

**Methods:** All animals procedures were approved by the regional ethical committee for animal use. 12-week-old female BALBc mice underwent bilateral ovariectomy. Sham-operated mice served as controls. They were maintained under SOPF or conventional conditions during 8 weeks before sacrifice. Three-point bending, vertebral compression and microCT were performed to assess bone strength and microarchitecture. Total bacterial and fungal mass in feces were assessed by 16S and ITS1 rRNA qPCR. One way ANOVA with Tukey post-hoc test has been performed and considered significant at $p<0.05$.

**Results:** Sham animals maintained in SOPF showed increased bone strength in cortical (+ 21%, $p<0.001$) but not in trabecular bone compared with animals raised in conventional conditions. OVX led to reduced bone strength (-9%, $p=0.011$) and toughness (-31%, $p=0.036$) in conventional conditions. However, SOPF conditions prevented cortical bone fragility associated with OVX but showed a greater decrease in trabecular bone strength (-31%, $p<0.05$).

**Conclusion**: Bone health and ovariectomy-induced bone loss differed significantly according to sanitary conditions and this must be taken into account when studying interactions between microbiota and bone in animals maintained under SOPF conditions.

**Figure**. Bone strength at the femoral shaft (cortical bone) and the fourth lumbar vertebra (trabecular bone)

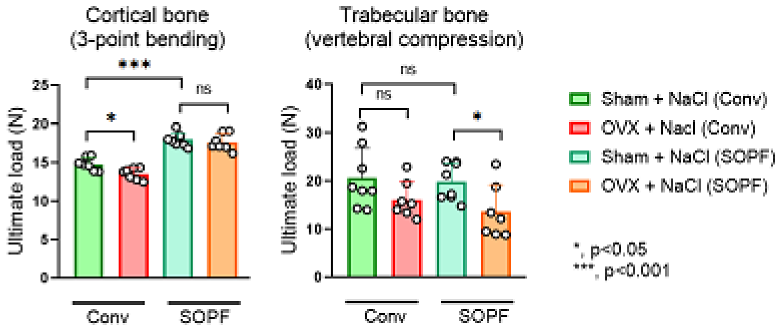

**P119**

**Intermittent fasting supports elevation of alkaline phosphatase activity and adipogenesis of bone marrow mesenchymal stromal cells in middle-aged CBA mice**

*Drenka Trivanovic*[1], *Sanja Momčilović*[2], *Aleksandra Arsić*[3], *Slavko Mojsilović*[1], *Milica Rajković*[1], *Tamara Kukolj*[1], *Srdja Janković*[4], *Aleksandra Jauković*[1]
[1]*Institute for Medical Research- University of Belgrade- Serbia, Group for Hematology and Stem Cells, Belgrade, Serbia ;* [2]*Institute for Medical Research- University of Belgrade- Serbia, Group for Neuroendocrinology, Belgrade, Serbia ;* [3]*Institute for Medical Research- University of Belgrade- Serbia, Centre of Research Excellence in Nutrition and Metabolism, Belgrade, Serbia ;* [4]*University Children's Hospital, Laboratory for immunology, Belgrade, Serbia*

**Abstract Text**

Alkaline phosphatase (ALP) deficiency induces premature bone aging features, marrow adiposity gain, and impaired differentiation in bone marrow mesenchymal stromal cells (BMSCs). Intermittent fasting (IF) has been described as anti-aging intervention mainly in C57BL/6 mouse strain. However, there is no data whether IF causes the interplay between ALP, bone marrow (BM) adipogenesis, and longevity molecules during aging.

In this study, female and male CBA mice were randomly assigned to the ad libitum (AL) or IF regimen initiated in 12 months-old mice (aged) for 30 days (Ethical approval 120-01-511412024-0). Fatty acids (FAs) were assessed by gas chromatography. Ex vivo cellular and molecular assays were performed at 3% oxygen. Data were compared to young (3 months-old) and aged (12 months-old) mice groups fed AL. Values are presented as mean ± SD (n=36) and significance was estimated by Mann-Whitney test.

IF reduced aging-related increase of total body weight (p=0.039) and visceral fat (p=0.056) in male mice only. In both sexes, IF significantly reduced blood plasma n-6/n-3 polyunsaturated FAs ratio, following decline in arachidic, oleic, and linoleic FA levels. Using C11-BODIPY$^{581/591}$ lipid peroxidation reporter molecule, we showed that IF abolished aging-related lipid peroxidation in BM cells in both sexes. In females, the frequency of dividing Sca-1$^{+}$CD24$^{med}$ marrow cells was increased in aged group (young: 1.51±0.44%; aged: 2.04±0.15%), and this was additionally stimulated by IF (6.37±2.97%, p=0.047). IF increased ex vivo clonogenic capacity of BMSCs in both sexes, coupled with elevated activity and protein expression of ALP and adipogenesis in BMSCs. In females, IF reverted age-related changes in subcellular localization of Sirt1, PPARγ, and FoxO3 in BMSCs.

Our study elucidated the existence of sex-dependent response of CBA mice to IF during aging, demonstrating that even in absence of IF effects on extramedullary fat depot, this dietary intervention can stimulate marrow ALP activity and adipogenesis.

**P120**

**Aging and obesity impact subcutaneous, visceral, liver, bone marrow, and muscle adiposity differently as measured with CT and MR imaging**

*Ling Wang*[1], *Yandong Liu*[2], *Glen Blake*[3], *Xiaoguang Cheng*[2], *Yajun Liu*[4], *Klaus Engelke*[5], *Annegreet Vlug*[6]
[1]*Beijing Jishuitan Hospital- National Center for Orthopaedics- Capital Medical University, Department of Radiology, Beijing, China ;* [2]*Beijing Jishuitan Hospital- National Center for Orthopaedics, Department of Radiology, Beijing, China ;* [3]*King's College London, School of Biomedical Engineering &*

*Imaging Sciences, London, United Kingdom ; [4]Beijing Jishuitan Hospital- National Center for Orthopaedics, Department of Spine Surgery-, Beijing, China ; [5]FAU University Erlangen-Nürnberg and Universitätsklinikum Erlangen, Department of Medicine 3, Erlangen, Germany ; [6]Leiden University Medical Center, Center for Bone Quality- Department of Internal Medicine, Leiden, Netherlands*

**Abstract Text**

**Objective:** We analyzed quantitative computed tomography (QCT) and chemical shift–encoded (CSE) magnetic resonance imaging (MRI) data from a Chinese cohort to investigate the effects of obesity and aging on different adipose tissue (AT) depots.

**Methods:** In 400 healthy community-dwelling volunteers aged 22 to 83 years we used MRI to quantify the proton density fat fraction (PDFF) of bone marrow adipose tissue (BMAT), psoas major (PS) and erector spinae (ES) muscles, and the liver. Abdominal total adipose tissue (TAT), visceral adipose tissue (VAT), and subcutaneous adipose tissue (SAT) areas were measured at the L2/L3 level with QCT. Partial correlation analysis was used to evaluate the relationship of each AT variable with age and body mass index (BMI). Multiple linear regression analysis was performed in which each AT variable in turn was evaluated as a function of age and the other five independent AT measurements.

**Results:** Of the 168 men, 29% had a normal BMI (< 24.0 $kg/m^2$), 47% were overweight (24.0-27.9 $kg/m^2$), and 24% were obese (≥ 28.0 $kg/m^2$). For the 232 women, the percentages were 46%, 32%, and 22%, respectively. Strong or very strong correlations with BMI were found for TAT, VAT, and SAT in both sexes. BMAT and ES PDFF were strongly correlated with age in women and moderately correlated in men. In both sexes, BMAT PDFF correlated only with age and with none of the other adipose tissues. Psoas PDFF correlated only with ES PDFF and not with age or the other adipose tissues. Liver PDFF correlated with BMI and VAT and weakly with SAT in men. VAT and SAT correlated with age and with each other in both sexes.

**Conclusions:** Age and BMI are both associated with adiposity, but their effects differ depending on the type of adipose tissue.

**P121**

### Caloric restriction (CR) causes bone loss in female 11β-Hydroxysteroid dehydrogenase type 1 (11Hsd1) global knockout (KO) mice, but not in male mice

*<u>Iana Mizumukai De Araujo</u>[1], Phuong T Le[2], Caroline De Carvalho Picoli[2], Rowan Hardy[3], Clifford J Rosen[2], Francisco JA De Paula[1]*
*[1]Ribeirão Preto Medical School, Internal Medicine, Ribeirão Preto, Brazil ; [2]MaineHealth Institute for Research, Center of Molecular Medicine, Scarborough, United States ; [3]University of Birmingham, School of Biomedical Sciences, Birmingham, United Kingdom*

**Abstract Text**

CR deeply affects body composition, including bone mass. The influence of cortisol on bone phenotype is remains to be elucidated. We aimed to test the effects of 30% CR on body composition and skeletal phenotype. Eight-week-old age-matched control (WT) and KO female and male mice were used. A total of 80 mice were divided into: WT *ad libitum diet* (AL), WTCR, KOAL, and KOCR. Body composition was assessed by FAXITRON-DXA. Bone microarchitecture in the right femur was examined through micro-computed tomography. Bone markers were determined by ELISA. Statistical analysis was performed using Graphpad Prism with one-way ANOVA followed by Tukey's post hoc test. CR mice lost lean mass in both sexes. CR males lost fat mass but maintained their bone mass. Females had preserved fat mass but lost bone mass. Females appear to have cortical trabecularization. In females, we observed that cortical porosity and BV/TV were higher in KOCR than in KOAL. Also, trabecular number (Tb.N) was higher while trabecular thickness (Tb.Th) and trabecular separation (Tb.Sp) were lower in CR in compared to AL groups. In addition, Tb.Th in KOCR is higher than in WTCR. In males, only Tb.Th is lower in CR groups. We observed that, in both sexes, CTX and P1NP were lower in CR (Table1), and serum levels of corticosterone were higher in KOCR compared to KOAL. In addition, in males, both WT and KOCR groups had elevated levels of corticosterone compared to the AL diet groups. This suggests that KO males appear to be protected from bone loss even at higher levels of corticosterone. Females maintained their fat mass and lost bone mass, which is the opposite of what occurs in males. The loss of bone mass that occurs in females appears to be due to cortical trabecularization, with increased cortical porosity measured by micro-CT.

| | Females | | | | Males | | | |
|---|---|---|---|---|---|---|---|---|
| | WT AL | WT CR | KO AL | KO CR | WT AL | WT CR | KO AL | KO CR |
| **Femur BMC (mg)** | 30.4±2.7 | 25.1±2.0# | 33.3±4.1 | 26.8±2.0## | 34.7±2.5 | 32.0±2.2 | 35.1±2.8 | 32.4±1.9 |
| **Cortical porosity. (%)** | 0.36±0.05 | 0.41±0.04 | 0.33±0.03 | 0.40±0.05* | 0.34±0.05 | 0.37±0.03 | 0.38±0.04 | 0.36±0.03 |
| **Tb.Th (mm)** | 0.05±0.003 | 0.04±0.002# | 0.06±0.002 | 0.05±0.001##¶ | 0.05±0.002 | 0.04±0.002# | 0.05±0.002 | 0.05±0.005##¶ |
| **Tb.Sp (mm)** | 0.26±0.04 | 0.22±0.02# | 0.28±0.02 | 0.22±0.02## | 0.15±0.01 | 0.15±0.02 | 0.15±0.01 | 0.16±0.01 |
| **Tb.N (1/mm)** | 4.0±0.6 | 4.6±0.5** | 3.7±0.3 | 4.6±0.6* | 6.2±0.5 | 6.3±0.8 | 6.2±0.4 | 6.0±0.4 |
| **BV/TV (%)** | 11.6±1.5 | 13.1±2.9 | 11.3±2.0 | 16.1±2.7* | 18.9±3.3 | 16.7±4.8 | 19.0±2.1 | 17.4±1.7 |
| **CTX (ng/mL)** | 36±19 | 16±10# | 23±6 | 15±5 | 26±11 | 13±2# | 26±6 | 16±2## |
| **P1NP (ng/mL)** | 55±25 | 32±19# | 47±12 | 34±15 | 30±9 | 18±7 | 42±17 | 24±12## |
| **Corticosterone (pg/mL)** | 2059±1509 | 2285±1247 | 2866±1412 | 7732±6853*¶ | 1000±385 | 2765±301** | 1805±556 | 4031±2148* |

* KO CR > KO AL, p>0.05; ** WT CR > WT AL; # WT CR < WT AL; ## KO CR < KO AL; ¶ KO CR > WT CR

BMC: bone mineral content, Tb.Th: trabecular thickness, Tb.SP: trabecular space, Tb.N: trabecular number, BV/TV: bone volume relative to total tissue volume.

**P122**

**Effect of chrysin on changes in the rat skeletal system induced by experimental type 1 diabetes**

*Piotr Londzin[1], Weronika Borymska[2], Maria Zych[2], Ilona Kaczmarczyk-Żebrowska[2], Joanna Folwarczna[1]*

*[1]Medical University of Silesia - Katowice, Department of Pharmacology - Faculty of Pharmaceutical Sciences in Sosnowiec, Sosnowiec, Poland ; [2]Medical University of Silesia - Katowice, Department of Pharmacognosy and Phytochemistry - Faculty of Pharmaceutical Sciences in Sosnowiec, Sosnowiec, Poland*

**Abstract Text**

It is believed that some substances of plant origin may exert health-promoting activities in diabetes and its complications, including increased fracture risk. Chrysin, a representative of flavonoids, has been reported to exert, among others, antioxidative, antiinflammatory and antidiabetic effects. The aim of the study was to investigate the effects of chrysin on the skeletal system of rats with experimental type 1 diabetes (T1D).

The experiments were carried out on 3-month-old male Wistar rats, divided into four groups: I – healthy control rats, II – streptozotocin-induced diabetic control rats, III –diabetic rats receiving chrysin (50 mg/kg p.o.), IV – diabetic rats receiving chrysin (100 mg/kg p.o.). T1D was induced by a single streptozotocin injection (60 mg/kg i.p.). Administration of chrysin (once daily by oral gavage) started two weeks later and lasted four weeks. Bone mass, density and mineralization (in the tibia, femur and L4 vertebra), serum bone turnover markers, and mechanical properties of cancellous (the proximal tibial metaphysis) and compact (the femoral diaphysis) bone were examined.

T1D induced profound disorders of bone metabolism and deterioration of cancellous and compact bone strength. Administration of chrysin did not favorably affect the serum bone turnover markers, and bone mass and mineralization in the diabetic rats (increases in the tibia bone mineral content after the lower dose and L4 vertebra bone density after the higher dose were only noted). However, chrysin improved some bone mechanical properties: at the lower dose, it tended to increase Young's modulus in the proximal tibial metaphysis, and, at the higher dose, it increased the values of the yield point load and energy in the femoral diaphysis. There were no significant effects of chrysin on other bone mechanical parameters.

In conclusion, results of the present study indicate that chrysin may exert some limited favorable effects on the skeletal system in diabetic conditions.

**P123**

**Mechanical loading enhances the effect of insulin treatment on bone mass in young type 1 diabetic mice (Akita)**

*Indira Toillon*[1]*, Isabelle Badoud-Georges*[1]*, Serge Ferrari*[1]*, Maude Gerbaix*[1]
[1]*University of Geneva, Department of Medicine - Service of Bone Diseases, Geneva, Switzerland*

**Abstract Text**

**Background:** Type 1 diabetes (T1D) is recognized as an important risk factor for fragility fractures, despite initiation of insulin therapy upon diagnosis (Hough,2017). Mechanical loading is anabolic for bone and could therefore improve bone mass in T1D (Rubin,2006).

**Purpose:** To evaluate the effects of insulin treatment combined with mechanical loading on young T1D Akita mice bone parameters.

**Methods:** Three groups of 8 weeks-old mice: Akita$^{+/-}$, Akita$^{+/-}$ treated with insulin implants and WT littermates were subjected to dynamic axial compression (2 weeks, 6 sessions, 8N, 0.1 Hz, 40 cycles) on the left tibia (right tibia served as non-loaded control). Tibia microarchitecture was assessed by µ-CT, femur strength by three-point bending test, insulin and P1NP levels by ELISA.

**Results:** At 8 weeks-old, Akita$^{+/-}$ mice showed high fasting glycemia (27.07mmol/L, p<0.0001), insulinopenia (-90%, p<0.05), lower P1NP levels (-40%, p<0.01), slightly lower BV/TV (-20%) and lower Mid.Ct.Th (-11%, p<0.001), resulting in lower maximal breaking force (-13%, p<0.05) vs WT. The diabetic bone phenotype worsened in 10 weeks-old mice, with significant lower BV/TV, Tb.Th and Mid.Ct.Th (-50%, -23% and -20% p<0.01 respectively vs WT). From 2 weeks of insulin treatment, fasting glycemia, insulin and P1NP levels were restored. From 4 weeks of treatment, Tb.Th, Ct.Th and maximal breaking force were improved (+20%, +18% and +19%, p<0.01 respectively vs untreated mice). Mechanical loading improved Mid.Ct.Th in both untreated and treated mice ($p_{Loading}<0.05$, $p_{Insulin}<0.001$,$p_{Int}$NS). However, at trabecular site, the biomechanical response to loading was higher in insulin-treated mice (BV/TV: +38%, p<0.01 vs unloaded tibia) than in untreated mice (BV/TV: +15%, pNS vs unloaded tibia).

**Conclusions:** T1D is associated with progressive bone microstructural alterations that are partially improved by insulin. Interestingly, combining insulin treatment with mechanical loading fully restores the trabecular bone microarchitecture. Physical exercise could therefore be a key factor in the management of type 1 diabetic bone disease.

**P124**

**Estrogen suppresses adipocyte differentiation by inhibiting ADAR2-mediated RNA editing**

*Hiroaki Saito*[1,2], *Tang Rui*[1,2], *Hanna Taipaleenmäki*[1,2], *Eric Hesse*[1,2]
[1]*University Hospital- LMU Munich, Institute of Musculoskeletal Medicine, Munich, Germany*
*; [2]University Hospital- LMU Munich, Musculoskeletal University Center Munich, Munich, Germany*

**Abstract Text**

Upon menopause, 17-β-estradiol (estrogen) serum concentration declines. Since estrogen has restrictive effects on adipocyte differentiation, its deficiency contributes to a relative increase in adipocytes. However, the underlying molecular mechanisms are not yet fully elucidated. We observed a significant increase ($p<0.05$) in the expression of the RNA editor adenosine deaminases acting on RNA-2 (ADAR2) in immortalized human mesenchymal stromal cells (hMSCs) during adipogenic differentiation. ADAR2 causes adenosine to inosine (A-to-I) editing at a specific site in the pre-mRNA encoding glutamine (Q) of the glutamate ionotropic receptor AMPA type subunit 2 (GRIA2). This editing results in the conversion of glutamine (Q) to arginine (R) (Q/R editing) in the GRIA2 protein, rendering it calcium impermeable. Given that estrogen activates calcium signaling, we hypothesized that this mechanism might be involved in the suppressive effect of estrogen on adipocyte differentiation. Confirming our hypothesis, antagonizing ADAR2 expression in hMSCs using siRNA reduced Q/R editing from >80% to <20% during adipogenic differentiation. Additionally, ADAR2 inhibition constrained adipocyte differentiation, as evidenced by decreased oil red O staining and reduced expression of the adipocyte markers FABP4 and ADIPOQ ($p<0.05$). Furthermore, the impaired adipocyte differentiation was restored using an inhibitor of GRIA2 (IEM-1460), and antagonizing ADAR2 was associated with an increased calcium influx in hMSCs ($p<0.05$). Conversely, stimulating hMSCs with 10mM calcium or 10nM estrogen increased intracellular calcium influx while estrogen also decreased ADAR2 expression. Consistently, estrogen inhibition using the aromatase inhibitor anastrozole increased ADAR2 expression in hMSCs, restored editing of GRIA2 at the Q/R site, and enhanced adipocyte differentiation. These findings unveil a novel mechanism by which ADAR2-mediated Q/R editing of GRIA2 reduces intracellular calcium influx, thereby promoting adipocyte differentiation. The reduction in ADAR2 expression induced by estrogen might contribute to its inhibitory effect on adipocyte differentiation and the subsequent relative increase in adipocytes after menopause.

**P125**

**Long-term effects of weight restoration on bone mineral density (BMD) in patients with anorexia nervosa**

*Giovanni Adami*[1], *Angelo Fassio*[1], *Calugi Simona*[2], *Manuela Molgora*[2], *Mirko Chimini*[2], *Barbara Segattini*[2], *Davide Gatti*[1], *Anna Dalle Grave*[2], *Riccardo Dalle Grave*[2]
[1]*University of Verona, Rheumatology Unit, Verona, Italy ; [2]Casa di Cura Villa Garda, Eating and weight disorders unit, Verona, Italy*

**Abstract Text**

**Background:** Anorexia nervosa is an eating disorder characterized by extremely low body mass index (BMI) with consequent low bone mineral density (BMD) and higher risk of fractures.

**Purpose:** To investigate the long-term effects of body weight restoration on BMD in patients with anorexia nervosa.

**Material and methods:** We conducted a prospective observational study of patients with anorexia nervosa admitted for intensive weight restoration program, inpatient (20-week program) followed by an outpatient long-term follow-up. Clinical, demographic, body composition and BMD data were collected at baseline (admission), at week 20 (W20) and after >5 years.

**Results:** 53 women were enrolled in the study. Mean age at baseline was 32±9.2 years and the median follow-up was 8.0 years (IQR 9.1-7.0). Mean BMI at baseline was 15.8±1.7. Lumbar spine BMD Z-score at baseline was -0.72±1.14, femoral neck Z-score at baseline was -0.37±0.97 and total hip Z-score at baseline was -0.40±1.06. All patients had normal (>20 ng/mL) 25-OH-vitamin D levels throughout the study. All subjects achieved BMI ≥18 at W20 and in aggregate BMD increased at all sites at W20. However, over a median follow-up of 8.1 years (IQR 7.3-8.6), 14 patients had BMI <18 (weight loss after discharge), whereas 39 subjects kept BMI ≥18 over a median follow-up of 8.0 years (IQR 6.9-9.2). **Figure 1A** shows the cumulative probability of losing BMD at various sites stratified by maintenance of BMI above or below the threshold of 18. **Figure 1B** shows the trend in BMD levels at the femur and lumbar spine in patients that maintained BMI≥18 or <18.

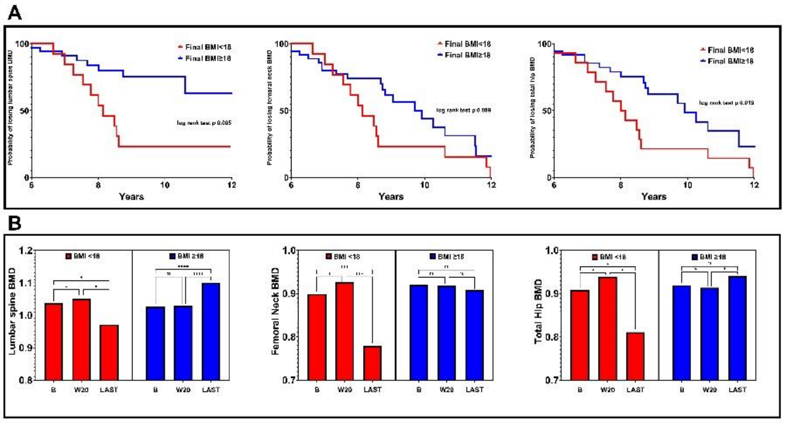

**Conclusions:** In aggregate, short-term weight restoration was associated with a significant increase in BMD at all sites. Keeping BMI ≥18 in the long term was associated with a positive non-plateau effect on lumbar spine BMD. In contrast, weight loss after discharge was associated with significant bone loss at all sites.

**P126**

**High glucose levels negatively affect bone formation and regeneration in zebrafish larvae**

*Tessa Krappa[1], Livia Sztraka[2], Alejandra Cristina López-Delgado[1], Prateek Chawla[2], Lorenz Hofbauer[3], Nikolay Ninov[2], Franziska Knopf[1]*
*[1]Center for Regenerative Therapies TU Dresden CRTD- TU Dresden- 01307 Dresden- Germany, Center for Healthy Aging- Faculty of Medicine Carl Gustav Carus- TU Dresden- 01307 Dresden- Germany, Dresden, Germany ; [2]Center for Regenerative Therapies TU Dresden CRTD- TU Dresden- 01307 Dresden- Germany, Paul Langerhans Institute Dresden of the Helmholtz Zentrum München at the University Hospital and Faculty of Medicine Carl Gustav Carus of Technische Universität Dre, ; [3]University Medical Center- Technische Universität Dresden- Dresden- Germany, Division of Endocrinology- Diabetes and Bone Diseases- Department of Medicine III- Center for Healthy Aging- Faculty of Medicine Carl Gustav Carus- TU Dresden- 01307 Dresden-,*

**Abstract Text**

Diabetes is a common cause of bone fragility leading to significant morbidity and mortality among affected patients. While bone mass and bone strength are reduced in type 1 diabetes, patients with type 2 diabetes have an increased risk of fracture despite normal bone mass. The pathophysiologic mechanisms are multifactorial and not yet fully understood. The zebrafish is an established model organism in the fields of diabetic and skeletal research. However, there are only few studies investigating bone under diabetic metabolic conditions in zebrafish. The aim of this study is to assess whether bone tissue formation is affected by high glucose in zebrafish larvae similarly to human bone in diabetes.

1- 5 day old zebrafish larvae were used to study the effect of elevated glucose levels on bone development and regeneration after osteoblast ablation by assessing the number of osteoblasts and mineralized area. Furthermore, activation of the immune system following laser assisted osteoblast ablation was examined, specifically focusing on the recruitment of macrophages and neutrophils. High glucose conditions were enabled by either incubation or nitroreductase-mediated cell ablation of ß-cells, respectively.

High glucose conditions lead to impaired bone formation during development of zebrafish larvae with 49.75 (glucose) vs. 57.50 (control) osteoblasts (p=0.049), characterized by decreased mineralized bone matrix area with 2956µm$^2$ (glucose) vs. 4047µm$^2$ (control) (p=0.0174). Likewise, osteoblast number was decreased during bone regeneration following osteoblast ablation with [MOU1] 69.75 (glucose) vs. 86.75 (control) osteoblasts (p=0.007). Glucose treated zebrafish also showed increased recruitment of macrophages with a total area of 2772µm$^2$ (glucose) vs. 1804µm$^2$ (control) (p=0.022) covered with macrophages and neutrophils with 4.8 (glucose) vs. 1.4 (control) cells (p=0.027) per injured bone.

These findings are consistent with some observations in diabetic patients and suggest that zebrafish larvae serve as a suitable model to address skeletal complications in the context of diabetes.

**P127**

**The functional and metabolic role of the glycolytic regulator PFKFB3 in skeletal progenitors**

*Lore Rosseels[1], Ingrid Stockmans[1], Karen Moermans[1], Lina Blockx[2], Pieter-Jan Coolen[2], Peter Carmeliet[2], Geert Carmeliet[1], Steve Stegen[1]*
*[1]KU Leuven, Clinical and Experimental Endocrinology CHROMETA, Leuven, Belgium ; [2]KU Leuven, Laboratory of Angiogenesis and Vascular Metabolism VIB-CCB, Leuven, Belgium*

**Abstract Text**

**Introduction:** Glycolysis, the conversion of glucose into lactate, is considered to be the predominant mode of glucose metabolism in osteolineage cells, although the functional role of this metabolic pathway has not yet been formally tested *in vivo*.

**Purpose/methods:** Our goal was to investigate the role of glycolysis in osteolineage cells, through genetic inactivation of the allosteric regulator phosphofructo-2-kinase/fructose-2,6-biphosphatase 3 (PFKFB3) using Prrx1-Cre driver mice (Pfkfb3sp-). Cre-negative mice (Pfkfb3sp+) were used as wild-type control.

**Results:** PFKFB3 deletion in skeletal progenitors resulted in decreased glycolysis, as evidenced by 13C-glucose tracing (lactate 13C fractional contribution: -30.2%, p<0.05), glycolytic flux (-41.1%, p=0.11) and Seahorse analysis (extracellular acidification rate: -46.1%, p<0.05). Despite the reduction in glycolysis, male Pfkfb3sp- mice showed normal trabecular bone volume (Pfkfb3sp+ 11.3% vs Pfkfb3sp- 12.1%, p=0.65) and cortical thickness (Pfkfb3sp+ 140.6µm vs Pfkfb3sp- 133.5µm, p=0.27) at 8-weeks-of-age. A similar phenotype was observed at 4-weeks-of-age and in female mice. *In vitro* proliferation of isolated PFKFB3-deficient skeletal progenitors, as well as their differentiation into mature osteoblasts, was comparable to control, indicating compensatory mechanisms. Indeed, although glycolytic flux was reduced, bioenergetics (AMPK phosphorylation) and biosynthesis (extracellular matrix content) were not affected, suggesting the use of other metabolic substrates and pathways. In accordance, metabolomics analysis on spent medium revealed a trend towards increased uptake of pyruvate (+33.5%, p=0.16), which was subsequently more converted to TCA cycle intermediates and amino acids, as shown by 13C-pyruvate tracing. Intriguingly, while osteogenesis was not affected, adipogenic differentiation of PFKFB3-deficient progenitors was increased, which may be explained by enhanced glucose and pyruvate-mediated acetyl-CoA synthesis and subsequent lipogenesis, although this hypothesis requires further testing.

**Conclusion:** Collectively, our data show that deletion of PFKFB3 in skeletal progenitors does not affect osteogenesis and bone properties, likely because extracellular pyruvate can compensate and thereby highlighting their metabolic flexibility.

### P128

### Omega-6 Arachidonic acid is increased in Osteoarthritis Bone Marrow Adipose Tissue and negatively impacts osteogenesis.

*Léa Loisay[1], Diego De Haro[1], Elke Moradpour[1], Antoniadis Alexander[2], Thomas Hügle[1], Jeroen Geurts[1]*
*[1]Lausanne University Hospital, Department of Rheumatology, Lausanne, Switzerland ; [2]Lausanne University Hospital, Department of Orthopaedics and Traumatology, Lausanne, Switzerland*

**Abstract Text**

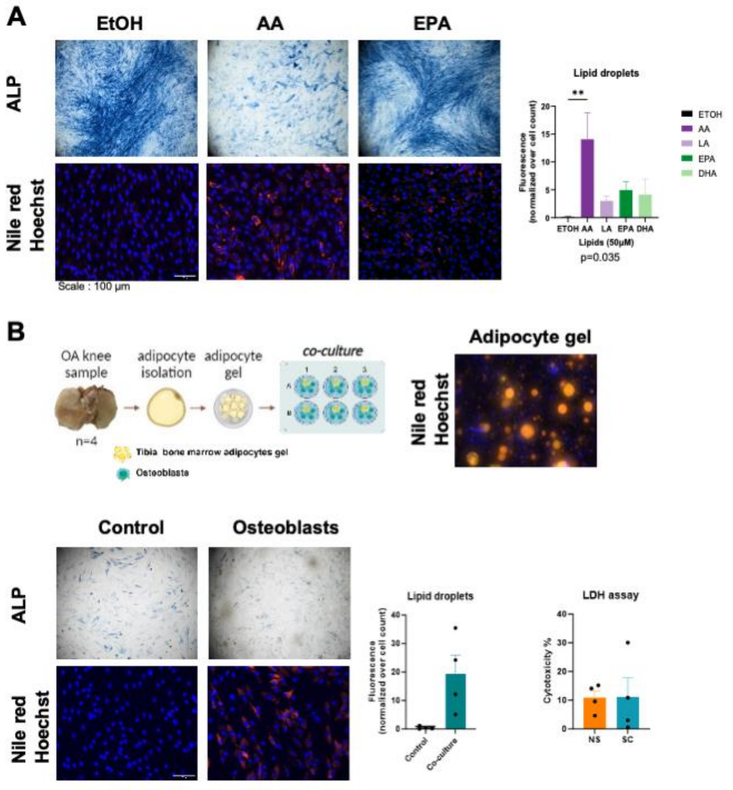

**Purpose:** Osteoarthritis (OA) is a degenerative joint disease marked by subchondral bone remodeling and cartilage degeneration. In human OA, omega-6 lipids have been implicated in exhibiting a pro-inflammatory effect in cartilage and synovial fluid. However, the lipid species secreted by Bone Marrow Adipose Tissue (BMAT) and their role in OA remain unclear. This study aims to profile BMAT lipids in OA subchondral bone and assess their impact on osteogenesis.

**Methods:** Lipidomic analysis of eicosanoid species was performed on the secreted media of non-sclerotic and sclerotic OA knee bone explants (n=7). *In vitro* stimulation for 7 days using osteoblasts isolated from OA samples (n=5) with omega-6 arachidonic acid (AA) or omega-3 eicosapentaenoic acid (EPA), or co-culture with primary mature bone marrow adipocytes (n=4). Viability (*TP53)*, bone marker (*ALPL)* and inflammation (*IL-6, IL-8*) levels were assessed through qPCR. Osteogenesis, lipid uptake and viability were validated with ALP, Nile Red staining and LDH assay.

**Results:** Lipidomics identified increased AA (+71 %; p=0.08) versus decreased EPA (-31,9%; p=0.04) in sclerotic samples. Osteoblasts showed lipid uptake in the presence of AA and EPA (Figure 1A) and with adipocyte co-culture (Figure 1B). No lipotoxicity was observed in any of the conditions. Opposite effects were observed for AA and EPA on osteogenic and inflammatory genes. With AA, *ALPL* gene expression decreased significantly (0.5 fold; p=0.0226), accompanied by an increase in *IL6* (2 fold; p=0.0011) and *IL8* (8 fold; p=0.0048). In the presence of EPA, *IL6* was reduced (0.5 fold; p=0.0449). Finally, we also found a strongly decreased ALP staining in the co-culture experiments (Figure1B).

**Conclusion:** BMAT-secreted lipids are taken up by osteoblasts and exert species-dependent effects on osteogenesis and inflammation. Taken together, these data suggest that lipids secreted by BMAT, specifically omega-6 arachidonic acid, may play a role in aberrant bone formation in OA.

**P129**

**Lipolysis in bone marrow adipocytes and osteoblasts is involved in knee Osteoarthritis subchondral bone sclerosis.**

*Léa Loisay[1], Sabrina Bouagga[1], Gowsika Kanagasabai[1], Alexander Antoniadis[2], Elke Moradpour[1], Thomas Hügle[1], Jeroen Geurts[1]*
*[1]Lausanne University Hospital, Department of Rheumatology, Lausanne, Switzerland ; [2]Lausanne University Hospital, Department of Orthopaedics and Traumatology, Lausanne, Switzerland*

**Abstract Text**

**Purpose:** Osteoarthritis (OA), a degenerative joint disease, is marked by abnormal subchondral bone remodeling. In mice, it has been proven that bone marrow adipose tissue (BMAT) and adipocytes (BMAds) are emerging as regulators of bone remodeling through lipolysis by releasing free fatty acids (FFA) and glycerol. However, BMAT lipolysis has not been investigated in the context of OA. In this study, we aim to evaluate the role of BMAT in pathological bone formation in OA.

**Methods:** Resection specimens were collected from non-sclerotic and sclerotic tissue of patients (n=22) undergoing knee replacement. BMAd size and bone fraction histological measurement were performed using MarrowQuant. Expression of adipose triglyceride lipase (ATGL), phosphorylated hormone-sensitive lipase (p-HSL) and monoacylglycerol lipase (MGLL) was assessed by immunohistochemistry (n=10) and verified by qPCR on primary osteoblasts (n=5). Osteochondral explants culture was performed on a subset cohort (n=10). Alkaline phosphatase and BMAd-derived factors (FFA, glycerol) were measured in supernatants.

BMAd size (-40%; p<0.0001) was reduced in sclerotic tissue, accompanied by an increase in the bone fraction (+54%; p<0.001). Secretion of ALP (2.3-fold), FFA (1.5-fold), and glycerol (1.6-fold) was significantly elevated in sclerotic tissues. The reduction in BMAd size correlated with increased ALP activity (r = -0.64) and decreased FFA levels (r = 0.78). The highest increase in osteoblast activity was associated with the smallest increase in FFA secretion (r = -0.81), suggesting enhanced FFA uptake by osteoblasts. Lipolysis enzyme expression was heightened in BMAds and osteoblasts in sclerotic areas. Interestingly, qPCR confirmed the presence of these three enzymes inosteoblasts, with a higher expression of *MGLL* (0.004) and *ATGL* (0.01931).

**Conclusions:** These data suggest that BMAd-derived factors may act in regulating pathological bone remodeling in OA, through lipolysis by BMAT on osteoblasts as a source of energy to sustain increased bone remodeling.

**P130**

**Type 2 Diabetes alters Alveolar Bone Morphology and Microarchitecture**

*William Ndjidda Bakari*[1], *Rebecca Landon*[1], *Seddik Kchaou*[1], *Morad Bensidoum*[1], *Fani Anagnostou*[1]
[1]*University of Paris, Université Paris Cité - CNRS UMR7052- INSERM U1271- ENVA- B3OA, Paris, France*

**Abstract Text**

**Background**: Type 2 diabetes (T2D) is a systemic, chronic, progressive disease affecting several organs, including bone. It is associated with skeletal health disorders, including osteoporosis, fragility, and poor bone healing. In the maxillofacial area, it is associated with periodontal, peri-implant bone loss, but data on the impact of the T2DM progression on maxillofacial bone quality are scarce.

**Purpose:** To evaluate the impact of T2D progression on alveolar/jaw bone structure and microarchitecture using Zucker diabetic fatty (ZDF) rats a T2D animal model with musculoskeletal complications.

**Methods:** Characteristics of the alveolar/jaw of ZDF rats (n=8) and their Lean (n=8) (ZL) age-matched controls were assessed at 7w (prediabetic) and 23w (long-standing diabetes) by micro-CT and histological analyses.

**Results:** At 23w, compared to ZL, the mandibles of ZDF rats exhibited significantly smaller dimensions (area, $mm^2$: 226.3±9.6 vs. 250±8.5; height, mm: 11.9±0.24 vs. 12.6±0.27; length, mm: 29.9±0.33 vs. 31.7±0.65, p<0.05), and decreased distance between the alveolar bone crest and the enamel-cement junction (mm: 0.58±0.03 vs. 0.97±0.07, p<0.05). Moreover, independently of the diabetes condition, compared to ZL, ZDF rats showed denser alveolar trabecular bone (BV/TV: 12.3% and 13.6% higher respectively at 7w and 23w, P<0.05), less porosity (Po (tot): 18.6% and 32.6% lower at 7W and 23W respectively, P<0.05) and higher trabecular thickness (TbTh: 17.6% and 18.2% increase at 7W and 23W, P<0.05). Besides, ZDF rats' trabecular bone volume and the number of trabeculae per mm were significantly lower by 21.2% and 0.97% respectively at 23w. Moreover, at both alveolar and mandibular locations, while the number the osteocyte lacunae remained unchanged, the number of the occupied ones was increased in the ZDF rats compared to the ZL.

**Conclusion:** our results provide evidence that T2D impairs the structure and quality of the alveolar/jaw bone since the early stages and deteriorates as the disease progresses.

**ND-P016**

**Prevalence of osteoporosis among type 1 diabetes patients**

*<u>Jakob Starup-Linde</u>[1], Julie Støy[2], Bente Langdahl[3], Torben Harsløf[1]*
*[1]Aarhus University Hospital, Department of Endocrinology and Internal Medicine, Aarhus N, Denmark ; [2]Aarhus University Hospital, Steno Diabetes Center Aarhus, Aarhus N, Denmark ; [3]Aarhus University Hospital, Department of Endocrinology and Internal Medicíne, Aarhus N, Denmark*

**Abstract Text**

**Background:** Type 1 diabetes (T1D) is associated with an increased fracture risk. Recent American Diabetes Association (ADA) guidelines recommend a treatment threshold of bone mineral density (BMD) T-score ≤-2.0.

**Purpose:** In this study we aim to investigate the prevalence of osteoporosis (DXA) in T1D ≥50 years.

**Methods:** In this cross-sectional database study we collected data from routine clinical DXA which was implemented in the period 2020-2022 and offered to men and postmenopausal women with T1D ≥50 years seen in the diabetes outpatient clinic. DXA of the lumbar spine and hip were performed by cross-calibrated Hologic Horizon scanners. Information on biochemical measures and osteoporosis risk factors were collected from questionnaires and electronic health records. Adherence to prescribed alendronate was audited using the prescription database. Permission for data collection was received from Region Central Jutland, Denmark.

**Results:** In total 640 T1D individuals were investigated. Of these 17.8 % had osteoporosis based on a T-score ≤-2.5 and 28.6 had osteoporosis based on a T-score ≤-2.0. In multivariate analysis, increasing age (1 year) (OR=1.3, 95 % CI :1.0;1.7) and a family history of osteoporosis (OR=1.9, 95 % CI :1.2;3.0) were associated with an increased risk of osteoporosis, whereas increase in BMI (1 kg/m$^2$) were associated with a decreased risk of osteoporosis (OR=0.87, 95 % CI: 0.82;0.92). Figure 1 shows the distribution of osteoporosis based on different T-score thresholds. HbA1c levels and diabetes duration were not related to the presence of osteoporosis. Among those treated with alendronate (n=76) for osteoporosis the compliance rate was 92.7 %.

**Conclusion:** In conclusion, the present study finds an osteoporosis prevalence of 17.8 % in postmenopausal women or men ≥ 50 years of age with T1D and using a T-score ≤-2.0 28.6 % has osteoporosis. These findings illustrate that a high proportion of T1D has osteoporosis using ADA guidelines.

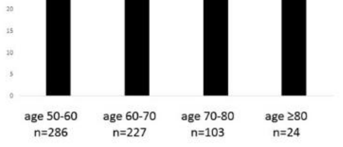

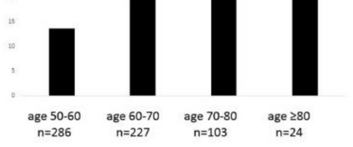

**ND-P017**

### Hip and knee arthroplasty. Nutritional characteristics and body composition

*Blanca Varas de Dios*[1], *María Martín Fuentes*[1], *Ana María Valverde Villar*[2], *José Ramón Lamúa Riazuelo*[3], *Iker Fernández-Navamuel Basozabal*[4]
*[1]Santa Cristina Universitary Hospital, Rheumatology, madrid, Spain ; [2]Santa Cristina Universitary Hospital, Orthopedics surgery, Madrid, Spain ; [3]Henares Hospital, Rheumatology, Madrid, Spain ; [4]Jimenez Diaz Foundation, Pulmolonogy, Madrid, Spain*

### Abstract Text

**Introduction:** Malnutrition,caused by either deficiency and/or excess of nutrients,is related to morbidity of the surgical patient.It has a major impact on muscle and bone health.The presence of osteosarcopenia implies less muscle,less bone mass and a weaker strength,which entails greater limitation of physical activity and a greater risk of falls and fractures.Within traumatology and orthogeriatry,the patient with hip fracture is classically studied from the nutritional point of view.The evaluation of patients in whom arthroplasty is performed is less common.

**Objectives:** To evaluate the nutritional situation and body composition in patients undergoing elective arthroplasty.

**Methods:** Observational cross-sectional study of patients admitted on a scheduled hip or knee replacement surgery, from February-September 2020. Test performed upon admission:Nutritional screening using the Malnutrition Universal Screening Tool(MUST),Anthropometric measurements,Manual dynamometry,nutritional parameters,Assessment of body composition with bioimpedanciometry,Spine x-ray and bone densitometry.We compared the clinical,anthropometric and body composition characteristics with the reference values established for age and sex.

**Results:** 86patients(61,6%women), mean age69.5±9.5years(72.1%≥65).71.6%knee surgeries and28.4%hip surgeries.Mean time of admission:4.9±1.3days.

According toMUST,21.3%of the patients were at risk of malnutrition(score≥2);16.9%had decreased triceps skinfold respect top50 and20% had a pathological hand-grip dynamometry.

The BMI was31.3±4.5. According to BMI:Normal weight3.8%,Overweight39.7%,Obesity56.4%.In91.4%,vitaminD was<30 pg/ml. Bioimpedanciometry, women presented significantly decreased muscle mass values.Age was correlated with a lower presence of fat-free mass,total and appendicular muscle mass.In those over65 years,52.6%of men vs14.3%of women had a decreased muscle mass index;58.5%had low bone mineral density.We observed vertebral bone collapses in13.9%.

**Outcomes:** There is a high prevalence of obesity in patients who are candidates for knee and hip arthroplasty. The presence of obesity doesn´t exclude the existence of a risk of malnutrition upon admission. They may present a decrease in muscle mass and strength that could condition their recovery. Special attention to those over 65 years old. They are the ones who most frequently show these alterations. Nutritional education and physical exercise recommendations are essential in these patients to optimize nutritional status for surgery.

**Poster Sessions: Genetics and epigenetics**

### P131

### IL-6 rs1800796 is associated with spine and hip BMD in Maltese postmenopausal women

*Maya Apap*[1], *Melissa M Formosa*[1]
[1]*University of Malta, Department of Applied Biomedical Science- Faculty of Health Sciences, Msida, Malta*

**Abstract Text**

**Background:** Interleukin-6 encoded by the *IL-6* gene, is a pro-inflammatory cytokine that increases osteoclastogenesis and bone resorption by upregulating the Receptor Activator of NF-Kb Ligand (RANKL). The *IL-6* rs1800796 variant (G>C) is located in the gene's promoter region, 572 bases upstream from the translational start site which is hypothesized to alter regulatory modifiers decreasing *IL-6* transcriptional activity.

**Purpose:** The study aimed to investigate the effect of *IL-6* rs1800796 on bone mineral density (BMD) and fracture risk at specific anatomical sites in Maltese individuals.

**Methods:** Genotyping was performed using the TaqMan® fluorogenic 5' nuclease allelic discrimination assay in 1,045 Maltese postmenopausal women from the Malta Osteoporotic Fracture Study (MOFS). Genotype-phenotype associations were analysed using the Mann-Whitney U test and Kruskal-Wallis test, whereas odds ratios (OR) with 95% confidence intervals (CI) were computed using logistic regression analysis adjusted for confounders.

**Results:** Genotyping was successful in 1,039 samples, with the G and C allele frequencies detected at a frequency of 87.9% and 12.1% respectively. Genotype-phenotype associations demonstrated that homozygosity for the alternative C allele exhibited a protective effect on BMD at the lumbar spine (LS BMD $p$=0.048), femoral neck (FN BMD $p$=0.039, FN T-score $p$=0.025) and total hip (TH BMD $p$=0.026, TH T-Score $p$=0.023) relative to women with the GG genotype. Additionally, women with the CC genotype had a decreased risk of low BMD at the LS (adj-OR: 0.207 [95% CI 0.055–0.775], $p$=0.019), FN (adj-OR: 0.203 [95% CI 0.041- 0.993], $p$=0.049) and TH (adj-OR: 0.207 [95% CI 0.041–0.993], $p$=0.019). No association between the *IL-6* rs1800796 variant and all-type of low-trauma fractures or site-specific fracture risk was observed.

**Conclusion:** Findings indicate that the *IL-6* rs1800796 C allele exerts a protective effect on spine and hip BMD, making it a potential underlying genetic determinant of bone mass in Malta.

### P132

### Transcriptomic analysis of coding and non-coding RNAs in modeled osteoporosis

*Elisavet Ioannidou*[1,2], *Vagelis Rinotas*[1], *Panagiotis Theofilidis*[1,2], *Marili Skalioti*[1,2], *Dimitra Andrikopoulou*[1,2], *Trias Thireou*[2], *Maria Yavropoulou*[3], *Eleni Douni*[1,2]
[1]*Biomedical Sciences Research Centre "Alexander Fleming", Institute for Bioinnovation, Vari, Greece ;* [2]*Agricultural University of Athens, Department of Biotechnology-Laboratory of Genetics, Athens, Greece ;* [3]*National and Kapodistrian University of Athens-Medical School, First Department of Propaedeutic and Internal Medicine-Endocrinology Unit, Athens, Greece*

**Abstract Text**

**Background:** RANKL is a key regulator in osteoclast development and bone resorption. Our lab has established a genetic mouse model of osteoporosis by overexpression of human RANKL in transgenic mice (TgRANKL) for the elucidation of the molecular basis in bone resorption.

**Purpose:** We aimed to identify and validate differentially expressed (DE) genes in TgRANKL mice with potential clinical value in osteoporosis.

**Methods:** We performed RNA-Seq for mRNAs, miRNAs, and lncRNAs in flushed femurs of TgRANKL and wild-type (WT) mice and identified DE genes (|log2FoldChange| > 1), while selected genes were validated with qPCR. All animal procedures were approved.

**Results:** We identified in total 2,747 DE mRNAs in TgRANKL femurs compared to WT. Enrichment analysis of the upregulated genes revealed correlation with protein degradation, transport, response to cytokines, cell adhesion, and bone remodeling, while downregulated genes were mainly related to metabolism, transport, cytoskeleton organization, muscle structure, and oxidative phosphorylation. STRING analysis revealed genes which act as hubs indicating their importance in osteoporosis. More than 50 upregulated genes have been validated in TgRANKL femurs with qPCR, while treatment of TgRANKL mice with Denosumab return their expression to normal levels. Furthermore, the expression of such genes was examined in osteoclasts and osteoblasts to identify the cellular source. We also identified 63 DE miRNAs and validated selected miRNAs in femurs and mouse sera with qPCR. Finally, we identified 648 DE lncRNAs, and the expression of 7 upregulated lncRNA genes was confirmed in TgRANKL femurs, 2 of which were expressed in osteoblasts.

**Conclusions:** The DE genes revealed in this study may serve as the basis for the discovery of novel pathogenic and epigenetic mechanisms in osteoporosis and the establishment of novel biomarkers.

*The research work was supported by the Hellenic Foundation for Research and Innovation (HFRI) under the 4th Call for HFRI PhD Fellowships (Fellowship Number: 10704).*

**P133**

**Apolipoprotein ε4 is associated with increased long-term risk of fall- and fracture-related hospitalisation in older women: the Perth Longitudinal Study of Ageing Women**

*<u>Jedd Pratt</u>[1], Jack Dalla Via[2], Craig Sale[1], Abadi Gebre[2], Blossom Stephan[3], Simon Laws[2], Kun Zhu[4], Wai Lim[4], Richard Prince[4], Joshua Lewis[2], Marc Sim[2]*
*[1]Manchester Metropolitan University, Department of Sport and Exercise Sciences- Institute of Sport, Manchester, United Kingdom ; [2]Edith Cowan University, School of Medical and Health Sciences, Perth, Australia ; [3]Curtin University, Curtin enAble Institute, Perth, Australia ; [4]The University of Western Australia, Medical School, Perth, Australia*

**Abstract Text**

**Background:** Genotypic screening has potential to enhance strategies designed to combat osteoporosis. Apolipoprotein ε4 (*APOE* ε4) status has promising value for identifying people at risk of poor bone and functional outcomes, although existing data is limited, and at times, conflicting.

**Purpose**: To examine the association between the presence of the *APOE* ε4 allele and long-term fracture- and fall-related hospitalisation risk in a large, well-characterised cohort of community-dwelling older women from Australia.

**Methods:** 1276 older women (mean age = 75.2±2.7 years), who were prospectively studied over 14.5 years as part of the Perth Longitudinal Study of Ageing Women, were included. Participants were genotyped for *APOE* and completed an in-depth health and lifestyle assessment at baseline. Fall-, any fracture-, and hip-fracture-related hospitalisations were obtained from linked health records. Multivariable-adjusted Cox-proportional hazard models were used to assess the data.

**Results:** Over 14.5 years of follow-up, 507 (39.7%) participants experienced a fall-related hospitalisation, 360 (28.2%) experienced a fracture-related hospitalisation, and 143 (11.2%) experienced a hip fracture-related hospitalisation. In the multivariable adjusted analysis, *APOE* ε4 carriers (n=297, 23.3%) had greater risk for a fracture-related hospitalisation (HR 1.30 95%CI 1.02–1.65), a hip fracture-related hospitalisation (HR 1.89 95%CI 1.32–2.68), as well as a fall-related hospitalisation (HR 1.50 95%CI 1.23–1.83), compared to non-carriers. These associations remained robust to further adjustment for various fall and fracture risk factors, such as plasma 25-hydroxyvitamin D, fraction undercarboxylated osteocalcin, grip strength, timed-up-and-go and hip bone mineral density.

**Conclusions:** Presence of the *APOE* ε4 allele was associated with increased risk of fracture- and fall-related hospitalisations in older women. Future consideration should be given to the potential utility of *APOE* ε4 screening for identifying older people who are vulnerable to negative musculoskeletal outcomes, and subsequently, guiding the early intervention and delivery of preventative measures.

**P134**

**PADI2 plays a crucial role in osteogenesis by enhancing RUNX2 stability through posttranslational citrullination**

*Hyun-Mo Ryoo*[1]*, Young-Dan Cho*[2]*, Woo-Jin Kim*[3]
[1]*Seoul National University, Molecular Genetics and Pharmacology, Seoul, Korea- South ;* [2]*Seoul National University Dental Hospital, Department of Periodontology, Seoul, Korea- South ;* [3]*Seoul National Unversity, Molecular Genetics and Pharmacology, Seoul, Korea- South*

**Abstract Text**

Peptidylarginine deiminase (PADI) 2 catalyzes the post-translational conversion of peptidyl-arginine to peptidyl-citrulline in a process called citrullination. However, the precise functions of PADI2 in bone formation and homeostasis remain unknown. In this study, our objective was to elucidate the function and regulatory mechanisms of PADI2 in bone formation employing global and osteoblast-specific Padi2 knockout mice. Our findings demonstrate that Padi2 deficiency leads to the loss of bone mass and results in a cleidocranial dysplasia (CCD) phenotype with delayed calvarial ossification and clavicular hypoplasia, due to impaired osteoblast differentiation. Mechanistically, Padi2 depletion significantly reduces RUNX2 levels, as PADI2-dependent stabilization of RUNX2 protected it from ubiquitin-proteasomal degradation. Furthermore, we discovered that PADI2 binds to RUNX2 and citrullinates it, and identified ten PADI2-induced citrullination sites on RUNX2 through high-resolution LC-MS/MS analysis. Among these ten citrullination sites, the R381 mutation in mouse RUNX2 isoform 1 considerably reduces RUNX2 levels, underscoring the critical role of citrullination at this residue in maintaining RUNX2 protein stability. In conclusion, these results indicate that PADI2 plays a distinct role in bone formation and osteoblast differentiation by safeguarding RUNX2 against proteasomal degradation. In addition, we demonstrate that the loss-of-function of PADI2 is associated with CCD, thereby providing a new target for the treatment of bone diseases.

**P135**

**Williams-Beuren Syndrome**

Muhammad Faiz Muhamad[1], Adesuwa Ero[2], McGowan Anne[2], Conor Woods[2]
[1]Robert Graves Institute- Tallaght University Hospital- Dublin- Ireland, Department of Endocrinology, Dublin- Ireland, Ireland ; [2]Robert Graves Institute- Tallaght University Hospital, Endocrinology and Diabetes, Dublin, Ireland

**Abstract Text**

Williams-Beuren Syndrome (WBS) is a rare multisystem genetic disorder, caused by the heterozygous mutation in the 7q11.23 region of chromosome 7. WBS is characterised by distinctive facies, mild intellectual disability, cardiovascular disease, and endocrine disorders including hypothyroidism, hypercalcaemia, and diabetes. Hypercalcaemia is seen in 5 to 50% of individuals with WBS. It is usually identified in infancy and resolves during childhood. The first case report of WBS with primary hyperparathyroidism (PHPT) was published in 2017. Our report describes a patient with PHPT, where the diagnosis of WBS was established on genetic testing undertaken as part of the workup for PHPT.

A 35-year-old gentleman has a history of mild intellectual disability, type 2 diabetes and osteopenia. He is investigated for persistently elevated serum calcium levels. The tests report an elevated parathyroid hormone level and a sestamibi scan is positive for a left inferior parathyroid adenoma. He undergoes a parathyroidectomy of the isolated gland and the histology is consistent with a parathyroid adenoma. He has been normocalcaemic one year postoperatively. He underwent genetic testing, which reported a heterozygous deletion of the long arm of chromosome 7, as seen in WBS. A review of his notes highlighted that WBS was suspected in infancy by a paediatrician and paediatric cardiologist, given his distinctive facies, hyperactivity, and presence of a cardiac murmur, but genetic testing was not performed at that time.

The mechanism of hypercalcaemia with WBS is not fully understood. Various mechanisms have been proposed, including the potential role of transient receptor potential C3 (TRPC3) channels in increasing calcium absorption. The American Academy of Paediatrics recommends that patients with WBS have 2-yearly calcium checks from age 6, and more frequently at younger ages. Hypercalcaemia in individuals with WBS should be investigated, to ensure that alternative aetiologies are diagnosed and managed appropriately.

**P136**

**Disentangling phenotypic heterogeneity via polygenic BMD assessment in general population individuals at risk of skeletal fragility**

Fjorda Koromani[1], Kirsty Huininga[1], Jeroen Van Rooij[1], Carola Zillikens[1], Kavousi Maryam[2], Arfan Ikram[2], Andre Uitterlinden[1], Fernando Rivadeneira[1]
[1]Erasmus MC, Internal Medicine, Rotterdam, Netherlands ; [2]Erasmus MC, Epidemiology, Rotterdam, Netherlands

**Abstract Text**

**Background**: Genetic discoveries from genome-wide association studies (GWAS) have empowered the application of polygenic risk scores (PRS) as a means to evaluate unconfounded evidence for a causal relation between risk factors and outcomes. Skeletal fragility often is the result of a complex process affected by systemic and local factors complicating the elucidation of etiological mechanisms suitable of intervention.

**Purpose**: To evaluate the impact of a highly heterogeneous etiological base of skeletal fragility on BMD polygenic enrichment; hypothesizing that risk factors not related to BMD will be "randomized" across the extremes of the BMD PRS distribution.

**Methods:** Observational cross-sectional population-based study in genotyped individuals (mean age 66.5 years;68.9% women) with deep phenotyping for risk factors, including BMD and osteoporotic fracture (OPFx). Out of 5331 individuals, seven independent subsets (n~750 each) were created, so that each subset would be enriched for a known bone fragility risk factor such as: 1)T2D, 2)low eGFR (<56.5), 3)high BMI (>31kg/m2), 4)falling history, 5)high FRAX MOF without BMD (>10%), 6)high cholesterol levels (>7.5 mmol/L) and 7)high TSH levels (>3.85 mU/L). BMD, OPFx, and presence of risk factor were compared between participants in the lower (n=666) and higher (n=666) 12.5% percentiles of the weighted BMD PRS (wPRS) distribution using t- or chi-squared tests.

**Results:** Participants in the lower wPRS BMD bin had significantly lower FN-BMD (0.83;SD=0.13 vs 0.92;SD=0.14;p-value<0.001), and higher OPFx prevalence (30.6% vs. 24%;p-value=0.008). Age, sex and all risk factors were equally distributed between the two groups (all p-values >0.3) (Table1).

**Conclusions:** Despite the highly heterogeneous base, individuals in the extremes of the BMD PRS distribution preserved significant differences in BMD and fracture risk, with other risk factors being "randomized". These findings constitute "proof-of-concept" for using Recall by genotype study designs, where an "unconfounded" contrast in BMD can be employed for deeper phenotyping or

**Table 1. Characteristics of the study population compared between low and high BMD wPRS**

| | low BMD wPRS n=666 | high BMD wPRS n=666 | P-value |
|---|---|---|---|
| wGRS BMD (SD) | 4.67 (0.13) | 5.72 (0.14) | <0.001 |
| FN-BMD (SD) | 0.83 (0.13) | 0.92 (0.14) | <0.001 |
| OPFx (%) | 204 (30.6) | 160 (24.0) | 0.008 |
| | | | |
| age (SD) | 66.6 (8.8) | 66.9 (9.2) | 0.4 |
| sex (%) | 466 (70.0) | 449 (67.4) | 0.3 |
| FRAX MOF >10% (%) | 173 (26.0) | 164 (24.6) | 0.6 |
| T2D (%) | 87 (13.1) | 99 (14.9) | 0.3 |
| TSH >3.85 mU/L (%) | 90 (13.5) | 104 (15.6) | 0.3 |
| eGFR <56.5 (%) | 90 (13.5) | 98 (14.7) | 0.5 |
| falling last month (%) | 101 (15.2) | 108 (16.2) | 0.6 |
| BMI >31 kg/m2 (%) | 92 (13.8) | 99 (14.9) | 0.6 |
| cholesterol >7.5 mmol/L (%) | 86 (12.9) | 98 (14.7) | 0.3 |

**Poster Sessions: Inflammation, arthritis and other joint diseases**

**P137**

**Innate immune response modulation by osteoblasts in Cutibacterium acnes related bone prothesis infections.**

*Léa Thoraval[1,2], Min Tang-Fichaux[1], Jennifer Varin-Simon[1], Christine Guillaume[1], Sophie C. Gangloff[1,2], Céline Mongaret[1,3], Fany Reffuveille[1,2], Frédéric Velard[1]*

[1]University of Reims Champagne-Ardenne, EA4691 BIOS "Biomatériaux et inflammation en site osseux", Reims, France ; [2]University of Reims Champagne-Ardenne, Microbiology division- UFR Pharmacy, Reims, France ; [3]University Hospital of Reims CHU Reims, Pharmacy division, Reims, France

**Abstract Text**

*Cutibacterium acnes* is a commensal skin bacterium, involved in bone prosthesis infections (BPI) [Boisrenoult, 2018] presenting low-grade clinical symptoms that delay the diagnosis [Portillo, 2013]. We hypothesized that *C. acnes* could escape immune system in bone site. This work aims to study the ability of *C acnes*-infected osteoblasts (iOB) to control innate immune cells response.

Human primary OB (n=4), cultured on titanium alloy, were infected with two *C. acnes* strains (a reference and a BPI-related strain) for 3h (MOI 20:1 and 200:1). After 24h, human primary neutrophils (PMN) (n=16) were put into contact with iOB for 4h. Pro-inflammatory mediators' production by OB and PMN, independently or during PMN/OB/*C. acnes* interaction, or by PMN challenged with iOB-conditioned supernatants, was determined by ELISA. Confocal fluorescence microscopy (CLSM) and scanning electron microscopy (SEM) allowed to visualize these complex interactions.

We demonstrated an increased secretion of IL-8, IL-6 and MCP-1 by iOB ($p$<0.05) only at MOI 200:1. Conditioned supernatants from iOB did not trigger PMN response, whereas PMN/iOB contact elicited a MOI-dependent production of IL-8, TNF-α and MIP-1β ($p$<0.05) by PMN. iOB-mediated IL-6 and MCP-1 production was noticed only after the triple interaction. Previous data evidenced a more sustained PMN inflammatory response and recruitment with BPI strain. Here, no differences were found between both strains. CLSM showed the presence of *C. acnes* in both OB and PMN, while SEM highlighted an absence of bacteria at the cell surface, suggesting an effective clearance by PMN, and a real ability of OB to hide *C. acnes* from PMN.

This work proposes that *C. acnes* could escape immune system by, at least, partial internalization within the OB. Even if precise mechanisms remain to be elucidated, these results show for the first time that OB may temper the immune response to *C. acnes* despite production of inflammatory mediators.

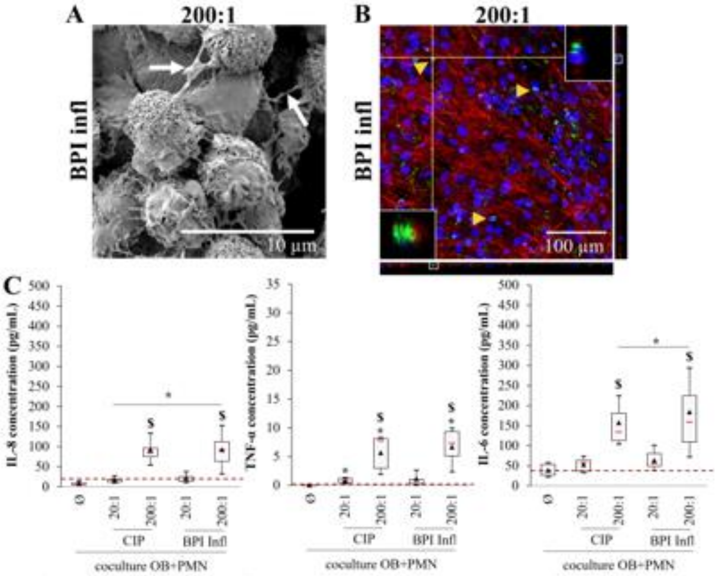

**A complex interaction between bone cells, immune cells and *C. acnes* leads to the production of pro-inflammatory mediators.** (**A**) Representative (n=4 OB independent donors) SEM images (magnification ×5000, yellow triangles indicate bacteria) and (**B**) CLSM images (DAPI-stained nuclei in blue, phalloidin-AlexaFluor®568-stained actin filaments in red, *C. acnes* immunostaining in green –AlexaFluor®488, yellow triangles indicate bacteria inside PMN) of primary OB (n=3 independent donors) infected with -BPI infl- *C. acnes* strain in interaction with PMN (n=6 independent donors).(**C**) Secretion of pro-inflammatory mediators by human primary OB and/or PMN induced by *C. acnes*. The dotted red line indicates the median production by OB or iOB over the same period without PNN. * $p$<0.05 *vs* control OB, **$** $p$<0.05 between MOI, n=8 independent PMN donors on 4 independent osteoblast donors.

**P138**

### Positioning of coronavirus infection in patients admitted with inflammatory rheumatic diseases.

*Jose Ramón Lamúa Riazuelo[1], Angela Herranz Varela[1], Blanca Varas de Dios[2]*
*[1]Hospital Universitario del Henares, Rheumatology, Madrid, Spain ; [2]Hospital Universitario Santa Cristina, Rheumatology, Madrid, Spain*

**Abstract Text**

**Background/Objectives:** To analyse the frequency of coronavirus infection in patients with inflammatory rheumatic diseases requiring hospital admission

**Methods:** Retrospective descriptive observational study of admissions of patients with inflammatory rheumatic diseases between January 2021 and August 2023 at the Hospital Universitario del Henares

**Results:** Between January 2021 and August 2023 there were 254 non-scheduled hospital admissions of patients with inflammatory rheumatic diseases.

Infectious causes account for 46.1%,of the admissions, being respiratory (67.5%), digestive (13.7%) and urinary (12%) the most frequent.

32 COVID-19 cases were confirmed, accounting for 12.6% of the total admissions. 19 were females (59.4%) versus 13 males (40.6%)

3 patients (9.4%) required intensive care unit support and there was 1(3.1%) exitus.

Coronavirus/COVID infection accounts for 40.5% of respiratory infections and 27.3% of overall admissions due to infectious causes, being more frequent in patients with Rheumatoid arthritis, psoriatic arthritis and ankylosing spondylitis.

As an example, respiratory infection of bacterial origin, which is the most frequent cause of hospital admission in patients with inflammatory rheumatic diseases, accounts for 53.2% of admissions for infectious respiratory reasons. Image 1

Analysing the concomitant biologic or synthetic-directed DMARDs received by the patients, only 8 patients received them (25%) (2 Adalimumab, 2 Tocilizumab, 2 Baricitinib, 1 Abatacept and 1 Infliximab), finding no significant relationship between these treatments and the frequency of admission due to coronavirus infection.

No admissions were recorded in patients on active treatment with Rituximab.

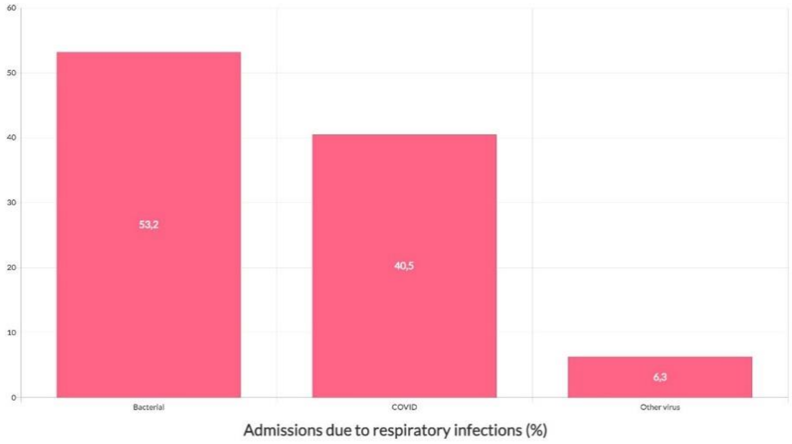

**Conclusion:** Coronavirus infection is a very frequent viral infection in hospital admissions of patients with inflammatory rheumatic diseases. We found no association between biologic or synthetic-directed DMARDs and the frequency of admission due to coronavirus infection.

**P139**

**Baseline Knee Pain Predicts Long-Term Response of Intra-Articular Steroid Injection in Symptomatic Knee Osteoarthritis: Data from OAI**

*Guangtao Fu*[1]
[1]*Guangdong Provincial Peoples' Hospital, Orthopedics, Guangzhou, China*

**Abstract Text**

**Objective:** The current study aims to investigate the factors that could predict response to intra-articular corticosteroid injection (IACI) in patients with knee osteoarthritis (KOA).

**Methods:** Data of participants were retrieved from the Osteoarthritis Initiative database. Participants with at least one IACI treatment on single or bilateral knees within the first 5 years of follow-up were retrospectively included. Demographic data and clinical and radiographic variables were collected at both baseline and the first follow-up after IACI treatment. Positive response to IACI treatment was defined as >20% reduction of Western Ontario and McMaster Universities Osteoarthritis index (WOMAC) pain score from V0 to V1. All the variables with $P < 0.2$ after the comparison between the response and non-response groups were included in a multivariable logistic regression model to identify independent response predictive patient-specific valuables. receiver operating characteristic curves were performed to establish the cutoff values of independent predictors.

**Results:** the current study included a total of 385 participants (473 knees), with 155 and 318 knees classified into the response group and non-response group, respectively. those with satisfied responses to IACI treatment had significantly higher WOMAC pain score ($P < 0.001$), disability score ($P = 0.002$), and stiffness score ($P = 0.015$) at the baseline. The baseline WOMAC pain score showed significant association with positive response to IACI treatment in multivariate logistic analysis and the best cutoff value was 5 points. the rate of analgesic utilization was lower ($P = 0.014$) in the response group than in the non-response group after the IACI treatment.

**Conclusion:** KOA patients with a baseline WOMAC pain score ≥5 are more likely to benefit from IACI treatment.

**P140**

**Phenotypes of body composition in women with systemic sclerosis**

*Arina Sorokina[1], Natalia Toroptsova[1], Olga Dobrovolskaya[1], Nikolay Demin[1]*
*[1]V.A. Nasonova Research Institute of Rheumatology, Department of Osteoporosis, Moscow, Russia C.I.S.*

**Abstract Text**

**Aim:** to assess body composition and risk factors of pathological phenotypes in women with systemic sclerosis (SSc).

**Methods:** Study involved 64 women (median age 54.0 [48.0;62.0] years) with SSc. Dual energy X-ray absorptiometry was performed to evaluate body composition and bone mineral density of lumbar spine, femoral neck (FN), total hip (TH). Osteoporosis (OP), sarcopenic phenotype (SP), and obesity phenotype were defined. Univariate linear regression was used to identify risk factors for each phenotype. The study was approved by the local ethics committee.

**Results:** 40 (62.5%) patients had limited and 24 (37.5%) - diffuse skin subtype of SSc. 30 (46.8%) patients had OP, 47 (73.4%) – obesity, 22 (34.4%) - SP, 4 (6.3%) - normal phenotype. 15 (23.4%) persons had isolated and 49 (76.6%) – combined phenoytpes. SP positively associated with BMI (β= 0.77, p<0,001), nutrition status by MNA-SF (β= 0.52, p<0,001), BMD FN (β= 0.52, p<0,001) and TH (β= 0.50, p<0,001) and negatively – with modified Rodnan score (β= -0.40, p=0,005), cumulative dose of glucocorticoids (GC) (β= -0.39, p<0,001). OP associated with hand joint contractures (β=1.56, p<0,001), disease duration (β=0.01, p<0,001), age (β=0.06, p<0,001), diffusing capacity of the lung for carbon monoxide (DLCO) (β=1.74, p<0,001), duration proton pump inhibitors (PPI) using (β=0.02, p<0,001), postmenopause (β=1.56, p<0,001), duration and cumulative dose of GC (β=0.12 and 0.06, p<0,001, respectively). No associations with obesity were found.

**Conclusion:** 93.7% of patients with SSc had pathological phenotypes.The most frequent phenotype was obesity, isolated or combined with OP or sarcopenia. BMI, nutrition status by MNA-SF, modified Rodnan score, cumulative dose of GC, and BMD of proximal femur were associated with SP in SSc patients. Age, postmenopause, hand joint contractures, duration of SSc, DLCO, PPI use, duration and cumulative dose of GC, lean mass were associated with OP. No association with the obesity was observed.

**P141**

**Uric acid and bone mineral density association in postmenopausal women with rheumatoid arthritis**

*Olga Dobrovolskaya[1], Nikolay Demin[1], Elena Samarkina[1], Natalia Toroptsova[1]*
*[1]V.A. Nasonova Research Institute of Rheumatology, Department of Osteoporosis, Moscow, Russia C.I.S.*

**Abstract Text**

**Aim:** to assess the frequency of hyperuricemia and its association with bone mineral density (BMD) in postmenopausal women with rheumatoid arthritis (RA).

**Methods:** 173 postmenopausal women with RA (aged 40-75 years) enrolled in the study. All patients underwent clinical and laboratory examination to determine the level of uric acid (UA) in the blood serum (spectrophotometry in fresh specimens; Cobas C311 analyzer). The level of MK>360 mmol/l was taken as hyperuricemia. Dual-energy X-ray absorptiometry (DXA, Lunar prodigy, GE, USA) was performed to measure the BMD of lumbar spine (LS), femoral neck (FN) and total hip (TH). The study was approved by the local ethics committee.

**Results:** Median age of patients with osteoporosis (OP) and patients without OP was 62 [57; 68] and 61 [55; 68] years, respectively (p>0.05). UA serum level in patients without OP was more than in persons with OP (278.3 [241.8; 329.1] µmol/l and 259.3 [198.9; 307.7] µmol/l, respectively; p<0.05). Hyperuricemia was identified in 4.5% patients with OP and in 17.9% women without OP (p<0.05). UA level correlated with LS (r=0.32, p<0.001), FN (r=0.22, p<0.05), TH (r=0.27, p<0.001) and duration of postmenopause (r=0.26, p<0.001). In logistic regression analysis the normal level of UA was a protective factor for OP in postmenopausal women with RA (OR 0.55 95%CI 0.33-0.90, p=0.017).

**Conclusion:** UA serum level in women without OP was higher than in patients with OP (p<0.05). Hyperuricemia was 3.9 times more often in persons without OP compared to those with OP. A high level of UA was a protective factor for low BMD and the presence of OP.

**P142**

**Machine learning to characterize bone biomarkers profile in rheumatoid arthritis**

*Giovanni Adami*[1], *Angelo Fassio*[1], *Camilla Benini*[1], *Maurizio Rossini*[1], *Ombretta Viapiana*[1], *Davide Gatti*[1]
[1]*University of Verona, Rheumatology Unit, Verona, Italy*

**Abstract Text**

**Background:** Bone metabolism is disrupted in rheumatoid arthritis (RA); however, the bone metabolic signature of RA is poorly known.

**Purpose:**
The objective of the study is to further characterize the bone metabolic profile of RA and compare it to psoriatic arthritis (PsA), systemic sclerosis (SSc) and healthy controls.

**Methods:** We did a cross-sectional case-control study on consecutively enrolled patients and age-matched controls. We collected clinical characteristics, serum biomarkers related to bone metabolism and Bone Mineral Density (BMD). A multiple correlation analysis using Spearman's rank correlation coefficient was conducted within the RA patient group to investigate associations between biomarker levels and clinical variables. Machine learning (ML) models and Principal Component Analysis (PCA) was performed to evaluate the ability of bone biomarker profiles to differentiate RA patients from controls.

**Results:** We found significantly lower BMD in RA patients compared to PsA, and Systemic Sclerosis SSc groups. RA patients exhibited higher Dkk1, sclerostin and lower P1nP and B-ALP levels compared to controls (Figure 1 panel A). No significant differences in CTX levels were noted. Correlation analysis revealed associations between bone biomarkers and clinical variables (Figure 1 panel B). PCA and ML highlighted distinct biomarker patterns in RA which can effectively discriminated bone biomarkers profile in RA from controls (Figure 1 panel C).

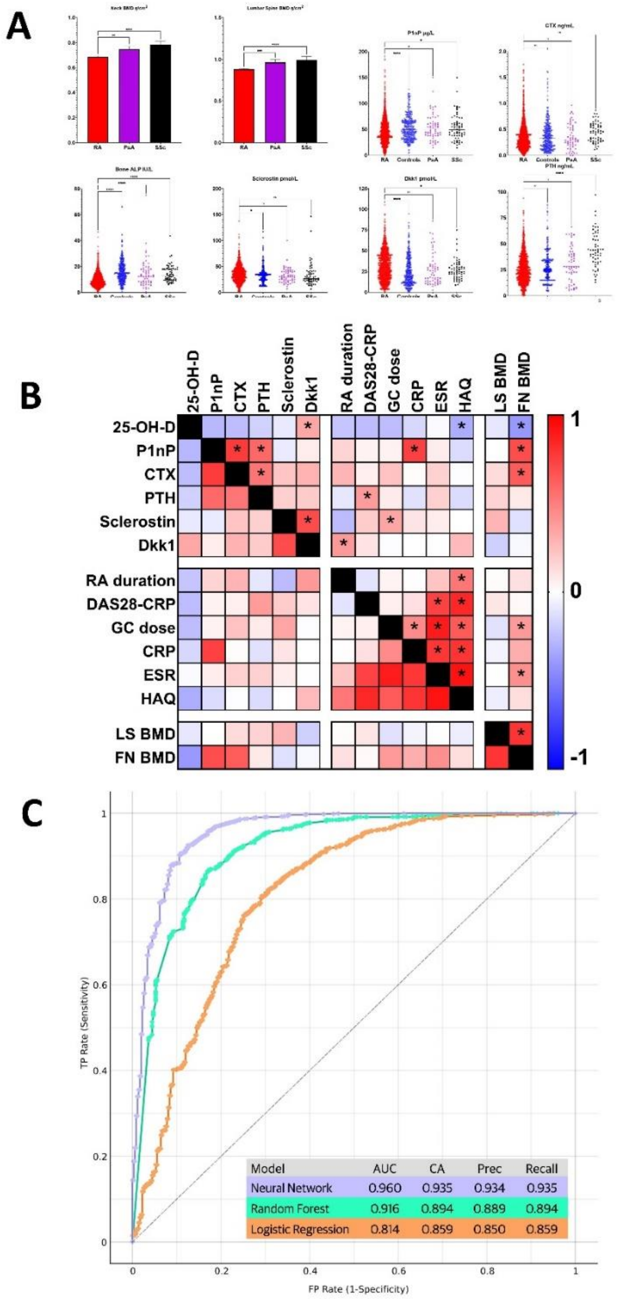

**Conclusion:** Our study helped uncover the distinct bone profile in RA, including changes in bone density and unique biomarker patterns. These findings enhance our comprehension of the intricate links between inflammation, bone dynamics, and RA activity, offering potential insights for diagnostic and therapeutic advancements in managing bone involvement in this challenging condition.

**P144**

**The Relationship Between Osteoarthritis Grading and the Risk of Subsequent Hip Fractures in Older Women: A Prospective Cohort Study**

*Wenshuang Zhang*[1], *Yufeng Ge*[2], *Yandong Liu*[3], *Fengyun Zhou*[3], *Yi Yuan*[3], *Xiaoguang Cheng*[3], *Ling Wang*[3]

[1]Beijing Jishuitan Hospital- Capital Medical University- Peking University Fourth School of Clinical Medicine, Department of Radiology, Beijing, China ; [2]Beijing Jishuitan Hospital- Capital Medical University, Department of Orthopaedics and Traumatology, Beijing, China ; [3]Beijing Jishuitan Hospital- Capital Medical University, Department of Radiology, Beijing, China

**Abstract Text**

**Background:** Hip fractures in older women impact public health, leading to increased morbidity and mortality. A critical health concern is the risk of a second hip fracture and prevalence of hip osteoarthritis (HOA), which may affect skeletal integrity and increase fracture risk.

**Purpose:** To explore the relationship between Kellgren-Lawrence (KL) grading of osteoarthritis (OA) and the risk of hip second fractures in older women.

**Methods:** Hip computed tomography (CT) and radiographic examinations were conducted post-first fracture. Areal bone mineral density (aBMD) at total hip (TH), femoral neck (FN), trochanter (TR), and intertrochanter (IT) of contralateral femur was measured using CT X-ray absorptiometry. HOA classification was assessed on baseline KL grades. HRs for the risk of second hip fractures were evaluated using Cox proportional hazards models. AUC with 95%CI was calculated using multivariate logistic regressions to assess predictive power of incorporating OA grading into models combining BMD parameters.

**Results:** The average age at baseline was 74.9(±9.5) years. Initial HOA Grade distribution was 108 in Grade0, 79 in Grade1, 16 in Grade2, and 3 in Grade3. Over 4.5 years, 35 participants experienced second hip fractures, 153 without refractures, and 18 deceased. In second fracture group, HOA grades were distributed as 14 in Grade0, 17 in Grade1, 3 in Grade2, and 1 in Grade3. Significant HRs for predicting second hip fractures were observed for TH aBMD (HR,1.79;*p*=.02) and IT aBMD (HR,2.04;*p*<.01). Except for the combined HOA Grades2 and 3 (HR,4.27;*p*=.04), no significant HRs were found for HOA classification. The AUC for models incorporating OA grading was higher than 77%, but the difference in AUC between models with and without OA grading was not statistically significant.

**Conclusion:** The presence of OA in the contralateral hip may increase the risk of hip second fractures in older women, although the exact statistical impact of incorporating OA grading into predictive models requires further investigation.

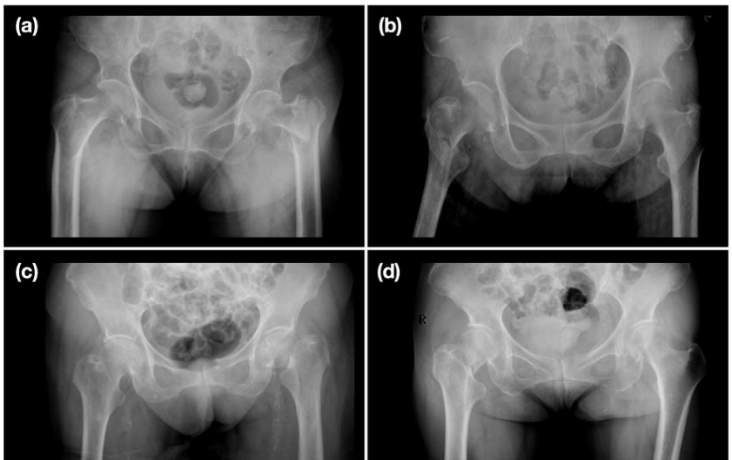

**Figure 1.** Radiologic illustrations depicting hip second fractures and osteoarthritis (OA) grades in elderly females:
(a) 83-year-old female with an initial left hip fracture, right hip OA grade 3, and a right hip second fracture.
(b) 80-year-old female with an initial right hip fracture, left hip OA grade 2, and a left hip second fracture.
(c) 87-year-old female with an initial left hip fracture, right hip OA grade 1, without a second fracture in the right hip.
(d) 77-year-old female with an initial right hip fracture, left hip OA grade 0, without a second fracture in the left hip.

**P145**

### Inhibiting lysyl oxidases (LOX(L)) prevents cartilage calcification in osteoarthritis

*Sonia Nasi[1], Ilaria Bernabei[1], Elodie Faure[2], Mario Romani[3], Julien Wegrzyn[4], Jürgen Brinckmann[5], Veronique Chobaz[2], Alexander So[2], Thomas Hugle[2], Nathalie Busso[2]*
*[1]CHUV, Musculoskeletal Medicine, Epalinges, Switzerland ; [2]CHUV, Musculoskeletal Medicine, Lausanne, Switzerland ; [3]CHUV, Medicine, Lausanne, Switzerland ; [4]CHUV, Orthopedic Surgery, Lausanne, Switzerland ; [5]University Lubeck, Dermatology, Lubeck, Germany*

### Abstract Text

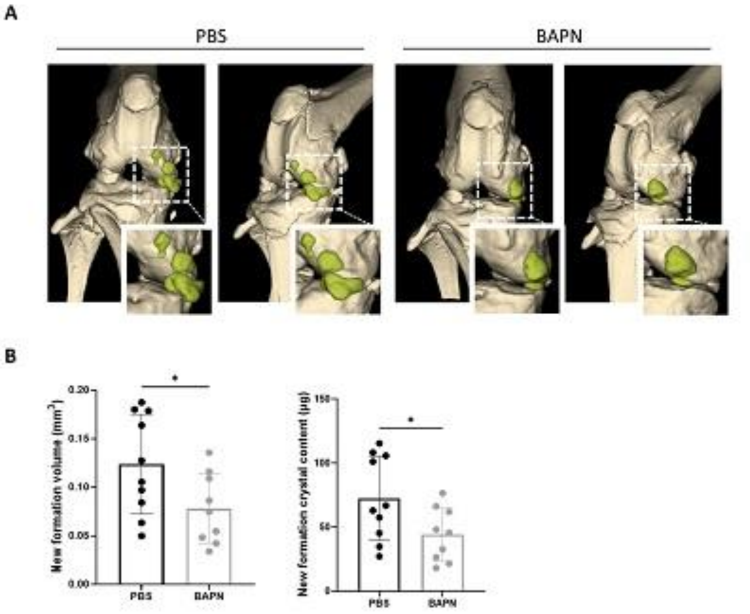

**Background:** Pathologic calcification of cartilage, characterized by the deposition of calcium-containing crystals, is a pivotal feature in osteoarthritis (OA). Lysyl oxidases (LOX(L)) are a group of enzymes catalyzing cross-link formation in collagen and elastin fibers. While their role in physiologic calcification of bone has been extensively elucidated, their involvement in pathologic cartilage calcification remains unexplored.

**Purpose:** To examine the involvement of LOX(L) in cartilage calcification, and the underlying mechanisms.

**Methods:** In vitro, murine and human chondrocytes were cultured in calcifying medium and treated with the pan-LOX(L) inhibitor β-aminoproprionitrile (BAPN). Calcification was evaluated by Alizarin red, gene expression by qRT-PCR, fibrosis and hypertrophy by immunohistochemistry, IL-6 secretion by ELISA, and ROS production by fluorometric assay. In vivo, meniscectomized mice were treated with either PBS or BAPN over two months post-surgery. Knee calcific deposits were analyzed by MicroCT scanner. To explore potential correlations between LOX(L) genes and calcification-related genes, we conducted a GeneBridge bioinformatics analysis.

**Results:** LOX(L) inhibition by BAPN diminished crystal deposition in chondrocytes ($p<0.0001$) and in meniscectomized mice ($p < 0.05$, see figure). Underlying mechanisms included classical and non-classical roles of LOX(L). First, diminished cross-links in extracellular matrix suppressed chondrocyte calcification via downregulation of Anx5, Enpp1, Pit1 and Pit2 genes ($p < 0.05$). Second, LOX(L)

inhibition downregulated fibrotic markers Col1 ($p<0.0001$) and Col3 ($p<0.0001$). Additionally, BAPN blocked chondrocytes hypertrophic differentiation (Runx2 $p<0.0001$ and COL10 $p < 0.05$), IL-6 release ($p < 0.05$) and ROS production ($p < 0.05$), all triggers of cartilage calcification. Finally, bioinformatics transcript analysis revealed that LOX(L)-containing module positively correlates with calcification, hypertrophy and catabolic enzymes modules. This association was conserved through species (mouse and human) and through different potentially calcifying tissues (kidney, arteries, skin).

**Conclusion:** LOX(L) provide a novel target for future therapies aimed at treating pathological cartilage calcification in osteoarthritis.

**P146**

**Body Composition and Bone Mineral Density in Postmenopausal Women with Advanced Knee Osteoarthritis Undergoing Surgical Treatment**

*Jung Yoon Park*[1]*, Hyoung Moo Park*[2]*, Kim Mee-Ran*[1]*, Song Jae-Yen*[1]*, Jung Yoon-Jee*[1]
*[1]Seoul St. Mary's Hospital- College of Medicine- The Catholic University of Korea- Seoul- Korea., Department of Obstetrics and Gynecology, Seoul, Korea- South ; [2]Grace Women's Hospital - Goyang- Korea., Department of Obstetrics and Gynecology- Menopause Clinic, Goyang, Korea- South*

**Abstract Text**

**Objectives:** This study aimed to demonstrate the bone mineral density (BMD) and body composition in postmenopausal women with knee osteoarthritis (OA) who underwent surgical treatment, such as total knee arthroplasty, osteotomy, or meniscectomy.

**Methods:** A total of 254 women with OA aged 50 years who underwent surgical treatment were enrolled in this study. We evaluated obesity-related factors, muscle components, and BMD using dual-energy X-ray absorptiometry.

**Results:** No significant differences were noted in the BMD of the hip joint between the symptomatic side of the leg with knee OA and the contralateral side. However, when comparing the BMD of each component, the results indicated a significantly higher BMD in the obesity group based on body mass index (BMI). When defining sarcopenic obesity (SO) using various indicators of obesity (BMI, the estimated visceral adipose tissue area, android/gynoid ratio, and total body fat percentage), the prevalence of SO in the OA group who underwent surgical treatment ranged from 22.0% to 49.6%.

**Conclusions:** This study investigated obesity-related factors in patients with advanced knee OA who underwent surgery, revealing a high prevalence of overweight/obese individuals, the presence of SO, and a complex relationship between obesity, body composition, and bone density, highlighting the potential protective effects of weight-bearing on bone health while exploring the impact of sarcopenia on bone density differences in the context of OA. Depending on various definitions of obesity, diverse proportions of SO in patients with OA have been observed, and further detailed research is required to understand its impact on the condition.

**P147**

**Exploring the Influence of Bone Marrow Adiposity on Osteoarthritis-Associated Bone Remodeling**

*<u>Natalia Zapata Linares</u>[1], Indira Toillon[1], Kristell Wanherdrick[1], Audrey Pigenet[1], Fanny Duhalde[1,2], Marie Binvignat[1], Patxi San Martin-Uriz[3], Clément Guibert[2], Loïc Louvet[4], Maria E. Calleja-Cervantes[3,5], Olfa Ghali[4], Geoffroy Nourissat[1], Damien Laterme[4], Odile Broux[4], Felipe Prosper[3,6,7,8], Christophe Chauveau[4], Francis Berenbaum[1], Jessem Landoulsi[2], Juan R. Rodriguez-Madoz[3,6,8], Marie-Hélène Lafage-Proust[9], Stéphanie Lucas[4], Xavier Houard[1]*
*[1]Sorbonne Université / INSERM, Centre de Recherche Saint-Antoine, Paris, France ; [2]Sorbonne Université / CNRS, Laboratoire de Réactivité de Surface LRS, Paris, France ; [3]Cima/Universidad de Navarra / IdiSNA, Hemato-Oncology Program, Pamplona, Spain ; [4]Université du Littoral Côte d'Opale, Marrow Adiposity and Bone Lab MABLab, Boulogne sur Mer, France ; [5]Cima/Universidad de Navarra / IdiSNA, Computational Biology Program, Pamplona, Spain ; [6]Universidad de Navarra, Cancer Center Universidad de Navarra CCUN, Pamplona, Spain ; [7]Clinica Universidad de Navarra / IdiSNA, Hematology and Cell Therapy Department, Pamplona, Spain ; [8]Instituto de Salud Carlos III, Centro de Investigación Biomédica en Red de Cáncer CIBERONC, Madrid, Spain ; [9]Université Jean Monnet, Sainbiose/Inserm, Saint-Étienne, France*

**Abstract Text**

**Background:** Osteoarthritis (OA), a prevalent age-related musculoskeletal disease lacking a definitive cure needs exploration of novel contributing factors. While bone marrow adipocytes (BMAds) have been implicated in age-related bone diseases, their role in OA remains unclear.

**Purpose:** To explore the function of BMAds in bone remodeling associated with OA.

**Methods:** This pioneer study employed C57BL/6 mice undergoing meniscectomy (MNX) or SHAM surgery, upon ethical approval, assessing OA development over 15 weeks through OARSI scores and quantifying perilipin+ adipocytes in epiphyseal and metaphyseal regions. Tibial Mesenchymal Stromal Cells (MSCs) isolated from MNX and SHAM mice were co-differentiated *in vitro*. Human BMAds (OA-BMAds) and OA-hMSCs were isolated from hip OA patients upon ethical approval. RNAseq assay evaluated gene expression patterns in OA-BMAds and differentiated human adipocytes (OA-Diff-hAdipo), while *in vitro* assays explored the impact of conditioned medium (CM) from OA epiphyseal bone on alkaline phosphatase (ALP) activity.

**Results:** OA progression was evidenced by cartilage lesions post-MNX ($p<0.001$), correlating positively with increased BMAd density ($p=0.0488$). MNX-MSCs and human OA-hMSCs exhibited compromised osteoblastic differentiation and mineralization. Notably, distinct transcriptomes were observed among OA-BMAds from different anatomical locations (epiphysis, metaphysis, and subcutaneous adipose tissue), implying different functions. Metaphyseal OA-BMAds were associated with hematopoietic stem cell differentiation, while epiphyseal OA-BMAds overexpressed genes linked to bone mineralization and remodeling. Functional assays demonstrated that CM from OA epiphyseal bone inhibited ALP activity, slowing calcium phosphate formation.

**Conclusion:** This study shows two key associations with OA development: increased bone marrow adiposity and impaired MSC differentiation. Moreover, distinct molecular profiles of epiphyseal and metaphyseal BMAds suggest varied roles in regulating the OA bone microenvironment. Specifically, molecules secreted by epiphyseal OA-BMAds delay mineralization, proposing novel therapeutic avenues. These findings suggest a relationship between BMAds and OA pathogenesis, providing insights for potential therapeutic interventions.

**P148**

**Physical activity and its impact on patients with rheumatic diseases**

Olga Seifert[1], Lev Lipovskiy[1], Benjamin Graul[1], Christoph Baerwald[1]
[1]University Hospital, Rheumatology Unit- Department of Internal Medicine, Leipzig, Germany

**Abstract Text**

**Background:** Rheumatic diseases such as systemic lupus erythematosus (SLE) or rheumatoid arthritis (RA) are associated with musculoskeletal complaints. Physical exercise can play a crucial role in reduction of comorbidities, optimizing both physical and mental health, and improving the quality of life.

**Purpose:** To characterize physical activity and its impact on patients with rheumatic diseases.

**Methods:** 285 outpatients (85 SLE and 200 RA) were included in this study. The following questionnaires were utilized: short-form health survey (SF - 36), the FACIT-Fatigue Scale and the Health Assessment Questionnaire (HAQ - DI). Physical activity was assessed for every patient using the long form of International Physical Activity Questionnaire (IPAQ - LF) and the Metabolic Equivalent of Task (MET) minutes per week.

**Results:** 10.6 % of SLE patients were physically inactive (525.2 ± 277.3 MET-Min/wk), 31.8 % had a moderate physical activity. Physical inactivity was associated with higher fatigue ($p < 0.04$) and lower «vitality» (SF-36, $p < 0.03$). In RA 14,4 % of patients did not met the WHO activity recommendation of at least 600 MET minutes per week, and another 34 % had moderate physical activity. Moderate intensity activities accounted for the largest proportion of total energy consumption (1,918 ± 1,447 MET-min/week). The following significant associations with lack of exercise can be identified: physical sum score (SF-36) ≤ 28, HAQ > 2.0, social functioning (SF-36) < 50, disease activity, sitting time/week > 67 h, age > 68 years.

**Conclusion:** The study indicates that not only somatic symptoms could decrease the levels of physical activity in SLE/RA patients. The study underlines the need for management strategies that specifically target physical activity as a part of an overall management program of rheumatic diseases.

**P149**

**The drug-loading nanoperfluorocarbon with enhanced cartilage repair and anti-inflammatory for osteoarthritis treatment triggered by radial extracorporeal shock wave**

Yingyu Zhang[1], Yajun Liu[2], Yingying Liu[1], Linlin Zhao[1]
[1]Shandong First Medical University & Shandong Academy of Medical Sciences, Medical Science and Technology Innovation Center, Jinan, China ; [2]Beijing Jishuitan Hospital, Beijing research Institute of Traumatology and Orthopedics, Beijing, China

**Abstract Text**

**Background** ： Osteoarthritis is characterized by the degradation of joint cartilage and internal homeostasis imbalance. This condition is further aggravated by tissue damage and a hypoxic microenvironment within the joint, which escalates inflammation. Radial Extracorporeal Shock Wave Therapy (rESWT) has been somewhat effective in treating bone disorders and aiding cartilage regeneration, yet it remains an isolated treatment modality. In recent developments, Nanomaterial-based Drug Delivery Systems (NDDS) have made strides in achieving targeted and controlled drug release, opening new avenues for osteoarthritis therapy.

**Objective** : Our research has synthesized a nanoperfluorocarbon that carries both drugs and oxygen, and developed a strategy for combining rESW with the nanoperfluorocarbon for treatment.

**Methods**: In vitro, the response capability of the nanoperfluorocarbon under different frequencies of rESW stimulation was assessed through changes in morphology and particle size, dissolved oxygen concentration, microbubble production, nanoparticle quantity and drug release experiments. In vivo animal studies, the osteoarthritis rat model was treated and then analyzed using Micro-CT, histopathological sections to examine the morphology and repair levels of the bone and joint tissues.

**Results**: Changes in morphology and particle size, dissolved oxygen concentration, microbubble production, nanoparticle quantity, and drug release experiments before and after rESW treatment demonstrated the nanoperfluorocarbons' effective response to rESW, and the optimal frequency for rESW treatment was identified. Results from Micro-CT and immunofluorescence staining indicated that the combined therapy could alleviate inflammation, cartilage destruction, and joint erosion in osteoarthritis rats.

**Conclusions**: This study developed a nanoperfluorocarbon capable of responding to rESW, efficiently carrying oxygen and drugs. It releases oxygen and medication under the assistance of rESW and exhibits good biocompatibility. The synergistic action of rESW and nanoperfluorocarbon can improve the hypoxic microenvironment in osteoarthritis and reduce inflammatory responses, enabling early intervention and treatment of joint injury diseases.

**P150**

**Influence of fat mass and sarcopenia on treatment response in rheumatoid arthritis**

*Ji-Won Kim*[1]*, Jun Sik Yoon*[2]*, Bo young Kim*[1]*, Hwajeong Lee*[1]*, Sung-Hoon Park*[1]*, Seong-Kyu Kim*[1]*, Jung-Yoon Choe*[1]

[1]*Daegu Catholic University School of Medicine, Division of Rheumatology- Department of Internal Medicine, Daegu, Korea- South ;* [2]*Inje University College of Medicine, Department of Internal Medicine, Busan, Korea- South*

**Abstract Text**

**Background/Introduction:** There are conflicting results regarding the association between body mass index and treatment response in patients with rheumatoid arthritis (RA). However, influence of body composition, including fat and muscle mass, on treatment response of RA is not fully understood. In addition, sarcopenia is more prevalent in RA patients compared with the general population, but the influence of sarcopenia on RA treatment response is unknown.

**Purpose:** We aimed to investigate the effect of body composition and sarcopenia on treatment response of RA patients.

**Methods:** Patients with active RA (defined by disease activity score in 28 joints ≥3.2) treated with biologic or nonbiologic disease-modifying antirheumatic drugs were prospectively enrolled in this study. Study participants underwent dual-energy X-ray absorptiometry (DXA) and bioelectrical impedance analysis (BIA) and were examined for hand grip strength at baseline. Treatment response at month 6 was evaluated by EULAR response criteria.

**Results:** A total of 121 patients had baseline and 6-month follow-up visits. Mean age was 57 years and 93% were women. Forty-five and 53 patients were treated with tumor necrosis factor inhibitors and janus kinase inhibitors, respectively. After 6 months of treatment, 75, 35, and 11 were good, moderate, and no responders, respectively. Area under the ROC curve (AUC) of high trunk-to-peripheral fat mass ratio by DXA was 0.720 (95% CI 0.604-0.837, p=0.016) for predicting no response. Combination of high trunk-to-peripheral fat mass ratio by DXA and low appendicular skeletal muscle mass by BIA improved diagnostic accuracy for no response at month 6 (AUC 0.747, 95% CI, 0.647-0.846, p=0.007). Hand grip strength was not associated with treatment response at month 6.

**Conclusions:** Combination of high truncal adiposity and low appendicular skeletal muscle mass can distinguish poor response after 6 months of treatment in RA patients. Hand grip strength has limited diagnostic accuracy in predicting RA treatment response.

**P151**

**Histological changes to the osteocyte peri-lacunocanalicular bone matrix that distinguish aseptic loosening from periprosthetic joint infection.**

*Dzenita Muratovic*[1], *Dongqing Yang*[1], *Lucian Bogdan Solomon*[1,2], *Gerald Atkins*[1]
*[1]The University of Adelaide, Centre for Orthopaedic and Trauma Research, Adelaide, Australia ; [2]Royal Adelaide Hospital and Central Adelaide Local Health, Department of Orthopaedics and Trauma Service, Adelaide, Australia*

**Abstract Text**

**Introduction:** Previous analysis of bone from patients with periprosthetic joint infection (PJI) revealed a widespread lack of intact or mature collagen, commonly surrounding osteocytes. This was attributed to increased expression of matrix-degrading enzymes by osteocytes and was independent of infective organisms. It has been proposed that aseptic loosening (AsL) may represent cases of subclinical infection. We examined possible means of distinguishing between these pathologies at the bone histological level.

**Methods:** Bone tissue samples were collected from confirmed PJI cases (n=17) and compared with bone samples collected from patients with AsL with no clinical signs of infection (n=17) and with control bone (Ctrl) taken from patients undergoing primary hip arthroplasty (n=18). Samples were histologically processed and subjected to Masson’s trichrome and new RGB stain specifically optimised and adapted for bone tissue.

**Results:** PJI bone is characterised by increased interlamellar collagen but decreased non-mineralised osteoid compared to both AsL (P=0.0001 and Ctrl bone (p<0.0001). The size of the lacune was not significantly different between groups, while PJI bone had a rounder lacune compared to both AsL (p=0.0004 and Ctrl bone (p<0.0001).

AsL bone is characterised by a distinctive ‘mottled’ appearance, with circular clearings in the matrix often aligned along canaliculi. Overall, AsL bone showed many more prominent canaliculi compared to Ctrl bone (p=0.02), while PJI bone had a relative deficit of canaliculi compared to both AsL p<0.0001 and Ctrl bone (p=0.04). See Figure 1.

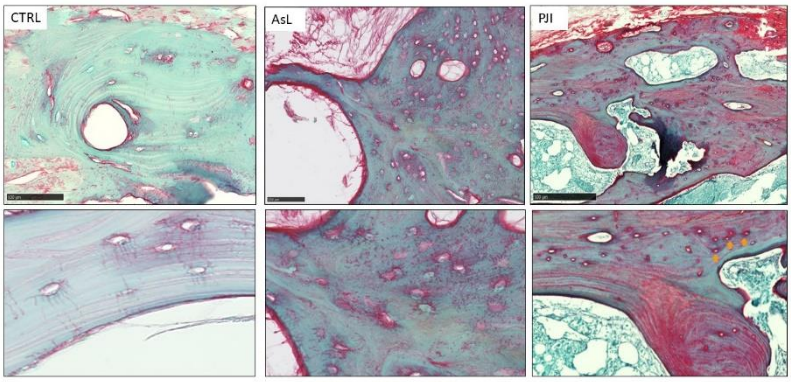

PJI bone tissue is characterised by significantly more degraded collagen (red colour), the presence of 'collars' degraded collagen around osteocyte lacunae (indicated by orange arrows) and a deficit of canaliculi.
AsL bone tissue displays a distinctive 'mottled' appearance due to abundant prominent canaliculi.

**Conclusions:** AsL bone, in all cases, displayed characteristic prominent canaliculi with associated nodules, likely due to increased peri-canalicular osteolysis/remodelling. This was distinct from PJI bone, where more widespread matrix degradation was evident with a relative lack of canalicular structures emanating from osteocyte lacunae. Histological examination of bone collagen composition and osteocyte lacuno-canalicular morphology may provide a valuable means of distinguishing cases of AsL loosening from PJI.

**ND-P018**

**Efficacy and safety of Upadactitinib on real world practise. A multicenter study**

*Blanca Varas de Dios*[1]*, José Ramón Lamúa Riazuelo*[2]*, María Laiño Piñeiro*[3]*, Isabel Pastora Granados Bautista*[4]*, Patricia Castro Pérez*[5]*, Irene Patricia Amil Casas*[6]
[1]*Santa Cristina Universitary Hospital, Rheumatology, madrid, Spain ;* [2]*Henares Hospital, Rheumatology, Madrid, Spain ;* [3]*Hospital Universitario de Navarra, Rheumatology, Pamplona, Spain ;* [4]*Hospital General Universitario de Toledo, Rheumatology, Toledo, Spain ;* [5]*Hospital Getafe, Rheumatology, Madrid, Spain ;* [6]*Clínica Cemtro, Rheumatology, Madrid, Spain*

**Abstract Text**

**Introduction:** Janus kinase inhibitors(JAKi) belong to a class of targeted, synthetic disease-modifying drugs that have recently expanded the therapeutic arsenal for some immune-mediated diseases.We are aware of the efficacy and safety data provided by clinical trials,but it is necessary to have safety and effectiveness data in real clinical practice, which is limited to date.

**Background**: To evaluate the efficacy and safety of Upadacitinib (UPA)in real clinical practice in patients with RA and to analyze its persistence.

**Methods**: Retrospective,observational,multicenter, real-life study.Patient sociodemographic and baseline variables related to the disease, time elapsed since RA diagnosis, activity indices and laboratory tests,concomitant and previous treatments with conventional,biological and targeted synthetic DMARDs were collected.During the nine-month follow-up,adverse events(AEs)and reasons for discontinuation were recorded.

**Results**: 60 patients have been recruited in 5national hospitals, diagnosed with RA according to the 2010 ACR/EULAR criteria.The baseline characteristics of the patients are shown in Table 1.Only 2patients(3.3%)received UPA in the first line of treatment and 38%in 4th line or more, with the average per patient being 3.2bDMARD or other SD prior to UPA.

Improvement is seen in the disease activity index and all patient-related outcomes since the start of treatment.No statistical significance was found between disease activity and demographic variables.Smoker patients had significantly higher DAS28values(p=0.029)at the third month of treatment.

22 AEs were reported in 17patients(28.3%).The most frequently were infections(8 respiratory,7 Covid,2 gastrointestinal),but they didn´t lead to treatment interruption in any case.No statistical significance was found between the use of concomitant treatments and AEs.

Treatment interruption was observed in7patients(11.67%)due to ineffectiveness(4),herpes zoster(HZ)(1),dyslipidemia(1),alopecia(1)and personal decision(1),which represents a persistence of Upadacitinib in88.33% of patients.Although only24(40%) patients were correctly vaccinated against HZ before the start of treatment,only1case of HZ was detected in the first 9months of follow.

**Conclusions:** Despite the limitations of retrospective observational studies,these real-life data support the efficacy and safety profile of Upadacitinib after 9months of treatment,with a high persistence rate.

**Poster Sessions: Mammalian and non-mammalian models, including preclinical imaging**

**P152**

**A novel in vitro model for peri-implant infections with Staphylococcus epidermidis**

*Judith Luisa Kokot[1], Samira Weisselberg[2], Jan Sevecke[1], Christine Dowling[1], Ruben Augustin[1], Anke Baranowsky[1], Holger Rohde[2], Johannes Keller[1]*
*[1]UKE Hamburg, Trauma and Orthopedic Surgery, Hamburg, Germany ; [2]UKE Hamburg, Medical Microbiology- Virology and Hygiene, Hamburg, Germany*

**Abstract Text**

**Background:** Staphylococcal infections have a high disease burden worldwide in trauma and orthopedic surgery. Although surgical techniques and implant design have improved significantly over the past decades, implant-associated infections are still associated with high morbidity. In particular, biofilm formation as a resistance mechanism of *Staphylococcus epidermidis* (*S. epidermidis*) leads to immune evasion and failure of antibiotic treatment and thus requires novel strategies to identify targets for the treatment of peri-implant infections.

**Objectives:** The purpose of this study was to establish a novel in vitro model of peri-implant infection with *S. epidermidis* to better understand its interaction with osteoblasts and macrophages during infection.

**Methods:** Mouse bone marrow-derived osteoblasts and macrophages were exposed to glass coverslips coated with biofilm-positive and biofilm-negative *S. epidermidis* strains in a defined 3D in vitro system. Infiltration of the biofilm by cultured cells was evaluated by confocal microscopy. Osteoblast and macrophage differentiation and function were assessed by alizarin red staining and gene expression analysis (qRT-PCR).

**Results:** Bacterial biofilm was rapidly infiltrated by macrophages, whereas osteoblasts showed a significantly reduced capacity for biofilm infiltration. Matrix mineralization by osteoblasts was similarly impaired in both biofilm-positive and biofilm-negative *S. epidermidis*. This observation was associated with decreased expression of osteoblast markers after long-term, but not short-term, infection with both *S. epidermidis* strains. Induction of pro- and anti-inflammatory cytokines in macrophages was primarily dependent on incubation time and the presence or absence of biofilm.

**Conclusions:** This in vitro model provides a reproducible way to test different infection scenarios in terms of duration and bacterial strain in osteoblasts and macrophages. We propose that this model could help to further characterize the molecular crosstalk of osteoblasts and macrophages with *S. epidermidis*.

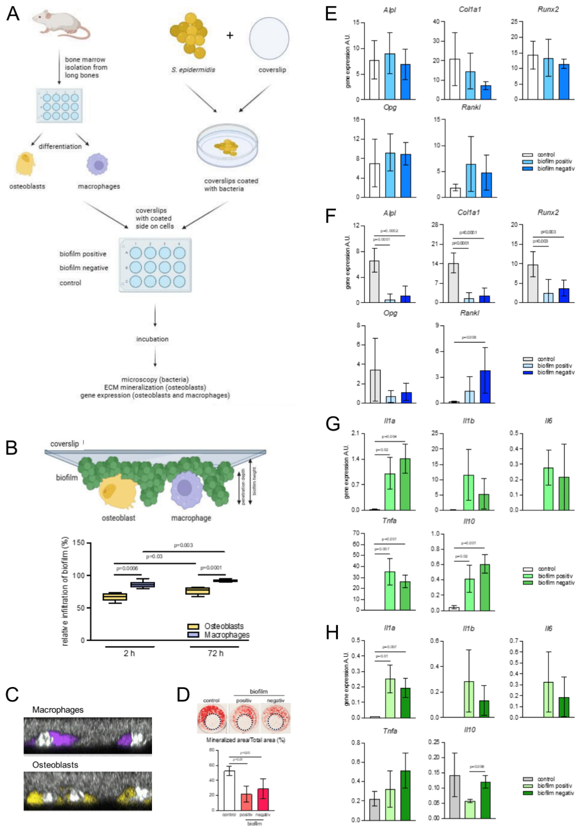

**Figure 1.** A novel in vitro model for peri-implant infections with *S. epidermidis*. **A)** Schematic representation of the workflow of the in vitro model. **B)** Measurement of biofilm infiltration by osteoblasts and macrophages in relation to biofilm height after 2h or 72h of infection at day 7 of respective cell differentiation. **C)** Representative images of biofilm infiltration by confocal microscopy after 2h of infection. **D)** Representative alizarin red stainings of osteoblast matrix mineralization after 72h of exposure to *S. epidermidis*. The dotted black circles indicate the former position of the *S. epidermidis*-coated coverslips. **E)** Gene expression of the indicated genes in osteoblasts with short-term exposure (2h) to *S. epidermidis*. *Alpl*=alkaline phosphatase; *Col1a1*=collagen type 1 alpha 1; *Runx2*=runt-related transcription factor-2; *Opg*=osteoprotegerin; *Rankl*= receptor activator of NF-κB ligand. **F)** Expression of the same genes in osteoblasts with long-term exposure (72h) to *S. epidermidis* strains. **G)** Gene expression of the indicated cytokines in macrophages with short-term exposure (2h) to *S. epidermidis*. *Il*=interleukin; Tnfa=tumor necrosis factor alpha. **H)** Expression of the same genes in macrophages with long-term exposure (72h) to *S. epidermidis*. One- or two-way ANOVA followed by Tukey's post-hoc-test were performed for data analysis. All experiments were approved by the local animal care committee.

### P153

### Exploring the role of transferrin receptor 1 in bone homeostasis.

*Vanessa Passin*[1], *Maria Ledesma-Colunga*[1], *Lorenz Christian Hofbauer*[1], *Martina Rauner*[1]
[1]*Medical Faculty and University Hospital Carl Gustav Carus- Dresden University of Technology, Department of Medicine III & Center for Healthy Aging, Dresden, Germany*

### Abstract Text

**Introduction:** Iron is an essential element for the function of all cells as it participates in the regulation of various cellular processes. Iron is available in the circulation bound to transferrin to keep it in a redox-inert state and facilitate its delivery to tissues. In most cells, uptake of iron-loaded transferrin by the transferrin receptor 1 (Tfr1) is a primary mechanism of iron supply. Yet, the mechanisms of iron acquisition in bone cells remains largely unknown.

**Purpose:** Purpose of this study was to investigate the significance of Tfr1, which mediates the uptake of transferrin-bound iron, for the function of osteoblasts and osteoclasts.

**Methods:** Twelve-week-old male and female Tfr1$^{fl/fl}$;Osx:Cre+ (doxycycline-repressible) and Tfr1$^{fl/fl}$;LysM:Cre+ conditional knockout mice and their littermate controls were used to asses bone microarchitecture as well as systemic bone turnover markers PINP and TRAcP5b (microCT, histomorphometry, ELISA). Bone marrow-derived cells from these mice were cultured *in vitro* to assess cellular iron status, differentiation, and function (qPCR, Alizarin Red S, TRAP staining, western blot).

**Results:** Tfr1$^{fl/fl}$;Osx:Cre+ males showed an increased L4 BV/TV [1.2-fold; $p<0.01$] with a decreased bone formation rate [2.2-fold; $p<0.05$] and lower levels of serum bone turnover markers [PINP: 1.3-fold; $p<0.01$, TRAcP5b: 1.5-fold; $p<0.01$]. Similarly, females displayed a higher femoral BV/TV [1.5-fold; $p<0.01$]. Tfr1-deficient osteoblasts showed decreased ferritin protein levels [1.3-fold; $p<0.05$] but no changes in mRNA levels of *Alpl*, *Col1a1* or *Bglap* nor in their mineralization capacity. Conversely, Tfr1 deficiency in osteoclast precursors did not alter the bone phenotype in mice nor the iron status and differentiation of osteoclasts *ex vivo*.

**Conclusion:** In summary, Tfr1 might be important for iron uptake in osteoblasts ensuring proper bone turnover, with the deletion of Tfr1 in osteoclasts showing no impact on bone microarchitecture. These results emphasize diverse iron acquisition strategies employed by various cell types to maintain cellular iron homeostasis.

**Poster Sessions: Muscle, physical activity and bone**

**P155**

**The effect and mechanism of small extracellular vesicles from exercise skeletal muscle in alleviating osteoporosis.**

*Minghui Zheng*[1], *Xiao Lin*[2], *Sukang Shan*[1], *Limin Lei*[1], *Feng Xu*[1], *Lingqing Yuan*[1]
[1]*the Second Xiangya Hospital- Central South University, Department of Metabolism and Endocrinology- National Clinical Research Center for Metabolic Diseases, changsha, China ;* [2]*the Second Xiangya Hospital- Central South University, Department of Radiology, changsha, China*

**Abstract Text**

Endurance exercise is an important lifestyle to improve the body's metabolism, delay aging, reduce inflammation, promote bone health, and maintain homeostasis. Muscles undergo drastic metabolic changes during exercise, which can secrete muscle factors, and reduce bone loss. During exercise, a large number of small extracellular vesicles (sEVs) are released into the circulation, mediating inter-organ crosstalk during exercise. This research aims to investigate the regulatory role of muscle-derived sEVs on bone homeostasis during endurance exercise and its mechanism. Previous studies have found that the exosome inhibitor GW4869 could partially block the protective effect of exercise on bone loss. Compared with control sEVs (Sed-sEVs), muscle-derived sEVs in exercise mice (Exe-sEVs) could promote the osteogenic differentiation of bone marrow mesenchymal stem cells (BMSCs), alleviate bone loss in ovariectomy (OVX) mice. In addition, the proteomics results showed that the expression of Sirtuin 3 (SIRT3) protein in Exe-sEVs was significantly higher than that in Sed-sEVs. Based on the above results, we hypothesize that exercise promotes the secretion of SIRT3-enriched sEVs by muscles, which are taken up by bones through circulation, thereby increasing bone mass. From a new perspective of musculoskeletal, this research will reveal the deep mechanism of inter-organ crosstalk mediated by exercise muscle-derived sEVs, and provide a new way for the prevention and treatment of osteoporosis.

**P156**

**Association between sarcopenic phenotype of body composition and nutritional status in women with rheumatoid arthritis**

*Olga Dobrovolskaya[1], Oxana Nikitinskaya[1], Nikolay Demin[1], Natalia Toroptsova[1]*
*[1]V.A. Nasonova Research Institute of Rheumatology, Department of Osteoporosis, Moscow, Russia C.I.S.*

**Abstract Text**

Patients with rheumatoid arthritis (RA) often have secondary sarcopenia due to a chronic inflammatory process. In addition, these patients may suffer from gastrointestinal pathology, the causes of which include both the disease itself and medications that lead to malnutrition.
**Aim:** To evaluate association between sarcopenic phenotype of body composition and nutritional status in women with RA.

**Material and methods:** 104 women (mean age 59,5±8,7 years), with RA (ACR/EULAR criteria, 2010). All patients underwent dual-energy X-ray absorptiometry and nutritional status assessment using Mini Nutricial Assessment (MNA). The sarcopenic phenotype was diagnosed with an appendicular muscle mass (AMM) of less than 15 kg or an appendicular muscle index of less than 5.5 kg/m$^2$. Patients with malnutrition and risk of malnutrition according to the MNA formed a group with a low nutritional status. The study was approved by the local ethics committee.

**Results**: 39 (37.5%) women had sarcopenic phenotype and 52 (50.0%) persons had low nutritional status. The patients with low nutritional status had a higher risk of osteoporotic hip fracture according to FRAX (p<0.05), lower AMM (p<0.05), and lower self-assessment of health status according to EQ-5D (p<0.05) compared with women with normal nutritional status. Persons with a sarcopenic phenotype significantly differed in nutritional status according to MNA, daily calcium intake, mid-upper arm circumferences and creatinine clearance compared with women with normal muscle mass. Multivariate regression analysis revealed that the sarcopenic phenotype was related to the nutritional status less than 24 points (odds ratio (OR) 4.13, p<0.05), daily calcium intake less than 500 mg (OR 4.62, p<0.05), and mid-upper arm circumferences less than 25 cm (OR 5.23, p<0.01).

**Conclusion:** Low nutritional status was found in a half of the examined RA women. The sarcopenic phenotype was associated with low nutritional status according to MNA, low calcium intake, and mid-upper arm circumferences less than 25 cm.

**P157**

**Dynamic changes of muscle immune microenvironment during exercise promoting muscle repair**

*Quanzhong Ren[1], Renxian Wang[1], Lining Xu[1], Linlin Fan[1], Dingding Wang[1], Yajun Liu[1]*
*[1]Beijing Research Institute of Traumatology and Orthopaedics- Beijing Jishuitan Hospital- Capital Medical University, JST sarcopenia Research Centre, Beijing, China*

**Abstract Text**

**Background:** Muscle regeneration is influenced by the coordinated response of immune cells, muscle stem cells, and fibro-adipogenic progenitor cells (FAPs). Severe trauma or aging interference can result in sustained inflammatory response, leading to muscle fiber necrosis and intermuscular fat infiltration. Exercise has an impact on the immune system. However, it is currently unclear whether exercise can prevent fat infiltration by modulating immune homeostasis.

**Objective:** This study aimed to investigate the effect of exercise on muscle immune cells during the process of muscle fat infiltration and the mechanisms underlying exercise-induced changes in immune cells.

**Methods:** We conducted experiments using a muscle glycerol injury model and a swimming exercise model. The therapeutic effect of exercise was verified through hematoxylin-eosin (H&E) staining and oil red O staining. Changes in immune cell numbers were observed using flow cytometry.

**Results:** The experimental results showed that exercise could reduce the body weight of mice and restore muscle strength to normal levels. H&E and oil red O staining results demonstrated that exercise could decrease muscle fat infiltration. Blood routine examination results indicated that exercise could suppress the inflammatory response. Flow cytometry results showed that exercise could increase the number of IL-33$^{+}$FAP cells, thereby recruiting Treg cells to migrate to the muscle site and promoting the transition of the immune environment.

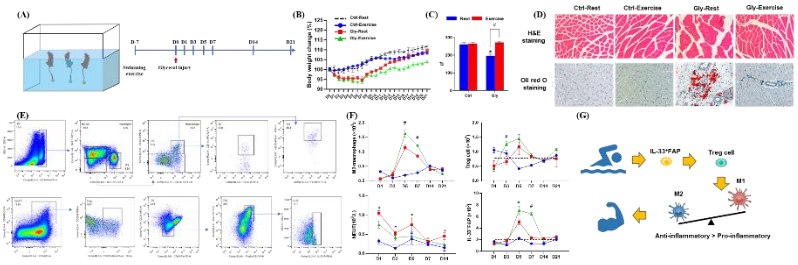

**Conclusion:** The findings of this study suggest that exercise can alleviate muscle fat infiltration and promote muscle repair. Exercise exerts its therapeutic effects by modulating changes in muscle immune cells, recruiting Treg cells, and facilitating the transition of the immune environment from pro-inflammatory to anti-inflammatory. These findings are of significant importance in the search for effective treatment methods for muscle dystrophy.

**P158**

**Exercise inhibits fat infiltration and promotes muscle repair by regulating muscle immune microenvironment**

*Quanzhong Ren[1], Lining Xu[1], Dingding Wang[1], Linlin Fan[1], Anyi Guo[1], Hui Qi[1], Renxian Wang[1], Yajun Liu[1]*

[1]*Beijing Research Institute of Traumatology and Orthopaedics- Beijing Jishuitan Hospital- Capital Medical University, JST sarcopenia Research Centre, Beijing, China*

**Abstract Text**

**Background:** Muscle regeneration is influenced by the coordinated response of immune cells, muscle stem cells, and fibro-adipogenic progenitor cells (FAPs). Severe trauma or aging interference can result in sustained inflammatory response, leading to muscle fiber necrosis and intermuscular fat infiltration. Exercise has an impact on the immune system. However, it is currently unclear whether exercise can prevent fat infiltration by modulating immune homeostasis.

**Objective:** This study aimed to investigate the effect of exercise on muscle immune cells during the process of muscle fat infiltration and the mechanisms underlying exercise-induced changes in immune cells.

**Methods:** We conducted experiments using a muscle glycerol injury model and a swimming exercise model. The therapeutic effect of exercise was verified through hematoxylin-eosin (H&E) staining and oil red O staining. Changes in immune cell numbers were observed using flow cytometry.

**Results:** The experimental results showed that exercise could reduce the body weight of mice and restore muscle strength to normal levels. H&E and oil red O staining results demonstrated that exercise could decrease muscle fat infiltration. Blood routine examination results indicated that exercise could suppress the inflammatory response. Flow cytometry results showed that exercise could increase the number of IL-33$^+$FAP cells, thereby recruiting Treg cells to migrate to the muscle site and promoting the transition of the immune environment.

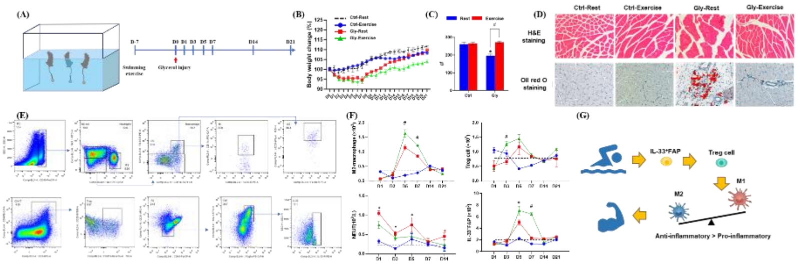

**Conclusion:** The findings of this study suggest that exercise can alleviate muscle fat infiltration and promote muscle repair. Exercise exerts its therapeutic effects by modulating changes in muscle immune cells, recruiting Treg cells, and facilitating the transition of the immune environment from pro-inflammatory to anti-inflammatory. These findings are of significant importance in the search for effective treatment methods for muscle dystrophy.

**P159**

**Physical activity and sarcopenic phenotype of body composition in postmenopausal women with rheumatoid arthritis**

*Olga Dobrovolskaya[1], Alexey Feklistov[1], Nikolay Demin[1], Natalia Toroptsova[1]*
[1]*V.A. Nasonova Research Institute of Rheumatology, Department of Osteoporosis, Moscow, Russia C.I.S.*

**Abstract Text**

Sarcopenia and osteoporosis are associated with insufficient physical activity in healthy people. Aim: to evaluate the interaction of the sarcopenic phenotype of body composition with physical activity (PhA) in postmenopausal women with rheumatoid arthritis (RA).

**Methods:** 117 postmenopausal women (mean age 59.4 ± 8,8 years) with RA underwent clinical and laboratory examination, dual-energy X-ray absorptiometry of whole body. Sarcopenic phenotype was diagnosed if appendicular muscle mass index was less than 5.5 kg/m2. The level of physical activity (PhA) was assessed using the International Physical Activity Questionnaire (IPAQ) and physical performance tests (Short Physical Performance Battery, SPPB). The study was approved by the local ethics committee.

**Results:** Sarcopenic phenotype was diagnosed in 36 (30.8%) of patients. Moderate and low PhA levels were detected in 53 (45.3%) and 8 (6.8%) women, respectively, among them 19 (31.1%) patients had sarcopenic phenotype and 42 (68.9%) - without it. In multivariate logistic regression analysis, independent factors associated with the sarcopenic phenotype were determined: the frequency of vigorous PhA less than 3 times a week (OR 4.67 (95%CI 1.06-15.47), p=0.014), walking less than 1 hour a day (OR 3.75 (95%CI 1.08-12.47), p=0.037), body mass index (BMI) less than 25 kg/m2 (OR 5.22 (95%CI 1.68-23.45), p=0.012).

**Conclusion:** 52.1% of patients with RA had moderate or low PhA level. The risk of sarcopenic phenotype increased with a low frequency of vigorous PhA, walking time less 1 hour a day, BMI <25 kg/m2.

**P160**

**Follistatin-compound hFSTΔHBS-FC improves bone and muscle mass during immobilization and ageing and adds to bone effect of bisphosphonates**

*<u>Bram Van der Eerden</u>[1], Andreas Lodberg[2], Mikkel Brent[2], Marijke Koedam[1], Renata Brandt[3], Jeroen Essers[4], Jesper Skovhus Thomsen[2], Ingrid Van der Pluijm[5], Annemarie Bruel[2], Johannes Van Leeuwen[1]*
*[1]Erasmus University Medical Center, Internal Medicine, Rotterdam, Netherlands ; [2]Aarhus University, Biomedicine, Aarhus, Denmark ; [3]Erasmus University Medical Center, Molecular Genetics, Rotterdam, Netherlands ; [4]Erasmus University Medical Center, Molecular Imaging, Rotterdam, Netherlands ; [5]Erasmus University Medical Center, Surgery, Rotterdam, Netherlands*

**Abstract Text**

**Background:** Sarcopenia and osteoporosis are natural consequences of aging and often related. Sarcopenia is associated with increased fracture risk, while immobilization leads to loss of muscle and bone mass. We developed a follistatin (FST)-based compound (hFST$_{\Delta HBS}$-FC) that can bind activin A and myostatin and prevents OVX-induced bone loss.

**Purpose:** We examined the impact of hFST$_{\Delta HBS}$-FC on bone and muscle mass in a premature aging mouse model (Ercc1$^{\Delta/-}$) and a botox-induced immobilization mouse model. We also examined the combined treatment of hFST$_{\Delta HBS}$-FC and zoledronate (ZOL).

**Methods:** Female mice (Botox model 16 weeks old; Ercc1$^{\Delta/-}$ model, 8 weeks old) were treated with vehicle, 10 mg/kg hFST$_{\Delta HBS}$-FC twice a week intraperitoneally, a single subcutaneous injection of 100 µg/kg zoledronate once, or a combination of both treatments. After 3 weeks, animals were sacrificed, and tissues were harvested.

**Results:** Botox-treatment led to a decrease in both bone mass (BV/TV(%): 11.1>6.7%) and muscle mass (quadriceps: -17%). $hFST_{\Delta HBS}$-FC and ZOL both increased BV/TV (6.7>8.6% and 6.7>16.9%, respectively), which was further elevated by combination treatment of $hFST_{\Delta HBS}$-FC with ZOL (6.7>20.3%). Similar effects were observed for other bone parameters like Tb.N and Tb.Sp. $hFST_{\Delta HBS}$-FC prevented weight loss and loss of muscle mass caused by systemic stress of immobilization. Bone and muscle mass were both reduced in $Ercc1^{\Delta/-}$ versus wildtype mice (*PMID:27556946).* ZOL prevented the decrease in bone mass and strongly enhanced by co-treatment with $hFST_{\Delta HBS}$-FC (*see Table*). $hFST_{\Delta HBS}$-FC also increased muscle mass (+20%) as well as total body weight (+7%) in $Ercc1^{\Delta/-}$ mice, which was not observed for ZOL.

**Conclusion:** $hFST_{\Delta HBS}$-FC improves bone mass and bone structure in addition to zoledronate but also stimulates muscle mass and prevents weight loss in conditions reflecting immobilization and aging. These results hold promise for a $hFST_{\Delta HBS}$-FC combination therapy with bisphosphonates against bone loss with the additional benefit of a muscle anabolic effect.

| $Ercc1^{\Delta/-}$mice | Control | $hFST_{\Delta HBS}$-FC | ZOL | $hFST_{\Delta HBS}$-FC + ZOL |
|---|---|---|---|---|
| BV/TV (%) | 4.7+/- 0.57 | 7.1+/-0.82 | 16.6+/-1.0 | 24.73+/-3.6 |
| Tb.N ($mm^{-1}$) | 1.1+/-0.1 | 1.6+/-0.15 | 3.3+/-0.15 | 4.2+/-0.71 |
| Tb.Sp (µm) | 375.2+/-38 | 250.4+/-21 | 113.2+/-5.4 | 84.4+/-13.7 |
| TB.Pf ($mm^{-1}$) | 43.7+/-2.5 | 37.33+/-2.7 | 21.5+/-0.92 | 19.5+/-2.6 |
| Tb.Th (µm) | 44.4+/-1.5 | 43.5+/-1.5 | 50.43+/-1.2 | 52.6+/-1.5 |
| SMI | 2.6+/-0.05 | 2.33+/-0.07 | 1.78+/-0.07 | 1.30+/-0.24 |

+/- SEM, n=8 or 9 mice

**P161**

**Reference values for paravertebral muscle size and myosteatosis in 21 to 80 years Chinese community adults, a nationwide, multicenter study**

*Wenkai Wu[1], Fangfang Duan[2], Renxian Wang[3], Ling Wang[4], Yajun Liu[1]*
*[1]Beijing Jishuitan Hospital- Capital Medical University, Department of Spine Surgery, Beijing, China ; [2]Beijing Jishuitan Hospital- Capital Medical University, Clinical Epidemiology Research Center, Beijing, China ; [3]Beijing Research Institute of Traumatology and Orthopaedics- Beijing Jishuitan Hospital, JST sarcopenia Research Centre, Beijing, China ; [4]Beijing Jishuitan Hospital- Capital Medical University, Department of Radiology, Beijing, China*

**Abstract Text**

**Objectives:** With increasing age, the paravertebral muscles suffer severe size loss in the human body. However, reference values to describe the differences in muscle parameters in people of different ages are still lacking. Here, we aimed to establish reference values for psoas and paraspinal muscle parameters in an adult Chinese community population.

**Methods:** This cross-sectional study used baseline data from the China Action on Spine and Hip (CASH) cohort. A total of 4120 community-dwelling participants (21-80 years) from 12 regions in China were analyzed. Psoas and paraspinal muscle area (PMA and PSMA) and density (PMD and PSMD) and paraspinal muscle fat fraction (PSMFF) at the L3 and L5 levels were manually segmented and measured. Age-related reference values were constructed as percentile charts using the lambda-mu-sigma (LMS) method.

**Results:** Reference percentiles for paravertebral muscles showed an age-related decrease in muscle area and density and an increase in muscle fat fraction. The differences between reference values for 26- and 76-year-olds were -5.47%/-23.71% in L3-PMA, -8.12%/-27.35% in L3-PSMA, -19.37%/-15.95% in L3-PMD, and -26.95%/-21.58% in L3-PSMD (female/male). Most parameters showed the greatest differences between 71 and 76 years of age. Specifically, L5-PSMA showed the greatest difference between 41-46 (men) and 46-50 (women) years. And the L3-/L5-PMA and PMD and L5-PSMFF in women showed the greatest differences around 51-66 years. Men generally had higher muscle area and density than women, and women had higher paraspinal muscle fat fraction than men (P<0.001).

**Conclusions:** We calculated reference values for PMA, PMD, PSMA, PSMD, and PSMFF in a Chinese community-dwelling adult population. The most rapid decrease in muscle area and density occurred in the oldest age group (71-76 years). While L5-PSMA in both sexes (41-51 years) and psoas in women (51-66 years) showed the greatest differences in middle age.

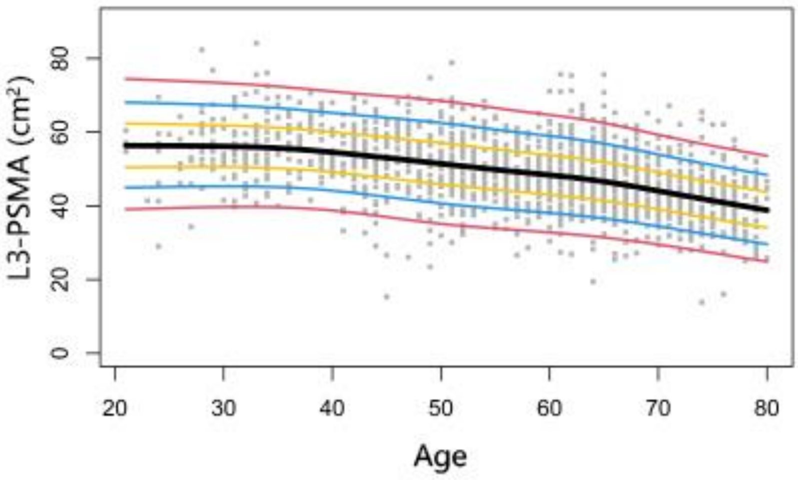

**Figure 1.** Percentiles of L3 paraspinal muscle cross-sectional area in men

**P162**

**Extracorporeal shock wave promoted macrophage polarization and inhibited muscle fat infiltration**

*<u>Wenkai Wu</u>[1], Renxian Wang[2], Dingding Wang[2], Anyi Guo[2], Quanzhong Ren[2], Lining Xu[2], Yajun Liu[1]*
*[1]Beijing Jishuitan Hospital- Capital Medical University, Department of Spine Surgery, Beijing, China ; [2]Beijing Research Institute of Traumatology and Orthopaedics- Beijing Jishuitan Hospital-, JST sarcopenia Research Centre, Beijing, China*

**Abstract Text**

**Objective:** Muscle aging is manifested by a decrease in muscle size and strength and an increase in muscle fat infiltration, but there is currently no effective treatment other than exercise. This study aimed to investigate the potential effects of extracorporeal shock wave therapy (ESWT) on intermuscular adipose tissue (IMAT) in vivo using a mouse model of muscle fat infiltration.

**Methods:** A tibialis anterior muscle fat infiltration (TAFI) mouse model was induced by glycerol injection (50ul). IMAT could be observed by HE staining 10 days after glycerol injection, then TAFI mice were treated with different energy of ESWT (for 2 weeks with different energies (1/2/3 bar), n=9) or without ESWT (GLY group, n=9) and compared with control group (with 50ul saline injection, n=9). Grip strength, and body weight were measured weekly. Flow cytometry was used to detect immune cells and FAP cells at 2 and 7 days after initial ESWT. HE staining, muscle transparency and fat 3D staining were performed 6 weeks after ESWT.

**Results:** The grip strength of the ESWT groups showed significantly faster recovery of grip strength than the GLY group at 2, 4, and 6 weeks after ESWT. Flow cytometry showed that glycerol injection significantly increased NK, DC, and macrophages (total, M1, and M2) ($P<0.01$). However, ESWT significantly increased total and M2 macrophages ($P<0.01$) compared with the GLY group, while M1 macrophages remained unchanged ($P>0.05$). In the 6th week after ESWT, volume imaging of IMAT showed that 2 and 3 bar ESWT caused significantly less IMAT percentage ($P<0.05$) (Figure 1).

**Conclusions:** ESWT promoted the recovery of glycerol-injured tibialis anterior muscle and inhibited muscle fat infiltration in the mouse. ESWT showed a dose-dependent inhibition of muscle fat infiltration, which may be achieved through macrophage recruitment and M2 polarization activation.

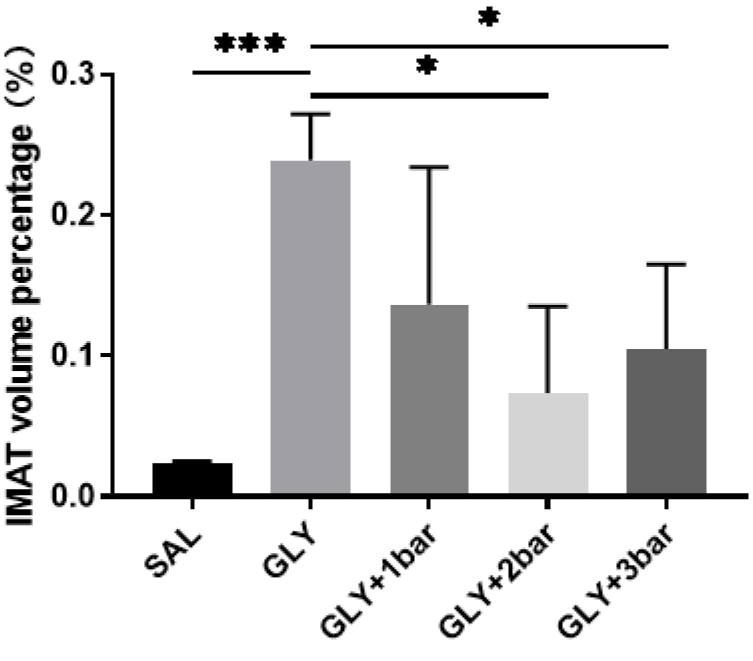

Figure 1. Volume analysis of 3D adipose staining in transparent tibialis anterior muscles

**P163**

**Application of Lower Extremity CT Auto Segmentation and AI-Based Pose Estimation for Sarcopenia Classification**

*Jun-Il Yoo[1], Deog Yoon Kim4 Kim[2], Young-Kyun Lee[3], Yong-Chan Ha[4], Dong-Won Byun[5]*
*[1]Inha University Hospital, Department of Orthopaedic surgery, Incheon, Korea- South ; [2]Kyung Hee University Hospital- Kyung Hee University School of Medicine, Department of Nuclear Medicine, Seoul, Korea- South ; [3]Seoul National University Bundang Hospital, Department of Orthopaedic Surgery, Seongna, Korea- South ; [4]Bumin Seoul Hospital, Department of Orthopaedic Surgery, Seoul, Korea- South ; [5]Soonchunhyang University College of Medicine, Division of Endocrinology and Metabolism- Department of Internal Medicine, Seoul, Korea- South*

**Abstract Text**

**Background:** Sarcopenia, the degenerative loss of skeletal muscle mass and strength, is a prevalent condition in older adults that can lead to functional decline and increased risk of falls and fractures. Accurate and efficient methods for the assessment and classification of sarcopenia are crucial for early detection and intervention.

**Methods:** In this study, we propose the application of lower extremity CT auto segmentation and AI-based pose estimation for sarcopenia classification. The lower extremity muscles of older adults were automatically segmented using advanced image processing techniques, which allowed for precise quantification of muscle volume, and cross-sectional area. Additionally, an artificial intelligence-based pose estimation algorithm was employed to assess muscle quality and asymmetry.

**Results:** The results showed a high level of accuracy and reproducibility in sarcopenia classification using our proposed method. The U-net based segmentation of lower extremity muscles enabled precise measurements of muscle volume, and cross-sectional area. The AI-based pose estimation using mediapipe algorithm effectively identified muscle quality and asymmetry, providing valuable information for sarcopenia classification.

**Conclusion:** The application of lower extremity CT auto segmentation and AI-based pose estimation offers a promising approach for sarcopenia classification. This integrated method provides accurate and efficient assessment of muscle volume, and cross-sectional area in older adults. Early detection and classification of sarcopenia using this approach can enable timely interventions and personalized treatment plans to prevent further functional decline and improve overall quality of life in older adults. Further research and validation studies are needed to establish the clinical utility and broader application of this approach in sarcopenia assessment and management.

**P164**

**Effect of Cortical Thickness and Individual Thigh Muscle Volume on Hip Fracture Severity**

*Jun-Il Yoo[1], Deog Yoon Kim Kim[2], Dong-Won Byun[3], Yong-Chan Ha[4], Yong-Kyub Lee[5]*
*[1]Inha University Hospital, Department of Orthopaedic surgery, Incheon, Korea- South ; [2]Kyung Hee University Hospital- Kyung Hee University School of Medicine, Department of Nuclear Medicine, Seoul, Korea- South ; [3]Soonchunhyang University College of Medicine, Division of Endocrinology and Metabolism- Department of Internal Medicine, Seoul, Korea- South ; [4]Seoul Bumin Hospital, Depatrment of orthopaedic surgery, Seoul, Korea- South ; [5]Seoul National University Bundang Hospital, Department of Orthopaedic Surger, Seongnam, Korea- South*

**Abstract Text**

**Background**: Hip fractures in the elderly are a significant health issue, often leading to high mortality. Factors like cortical thickness, osteoporosis, sarcopenia, and their relationship with physical performance are thought to influence hip fracture severity, but this relationship is not fully understood.

**Methods**: This study examines the impact of cortical thickness and thigh muscle volume on hip fracture severity in the elderly. A retrospective analysis of 60 elderly patients with hip fractures was conducted. Cortical thickness on the femur was measured using StradView software from the University of Cambridge. Thigh muscle volume was determined using an AI semantic segmentation model with a Dice Score of 0.84 applied to CT scans. Fracture severity was evaluated through radiographic and clinical assessment.

**Results**: The study found a significant correlation between cortical thickness and hip fracture severity; thinner cortices were associated with more severe fractures. Similarly, a positive correlation was found between lower thigh muscle volume and increased fracture severity. Additionally, a synergistic effect of low cortical thickness and lower muscle volume leading to more severe fractures was observed.

**Conclusion**: The findings highlight the significant impact of cortical thickness and thigh muscle volume on hip fracture severity in the elderly. Reduced cortical bone thickness and lower thigh muscle volume contribute to more severe fractures. Notably, specific thigh muscles correlated significantly with fracture severity, indicating their volume as a crucial indicator of physical performance. These insights underline the importance of targeted muscle strengthening and bone health strategies in elder care to reduce the severity of hip fractures.

**P165**

**Research on the Mechanism of Black phosphorus quantum dots Regulating Skeletal Muscle Fat Infiltration to Promote Muscle Regeneration**

*Dingding Wang[1], Wenkai Wu[2], Quanzhong Ren[1], Lining Xu[1], Anyi Guo[1], Hui Qi[1], Renxian Wang[1], Yajun Liu[1,2]*
*[1]Beijing Jishuitan Hospital- Capital Medical University, Beijing Research Institute of Traumatology and Orthopaedics, Beijing, China ; [2]Beijing Jishuitan Hospital- Capital Medical University, department of spine surgery, Beijing, China*

**Abstract Text**

Sarcopenia, an age-related progressive skeletal muscle disease, is characterized by a decline in overall muscle mass and/or muscle strength and function. Impaired activity and function of skeletal muscle stem cells, along with fat infiltration, are prominent features of sarcopenia. Understanding effective strategies to prevent fat infiltration in skeletal muscle and promote muscle regeneration is crucial for comprehending and treating aging and degenerative diseases, including sarcopenia.
Black phosphorus quantum dots (BPQD), a novel nanomaterial, have demonstrated favorable biocompatibility and have been extensively employed in biomedical applications such as tumor photothermal therapy and chronic inflammation. However, their potential use in treating sarcopenia has not yet been explored.
In this study, we investigated the effects of BPQD on myogenic and adipogenic differentiation in C2C12 myoblasts and human adipose-derived mesenchymal stem cells. qRT-PCR analysis revealed that the BPQD-treated group exhibited a significant upregulation of myogenic marker genes MyoG and MyoD expression, as well as a significant downregulation of the adipogenic marker gene PPARγ expression, compared to the differentiation group.
Our findings indicate that BPQD may play an important role in promoting skeletal muscle regeneration by suppressing fat infiltration, offering new insights into the treatment of aging and degenerative diseases, including sarcopenia.

**P166**

**A 5-yr longitudinal study of skeletal muscle group-specific atrophy with aging and the association with loss of muscle strength and physical performance**

*Yi Yuan[1], Wenshuang Zhang[1], Fengyun Zhou[1], Ling Wang[1], Xiaoguang Cheng[1]*
*[1]Peking University Fourth School of Clinical Medicine- Beijing Jishuitan Hospital, Department of Radiology, Beijing, China*

**Abstract Text**

**Background:** A progressive decline in skeletal muscle mass and muscle strength is well-known to be caused by atrophy with aging.

**Purpose:** Our objective was to examine the 5-yr changes in muscle size and density of different skeletal muscle groups in older individuals and explore the longitudinal associations of changes in muscle groups size and density with changes in handgrip strength (HGS) and the Timed Up and Go test (TUG).

**Methods:** Participants were 120 older individuals who performed baseline and follow-up HGS, TUG and quantitative computed tomography (QCT) scanning 5 years apart. From the CT images, cross-sectional area ($cm^2$) and density (HU) were measured for the mid-thigh, hip, and trunk muscle groups at L2 level. Changes in the muscle properties from baseline to the 5-yr follow-up were compared. Linear regression models estimated the association of changes in muscle properties with changes in HGS and TUG.

**Results:** The mid-thigh, hip and trunk muscle groups areas and density declined over the 5 years (all $P<0.01$). The magnitude of mid-thigh muscles and trunk muscles atrophy were greater in individuals over 70 years ($P<0.05$). After adjustment, associations with changes in HGS were significant in females for changes in mid-thigh muscle size ($\beta=0.75$, $P=0.03$), trunk muscle size ($\beta=0.69$, $P=0.01$) and in males for changes in mid-thigh muscle size ($\beta=0.67$, $P=0.02$). Associations with changes in TUG were significant in females for changes in muscle size of mid-thigh ($\beta=-1.14$, $P=0.04$) and in males for changes in trunk muscle size ($\beta=-3.48$, $P=0.02$).

**Conclusions:** Atrophy of mid-thigh and trunk muscle groups are associated with loss of muscle strength and physical performance. A comprehensive understanding of skeletal muscle group-specific atrophy would aid development of muscle group-specific interventions to mitigate the aging process and improve the quality of life of older individuals.

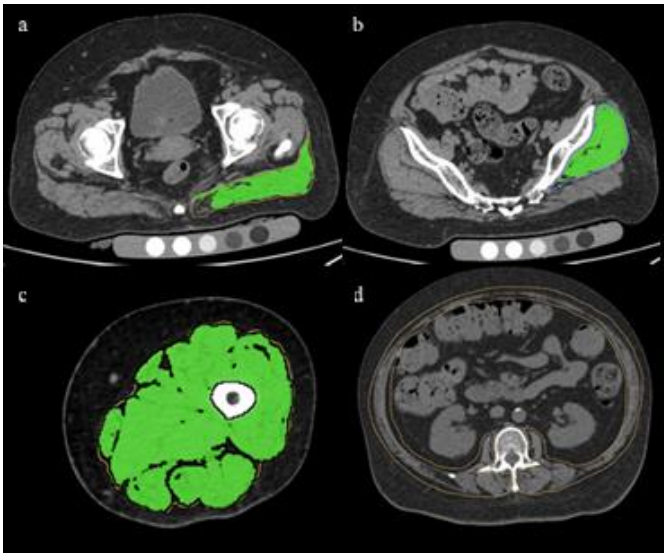

**P167**

**What are risk factors for osteosarcopenia amongst hip fracture patients?**

*Ronald Man Yeung Wong*[1]*, Pui Yan Wong*[1]*, Chaoran Liu*[1]*, Wai Wang Chau*[1]*, Hong Tim Lau*[1]*, Mei Shuen Chan*[1]*, Ning Zhang*[1]*, Chi Yin Tso*[2]*, Ning Tang*[2]*, Wing Hoi Cheung*[1]
[1]*The Chinese University of Hong Kong, Department of Orthopaedics & Traumatology, Hong Kong, China ;* [2]*Prince of Wales Hospital- Hospital Authority, Department of Orthopaedics & Traumatology, Hong Kong, China*

**Abstract Text**

**Background:** Hip fractures are the most serious form of complication of osteoporosis and are a global health concern with high rates of mortality. Previous studies have shown that fragility fracture patients have high prevalence of sarcopenia as well. Osteosarcopenia occurs when there is presence of both osteopenia/osteoporosis and sarcopenia, and studies have shown that the entity increases the risk of mortality and disability. In fact, there is also evidence that osteosarcopenia may pose risk of earlier death when compared to osteoporosis and sarcopenia alone. Identifying the risk factors for osteosarcopenia amongst hip fracture patients may therefore prove useful clinically.

**Purpose:** The objective of this study was to determine risk factors of osteosarcopenia amongst hip fracture patients.

**Methods:** Elderly patients with fragility hip fractures were recruited, and ethical approval was obtained. The Asian Working Group on Sarcopenia (AWGS) 2019 consensus was used to diagnose sarcopenia. Appendicular skeletal muscle mass (ASM) was identified with bioelectrical impedance analysis (BIA). Physical performance was assessed by 5-time chair stand test. Muscle strength was assessed by handgrip strength. Sarcopenia was defined with presence of low ASM and either low muscle strength or low physical performance. Dual-energy X-ray Absorptiometry (DEXA) was used to determine bone mineral density. Risk factors were identified with logistic regression. A p-value ≤0.05 was considered to be statistically significant.

**Results:** Our analysis showed that age and BMI (body mass index) <23kg/m^2 had significantly increased risk of osteosarcopenia amongst hip fracture patients. Furthermore, the presence of sarcopenia was > 90% in hip fracture patients, with osteosarcopenia >88%.

**Conclusion:** Our study showed that age and BMI<23kg/m^2 were risk factors for osteosarcopenia in hip fracture patients. Further research on optimal body weight and BMI may be useful.

**Acknowledgement:** This study was supported by the General Research Fund, Research Grants Council of the HKSAR, China (Ref: 14116223)

**P168**

**Growth Differentiation Factor 15 in Type 2 Diabetes: The Effect Of Exercise Training and its Relationship to Musculoskeletal Health.**

*Lorenza Mattia*[1], *Fatma Gossiel*[2], *Jonida Haxhi*[1], *Stefano Balducci*[1], *Giuseppe Pugliese*[1], *Richard Eastell*[2]
*[1]University of Rome La Sapienza, Department of Clinical and Molecular Medicine, Rome, Italy ; [2]University of Sheffield, Department of Oncology and Metabolism, Sheffield, United Kingdom*

**Abstract Text**

**Introduction:** Fracture risk is increased in type 2 diabetes (T2D). Growth Differentiation Factor-15 (GDF15), a senescence-associated secretory phenotype protein, is increased in T2D. Exercise can reduce fracture risk. It is not known whether exercise can decrease GDF15 in T2D.
Purpose: To evaluate GDF15 levels in T2D and examine their relationship to measures of musculoskeletal health and the impact of an exercise intervention.

**Methods:** This was a cross-sectional analysis of 64 trained T2D who completed 2 years of combined exercise program, 54 untrained T2D, and 48 non-diabetic controls, aged 65 to 75 years. We measured glucose levels and kidney function, bone outcomes (BMD, TBS, BTMs, calcaneal QUS and pQCT), physical performance (short physical performance battery -SPPB), physical fitness (expressed by VO2peak and metabolic equivalents, METs), muscle strength, mass and density. Physical activity (PA) level was assessed using questionnaires. Serum GDF15 was measured using an ELISA (R&D Systems).

**Results:** GDF15 significantly differed among groups, with the highest levels in untrained T2D (figure;*p<0.05,***p<0.001). Trained T2D had better muscle (but not bone) outcomes than untrained counterparts. In T2D, GDF15 was negatively associated with SPPB (ρ-0.18, *p*0.05), VO2peak (ρ-0.25, *p*0.005), METs (ρ-0.27,*p*0.003), PA level (ρ-0.2,*p*0.03), muscle density (ρ-0.23,*p*0.02), BMI (ρ -0.2, *p*0.04), and eGFR (ρ-0.24,*p*0.01). Multiple linear regression analysis revealed that SPPB (β-0.4, *p*0.000), BMI (β-0.3,*p*0.008), and eGFR (β-0.24,*p*0.018) were independent determinants of GDF15 in T2D. Longitudinal analysis of 15 T2D participants showed significant GDF15 changes over 2-year follow-up (Δ-415.6; *p*0.03): GDF15 decreased by -155.7pg/ml in trained T2D, with improvements in VO2, METs, SPPB and kidney function, while it increased by +260pg/ml in untrained T2D.

**Conclusions:** Exercise training in T2D was associated with GDF15 reduction, supporting the hypothesis that exercise may counteract the ageing process in T2D and that GDF15 may mediate this response. GDF15 might influence fracture risk by exerting its actions on muscle (but not bone) function.

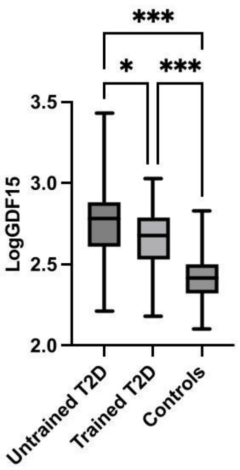

Figure – box-and-whisker plots of GDF15 levels in three groups compared after log-transformation by ANOVA (p<0.001);
the groups were then compared by Scheffe test, * p<0.05, *** p<0.001.

**P169**

**Time Course of Disuse Muscle Atrophy During Acute Immobilization Following Acetabular Fracture**

*<u>Yufeng Ge</u>[1], Minghui Yang[1], Xinbao Wu[1], Ling Wang[2]*
*[1]Beijing Jishuitan Hospital- Capital Medical University, Department of Orthopaedics and Traumatology, Beijing, China ; [2]Beijing Jishuitan Hospital- Capital Medical University, Department of Radiology, Beijing, China*

**Abstract Text**

**Background:** Disuse muscle atrophy (DMA) resulting from immobilization, particularly in patients with fractures, is a recognized phenomenon. However, the specific dose-response relationship between the rate of DMA and the duration of immobilization in fracture patients is not well-defined.

**Purpose:** To investigate the time-dependent progression of DMA during immobilization in patients with acetabular fractures.

**Methods:** Data from 161 acetabular fractures patients (age: 47.1±14.8 years, 22.4% female) at a trauma center in Beijing, China were retrospectively analyzed. Each patient underwent two hip CT scans: the first within three days post-injury and the second within 28 days, during which they were confined to bed rest. The cross-sectional area (CSA) of the muscle at the upper-thigh level were measured and quantitative DMA was then calculated ((2nd CSA-1st CSA)/ 1st CSA, %). Generalized additive and piecewise linear regression models were employed to explore the nonlinear relationship and the threshold effect of immobilization duration on muscle loss.

**Results:** Significant increases in DMA correlated with prolonged immobilization (β -1.17, 95%CI -1.41, -0.93, P<0.001). An inverted S-shaped pattern emerged in the dose-response relationship between the duration of immobilization and DMA. Two inflection points, approximately on day 7 and 17 was identified (likelihood ratio test *P*=0.045). The rate of DMA was not significant prior to day 7 (β 0.1, 95%CI -0.98, 1.17, P=0.861), after which a marked increase in DMA rate was statistically significant (β -1.47, 95%CI -2.05, -0.89, *P*<0.001). After day 17, this rate appeared to be slower (β -1.12, 95%CI -3.71, 1.46, *P*=0.422).

**Conclusion:** The trajectory of DMA in patients with acetabular fractures follows a distinct nonlinear pattern, described as a "slow-rapid-slow" trend. Rapid rate of DMA is observed during the second and third week of immobilization. These insights are critical for developing effective strategies to preserve muscle mass and mitigate the adverse effects of prolonged immobilization in fractured patients.

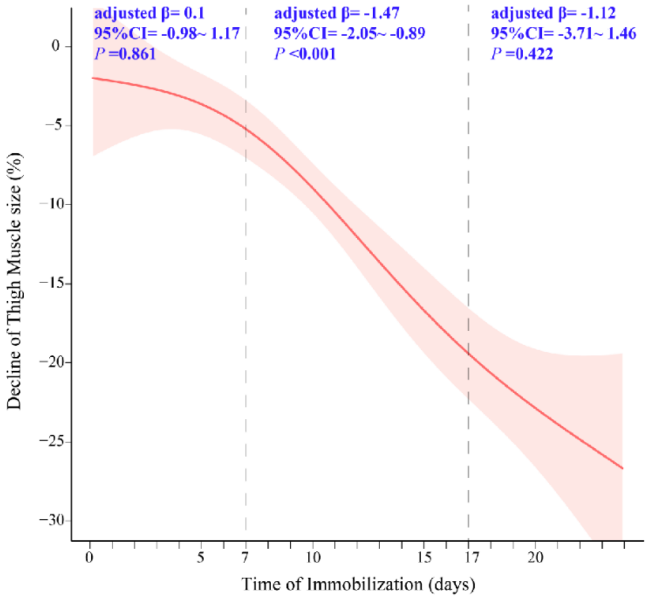

**Fig.** Nonlinear “slow-rapid-slow” relationship between disuse muscle atrophy and immobilization duration.
*Ajustment for age and sex

**P170**

**The effect of resistance exercise on peak bone density of young adults**

*Georgios Nagornis*[1], *Christos Valkanis*[2], *Eleni Douda*[3], *Christos Bogiatzis*[1]
*[1]General University Hospital of Alexandroupolis, Orthopedic Clinic, Alexandroupoli, Greece ; [2]Health Center, Orthopedic Outpatient Clinic, Alexandroupoli, Greece ; [3]Democritus University of Thrace, Department of Physical Education and Sports Sciences, Komotini, Greece*

**Abstract Text**

**Introduction:** Osteoporosis burdens the global population due to its high prevalence and morbidity by causing fragility fractures. Resistance training prevents osteoporosis by significantly reducing the rate of age-related bone loss.

**Purpose:** The purpose of this work was to study the effect of resistance exercise on increasing peak bone density in a young population.

**Methods:** Forty young adults (men n=20, women n=20) voluntarily participated in the study and were divided into groups according to whether they exercised (Exercise Group: men, n=9 & women, n=10 and Control Group: men, n=11 & women, n=10). Athletes exercised at least 3 times per week for the past 3 years, performing at least 2 sets of 10-12 repetitions per muscle group, for at least 8 muscle groups. Measurements performed included laboratory blood tests and bone mineral density with DEXA of the lumbar spine and hip (BMD, T-Score, Z-Score).

**Results:** Data analysis showed that the exercising group had higher bone density than the nonexercising group in the lumbar spine ($p<.001$) and the hip ($p<.01$). Specifically, the effect of the Exercise factor was statistically significant on the total BMD of the vertebrae ($p<.001$), the T-Score ($p<.001$) and the Z-Score ($p<.001$) as well as the neck ($p<.05$ for BMD, T-Score, Z-Score) and total hip ($p<.01$ for BMD, T-Score, Z- Score) respectively. Regarding the Gender factor, a statistically significant effect was observed on the T-Score ($p<.05$) and Z-Score ($p<.01$) of the total BMD of the vertebrae and the total BMD of the neck and hip ($p<.05$), while no significant differences were observed in the laboratory test.

**Conclusion:** The results of the present study show that resistance training from a young age has a positive effect on bone density with an increase in peak bone mass values. Consequently, it could be a preventive measure against osteoporosis by delaying its onset.

**P171**

**Muscle transcriptomic signatures and bone quality modifications in a murine model of reversible immobilization**

*<u>Hélène Libouban</u>[1], Méline Wery[2], Morgane Mermet[1], Quentin Massiquot[2], Marie Veziano[1]*
*[1]University of ANGERS, Inserm RMES-REGOS, Angers, France ; [2]University of Angers, Sfr ICAT, Angers, France*

**Abstract Text**

**Background:** Long term immobilization induces a severe muscle wasting and bone loss. A physical and biochemical link between the two organs exists. Around twenty mediators of bone and muscle communication has been identified and although some of them have been targeted to prevent disuse bone loss and fragility, none of them has proven to be completely effective.

**Purpose:** To establish in an animal model of reversible immobilization the link between bone quality data and the muscle transcriptomic signatures over the time.

**Methods:** 40 female mice were spread into 5 groups. At day 0, 32 mice received Botulinum toxin (BTX) injection in the quadriceps to induce paralysis of the right hindlimb. Mice were sacrificed at 7, 14, 21, 28, 56 days post-BTX. The 56-day period correspond to the recovery of disuse. The remaining group was not injected and sacrificed at day 0. Bone strength and quality were measured on femur by three points-binding, microCT, histomorphometry and Raman microscopy. A tow-way ANOVA was used to determined significant differences between times and sides. RNAseq was performed on 6 right quadriceps per time. Genes expression was compared from 7 to 56 days with the one from day 0. Differentially expressed genes were selected with p-value<0.05 and $Log_2FC$ of >0.6 or <-0.6).

**Results:** Ultimate load and stiffness were reduced compared to the left side at 21 days (resp. 11.3N±1.1 *vs* 12.8N±1.5 and 47N/mm±5 *vs* 67N/mm±1, $p<0.05$) and were recovered at 56 days. Cortical area expressed in percentage of the total area (Ct.Ar/Tt.Ar) was reduced at 21 days (41.4%±0.9 *vs* 44.8%±1.8, $p<0.05$). We identified 1869, 1569, 923 and 83 down-regulated and 1954, 1706, 836 and 169 up-regulated mRNAs respectively from 7 to 56 days.

**Conclusions**: The association of bone quality data with transcriptomic signatures help to understand better muscle and bone interaction during muscle atrophy and recovery periods.

**P172**

**Association of adiponectin with lean mass in patients with inflammatory bowel disease**

*IULIA SIMONA SOARE*[1]*, Anca Sirbu*[2]*, Mircea Diculescu*[3]*, Bogdan Mateescu*[4]*, Cristian Tieranu*[5]*, Simona Fica*[2]
[1]*University of Medicine and Pharmacy Bucharest, Endocrinology, Bucharest, Romania ;* [2]*University of Medicine and Pharmacy Carol Davila Bucharest, Endocrinology Elias Hospital, Bucharest, Romania ;* [3]*University of Medicine and Pharmacy Carol Davila Bucharest, Gastroenterology Fundeni Institute, Bucharest, Romania ;* [4]*University of Medicine and Pharmacy Carol Davila Bucharest, Gastroenterology Colentina Hospital, Bucharest, Romania ;* [5]*University of Medicine and Pharmacy Carol Davila Bucharest, Endocrinology, Bucharest, Romania*

**Abstract Text**

**Background:** Inflammatory bowel disease (IBD), comprising Crohn's disease (CD) and ulcerative colitis (UC), represents a chronic gastrointestinal disease. Adiponectin is the most abundant peptide secreted by adipocytes, whose reduction plays a central role in obesity-related diseases. Moreover, recent studies have shown a new potential impact of adiponectin on muscle mass.

**Aim of the study:** Because adiponectin represents a promising candidate marker, the current study wanted to describe the impact of adiponectin on muscle mass in IBD patients.

**Methods:** Anthropometric data were collected, and all patients underwent blood adiponectin measurements. ASMI (appendicular muscle skeletal index) was determined using dual-energy X-ray assessment (DXA) of the whole body. High glucocorticoid exposure was considered to be more than three pulses of prednisone >7.5 mg/day for more than three months. Fecal calprotectin (FC) was documented in the records of all patients.

**Results:** A multicentric cross-sectional study was conducted on 84 IBD patients (50 CD), 45 women, with a median age of 43 (IQR 23) years. ASMI negatively correlated with adiponectin when adjusted for weight and gender ($r=-0.32$, $p=0.03$) and with FC ($r=-0.397$, $p<0.05$).

Linear regression showed adiponectin ($\beta=-0.1098$, $p=0.03$) and fecal calprotectin ($r=-0.394$, $p<0.05$) as independent factors for ASMI in CD patients in an analysis that also included age, gender, high glucocorticoid exposure, weight.

**Conclusion:** In the present study, adiponectin was negatively associated with ASMI. Abnormally high adiponectin levels may harm muscle cells by excessively activating AdipoR signaling. Further studies should be conducted.

**P173**

**An occupational therapy project and its effectiveness in post femoral fractured patients affected by sarcopoenia**

*Ferdinando D Amico*[1]*, Rossella D'Amico*[2]
[1]*University of Messina, Biomorphological Science, Messina, Italy ;* [2]*IRCCS Oasi Troina Enna, Rehabilitative Medicine Unit, Troina Enna, Italy*

**Abstract Text**

**Objective:** 93 elderly patients with femoral fractures have been evaluated following surgery. The aim of the integrated rehabilitative occupational therapy programme was to evaluate the prevalence of sarcopoenia and to reestablish the functional condition prior to femoral fracture.

**Methods**: They have been assessed through a multifaceted (orthopedic-geriatric-rehabilitative) approach using MMSE, BADL, IADL, Barthel Index. Osteoporosis and Sarcopoenia were assessed through DEXA Bone Densitometry.

**Results:** The standing position recovery for 57 patients started within 3 days after prosthesis surgery due to femoral fracture. They were dismissed after a 15/25-day hospitalization. 34 elderly subjects recovering from osteosynthesis regained the sitting position in 2-3 days, load tests were made between 7 and 14 days and they left the unit 30/45 days after admittance. At discharge 10 subjects affected by femoral fracture and sarcopoenia (mean age 78±5) were moved to the Extended Care Unit for lack of assistance at home. There they followed an occupational therapy programme including aims like: 1) performing lower limbs mobilization through specific exercises; 2) working on muscle fibers type 2 to counterbalance the muscle loss. The group including patients following the programme was then compared to one including 8 subjects affected by femoral fracture and sarcopoenia (mean age 77± 6) discharged and going home to their caregivers after femoral fractures. A 6-month individual occupational therapy programme at the Extended Care Unit showed: 1) improvement in motor skills detected through scales scores (BADL 3.3/6 > 4.5/6 - IADL 2.5/8 > 5.7/8 - Barthel Index 50/100 > 90/100); 2) improvement both in muscle mass and muscle strength.

**Conclusion:** Effectiveness of an occupational therapy programme focused on walking ability and muscle strength recovery, aimed at patients discharged after femoral fractures and osteosynthesis, was evaluated. The Occupational Therapist approach was customized in order to make the patient regain his self-assurance and independence.

**ND-P019**

**Effects on Bone of Combinations of Physical Training and Nutritional Supplementation in a Microgravity Analog Model in Mice: the NEBULA study**

*Théo Fovet[1,2], Margot Issertine[2], Mathilde Lepileur[1,3], Norbert Laroche[1], Thomas Brioche[2], Angèle Choppard[2], Laurence Vico[1], Maura Strigini[1]*
*[1]University of Saint-Etienne, sainbiose-LBTO INSERM U1059, Saint-Etienne, France ; [2]Montpellier University- INRAE, DMeM, Montpellier, France ; [3]University Lyon 1, Biology, Lyon, France*

**Abstract Text**

**Background:** Current astronaut training programs can limit, but not completely prevent, musculoskeletal deconditioning; moreover, bone recovery remains incomplete even two years after return to Earth, calling for improved countermeasures.

**Purpose:** NEBULA (Nutrition and Exercise Biology for UnLoaded Astronauts) addresses these needs by evaluating combinations of physical exercise and nutritional supplementation in the murine hindlimb unloading (HU) microgravity model.

**Methods and Results:** First, 6 groups (n=10 each) of 14-week-old males C57B6J underwent 3 weeks of preconditioning exercise, followed by 7 or 21-day unloading (HU7 and HU21). Exercise consisted of progressive high intensity endurance and resistance training. Femurs were examined after preconditioning (HU0), at HU7 and HU21. Micro-computed tomography of femur distal metaphysis showed greater trabecular bone fraction (BV/TV, +25%; p<0.001) in preconditioned groups (PC) compared to control (Ctrl) at HU0. At HU7, trabecular BV/TV was preserved in PC while it decreased in Ctrl. However, at HU21, BV/TV was similarly decreased in the two. Histomorphometry confirmed the delay of trabecular bone deterioration in PC at HU7.

In the second part of NEBULA (8 groups, n=10 each), exercise and a nutritional supplement formula (nacre powder, betahydroxybetamethylbutyrate and LErgothionein) were employed before, during and after HU. Femurs were collected at HU0, HU21 and at 7 or 28-day of reloading (R7, R28). BV/TV was greater (p<0.01) in Experimental compared to Ctrl groups at most time points (HU0, HU21, R28). At H21 BV/TV was still preserved in Experimental, while it decreased, as expected, in Ctrl: BV/TV HU21 in Ctrl was 44% lower than in Experimental (p<0.0001) and this difference remained at R28 (-26%, p<0.01) (AuthorisationEthicalCommittee MontpellierUniversity).

**Conclusion:** Taken together, these results show that preflight training can delay bone deterioration and the relevance of the countermeasure combination tested to preserve bone mass and architecture. Further tests are underway to better dissect the relative contributions of exercise and nutrition.

**ND-P020**

**Association between directly measured free testosterone levels and sarcopenia in Korean elderly men: insights from the Ansung cohort study**

*Young-Sang Kim*[1], *Yu Byung Yeon*[2], *Han Kunhee*[3]
[1]*Cha Bundang Medical Center, Family Medicine, Seongnam, Korea- South ;* [2]*Konyang University Hospital, Family Medicine, Daejeon, Korea- South ;* [3]*Seoul Medical Center, Family Medicine, Seoul, Korea- South*

**Abstract Text**

**Background:** Previous studies have established an association between low testosterone levels and sarcopenia. However, most investigations focusing on free testosterone (FT) have relied on calculated levels derived from formulas rather than direct measurements.

**Purpose:** This study aimed to examine the relationship between directly measured FT levels and sarcopenia in elderly Korean men.

**Methods:** Data from the Ansung cohort, a biennial population-based study, were utilized for this analysis. Data from the 8th wave (2015-2016) were analyzed. FT levels were assessed using a radioimmunoassay method. Sarcopenia is defined as having both low muscle mass (SMI < 7.0 kg/m2) and low grip strength (< 28 kg).

**Results:** The analysis included 1103 men aged 54-83. The median concentration of FT was 8.33 pg/mL. Participants were categorized into quartiles based on their FT levels. The prevalence of sarcopenia was 17.5%, 9.5%, 8.7%, and 6.5% across the quartiles, respectively (P<0.001). Cubic spline analysis indicated a significant increase in sarcopenia risk for FT levels below 8 pg/mL. After adjusting for age and body mass index, 1-unit increase in FT was associated with a odds ratio of 0.900 (0.844–0.960) and 0.922 (0.858–0.988) for low muscle mass and grip strength, respectively.

**Conclusion:** This study provides evidence of an association between low directly measured FT levels and sarcopenia in elderly Korean men. These findings align with previous studies that relied on calculated FT levels. Future investigations incorporating measurements of sex hormone-binding globulin and other sex hormones may enhance our understanding of the relationship between sex hormones and sarcopenia.

**Poster Sessions: Osteoporosis: evaluation and imaging**

**P174**

**Prednisolone Use Is Associated with Impaired Changes in Trabecular Bone Score in Children and Adolescents with 21-Hydroxylase Deficiency Congenital Adrenal Hyperplasia: A Short-term Follow-up Study**

*Pattara Wiromrat[1], Phanpaphorn Namphaisan[2], Ouyporn Panamonta[1], Nantaporn Wongsurawat[3], Chatlert Pongchaiyakul[4]*
*[1]Khon Kaen University, Pediatrics, Khon Kaen, Thailand ; [2]Khon Kaen University, Pediatrics, Khon Ksen, Thailand ; [3]Khon Kaen University, Nuclear Medicine, Khon Kaen, Thailand ; [4]Khon Kaen University, Internal Medicine, Khon Kaen, Thailand*

**Abstract Text**

**Background:** Individuals with 21-hydroxylase deficiency (21OHD) congenital adrenal hyperplasia have an increased risk of fractures due to chronic glucocorticoid replacement which may compromise bone microarchitecture.

**Purpose:** We aimed to assess changes in trabecular bone score (TBS), a measure of bone microarchitecture, and its associated factors in children and adolescents with classic 21OHD.

**Methods:** Thirty-six children and adolescents with classic 21OHD, who had complete baseline data for anthropometry, lumbar spine bone mineral density (LSBMD), and bone turnover markers (BTMs), were recruited for a follow-up LSBMD scan. The iNsight™ software was used to analyze TBS on LSBMD images. Participant height, body mass index (BMI), LSBMD and TBS were converted to z-score according to bone age (BA). The change in TBS was expressed as percentage of TBS change per year (%ΔTBS/y).

**Results:** At baseline, participants (55% female, 69% salt-wasting type, 61% taking prednisolone) had mean (± SD) age of 15.2 ± 4.1 years, BA 17.2 ± 3.0 years, height z-score -2.1 ± 1.0, and BMI percentile (BMI%tile) 58.7 ± 36 kg/m$^2$. The mean follow-up duration was 1.4 ± 0.5 years. The average glucocorticoid dose (hydrocortisone equivalent) over the follow-up period was 18.1 ± 5.6 mg/m$^2$/day. The baseline median (Q1-Q3) LSBMD z-score was 0.2 (-0.6-0.8), TBS 1.47 (1.37-1.54) and TBS z-score -0.9 (-1.3-0.4), and %ΔTBS/y was -0.1 (-0.6-0.3) %/year. %ΔTBS/y correlated negatively with baseline BMI%tile (r=-0.395, p=0.017) and prednisolone use (r=-0.516, p=0.001), but not with sex, 21OHD subtype, glucocorticoid dose, baseline BA, 25-hydroxyvitamin D or BTMs. Regression analysis showed %ΔTBS/y was inversely associated with prednisolone use (B ± SE: 3.9 ± 1.7, p=0.025), adjusted for sex, baseline BA, and BMI%tile.

**Conclusion:** In children and adolescents with 21OHD, impaired bone microarchitecture is linked to prednisolone use but not the daily glucocorticoid dose. These findings support hydrocortisone as the preferred long-term glucocorticoid replacement for this patient population.

**P175**

**Trabecular bone score and frequency of fragility fractures in women with rheumatoid arthritis**

*Maria Kozyreva[1], Nikolay Demin[1], Oksana Nikitinskaya[1], Natalia Toroptsova[1]*
*[1]V.A. Nasonova Research Institute of Rheumatology, Department of Osteoporosis, Moscow, Russia C.I.S.*

**Abstract Text**

**Objective:** to assess the microarchitecture of bone using the trabecular bone score (TBS) and frequency of fragility fractures in postmenopausal women with rheumatoid arthritis (RA).

**Material and methods:** 95 postmenopausal women (mean age 62,3±8,1 years) with a confirmed RA. Clinical examination, laboratory tests and dual-energy X-ray absorptiometry (DXA) of the lumbar spine (L1–L4), proximal femur were done. Trabecular bone score (TBS) was assessed with "TBS Insight" software. Correlation analysis by Spearman was done. The study was approved by the local ethics committee.

**Results:** 85,3% and 81,8% of persons were seropositive by RF and ACCP, respectively. Oral glucocorticoids (GCs) were taken for≥ 3 months by 57,9% of persons (median duration 3,0 [1,8; 8,0] years and the cumulative dose 6,9 [3,7; 16,4] g). Fragility fractures had 34,7% women: vertebral - 11,6%, non-vertebral - 28,4% persons. Osteoporosis (OP) was diagnosed in 41 (43,1%) and osteopenia – in 34 (35,8%) of women. Degraded microarchitecture according to TBS was found in 38,9%, partially degraded - in 25,3%, and normal - in 35,8% of patients with RA. Among persons with OP normal microarchitecture was found in 5 (12,2%); partially degraded - in 15 (36,6%), and degraded microarchitecture- in 21 (51,2%) patients. Low TBS was significantly more often detected in women with a history of fragility fractures compared to persons without them (p<0,05). TBS negative correlated with age (r=-0,30, p=0,003), duration of postmenopausal period (r=-0,26, p=0,014), cumulative dose of GCs (r=-0,34, p= 0,045), and positive – with L1-L4 BMD (r=0,43, p<0,001), femoral neck BMD (r=0,21, p=0,038) and total hip BMD (r=0,23, p=0,02).

**Conclusion:** OP was found in 43,1% of postmenopausal women with RA. Degraded bone microarchitecture by TBS was detected in 38,9% of persons. Women with a history of fragility fractures significantly more often had low TBS. Value of TBS correlated with age, postmenopause duration, cumulative GCs dose and BMD of L1-L4 and proximal femur.

**P176**

**FRAX adjusted for trabecular bone score in patients with rheumatoid arthritis**

*Maria Kozyreva[1], Olga Dobrovolskaya[1], Oksana Nikitinskaya[1], Nikolay Demin[1], Natalia Toroptsova[1]*
*[1]V.A. Nasonova Research Institute of Rheumatology, Department of Osteoporosis, Moscow, Russia C.I.S.*

**Abstract Text**

**Objective:** to estimate the 10-year probability of fragility fractures adjusted for trabecular bone score (TBS) in postmenopausal women with rheumatoid arthritis (RA).

**Material and methods:** 95 postmenopausal women (median age 62,0 [57,0; 68,0] years) with confirmed diagnosis of RA (ACR/EULAR 2010) were included. Dual-energy X-ray absorptiometry (DXA)

was done, TBS was calculated using the "TBS Insight" software, version 3.0. 10-year probability of major osteoporotic fractures was assessed using the FRAX calculator (Russian version) without femoral neck (FN) BMD, with including the value of FN BMD and additionally adjusted for TBS. The study was approved by the local ethics committee.

**Results:** Normal bone microarchitecture (TBS ≥1,31) had 34 (35,8%) women, partially degraded (TBS between 1,23 and 1,31) - 24 (25,3%) persons, and degraded bone microarchitecture (TBS≤1,23) - 37 (38,9%) patients with RA. 24 (72,7%) patients with low-energy fractures had low TBS. Median risk of major osteoporotic fractures by FRAX without including FN BMD was 18,0 [12,0; 26,0], with correction on FN BMD – 20,0 [13,0; 31,0] and adjusted for TBS – 18,0 [13,0; 30,0]. High risk of fractures according to FRAX with including only clinical risk factors was found in 47 (49,5%) patients, when FN BMD was added - in 51 (53,7%) and adjusted for TBS - in 52 (54,7%) of examined women. The entry of TBS into FRAX allowed an additional 12 people to be identified with high risk, and 7 patients moved to the low-risk group. TBS negatively correlated with FRAX (r=-0,24, p<0,05) and positively – with L1-L4 BMD (r=0,43, p<0,001), FN BMD (r=0,21, p=0,038), TH BMD (r=0,23, p=0,02).

**Conclusion:** FRAX adjusted for TBS redistributed patients in risk groups, as a result 54,7% of RA patients had high probability of fragility fractures and needed anti-osteoporotic treatment.

**P177**

**Comparisons of Hounsfield units and volumetric bone density in discriminating vertebral fractures on lumber CT scans**

*Fengyun Zhou*[1], *Ling Wang*[1], *XiaoGuang Cheng*[1]
[1]*Beijing Jishuitan Hospital affiliated to Capital Medical University, Department of Radiology, Beijing, China*

**Abstract Text**

**Purpose:** To compare the performance of areal Hounsfield units (aHU), volumetric Hounsfield units (vHU), and volumetric bone mineral density (vBMD) by quantitative CT (QCT) in discriminating vertebral fractures (VFs) risk.

**Methods:** We retrospectively included CT scans of the lumber spine 101 VFs cases (60 women, mean age: 64 ± 4 years; 41 men, mean age: 73 ± 10 years) and sex- and age-matched 101 control subjects (60 women, mean age: 64 ± 4 years; 41 men, mean age: 72 ± 7 years). In order to assess the discriminatory capability of aHU, vHU, and vBMD measurements at the L1 and L2 levels in identifying vertebral fractures, we conducted binary logistic regression and receiver operating characteristic (ROC) curve analyses in men and women.

**Results:** In both men and women with and without VFs, aHU, vHU, and vBMD were highly correlated with each other ($r^2$ from 0.832 to 0.957, all p<0.001). There was a statistically significant difference in aHU, vHU, and vBMD between subjects with and without VFs (p<0.001). When age, gender and BMI were taken into account as covariances and adjusted simultaneously, odds ratios (ORs) for aHU, vHU, and vBMD values, which represent the risk of VFs, were significant (p<0.001). In both men and women, further, vBMD had higher values for AUC, sensitivity, specificity, PPV, and NPV compared to vHU, with vHU in turn surpassing aHU.

**Conclusions:** The QCT-measured volumetric bone mineral density is more associated with acute VFs than vHU and aHU values of the lumber spine. Although the use of vHU and aHU values for the diagnosis of osteoporosis and discriminating fracture risk is limited to scanner- and imaging protocol-specific, they have great potential for opportunistic osteoporosis screening, particularly vHU.

**P178**

**Bone mineral density T-scores comparison between obese and non-obese individuals included in a Fracture Liaison Service following a recent fragility fracture**

*Gauthier Marchasson[1], Cécile Philippoteaux[1], Isabelle Legroux-Gérot[1], Hélène Béhal[2], Bernard Cortet[1], Julien Paccou[1]*
*[1]Lille University Hospital, Rheumatology, Lille, France ; [2]Lille University Hospital, Biostatistics, Lille, France*

**Abstract Text**

**Purpose:** This study aimed to compare the mean T-scores of obese and non-obese patients after recent fragility fractures.

**Methods:** Over a period of 5 and a half years, from January 2016 to May 2021, patients from a fracture liaison service were identified and their demographic characteristics, osteoporosis risk factors, BMD T-scores, and fracture sites were compared between obese (BMI ≥ 30 kg/m²) and non-obese (19 kg/m² < BMI < 30 kg/m²) patients.

**Results:** A total of 712 patients were included (80.1% women; mean age 73.8 ± 11.3 years). Sixteen % had type 2 diabetes mellitus and 80% had a major osteoporotic fracture (MOF). 135 patients were obese and 577 non-obese, with obese patients younger ($p < 0.001$) and more frequently female ($p = 0.03$). Obese patients presented with fewer hip fractures (10% vs. 21%, $p = 0.003$) and more proximal humerus fractures (16% vs. 7%, $p < 0.001$) than non-obese patients. After adjusting for age, sex, and diabetes mellitus, BMD T-score values were significantly higher at all measurement sites (lumbar spine, total hip, and femoral neck) in obese patients than in non-obese patients for all types of fractures, with a mean difference of 1 standard deviation ($p < 0.001$ for all comparisons). The same results were observed in the population limited to MOF.

**Conclusions:** Given the crucial role of BMD T-score in determining the need for anti-osteoporotic medication following fragility fractures, it is reasonable to question the existing T-score thresholds in obese patients.

**P179**

**Validation of a New Point-of-Care Calcaneal Ultrasound Densitometer (BeeTLe)**

*Giovanni Adami[1], Maurizio Rossini[1], Davide Gatti[1], Paolo Serpi[2], Christian Fabrizio[2], Roberto Lovato[3]*
*[1]University of Verona, Rheumatology Unit, Verona, Italy ; [2]Caresmed SRL, Medical Imaging, Milan, Italy ; [3]Ospedale Casa di Cura Villa Berica, Bone Specialist Unit, Vicenza, Italy*

**Abstract Text**

**Introduction:**Dual-energy X-ray absorptiometry (DXA) represents the gold standard for measuring bone mineral density (BMD). However, its size and bulkiness limit its use in mass screening.

**Purpose:** To validate a new point-of-care ultrasound densitometer compared to gold-standard DXA.

**Materials and Methods:** We conducted a study to assess the repeatability, sensitivity, accuracy, and validation of a new ultrasound densitometer for the calcaneus (BeeTLe) compared to standard DXA. BMD (g/cm2) was measured at the femoral and lumbar spine levels using DXA (iLunar General Electric), and the Bone Quality Index (BQI, a dimensionless measure of bone quality) was measured with BeeTLe in patients attending the osteoporosis clinic at two bone specialist centers. The Bland-Altman test and simple linear regression were used to evaluate the association between values measured with the two instruments. Additionally, the ability of the T-score calculated with BeeTLe to identify patients with previous osteoporotic fractures was tested using ROC curves.

**Results:** A total of 201 patients (94.5% females) with a mean age of 62.1 ± 10.2 were included in the study. The BeeTLe instrument showed a coefficient of variation (CV, in 23 repeated measurements) of 1.22%, which was not statistically different from the CV of DXA (1.20%). Figure 1A shows the Bland-Altman plot for T-score differences at various sites. Figure 1B shows the regression curves between BQI and BMD at the femoral neck (r2 0.500, p <0.0001), total femur (r2 0.545, p <0.0001), and lumbar spine (r2 0.455, p <0.0001). Figure 1C displays the T score frequency with DXA and Beetle. Figure 1D presents the ROC curves describing the ability of BeeTLe and DXA at different sites to classify patients with fractures vs. non-fractured (AUC not significantly different).

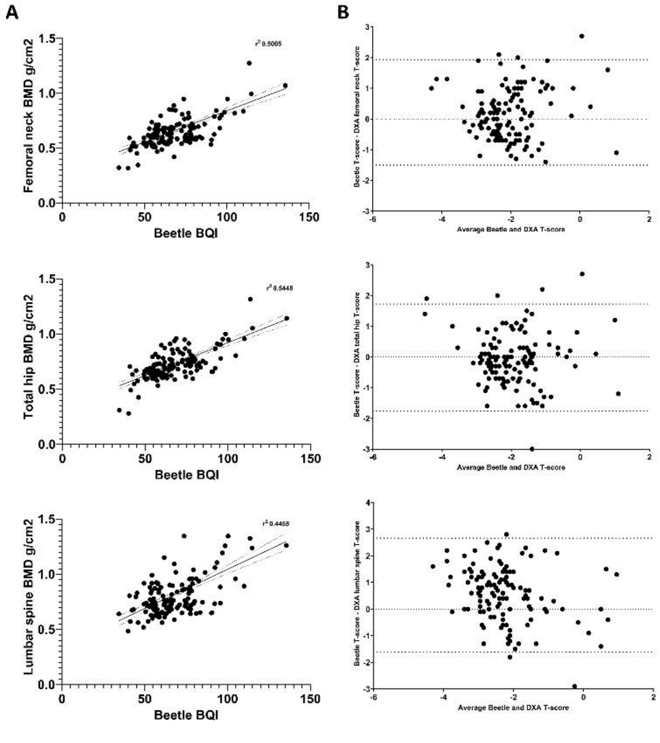

C

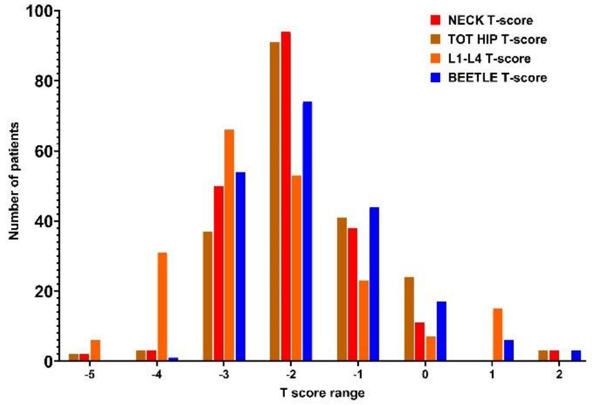

D

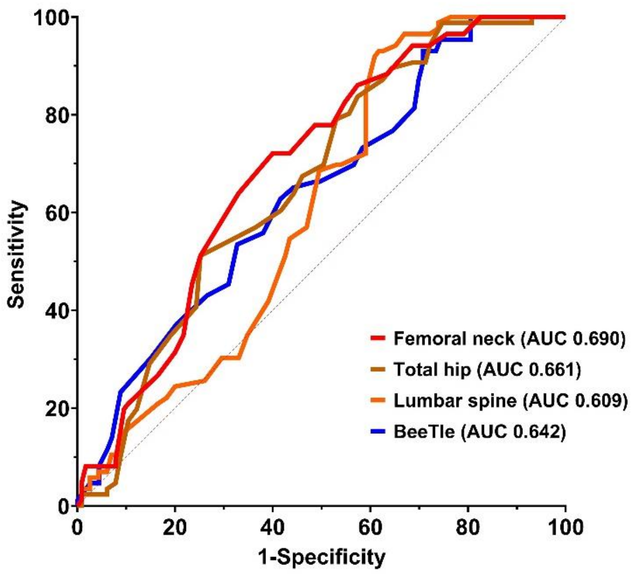

**Conclusion:** In this preliminary study, BeeTLe, a new point-of-care ultrasound densitometer, demonstrated good repeatability and performance similar to DXA. Therefore, its use can be proposed in screening for osteoporosis.

**P180**

**From 2D to 3D Imaging: 3Dshaper and EOS Edge applications in the Rotterdam study**

*Kirsty Huininga[1], Fjorda Koromani[1], Evert Van Velsen[1], Carola Zillikens[1], Fernando Rivadeneira[1]*
*[1]Erasmus MC, Internal Medicine, Rotterdam, Netherlands*

**Abstract Text**

**Introduction**: Areal bone mineral density (aBMD) values do not distinguish 3D properties of bone. 3D-Shaper® has been proposed as a method for partitioning volumetric cortical (cBMD) and trabecular (tBMD) from hip DXA scans. We aimed at assessing the contribution of cBMD and tBMD in interpreting aBMD and assessing fracture risk in a population-based setting.

**Methods**: Participants (n=2131) comprised 55% women, mean age 75 (SD=5.78) years. Incident osteoporotic fractures were collected from GP and hospital records within a mean fracture follow-up of 7.59 (SD=2.84) years. Hip DXA scans were analysed with 3D Shaper®. Pearson (partial) correlation was calculated for aBMD, cBMD and tBMD at the total hip, femoral neck and trochanter. Fracture risk (osteoporotic and hip) estimates were determined as hazard ratios (HR) per SD decrease in BMD from Cox-regression. All models were adjusted for age, sex and BMI. Model performance was assessed via Akaike Information Criterion (AIC) and Bayesian Information Criterion (BIC) coefficients.

**Results:** Lower aBMD was associated with higher incidence of osteoporotic (HR=1.41, 95%CI=1.22-1.61) and hip (HR=2.17, 95%CI=1.64-2.86) fracture. Risk estimates for cortical and trabecular parameters measured at the total hip were similar to those of aBMD for osteoporotic (cBMD HR=1.19, 95%CI=1.05-1.35 and tBMD HR=1.45, 95%CI=1.27-1.67) and hip (cBMD HR=1.54, 95%CI=1.22-1.92 and tBMD HR=2.27, 95%CI=1.72-2.94) fractures. Similar estimates were seen at the neck and trochanter sites. AIC and BIC values were similar across all models. High correlation was observed between aBMD, cBMD and tBMD at the distinct hip sites. (all rho>0.74; p<0.001). Inclusion of aBMD, tBMD and cBMD in the same Cox model resulted in high inflation (VIF>5).

**Conclusions:** Partitioning aBMD into trabecular and cortical components can aid interpreting aBMD values in specific individuals within a clinical setting; but the added contribution to assess hip fracture susceptibility in a population-based setting is extremely limited given the high correlation between parameters.

**P181**

**Association between Hip Bone Mineral Density and Mortality Risk after Hip Fracture: a Prospective Cohort Study**

*Yufeng Ge[1], Minghui Yang[1], Xinbao Wu[2], Ling Wang[3]*
*[1]Beijing Jishuitan Hospital- Capital Medical University, Department of Orthopaedics and Traumatology, Beijing, China ; [2]Beijing Jishuitan Hospital- Capital Medical University, Department of Orthopedics and Traumatology, Beijing, China ; [3]Beijing Jishuitan Hospital- Capital Medical University, Department of Radiology, Beijing, China*

**Abstract Text**

**Background:** Despite extensive research on factors influencing mortality post-osteoporotic hip fracture (HF), the role of proximal femoral bone mineral density (BMD) remains underexplored.

**Purpose:** This study investigates how hip BMD, as measured by quantitative computer tomography (QCT), correlates with mortality risk after HF, and whether this relationship varies across different proximal femur regions.

**Methods:** A cohort of 411 elderly HF patients in Beijing, China, was prospectively enrolled and followed up with a median time of 3 years. At baseline, QCT, as routine practice for HF patients, was used to measure areal BMD of the unaffected hip. Areal BMD of the total hip (TH), femoral neck (FN), trochanter (TR), and intertrochanter (IT) were obtained. Other factors like demographic information and perioperative records were also collected as covariates. The primary outcome was postoperative mortality, analyzed using multivariable Cox regression. All the BMD measurements underwent sex-specific Z-score normalization.

**Results:** A total of 394 patients (78.59±7.59 years, 75.4% female) were included in our final analysis, with 86 (82.23±7.00 years, 81.4% female) dead. While initial analysis showed significant associations between mortality and all measured hip BMD areas, TR BMD remained significantly correlated after adjusting for covariates. Compared to the lower TR BMD group, the higher TR BMD group yielded significantly lower death risks (HR 0.21 95% CI 0.05-0.9, *P*=0.036). Higher survival probabilities were observed for higher TH and TR BMD in survival analysis (*P*<0.001).

**Conclusion:** Hip BMD, especially TR BMD determined by QCT, is an independent risk factor for postoperative mortality in HF patients. This finding underscores the value of QCT in opportunistic analysis for immobilized patients, providing actionable insights for personalized interventions to improve outcomes.

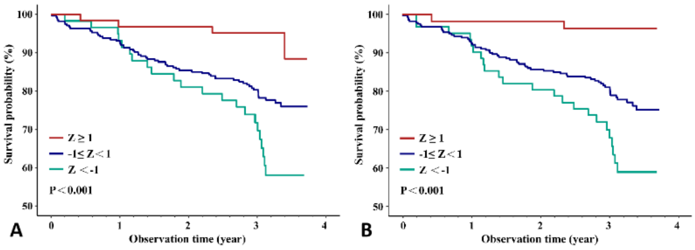

**Fig.** Kaplan–Meier curves for probability of death stratifed by sex-specifc TH BMD and TR BMD.
A: TH aBMD, B: TR aBMD

**P182**

**Comparison of osteoporosis prevalence in people with spinal cord injury and the general population**

*Jisun Lim[1], Chaeun Mun[2], Sumin Nam[2], Onyoo Kim[2]*
*[1]National Rehabilitation Center, National Rehabilitation Reserch Institute, Seoul, Korea- South ; [2]National Rehabilitation Center, Department of Rehabilitation Medicine, Seoul, Korea- South*

**Abstract Text**

**Background:** Spinal cord injury(SCI) is a complex cause of rapid low bone mass, and people with SCI are more easily predisposed to fractures than the general population(GP) due to osteoporosis. However, there is a lack of data regarding the comparison of osteoporosis prevalence in people with SCI and the GP

**Purpose**: This study attempted to understand osteoporosis in people with SCI and to help prevent osteoporosis and treat it by checking the prevalence and diagnosis sites of osteoporosis in people with SCI and the GP, respectively.

**Methods**: The data of 854 SCI patients aged 50 years or above, who underwent the DXA test from 2010 to 2020 at the NRC, were collected. Also, among 5,808 GP in the 2008–2010 KHANES data, 854 individuals with 1:1 matching of gender, age, and BMI to 854 SCI patients were selected. the osteoporosis prevalence and diagnosis sites were compared between the SCI patients and the GP

**Results**: There was no difference between the two groups(p>0.05)(Table1). However, the prevalence of osteoporosis was 39.11%(n=334) for patients with SCI and 19.44%(n=166) for the GP, which was approximately twice as high for patients with SCI as the GP(p<0.05). In addition, the area where osteoporosis was diagnosed was the highest at total hip 45.21% (n=151) for SCI patients, but the highest at Lumbar 40.96% (n=68) for GP(Figure1).

**Conclusion**: The prevalence of osteoporosis in individuals with SCI is higher than in the GP, indicating an elevated risk of fractures within this specific patient group. In addition, the difference in the diagnosis site of osteoporosis between the GP and patients with SCI was confirmed. therefore, to prevent osteoporosis in individuals with SCI, additional studies are needed to consider the characteristics of people with SCI

| | | SCI(n=854) | GP(n=854) | p-value |
|---|---|---|---|---|
| Gender | male | 70.96%(n=606) | 70.96%(n=606) | 1 |
| | female | 29.04%(n=248) | 29.04%(n=248) | |
| age(Mean±SD) | | 62.46±8.52 | 62.49±8.67 | 0.755 |
| BMI(Mean±SD) | | 22.97±3.03 | 22.97±3.09 | 0.985 |
| Osteoporosis | | 39.115%(n=334) | 19.44%(n=166) | <.0001 |

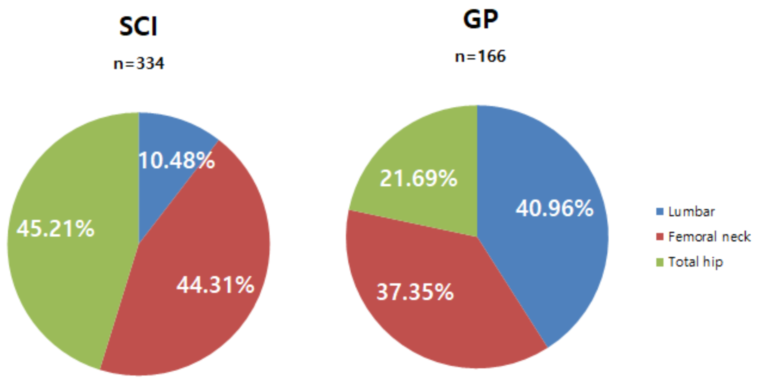

**P183**

**Can "Trabecular Bone Score" predicts bone micro-architecture in patients with end-stage chronic kidney disease?**

*Antoine Bouquegneau[1], Charline Rinkin[2], Laurence Seidel[3], Pierre Delanaye[1], Clio Ribbens[2], Olivier Malaise[2]*

*[1]CHU de Liège, Nephrology, Liège, Belgium ; [2]CHU de Liège, Rheumatology, Liège, Belgium ; [3]CHU de Liège, Biostatistic, Liège, Belgium*

**Abstract Text**

**Introduction:** Trabecular Bone Score (TBS) is a gray level textural parameter, measured from lumbar spine DXA-scans. Our objective is to determine if TBS is an effective tool to estimate bone micro-architecture in patients with end-stage chronic kidney disease, compared to high-resolution peripheral quantitative computed tomography (HR-pQCT) as reference.

**Methods:** 75 patients were included at the time of kidney transplantation. BMD and TBS were analyzed with DXA-scan. Volumetric bone density and bone micro-architecture were analyzed with HR-pQCT (tibia and radius). A second cohort of 61 patients with rheumatoid arthritis (RA) was used as comparator.

**Results:** TBS significantly correlates with volumetric BMD (vBMD) parameters in HR-pQCT (total, trabecular and cortical vBMD (r between 0.240 and 0.390; p<0.03) and trabecular structure (number of trabeculae (TbN) (r between 0.330 and 0.360, p<0.006)). A negative correlation is observed between TBS and separation of the trabeculae (TbSp) and inhomogeneity of the network (Tb 1/NSD) (r between -0.36 and -0.44, p<0.002)), but also with cortical structure at both site (cortical porosity and diameter of cortical pores (r between -0.250 and -0.30, p<0.03)).

BMD also shows a similar correlation with vBMD in HR-pQCT and trabecular structure, with similar correlation coefficients. Unlike TBS, BMD is not associated with cortical porosity parameters. The ultra-distal region of the radius shows stronger associations than the other sites with certain parameters in HR-pQCT (r > 0.70 for total and trabecular vBMD).

In RA cohort, we confirmed that several HR-pQCT cortical parameters were significantly associated with TBS, but not with BMD (Ct vBMD and cortical porosity).

**Conclusion:** Bone microarchitecture (estimated by HR-pQCT) is imperfectly correlated with TBS and BMD (correlation coefficients remain low, even if ultra-distal region of the radius in DXA shows stronger associations). However, TBS also correlated with cortical parameters observed in HR-pQCT demonstrating the microstructural added value of the TBS compared to aBMD at usual site.

**P184**

**Retrospective study shows sustained bone loss in bone sarcoma patients based on analysis of individual bone mineral density trajectories**

*Nicola Baldini[1], Luigi Falzetti[1], Alberto Bazzocchi[2], Fulvia Taddei[3], Enrico Schileo[3], Sofia Avnet[1]*
*[1]University of Bologna, Department of Biomedical and Neuromotor Sciences, Bologna, Italy ; [2]IRCCS Istituto Ortopedico Rizzoli, Diagnostic and Interventional Radiology, Bologna, Italy ; [3]IRCCS Istituto Ortopedico Rizzoli, BIC Bioengineering and Computing Lab, Bologna, Italy*

**Abstract Text**

**Background:** Whilst multiagent chemotherapy provides clear therapeutic benefits for cancer patients, it is also associated with chronic complications in survivors. Osteoporosis in particular affects quality of life, especially in paediatric oncology patients. However, very few studies have described the extent of bone mineral density (BMD) loss in bone sarcoma patients.

**Purpose:** We opportunistically analysed a retrospective case series of primary bone tumours (osteosarcoma and Ewing sarcoma) by retrieving individual patient BMD data at clinical onset, during treatment, and up to 5 years post-therapy. This enabled a thorough investigation of chemotherapy's effects on bone health and identification of patients at higher risk for secondary osteoporosis.

**Methods:** We analysed a retrospective cohort of children and adolescents with primary malignant bone tumours (52 osteosarcomas, 31 Ewing sarcomas), retrieving their BMD at diagnosis and follow-up as Hounsfield units (HU) on routine chest CT scans. Individual BMD trajectories were examined before and after chemotherapy over 5 years using vertebral ROI thresholding.

**Results:** At one-year post-chemotherapy, significant bone loss occurred versus diagnosis (17.6% osteosarcoma, 17.1% Ewing sarcoma). Furthermore, one-year bone loss over 49.2 HU predicted persistent reduced bone mass over the next 4 years, particularly in Ewing sarcoma. At 4 years, only

26% of osteosarcomas and 12.5% of Ewing sarcomas recovered/improved BMD, suggesting increased risk of low BMD-related morbidity.

**Conclusion:** This case series confirms an increased risk of bone loss in bone sarcoma survivors, especially Ewing sarcoma. Studying individual trajectories verifies treatment-induced bone changes per patient, enabling a personalised approach and identification of those patients at risk for early abnormal bone loss and related morbidities such as osteoporosis. Comparing existing CT scans at onset and one year feasibly predicts irreversible bone loss without added cost, radiation, or time and deserves further study, given the potential to significantly impact long-term quality of life for sarcoma survivors.

**P185**

**Influence of photoperiod-induced bone resorption on the bone quality of Djungarian Dwarf Hamsters**

*Praveer Sihota*[1]*, Felix N Schmidt*[1]*, Kathrin Mletzko*[1]*, Johannes Van Kaick*[1]*, Maximilian Neidhardt*[2]*, Alexander Schlaefer*[2]*, Johannes Kurg*[1]*, Ceyda Cubuk*[3]*, Katharina Jähn-Rickert*[1]*, Annika Herwig*[4]*, Björn Busse*[1]
[1]*University Medical Center Hamburg-Eppendorf, Department of Osteology and Biomechanics, Hamburg, Germany ;* [2]*Hamburg University of Technology, Institute of Medical Technology and Intelligent Systems, Hamburg, Germany ;* [3]*University of Hamburg, Institute of Zoology, Hamburg, Germany ;* [4]*Ulm University, Institute of Neurobiology, Ulm, Germany*

**Abstract Text**

**Background and purpose:** Mimicking postmenopausal osteoporosis with high bone fragility in murine animal models is typically induced through invasive surgeries like ovariectomy. However, certain animal species undergo seasonal torpor and experience natural, reversible bone loss, making them potential candidates to study osteoporosis-related bone changes.

**Method**: Fifteen female Djungarian hamsters (Phodopus sungorus) were bred and raised. The hamsters were individually housed with access to water and a hamster breeding diet throughout the experiment. Initially, all animals were kept under long-day conditions (LD; 16:8 h light:dark cycle at 21 °C ± 1 °C ambient temperature) for three months. Subsequently, ten hamsters were transferred to short-day conditions (SD; 8:16 h light:dark cycle at 19 °C ± 1 °C). After 12-14 weeks, all animals were euthanized in their respective housing conditions, and the femora and tibiae were dissected, fixed, and stored for further analysis. The right tibia and femoral midshafts were utilized for microstructural analysis (µCT), the left femur for three-point-bending tests, and the vertebrae and tibia were studied for bone marrow fat cell analysis.

**Result**: The Djungarian hamsters in the short day (SD) group experienced a significant loss of body weight after 14 weeks (LD: 36.08 ± 3.94 g, SD: 25.15 ± 2.68 g; $p \leq 0.001$). The short-day group also exhibited reduced Tb bone volume ($p=0.001$) and Ct.Th ($p=0.009$) resulting in lower mechanical properties such as maximum load ($p=0.013$) and work-to-fracture ($p=0.049$) compared to the summer group (long days, LD). In the short-day group also higher bone marrow fat density is observed ($p=0.041$) at tibial growth plate regions.

**Conclusion**: The study shows that Djungarian hamsters exposed to seasonal day-light variations display bone reorganization resembling human bone loss syndromes as osteoporosis, offering a valuable non-invasive model. This research highlights their potential to explore osteoporosis-related alterations and investigate reversible bone loss mechanisms, potentially aiding human osteoporosis therapies.

**Poster Sessions: Osteoporosis: pathophysiology and epidemiology**

**P186**

**BMSC-derived exosomes protect against ovariectomy-induced bone loss partially through ERK-estrogen receptor α signaling pathway**

*Hui Qi*[1]*, Enpu Shen*[2]*, Danping Liu*[3]*, Cheng'ai Wu*[4]
[1]*Beijing Research Institute of Traumatology and Orthopaedics- Beijing Jishuitan Hospital, Group of Bone Regeneration and Transplantation, Beijing, China ;* [2]*Putuo District Central Hospital, Department of Orthopaedics, Shanghai, China ;* [3]*The First Affiliated Hospital of Jinzhou Medical University, Orthopaedics, Jinzhou, China ;* [4]*Beijing Research Institute of Traumatology and Orthopaedics- Beijing Jishuitan Hospital, Group of Molecular Orthopaedics, Beijing, China*

**Abstract Text**

**Background:** Exosomes derived from BMSCs (BMSC-Exos) are considered to be good choice for osteoporosis (OP) therapy. Estrogen and its receptor are critical in the modulation of bone homeostasis.

**Purpose:** The role of estrogen and/or its receptor in BMSC-Exos treatment of OP still remains unclear. We offered a preliminary view of the mechanism of ERK-Estrogen receptor (ER) α in ovariectomized (OVX) rats treated with BMSC-Exos.

**Methods:** The proliferation, osteogenic differentiation, mineralization, and cell cycle distribution of MG-63 cells with or without BMSC-Exos were examined under CCK8 assay, alkaline phosphatase (ALP) activity and mineralization assay, and low cytometric assay, respectively. Western blot was used for the investigation of the protein expression of ERα and the phosphorylation of ERK. Female SD rats were divided into three groups: sham group, OVX group, and OVX + BMSC-Exos group. Bilateral ovariectomy was performed in the OVX and OVX + BMSC-Exos groups, and PBS or BMSC-Exos were given respectively after 2 weeks of surgery. Similar volume of adipose tissue around the ovary was removed in sham group. Micro-CT scanning and histological staining were used to evaluate the *in vivo* effects of BMSC-Exos.

**Results:** BMSC-Exos significantly enhanced the proliferation, alkaline phosphatase activity, and the Alizarin red S staining in MG-63 cells. The increasing-proportion of cells in G2+S phase and the decreasing-proportion of cells in G1 phase were found after giving BMSC-Exos to MG-63 cells. PD98059 inhibited both the activation of ERK and the expression of ERα, which were promoted by administration of BMSC-Exos. Micro-CT scan showed that in the OVX+BMSC-Exos group, bone mineral density, bone volume/tissue volume fraction, trabecular number were significantly upregulated, and the microstructure of the trabecular bone was preserved compared to those in the OVX group.

**Conclusion:** BMSC-Exos might be proper therapeutic choice in OP, and ERK-ERα signaling pathway play an important role in this process.

**P187**

**Explainable Deep Learning Approaches for Osteoporosis Risk Screening and Individualized risk evaluation using Large Population-Based Data**

*Jin-Woo Kim*[1], *Kim Hyeyeon*[2], *Choi Jongeun*[3]
[1]*Ewha Womans University, Oral and Maxillofacial Surgery, Seoul, Korea- South ;* [2]*Ewha Womans University, Family Medicine, Seoul, Korea- South ;* [3]*Yonsei University, Mechanical Engineering, Seoul, Korea- South*

**Abstract Text**

**Introduction:** For screening osteoporosis, several clinical tools such as FRAX and machine learning models have been attempted, but they presented limitations including low accuracy, limited risk factors and lack individualized explanation. Therefore, this paper presents an interpretable deep learning (DL) model for osteoporosis risk screening with clinical features. Clinical interpretation with individual explanations of feature contributions is provided using an explainable artificial intelligence (XAI) technique.

**Methods:** In this study, we used two separate datasets, namely NHANES and KNHANES datasets from the US and South Korea. DL model for osteoporosis diagnosis was trained on the datasets and significant risk factors were investigated. The performance of DL model was compared with ML models and conventional clinical tools. Additionally, contribution ranking of risk factors and individualized explanation of feature contribution were examined.

**Results:** Our DL model showed AUCs of 0.851 and 0.922 for the femoral neck and total femur bone mineral density, respectively, for the NHANES dataset. The corresponding AUC values for the KNHANES dataset were 0.827 and 0.912, respectively. Significant features were induced and each feature's integrated contribution and interpretation for individual risk were presented. We demonstrate that the developed DL model significantly outperforms conventional machine learning models and clinical tools.

**Conclusion:** Our XAI model produces high-ranked features along with the integrated contributions of each feature, which facilitates the interpretation of individual risk.

**P188**

**Frequency of low bone mineral density in fertile women with systemic sclerosis**

*Natalia Toroptsova*[1], *Olga Dobrovolskaya*[1], *Arina Sorokina*[1], *Nikolay Demin*[1]
[1]*V.A. Nasonova Research Institute of Rheumatology, Department of Osteoporosis, Moscow, Russia C.I.S.*

**Abstract Text**

**Objective:** To determine the frequency and risk factors of low bone mineral density (BMD) in fertile women with systemic sclerosis (SSc).

**Methods:** The study included 64 premenopausal women with SSc (median age 36,0 [31,5; 43,5] years; disease duration 5,0 [2,0; 9,0] years). 54.7% had a limited cutaneous subtype and 45.3% - a diffuse cutaneous subtype without overlaps rheumatic syndromes. 76.6% patients were treated with glucocorticoids (GC) (median duration 5,0 [2,0; 10,0] years). Dual energy X–ray absorptiometry (DXA) was performed to assess BMD. Low BMD was defined as a Z-score < -2.0 SD. An univariate linear regression analysis was conducted to identify factors associated with low BMD. The study was approved by the local ethics committee.

**Results:** 8 (12.5%) premenopausal women with SSc had low BMD. Among women with fragility fractures, only 1 had low BMD, while 4 had normal BMD. Several risk factors associated with low BMD were found out: body mass index (BMI) (OR=0,81; 95%CI 0,67-0,97, p=0,021), duration of SSc (OR=1.14; 95%CI 1.03-1.26, p=0,012), GC cumulative dose (OR=1.04; 95%CI 1.00-1.08, p=0,044) and hand joint contractures (OR=3.58; 95%CI 1.22-13.48, p=0,045). No associations were found between low BMD and age, family history of OP, smoking, treatment with proton pump inhibitors, subtype of SSc, modified Rodnan score, digital ulcer, cutaneous calcification, esophageal hypotension, interstitial lung disease, diffusing capacity of the lung for carbon monoxide, force vital capacity, CRP, erythrocyte sedimentation rate, creatinine, presence of antinuclear antibody, anticentromere antibody, or antitopoisomerase I antibody.

**Conclusion:** 12.5% of premenopausal women with SSc had low BMD. Several risk factors were associated with low BMD including BMI, duration of SSc, GC cumulative dose and hand joint contractures.

**P189**

**Osteoporosis in men with systemic sclerosis**

*Arina Sorokina[1], Natalia Toroptsova[1], Olga Dobrovolskaya[1], Nikolay Demin[1]*
*[1]V.A. Nasonova Research Institute of Rheumatology, Department of Osteoporosis, Moscow, Russia C.I.S.*

**Abstract Text**

**Objective:** to assess the frequency and risk factors of osteoporosis (OP) in men with systemic sclerosis (SSc).

**Methods:** 51 men with SSc without overlap syndromes were included. The median age was 56.0 [45.0; 61.0] years, disease duration – 5.0 [3.0; 7.0] years. 46 (90.2%) patients were treated with glucocorticoids (GC), GC cumulative dose – 8,2 [5,5; 13,7] g. Dual energy X–ray absorptiometry of lumbar spine (L1-L4), femoral neck (FN) and total hip (TH) was done. OP was diagnosed with a T-score ≤-2.5 SD and osteopenia – with a T-score from -1SD to -2.5 SD at any region. Z-score <-2 SD was used for OP diagnosis in men <50 years. Univariate logistic regression analysis was done. The study was approved by the local ethics committee.

**Results:** Low BMD at least in one region of interest was detected in 24 (47.1%) patients: OP – in 10 (19.6%) (7 persons 50 years and older and 3 men <50 years); osteopenia – in 14 (27.5%) men, without difference between the subtypes of the disease (p>0,05). 11 (21.6%) men had a history of low-energy fracture. In a univariate logistic regression analysis body mass index (BMI) [OR=0.72; 95%CI 0.54-0.98, p=0,037] and the low force vital capacity (FVC) was associated with OP [OR=12.53; 95%CI 1.78-88.22, p=0,012]. There was no association between OP and other factors. For OP in L1-L4 associated factor was only BMI [OR=0.66; 95%CI 0.44-0.99, p=0,042], for OP in hip – age [OR=1.19; 95%CI 1.02-1.40, p=0,033], [OR=1.19; 95%CI 1.02-1.40, p=0,033], low FVC [OR=15.55; 95%CI 2.36-19.63, p=0,007] and creatinine clearance [OR=0.97; 95%CI 0.95-0.99, p=0,016].

**Conclusions:** 47.1% of men with SSc had low BMD. There was no significant difference in the frequency of OP depending on the disease subtype. BMI, low FVC and creatinine clearance were associated with OP.

**P190**

**Effects of proline-rich calcium supplement on bone and calcium metabolism and pancreatic biomolecule constituents in spontaneously hypertensive rats**

*Kannikar Wongdee[1], Pornpailin Upanan[1], Kanokwan Kamkajon[2], Wacharaporn Tiyasatkulkovit[3], Narattaphol Charoenphandhu[4]*
*[1]Burapha University, Faculty of Allied Health Sciences, Chonburi, Thailand ; [2]Mahidol University, Faculty of Science- Center of Calcium and Bone Research COCAB, Bangkok, Thailand ; [3]Chulalongkorn University, Faculty of Science- Department of Biology, Bangkok, Thailand ; [4]Mahidol University, Institute of Molecular Biosciences, Bangkok, Thailand*

**Abstract Text**

**Introduction:** Hypertension is a chronic medical condition associated with an increased risk of cardiovascular diseases and impaired bone microstructure. Hypertension was found to deteriorate bone structure and aggravating osteoporosis. Calcium supplement is a common way to alleviate bone calcium loss. Since our previous investigation showed that amino acids–particularly proline–could enhance intestinal calcium absorption, we developed a proline-rich calcium supplement formula to help improve bone structure. Moreover, hypertension often coexists with diabetes; therefore, we hypothesized that hypertension probably associated with changes of biomolecules in exocrine pancreas.

**Methods:** We aimed to study the effects of 3-month calcium supplement (CaS) on bone and calcium metabolism in spontaneously hypertensive rats (SHR). SHR and wild-type (WT) rats were subjected to daily oral gavage with CaS or water for 3 months. Bones were collected and assessed microstructure and strength by µ-CT, and 3-point bending, respectively. Biomolecules in the pancreas were analyzed by synchrotron-radiation Fourier transform infrared spectrophotometry (SR-FTIR). This study has been approved by animal ethics committee of Faculty of Science, Mahidol University.

**Results:** CaS did not alter plasma pH, ionized calcium level, $pO_2$ and $pCO_2$. Regarding bone microstructural analysis, SHR showed less volumetric bone density with marked decreases in cortical and trabecular parameters as compared to WT group. However, 3-month CaS did not alter cortical and trabecular parameters (e.g., cortical and trabecular thickness) or bone mechanical properties in SHR. SR-FTIR analysis revealed that the pancreas of SHR had lower nucleic acid contents and polysaccharides, which might associate with microcirculation disturbance in the pancreas.

**Conclusion:** CaS formula could not rescue bone microstructure in SHR model. It is implicated that long-lasting elevation of arterial blood pressure profoundly deteriorates bone microstructure that is difficult to be rescued simply by oral calcium supplementation. Prevention of hypertension prior to commencement of osteopathy is still the best way to preserve bone health.

**P191**

**Misalignment of collagen fibers and aberrant organic matrix compositions in bones of spontaneously hypertensive rats**

*Narattaphol Charoenphandhu[1], Nattapon Panupinthu[2], Wacharaporn Tiyasatkulkovit[3], Kanokwan Kamkajon[4], Nuntaporn Kamonsutthipaijit[5], Kannikar Wongdee[6]*

[1]Mahidol University, Institute of Molecular Biosciences, Nakhon Pathom, Thailand ; [2]Mahidol University, Faculty of Science- Department of Physiology, Bangkok, Thailand ; [3]Chulalongkorn University, Faculty of Science- Department of Biology, Bangkok, Thailand ; [4]Mahidol University, Faculty of Science- Center of Calcium and Bone Research COCAB, Bangkok, Thailand ; [5]Synchrotron Light Research Institute Public Organization, Beamline 1.3W : SAXS Small Angle X-ray Scattering, Nakhon Ratchasima, Thailand ; [6]Burapha University, Faculty of Allied Health Sciences, Chonburi, Thailand

**Abstract Text**

Hypertension profoundly affects various organs and tissues, such as heart, kidney, blood vessels, etc. It also leads to osteopenia and increased fracture risk, but the underlying cellular and molecular mechanisms remains elusive. Here, long bones of spontaneously hypertensive rats (SHR) with mean arterial pressure of ~150 mmHg were examined for collagen fiber alignment and organic matrix compositions by using synchrotron radiation (SR). Two-dimensional SR small-angle X-ray scattering analysis indicated well organized pattern of collagen fiber alignment in normotensive controls, whereas SHR revealed a random orientation of collagen fiber. SR wide-angle X-ray scattering analysis for bone crystallite dimensions also showed a decreased crystal length in SHR. In the tibial cortical envelope of SHR, several functional groups of organic macromolecules were altered (e.g., Amide I, C-H bending, Amide III protein and C-O-C), as determined by SR Fourier-transform infrared (SR-FTIR) spectroscopy. Further microstructural analysis of the tibiae by using bone histomorphometry showed that osteoid thickness (O.Th), osteoid surface per bone surface (OS/BS) and osteoid volume per bone volume (OV/BV) were markedly decreased in SHR as compared to normotensive controls, suggesting a reduction in unmineralized organic matrix production. An increase in the mRNA level of tissue inhibitor of metalloproteinase (Timp1)—an endogenous protein regulator of the matrix metalloproteinase—was also observed in bone tissues of 36-week-old SHR. In conclusions, hypertension was probably associated with a decrease in osteoid production, misalignment of collagen fibers, and alteration in organic matrix compositions of cortical bone, which might, in turn, negatively affect bone mechanical properties and increase fracture risk. The present study has been approved by Institutional Animal Care and Use Committee of Faculty of Science, Mahidol University, Thailand

**P192**

**Incidence of Vertebral Fractures and 1-year Mortality in COVID-19 era using Korean Nationwide Claims Data**

*<u>Young-Kyun Lee</u>[1], Dong Won Byun[2], Deog-Yoon Kim[3], Youjin Lee[4], Yong-Chan Ha[5]*
*[1]Seoul National University Bundang Hospital, Department of Orthopedic Surgery, Seongnam, Korea-South ; [2]Soonchunhyang University Hospital, Department of Endocrinology, Seoul, Korea- South ; [3]Kyung Hee University Medical Center, Department of Nuclear Medicine, Seoul, Korea- South ; [4]National Cancer Center, Department of Endocrinology, Goyang, Korea- South ; [5]BUMIN Hospital, Department of Orthopedic Surgery, Seoul, Korea- South*

**Abstract Text**

**Background:** Vertebral fractures have been recognized as a major health concern. The outbreak of COVID-19 could affect the incidence of vertebral fracture and mortality in South Korea.

**Purpose:** Our purposes were to evaluate the trends in the incidence and mortality of vertebral fractures and the effects of COVID-19 between 2006 and 2022 in Korea, using nationwide data from the National Health Insurance Service (NHIS).

**Methods:** A nationwide data set was evaluated to identify all new visits to medical institutes for vertebral fractures in men and women aged 50 years or older between 2006 and 2022. The incidence of vertebral fractures and 1-year mortality rates were evaluated.

**Results:** The number of vertebral fractures increased over for the study period. But, the incidence of vertebral fractures reached plateau at 2010 and slightly decreased at 2020 after the time of outbreak of COVID-2019 especially in women. Men and women experienced 17,872 and 66,980 vertebral fractures in 2006 and 43,901 and 136,826 in 2022, respectively. This reflects an increase in the incidence of vertebral fractures for both genders (men, 319.4/100,000 in 2006 and 408.5/100,000 in 2022; women, 1011.1/100,000 in 2006 and 1,155.5/100,000 in 2022). The 1-year mortality rate after vertebral fractures gradually decreased from 6.5% (4,678/84,852) in 2006 to 6.2% (9,845/180,727) in 2022. There was no remarkable change of 1-year mortality during the outbreak of COVID-19. The overall 1-year mortality of vertebral fractures in men was about twice as high women as during the whole study period.

**Conclusion:** The incidence of vertebral fractures reached a plateau in Korea. During the outbreak of COVID-19, incidence of vertebral fractures decreased while the 1-year mortality after vertebral fractures was not affected.

**P193**

**Smoking behaviours in community-dwelling men subjected to dual-X-ray absorptiometry: a cross-sectional study from the SIMON protocol**

*Anita Vergatti*[1], *Veronica Abate*[1], *Lanfranco D'Elia*[1], *Gianpaolo De Filippo*[2], *Ferruccio Galletti*[1], *Domenico Rendina*[1]
[1]*University of Naples- Federico II, Clinical Medicine and Surgery, Naples, Italy ;* [2]*Assistance Publique-Hôpitaux de Paris- Hôpital Robert-Debré, Service d'Endocrinologie et Diabétologie, Paris, France*

**Abstract Text**

**Background:** Active smoke and Environmental Tobacco Smoke (ETS) are cause of death in the world. Osteoporosis (Op) is the most common metabolic bone disorder worldwide, impacting mortality and disability, with higher health and welfare costs.

**Purpose:** Active smoking is a known risk factor for Op and frailty fractures, but few information is available regarding Op and ETS in men.

**Methods:** We performed a cross-sectional study, based on administrative data, to evaluate the association between Op and smoking habits in community-dwelling men, subjected to DXA according to Italian Essential Assistance levels (EALs), and who have completed a questionnaire regarding their smoking habits and their cohabiter's. This study is part of the SIMON protocol.

**Results:** Four hundred sixteen men were selected, and based on questionnaire replies, 167 were classified as current smokers (CS), 93 as passive smokers (PS) and 156 as never smokers (NS). NS showed a lower prevalence of previous frailty fracture and radiological finding of osteoporosis compared to PS and CS ($p<0.05$). The prevalence of vitamin D deficiency was lower in NS compared to both PS and CS (p<0.05). The NS showed a lower prevalence of Op compared to PS and CS, also after correction for age, BMI and eGFR (p<0.05).

**Conclusion:** The study results demonstrate an association between ETS and Op and frailty fractures in community-dwelling men, subjected to DXA according to EALs. This suggests that active smoke and ETS can be considered as equivalent risk factors for Op in men.

**P194**

**Increased risk of osteoporosis, use of osteoporosis medication and major osteoporotic fractures in patients with Addison´s disease in Sweden: a nationwide population-based cohort study.**

*Stavros Stergianos[1,2], Tim Spelman[3], Daniel Eriksson[4,5], Sara Öster[1], Sigridur Björnsdottir[1,2], Olle Kämpe[2,4], Jakob Skov[1,6], Sophie Bensing[1,2]*
*[1]Karolinska Institutet, Department of Molecular Medicine and Surgery, Stockholm, Sweden ; [2]Karolinska University Hospital, Department of Endocrinology, Stockholm, Sweden ; [3]Karolinska Institutet, Department of Clinical Neuroscience, Stockholm, Sweden ; [4]Karolinska Institutet, Department of Medicine Solna- Center for Molecular Medicine, Stockholm, Sweden ; [5]Uppsala University, Department of Immunology- Genetics and Pathology, Uppsala, Sweden ; [6]Karlstad Central Hospital, Department of Medicine, Karlstad, Sweden*

**Abstract Text**

**Background:** There is no solid evidence that autoimmune Addison´s disease (AAD) is associated with increased risk of osteoporosis and osteoporotic fractures.

**Purpose:** To study the risk of osteoporosis and major osteoporotic fractures (MOF) in patients with AAD and examine possible correlation with replacement doses of adrenal hormones.

**Methods:** We linked national health registries and the Swedish Addison Registry and identified 2,056 patients with AAD and 19,042 matched comparators without prevalent osteoporosis at baseline. The primary outcome of the study was a composite of a diagnosis of osteoporosis, MOF or dispensed osteoporosis drugs. The study period was defined from 1st January 2007 until 31st December 2020. We followed individuals at risk from inclusion until censoring or termination of the study.

**Results:** The composite primary outcome was observed in 333 patients with AAD (18.5/1000 person years (PY)), and 1,074 matched comparators (6.4/1000 PY). Patients with AAD were more likely to develop osteoporosis or MOF: aHR 2.72; 95% CI, 2.39-3.10, p<0.001, compared to their matched comparators. The risk of any of the secondary outcomes (the three components of the primary outcome) was significantly higher, including MOF: aHR 1.74; 95% CI, 1.31-2.31, p<0.001.

We did not find any significant rate difference regarding the primary outcome among patients receiving replacement therapy with intermediate or high doses of glucocorticoids compared to those treated with low doses. A reduced risk of osteoporosis or MOF was seen in patients treated with higher doses of mineralocorticoids: aHR 0.70; 95% CI, 0.54-0.90, p=0.006.

**Conclusion:** AAD is associated with increased risk of osteoporosis and major osteoporotic fractures. The glucocorticoid replacement dose does not seem to be the main contributing factor.

**P195**

**Association between frailty and the risk of osteoporotic fracture: The Chungju metabolic disease cohort study**

*Kihyun Baek*[1]*, Jeongmin Lee*[2]*, Jeonghoon Ha*[3]
[1]*Yeouido St. Mary's Hospital- College of Medicine- The Catholic University of Korea, Division of Endocrinology and Metabolism- Department of Internal Medicine, Seoul, Korea- South ;* [2]*Eunpyeong St. Mary's Hospital- College of Medicine- The Catholic University of Korea, Division of Endocrinology and Metabolism- Department of Internal Medicine, Seoul, Korea- South ;* [3]*Seoul St. Mary's Hospital- College of Medicine- The Catholic University of Korea, Division of Endocrinology and Metabolism- Department of Internal Medicine, Seoul, Korea- South*

**Abstract Text**

**Context:** Osteoporotic fractures in older adults, influenced by low bone mineral density (BMD), and frailty from aging have been shown to significantly increase the risk of fractures and other negative health outcomes.

**Objective:** The aim of this study was to investigate the relationship between frailty, BMD and osteoporotic fracture risk among a cohort of community-dwelling older adults.

**Design/Setting/Participants:** This retrospective cohort study used data from a large cohort of rural Koreans in the Chungju Metabolic Disease Cohort study. Frailty was defined using Fried's frailty phenotype, which incorporates five criteria: unintentional weight loss, exhaustion, low physical activity, slow gait speed, and weakness. Fractures were assessed during the medical interview using a structured questionnaire that asked participants about any history of fractures, including the site, cause, and timing of the fracture.

**Results:** Osteoporosis (T-score <-2.5) correlated with a fracture risk, presenting adjusted odds ratios(OR) (95% CI) of 1.89 (1.23-3.47) for any fracture and 1.71 (1.03-3.63) for major osteoporotic fractures (MOF). Frail individuals, especially those classified as frail in univariate analysis, exhibited significantly increased risks for both any fracture (OR 4.13 [2.94-5.80]) and MOF (OR 4.78 [3.32-6.88]). The combination of frailty and BMD further elucidated risks, with frail individuals demonstrating elevated odds ratios (95% CI) across BMD categories. Frailty consistently outperformed BMD in predicting fractures, emphasizing its crucial role in accurate fracture risk assessment in individuals over 50 years old. FI was utilized alongside BMD, demonstrating an improvement in prediction for risk of MOF compared to either score alone (AUC 0.825, P = 0.000).

**Conclusions:** Frailty outperformed BMD in predicting fractures, emphasizing its significance in fracture risk assessment. The combined evaluation of frailty and BMD demonstrated higher risk factor for MOF. These findings underscore the importance of considering frailty with BMD in comprehensive fracture risk assessments in individuals over 50 years old.

**P196**

**Association between sarcopenia, its components, and osteoporosis in the FRISBEE cohort**

*Soulan Tchang*[1]*, Jeroen De Filette*[2]*, Laura Iconaru*[2]*, Amélie Bellanger*[3]*, Alexia Charles*[3]*, Felicia Baleanu*[2]*, Aude Mugisha*[1]*, Murielle Surquin*[1]*, Florence Benoit*[1]*, Pierre Bergmann*[4]*, Jean-Jacques Body*[2]
[1]*CHU Brugmann- Université Libre de Bruxelles, Internal Medicine, Brussels, Belgium ;* [2]*CHU Brugmann-*

*Université Libre de Bruxelles, Endocrinology, Brussels, Belgium ; [3]CHU Brugmann- Université Libre de Bruxelles, Laboratoire de Recherche Translationnelle, Brussels, Belgium ; [4]CHU Brugmann- Université Libre de Bruxelles, Nuclear Medicine, Brussels, Belgium*

**Abstract Text**

**Background:** Bone and muscle disease are both highly prevalent in aging adults but results from previous studies examining the relationship between osteoporosis and sarcopenia are inconsistent. This study evaluated the association between sarcopenia, and its components, with osteoporosis in postmenopausal women.

**Methods:** Cross-sectional data from the Fracture RISk Brussels Epidemiological Enquiry (FRISBEE) cohort, involving 3560 community-dwelling postmenopausal women included between 2007-2013. Randomly selected subjects (n = 500; median age 77.4 years) were reassessed 10 years after inclusion with evaluation of body composition by Dual Xray Absorptiometry (DXA), medical questionnaire and geriatric assessment. Diagnostic criteria for sarcopenia were low muscle mass (<5.5 kg/m$^2$), low muscle strength (<16 kg), and low gait speed (≤0.8 m/s) according to the definition of the European Working Group on Sarcopenia in Older People (EWGSOP2). Osteoporosis was defined by the National Bone Health Alliance Working Group extended criteria, including a history of major osteoporotic fractures and high fracture risk.

**Results:** Among the 500 women, 178 (35.6%) were osteoporotic. Significant correlations were found between sarcopenia components, bone mineral density (BMD) at all sites and trabecular bone score (TBS). The strongest correlations were between handgrip strength and distal forearm BMD (r=0.27; p<0.001) and appendicular lean mass index (ALMI) and total hip BMD (r=0.36; p<0.001). Furthermore, handgrip strength, short physical performance battery (SPPB) test, gait speed, and ALMI were significantly lower in osteoporotic subjects (p<0.001). After adjustment for all covariates, handgrip strength and gait speed remained osteoporosis-associated with odds ratios of 0.92 (0.88-0.97) and 0.33 (0.11-0.96), respectively. Handgrip strength <16 kg showed a 2.2-fold higher osteoporosis risk after adjustment for all covariates.

**Conclusion:** Sarcopenia defined by EWGSOP2 and its components, particularly handgrip strength and gait speed, was significantly associated with osteoporosis. TBS also showed a significant correlation with sarcopenia components and both should be considered when evaluating bone health and fracture risk.

**P197**

**Short- and long-term prognostic models (FRISBEE versus recency-adjusted FRAX®) in the treatment selection of high fracture risk patients**

*<u>Jeroen De Filette</u>[1], Laura Iconaru[1], Alexia Charles[2], Amélie Bellanger[2], Felicia Baleanu[1], Michel Moreau[3], Murielle Surquin[4], Florence Benoit[4], Jean-Jacques Body[1], Pierre Bergmann[5]*
*[1]CHU Brugmann- Université Libre de Bruxelles, Endocrinology, Brussels, Belgium ; [2]CHU Brugmann- Université Libre de Bruxelles, Laboratoire de Recherche Translationnelle, Brussels, Belgium ; [3]Inst. J. Bordet- Université Libre de Bruxelles, Data Centre, Brussels, Belgium ; [4]CHU Brugmann- Université Libre de Bruxelles, Internal Medicine, Brussels, Belgium ; [5]CHU Brugmann- Université Libre de Bruxelles, Nuclear Medicine, Brussels, Belgium*

**Abstract Text**

**Purpose:** The objective of this work was to analyze how patients with a recent fracture would be selected for pharmacological treatment using long-term (uncorrected or recency-adjusted FRAX®) versus short-term prediction models (FRISBEE imminent model for MOFs).

**Methods:** We identified subjects in the FRISBEE cohort who sustained an incident MOF after inclusion (mean age 76.5 ± 6.8 years). We calculated their estimated 10-year fracture risk with FRAX® (before and after recency-adjustment) and 2-year fracture risk with FRISBEE MOFs model.

**Results:** After 6.8 years of follow-up, we validated 480 incident and 54 imminent MOFs. 94.0% of the subjects who had an imminent fracture had a fracture risk estimated >20% by FRAX® before correction for recency and 98.1% after adjustment, with a specificity of 20.2% and 5.9%, respectively. The sensitivity and specificity of the FRISBEE imminent model were 72.2% and 55.4%, respectively, for a threshold of 10%.

For these thresholds, 47.3% of patients were identified as high risk in both models before correction and 17.2% of them had an imminent MOF. The adjustment for recency did not change this selection. Before the correction, 34.2% of patients were selected for treatment by FRAX® only and 18.8% would have had an imminent MOF. This increased to 47% after the adjustment for recency, but only 6% would suffer a MOF within 2 years.

**Conclusion:** In the FRISBEE cohort, the imminent model was less sensitive but more selective in the selection of subjects in whom an imminent fracture should be prevented. Correction for recency in this elderly population decreased the selectivity of FRAX®. Our resultdemonstrate the benefits of using the FRISBEE prediction model for imminent fractures in the selection of subjects at very high fracture risk, who are most in need of immediate treatment but require rigorous patient selection from a global health perspective.

**P198**

**Components of osteoporosis and long-term cardiovascular disease outcomes**

*Cassandra Smith[1], Ryan Teh[2], Marc Sim[1], Jack Dalla Via[1], Abadi K. Gebre[1], Kun Zhu[2], Wai H. Lim[2], Richard L. Prince[2], Gemma Figtree[3], Andrew J.S. Coats[4], John T. Schousboe[5], Joshua R. Lewis[1], William D. Leslie[6]*
*[1]Nutrition & Health Innovation Research Institute- School of Medical and Health Sciences, Edith Cowan University, Perth, Australia ; [2]Medical School, University of Western Australia, Perth, Australia ; [3]Northern Clinical School- Kolling Institute of Medical Research, University of Sydney, Sydney, Australia ; [4]Heart Research Institute, Sydney, New South Whales, Australia ; [5]Park Nicollet Clinic and HealthPartners Institute, HealthPartners, Minneapolis, United States ; [6]Departments of Medicine and Radiology, University of Manitoba, Winnipeg, Canada*

**Abstract Text**

**Background:** Osteoporosis has been linked to increased cardiovascular disease (CVD) risk. However, the long-term risk of CVD outcomes associated with components of osteoporosis (T-scores and osteoporotic fractures) remains unclear.

**Purpose:** We explored whether osteoporosis was associated with increased long-term risk of CVD mortality in 1,117 older women (cohort 1, mean±SD age of 75.0±2.6 years), then performed a replication study in a registry-based cohort of 80,483 people reflective of routine practice (cohort 2, 66.5±9.6 years, 94.7% female).

**Methods:** Bone mineral density (BMD) was assessed by dual-energy x-ray absorptiometry. Osteoporosis defined by a T-score of ≤-2.5 and osteopenia as T-score between -2.5 and -1.0. Mortality/event data were obtained from linked health data. Models were adjusted for CVD risk factors.

**Results:** In cohort 1, 59.7% had osteopenia at any site (total hip or femoral neck) and 27.7% had osteoporosis with 166 deaths (14.9%) over 15-years due to CVD. Compared to having normal BMD, osteoporosis at any site was associated with 92% increased risk for 15-year CVD mortality (**Fig 1a**). In cohort 2, 50.4% had osteopenia at any site (total hip, femoral neck or lumbar spine) and 25.4% had osteoporosis with 20,047 (24.9%) major adverse cardiovascular events (MACE) over 8.5 years. Compared with normal BMD, having osteoporosis at any site was associated with a 17-35% increase MACE and its outcomes, hospitalized acute myocardial infarction, ischemic cerebrovascular disease and all-cause mortality (**Fig 1b**). Regional differences were observed with a site-specific relationship with MACE, the strongest association was observed at the hip.

**Conclusion:** Osteoporosis, defined as low bone mineral density or previous fracture, is associated with higher long-term risk for CVD events. Further work is needed to identify whether this relationship is causal and to identify potential mechanisms. Low BMD may offer a novel strategy to screen for higher risk older women where CVD risk remains underrecognized.

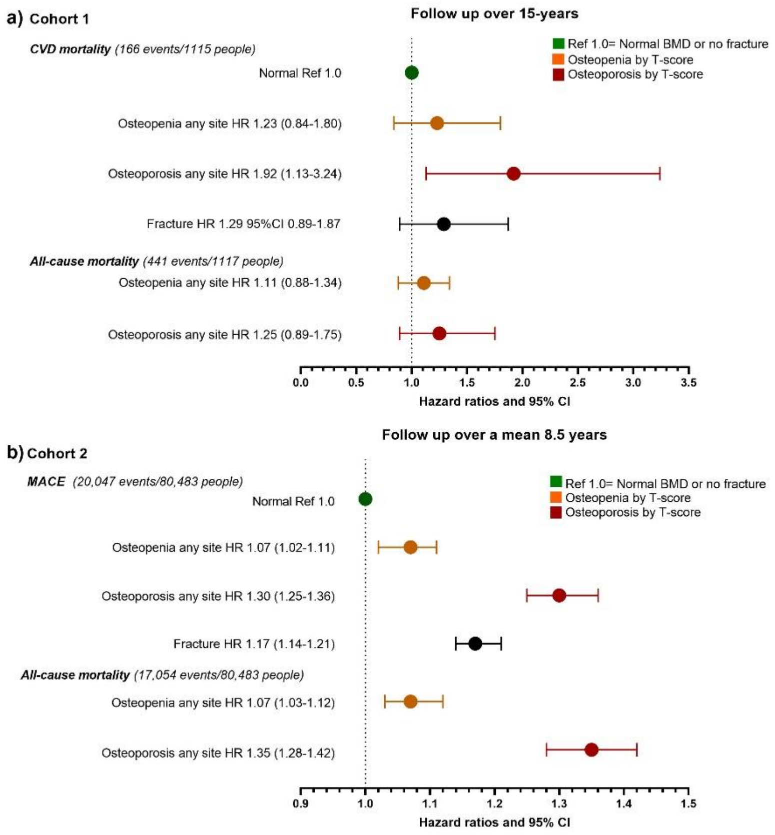

**Figure 1.** Survival analyses on the relationship between components of osteoporosis all-cause and CVD outcomes. Fig 1a multivariable adjusted: age, BMI, treatment [calcium/placebo], smoke, diabetes, antihypertensive, statins or aspirin use and prevalent CVD. Any site includes total hip or femoral neck. Fig 1b multivariable adjusted: age, sex, BMI, smoke, alcohol use, diabetes, hypertension, medication use (e.g., glucocorticoid, cholesterol lowering, anti-hypertensive, anticoagulants, spironolactone, nitrate, diuretics), income[low/high] ethnicity and living status [urban/rural]. Key: any site includes total hip, femoral neck or lumbar spine; MACE, major adverse cardiovascular events.

P199

### Analyzing the onset of osteoporosis and fractures in patients with spinal cord injury (SCI) in South Korea

*Jisun Lim[1], Hyunjin Kim[1], Onyoo Kim[2]*
*[1]National Rehabilitation Center, National Rehabilitation Reserch Institute, Seoul, Korea- South ; [2]National Rehabilitation Center, Department of Rehabilitation Medicine, Seoul, Korea- South*

**Abstract Text**

**Background**: After spinal cord injury(SCI), there is a higher risk of osteoporosis and fractures due to decreased bone density. Therefore, detecting the onset of in patients with SCI is important to prevent fractures osteoporosis and fractures

**Purpose:** This study focused on patients with SCI who were newly diagnosed in 2012, aiming to identify the onset of osteoporosis and fractures in patients with traumatic SCI(TSCI) until 2019 using the NHI data.

**Method:** Data incurred from 2010, to 2019, were used in this retrospective cohort study. We observed patients who sustained a TSCI in 2012 for 7 years to review their healthcare utilization until 2019. The mean and standard deviation of SCI onset to osteoporosis and fracture onset were analyzed using student's t-test and ANOVA.

**Results:** Among patients with TSCI with osteoporosis and fractures, a higher percentage was observed in women than in men(Table1) The mean time from TSCI to osteoporosis onset was 1.7 years. Osteoporosis onset was the longest in those aged 40–49 years (3.0 years). The mean time from TSCI to fracture onset was 2.5 years. The age group with the longest fracture onset was 60–69 years(3.1years). The onset of osteoporosis by gender was shorter in women than in men, but the onset of fractures was shorter in men than in women.(fuirge1)

**Conclusion**: This study was significant in observing patients with TSCI in Korea for 7 years using National Health Insurance Service(NHIS) claims data to identify the onset for osteoporosis and fracture. Further research is needed for the prevention of osteoporosis and fractures in patients with SCI, and the findings of this study will be useful in the treatment, prevention, and management of osteoporosis in patients with SCI in Korea.

| | | TSCI | Osteoporosis | Fracture |
|---|---|---|---|---|
| Age(Mean ± SD) | | 53.99±16.77 | 66.12±11.45 | 60.79±16.36 |
| Sex | Men | 64.17%(n=2,821) | 18.23%(n=72) | 47.5%(n=266) |
| | Women | 35.83%(n=1,575) | 81.77%(n=323) | 52.5%(n=294) |

Figure 1 Time from TSCI to osteoporosis and fracture onset

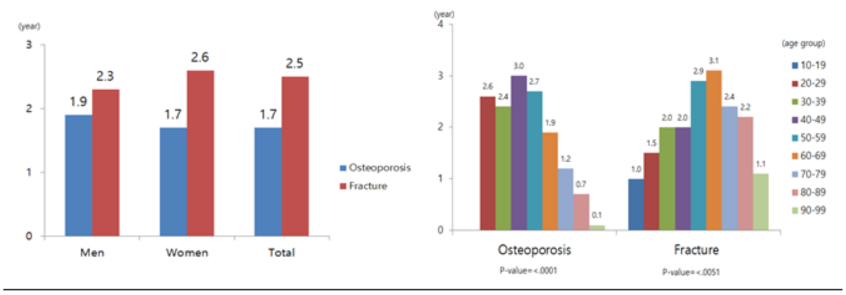

**P200**

**Seasonality of Atypical Femoral Fractures and Classic Hip Fractures – Are AFFs summer fractures?**

*Kasper Westphal Leth[1], Benjamin Bakke Hansen[1], Rick Dell[2], Bo Fan[3], Anne Grethe Jurik[4], Jens Brøndum Frøkjær[5], Mikael Ploug Boesen[6], Christopher D. Smith[7], Eric Vittinghoff[8], Katrine Hass Rubin[1], Douglas C. Bauer[8,9], Dennis M. Black[8], Bo Abrahamsen[7,10]*
*[1]Research Unit OPEN, Department of Clinical Research - University of Southern Denmark and Odense University Hospital, Odense, Denmark ; [2]Kaiser Permanente Southern California, Kaiser Permanente Southern California, Downey, United States ; [3]University of California, Department of Radiology and Biomedical Imaging, San Francisco, United States ; [4]Aarhus University, Department of Radiology, Aarhus, Denmark ; [5]Aalborg University Hospital, Departments of Radiology and Clinical Medicine, Aalborg, Denmark ; [6]Copenhagen University Hospital, Department of Radiology, Bispebjerg and Frederiksberg, Denmark ; [7]University of Southern Denmark, Department of Public Health, Odense, Denmark ; [8]University of California, Department of Epidemiology & Biostatistics, San Francisco, United States ; [9]University of California, Department of Medicine, San Francisco, United States ; [10]Research Unit OPEN, Department of Clinical Research - University of Southern Denmark - Odense University Hospital and Holbæk Hospital, Odense, Denmark*

**Abstract Text**

**Background/Introduction:** Because Atypical Femoral Fractures (AFF) share characteristics of stress fractures we speculated that they should be more prone to occur in summer with more outdoor exercise.

**Purpose:** Examine whether AFF exhibit seasonal patterns different to those of classic hip fractures.

**Methods and study population:** Nationwide descriptive study with radiology review of AFF and ICD codes for classic hip fractures the period 2010 to 2014; all 189 AFF in the country and 691 hip fractures in a 37K random population sample. Women: 623, Men: 257.

**Results:** Seasonality of Atypical Femoral Fractures (AFF) and Classic Hip Fractures is shown in Figure 1 with 95% confidence intervals (CI). For AFF, 54.5% of events were in summer (May to Oct) and for hip fractures this was 52.8% (Difference in proportions: 1.7; 95% CI: -9.692;6.341).

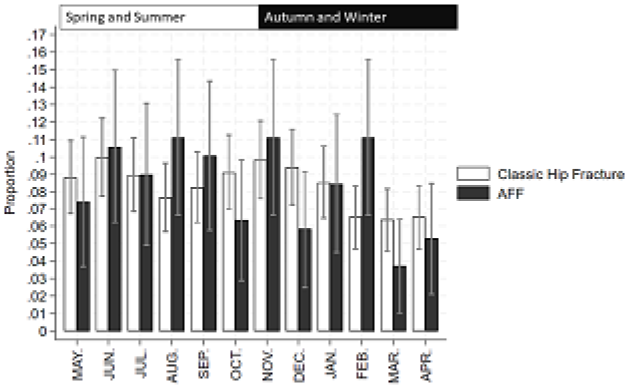

**Conclusions:** AFF in Denmark occurred with the same seasonal patterns as classic hip fractures. While we found that the seasonal patterns were similar, season is not a direct measure of exercise and therefore we cannot exclude the possibility that load-bearing exercise plays a role in development of AFFs.

Funded by the National Institute of Health (US) R01AR082562

**P201**

**Can addition of self-reported clinical risk factors improve prediction of osteoporotic fractures over that achieved from automated analysis of register data using the FREM algorithm?**

*Emilie Rosenfeldt Christensen[1], Frederik Lykke Petersen[1], Sören Möller[1], Tanja Gram Petersen[1], Bo Abrahamsen[2], Katrine Hass Rubin[1]*
*[1]Research Unit OPEN, Department of Clinical Research - University of Southern Denmark and Odense University Hospital, Odense, Denmark ; [2]Research Unit OPEN, Department of Clinical Research - University of Southern Denmark - Odense University Hospital and Holbæk Hospital, Odense, Denmark*

**Abstract Text**

**Introduction:** The Fracture Risk Evaluation Model (FREM) utilizes national register data to estimate an individual's risk of major osteoporotic fractures (MOF). FREM holds the potential to serve as a data-driven decision-support tool for general practitioners, thereby contributing to improve case-finding and treatment of osteoporosis patients.

**Purpose:** To investigate whether adding self-reported clinical data on known risk factors for osteoporosis to the FREM algorithm, improves precision in predicting 1-year osteoporotic fracture risk.

**Methods:** We obtained self-reported data on known risk factors for osteoporosis from women randomly selected to participate in the Danish ROSE (Risk-stratified Osteoporosis Strategy Evaluation) study who answered a questionnaire when invited to participate. Information on the risk factors in FREM's underlying algorithm were obtained through individual record linkage with the national health registers.

We calculated the 1-year predicted risk of MOF using both FREM on its own and FREM combined with clinical factors for all individuals in the cohort and categorized individuals into risk groups (<2%, 2-3%, 3-4%, 4-5% and >5%). Observed MOF incidence in each of these predicted risk groups was compared to predicted risk separately for FREM and for FREM combined with clinical risk factors.

**Results:** Among the 18,605 Danish women included (median age of 70 years), 280 (1.5%) sustained MOFs within one year. Observed incidence of MOF aligned well with predictions from FREM alone and FREM combined with clinical factors for low-risk (<2% and 2-3%) and high-risk (>5%) individuals. However, both models slightly overestimated fracture risk for medium-risk individuals (3-4% and 4-5%).

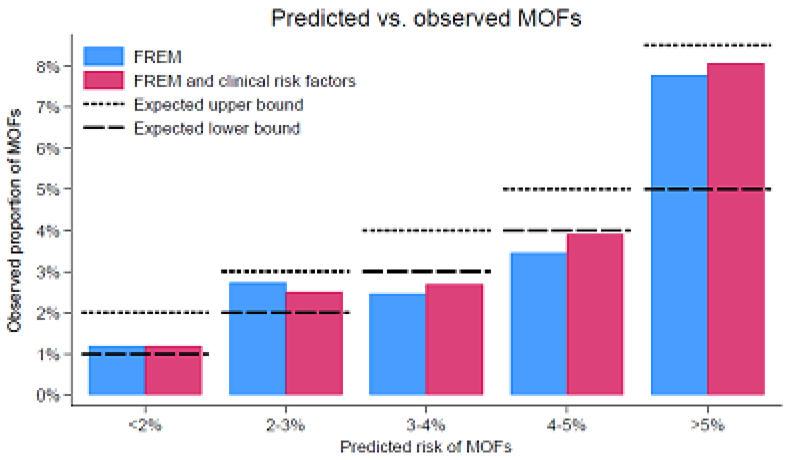

**Conclusion:** Adding self-reported clinical data to the fracture-algorithm, FREM, did not increase precision in identifying individuals at high risk of osteoporotic fractures, since no large differences between the two models were found in the 1-year fracture risk prediction.

**P202**

**Alcohol intake and bone microarchitecture deterioration in older men – the prospective STRAMBO study**

*Pawel Szulc*[1], *Danielle Whittier*[2], *Steven K Boyd*[2], *Roland Chapurlat*[3]
[1]*INSERM UMR1033- University of Lyon- Hospices Civils de Lyon, Epidemiology of Osteoporosis, Lyon, France ;* [2]*McCaig Institute for Bone & Joint Health Alberta Children's Hospital Research Institute- Cumming School of Medicine- University of Calgary, Department of Cell Biology and Anatomy, Calgary, Canada ;* [3]*INSERM UMR1033- University of Lyon- Hospices Civils de Lyon, Rheumatology and Bone Pathology, Lyon, France*

**Abstract Text**

**Background:** Data on the link between alcohol intake and bone microarchitecture are scarce.

**Purpose:** To assess the association between alcohol intake and bone microarchitectural decline prospectively assessed in older men.

**Methods:** In 810 men aged 60-87 years, bone microarchitecture was assessed at the distal radius and distal tibia at baseline, then after 4 and 8 yrs (XtremeCT, SCANCO). Linear mixed models were adjusted for age, BMI, serum bioavailable estradiol, osteoprotegerin, PTH, and C-reactive protein, and for grip strength (radius) or for the leg disability (tibia).

**Results:** Bone microarchitecture decline was the slowest in 263 men drinking 16–109 g alcohol per week (reference group). In 169 men consuming <16 g/week, distal radius trabecular bone mineral density (Tt.BMD) and trabecular number (Tb.N) decreased, whereas intraindividual distribution of trabecular separation (Tb.1/N.SD) increased faster vs. the reference group ($p<0.005$ to $<0.001$). At the distal tibia, total BMD (Tt. BMD), cortical area (Ct.Ar), cortical BMD (Ct.BMD), and failure load decreased faster vs. the reference group ($p<0.05$ to $<0.005$). This pattern may reflect alcohol avoidance and rapid bone decline in men with poor health status. However, the groups did not differ in terms of co-morbidities (except chronic obstructive pulmonary disease, $p<0.05$) or in terms of the treatments (except greater use of benzodiazepines and lower use of fibrates, $p<0.001$). Moreover, the patterns persisted after adjustment for these variables.

In 227 men consuming >219 g/week, distal radius Ct.Ar, Ct.BMD and Tb.N decreased, whereas Tb.1/N.SD increased faster vs. the reference group (all $p<0.05$). Distal tibia Tt.BMD, Ct.Ar, Ct.BMD and failure load decreased more rapidly vs. the reference group ($p<0.05$ to $<0.001$).

**Conclusion:** Moderate alcohol intake (16–109 g/week) may have a protective effect on bone. Higher alcohol intake (>219 g/week) was associated with slightly faster bone microarchitecture decline in older men when assessed prospectively.

**P203**

**Modelling the initial effects of systemic glucocorticoids (GCs) on absolute changes in total left hip BMD in GC treatment-naive and unopposed adults using linear regression**

*Benjamin Bakke Hansen*[1], *Katrine Hass Rubin*[1], *Morten Frost Nielsen*[2], *Pernille Hermann*[2], *Catharina Vind Nielsen*[3], *Bo Abrahamsen*[1,4]
[1]*Research Unit OPEN, Department of Clinical Research - University of Southern Denmark and Odense University Hospital, Odense, Denmark* ; [2]*Odense University Hospital OUH, Department of Endocrinology, Odense, Denmark* ; [3]*Research Unit of Endocrinology, Department of Clinical Research - University of Southern Denmark and Odense University Hospital, Odense, Denmark* ; [4]*Holbæk Hospital, Department of Medicine, Holbæk, Denmark*

**Abstract Text**

**Methods: Setting:** Danish Public Health Service Hospital, catchment area 500,000 individuals.
**Data Source:** BMD data from routine DXA scans in the period 2006-2021 and registry data on filled prescriptions.
**Inclusion Criteria:** All adult patients with at least two DXA exams (left hip), with a minimum of 6 month intervals.
**Exclusion Criteria:** Individuals with prescriptions for systemically acting GCs within five years of first DXA were excluded, allowing a three month grace period. Observations were censored after initiation of anti-osteoporosis agents. DXA scans after the 2$^{nd}$ were excluded for each patient in this model.

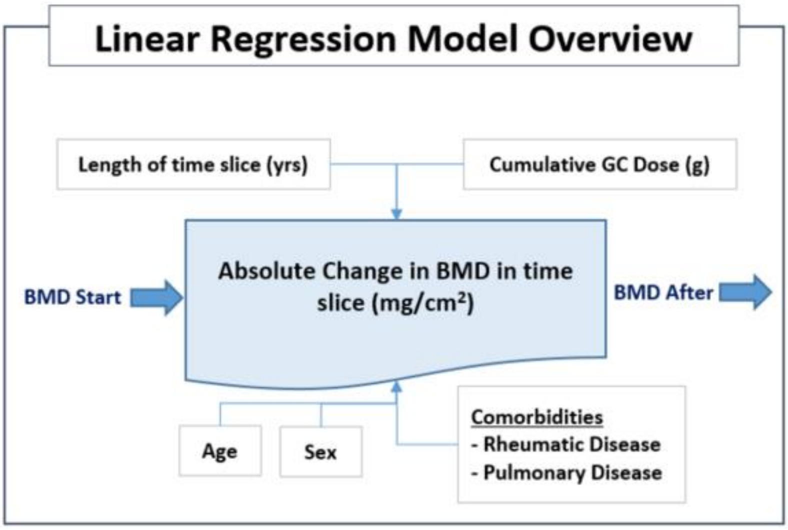

**Results:**

| | GC Exposed Females (N = 1049) | GC Exposed Males (N = 486) |
|---|---|---|
| **Independent Variables** | **Dependent Variable:** *Absolute Change in BMD ( $mg/cm^2$)* | |
| **Total GC Dose (Grams)** | -1.353*** | -1.904*** |
| | (0.436) | (0.539) |
| **Age at first DXA (Years)** | -0.357*** | -0.174 |
| | (0.098) | (0.121) |
| **Time Between Scans (Years)** | -7.793*** | -4.573*** |
| | (0.586) | (0.952) |
| **Rheumatic Disease prior second DXA (Yes/No)** | -2.748 | 1.969 |
| | (3.127) | (3.507) |
| **Pulmonary Disease prior second DXA (Yes/No)** | -0.522 | 2.903 |
| | (3.676) | (5.959) |
| **R-squared** | 0.187 | 0.110 |
| **RMSE** | 40.107 | 35.754 |

***Robust standard errors in parentheses***

***** p < 0.01, ** p < 0.05, * p < 0.1***

Regression analyses reveal a significant linear GC dose response on BMD in both genders, with a greater decrease observed in males than females. Age was a significant factor in females. Time between scans was significant for both genders. Presence of prior rheumatic and pulmonary diseases were not significant on BMD.

The R-squared values indicate that the models explain 18.7% of the variance in BMD changes for females and 11.0% for males, suggesting other factors may also play a role in BMD variation, or that the relationship is non-linear.

**Conclusions:** These findings confirm a significant initial linear dose-response relationship between GC use and BMD loss in unopposed and treatment-naive adults, emphasizing the need for early monitoring in patients undergoing GC therapy.

**P204**

**Zoledronate prevents bone loss in hemiplegic mice**

*Charlotte Logiou[1], Bruno Palmier[2], Morgane Bourmaud[3], Amelie Coudert[3], Isabelle Margaille[2], Nathalie Kubis[2], Martine Cohen-Solal[1], Johann Beaudreuil[4]*
*[1]Inserm U1132 Bioscar, Rehabilitation department- hopital Lariboisiere, Paris, France ; [2]IthEM, pharmacy school, Paris, France ; [3]Inserm U1132 Bioscar, hopital Lariboisiere, Paris, France ; [4]Inserm U1132 Bioscar, Rehabilitation- hopital Lariboisiere, Paris, France*

**Abstract Text**

**Introduction:** Osteoporosis is the most common metabolic bone disease. Its consequence is bone fracture, paralysis and paresis being risk factors. Bone mineral density and micro-architecture are two key of bone strength. Changes in bone micro-architecture (BMA) in paralyzed or paretic patients following stroke are poorly described.

**Objective:** The primary objective was to study bone micro-architecture in paralysis or paresis in a hemiplegic mouse model. The secondary objectives were to study bone mineral density (BMD) and the effect of a bisphosphonate (zoledronate) in the model.

Materials and methods: Sixty-three 10-week-old male C57BL6J mice underwent either transient middle cerebral artery occlusion (tMCAO), either sham operation. They received a subcutaneous injection of zoledronate (Zol, 100 µg/kg) or vehicle alone the day of surgery. BMD (was assessed before and at 28 days, BMA and osteoclast differentiation from bone marrow culture (BMC) were assessed before and at 28 days.

**Results:** At 28 days, tMCAO-induced stroke promoted bone loss at the femur versus sham group (hemiplegic side: DBMD -5±5% vs +3.2±5.6%, p<0.05). In both hemiplegic and non-hemiplegic femur revealed a decrease in Tb.Nb and an increase in Tb.Sp (p<0.05). Moreover, osteoclast number from BMC was higher in stroke mice versus sham mice (hemiplegic side: 22.2±2.3 vs 13.2±1.9, p<0.05). Post-stroke injection of Zol prevented the loss in BMD (hemiplegic side: DBMD +14.3±9.8% vs -5±5%, p<0.0001) and femoral BV/TV (p<0.0001). Osteoclast number was lower in Zol-treated than in vehicle-treated mice (hemiplegic side: 15.6±2.9 vs 22.2±2.3, p<0.05).

**Conclusion:** In the tMCAOt mouse model, stroke induces bone loss at the femur in both hemiplegic and non-hemiplegic sites, which are prevented by zoledronate. These data suggest that Zol could reduce the occurrence of fracture in hemiplegic patients.

**ND-P021**

### Clinical Frailty is Associated with a Previous Hip, Vertebral, and all Fracture plus Type 2 Diabetes: A Cross Sectional Study of a UK Older Population

*Chadi Rakieh*[1], *Shu Ho*[2], *Diane Powell*[1], *Lwin Maung*[2], *Tracey Roberts*[1], *Claire Mennan*[1]
[1]*Robert Jones and Agnes Hunt Orthopaedic Hospital, Metabolic Bone Unit, Oswestry, United Kingdom* ; [2]*Robert Jones and Agnes Hunt Orthopaedic Hospital, Care of the Elderly, Oswestry, United Kingdom*

**Abstract Text**

**Background:** Clinical frailty is associated with multiple adverse health outcomes, including fracture.

**Purpose:** To assess the relationship between clinical frailty and previous hip, spine, wrist, humerus, and all fracture.

**Methods:** A cross-sectional, retrospective, and single-centre study. Consecutive patients aged ≥65 years who attended a bone mineral density (BMD) scan at a UK metabolic bone service in 2023 were included. Frailty was assessed using the Rockwood Frailty Scale (scores of 1-9). Patients were considered frail with scores ≥4. The relationship between frailty and fracture history was assessed using logistic regression (significance level at 0.05).

**Results:** The study included 770 patients: mean age 76.5± 7, 83.9% female, and 51% with previous fracture including 55 (7.1%) hip and 107 (13.9%) vertebral fractures. Compared to the non-frail group, frail patients demonstrated a significantly higher prevalence of all (60.5% vs. 47.2%, p<0.001), hip (17.7% vs. 4.2%, p<0.001), and vertebral (22.8% vs. 14.5%, p = 0.009) fractures but not wrist or humerus fractures (p>0.05, Figure 1). The prevalence of type 2 diabetes was significantly higher in frail patients (13.6% vs. 4.7%; odds ratio (OR) 3.1, 95% confidence interval (95%CI) 1.8-5.4. Frailty was positively associated with current smoking and recurrent falls (≥ 2 within 12 months) and negatively associated with total hip (TH) T-score (p<0.05). In a multivariate logistic regression model, when adjusted for age, BMI, and TH BMD, a statistically significant correlation remained between frailty and hip fracture (OR 4.58, 95%CI 2-10.4, p<0.001) but not other fractures (p>0.05).

**Conclusions:** These results demonstrate significant correlations between clinical frailty and all, vertebral, and, most strongly, hip fractures. The association between frailty, type 2 diabetes, and smoking indicates that patients with frailty should be thoroughly assessed for cardiovascular risk. Prospective studies are needed to explore the predictive value of frailty for future fracture and global health, including cardiovascular disease.

Figure 1. Prevalence of previous fracture: Comparison between frail and non-frail patients

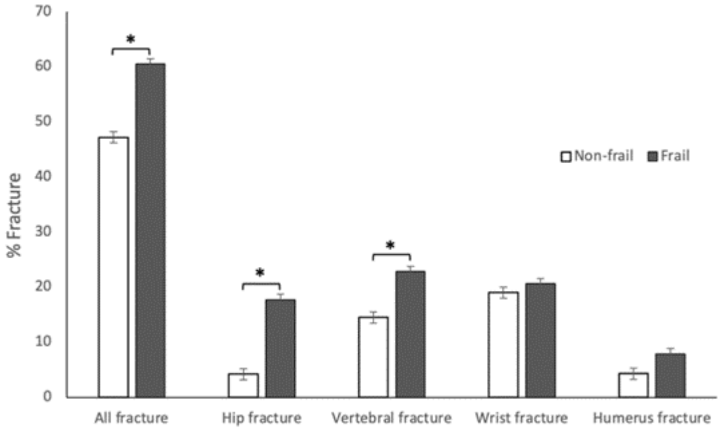

**Poster Sessions: Osteoporosis: treatment**

**P205**

**Analysis of risk factors for compression rate progression in osteoporotic compression fractures**

*Jaewan Soh*[1], *Taejong Jeong*[1]
[1]*Sahmyook Seoul Medical Center, Orthopaedic Surgery, Seoul, Korea- South*

**Abstract Text**

**Background/Introduction:** When OVCF occurs, treatment aims to reduce pain and prevent the progression of compression.

**Purpose:** In osteoporotic vertebral compression fractures(OVCF), vertebral body compression often progresses. We analyzed whether any factors affected the progression of the compression rate and also investigated the period when the compression rate progressed the most.

**Methods:** Among 76 patients visited with OVCF, 61 patients who could be followed up for more than 6 months were included in this study. The associations between age, gender, fractured level, bone mineral density(BMD), body mass index(BMI), previous osteoporosis medication, initial compression rate, pelvic incidence, and the compression rate progressed by more than 30% at the final follow-up compared to the initial compression were analyzed. In addition, compression rates were measured at the initial, 1st month, 2nd month, and 3rd month, and the time period affecting the compression rate progression was analyzed. Statistical univariate analysis was performed with the Chi-square test and multivariate logistic regression analysis was done by using SPSS 25($p<0.05$).

**Results:** In univariate analysis, an increase in compression rate was significantly high in cases that when the initial compression rate was 30% or more($p=0.001$). In multivariate logistic regression analysis, it was analyzed as significant when the initial compression rate was 30% or more($p=0.003$, odd ratio=5.932). Also, with respect to the time period affecting compression rate progression, in univariate analysis, the compression rate at the initial($p=0.001$) and 1st month($p=0.010$) were significant, and in multivariate logistic regression analysis, the compression rate at 1st month was significant(p=0.020, odd ratio=9.226).

**Conclusion(s):** If the initial compression rate was 30% or more, there was a high probability that the compression rate progressed. Also, it was analyzed that the period up to 1st month after the fracture had the most influence on the compression rate progression.

**P206**

**Effect of antiresorptive therapy on development of post-traumatic osteoarthritis after intra-articular distal radius fracture**

*Hyun Sik Gong*[1], *Hwang Ji Sup*[2]
[1]*Seoul National University Bundang Hospital, Orthopedic Surgery, Seongnam, Korea- South* ; [2]*Seoul National University Hospital, Orthopedic Surgery, Seoul, Korea- South*

**Abstract Text**

**Purpose:** Subchondral bone is a potential target of osteoarthritis (OA) prevention. Although anti-resorptive agent (ARA) has been demonstrated to be chondroprotective in surgically induced OA animal models, this has not been studied in humans. This study aimed to determine the effect of ARA therapy on the development of post-traumatic OA after intra-articular distal radius fracture (DRF).

**Methods:** We retrospectively reviewed 299 women (average age of 65 years) who underwent surgery for an intra-articular DRF with follow-up of more than one year. Patients indicated for secondary prevention of osteoporosis started taking ARA immediately after surgery. We evaluated development of radiographic OA at one year postoperatively. Odds ratio (OR) for the OA development was analyzed using logistic regressions for factors including age, lowest T score, bone mineral density, presence of osteoporosis, residual step-off after surgery, and ARA therapy.

**Results:** Development of radiographic OA was identified in 80 (27%) patients. In the regression analysis, radiographic OA was associated with presence of osteoporosis (OR = 3.965), residual step-off (OR = 3.683), and not starting the ARA therapy (OR = 0.289). In subgroup analysis after controlling for osteoporosis, radiographic OA was only associated with not starting the ARA therapy for patients without osteoporosis. However, this association was not present for patients with osteoporosis.

**Conclusion:** Initiation of ARA was associated with a lower incidence of radiographic OA after intra-articular DRF, especially in those without osteoporosis. This study suggests that attempts to decrease bone remodeling might be effective for OA prevention in a joint at risk.

**P208**

**Effectiveness of order sets and alarm services in OCS and EMR for diagnosis and management of osteoporosis in patient with hip fracture**

*Kwangkyoun Kim*[1]
[1]*Konyang university hospital, Orhthopedic surgery, Daejeon, Korea- South*

**Abstract Text**

**Purpose:** This study was aimed to compare the diagnosis, treatment of osteoporosis and refracture between facture liaison service group and no service group.

**Materials and methods:** Authors established the KYUH-FLS service for osteoporosis management in patients with hip fractures from March 2021 to February 2022. We developed order sets, patient education material (YouTube channel), alarm messages in order communication systems, electronic medical record based intervention, and mobile phone messages for KYUH-FLS group. Authors enrolled historic counterparts group (i.e., no KYUH-FLS group) who underwent surgery for osteoporotic hip fracture from March 2018 to February 2019 at the same hospital. The rate of DXA test, initiation rate of anti-osteoporosis medications and persistence of AOM at 6 months and 12 months' follow-up were compared between two groups. Re-fractures were X-ray verified.

**Results:** 154 people were enrolled in the KYUH-FLS group, and 164 people were enrolled in the no KYUH-FLS group. The KYUH-FLS increased the rate of DXA-investigated patients from 36% to 92% ($p < 0.001$), the treatment rate from 34 to 84 % ($p < 0.001$), maintenance of osteoporosis medication at least 6 months and 12 months were increased from 24% to 80% ($p < 0.001$), and 20% to 72% ($p < 0.001$). In the KYUH-FLS group, there were 2 cases of secondary fractures (1.2%) and 3 cases (1.8%) in the no KYUH-FLS group. There was no significant difference in re-fracture rate in the KYUH-FLS group compared to no KYUH-FLS group ($p = 0.81$). Time to secondary fracture was mean 8.7 months in the no KYUH-FLS group (2 cases) and 9.5 months in the KYUH-FLS group (3 cases) ($p=0.78$).

**Conclusion:** KYUH-FLS service increased the number of patients being DXA-examined and treated with AOMs. The KYUH-FLS was not related to reduced secondary fracture rate in hip fracture patients, but this result could have been affected by insufficient statistical power in the analysis.

**P209**

**Romosozumab improves bone mineral density in postmenopausal women with chronic kidney disease-mineral bone disease (CKD-MBD)**

*Chee Kian Chew*[1]
[1]*Tan Tock Seng Hospital, Endocrinology, Singapore, Singapore Rep. of*

**Abstract Text**

**Introduction:** Romosozumab has been shown to be effective and well tolerated in patients with mild to moderate chronic kidney disease (CKD). However, little is known about the use of Romosozumab in patients with chronic kidney disease-mineral bone disease (CKD-MBD).

**Case report:** A 70 years old postmenopausal women with background of type 2 diabetes mellitus, hypertension, hyperlipidemia and stage 4 CKD was referred to Endocrine Clinic for severe osteoporosis. She sustained T8 and L2 vertebral compression fractures which were shown on MRI spine following a fall. DXA showed severe osteoporosis with lumbar spine BMD of 0.668 g/cm$^2$ (T-score -2.9), femoral neck BMD of 0.388 g/cm$^2$ (T-score -3.9) and total hip BMD of 0.559 g/cm$^2$ (T-score -3.1). Her biochemistry showed creatinine 214 umol/L, eGFR 20 ml/min/1.73 m$^2$, adjusted calcium 2.42 mmol/L (2.15-2.55), phosphate 1.3 mmol/L (0.8-1.4), 25-OH-vit D 32 ug/L (>30), PTH 4.2 pmol/L (0.8-6.8) and bone specific alkaline phosphatase (BSAP) 10.2 ug/L. Both her PTH and BSAP levels suggest adynamic bone disease. She was started on monthly SC Romosozumab 210 mg. Her BSAP levels increased to 23.0 ug/L at 1 month and 19.8 ug/L at 6 months of treatment. She tolerated Romosozumab well with no hypocalcemia and didn't sustain any further fracture. At the end of 1-

year treatment, her DXA showed significant improvement with lumbar spine BMD of 0.769 g/cm² (+15.2%), femoral neck BMD of 0.402 g/cm² (+3.6%) and total hip BMD of 0.576 g/cm² (+3.0%).

**Conclusion:** Romosozumab for 12 months is an effective and safe treatment option for patient with CKD-MBD.

**Reference:**

1. Akimitsu Miyauchi et al. J Bone Miner Metab. 40 (4), 677-687, 2022
2. Paul D Miller et al. J Bone Miner Res. 37 (8), 1437–1445, 2022

**P210**

**Establishing consensus recommendations for long-term osteoporosis care for patients who have attended an Australian fracture liaison service: a Delphi study**

*Michael Bennett[1], Jacqueline Center[1], Lin Perry[2]*
*[1]The Garvan Institute of Medical Research & The University of New South Wales, Clinical Studies and Epidemiology Laboratory, Sydney, Australia ; [2]University of Technology Sydney, Faculty of Health, Sydney, Australia*

**Abstract Text**

**Purpose:** Evidence supporting an optimal coordination strategy between fracture liaison services (FLS) and primary care is lacking. This study aimed to develop consensus statements to support consistency and benchmarking of clinical practice to improve coordination of care for patients transitioning from FLS to primary care following an osteoporotic fracture.

**Methods:** A Delphi technique was used to develop consensus among a panel of experts, including: FLS clinicians (medical and non-medical), General Practitioners (GPs), and consumers.

**Results:** Results of a preparatory questionnaire (n=33) informed the development of 34 statements for review by expert panellists over two Delphi rounds (n=25 and n=19, respectively). The majority of participants were from New South Wales (82%), employed as FLS clinicians (78.8%) and working in metropolitan centres (60.6%). Consensus was achieved for 24/34 statements in round one and 8/10 statements in round two. All statements concerning patient education, communication, and the GP-patient relationship achieved consensus. Expert opinions diverged in some areas of clinician roles and responsibilities and long-term monitoring and management recommendations.

**Conclusion:** We found clear consensus among experts in many key areas of FLS integration with primary care. While experts agreed that primary care is the most appropriate setting for long-term osteoporosis care, overall confidence in primary care systems to achieve this was low. The role of (and responsibility for) adherence monitoring in a resource-limited setting remains to be defined. Our consensus statements provide a vision of well-integrated post-fracture care and serve as a useful instrument for policy makers to develop national standards for FLS.

**P211**

**Patient perception on osteoporosis in Korean female patients with osteoporosis**

*Yong-Chan Ha*[1], *Dong-Won Byun*[2], *Deog-Yoon Kim*[3], *Young-Kyun Lee*[4], *Jun-Il Yoo*[5], *Kyung-Hag Lee*[6]
[1]*Seoul Bumin Hospital, Department of Orthopaedic Surgery, Soul, Korea- South ;* [2]*Soonchunhyang University Hospital, Department of Endocrinology- Internal Medicine, Seoul, Korea- South ;* [3]*Kyung Hee University Medical Center, Department of Nuclear Medicine, Seoul, Korea- South ;* [4]*Seoul National University Bundang Hospital, Department of Orthopaedic Surgery, Seongnam, Korea- South ;* [5]*Inha university hospital, Department of Orthopaedic Surgery, Incheon, Korea- South ;* [6]*National Medical Center, Department of Orthopaedic Surgery, Seoul, Korea- South*

**Abstract Text**

**Background:** Patient perception is a key element for the improvement of osteoporosis medication compliance. The aim of this study was to evaluate the awareness, perception, sources of information, and knowledge of osteoporosis in Korean women with osteoporosis.

**Methods:** From 22 July 2021 to 13 August 2021, survey using questionnaire was conducted. Patients who were followed up in endocrinology (Endo), orthopedic surgery (OS), and gynecology (GY) were recruited (40, 40, 20 each) and age of the patients were allocated as follows: 15, 15, and 10 patients in their 60s, 70s, and 80s for Endo and OS, and 10 and 10 patients in their 60$^{th}$ and 70$^{th}$ in GY. Questionnaire was composed of the following topics: Patient Journey to hospital, drug related issues, communication with medical doctors, patient knowledge, and source of information about osteoporosis.

**Results:** Result of medical check-up was the most common reason making patients visit hospital for initial diagnosis of osteoporosis (61%). Patient's perceived severity (87%) and perceived susceptibility (95%) was high but knowledge on behavior modification for prevention was low (71%). Knowledge gap on mortality, re-fracture and drug induced osteoporosis was observed. Doctor was the most preferred and trustful source of information and TV health related show was the second most common source of information. Patients in OS showed lower perceived severity and higher drug discontinuation along with higher proportion of fracture as an initial reason of hospital visit for osteoporosis.

**Conclusion:** Variations in perception according to issues and groups were identified in this study. These should be taken into consideration in patient consultation to improve compliance with osteoporosis treatment.

**P212**

**Twelve-Months Treatment Effects of Romosozumab in Postmenopausal Korean Women with Osteoporosis: A Retrospective Real World Data**

*Jung Yoon Park*[1], *Hyoung Moo Park*[2], *Kim Mee-Ran*[1], *Jae-Yen Song*[1], *Yoon-Jee Jung*[1]
[1]*Seoul St. Mary's Hospital- College of Medicine- The Catholic University of Korea- Seoul- Korea., Department of Obstetrics and Gynecology, Seoul, Korea- South ;* [2]*Grace Women's Hospital - Goyang- Korea., Department of Obstetrics and Gynecology- Menopause Clinic, Goyang, Korea- South*

**Abstract Text**

**Introduction:** This study aimed to analyze the results of 12 months of romosozumab treatment in Korean postmenopausal women diagnosed with osteoporosis.

**Methods:** In this retrospective study, data from 48 osteoporosis patients with T-score of -3.0 or lower were analyzed. The primary outcomes included changes in bone mineral density (BMD) measured by dual-energy X-ray absorptiometry (DEXA) at the lumbar spine, total hip, and femoral neck. Secondary outcomes included changes in bone turnover markers (P1NP and CTX) as well as changes in body composition parameters, including skeletal muscle index (SMI), body mass index (BMI), and visceral adipose tissue (VAT) measured by DEXA.

**Results:** The average age of the patient group was 52.28 years, with 13 individuals concurrently undergoing hormone therapy.After 12 months of romosozumab treatment, significant improvements in BMD were observed at the lumbar spine (14.65%), total hip (4.19%), and femoral neck (6.58%). A greater increase in bone mineral density was observed after romosozumab treatment in the group taking HRT compared to the non-HRT group. P1NP showed a significant increase, with an average of 22.1 ng/mL observed approximately 3 months after the start of the treatment. However, by 12 months, there was a significant decrease, with an average of 10.9 ng/mL reduction compared to the baseline. CTX, after 12 cycles of drug treatment, demonstrated a significant decrease with an average of 10.9 ng/mL compared to the baseline. DEXA analysis of body composition revealed no significant change in SMI after 12 months of treatment, while VAT showed a significant but not substantial decrease.

**Conclusion:** Based on real world data, romosozumab treatment for 12 months significantly improved BMD at the lumbar spine, total hip, and femoral neck in postmenopausal Korean women with osteoporosis. Interestingly, combination therapy with HRT may also have a positive additive effect on increasing bone mineral density in the lumbar spine.

**P213**

**Treatment-related changes in total hip bone mineral density and fracture risk reduction for drugs with different mechanisms of action: the FNIH-ASBMR-SABRE Project**

*Tatiane Vilaca*[1], *Marian Schini*[1], *Austin Thompson*[2], *Eric Vittinghoff*[2], *Li-Yung Lui*[3], *Susan Ewing*[2], *Douglas Bauer*[2,4], *Dennis Black*[2], *Mary Bouxsein*[5,6], *Richard Eastell*[1]
[1]*University Of Sheffield, Division of Clinical Medicine, Sheffield, United Kingdom ;* [2]*University of California, Department of Epidemiology & Biostatistics, San Francisco, United States ;* [3]*California Pacific Medical Center, Research Institute, San Francisco, United States ;* [4]*University of California, Department of Medicine, San Francisco, United States ;* [5]*Beth Israel Deaconess Medical Center, Center for Advanced Orthopedic Studies, Boston, United States ;* [6]*Harvard Medical School, Department of Orthopedic Surgery, Boston, United States*

**Abstract Text**

**Background:** In trial-level analyses, we showed a significant association between treatment-related difference in 12- and 24-month total hip bone mineral density (THBMD) changes and reduced fracture risk.

**Purpose:** We examine whether these associations are affected by the drug's mechanism of action.

**Methods:** We used individual patient data from 22 randomised, placebo-controlled, double-blind trials of osteoporosis medications (17 anti-resorptive, 3 PTH analogues, 1 odanacatib, and 1 romosozumab). For each trial, we calculated the treatment-related difference (active-placebo) in mean percent change in THBMD at 12 and 24 months and the risk reductions for the entire follow-up period. We used logistic regression for radiologic vertebral fractures and Cox regression for all clinical fractures (a combination of non-vertebral and clinical vertebral fractures). We performed linear meta-regression to estimate the study-level association ($r^2$ and 95% confidence interval) between treatment-related differences in THBMD changes and fracture risk reduction, including only the 17 trials of anti-resorptive drugs and across all the trials.

**Results:** We included 122,017 participants. For all time points, the $r^2$ values were similar between anti-resorptive and all mechanisms for vertebral and all clinical fractures (Table). The figure shows similar meta-regression plots.

Table. Comparison of association between treatment-related difference in THBMD percent change and fracture risk reduction for all drugs vs. anti-resorptive only, for 12 and 24 months

| | BMD measurement interval | | | |
|---|---|---|---|---|
| Fracture | 12 months | | 24 months | |
| | All | Anti-resorptive | All | Anti-resorptive |
| Vertebral $r^2$(CI) | 0.59 (0.19, 0.75) | 0.70 (0.23, 0.83) | 0.73 (0.33, 0.84) | 0.78 (0.37, 0.87) |
| All clinical $r^2$(CI) | 0.46 (0.11, 0.65) | 0.51 (0.11, 0.71) | 0.71 (0.32, 0.83) | 0.65 (0.19, 0.80) |

All p<0.002

**Conclusion:** These results show that these associations are robust across anti-osteoporosis therapies with varying mechanisms of action and suggest that the treatment-related mean difference percent THBMD change would predict fracture reduction equally well for drugs with varying mechanisms of action.

**P214**

### 3D Modeling of Hip Dual-Energy X-Ray Absorptiometry (DXA) in post-menopausal women treated with romosozumab

*<u>Giovanni Adami</u>[1], Maurizio Rossini[1], Angelo Fassio[1], Camilla Benini[1], Ombretta Viapiana[1], Davide Gatti[1]*
*[1]University of Verona, Rheumatology Unit, Verona, Italy*

**Abstract Text**

**Background:** Romosozumab, a sclerostin inhibitor, can increase bone mineral density (BMD) faster and to a higher extent than any other anti-osteoporotic drugs available. However, the precise effects on cortical and trabecular bone are not fully clarified.

**Purpose:** To investigate the effect of romosozumab on three-dimensional QTC-like parameters of cortical and trabecular compartments of the proximal femur.

**Material and methods:** We conducted a 6-months prospective observational study on postmenopausal women treated with romosozumab. Image files of the hip DXA scans at baseline and after 6 months of romosozumab were transferred for DXA-based 3D modeling (3D-SHAPER v2.10.1, Galgo Medical, Spain). In brief, the software built a QCT-like 3D proximal femur model for each DXA image, based on a database of QCT scans from a reference population. Integral, cortical, and trabecular vBMD (mg/cm$^3$) were extracted. Other 3D-DXA measurements included: Cortical surface sBMD (mg/cm$^2$), cortical BMC (g), cortical and trabecular volume (cm$^3$), cortical Thickness (mm), cross-sectional area (CSA, $cm^2$), and cross-sectional moment of inertia (CSMI, $cm^4$).

**Results:** 19 post-menopausal women were included in the study. The increase in aBMD was paralleled by a significant increase in most trabecular parameters but not cortical parameters. **Figure 1** shows the 6-month % increase in 3D-DXA parameters and p-values adjusted with the Holm-Šídák method. In synthesis (list not complete): Trabecular vBMD Total (mg/cm3) increased from 74.82 to 83.56 (SE of difference 3,43, p 0.02), integral vBMD Total (mg/cm3) increased from 194.7 to 204.4 (SE of difference 3.79, p 0.02), CSA InterTroch ($cm^2$) increased from 0.97 to 1.03 (SE of difference 0,02, p 0.01) and CSMI InterTroch ($cm^4$) increased from 3.46 to 3.68 (SE of difference 0.09, p 0.02).

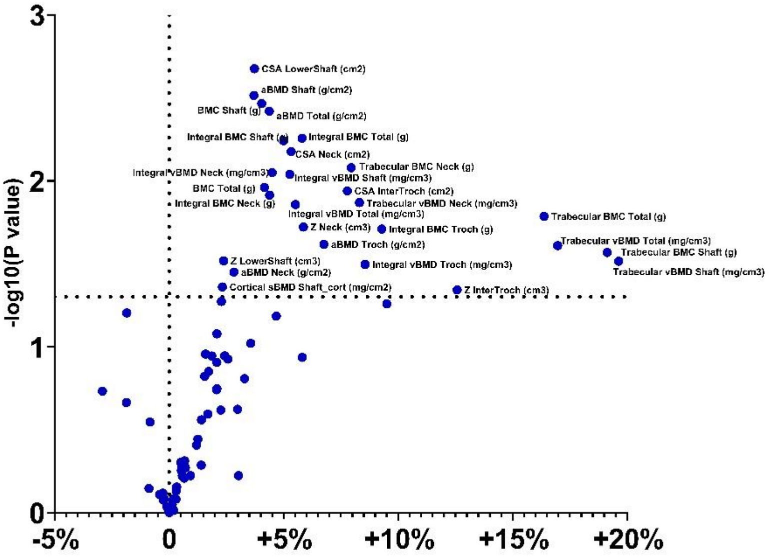

**Conclusions:** Romosozumab increased most trabecular 3D-DXA parameters in 6 months. Cortical parameters did not change over time.

**P215**

**Effect of 2-years monthly calcifediol administration in postmenopausal women with vitamin D insufficiency**

*Marco Occhiuto[1], Salvatore Minisola[2], Cristiana Cipriani[1], Jessica Pepe[1], Luciano Colangelo[1], Marco Lucarelli[3], Luciano Nieddu[4], Viviana De Martino[1]*

*[1]"Sapienza" Rome University, Sciac, Rome, Italy ; [2]"SAPIENZA"- ROME UNIVERSITY, SCIAC, ROME, Italy ; [3]"Sapienza" Rome University, Experimental Medicine, Rome, Italy ; [4]Unint University, Humanistic and Social International Sciences, Rome, Italy*

**Abstract Text**

**Introduction** There are many preparations for correcting vitamin D insufficiency. However, there are no long-term studies (that is, more than 12 months) utilizing calcifediol.

**Purpose** We assessed the long-term (24 months) efficacy and safety of monthly calcifediol administration (0.266 mg) in the correction and maintenance of total 25(OH)D levels in postmenopausal women with basal values less than 30 ng/mL.

**Methods** We initially enrolled 45 consecutive patients during the period September 2019-September 2020. After initial visit, patients were instructed to return at 3,6,9,12 and 24 months for measuring serum total 25(OH)D, ionized calcium, creatinine and isoenzyme of alkaline phosphatase (bALP). Here, we report only the per protocol analysis, because COVID-19 pandemic precluded adherence to the scheduled visits, in some patients. The effect of time on the trend of each single outcome has been assessed using linear mixed effect models for repeated measures.

**Results** The mean age of patients was 62.4±9.0 years. Mean basal values of 25(OH)D levels were 20.5±5.3 ng/mL. There was a continuous increase of mean 25(OH)D values (p for trend <0.001). However, mean values at month 24 (36.7±15.9) were not significantly different in respect to values at month 12 (41.2± 11.18). At the end of the study, only 1 out 19 patients had a value < 20 ng/mL. There was a significant decrease with time of mean values of bALP (p <0.0216) with no significant changes in mean values between 12 and 24 months. No significant changes were observed as far as mean values of ionized calcium or creatinine were concerned. No serum ionized calcium above 1.33 nmol/L, was documented.

**Conclusion** This is the first open label investigation carried out for as long as 2 years with monthly 0.266 mg calcifediol administration. Long-term administration of calcifediol maintain stable and sustained 25(OH)D concentrations with no reported safety concerns.

**P216**

**Novel Pyrimidine derivative (18a) prevents glucocorticoid induced osteoporosis through upregulation of autophagy and PINK1/PARKIN mediated mitophagy**

*Sonu Khanka[1], Krishna Bhan Singh[1], Sumit K Rastogi[2], Kriti Sharma[1], Ravindra Kumar[2], Divya Singh[1]*
*[1]Central Drug Research Institute, Division of Endocrinology, Lucknow, India ; [2]Central Drug Research Institute, Division of Medicinal & Process Chemistry, Lucknow, India*

**Abstract Text**

**Background**: Glucocorticoid-induced osteoporosis (GIOP) is the most common type of secondary osteoporosis. There is an unmet need to identify new drug candidates owing to the serious side effects of current therapeutics. Few reports indicate the role of pyrimidine derivatives in suppressing osteoclastogenesis. However, no report is on their osteogenic potential and role in GIOP.

**Purpose:** To evaluate the potential therapeutic effects and mechanism of action of novel pyrimidine derivative (18a) in GIOP.

**Methods:** *In vitro,* Calvarial osteoblast cells were treated with Dex (100µM) and 18a (100pM), for 24 h and osteoblast differentiation and viability was assessed. Further osteoblast apoptosis, intracellular/mitochondrial ROS generation, mitochondrial membrane potential and mechanisms involved were elucidated. Expression of autophagy and mitophagy markers genes was detected by qRT-PCR and western blot. *In-vivo* studies included Balb/C mice divided in 5 groups: Control, Dex (5mg/kg), Dex+18a (5mg/kg), Dex+18a (10mg/kg), Dex+18a (20mg/kg), Dex+ALN (3mg/kg) positive control. Dosing continued for 1 month. Bones & serum were extracted for *ex-vivo* mineralization, histomorphometry, ELISA and protein expression studies.

**Results:** Compound **18a** restored osteoblast alkaline phosphatase activity and viability by impairing Dex induced apoptosis, ROS generation, and mitochondrial dysfunction. 18a increased the expression of autophagy marker (LC3II), mitophagy markers (PINK1, PARKIN) and decreased expression of

P62. **18a** increased osteogenesis however in presence of 3MA (an autophagy inhibitor), osteoprotective effects of **18a** was partially eliminated. *In vivo*, compound **18a** treatment at 10mg and 20mg dose increased trabecular bone parameters, like, BV/TV, Tb.Th, Tb.N as well as elevated bone biomechanical strength. Notably, **18a** significantly increased P1NP and decreased CTX levels which were altered in Dex group. **18a** promoted autophagy and mitophagy induction.

**Conclusion:** Compound **18a** mitigates the deleterious effect of Dexamethasone by modulating autophagy and PINK/PARKIN mediated mitophagy. Thus, this study advocates the use of pyrimidine derivative (18a) as novel therapeutic option for glucocorticoid induced osteoporosis.

**P217**

**Prolonged treatment with irisin counteracts bone loss in the femurs of aged mice**

*Graziana Colaianni[1], Angela Oranger[1], Roberta Zerlotin[1], Patrizia Pignataro[2], Clelia Suriano[1], Giuseppina Storlino[3], Manuela Dicarlo[1], Giorgio Mori[3], Silvia Colucci[2], Maria Grano[1]*
*[1]University Of Bari, Department of Precision and Regenerative Medicine and Ionian Area - DiMePRe-J, Bari, Italy ; [2]University of Bari, Department of Translational Biomedicine and Neuroscience - DiBraiN, Bari, Italy ; [3]University of Foggia, Department of Clinical and Experimental Medicine, Foggia, Italy*

**Abstract Text**

**Background:** Over the past decade, it has become clear that irisin is a molecular exercise mimetic, thus eliciting beneficial effects on various tissues, including bone. Our previous data showed that 4-week intermittent administration with low dosage of recombinant irisin prevents cortical and trabecular bone loss in a young mouse model of disuse-induced osteoporosis.

**Purpose:** Given the emerging role of irisin as an osteoanabolic agent, in the present study we sought to understand whether the 4-week therapeutic regimen or a more prolonged treatment with irisin might be a possible therapeutic option to counteract age-related bone loss.

**Methods:** Ex-vivo analysis of femurs by microCT and cortical bone by qPCR were used to evaluate the effect of irisin in N= 50 aged C57BL6 mice divided in four groups and treated subcutaneously with vehicle or irisin (100 µg/kg/weekly) for 4 weeks or 8 months.

**Results:** 4-week treatment with irisin increased in cortical bone the gene expression of *Osterix* (*p=0.017*) and *Tfam* (*p=0.025*), transcription factors of osteoblast differentiation and mitochondrial activity, respectively, and reduced *Dkk1* (*p=0.018*), thereby promoting bone anabolism. However, the 4-week treatment was not sufficient to induce a significant increase in bone mass. Interestingly, micro-CT analysis of femurs in mice treated with irisin for 8 months showed an increase in cortical bone mineral density (TMD) (*p=0.008*), bone surface (*p=0.036*), trabecular bone volume fraction (BV/TV) (*p=0.02*) and number of trabeculae (Tb. N) (*p=0.0002*) versus vehicle-mice. Of note, irisin significantly increased fractal dimension (FD) (*p=0.0004*) and reduced the degree of anisotropy (DA) (*p=0.035*) compared with vehicle-mice, thereby indicating an improved microstructural organization of trabecular bone.

**Conclusions:** Our results suggest that therapeutic treatment with irisin, based on low dosage and intermittent administration but for a prolonged time, improves cortical and trabecular bone mass in aged mice, slowing down the skeletal decline caused by aging.

**P218**

**Calf circumference is a predictor of denosumab response in older women with osteoporosis**

*Cecilia Oliveri[1], Adele Di Giovanni[1], Elisa Lupo[1], Federica Bellone[1], Giorgio Basile[1], Nunziata Morabito[1], Antonino Catalano[1]*
*[1]University of Messina, Clinical and Experimental Medicine, Messina, Italy*

**Abstract Text**

Aging is associated with deterioration of muscle and bone, resulting in an increased risk of fragility fractures. The purpose of this study was to analyze the association between muscle mass and strength and response to denosumab treatment in postmenopausal osteoporotic women. Postmenopausal women referred to the Outpatient Clinic for Bone and Mineral Metabolism Disorders of the Department of Clinical and Experimental Medicine of the University Polyclinic of Messina were clinically evaluated. The likelihood of sarcopenia was estimated by administering the SARC-F questionnaire, which analyzes strength, assistance in walking, getting up from a chair, climbing stairs, and falls. Muscle mass was assessed by measuring calf circumference, and muscle performance was measured by hand grip strength with a Jamar dynamometer. Bone mineral density (BMD) measurements were taken at the lumbar spine and femur, and X-rays of the spine were taken to check for vertebral fractures. A total of 130 women (age 70.2 ± 9.4 years) were recruited. At baseline, mean BMD T-score values were -2.6 ± 1.1 SD and -2.3 ± 0.7 SD at the lumbar spine and femoral neck, respectively. Mean calf circumference and grip strength were 31.9 ± 2.9 cm and 22.7 ± 6.7 kg, respectively. The SARC-F score was associated with the 10-year probability of major osteoporotic fracture ($r=0.21$, $p<0.05$). Calf circumference was positively associated with the T-score values of both lumbar spine ($r=0.262$, $p=0.034$)and femur ($r=0.359$, $p=0.004$). After 42 months of treatment, BMD increased significantly by 9.6% at the lumbar spine level and 7.3% at the femur level.At multiple regression analysis, after adjustment for patient comorbidities, fracture risk and treatment duration, femoral neck circumference($\beta=1.76$, SE=0.82, $p=0.03$) and femoral BMD value at baseline ($\beta=-94.19$, SE=26.09, $p=0.0009$)were independently associated with change in femur BMD. In postmenopausal osteoporotic women receiving denosumab, calf circumference was independently and positively associated with treatment response.

**P219**

**Influence of mechanisms of calcium homeostasis on the response of bone to denosumab treatment against postmenopausal osteoporosis**

*Javier Martinez-Reina[1], José Luis Calvo-Gallego[1], Peter Pivonka[2]*
*[1]University of Seville, Mechanical Engineering and Manufacturing, Seville, Spain ; [2]Queensland University of Technology, School of Mechanical- Medical and Process Engineering, Brisbane, Australia*

**Abstract Text**

**Background:** Bone remodelling (BR) models can simulate the response of bone to postmenopausal osteoporosis (PMO) and its treatment with denosumab [1]. In this case, bone mass gain is mainly explained by mineralisation of existing tissue, which is influenced by calcium and phosphorus concentrations in bone marrow, which are regulated at three levels: 1) intestinal absorption, 2) renal filtration and 3) bone resorption. The latter constitutes a mechanism for recovery of calcium accumulated in the bone matrix. These processes are regulated by endocrine system and diet (mainly calcium and vitamin D intake).

**Purpose:** The concurrence of PMO with endocrine and dietary disorders can aggravate the disease and compromise the effectiveness of drug treatments, which justifies the study of the coupling between all processes.

**Methods:** A previously developed model of BR [1] has been coupled with a model of regulation of calcium homeostasis [2]. The coupling between both allowed to analyse the effect of diet and other diseases on the evolution of bone mineral density (BMD) during PMO and the effectiveness of denosumab treatment. Menopause was simulated through an increase in RANKL production and treatment starts 10 years after menopause.

**Results:** The figure shows the normalized evolution of BMD in five cases: 1) PMO treated with denosumab (60Q6 during 2 years, black line), 2) plus an extra calcium intake (red), or 3) plus a vitamin D supplement (blue) and concurrence of PMO with 4) hyperparathyroidism (green) or 5) renal deficiency (cyan), both treated only with denosumab.

**Conclusions:** The concurrence of renal insufficiency or hyperparathyroidism with PMO has a strong impact on BMD and also on the efficacy of denosumab. An extra calcium intake does not contribute to bone mass gain during the treatment, but a vitamin D supplement does have a beneficial effect on the efficacy of denosumab.

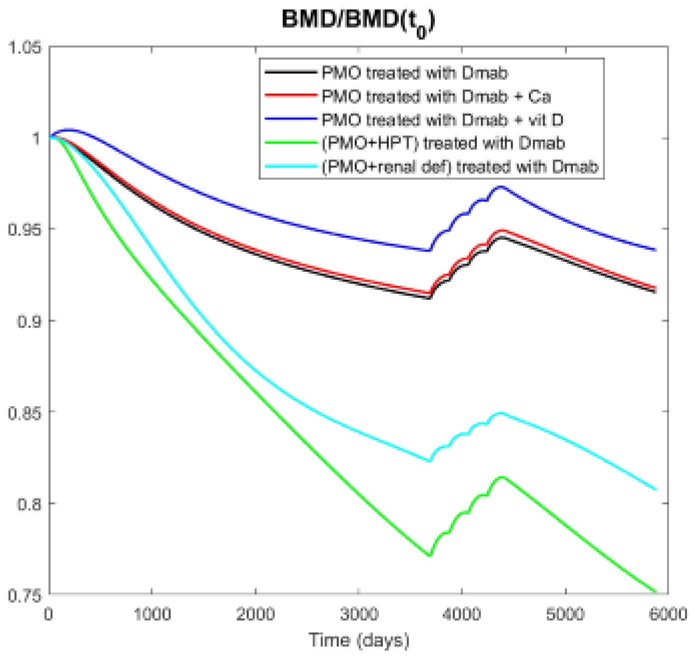

Figure. Temporal evolution of normalized BMD in the five cases analysed.

### P220

### Incident Vertebral Fractures during Romosozumab Treatment in a Patient with a Pathogenic LRP5 variant

*Evert Van Velsen*[1], *Mark Wijnen*[1], *Galied Muradin*[2], *M. Carola Zillikens*[1]
[1]*Erasmus MC, Erasmus MC Bone Center- Department of Internal Medicine, Rotterdam, Netherlands* ; [2]*Erasmus MC, Department of Radiology, Rotterdam, Netherlands*

**Abstract Text**

**Background:** The canonical Wnt-β-catenin pathway plays a significant role in skeletal homeostasis. Defects in this pathway may lead to reduced bone strength and increased fracture risk. Sclerostin is a key inhibitor of this pathway by binding to LDL-receptor-related protein (LRP)-5/6, leading to decreased bone formation. Romosozumab, a human monoclonal antibody that binds to sclerostin and prevents this inhibitory effect, has a strong anabolic and mild antiresorptive effect and is available for patients with severe osteoporosis. However, it is unknown how effective it is as an anabolic agent in patients with inactivating mutations in *LRP5* or *LRP6.*

**Patient Case:** A 69-year old female was seen because of severe osteoporosis (bone mineral density (BMD) of Lumbar spine (LS): T -5.4 SD; Femur neck (FN) [left] T -3.7 SD) with multiple grade 2 vertebral fractures (thoracic (T) and lumbar (L) vertebrae: T5-7 and L5). Except for smoking there were no secondary causes for osteoporosis. Genetic testing revealed a pathogenic heterozygous variant in *LRP5*. Two years earlier she was initially treated for one year with teriparatide because of vertebral fractures (T5-7), which she stopped because of side effects. Within 12 months after stopping, the L5 fracture occurred. She was treated with romosozumab for one year, after which a routine spine X-ray showed five new vertebral fractures, at T11 (grade 3), and L1-4 (grade 2), while BMD had increased at both LS (T -2.6 SD) and FN (T -2.8 SD).

**Conclusions:** This 69-year old female with severe osteoporosis due to a pathogenic heterozygous variant in the *LRP5* gene had several new vertebral fractures during treatment with romosozumab despite a strong increase in BMD. This suggest that romosozumab can increase BMD in patients with a defective *LRP5* gene, but the reason for ongoing vertebral fracture development in this patient is currently unclear.

**P221**

**Denosumab-associated Jaw Osteonecrosis: Descriptive Analysis and Comparison with Zoledronic Acid Using European EudraVigilance Data**

*Sofia Ferreira Azevedo*[1], *Claudia Pinto Oliveira*[1], *Carolina Mazeda*[1], *Inês Cunha*[1], *Anabela Barcelos*[1]
[1]*Centro Hospitalar do Baixo Vouga, Rheumatology department, Aveiro, Portugal*

**Abstract Text**

**Introduction:** Jaw osteonecrosis(JON) is a rare yet serious condition most frequently associated with bisphosphonates, although it can also occur with Denosumab treatment. This study investigated the occurrence of JON as a suspected adverse event(SAE) with Denosumab by analyzing real-world data from the European EudraVigilance database and compared its occurrence proportion with Zoledronic acid.

**Methods:** Retrospective study of EudraVigilance database reports related to Denosumab administration between January 2021 and December 2023. Only adverse events reported by healthcare professionals, occurring in the European Economic Area were included. Duplicated cases or those possibly related to other drugs were excluded. The same process was conducted for Zoledronic acid (Figure 1). Statistical analysis was performed for Denosumab, and Reporting Odds Ratio(ROR) was calculated.

**Results:** A total of 6,675 Denosumab-related SAEs were reported. After inclusion and exclusion criteria, 2,060 reports were considered. Among these, 266 cases concerned JON, (12.9%). Zoledronic acid resulted in 5,986 SAEs, with a final number of 297 JON cases (Figure 1). The risk of JON was lower for Denosumab [(ROR)0.67;CI(-1.037-0.230)].Regarding Denosumab reports, most patients were female (87.2%), aged between 65-85 years (49.6%). The majority recovered/were recovering

(61.1%). The percentage of patients not recovering was higher in elderly groups (>64 years) (43.6%vs.20%;p<0.001). Most patients had severity criteria, although no differences were encountered regarding the presence or absence of severity criteria and the outcome. Similarly, no differences were found for different criteria, except for "disabling," present in 50% of the patients who recovered with sequelae, representing 25% of all patients with this criteria. This percentage was significantly higher when compared to other criteria (p<0.05).

**Conclusion:** Our findings suggest that risk of JON is lower with Denosumab compared to Zoledronic acid treatment. Among SAEs associated with Denosumab, JON represented 12.9% of the total reported cases. This suggests that while significant, it remains an infrequent occurrence in the overall SAE profile of Denosumab.

**P222**

**Comparative analysis of mechanoregulatory trabecular bone adaptations in response to mechanical loading or sclerostin antibody treatment**

*Friederike Schulte*[1], *Julia K. Griesbach*[1], *Francisco C. Marques*[1], *Claudia Weigt*[1], *Marcella Von Salis-Soglio*[1], *Floor M Lambers*[1], *Peter J. Richards*[2,3], *Michaela Kneissel*[2], *Gisela Kuhn*[1], *Ralph Müller*[1]
[1]*ETH Zurich- Institute for Biomechanics, Department of Health Sciences and Technology, Zurich, Switzerland* ; [2]*Musculoskeletal Disease Area, Novartis Institutes for Biomedical Research, Basel, Switzerland* ; [3]*CABMM, University of Zurich, Zurich, Switzerland*
**Abstract Text**

**Introduction:** Among current anabolic osteoporosis therapies, mechanical loading and sclerostin antibodies are promising approaches. Nevertheless, little is known about possible similarities in their bone responses, although this would allow better insights into the yet unexplored mode of action of mechanical loading.

**Purpose:** To compare bone responses of mechanical loading (ML), sclerostin antibody (SclAB) treatment, and vehicle controls (VEH) in terms of morphometric, mechanical, and mechanoregulatory properties.

**Methods:** We evaluated registered and thresholded biweekly time-lapsed vertebral micro-computed tomography scans of C57BL/6J mice, ovariectomized at week 15 and treated from weeks 20 to 24 with ML (n=10), SclAB (n=9), or VEH (n=9). Static and dynamic trabecular bone morphometry, strength, and strain energy density (SED) were compared between groups. Mechanoregulatory analysis linked conditional probabilities of formation, quiescence and resorption to local SED and allowed calculation of remodeling thresholds and amounts of untargeted remodeling.

**Results:** ΔBV/TV increased by 16.0% and 21.3% (ML and SclAB; p=0.911) compared to VEH: -10.2% (p<0.001). ML and SclAB showed equally higher ΔTb.Th (p=0.939) than VEH (p<0.001; p<0.001; Figure 1A). The dual effect of increased mineralizing surfaces and decreased eroded surfaces was observed in ML and SclAB (p=0.984; p=0.878) compared to VEH (p<0.001; p<0.001). Strength increased equally in ML and SclAB (p=0.833) and mean SED decreased equally (-25.8% and -30.8%; p=0.935) compared to VEH (-14.0%; p<0.001; Figure 1B,C). The curves of treatment-induced remodeling probabilities followed each other closely (Figure 1D). Remodeling thresholds and amounts of untargeted remodeling were decreased in ML and SclAB compared to VEH (p<0.05; p<0.05; Figure 1E).

**Conclusion:** Mechanoregulatory similarities in bone responses to ML and SclAB suggest that ML and SclAB are similarly more mechanoregulated than VEH. Furthermore, ML may be considered as effective as SclAB to counteract osteoporotic bone loss and its effects on bone strength, highlighting its potential to be included in tailored treatment plans.

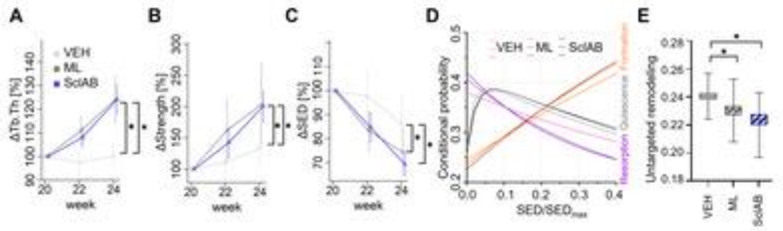

Figure 1: Vehicle (VEH), mechanical loading (ML), and sclerostin antibody treatment (SclAB) in 20-week-old ovariectomized mice over a period of 4 weeks of treatment. A) Change in trabecular thickness (Tb.Th) B) Change in trabecular strength C) Change in trabecular strain energy density (SED) D) Conditional probabilities of trabecular formation, quiescence and resorption in relation to the mechanical signal SED and normalized by SED$_{max}$ (w20-22) E) Amount of trabecular untargeted remodeling (w20-22). *p<0.05

**P223**

**Romosozumab for osteoporosis treatment in patients at very high risk for fracture. A window of opportunity**

*<u>Arantxa Conesa Mateos</u>[1], Bartolome Lladó[2], Ângels Martinez-Ferrer[3], Luis Bernacer[4]*
*[1]Hospital General Universitario de Castellón, Rheumatology, Castellón, Spain ; [2]Hospital Universitario Son Llátzer, Internal Medicine, Palma de Mallorca, Spain ; [3]Hospital Universitario Doctor Peset Valencia, Rheumatology, Valencia, Spain ; [4]Hospital Clínico Universitario de Valencia, Internal Medicine, Valencia, Spain*

**Abstract Text**

**Background:** OP patients with very high fracture risk (VHFR) have a higher risk to suffer FF within two years following the incident fragility fracture (imminent risk) and require an aggressive diagnostic and therapeutic approach

A programme focused in the evaluation of fracture risk and in the sequential treatment (firstly an osteoanabolic and then and antiresorptive agents) in OP patients with VHFR has shown as a more efficient and cost-effective strategy in the management of these patients.

**Objectives:** To define the baseline demographic, clinical, biochemical and densitometric characteristics of patients treated with Romosozumab in real clinical practice

**Methods:** Multicenter Ambispective observational cohort study on OP patients with VHFR in follow-up for FLS or BMU, in treatment with Romosozumab started before October 1, 2023.

Patients with a history of fragility fractures whom initiate romosozumab treatment are included.

Comorbidities, Barthel's index, OP and FF risk factors, bone turnover biomarkers, BMD, FRAX, fractures and previous treatments were analyzed.

**Results:** There were 111 patients included, 96,4% women, mean age 74years(42-93). The 96% were ≥1 recent FF. The 68% had historial FF (1FF (58,3%), ≥2 FF (9,74%)). Calcium 9,3 (7,5-11-5) VitD 24 (4,5-65), PTH 69 (16-267), FRAX MOP 25% (2,8-55) BMD Hip – 2,8 (-5,3 - 0,8). Barthel 89 (40-100).

History of DM (20,3%), AHT (55,5%), CKD 15,3%, neoplasia 22,2%.

From patients with history of previous FF, 51,4% were on antifracture treatment (15 (40,5%) Bbisphosphonates 14 (37,8%) Denosumab, 7 (18,9%) Teriparatide, 1 (2,7%) (SERMs)) and the 48,6% were treatment-naïve patients that initiated Romosozumab therapy.

**Conclusions:** A change in the care model is required that includes the identification and evaluation of fracture risk in OP patients in order to identify those with VHFR and decrease the imminent fracture risk. A paradigm shift focused on patients with VHFR and based on sequential treatment starting with a dual-action osteoanabolic (Romosozumab), provides a new therapeutic option in OP patients with VHFR.

**P224**

**Single-centre real-world experience on bone mineral density outcomes with the use of zoledronic acid in patients with acute and chronic spinal cord injury**

*Shejil Kumar[1], Jean Doyle[1], Roxana Heriseanu[2], Gerard Weber[2], Lianne Nier[3], Roderick Clifton-Bligh[1], Christian Girgis[4]*
*[1]Royal North Shore Hospital, Endocrinology, Sydney, Australia ; [2]Royal Rehab Hospital, Rehabilitation Medicine, Sydney, Australia ; [3]Royal North Shore Hospital, Rehabilitation Medicine, Sydney, Australia ; [4]Westmead Hospital, Endocrinology, Sydney, Australia*

**Abstract Text**

**Introduction**: Traumatic spinal cord injury (SCI) is associated with accelerated sub-lesional bone loss in the first 2-3-years in the knee and hip. Few studies have explored antiresorptive efficacy in preventing bone loss in acute SCI, with no follow up beyond 24-months. Data is even scarcer regarding osteoporosis management in chronic SCI.

**Methods**: A prospective cohort study was performed at Royal North Shore Hospital, Sydney, between 2018-2023. Patients (n=11) with acute traumatic SCI (duration ≤8-12-weeks) received a single baseline zoledronic acid infusion (ZOL) and underwent baseline and 12-monthly DXA BMD scans (including the knee). A retrospective audit was also performed of patients (n=24) with chronic SCI (duration ≥1-year) who had ≥1 dose of ZOL for osteoporosis between 2013-2023 and ≥1 follow-up hip DXA scan.

**Results**: In the acute SCI cohort, median follow-up was 48-months and majority experienced an acute phase reaction post-ZOL (9/11; 82%). Left hip BMD fell by mean 10% at 24-months and 15% at 48-months. Left knee BMD declined by mean 15% at 24-months and 20% at 48-months. In the chronic SCI cohort, mean time since injury was 15-years and patients received median three ZOL infusions. One-third had a fragility fracture prior to ZOL. Only one patient fractured after commencing ZOL. Mean baseline proximal femur and femoral neck BMD and T-score were 0.60 g/cm$^2$ (±0.19), -3.0 (±1.3 SD) and 0.53 g/cm$^2$ (±0.16), -2.8 (±1.8 SD), respectively. BMD improvement 1-year post-ZOL (n=15) was 1.9% (±3.5%, p=0.097) and 3.8% (±4.3%, p=0.005), respectively.

**Discussion**: Early ZOL in acute SCI was unable to prevent rapidly declining hip and knee BMD. Ours is the largest cohort reported receiving ZOL for chronic SCI-related osteoporosis, showing a promising initial response to ZOL. Further controlled studies are needed to establish optimal strategies for preventing early bone loss after acute SCI and minimising fracture risk in chronic SCI.

**Poster Sessions: Other diseases of bone and mineral metabolism**

**P225**

### Unveiling Unique Clinical Phenotypes of Hip Fracture Patients and the Temporal Association with Cardiovascular Events in Hong Kong and the United Kingdom: A Retrospective Study

*Xiaowen Zhang*[1], *Warrington WQ Hsu*[1], *Chor-Wing Sing*[1,2], *Kathryn CB Tan*[3], *Ian CK Wong*[1,2,4,5], *Wallis CY Lau*[1,2,4,5], *Ching-Lung Cheung*[1,2]
[1]*University of Hong Kong, Department of Pharmacology and Pharmacy, Hong Kong, China ;* [2]*Hong Kong Science Park, Laboratory of Data Discovery for Health Limited D24H, Hong Kong, China ;* [3]*University of Hong Kong, Department of Medicine, Hong Kong, China ;* [4]*University College London Hospitals NHS Foundation Trust, Centre for Medicines Optimisation Research and Education, London, Hong Kong ;* [5]*University College London, Research Department of Practice and Policy, London, United Kingdom*

**Abstract Text**

**Introduction:** Cardiovascular events (CVEs) are the leading cause of death among hip fracture patients. The clinical characteristics of hip fracture patients may account for their different prognoses.

**Purpose:** To identify the subphenotypes of hip fracture patients and evaluate their association with incident CVEs in both the Hong Kong and United Kingdom (UK) populations.

**Methods:** A retrospective study was conducted on patients aged ≥65 years with newly diagnosed hip fractures from Hong Kong and the UK. Latent class analysis (LCA) was applied to identify the subphenotypes of hip fracture patients using 22 predefined variables. The prognosis across subphenotypes was assessed using survival analyses, while the prognosis within individuals was evaluated using self-controlled case series (SCCS) analyses.

**Results:** Three distinct clusters were identified in the Hong Kong cohort, each exhibiting unique phenotype profiles. Cluster 1 (n = 11,886; 21.65%) was characterized by patients with cerebrovascular diseases, hypertensive diseases, hyperlipidemia and diabetes, while congestive heart failure was almost exclusively present in Cluster 2 (n = 6,588; 12.00%). Cluster 3 (n = 36,418; 66.34%) comprised relatively healthy patients. Compared with Cluster 3, Cluster 1 and 2 were associated with a higher post-hip fracture risk of major adverse cardiovascular events (MACE), mortality, more unplanned accident and emergency (A&E) visits, and prolonged hospitalization (Figure 1). At the individual level, the SCCS results suggested that the risk of MACE was significantly elevated within 60 days post-hip fracture. Similar associations were observed in the UK cohort (n=27,948).

**Conclusion:** Pre-existing heart failure was identified as a unique subphenotype associated with poor prognosis after hip fractures in both the Hong Kong and UK populations. Our study implied the importance of heterogenous and prompt management of hip fracture patients and prevention of hip fractures among heart failure patients.

Figure 1. The association between hip fracture subphenotypes and outcomes of interest.

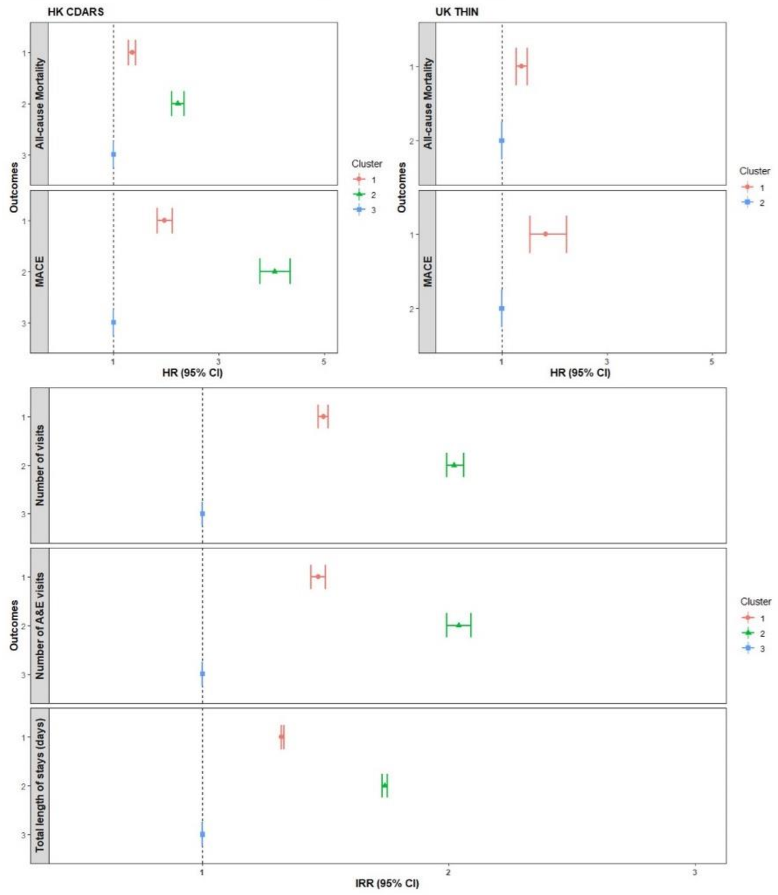

**P226**

**Secular trend in cause-specific mortality in Paget's disease of bone compared with the general population**

*Laetitia Michou[1], Philippe Gamache[2], Isaora Zefania Dialahy[3], Jason R Guertin[4], Jean-Eric Tarride[5], Jacques P Brown[6], Sonia Jean[7]*
*[1]CHU de Québec-Université Laval research centre, Medicine- Université Laval, Quebec, Canada ; [2]Institut national de santé publique, Institut national de santé publique, Quebec, Canada ; [3]Institut national de santé publique, Institut national de santé publique-, Quebec, Canada ; [4]CHU de Québec-Université Laval research centre, Department of preventive and social medicine- Université Laval, Québec, Canada ; [5]Faculty of Health Sciences- McMaster University, Department of Health Research Methods- Evidence and Impact, Hamilton ON, Canada ; [6]CHU de Québec-Université Laval research centre-, Department of Medicine- Université Laval, Québec, Canada ; [7]Institut national de santé publique, Department of Medicine- Université Laval, Québec, Canada*

**Abstract Text**

**Introduction:** Paget's disease of bone (PD) is a chronic, late-onset focal metabolic bone disorder. A decline in the incidence and severity of PD was reported in several countries. We previously reported on an increase in the adjusted all-cause mortality rate in PD from 2015/2016 to 2019/2020.

**Purpose:** We studied the evolution of cause-specific mortality in PD from health administrative databases, comparing them with the general population aged ≥ 55 years.

**Methods:** For each fiscal year, the adjusted cause-specific mortality rate for the main organ systems (e.g., cardiovascular, endocrine, neurological) among people aged ≥ 55 years with PD and in the general population, was determined from health administrative databases from 2000-2001 to 2019-2020. The case definition was based on one hospitalization or two physician services coded for PD (ICD-9: 731.x except 731.2, ICD-10: M88). Standardized mortality rate ratios (SMRR) and 95% confidence interval were calculated. The percentage of cause-specific deaths is described.

**Results:** An increase in SMRR was observed between 2015/2016 and 2019/2020 for diseases of the endocrine system, increasing from 3.73 (1.34-10.4) to 13.1 (6.01-28.6), as well as for diseases of the nervous system, increasing from 0.98 (0.59-1.63) to 2.97 (1.28-6.87). The SMRR has gradually increased for cardiovascular diseases, which have more than doubled in 20 years. In the endocrine system, 76.2% of deaths were due to diabetes. In the nervous system, 60.9% of deaths were related to dementia (including Alzheimer's). The main cause of cardiovascular death was ischemic heart disease (including myocardial infarction) in 50.6% of cases.

**Conclusion:** The increase in mortality from endocrine, cardiovascular and neurological causes may reflect an aging of the PD cohort, as well as the presence of vascular calcifications well-known in PD. The progressive increase in cardiovascular mortality in PD should be further investigated because screening and preventive measures could be offered to patients.

**P227**

**Daily and weekly administration of rhPTH for Medication-Related Osteonecrosis of the Jaw**

*Jin-Woo Kim[1], Kim Sun-Jong[1]*
*[1]Ewha Womans University, Oral and Maxillofacial Surgery, Seoul, Korea- South*

**Abstract Text**

**Introduction:** Medication-related osteonecrosis of the jaw (MRONJ) is uncommon but can result in severe destruction of the jaw. The current study prospectively investigated therapeutic effects of daily or weekly administration of teriparatide in the management MRONJ.

**Methods:** Patients who were diagnosed with MRONJ and consented to teriparatide administration were randomly assigned either to a group of daily-injection or of weekly-injection and completed a four-week course of injection preoperatively and at least eight-week course postoperatively. Control group received either the intraoperative rhBMP treatment (CG_BMP) or no additional perioperative treatment (CG_noBMP). In addition, the patients in teriparatide group received rhBMP treatment. The state of MRONJ was evaluated 2-months (T1) and 6-months (T2) postoperatively for all participants.

**Results:** Either group of daily- (8.35 weeks±1.58;n=17) or weekly-injection (9.17±3.79;n=12) showed significantly faster healing than those of CG_BMP (14.40±6.08;n=25) or CG_noBMP (15.79±9.79;n=39). MRONJ was resolved completely in 24 out of 29 participants who completed the course of teriparatide injections, whereas 46.9% of CG showed delayed resolution. Multiple regression analysis indicated 7.50 times (95% CI, 1.77-31.82) more likelihood of complete resolution of MRONJ for participants with teriparatide injections.

**Conclusion:** A course of daily or weekly administration of teriparatide injections may improve treatment outcomes for patients with MRONJ.

**P228**

**A giant parathyroid lesion with innumerable lytic lesions**

*Muhammad Faiz Muhamad*[1]
[1]*Robert Graves Institute- Tallaght University Hospital- Dublin- Ireland, Department of Endocrinology, Dublin- Ireland, Ireland*

**Abstract Text**

42 year old South-East Asian woman admitted with persistent vomiting on the background of 6 months history of lethargy, generalised aches and visibly large right sided neck mass. She is a non smoker with medical history of hypertension and hyperlipidaemia.

The clinical diagnosis was made at this stage – parathyroid crisis with raised suspicion of parathyroid carcinoma based on the presence of large neck mass and exceedingly high level of PTH(30 times upper limit).

| | |
|---|---|
| Corrected Calcium | 3.16mmol/L |
| Phosphate | 0.91mmol/L |
| ALP | 1338mmol/L |
| Vitamin D | 31nmol/L |
| PTH | 1926pg/ml |

The Technetium-99 Sestamibi scan coupled with SPECT/CT neck confirmed the initial finding on the ultrasound of neck as it revealed intense uptake in the right neck mass which representing a giant parathyroid lesion. The whole nuclear body scan showed uniformly increased uptake affecting the entire skeleton, characterised as a *superscan* as there was diminished uptake by the kidneys. The SPECT-CT scan of pelvis confirmed the presence of innumerable lytic lesions consistent with Osteitis Fibrosa Cystica.

The initial management included rigorous IV fluid, followed by IV bisphosphonates. A 44.9 grams measuring 6cm right parathyroid lesion was resected. Her postoperative course was complicated by development of hungry bone syndrome with prolonged HDU stay.The histology analysis reported a well circumscribed encapsulated lesion with no lymphovascular or perineural invasion or necrosis, suggestive of parathyroid adenoma. Additional results include negative genetic analysis, elevated CTX-1, osteocalcin, P1NP.

This case illustrates a severe skeletal manifestation of hyperparathyroidism caused by a large autonomous parathyroid lesion with underlying chronic vitamin D deficiency. WHO in 2022 listed atypical parathyroid tumour as the third classification, to reflect its unknown potential of malignancy[1]. This lady is under close observation due to concern of possible recurrence.

[1] Lori A. Erickson, O. Mete et al. Overview of the 2022 WHO Classification of Parathyroid Tumours. Endocrine Pathology (2022) 33:64-89

**P229**

**Combination of cannabidiol and Taurine synergistically treat periodontitis in ligature-induced rats**

*Yunjo Soh*[1], *Sewoong Kim*[2], *Badmaarag-Altai Chuluunbaatar*[2]
[1]*Jeonbuk National University- School of Pharmacy, Pharmacology, Jeonju-si-Jeollabuk-do-, Korea-South ;* [2]*Jeonbuk National University, School of Pharmacy, Deokjin-gu- Jeonju-si, Korea- South*

**Abstract Text**

**Background:** The active component in cannabis, cannabidiol (CBD), was first isolated from the hemp plant in 1940. The fundamental mechanisms of CBD's effects on periodontal inflammation have yet to be fully understood. The amino sulfonic acid taurine is a substance that naturally exists in the body and is an inhibitory modulator of inflammation. This study intended to examine therapeutic effects of the combination of CBD and taurine on periodontitis.

**Methods:** To assess the expression of inflammatory markers of INOS, COX-2, TNF-, IL-1, TRAP and resorbed pit areas, CBD and taurine were applied to RAW 264.7 cells. Total 36 SD rats were divided to 4 groups: Sham (healthy), vehicle (induced only periodontitis), L-CBD + taurine (low dose CBD), and H-CBD + taurine (high dose CBD). Rats in all groups except the healthy group received *P. gingivalis* ligatures for seven days, followed by daily treatment with specific drug regimens for 14 days. Upon completion of treatment, the depth of each probing pocket was measured. Rat tooth were obtained and subjected to histomorphometric studies.

**Results:** The combination of CBD and taurine synergistically decreased the expression of inflammatory markers TNF and IL-1 and the amount of TRAP+ cells and resorbed pit areas. The levels of alveolar bone resorption and periodontal pocket depth in rats with *P. gingivalis*–induced periodontitis dramatically decreased after combined CBD and taurine treatment. Periodontal pocket depth was significantly reduced, and bone mineral density (BMD) increased after treatment with a combination of L-CBD, and H-CBD with taurine.

**Conclusion:** A combination of CBD and taurine may be useful as a novel therapeutic agent against periodontal disease since it decreases inflammation indicators and bone resorption activity with reduced alveolar bone loss and periodontal inflammation

**P230**

### Impairment of platelet function and high atherothrombotic risk in both hypoparathyroidism and primary hyperparathyroidism

*Anda Mihaela Naciu*[1], *Andrea Palermo*[1], *Annunziata Nusca*[2], *Alessandra D'Amico*[3], *Alfonso Maria Di Tommaso*[4], *Giulia Sterpetti*[4], *Roberto Carnevale*[5], *Sebastiano Sciaretta*[5], *Francesco Grigioni*[2], *Gaia Tabacco*[1]
[1]*Fondazione Policlinico Universitario Campus Bio-Medico, Unit of Metabolic bone and thyroid disorders, Rome, Italy ;* [2]*Fondazione Policlinico Universitario Campus Bio-Medico, Unit of Cardiac Sciences, Rome, Italy ;* [3]*University of Rome "Foro Italico" Italy, Department of Movement- Human and Health Sciences, Rome, Italy ;* [4]*Campus Bio-medico University of Rome, Endocrinology and diabetes, Rome, Italy ;* [5]*Sapienza University of Rome- Latina, Department of Medical and Surgical Sciences and Biotechnologies, Latina, Italy*

**Abstract Text**

**Background**: there is an increasing body of evidence that parathyroid hormone (PTH) affects functional and structural properties of arteries. Human platelets express PTH1R that seems to mediate a pro-aggregating effect of PTH through a mechanism involving ERK activation. All these findings may explain the endothelial dysfunction/impaired platelet reactivity in primary Hyperparathyroidism (PHPT). Furthermore, also patients with chronic post-surgical hypoparathyroidism (hypoPT) have higher incidences of arterial stiffness and increased risk of mortality. Up to now, no studies have investigated the impact of chronic lack of PTH on endothelial cell activation/dysfunction and platelet reactivity.

**Aim**: to assess platelet reactivity, oxidative stress, endothelial and atherosclerotic predictors in subjects affected by hypoPT compared with PHPT and controls.

**Methods**: in a monocentric, cross-sectional study we enrolled hypoPT patients, PHPT subjects and age-matched controls. All patients underwent a biochemical examination including calcium-phosphorus metabolism and platelet aggregometry tests. Moreover, we evaluated brachial artery endothelial function (flow-mediated dilation-FMD), common carotid intima-media thickness (ccIMT) with ultrasound.

**Results**: 120 subjects (40 hypoPT, 40 PHPT and 40 controls) have been consecutively enrolled. All study groups presented similar age, BMI and TSH. HypoPT and PHPT had impaired oxidative stress parameters than controls [H2O2: 86.1(55.3-109.5); 99.5(61.7-128.4) vs 42.7(30.9-59.0) uM, $p<0.001$; sNOX2-dp: 36.6 (20.4-43.0); 41.2(31.7-66.5) vs 15.3(11.5-21.2) pg/mL, $p<0.001$). In both HypoPT and PHPT vs controls, platelet reactivity was significantly impaired (platelet aggregation%: 44.0(16.3-74); 72.5(57.0-85.0) vs 10.00(4.3-56.0), $p<0.05$; sPSELECTIN: 10.5(7.5-12.5); 19.4 (15.5-22.2) vs 2.3(1.8-4.6) ng/mL, $p<0.001$). Both, hypoPT and PHPT presented endothelial dysfunction compared with controls (VCAM: 390 (340-438); 420(368-494) vs 350(293-390) ng/mL, $p<0.001$; ICAM: 81.7(75.7-96.4); 93.5(77.4-108.4) vs 70.3(50.6-87.4), $p<0.05$). HypoPT and PHPT showed higher ccIMT ($p<0.01$) and lower FMD ($p<0.001$) than controls.

**Conclusion**: our findings suggest that mainly PHPT but also hypoPT have an impaired platelet reactivity, an increased atherothrombotic risk and needs adequate cardiovascular evaluation. Further comprehensive and prospective studies are needed.

**P231**

**Identification of genetic and molecular causes of hypophosphatasia**

*Timur Yorgan*[1], *Philip Wiedemann*[1], *Michael Amling*[1], *Thorsten Schinke*[1], *Florian Barvencik*[1]
[1]*University Medical Center Hamburg-Eppendorf, Department of Osteology and Biomechanics, Hamburg, Germany*

**Abstract Text**

**Background:** Hypophosphatasia (HPP) is a metabolic disorder with detrimental skeletal effects including osteomalacia, caused by decreased activity of the tissue non-specific alkaline phosphatase (TNSALP/*ALPL*). While enzyme replacement therapy is available for childhood-onset HPP, no adequate treatment has been established for moderate and mild cases.

**Purpose:** Investigating genetic variants causing HPP, modeling and modulating the residual activity of TNSALP and establishing a corresponding animal model.

**Methods:** Database evaluation, investigating TNSALP activity in MC3T3-E1 cells, skeletal phenotyping of a HPP mouse model.

**Results:** Currently, a potentially disease causing variant of *ALPL* has been identified in 80 % of our cohort of more than 230 patients with adult-onset HPP. The most common variant is E191K (22 cases ~ 9,5 %), and functional assays revealed a 95.6 % (±0.6 %, p<0.001) decrease of enzymatic activity compared to intact *ALPL*. Interestingly, the residual activity could be enhanced by 550 % (±48 %, p<0.001) through the addition of 1 mM $MgCl_2$. To explore this $Mg^{2+}$-dependent effect *in vivo*, we have generated a mouse line harboring the E191K variant of TNSALP. Analysis of 12-week-old male and female animals revealed residual serum ALP activity of only 13 % (±5.7 %, p<0.001) in homozygous animals and increased osteoid content (1.43±0.16 % vs. 0.81±0.31 %, p=0.007).

**Conclusions:** The residual activity of the E191K TNSALP variant can be increased by $Mg^{2+}$ *in vitro*. The established mouse line models HPP caused by the E191K variant and provides a platform for studying novel therapeutic approaches including nutritional micronutrient manipulation. Interestingly, 20 % of the patients in our cohort show symptoms and serum profiles typical for HPP, although no potentially disease causing variants of *ALPL* were identified. Here we are currently applying extended genetic analysis to identify alternative genetic causes for HPP and potentially expand the genetic landscape of HPP mechanisms and diagnostics.

**P232**

**Hypercalcemia due to CYP24A1 loss of function mutations. Report of four new cases**

*Symeon Tournis[1], Anna Papadopoulou[2], Etienne Cavalier[3], Konstantinos Stylianou[4], Michael Krikelis[1], George Trovas[1], Stathis Tsiakas[5], Stavroula Paschou[6], Konstantinos Makris[7], Efstathios Chronopoulos[1]*
*[1]National & Kapodistrian University of Athens, Laboratory for Research of Musculoskeletal System, Athens, Greece ; [2]National & Kapodistrian University of Athens, Laboratory of Clinical Biochemistry-University General Hospital "Attikon"- Medical School, Athens, Greece ; [3]University of Liège, Department of Clinical Chemistry, Liege, Belgium ; [4]University Hospital of Heraklion, Nephrology Department, Heraklion, Greece ; [5]Laiko General Hospital, Department of Nephrology and Renal Transplantation, Athens, Greece ; [6]National & Kapodistrian University of Athens, Endocrine Unit and Diabetes Center- Department of Clinical Therapeutics- Alexandra Hospital, Athens, Greece ; [7]KAT Hospital- Athens, Department of Biochemistry, Athens, Greece*

**Abstract Text**

**Introduction:** Loss of function mutations in CYP24A1, a disorder called infantile hypercalcemia type 1 (HCINF1), is characterized by PTH- independent hypercalcemia and hypercalciuria due to defective inactivation of $1,25(OH)_2D$.

**Methods:** We report the clinical and biochemical phenotype of four new unrelated cases with HCINF1. In one case, we also report the short-term response to rifampin.

**Results:** Four adult male patients (age: 19-57 yrs.) with long-term PTH-independent hypercalcemia and nephrocalcinosis were referred to our department for further evaluation (table 1). All patients had mild to moderate hypercalcemia, suppressed PTH levels, with the exception of one case with concomitant CKD (stage 3b), normal 25(OH)D, inappropriately (high) normal $1,25(OH)_2D$ levels and frank hypercalciuria. All cases had undetectable $24,25(OH)_2D$ levels. Genetic testing revealed that 3 patients were homozygous for c.428_430del AAG, while case 4 was compound heterozygote for c.428_430del AAG and c.1186C>T. In case 1, treatment with Rifampin 600 mg, after holding cinacalcet treatment, resulted in moderate decrease in calcium levels, from 11,28 mg/dl to 10,4 mg/dl. The patient developed asthenia, leading to transient interruption of rifampin.

**Conclusions:** Biallelic CYP24A1 inactivating mutations result in 1,25$(OH)_2$D mediated hypercalcemia. Rifampin might be an alternative treatment in severe cases with HCINF1.

| Case | 1 | 2 | 3 | 4 |
|---|---|---|---|---|
| Age (yrs) | 57 | 25 | 24 | 44 |
| Ca (mg/dl) | 11,5 | 10.2 | 10.9 | 10.2 |
| P (mg/dl) | 3,4 | 3.4 | 2.6 | 4.9 |
| Creatinine (mg/dl) | 2,0 | 1,15 | 1,0 | 0,84 |
| eGFR (ml/min/1.73$m^2$) | 38 | 91 | 108 | 110 |
| 24h U Calcium (mg) | 326 | 320 | 685 | 498 |
| 24h U Creatinine (mg) | 1128 | 1400 | 2271 | 1896 |
| PTH (pg/ml) | 23 | 1.1 | 2.2 | 5.7 |
| 25(OH)D (ng/ml) | 39 | 70.9 | 28.8 | 43 |
| 1,25$(OH)_2$D (pg/ml) | 50 | 73 | 69 | 42 |
| 24,25$(OH)_2$D (ng/ml) | <0.26 | <0.26 | <0.26 | <0.26 |
| Genetic testing | Homozygote c.428_430 del AAG | Homozygote c.428_430 del AAG | Homozygote c.428_430 del AAG | Compound heterozygote for c.428_430 del AAG and c.1186C>T |
| Treatment | Cinacalcet 30 mg bid | - | HTZ | HTZ, ALEN |

**P233**

**Hexasodium fytate (SNF472) inhibits ectopic calcification in pseudoxanthoma elasticum animal models**

*<u>Maria del Mar Perez</u>[1], Miguel D. Ferrer[1,2], Firas Bassissi[1,3], Ida Joely Jacobs[4], Qiaoli Li[4], Olivier Vanakker[3,5], Lisa Dangreau[3,5], Joan Perelló[1,6], Carolina Salcedo[1,3]*
*[1]Sanifit Therapeutics, N/a, Palma, Spain ; [2]University of the Balearic Islands, Department of Fundamental Biology and Health Sciences, Palma, Spain ; [3]International Network on Ectopic Calcification, Intec, Ghent, Belgium ; [4]Thomas Jefferson University, Department of Biochemistry and Molecular Biology- PXE International Center of Excellence in Research and Clinical Care, Philadelphia, United States ; [5]Ghent University Hospital, Center for Medical Genetics, Ghent, Belgium ; [6]University of the Balearic Islands, Department of Chemistry, Palma, Spain*

**Abstract Text**

**Background:** Pseudoxanthoma elasticum (PXE) is an autosomal recessive disorder leading to ectopic calcification (EC) in the skin, eyes and cardiovascular tissues. Hexasodium fytate (SNF472) is a crystallization inhibitor that showed efficacy in reducing cardiovascular EC progression in a randomized phase II clinical trial.

**Purpose:** Our aim was to assess the potential of SNF472 for the treatment of PXE in a zebrafish model with spinal hypermineralization and a mouse model with connective tissue calcification.

**Methods:** In a first study, abcc6$^{+/-}$ zebrafish were intercrossed and 100 of the resulting larvae were exposed to 1 mM SNF472 for 7 days while 100 other larvae were kept in the same conditions without SNF472. The fish were then euthanized, stained with Alizarin Red S and mineralization was quantified, after which the larvae were genotyped. In a second study, Abcc6$^{-/-}$ mice (5 females and 5 males) were administered with subcutaneous SNF472 at 15 mg/kg thrice weekly for eight weeks. Vehicle-treated WT (C57BL/6J) and Abcc6$^{-/-}$ mice served as negative and positive controls, respectively. Calcification of muzzle skin biopsies, plasma pyrophosphate (PPi) and SNF472 levels and serum alkaline phosphatase (ALP) activity were measured. All procedures were approved by the respective Ethical Committees.

**Results:** SNF472 significantly reduced the calcified area (~40%) in abcc6a$^{-/-}$ zebrafish larvae. The Abcc6$^{-/-}$ mice receiving SNF472 showed a 57% inhibition of the EC in the muzzle (144 µg calcium/g tissue) compared to vehicle-treated Abcc6$^{-/-}$ mice (208 µg calcium/g tissue) and WT mice (97 µg calcium/g tissue). SNF472-treated Abcc6$^{-/-}$ mice had plasma PPi concentrations indistinguishable from the vehicle-treated Abcc6$^{-/-}$ mice (~41% of PPi in the WT mice). The disease and the treatment with SNF472 did not affect serum ALP activity.

**Conclusion:** SNF472 inhibits EC in zebrafish and mouse PXE models, pointing to a potential use of this compound for the treatment of this disease.

**P234**

**Primary hyperparathyroidism in the geriatric population: Bone Density changes after parathyroidectomy**

*Sara Gonnelli*[1]*, Moustapha Niasse*[1]*, Didier Borderie*[2]*, Catherine Cormier*[1]*, Sébastien Gaujoux*[3]*, Eugenie Koumakis*[1]
[1]*Cochin Hospital, Bone Disease Unit-Exploration du métabolisme phosphocalcique, Paris, France ;* [2]*Cochin Hospital, Biochimie, Paris, France ;* [3]*Pitié-Salpêtrière Hospital, Chirurgie Viscérale et hépato-biliaire, paris, France*

**Abstract Text**

**Context:** The positive impact of parathyroidectomy (PTX) on BMD is well-established in RCTs and various biochemical profiles of primary hyperparathyroidism (PHPT). However, limited data exist for geriatric patients, raising questions about PTX's purpose in older age population.

**Purpose:** to assess BMD change after PTX and to compare it between geriatric (≥ 75 years-old) and non-geriatric patients with moderate biological PHPT.

**Methods**: We included 157 PHPT patients (mean age 65.90±11.18 years), successfully treated by PTX. Two groups were categorized by age: 37 geriatric (mean age 79.6±4.6, mean baseline serum calcium 2.61, ionized calcium 1.39 mmol/L), and 120 non-geriatric patients (mean age 61.70, mean serum calcium 2.58, ionized calcium 1.37 mmol/L). Wilcoxon tests assessed BMD change within groups, t-tests were performed to compare groups. A p-value < 0.05 was considered significant.

**Results:** In the geriatric group, 89.2% of patients displayed osteoporosis defined by a T-score ≤-2.5DS at any site, 14 (37.8%) had a history of fractures. After PTX (mean delay after PTX of 1.31±0.68 years),

BMD significantly increased at the spine in both the geriatric and non-geriatric groups (+4%, +2% respectively), and at the total hip in the non-geriatric group. After PTX, 24/37 (64.9%) geriatric and 79/120 (65.8%) non-geriatric patients exhibited BMD gain defined by an increase of at least 30 mg/cm$^2$ (p 0.55). There was no difference in absolute BMD gain at the spine or hip between the geriatric and non-geriatric groups. Distal radius showed a BMD decrease, though not statistically significant, in both groups **(Figure 1). Conclusions:** This study shows a significant increase in spine and hip BMD at in a mild biological PHPT cohort, encompassing 23.6% geriatric patients. The absence of age-related difference in BMD gain after PTX  suggests that bone improvement is independent of age, supporting PTX consideration  for older individuals with PHPT meeting surgical criteria.

Figure 1: Increase in BMD post PTX in patients compared by age

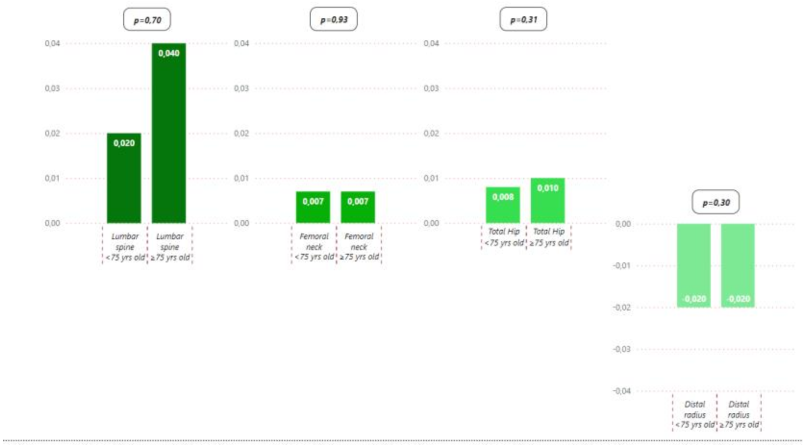

**P235**

**Characterization of the clinical relevant c.1050-3C>G PTH1R variant in periodontal ligament cells via live cell imaging to study molecular effects of primary failure of eruption**

*Katharina Marnet*[1], *Hariharan Subramanian*[2], *Martina Wiesler*[3], *Angela Borst*[4], *Daniel Liedtke*[4], *Denitsa Docheva*[5], *Viacheslav O. Nikolaev*[2], *Martin Eigenthaler*[3], *Marietta Herrmann*[1]
*[1]University Hospital of Wuerzburg, IZKF Group Tissue Regeneration in Musculoskeletal Diseases, Würzburg, Germany ; [2]University Hospital Eppendorf, Institute of Experimental Cardiovascular Research, Hamburg, Germany ; [3]University Hospital of Wuerzburg, Polyclinic for orthodontics, Würzburg, Germany ; [4]University of Wuerzburg, Institute of Human Genetics, Würzburg, Germany ; [5]University of Wuerzburg, Orthopaedic Hospital König-Ludwig-Haus, Würzburg, Germany*

**Abstract Text**

**Introduction:** Primary failure of eruption (PFE) is a rare autosomal nonsyndromic disorder resulting in an open bite situation due to incomplete eruption of mainly posterior teeth and growth deficiency in the alveolar process. PFE is causally linked to genetic variants in the PTH1 receptor gene (*PTH1R*) which belongs to the family of G protein-coupled receptors. These receptors regulate many intracellular signalling molecules, like cAMP which activates downstream protein kinase (PKA), cAMP-gated ion channels and two isoforms of exchange protein directly activated by cAMP (Epac).

**Purpose:** To understand the impact of PFE-linked *PTH1R* variants on intracellular signalling and cellular function in periodondal ligament (PDL) cells.

**Methods:** Using CRISPR/Cas9, the clinically validated c.1050-3C>G *PTH1R* variant was introduced into an immortalized PDL cell line. The variant was incorporated into the genome by a specific homology directed repair (HDR) method, facilitated by a knock-in donor template and proofed by sequencing. cAMP synthesis (Epac1 biosensor) and PKA activation (AKAR3 biosensor) in the wild-type and mutant cell lines were measured by live-cell imaging using Förster Resonance Energy Transfer (FRET)-based biosensors. Phosphorylation of Vasodilator Stimulated Phosphoprotein (VASP) was measured to further validate and compare the PKA activation between the cell lines.

**Results:** The newly created cell line exhibited a heterozygous substitution from C to G precisely at the intended position within the *PTH1R* gene. FRET data revealed that PTH increased cAMP in the wild type, but not in the mutant (P<0.0006, FC ~5.8). PKA activation was also significantly higher in the wild types compared to mutants (P<0.0001, FC ~4.7). Phosphorylation of VASP was disrupted in the mutant cells.

**Conclusion:** FRET assays of PDL cells with a *PTH1R* variant revealed significant intracellular-signalling differences in comparison to the wild type providing important insights in molecular triggering of PFE as a first step towards the development of innovative therapeutic strategies.

**P236**

**Abdominal aortic calcification determined by Kaupilla score and its correlation to echocardiographic measurements in hemodialysis and predialysis patients**

*<u>Tanja Ćelić</u>[1], Antun Lončarić[2], Josip Španjol[3]*
*[1]Faculty of medicine- University of Rijeka- Rijeka- Croatia, Department of anatomy, Rijeka, Croatia ; [2]General Hospital Dr Ivo Pedišić, Cardiology- Internal hospital, Sisak, Croatia ; [3]Clinical Hospital Rijeka- University of Rijeka- Rijeka- Croatia, Urology, Rijeka, Croatia*

**Abstract Text**

Echocardiographic measurements show the characteristics and function of the aortic valve and the left ventricle. Kauppila Score is semi-quantitative system for determining the abdominal aortic calcification on lateral lumbar radiograph and is associated with cardiovascular risk. The latest guidelines suggest lateral abdominal radiograph detects the presence or absence of vascular calcification in patients with chronic kidney disease.

Our study aimed to perform a study to determine whether there is a correlation of echocardiographic measurements and Kauppila Score among dialysis and predialysis patients.

In this study, 63 hemodialysis (HD) and 35 predialysis (PD) patients from General Hospital Ivo Pedišić in 2022 were prospectively enrolled after obtaining informed consent. Echocardiography measurements and Kauppila score for AAC were assessed. Spearman correlation test was used to analyze the relationship between two measurements

There was a statistical difference between HD and PD patients with higher Kaupilla score in the HD group. The correlation analysis found that the correlation between echocardiographic parameters LVEDd, Vmax AoV, PGmax AoV, and Kauppila Score for AAC was significant ($r= 0.518$; $p<0.05$).

Kauppila score has possible use to assess vascular calcification and subsequent cardiovascular risk among dialysis and predialysis patients.

**P237**

### Safety and Exploratory Efficacy of INZ-701 in Adults with ABCC6 Deficiency Manifesting as Pseudoxanthoma Elasticum– Results from 48-week Phase 1/2 Open Label Study

*Yves Sabbagh*[1], *Magdy Shenouda*[2], *Taubel Jorg*[3], *Mark Lebwohl*[4], *Kurt Gunter*[1]
*[1]Inozyme Pharma, Research and Development, Boston- MA, United States ; [2]CliniLabs Drug Development Corporation, N/a, Eatontown- NJ, United States ; [3]St. George's University of London, Richmond Research Institute, London, United Kingdom ; [4]Icahn School of Medicine at Mount Sinai, Department of Dermatology, Mount Sinai- NY, United States*

**Abstract Text**

**Background:** Pseudoxanthoma elasticum (PXE) is a rare mineralization disorder caused by mutations in the *ABCC6* gene. ABCC6 transports ATP into the extracellular space where it is metabolized by the ENPP1 enzyme to AMP and inorganic pyrophosphate (PPi), a critical mineralization inhibitor. PXE therefore is associated with reduced levels of PPi and characterized by ectopic calcification of elastic tissue affecting the skin, eyes, and vasculature. INZ-701 is a recombinant ENPP1-Fc investigational product.

**Purpose:** Describe the safety and exploratory efficacy of INZ-701 in adults with ABCC6 Deficiency manifesting as PXE through end of the phase 2 study period (week 48).

**Methods:** Phase 1/2, multicenter, open-label, multiple ascending dose study including 10 adults total across three dose cohorts (0.2, 0.6 and 1.8 mg/kg), with diagnosis of PXE, biallelic *ABCC6* mutations, and PPi <1300 nM (NCT05030831). Participants were dosed subcutaneously on day 1, then twice weekly from day 8 to end of study. Data as of August 15 2023 are included; topline week 48 data will be presented.

**Results:** Rapid (within 24 hours) increase in mean PPi from baseline of 969±62 nM was noted in 8/9 patients after a single dose of INZ-701, with sustained elevation in the 1.8 mg/kg dosing cohort (Fig 1). Improvements from baseline to week 48 were observed in 9/9 patients in clinician and 7/9 patients in patient global impression of change, respectively (Fig 2). Across cohorts, INZ-701 was well-tolerated with no serious or severe (> grade 2) adverse events. Low titers of non-neutralizing ADA (≤1280) were observed in 8/9 patients. A long half-life of approximately 126 hours suggests the potential for once-weekly dosing.

**Conclusions:** In adults with ABCC6 Deficiency manifesting as PXE, INZ-701 was well tolerated with no serious or severe adverse events. A PPi dose response was observed, with improvements in clinician and patient-reported outcomes across dosing groups.

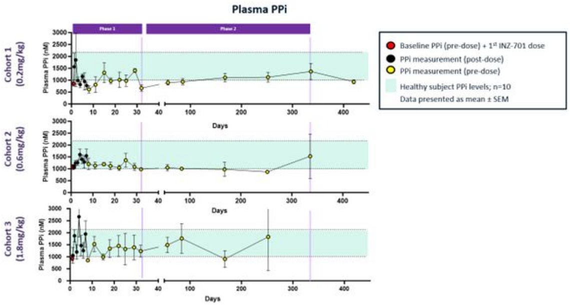

**Figure 1.** Mean plasma pyrophosphate (PPi) across dosing cohorts at each collected time point. Not all patients had reached week 48 as of the August 15, 2023 data cut.

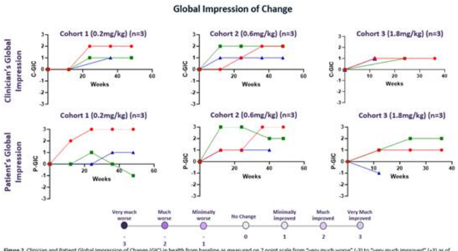

**Figure 2.** Clinician and Patient Global Impression of Change (GIC) in health from baseline as measured on 7 point scale from "very much worse" (-3) to "very much improved" (+3) as of August 15, 2023. Colors represent individual patients in respective cohorts.

**P238**

**The effect of topically applied bone morphogenetic protein-7 on the kidney parenchyma recovery in the acute kidney injury**

*<u>Tanja Ćelić</u>[1], Ante Jakšić[2], Vedran Micek[3], Dragica Bobinac[4], Ivana Marić[4], Josip Španjol[2]*
*[1]Faculty of medicine- University of Rijeka- Rijeka- Croatia, Department of anatomy, Rijeka, Croatia ; [2]Clinical hospital Rijeka- University of Rijeka- Rijeka- Croatia, Urology, Rijeka, Croatia ; [3]Institute for medical investigations and work medicine- Zagreb, Unit for laboratory animals, Zagreb, Croatia ; [4]Faculty of medicine- University of Rijeka- Rijeka- Croatia, Department of anatomy-, Rijeka, Croatia*

**Abstract Text**

Acute insults such as hypoxia, ischemia, medications, toxins, and infections can cause kidney damage, resulting in acute kidney injury (AKI). The kidneys can regenerate, but if the injury is severe or recurrent, the repair will be abnormal, resulting in the development of renal fibrosis. Bone morphogenetic protein-7 (BMP-7) has been recognized as a key signaling molecule during renal development.

In the context of acute and chronic kidney damage in experimental animal models, BMP-7 has demonstrated the ability to counteract the profibrotic effects of transforming growth factor beta (TGFβ), thereby protecting against fibrosis. This study aims to test the hypothesis that the topical application of BMP-7 in cases of acute renal injury promotes parenchymal regeneration and prevents the onset of fibrosis.

A traumatic acute kidney injury (AKI) model was established in rats, and the animals were randomly assigned to either a group without treatment (AKI) or a group receiving BMP-7 application (AKI+BMP7). Histological and immunohistochemical examinations of tissue were conducted, quantifying parameters such as tubule dilation, tubular necrosis, loss of the brush border, and apoptosis. Statistical analysis, utilizing the Student t-test, was employed to assess the significance of observed differences.

The results indicated a substantial statistical difference in tubular dilation, necrosis, and brush border loss between the AKI and AKI+BMP7 groups (p<0.05 for each parameter). Apoptosis was significantly increased in the AKI group compared to the AKI+BMP7 group (p<0.05). These findings suggest that BMP-7 application may have a protective effect against the structural damage associated with acute kidney injury.

The study underscores the potential clinical application of BMP-7 as an anti-fibrotic therapy for renal injuries, offering promise for mitigating the consequences of acute insults on kidney function and structure.

**P239**

**Gorham's disease associated with thyroid hormone resistance, successfully treated with mTOR inhibitor and zoledronic acid: a case report.**

*Arnaud Vanjak*[1,2,3]*, Valérie Bousson*[4]*, Rachid Kaci*[5]*, Martine Cohen-Solal*[1,2,3]*, Thomas Funck-Brentano*[1,2,3]

[1]*Assistance Publique des Hôpitaux de Paris- Lariboisière Hospital- DMU Locomotion, Department of Rheumatology, Paris, France ;* [2]*BIOSCAR UMRS 1132- INSERM- Université Paris Cité, Inserm, Paris, France ;* [3]*National reference center for rare bone diseases- Lariboisière Hospital- APHP.Nord, Filière OSCAR, Paris, France ;* [4]*Assistance Publique des Hôpitaux de Paris- Lariboisière Hospital, Department of Radiology, Paris, France ;* [5]*Assistance Publique des Hôpitaux de Paris- Lariboisière Hospital, Department of Anatomopathology, Paris, France*

**Abstract Text**

We here report the case of a 50-year-old patient with Gorham's disease treated favorably with sirolimus and zoledronic acid for 2.5 years. The patient's main antecedent was syndrome of thyroid hormones resistance.

Her history began 6 months before her admission to hospital, with the sudden onset of right lateralized cervicalgia, with a C6 irradiation. Imaging revealed an osteolytic lesion of the right posterior lamina of C5, with tissue replacement extending outside the bone framework. A bone biopsy revealed the histological presence of vessels within the bone tissue, with the presence of turgid epithelial cells labelled with CD34, anti-d2-40 and anti-prox1, confirming the suspicion of Gorham's disease. Given the instability of her cervical spine, an operation by osteosynthesis was carried out, reducing her pain.

Post-operatively, sirolimus 2 mg daily was started in combination with monthly infusions of zoledronic acid 4 mg for 6 months, then every 3 months. This treatment led to a progressive improvement in pain and frank remineralization of osteolytic areas (Figure). Given the moderate tolerance of sirolimus (asthenia, headache, mouth ulcers) after 2.5 years of treatment, the latter was discontinued with continued close clinical and radiological monitoring.

The aim of this report is to describe the clinical and radiological evolution of Gorham's disease treated with sirolimus and zoledronic acid over 2.5 years. Most of the bone remineralization was already observed after one year of treatment. What happens after treatment discontinuation remains unknown and undocumented in the literature. The respective contribution of treatment with sirolimus alone or zoledronic acid alone is not well known. However, treatment with Interferon combined with zoledronic acid did not give such good results radiologically. The other element of this observation is the association of two extremely rare diseases - thyroid hormone resistance &

Gorham's disease - suggesting a possible causal link between.

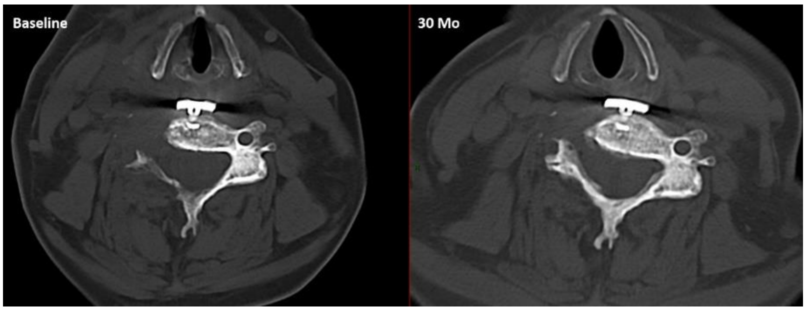

**P240**

**Identification of Dysregulated MicroRNAs in Renal Osteodystrophy by Bioinformatic Analysis**

*Praopilad Srisuwarn*[1], *Syazrah Salam1 Salam*[2,3], *Richard Eastell*[2]
[1]*University of Sheffield, Division of Clinical Medicine, Sheffiled, United Kingdom ;* [2]*University of Sheffield, Division of Clinical Medicine, Sheffield, United Kingdom ;* [3]*Sheffield Teaching Hospitals NHS Foundation Trust, Metabolic Bone Centre, Sheffield, United Kingdom*

**Abstract Text**

**Background:** Renal osteodystrophy (ROD), the bone disorder in chronic kidney disease patients, significantly increases fracture risk and mortality. The dysregulation of microRNAs, non-protein-coding RNA molecules that modulate gene expression, may be relevant in the pathogenesis of ROD.

**Purpose:** To identify candidate miRNAs which are associated with gene dysregulation in ROD.

**Methods:** Next-generation sequencing data (SRP356156) investigating gene expressions in bone tissues from biopsy-proven ROD patients and healthy controls were obtained from NCBI GEO. The data were uploaded to the Galaxy web tool and analysed to identify significantly differentially expressed protein-coding genes ($p$-value < 0.05). Candidate miRNAs were identified using the enrichMIR web tool, based on mirTarBase8, which curates miRNAs-gene data from experiments.

**Results:** Sequencing data of 29 patients, including 9 controls, 9 low-turnover ROD (L-ROD) and 11 high-turnover ROD (H-ROD), were analysed. The median age (interquartile range) were 47(43.5), 39(35.5) and 39(32.5) years, for controls, L-ROD and H-ROD, respectively. One control was excluded due to sequencing quality concerned. Predictive analysis revealed 49 significantly upregulated microRNAs in L-ROD and 69 in H-ROD ($p$-value < 0.05). Notably, 9 of these miRNAs were previously reported as upregulated in the serum of osteoporosis patients.

**Conclusion:** The study suggests a potential overlap in miRNA expression abnormalities between ROD and osteoporosis. Validation of candidate miRNA expression in ROD patients is warranted to further elucidate their role.

**P241**

**Carotid-femoral pulse wave velocity in patients with Paget's disease of bone: a cross-sectional and prospective study**

Veronica Abate[1], Anita Vergatti[2], Alfonso Giaquinto[1], Lanfranco D'Elia[1], Daniela Merlotti[3], Luigi Gennari[3], Domenico Rendina[1]
[1]Federico II University, Department of Clinical Medicine and Surgery, Napoli, Italy ; [2]Federico II University, Department of Clinical Medicine and Surgery, Naples, Italy ; [3]University of Siena, Department of Medicine- Surgery and Neurosciences, Siena, Italy

**Abstract Text**

**Introduction:** Cardiovascular diseases (CVDs) are frequently described in patients with Paget's disease of bone (PDB), a common metabolic bone disorder (MBD) affecting one (monostotic PDB) or more (polyostotic PDB) skeletal sites. The pulse wave velocity (PWV) is a early and sensible marker for CVDs.

**Purpose:** This study aims to evaluate the PWV in PDB patients compared to controls without MBD.

**Methods:** PDB patients and controls were recruited among ones referring to Federico II Univerisity of Naples. All patients underwent a biochemical and PWV evaluation at the enrollment. PDB patients were classified according to disease extension (monostotic vs. polyostotic PDB), occurrence of *sequestosome 1* mutations, and disease activity [total alkaline phosphatase (tALP) levels elevated vs. normal]. PDB patients with elevated tALP (active disease) were evaluated at the enrollment (T0) and 6 months after (T6) zoledronate (Zol) treatment.

**Results:** 37 PDB patients and 25 controls were recruited.
At T0, PDB patients showed higher PWV compared to controls (9.76±2.02 vs 8.00±1.96 m/s; $p<0.01$).
PDB patients showing elevated tALP levels, polyostotic disease and *sequestosome 1* mutations are 7, 25, and 12, respectively. The PWVs were not different between PDB dichotomized according to tALP levels, PDB extension, and occurrence of *sequestosome 1* mutations. A significant correlation between 25-hydroxy-vitamin D (25OHD) and PWV values ($r= -0.38$; $p<0.05$).
At T6, all PDB patients treated with Zol showed normal tALP levels without significant PWV variation.
Correlation between 25OHD and PWV evaluated at T6 only in PDB patients treated with zoledronic acid was not significant ($r= 0.14$; $p=0.79$).

**Conclusion:** The study results indicate that the increased PWV is a constitutive element of PDB itself, which should be considered a clinical condition predisposing CVDs.

**P242**

### Model severity-dependent inhibition of ectopic calcification by hexasodium fytate (SNF472): insights from in vitro and in vivo investigations

Miguel D. Ferrer[1,2], M. Mar Pérez[1], Marc Blasco[1], Andrea López[1], Gianluca Malagraba[2], Firas Bassissi[1], Joan Perelló[1,3], Carolina Salcedo[1]
[1]Sanifit Therapeutics, N/a, Palma, Spain ; [2]University of the Balearic Islands, Department of Fundamental Biology and Health Sciences, Palma, Spain ; [3]University of the Balearic Islands, Department of Chemistry, Palma, Spain

**Abstract Text**

**Background:** Hexasodium fytate (SNF472) is a crystallization inhibitor being developed for ectopic calcification-related disorders such as calciphylaxis.

**Purpose:** To evaluate the efficacy of SNF472 in models of ectopic calcification with variable degrees of calcification.

**Methods:** Calcification was induced in two in vitro models of vascular smooth muscle cells by 3.3 mM phosphate (three weeks) or 3 mM calcium and 3 mM phosphate (two weeks). SNF472 was tested at 10, 30 and 100 µM. In an in vivo study, calcinosis cutis was induced in 24 Sprague Dawley rats by vitamin D (50,000 IU/kg) followed by subcutaneous administration of 0.15 mg $FeCl_3$ at the two ventral sites in the thorax on day 4. Twelve rats were treated daily with subcutaneous 60 mg/kg SNF472, 12 rats were administered with 0.9% saline and six healthy rats were included. The skin surrounding the $FeCl_3$ injection sites and the femoral arteries were collected at day 8 for calcium content quantification. All procedures were approved by the respective Ethical Committees.

**Results:** The cultures treated with calcium and phosphate were 10-fold more calcified than those treated with phosphate alone. The inhibitory potency of SNF472 was influenced by the extent of calcification, with $IC_{50}$ of 2.1 µM in the phosphate-only model and 14 µM in the calcium and phosphate model. A low degree of calcification was induced in femoral arteries (1.6 mg/g in vehicle-treated animals vs 0.4 mg/g in healthy animals) with an 80% inhibition of calcification by SNF472, while a higher degree of calcification was induced in the skin (3.9 mg of calcification in vehicle-treated animals vs 0.06 mg in healthy animals) with 60% inhibition by SNF472.

**Conclusion:** The inhibitory effect of SNF472 is influenced by the degree of calcification, suggesting that its potential therapeutic effect could be more pronounced in the early stages of calcification related diseases.

**P244**

**Assiciations of bone microstrucutre and lower leg arterial calcifiaction in non CKD patients**

*Mikolaj Bartosik[1], Alexander Simon[2], Björn Busse[1], Florian Barvencik[1], Ralf Oheim[1], Michael Amling[1], Felix Nikolai Schmidt[1]*
*[1]University Medical Center Hamburg-Eppendorf, Department of Osteology and Biomechanics, Hamburg, Germany ; [2]University Medical Center Hamburg-Eppendorf, Department of Trauma and Orthopaedic Surgery- Division of Orthopaedics, Hamburg, Germany*

**Abstract Text**

**Introduction:** Calcium homeostasis and related bone loss is a main topic of osteoporosis. In this context, especially by patients, the topic of vascular calcifications is discussed frequently when it comes to oral calcium uptake. Thus, an interaction of the occurrence of vascular plaques is suspected. Here, we aimed to analyze associations that may exist between bone mineralization and vascular calcification occurrence at the distal tibia. Furthermore, we studied the influence of calcium supplementation and interactions of vessel calcification on bone mineral density (BMD) measurements.

**Methods:** 774 patients and the relating high-resolution quantitative computed tomography (HR-pQCT) scans of the distal tibia were retrospectively analyzed for the presence of vascular calcifications. Patients with calcifications were sex-specifically propensity score matched for age and BMI. Bone microstructure and bone density was analyzed, as well as lower leg artery calcification (LLAC) was quantified, and laboratory parameters of bone turnover were measured in 132 patients. Patients with genetic diseases, CKD grade ≥3B (<45ml/min) or active tumors were excluded. Interactions were calculated by regression analyses.

**Results:** Bone and calcium metabolism and calcium supplementation did not differ between individuals with and without LLAC. A higher cortical perimeter (LLAC: Ct.Pm=107.7±7.9 mm *vs.* no LLAC: Ct.Pm=103.1±8.7 mm; $p$=0. 016) was detected in women with LLAC compared to women without LLAC. In men, patients with LLAC had significantly lower cortical pore diameter ($p$<0.027) than men without LLAC. Plaque density and age was associated with bone parameters from HR-pQCT in women. However, in men plaque volume was associated with HR-pQCT parameters ($R^2$=0.435, $p$=0.026).

**Conclusions:** LLAC appearance and its association to HR-pQCT parameters is sex-specific. In women, increased plaque density is linked to trabecular thinning and age. Increasing plaque volume, in contrast to women, in men is linked to decreasing cortical pore diameter and trabecular number along with increasing bone mineralization.

### Poster Sessions: Paediatric bone diseases

### P245

### Identification of circulating microRNAs signature in in adolescent females with severe idiopathic scoliosis

*Lavinia Raimondi[1], Fabrizio Perna[2], Alessia Gallo[3], Angela De Luca[1], Daniele Bellavia[1], Viviana Costa[1], Nicola Cuscino[3], Dario Simone Scilabra[4], Gianluca Giavaresi[1], Angelo Toscano[2]*
*[1]IRCCS Istituto Ortopedico Rizzoli, Scienze e Tecnologie Chirurgiche, Bologna, Italy ; [2]IRCCS Istituto Ortopedico Rizzoli, Ortopedia Generale, Bologna, Italy ; [3]IRCCS-ISMETT, Dipartimento di Ricerca, Palermo, Italy ; [4]IRCCS ISMETT-Fondazione Ri.MED, Dipartimento di Ricerca-Unità di proteomica, Palermo, Italy*

**Abstract Text**

Adolescent Idiopathic Scoliosis (AIS) is an abnormal curvature of the spine. Although several studies have not evidenced significant differences between males and females in the moderate forms of AIS, the diagnoses are strongly correlated to females in the severe disease. Our study aimed to identify predictive biomarkers for curve progression. We performed miRNA profiling through qRT-PCR array cards, in order to analyze miRNAs in the plasma of 20 patients (Table 1) and 10 healthy controls (HC). Volcano plot analysis of deregulated miRNAs from plasma of AIS patients and HC ($p \leq 0.05$ and fold change ≥ 1.5) showed a specific miRNAs signature (Figure 1). Further analysis revealed the expression of miR-30 family members restricted to severe AIS females. Circulating extracellular vesicle (EVs) from severe females decreased the osteogenic differentiation of mesenchymal stem cells (*, p < 0.05-AIS EVs MSC treated versus MSC untreated) and reduced extracellular mineralization (***, p < 0.0005—AIS-EVs MSC treated versus MSC untreated). LC-MS-based proteomic analysis of EVs from severe females revealed proteins involved in orthopedic disorders. Our results showed that circulating miRNAs could discriminate AIS patients from HC, also through an association between gender and grade of disease.

| Variable | Mean [95% CI] | Range |
|---|---|---|
| AGE | 14.7 [14.3, 15.0] | 13-17 |
| GENDER: | | |
| Male | 3 | |
| Female | 17 | |

| | | |
|---|---|---|
| Cobb Angle (°) | 54.6 [50.5, 58.7] | 21-92 |
| Risser Angle | 3.4 [3.0, 3.7] | 0-5 |
| BMI | 21.6 [20.6, 22.6] | 16.4-33.2 |
| LENKER CLASSIFICATION | | |
| Type 1A | 8 | |
| Type 1B | 1 | |
| Type 1C | 6 | |
| Type 2A | 1 | |
| Type 3 | 1 | |
| Type 5 Type 6 | 21 | |

**Table 1**. Demographic and clinical parameters of adolescent idiopathic scoliosis patients; Mean value and 95% CI for age, Cobb and Risser angles, and BMI.

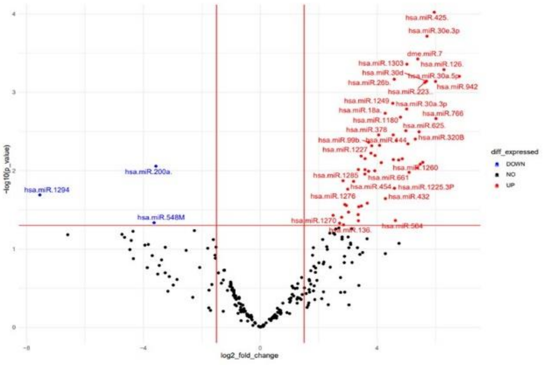

**Figure 1.** Volcano Plot analysis of differentially expressed miRNAs analysis in AIS patients and HC.

The study was conducted according the guidelines of the Declaration of Helsinki and approved by Ethic Committee of Comitato Etico di Aria Vasta Emilia Romagna (Italia)- Istituto Ortopedico Rizzoli- Protocol ID: CE-AVEC 575-2020-Sper-IOR.

### P246

### Hydrogel-based bioink delivering in situ therapeutic small molecules within calvarial mesenchymal stromal cells as an innovative strategy for the treatment of paediatric calvarial bone defect

*Martina Salvati[1], Federica Tiberio[2], Luca Polito[1], Lorena Di Pietro[2], Gianpiero Tamburrini[3], Noah Giacon[4], Ornella Parolini[2], Alessandro Arcovito[5], Wanda Lattanzi[2]*
*[1]Università Cattolica del Sacro Cuore, Dipartimento di Scienze della vita e Sanità Pubblica, Rome, Italy ; [2]Università Cattolica del Sacro Cuore- Fondazione Policlinico Universitario A. Gemelli IRCCS, Dipartimento di Scienze della Vita e Sanità Pubblica, Rome, Italy ; [3]Università Cattolica del Sacro Cuore- Fondazione Policlinico Universitario A. Gemelli IRCCS, Dipartimento Scienze dell'invecchiamento- neurologiche- ortopediche e della testa-collo, Rome, Italy ; [4]Università Cattolica del Sacro Cuore, Dipartimento di Scienze Biotecnologiche di Base- Cliniche Intensivologiche- e Perioperatorie, Rome, Italy ; [5]Università Cattolica del Sacro Cuore- Fondazione Policlinico Universitario A. Gemelli IRCCS, Dipartimento di Scienze Biotecnologiche di Base- Cliniche Intensivologiche- e Perioperatorie, Rome, Italy*

**Abstract Text**

**Introduction:** Paediatric cranioplasty is an invasive bone reconstructive, burdened by severe risks and high morbidity, due to infections, dislocations and resorption of currently used grafts, also due to the dynamic nature of the paediatric skull.

**Purpose**: This study aims to develop a mouldable hydrogel-based bioink delivering *in situ* therapeutic molecules able to modulate the osteogenic cascade, as an innovative strategy to improve the treatment of paediatric calvarial defects.

**Methods:** Calvarial mesenchymal stromal cells (CMSCs) were isolated from bone tissue waste of paediatric patients undergoing cranioplasty (protocol n. 4876). Poly (lactic-*co*-glycolic acid) (PLGA) nanoparticles (NPs) were used as nanocarriers for small interfering RNAs (siRNAs) delivery. PLGA NPs were produced by double emulsion technique, characterized through dynamic light scattering and scanning electron microscopy; biocompatibility was evaluated by MTT viability assay. NPs intracellular trafficking was investigated through Live-cells analysis and confocal microscopy using 6-coumarin-fluorescently-labeled-PLGA NPs and Lysotracker. The *in vitro* treatment efficiency of siRNA-loaded-PLGA NPs was analysed using qPCR. Fluorescently-labeled-PLGA NPs were printed with hydrogel and incubated with CMSCs to evaluate intracellular NPs release.

**Results:** PLGA biocompatibility was confirmed in CMSCs after 48 hours treatment. Trafficking analysis revealed an intracellular uptake after 15 minutes of NPs incubation; the cytoplasmic distribution mostly spared lysosomes. FGFR2 siRNAs were efficiently encapsulated in PLGA NPs and qPCR revealed a significant and stable inhibition of *FGFR2* until 96 hours of treatment in CMSCs. Live-cell microscopy showed a sustained intracellular release of fluorescently-labeled-PLGA NPs from the hydrogel until 3 days of incubation. CMSCs invariable grew in presence of hydrogel-PLGA NPs confirming the biocompatibility.

**Conclusion:** Our data suggest that PLGA NPs are suitable for intracellular siRNAs delivery in CMSCs and that hydrogel-PLGA-NPs bioink enables a controlled and stable release of NPs in surrounding cells. This approach may represents a promising strategy for *in situ* delivery of therapeutic molecules in bone defects.

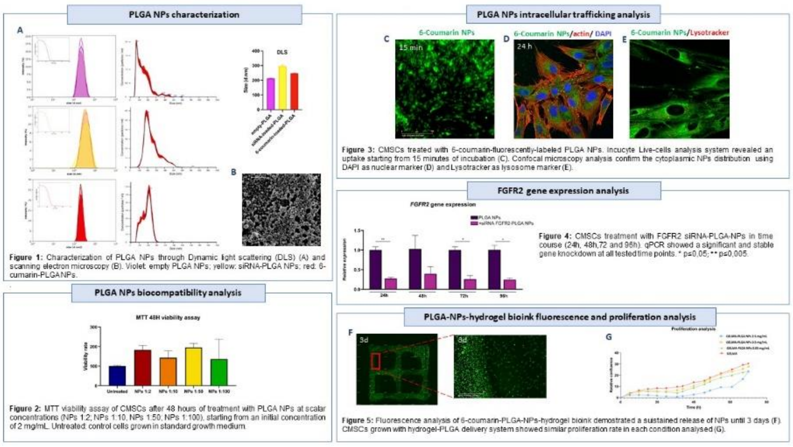

**P247**

**Availability of physical therapy for children with osteogenesis imperfecta in Austria**

*Magdalena Jablonska[1,2], Gabriel Mindler[3,4], Adalbert Raimann[1,2]*
*[1]Medical University of Vienna, Division of Pediatric Pulmonology- Allergology and Endocrinology-*

*Department of Pediatrics and Adolescent Medicine, Vienna, Austria ; [2]Vienna Bone and Growth Center, Pediatrics, Vienna, Austria ; [3]Orthopedic Hospital Speising, Department of Paediatric Orthopaedics, Vienna, Austria ; [4]Vienna Bone and Growth Center, Orthopedics, Vienna, Austria*

**Abstract Text**

**Background:** Physical therapy is an essential component in the multidisciplinary care for patients with rare bone disorders, including osteogenesis imperfecta (OI). Similar to other European regions, most patients with OI in Austria rely on the availability of extramural therapists. However, access may be limited due to factors such as long waiting times and lack of public insurance coverage. Structured data on accessibility and burdens to receive extramural physical treatment is sparsely available.

**Purpose:** This study aims to depict the access to physical therapy for pediatric patients with OI in Austria as a sample region.

**Methods:** Physical therapists of the official Austrian registry (Physio Austria) have been contacted with a standardized call for therapeutic consultation with a 5 year old patient with OI type I. Waiting time to the first appointment and the cost of treatment will be documented and analyzed regarding regional differences.

**Results:** 483 functional therapists across Austria with declared focus on pediatrics have replied to an initial request. Nationwide, 21 physiotherapists (4%) offered an appointment on public health insurance plans with an average waiting time of 50 days. 263 physiotherapists (55%) offered a private appointment with an average waiting time of 36 days. 62 (13%) rejected any offer due to the diagnosis of the sample patient, the remaining 137 (28%) declined for other or unspecified reasons.

**Discussion:** The results of this confirm reported difficulties faced by families of children with OI in accessing functional treatments. Despite regional differences in health care systems, similar findings can be expected in other European regions. Waiting times and high costs could prevent children from receiving necessary treatments with negative impact on their physical and mental health. By identifying regional differences, this study aims to raise awareness and to improve access to physical treatments for children with chronic bone disorders.

**Poster Sessions: Rare bone diseases**

**P248**

**CFTR inhibition favors lipid accumulation in skeletal stem cells: a new paradigm to explain the Cystic Fibrosis-related Bone Disease**

*Claire Dumortier[1], Laurine Hamon[1], Johan Sergheraert[1], Mezhoura Ouhamou[1], Olivier Piot[2], Emilie Buache[2], Nicolas Goffin[2], Christophe Chauveau[3], Sophie Gangloff[1], Denise Alalam[4], Frédéric Velard[1]*
*[1]University of Reims Champagne-Ardenne, EA 4691 BIOS « Biomatériaux et Inflammation en site Osseux », Reims, France ; [2]University of Reims Champagne-Ardenne, EA 7506 BioSpeCT « BioSpectroscopie Translationnelle », Reims, France ; [3]University Littoral Côte d'Opale, ULR 4490 MabLab « Marrow Adiposity & Bone Lab », Boulogne-sur-Mer, France ; [4]The Lundquist Institute, Harbor-UCLA Medical Center, Torrance, United States*

**Abstract Text**

Cystic Fibrosis related Bone Disease (CFBD) affects 50% of CF adult patients. Osteoblasts obtained from CF patients' induced pluripotent stem cells (iPSCs) evidenced a higher PPARG level (adipocyte transcription factor) than healthy donors-derived control line. As a relationship exists between low bone density and increased medullar adiposity in several pathologies (Verma, 2002; Piccinin, 2014, Schafer, 2015; Bredella, 2009), we hypothesised that adipocyte differentiation would be preferentially triggered in cells bearing mutation in CFTR gene, which causes CF. Up to date, medullar adiposity has never been investigated in CFBD.

Skeletal stem cells (SSC) from non-CF patients have been differentiated into adipocytes during 21 days with or without $Inh_{172}$ (CFTR pharmacological inhibitor). Oil Red'O, Bodipy®, Perilipin, FABP4 (adipocytes specific proteins) and PPARG stainings were performed. RAMAN microspectroscopy was used to assess lipid content. MicroCT analysis of 10 weeks-old F508del CFTR mice was performed to determine bone marrow adiposity.

Lipid vesicles were 50% bigger when CFTR inhibitor was added in the medium (n=6 donors, $p<0.05$). However, no change in PLIN1, FABP4 and PPARG genes or protein expression, or adiponectin and leptin secretion were highlighted. Lipid droplets spontaneous generation was evidenced by RAMAN, Oil Red O and BODIPY stainings, in SSC cultured in presence of $Inh_{172}$. This may reflect the spontaneous adipocyte commitment of SSC associated with impaired CFTR function. In addition, we evidenced increased bone marrow adiposity in mice bearing F508del CFTR mutation as compared to wildtype littermates.

These data are the first proof of concept that CFTR loss of function influences size and content of mature adipocytes lipid vesicles, as well as on SSC commitment towards adipocyte lineage. More, increased bone marrow adiposity observed in CF mice tibia enforced the idea of a defective lipid metabolism in CF context that may be a promising axis to better understand and cure CFBD.

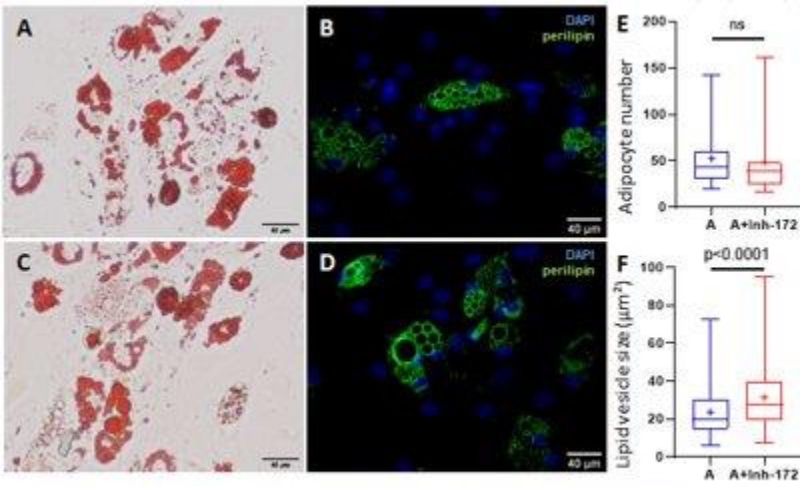

**A, C)**, O red oil staining and **B, D)** Perilipin (green) with DAPI (blue) immunostaining images of adipocytes generated with or without $Inh_{172}$. **E)** Adipocyte number per field at day 21. **F)** Lipid vesicle size (µm²) at day 21 (ImageJ Software). n=6 independent SSC donors. Non parametric stratified Wilcoxon Mann Whitney Test.

**P249**

**An Unprecedented Association; Coronary Artery Disease and Sagliker Syndrome**

*Irem Yilmaz*[1], *Nilufer Eksi Duran*[1], *Mehmet Uzun*[1]
[1]*Sultan Abdulhamit Han Traning and Research Hospital, Cardiology, istanbul, Turkey*

**Abstract Text**

A 39-year-old woman presented to the outpatient clinic with complaints of exertional dyspnea, joint pain and weakness in the legs. She had been on hemodialysis for 5 years due to end-stage renal failure. Her medical history revealed that she underwent kidney transplantation in 2012 and had a history of rejection 2.5 years after transplantation. Two years earlier, he had undergone stent implantation for a critical lesion in the left anterior descending artery (Figure 1-2). Physical examination revealed dental deformities, malocclusion, caries, protruding lips, enlargement of the mandible and maxilla, short neck, short stature (Figure 4), finger deformities (Figure 5), with distinct phenotypic features consistent with Sagliker Syndrome, which is rare in patients with chronic renal failure compared to cases in the literature.

In the medical history of the patient, it was reported that parathyroidectomy was performed 8 years ago and weakness, fatigue and serum PTH level was 1989 ug/L three years after the operation. The patient underwent a second parathyroidectomy. However, despite two parathyroidectomies, the deformities persisted in the long- term follow-up. At the last nephrology clinic evaluation, FDG PET-CT was ordered because of diffuse bone pain and revealed 44mm enlarging sclerotic lesion in the right 9th rib (Figure 6), a similar lesion with 37 mm FDG uptake in the left 10th rib(Figure 7).

Sagliker Syndrome (SS) is a rare complication seen in patients with chronic kidney disease (CKD). Our patient contributed to the literature with his clinical history, extensive systemic involvement and genetic analysis of SS. Especially in young patients with CKD, the risk of SS, coronary artery disease (CAD) and bone marrow fibrosis due to hyperparathyroidism may increase. It is crucial to raise awareness for regular control and follow-up of such cases. Increased vigilance and awareness can significantly impact prognosis, leading to early diagnosis and appropriate treatment options.

**P250**

### Interactions between Fibroblast Growth Factor 23 and Iron Metabolism in X-Linked Hypophosphatemia (XLH)

*Gaetano Paride Arcidiacono*[1], *Valentina Camozzi*[2], *Martina Zaninotto*[3], *Marco Onofrio Torres*[1], *Francesca Zanchetta*[1], *Michele Cannito*[2], *Alberta Cecchinato*[1], *Martin Diogo*[1], *Mor Peleg Falb*[1], *Paolo Simioni*[1], *Mario Plebani*[3], *Stefania Sella*[1], *Sandro Giannini*[1]
*[1]University of Padua, Department of Medicine - Clinica Medica 1, Padua, Italy ; [2]University of Padua, Department of Medicine - Endocrinology Unit, Padua, Italy ; [3]University of Padua, Department of Medicine - Laboratory Medicine Unit, Padua, Italy*

**Abstract Text**

**Background**: X-linked hypophosphatemia (XLH) is a rare hereditary disorder, characterized by renal phosphate wasting, resulting in impaired bone mineralization. XLH is caused by increased levels of Fibroblast Growth Factor 23 (FGF23), a bone-derived phosphaturic hormone. FGF23 is released in its intact biologically active form and issubsequently cleaved into inactive degradation products. Commercially available assays can detect either intact form (iFGF23) or both intact and C-terminal fragments (cFGF23). Iron deficiency (ID) has been described to induce FGF23 production under various conditions, but limited evidence is available on the effect this mechanism could have in XLH. This study aimed to investigate the relationship between iron status and FGF23 levels in patients with XLH.

**Methods**: We enrolled adult patients with a diagnosis of XLH, excluding those with anemia and/or taking iron supplementation. We defined ID with ferritin < 30 mcg/L and/or transferrin saturation

<16%.

**Results**: We included 19 patients, aged 25-60 years, of whom 12 were female (63.2%) and 5 had ID (26.3%). No significant correlations were found between serum iron and iFGF23 (p = 0.587) or cFGF23 (p = 0.312); however, a significant correlation was observed between serum iron and iFGF23 to cFGF23 ratio (r = 0.660, p = 0.03). Patients with ID had a higher iFGF23 to cFGF23 ratio compared to those without ID (p = 0.03), but no differences were found in serum phosphate levels (p = 0.701) and TmP/GFR (p = 0.416) between the two groups.

**Conclusions**: We hypothesize that in XLH, where FGF23 increases but its cleavage is not affected, at low iron concentrations the rise in hormone concentrations may be balanced by an increase in cleavage (as suggested by an altered ratio) and therefore this may mitigate the impact of ID on serum phosphate and urinary phosphate excretion.

**P251**

**Osteoblast from cranio facial fibrous dysplasia of bone : in vitro characterization and impact on bone homeostasis**

*Johan Sergheraert[1,2,3], Claire Dumortier[1], Christine Guillaume[1], Sébastien Laurence[2,3], Fabien Bornert[4], Sophie C Gangloff[1,5], Cédric Mauprivez[1,2,3], Frédéric Velard[1]*
*[1]Université de Reims Champagne-Ardenne, Biomatériaux et Inflammation en Site Osseux- BIOS EA 4691, Reims, France ; [2]Centre Hospitalo-Universitaire de Reims, Pôle de Médecine Bucco-Dentaire, Reims, France ; [3]Université de Reims Champagne-Ardenne, UFR d'Odontologie, Reims, France ; [4]Hôpitaux Universitaires de Strasbourg, Pôle de Médecine et de Chirurgie bucco-dentaires, Strasbourg, France ; [5]Université de Reims Champagne-Ardenne, UFR de Pharmacie, Reims, France*

**Abstract Text**

Fibrous Dysplasia (FD) is a benign and rare genetic disease characterized by progressive replacement of normal bone by a non-mineralized matrix. FD is caused by GNAS mutations, causing $G\alpha_s$ overactivation which increases intracellular cAMP levels. While appendicular lesions have been widely studied for improvement in understanding and management, craniofacial lesions pathogenesis remains poorly described although these two skeletal regions present embryological, phenotypic and mechanical differences. Our objective was to evaluate the impact of craniofacial FD on bone homeostasis.

Osteoblasts were obtained from FD (n=7) and non-FD (n=8) patients during oral surgery (DC-2014-2262) and cultured in osteogenic medium for 21 days. cAMP level was measured as a hallmark of GNAS mutation. Proliferation assays (DNA, WST-1), osteoblastic phenotype characterization (ALPL, Alizarin red, COLIA1 and osteocalcin immunostaining, RT-qPCR) were evaluated at day 7, 14 and 21. Additionally, osteoclast differentiation was performed in osteoblasts' conditioned medium. After 21 days of differentiation, OC were stained (Phalloïdin-AlexaFluor488®, Vinculin-immunofluorescent staining, DAPI) to determine osteoclasts' number and size.

Higher intracellular cAMP levels were observed in FD cultures from craniofacial lesions. We showed an increased proliferative capacity in craniofacial FD osteoblasts, associated with decreases in osteoblastic differentiation and a lack on mineralization capacities marked by a decreased RA staining and osteocalsin immunostaining. Interestingly, we demonstrated a decrease in osteoclastogenic potential marked by an increase in OPG expression and secretion (3.6fold at day 21, p<0.05) from craniofacial FD osteoblasts compared to controls. This observation was sustained by significantly twice less OC formation in FD-conditioned medium as compared to nonFD-conditioned medium at 21-days-culture without any variation of their area.

Our data suggest shared characteristics between osteoblasts from craniofacial and appendicular skeleton but also discrepancies regarding osteoclastogenic potential. These features could explain differences observed between appendicular and cranio-facial lesions and should lead to further studies on pathogenesis of craniofacial fibrous dysplasia.

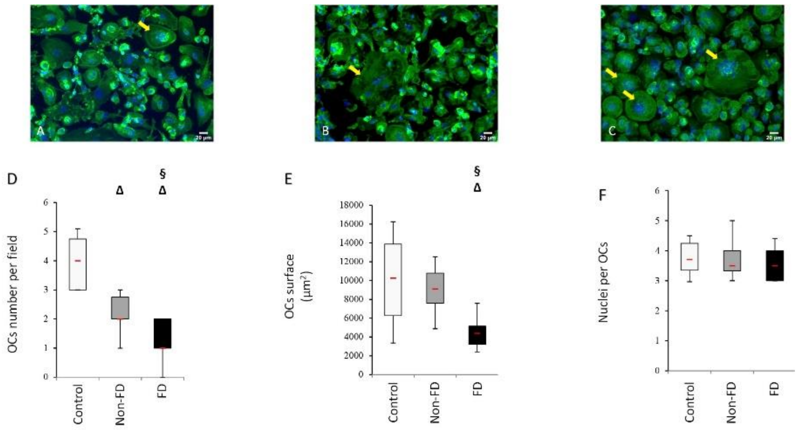

Representative photographs of actin F staining in FD medium (A), non-FD medium (B) and control (C) cultures. Phalloidin-AlexaFluor®488 / nucleus (DAPI). Scale bar: 20µm. Yellow arrows highlighted Ocs. A decreased OCs number in FD-medium culture compared to nonFD-medium culture and control medium (D). FD medium induced a decreased OCs surface (E). No variation in nuclei per OCs (F). White values represent control, grey values represent non-FD cells and black values represent FD cells.
Control medium, n=6 ; FD-medium, n=6 ; nonFD-medium, n=6. Δ $p<0.05$ versus control ; § $p<0.05$ versus non-FD

**P252**

**Evolutive bone phenotype in the cherubism mouse model**

*Badre Rabhi*[1], *Sylvie Thomasseau*[2], *Xavier Decrouy*[3], *Martine Cohen-Solal*[4], *Marcel Deckert*[5], *Amélie Coudert*[6], *François Brial*[4]
*[1]BIOSCAR- Inserm U1132, Université Paris Cité, Paris, France ; [2]BIOSCAR- Inserm U1132, Inserm U1132, Paris, France ; [3]IMRB- Creteil, Université Paris Est Creteil, Creteil, France ; [4]BIOSCAR- Inserm U1132, UFR de Médecine- Université de Paris Cité, Paris, France ; [5]INSERM U1035- Université Nice Sophia-Antipolis- Microenvironment- Signaling and Cancer, Université Nice Sophia-Antipolis, Nice, France ; [6]BIOSCAR- Inserm U1132, UFR d'Odontologie- Université de Paris Cité, Paris, France*

**Abstract Text**

Cherubism (OMIM # 118400) is an evolutive paediatric rare bone disorder caused by SH3BP2 mutations and characterised by osteolysis of the jaws with replacement of bone by a granuloma containing multinucleated giant cells. To gain insight into the pathogenesis of the disease, a knock-in mouse model, Sh3bp2 KI mouse, has been generated and is characterised by a systemic bone loss at 10 weeks. We want to know if the development of jaw granulomas is associated with the bone loss phenotype appearance in mice. We monitored the disease progression at 6, 8 and 10 weeks in the mouse model. Hence, we measured body weight and length, bone mineral content and density, bone architecture in Sh3bp2KI femurs, vertebrae and erosion in calvarias. As early as 6 weeks, the Sh3bp2KI males are significantly lighter than the WT (20 vs 22g, p= 0,0026). The BMD of the SH3BP2 KI is 9,5 % lower than that of the WT (52 vs 57 mg/cm2, p=0,0004) at 6 weeks. In addition, the BV/TV

in the femur of the Sh3bp2KI was significantly decreased by 50,4 % (p= 0,0006) at 6 weeks, 63,9% (p<0.0001) at 8 and 70,5% (p<0.0001) at 10. The trabecular parameters of the Sh3bp2KI were different from the WT (Tb.Th -16.71 % p= 0,0230, Tb.Sp + 40.45 % p= 0,0014, Tb.N - 45.88 % p= 0,0004) at 6weeks.We found the same bone loss phenotype at 8 and 10 weeks and in the vertebrae of the Sh3bp2KI mice. From 6 weeks, the eroded region of the skull was increased in the Sh3bp2KI compared to the WT (+49.65 % p=0,0293), as at 8 and 10 weeks. Female mice show the same bone loss phenotype. These results show that from 6 to 10 weeks, Sh3bp2KI mice have a gradually worsening bone loss phenotype, suggesting an earlier onset.

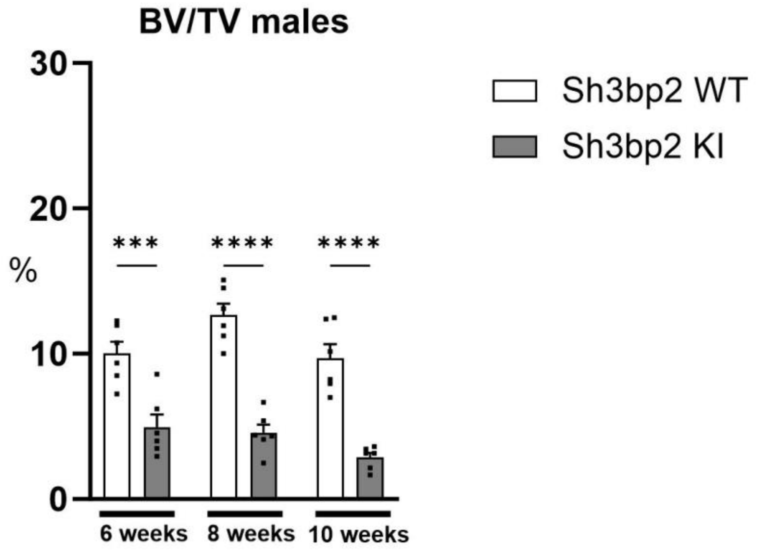

**P253**

**Chronic nonbacterial osteitis (CNO) complicated by progressive inflammatory tibial periostitis**

*Anne Leerling*[1,2], *Natasha Appelman-Dijkstra*[1], *Elizabeth Winter*[1]
[1]*Leiden University Medical Center, Internal Medicine- Division of Endocrinology- Center for Bone Quality, Leiden, Netherlands ;* [2]*Leiden University Medical Center, Clinical Epidemiology, Leiden, Netherlands*

**Abstract Text**

**Background/introduction:** Chronic nonbacterial osteitis (CNO) is a rare disease spectrum characterized by sterile bone inflammation. Beside the core feature of osteitis, CNO patients may exhibit a variety of other inflammatory manifestations. We present an unusual case of adult CNO that featured tibial periostitis. While periostitis typically has traumatic or infectious origins, in the present case it was considered autoinflammatory and secondary to CNO.

**Case description:** A 64 year-old female presented at our expert clinic for follow-up of CNO, which was diagnosed 5 years prior based on radiologically proven osteitis of the manubrium, clavicles, and upper ribs and palmoplantar pustulosis. As there is no proven therapy for CNO, she received our standard but off-label therapy of intravenous pamidronate in 3-monthly intervals combined with on-demand non-steroidal anti-inflammatory drugs (NSAIDs) which led to clinical remission of bone pain. During follow-up, the patient reported weight-bearing pain and functional limitation in the right ankle, where she had sustained a fracture 6 years prior. Physical examination was unremarkable, but imaging revealed a tibial periosteal reaction with bone marrow edema, indicative of periostitis

(**figure 1**). Initially, the periostitis was deemed to be post-traumatic. However, pain and radiologic changes progressed despite repetitive conservative therapy with six weeks of immobilization therapy, ultimately resulting in inability to walk. Prednisone at an initial dose of 30 mg daily was started, which induced a quick complete clinical response. Tapering below 20 mg was hindered by recurring weight-dependent pain, prompting the addition of adalimumab. Adalimumab further improved ankle pain and mobility and led to cessation of prednisone and full restoration of physical functioning.

**Conclusions:** Besides traumatic and infectious factors, tibial periostitis may manifest as an auto-inflammatory phenomenon associated with systemic immune-mediated conditions like CNO. In such instances, anti-inflammatory treatment may be effective.

**P254**

**RUNX2-related metaphyseal dysplasia with maxillary hypoplasia: a rare skeletal disorder resembling SFRP4-related Pyle disease**

*Ewa Hordyjewska-Kowalczyk*[1], *Wim Wuyts*[2], *Nele Boeckx*[2], *An Verdonck*[3,4], *Gretl Hendrickx*[1], *Geert Mortier*[1,5]
[1]*KU Leuven, Department of Human Genetics- Laboratory for Skeletal Dysplasia Research, Leuven, Belgium ;* [2]*Antwerp University Hospital and University of Antwerp, Department of Medical Genetics, Edegem, Belgium ;* [3]*KU Leuven, Department of Oral Health Sciences - Orthodontics, Leuven, Belgium ;* [4]*University Hospitals Leuven, Service of Dentistry, Leuven, Belgium ;* [5]*University Hospital Leuven, Centre for Human Genetics, Leuven, Belgium*

**Abstract Text**

**Background**: Metaphyseal dysplasia with maxillary hypoplasia with(out) brachydactyly (MDMHB – OMIM# 156510) is an ultra-rare disorder that has been reported in only four families to date. MDMHB is characterised by dental anomalies, midfacial hypoplasia, widening of the clavicles, metaphyseal flaring and cortical thinning of the long bones. It may be associated with brachydactyly, osteoporosis and short stature. Heterozygous intragenic duplications within *RUNX2* have been identified in all cases, and they comprise either exons 3 to 5 or exons 3 to 6 and therefore duplicate the glutamine-alanine and an intact Runt (DNA-binding) domains of RUNX2, explaining their gain-of-function effect.

**Methods**: Phenotypic assessment of the proband consisted of a clinical evaluation and radiographic examination. Genetic analyses included whole exome sequencing, Multiplex Ligation-dependent Probe Amplification, cDNA analysis and direct sequencing of the exon junctions.

**Results**: We report on a fifth MDMHB family with one affected individual. Clinical and radiographic examination revealed midface hypoplasia, dental anomalies, enlarged clavicles, genua valga and metaphyseal flaring and thin cortices with an osteoporotic skeletal appearance. The proband showed a striking phenotypic resemblance to *SFRP4*-related Pyle disease. Exome sequencing identified a *de novo* heterozygous tandem duplication within *RUNX2*, encompassing exons 3 to 7. This duplication is larger than the ones previously reported since it extends into the C-terminal activation domain of RUNX2.

**Conclusion**: We describe a new case of *RUNX2*-related MDMHB and highlight the phenotypic resemblance of MDMHB with Pyle disease. We identified a unique *de novo* heterozygous intragenic tandem duplication of exons 3 to 7 of *RUNX2* as the disease-causing variant in the proband. This study extends our knowledge of the genotypic and phenotypic characteristics of MDMHB and *RUNX2*-related bone disorders.

**P255**

**Leukocyte adhesion deficiency type III and osteoclast dysfunction: a case report**

*Yael Levy-Shraga*[1], *Sarah Malkiel*[2], *Etai Adam*[2]
[1]*The Edmond and Lily Safra Children's Hospital- Sheba Medical Center, Pediatric Endocrinology and Diabetes Unit, Ramat Gan, Israel ;* [2]*The Edmond and Lily Safra Children's Hospital- Sheba Medical Center, Department of Pediatric Hemato-Oncology, Ramat Gan, Israel*

**Abstract Text**

**Background:** Leukocyte adhesion deficiency (LAD) III is a rare primary immune deficiency syndrome caused by a defect in neutrophil adhesion to the endothelium. Additional manifestations are bleeding diathesis and osteoclast dysfunction. The disorder is caused by mutations in *FERMT3*, which encodes the kindlin-3 protein. This cytoplasmic protein is highly expressed in hematopoietic cells, and is essential for the integrin-mediated hematopoietic cell adhesion.

**Aim:** To present a nonsense mutation in *FERMT3* in a patient with LAD III, specifying its role in osteoclast function.

**Presenting problem**: A five-year-old patient was presented to our hospital for hematopoietic stem cell transplantation (HSCT). She was born in another hospital by Cesarian section at term to healthy consanguineous parents. On her first day of life, she had bleeding episode from her mouth and was transferred to NICU for treatment and evaluation. Genetic testing confirmed the diagnosis of LAD III due to a homozygous *FERMT3* mutation (NM_001382362, c.687G>A; p.Try229X). By the age of five, she had several infections (skin, urinary tract, respiratory and osteomyelitis), as well as frequent bleeding episodes. To note, her X-RAYs didn't show osteopetrosis.

**Clinical management:** A matched related donor HSCT was performed. Five days after engraftment she developed symptomatic hypercalcemia up to 13 mg/dl. she was treated with hydration, furosemide and calcitonin.  Calcium level decreased but eight days later reached 11.8 mg/dl and she was treated with zoledronic acid (0.012 mg/kg). Since then, calcium levels remained in the normal range.

**Discussion:** This case highlights the effect of homozygous *FERMT3* mutation on the bone. The patient presented with the typical clinical picture of LAD III deficiency, i.e. recurrent infections and bleeding. Before the HSCT, she did not have clinical or radiological signs of osteopetrosis. However, hypercalcemia following HSCT is typical of osteopetrosis secondary to engraftment of functioning osteoclasts differentiated from the donor-derived hematopoietic precursors.

**P256**

**Imiglucerase, Cholecalciferol, and Bone-diet in Skeletal Health Management of Type I Gaucher Disease Patients: a Pilot Study and a Systematic Review.**

Anita Vergatti[1], Veronica Abate[1], Antonio Barbato[1], Gianpaolo De Filippo[2], Domenico Rendina[1]
[1]University of Naples- Federico II, Clinical Medicine and Surgery, Naples, Italy ; [2]Assistance Publique-Hôpitaux de Paris- Hôpital Robert-Debré, Service d'Endocrinologie-Diabétologie, Paris, France

**Abstract Text**

**Introduction:** The skeletal anomalies represent a characteristic feature of Gaucher disease type 1 (GD1). We evaluate the impact of an integrated therapy with Enzyme Replacement Therapy (GD-ERT), cholecalciferol, and a normocalcemic-normocaloric-hyposodic diet (bone diet) on bone health in GD1 patients. We also performed a systematic review to compare our results with available data.

**Methods:** From January 1st,2015 to February 28th, 2019, all GD1 patients referring to Federico II University were enrolled and treated with the integrated therapy. The bone turnover markers (BTMs) and bone mineral density (BMD) were evaluated at baseline (T0) and after 24 months (T24). The systematic review was performed according to PRISMA criteria.

**Results:** We enrolled 25 GD1 patients, all showing 25OHD levels <50 nmol/l (hypovitaminosis D) at T0. The response to cholecalciferol treatment was effective, showing a direct relationship between 25OHD levels before and after treatment. At T0, 2 GD1 patients showed frailty fractures, 5 the Erlenmeyer flask deformity, 3 osteonecrosis and 7 BMD Z-score <-2. Overall, GD1 patients with bone anomalies showed higher C-terminal telopeptide levels compared to those without bone anomalies. During the 2-years follow-up no new bone anomalies occurred. At T24, the BMD remain stable in the entire study cohort and in patients with bone anomalies. The systematic review showed that our study is the first that evaluated all bone health parameters.

**Conclusion:** In GD1 patients, hypovitaminosis D is prevalent. The response to cholecalciferol treatment is effective but different to those previously observed in healthy subjects and in patients with metabolic bone disorders. The integrated therapy including GD-ERT, cholecalciferol, and bone diet guarantee the bone health.

**P257**

### Case Report: High osteoid accumulation in an iliac crest biopsy of an ARHR2 patient

Simon Von Kroge[1], Alexander Spiro[2], Birgit Schipper[3], Michael Amling[1], Ralf Oheim[1]
[1]University Medical Center Hamburg-Eppendorf, Department of Osteology and Biomechanics, Hamburg, Germany ; [2]Altona Children's Hospital, Department of Pediatric Orthopaedic Surgery, Hamburg, Germany ; [3]Altona Children's Hospital, Department of Pediatric Endocrinology, Hamburg, Germany

**Abstract Text**

**Background:** Deactivating mutations in *Enpp1*, the gene encoding ectonucleotide pyrophosphatase phosphodiesterase 1, cause autosomal recessive hypophosphatemic rickets type 2 (ARHR2) in children. Although the clinical / radiological phenotype in patients and the histological phenotype in Enpp1-deficient mouse models have been analyzed in detail, the effect of Enpp1 deficiency on bone cells and tissue mineralization in ARHR2 patients is still unknown.

**Purpose**: This case report aims to histologically characterize the bone mineralization in a patient with ARHR2 to validate analyzed mouse models and provide a clinical reference for this severe skeletal manifestation.

**Methods:** A transiliac crest biopsy was obtained from a 13-year-old female patient, which has previously been characterized clinically and radiologically, harboring a homozygous mutation in *Enpp1* causing ARHR2. At the time of the biopsy, the patient was receiving conventional treatment with phosphate and calcitriol supplements. Undecalcified histological sections of the sample were stained with von Kossa / van Giesson and toluidine blue, histomorphometrically evaluated, and compared to an age- and sex-matched healthy control.

**Results:** Compared to the control, the bone volume fraction (BV/TV) in primary and secondary spongiosa of the patient was decreased by 19.6 % and 32.3 %, respectively. Further, osteoid volume (OV/BV) was increased by 870 % and 542 %, while the number of osteoblasts and osteoclasts per bone perimeter were in range of the control. Interestingly and in contrast to findings in Enpp1-deficient mice, the number of osteocytes per bone area was decreased in the ARHR2 patient by 39.8 % and 71.4 % for primary and secondary spongiosa, respectively.

**Conclusion**: This first histological analysis of an ARHR2 patient underlines the detrimental effect of Enpp1 deficiency on bone mass and mineralization in patients with ARHR2, although osteoblast count was normal. The immense osteoid accumulation and normal cellular parameters correlate with observations in mouse models thereby validating these in terms of osteological research.

**P258**

**Describing the clinical characteristics of patients living with fibrodysplasia ossificans progressiva (FOP): a non-interventional cohort in the USA**

*Hind Harrak[1], Alexander Breskin[1], Victor O. Popoola[1], Susan Rhee[1], Amal Souttou[1], Michelle Davis[2], Caitlin Knox[1]*
*[1]Regeneron Pharmaceutials- Inc., None, Tarrytown, United States ; [2]International Fibrodysplasia Ossificans Progressiva Association, None, North Kansas City, United States*

**Abstract Text**

**Background:** FOP is an extremely rare disease with a prevalence estimated at 1.36 per million people in France and 0.88 per million in the USA. It is a severely disabling genetic condition characterised by progressive ossification of soft tissues. There is limited information on the natural history of FOP.

**Purpose:** To describe the demographics, clinical characteristics and medication use in people living with FOP in the USA.

**Methods:** A descriptive retrospective cohort study of people with a confirmed FOP diagnosis who were part of the International FOP Association (IFOPA) US membership database, and who consented to have a deidentified tokenization and linkage with HealthVerity Marketplace data (NCT06064656).

A descriptive analysis of demographic attributes was performed at cohort entry. Additionally, period prevalence was estimated for the clinical characteristics of interest (e.g. hematologic disorders, respiratory disorders, infections, fall-related injuries, neurologic, mood or dermatologic disorders, neoplasms, cardiovascular diseases, etc.) from 1 January 2018 to 31 December 2022. Medication use was estimated as the period prevalence of patients having a prescription and the median number of prescriptions per patient.

**Results:** Sixty-six patients met the cohort eligibility and had a mean follow-up time of 2.8 years. At cohort entry, the median age was 22 years old, ranging from 1 to 69 years; 54.5% of patients were female. Over the study period, more than 50% of the cohort had at least one or more of the following: neurologic, dermatologic and mood disorders, fall-related injuries and hearing loss. Over half of patients received a prescription for at least one or more of the following: systemic corticosteroids, antibacterial, anti-inflammatory and anti-rheumatic products, psycholeptics and psychoanaleptics.

**Figure 1. Estimated prevalence of clinical characteristics identified in people living with FOP, 1 January 2018 to 31 December 2022**

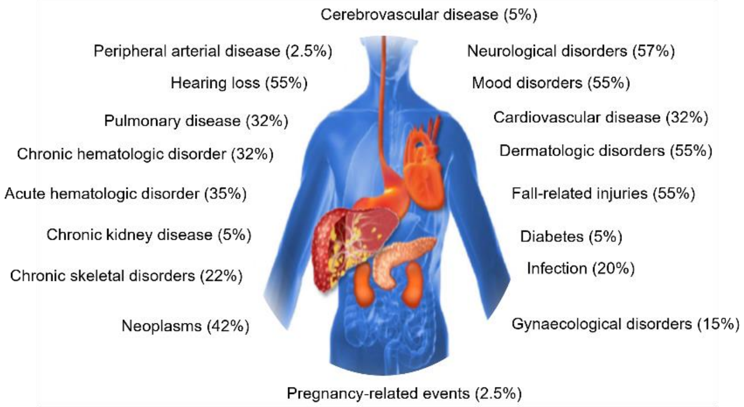

**Conclusion:** People living with FOP have high morbidity and are burdened by multiple chronic and acute disorders beyond the expected musculoskeletal impact caused by the disease.

**P259**

**Epidemiology of fibrodysplasia ossificans progressiva (FOP) mortality in the USA, 2017–2023**

*Victor O. Popoola[1], Alexander Breskin[1], Hind Harrak[1], Amal Souttou[1], Susan Rhee[1], Michelle Davis[2], Sammi Kile[2], Caitlin Knox[1]*
*[1]Regeneron Pharmaceuticals- Inc., None, Tarrytown, United States ; [2]International Fibrodysplasia Ossificans Progressiva Association, None, North Kansas City, United States*

**Abstract Text**

**Background**: FOP is an ultra-rare, severely disabling genetic disease characterised by progressive multi-focal heterotopic bone formation of skeletal muscles, ligaments, tendons and fascia. Limited information is reported on the measurable mortality risk of FOP.

**Purpose**: To estimate annual and period mortality, median survival age, and years of potential life lost (YPLL-80) among patients with FOP in the USA.

**Methods**: In collaboration with the International FOP Association (IFOPA), data sources included de-identified, aggregated data from the IFOPA US membership directory and the FOP Registry from 1 January 2017 until 30 November 2023. Annual mortality and overall mortality during the study period were estimated and expressed per 1000 persons. Additionally, we estimated YPLL and the median age of survival.

**Results**: Of 242 persons identified within the IFOPA data in 2017, 51.9% were female, with a mean age of 26.8 years (range, 3 months–79 years). During the study period, eight patients entered the cohort via birth and there were 35 deaths. The median age of death was 50 years (range 10–76 years) and 22.8% of deaths occurred before 30 years of age. Per 1000 population, age-adjusted annual mortality varied from 19.6 to 76.7, compared to 7.15 to 8.80 in the general US population.

All-cause mortality was 190.1 per 1000 population. Per 1000 persons, YPLL-80 ranged from 519.2 to 1075.9 versus 110.03 for the US general population (2020). Median age of survival was 51 years (95% confidence interval: 47–58) (**Figure**), compared to the general US population life expectancy of 76.4 years (NCHS, 2022).

**Figure. Median Age of Survival Among Patients with FOP (2017–2023)**

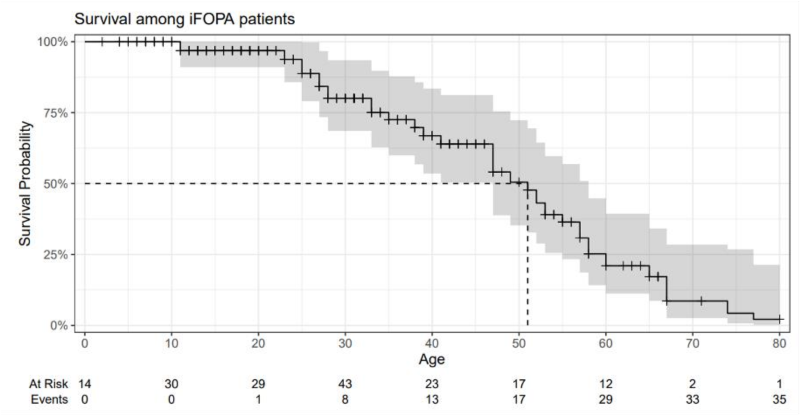

**Conclusion**: Our findings demonstrate that the FOP population may have substantial excess mortality compared to the US general population. Therapies are needed to address this significant mortality risk.

**P260**

**Variation in circulating levels of Periostin in Osteogenesis Imperfecta**

*Alexandre Mercier*[1]*, Evelyne Gineyts*[2]*, Marjorie Millet*[2]*, Olivier Borel*[2]*, Maxime Auroux*[1]*, Elisabeth Sornay-Rendu*[2]*, Pawel Szulc*[2]*, Elisabeth Fontanges*[3]*, Jean-Charles Rousseau*[2]*, Roland Chapurlat*[1]
[1]*Hospices Civils de Lyon - Université Lyon 1, Rheumatology - INSERM Unit 1033 - Hôpital Edouard Herriot, Lyon, France ;* [2]*UFR de Médecine Lyon-Est, INSERM Unit 1033, Lyon, France ;* [3]*Hospices Civils de Lyon, Rheumatology - Hôpital E. Herriot, Lyon, France*

**Abstract Text**

**Background:** Periostin (POSTN) interacts with type 1 collagen and the Wnt-β-catenin pathway, so it may play a role in the pathophysiology of osteogenesis imperfecta (OI). Higher POSTN levels have been associated with the risk of fractures in postmenopausal women. There is no biomarker for the severity of OI, and POSTN stands out as a promising candidate due to its straightforward measurement in serum through a standardized method.

**Objectives:** The primary aim is to investigate whether there is dysregulation of POSTN in OI. The secondary objective is to determine if POSTN levels correlate with the severity and various clinical and biological characteristics of the disease.

**Methods:** This is a secondary analysis of 60 adult patients from the miROI study cohort, including OI types 1 and 3. These patients were compared to 60 control individuals matched for age, sex, and body mass index. The measurements were made using an ELISA in serum samples. We used a t-test for the primary comparison, and Pearson's correlation coefficient was computed to identify potential correlations between serum POSTN levels and clinical and biological characteristics.

**Results:** Serum POSTN levels were significantly higher in the OI group compared to the control group (mean = 798 vs. 712 ng/mL; p = 0.01). There was no correlation with severity variables, such as the number of severe prevalent fractures, the ratio of height to wingspan, or the severity of scoliosis. However, a significant association with the presence of dentinogenesis imperfecta (DI) (mean = 819 vs. 685 ng/mL; p = 0.03) was observed.

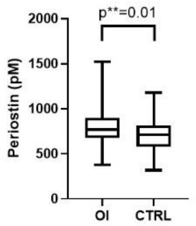

**Figure 1:** Box plot of the difference in circulating serum periostin levels between the Osteogenesis Imperfecta (OI) group and the control group (CTRL). P-value was calculated using a t-test

**Conclusion:** We have found that serum POSTN levels were higher in OI than in controls and were especially associated with the presence of DI. Considering POSTN role in collagen stability and in dentinogenesis, it may be considered as a factor in the pathophysiology of OI and a potential biomarker.

**P261**

**Altered growth plate morphology but normal bone matrix mineralization in the LmnaG609G/G609G mouse model of progeria**

*Stéphane Blouin*[1,2], *Markus A. Hartmann*[1,2], *Nadja Fratzl-Zelman*[1,2], *Phaedra Messmer*[1], *Charlotte Strandgren*[3], *Daniel Whisenant*[4], *Michael R. Erdos*[5], *Francis S. Collins*[5], *Maria Eriksson*[4], *Wayne A. Cabral*[5], *Thomas Dechat*[1,2]
[1]*Ludwig Boltzmann Institute of Osteology at the Hanusch Hospital of OEGK and AUVA Trauma Centre Meidling, 1st Medical Department Hanusch Hospital, Vienna, Austria ;* [2]*Vienna Bone and Growth Center, n/a, Vienna, Austria ;* [3]*Karolinska Institute, Department of Oncology-pathology, Solna, Sweden ;* [4]*Karolinska Institute, Department of Biosciences and Nutrition, Huddinge, Sweden ;* [5]*National Institutes of Health, Molecular Genetics Section- Center for Precision Health Research- National Human Genome Research Institute, Bethesda, United States*

**Abstract Text**

Hutchison-Gilford progeria syndrome (HGPS) is a rare disease caused by a mutation in *LMNA* encoding A-type lamins leading to premature aging and severely reduced life span. HGPS is a multi-systemic disorder characterized by growth deficiency, early loss of subcutaneous fat and muscle with development of sclerotic dermis, alopecia and atherosclerosis. Patients also develop a bone phenotype with reduced lumbar BMD by DXA, osteolysis in distal phalanges, clavicles and mandible and striking demineralization of long bones with slender diaphyses.

To better understand the development and progression of bone tissue alterations in HGPS, we characterized bone mineralization in the *Lmna*$^{G609G/G609G}$ progeria mouse model.

Femora from 8-week-old (8W) *Lmna*$^{+/+}$ (n=20) and *Lmna*$^{G609G/G609G}$ (n=18) and humeri from 15-week-old (15W) *Lmna*$^{+/+}$ (n=7) and *Lmna*$^{G609G/G609G}$ (n=7) mice were analyzed by quantitative backscattered electron imaging to assess bone mineralization density distribution, osteocyte lacunae sections (OLS) and structural bone histomorphometry. Thin sections were stained with Giemsa and Goldner trichrome for histologic evaluation.

*Lmna*$^{+/+}$ and *Lmna*$^{G609G/G609G}$ mice have similar mineral content showing both a gradual shift towards lower mineralization density from cortical diaphyseal to epiphyseal and metaphyseal bone, reflecting different site-specific tissue age. The OLS analysis does not reveal statistical differences between genotypes. However, bone histomorphometry shows decreased BV/TV in primary (8W: -23%, p=0.001; 15W: -38%, p=0.002) and secondary spongiosa (8W: -36%, p=0.001; 15W: -49 %, ns), paralleling a growth plate dysplasia with thinner, unmineralized resting and proliferative zones in the *Lmna*$^{G609G/G609G}$ mice versus controls (8W: -18%, p=0.006; 15W: -25%, p=0.001). Accordingly, histology shows narrow growth plates with thinner zones of unmineralized and mineralized cartilage in the mutants.

In conclusion, the *Lmna*$^{G609G/G609G}$ mouse presents as a chondrodysplasia with reduced trabecular bone volume. Our mineral content findings at several tissue and animal ages suggest that the dysplasia results from impaired bone formation, whereas turnover is unaffected.

**P262**

**Modified cell-matrix organization in osteogenesis imperfecta mice contributes to long bone fragility**

*Grégoire André[1], Antoine Chretien[1], Pierre-Louis Docquier[2], Catherine Behets[1]*
*[1]UCL, MORF, Brussels, Belgium ; [2]UCL, Nmsk, Brussels, Belgium*

**Abstract Text**

**Introduction:** Osteogenesis imperfecta (OI) is an uncommon congenital bone disorder often resulting from a mutation in one of the type I collagen genes. It is characterized by frequent fractures and bone deformities.

**Purpose:** The study examined the organization of collagen and the arrangement of osteocyte lacunae in the long bones of 17-week-old wildtype (WT, n = 17) and osteogenesis imperfecta mice (OIM, n = 16), in order to explore their potential contribution to bone fragility.

**Methods:** Fracture incidence was documented through *in vivo* scanning at weeks 5, 11, and 17. After euthanasia at week 17, diaphyses of humerus, femur, and tibia from both groups underwent *ex vivo* pQCT, histology, and Nano-CT analyses. The animal procedures followed Belgian Federal Law for Animal Care and were approved under reference 2021/UCL/MD/047.

**Results:** The OIM displayed an increasing number of fractures from age 5 to 17 weeks; the cumulative rate remained steady over time with an increase of 3.4 ± 1.6 fractures per mouse between 5 and 11 weeks and of 3.1 ± 2.2 fractures between 11 and 17 weeks. The bone mineral density of the humerus was significantly lower in OIM than in WT mice but not in the other two bones. Collagen birefringence was lower in OIM than WT femurs, in both cross-section (- 49.06%; p< 0.0001) and longitudinal section (-54.59%; $p < 0.01$). The osteocyte lacuna density was higher in the OIM humerus than in the WT ones (+80.01%; $p < 0.001$).

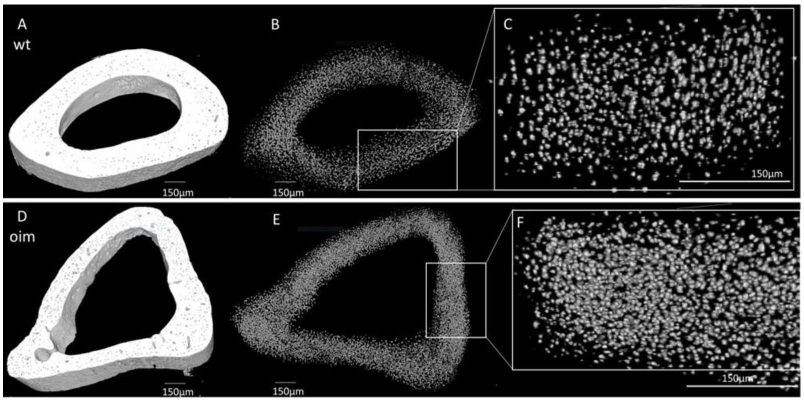

**Conclusion:** Qualitative and quantitative alterations were highlighted in the arrangement of collagen and osteocyte lacunae of OIM long bones, suggesting an immature, woven organization of the bone matrix, which could contribute to bone fragility.

**P263**

**PI3K inhibition reduces heterotopic ossification and restores muscle regeneration**

*Nicole Constanza Farfán*[1]*, Alexandre Deber*[1]*, Francesc Ventura*[1]
[1]*University of Barcelona, Department of Physiological Sciences, Barcelona, Spain*

**Abstract Text**

Heterotopic ossification (HO) is the pathological development of ectopic endochondral bone within soft tissue. HO happens following either mechanical trauma, burns or congenitally in patients suffering from fibrodysplasia ossificans progressiva (FOP). FOP patients carry a mutation (R206H) in the ACVR1 gene that trigger a neomorphic signaling cascade in response to activin A. Our group already verified that BYL719, an inhibitor of phosphatidylinositol 3-kinase-α (PI3Kα) prevents HO. Upon injury, healthy Muscle Stem Cells (MuSC) are the main cells that proliferate, differentiate and fuse to create new myotubes and repair the muscle tissue. In FOP, R206H mutation is believed to increase fibroadipogenic progenitor (FAPs) proliferation. Through close communication, increased FAPs numbers in FOP injuries would decrease MuSCs differentiation while producing the first stages of chondrogenesis and osteogenesis, needed for HO. Here, we show how *in vitro* BYL719-mediated PI3K inhibition reduces mesenchymal progenitors (MP) proliferation. In addition, we show *in vivo* how BYL719 is able to reduce heterotopic bone formation in Acvr1$^{R206H}$ mice. Furthermore, we show how BYL719 not only reduces HO and the number of FAPs within the muscle fibers, but also recovers myotube size and, thus, promotes regeneration. Taken together, this work provides evidence highlighting the importance of the not much known MuSC-FAPs intercommunication and its role in the prevention of ectopic bone formation in HO.

**P264**

**PI3Kα inhibitors block osteoprogenitor specification and the inflammatory response to prevent heterotopic ossification**

*Alexandre Deber[1], Jose Antonio Valer[1], Nicole Constanza Farfan[1], Francesc Ventura[1]*
*[1]University of Barcelona, Department of Physiological Sciences, Barcelona, Spain*

**Abstract Text**

Fibrodysplasia ossificans progressiva (FOP) is one of the causes of the pathological development of ectopic endochondral bone, commonly referred to as heterotopic ossification (HO). FOP patients suffer from an autosomic dominant mutation (R206H) in the *ACVR1* gene, which leads to a neomorphic signaling cascade in response to activin A. Our group previously demonstrated that BYL719 can prevent HO via phosphatidylinositol 3-kinase-α (PI3Kα) inhibition. We further studied the effects of BYL719 over osteoprogenitor specification and inflammatory responses, two of the main FOP drivers. PI3Kα inhibition prevented osteoprogenitor differentiation through on-target inhibition of PI3Kα. However, BYL719 is able to inhibit further HO in mice lacking PI3Kα. This data suggests that BYL719 is able to abolish additional mechanisms beyond osteoprogenitor specification. Therefore, we studied the effects of BYL719 in the inflammatory responses of several immune cell types involved in FOP. BYL719 treatment reduced the proliferation, migration and expression of pro-inflammatory cytokines in monocytes, macrophages and mast cells. Altogether, this work provides evidence that supports BYL719 as a promising new therapeutic strategy for HO by reduction of the osteoprogenitor specification and the inflammatory response in FOP lesions.

**P265**

**Identification of onco-suppressor activity of SWAP-70: implications for new therapeutic target in osteosarcoma**

*Laura Di Giuseppe[1], Jacopo Di Gregorio[2], Sara Terreri[3], Giulia Battafarano[3], Olivia Pagliarosi[3], Jessica Pepe[4], Salvatore Minisola[4], Andrea Del Fattore[3], Michela Rossi[3]*
*[1]"La Sapienza" University of Rome, Department of Clinical- Internal- Anaesthesiology and Cardiovascular Sciences- Policlinico Umberto I, Rome, Italy ; [2]University of L'Aquila, Department of Biotechnology and Applied Clinical Sciences, L'Aquila, Italy ; [3]Bambino Gesù Children's Hospital- IRCCS, Bone Physiopathology Research Unit- Translational Pediatrics and Clinical Genetics Research Division, Rome, Italy ; [4]"La Sapienza" University of Rome, Department of Clinical- Internal- Anaesthesiology and Cardiovascular Sciences- Policlinico Umberto I, Rome, Italy*

**Abstract Text**

**Background**: Osteosarcoma is a highly malignant skeletal tumor, particularly prominent among adolescents and young adults. Although surgery and chemotherapy are available for patients, there is a need for innovative therapeutic strategies for drug resistance and chemotherapy-associated side effects.

**Purpose**: Our study aims to unravel the role of SWAP-70, a phosphatidylinositoltrisphosphate (PIP3) binding protein, in osteosarcoma.

**Methods**: Gene and protein expression, mineralization activity, proliferation, cell migration, invasion, apoptosis, osteoclastogenesis were evaluated by *in vitro* assays.

**Results**: To identify the implications of SWAP-70 in tumor progression, we examined its expression in different cell types: mesenchymal stem cells (MSC), osteoblasts and Saos-2 osteosarcoma cells. Our findings demonstrated an increase of SWAP-70 levels during the differentiation of MSC into osteoblasts, while in Saos-2 cells was undetected. Therefore, we transfected Saos-2 cells with a vector overexpressing SWAP-70 (pSWAP-70) fused with GFP protein or an empty plasmid (E). The overexpression of SWAP-70 resulted in an increase of cells differentiation, as shown by the upregulation of RUNX2, SP7, ALP, DMP1 and MEPE, and a simultaneous two-fold increase of the ability to form mineralized nodules. Additionally, pSWAP-70-Saos-2 showed reduced proliferation rate [Proliferative rate; E: 1.000±0.007, pSWAP-70: 0.815±0.119. **p<0.01], decreased migration [Wound closure (%); E: 100.00±1.00, pSWAP-70: 52.11±20.76. *p<0.05] and invasion abilities [Absorbance (%); E: 100.00±1.00, pSWAP-70: 62.54±11.26. **p<0.01] compared to empty vector transfected cells. No substantial changes between pEmpty- and pSWAP-70-transfected cells were observed in the apoptosis induced by H2O2 treatment. Finally, a 47% decrease of osteoclastogenesis was observed in coculture experiments between pSWAP-70-Saos-2 and monocyte/macrophage RAW264.7 cells compared to the levels detected in pEmpty-Saos-2/RAW264.7 cocultures.

**Conclusions**: Our results strongly indicate that the overexpression of SWAP-70 induces osteoblast differentiation of osteosarcoma cells, implying a potential role of SWAP-70 as an onco-suppressor and proposing it as a novel target to set new therapeutic approaches for patients.

**P266**

**Neuropathic and nociplastic pain are common in adult chronic nonbacterial osteitis (CNO)**

*Anne Leerling*[1,2], *Marieke Niesters*[3], *Marcel Flendrie*[4], *Marije Tel*[1], *Natasha Appelman-Dijkstra*[1], *Olaf Dekkers*[2,5], *Elizabeth Winter*[1]
*[1]Leiden University Medical Center, Internal Medicine- Division of Endocrinology- Center for Bone Quality, Leiden, Netherlands ; [2]Leiden University Medical Center, Clinical Epidemiology, Leiden, Netherlands ; [3]Leiden University Medical Center, Department of Anesthesiology- Center for Pain Treatment, Leiden, Netherlands ; [4]Sint Maartenskliniek, Department of Rheumatology, Nijmegen, Netherlands ; [5]Leiden University Medical Center, Internal Medicine- Division of Endocrinology, Leiden, Netherlands*

**Abstract Text**

**Background:** Chronic nonbacterial osteitis (CNO) is a rare bone disease characterized by sterile bone inflammation causing bone pain. However, other mechanisms beyond nociceptive inflammatory pain may be involved in patients' pain experience. We investigate the prevalence of neuropathic and nociplastic pain profiles in adult CNO and assess their association with clinical characteristics and treatment outcomes.

**Methods:** Survey study among the Dutch adult CNO cohort. Patients were invited to participate in surveys about pain profiles, including PAIN-detect for neuropathic pain, Central Sensitization Inventory (CSI), Fibromyalgia Rapid Screening Tool (FiRST), and ACTTION-APS Pain Taxonomy (AAPT) scoring for nociplastic pain and symptoms consistent with fibromyalgia. Clinical characteristics and CNO-related pain scores assessed by Brief Pain Inventory were compared between patients with nociceptive pain exclusively and those with nociceptive pain plus neuropathic and/or nociplastic pain (mixed pain profile).

**Results:** 80 patients participated in the surveys (response rate 44%). 31% (95% CI 21-41) of patients classified as "likely (>90% probability)" having neuropathic pain according to PAIN-detect. 53% (95% CI 41-64) of patients displayed degrees of sensitization consistent with a nociplastic pain profile. 61%

(50-72) screened positive for fibromyalgia on FiRST and 14% (7-23) of patients fulfilled the AAPT criteria for fibromyalgia. The presence of neuropathic and/or nociplastic pain (mixed pain profile) was associated with longer diagnostic delay (mean difference 2.8 years, 95% CI 0.4-5.2, p=0.023), lower educational level (72% versus 20%, p<0.001), and opioid use for CNO-related symptoms (37% versus 13%, p=0.036). Despite comparable disease duration and extent, patients with a mixed pain profile reported significantly higher CNO-related bone pain scores.

**Conclusion and discussion:** This study demonstrates the frequent coexistence of neuropathic and nociplastic pain alongside nociceptive inflammatory bone pain in adult CNO patients. Disease burden in CNO may extend beyond just inflammatory activity, highlighting the need for a multifaceted management approach.

**P267**

**The role of physical therapy in adult chronic nonbacterial osteitis (CNO): patients' and therapists' perspective**

*<u>Anne Leerling</u>[1,2], David Ueckert[3], Florus Van der Giesen[4], Thea Vliet Vlieland[5], Elizabeth Winter[1]*
*[1]Leiden University Medical Center, Internal Medicine- Division of Endocrinology- Center for Bone Quality, Leiden, Netherlands ; [2]Leiden University Medical Center, Clinical Epidemiology, Leiden, Netherlands ; [3]Leiden University Medical Center, Physical Therapy, Leiden, Netherlands ; [4]Zuyderland Ziekenhuis, Rheumatology, Heerlen, Netherlands ; [5]Leiden University Medical Center, Orthopaedics-Rehabilitation and Physical Therapy, Leiden, Netherlands*

**Abstract Text**

**Background:** Chronic nonbacterial osteitis (CNO) is a rare bone disease causing bone pain and functional impairment. We map the use, perceived effects, and preferences regarding the provision of physical therapy (PT) for adult CNO from the patients' and first-line therapists' perspective.

**Methods:** Cross-sectional study among the unique Dutch adult CNO cohort (spanning 1992–present). An existing survey on PT-use for axial spondylarthritis was adapted for CNO, covering the current PT-use and/or use in the past 24 months and reasons for non-use if applicable. PT-users were asked about treatment modalities, perceived health-related effects, satisfaction (scale 1-10) and preferences for future use. Their physical therapists were invited to complete a self-developed survey covering therapy details and preferences and needs.

**Results:** 80/195 and 14/16 patients and therapists completed the survey respectively. 41 (51%) patients had used PT for CNO in the preceding 2 years. Reasons for non-use included prior ineffectiveness of PT (n=14/39, 36%), lack of physician recommendation (n=12/39, 31%), and cost concerns (n=6/39, 15%) (multiple answers possible). PT-users reported diverse methods, commonly involving passive therapy like massage (n=25/41, 61%) and joint mobilizations (n=18/41, 44%), and active therapy like muscle strengthening (n=17/41, 42%) and breathing exercises (n=20/41, 49%). PT-users were mostly, but variably satisfied (median satisfaction score 8/10, range 1-10). 51/80 (64%) of patients desired greater emphasis from their clinicians regarding PT-value. Therapists reported to appreciate referral letters with CNO-specific information (n=13/14, 93%), and consultations with second-line specialized therapists (n=12/14, 86%).

**Conclusions and discussion:** Half of adult CNO patients currently use or recently used PT, which involves variable therapeutic methods. The majority of patients desire more clinical emphasis on PT, and the majority of therapists prefer more CNO-specific information to support their care for this rare disease. Future studies should investigate the effectiveness of CNO-tailored exercise programs to define the role of PT in CNO-management.

**P268**

**Multidisciplinary recommendations for the diagnosis and management of chronic nonbacterial osteitis (CNO) in adults**

*Elizabeth Winter[1], Olaf Dekkers[2,3], Anne Leerling[1,2], Caroline Andreasen[4], Natasha Appelman-Dijkstra[1], Simone Appenzeller[5], Gunter Assmann[6], Judith Bubbear[7], Oana Bulaicon[1], Roland Chapurlat[8], Varvara Choida[9,10], Gavin Clunie[11], Salvatore D'Angelo[12], Dimitrios Daoussis[13], Torsten Diekhoff[13], Marcel Flendrie[14], Olivier Fogel[15,16], Roba Ghossan[16], Hermann Girschick[17], Femke Van Haalen[1], Neveen Hamdy[1], Barbara Hauser[18,19], Christian Hedrich[20], Philip Helliwell[21], Kay Geert A. Hermann[22], Antonella Insalaco[23], Anne G. Jurik[24], Mitsumasa Kishimoto[25], Willem F. Lems[26], Paivi Miettunen[27], Burkhard Muche[28], Ana Navas Cañete[29], Natalia Palmou[30], Frits Smit[31,32], James Teh[33], Charlotte Verroken[34], Kurt De Vlam[35], Daniel Wendling[36], Wei Zhou[37], Hans-Georg Zmierczak[38]*
*[1]Leiden University Medical Center, Internal Medicine- Division of Endocrinology- Center for Bone Quality, Leiden, Netherlands ; [2]Leiden University Medical Center, Clinical Epidemiology, Leiden, Netherlands ; [3]Leiden University Medical Center, Internal Medicine- Division of Endocrinology, Leiden, Netherlands ; [4]Gødstrup Hospital, Rheumatology and Internal Medicine, Herning, Denmark ; [5]School of Medical Science- Universidade Estadual de Campinas, Rheumatology and Traumatology-, Campinas, Brazil ; [6]Ruhr-Universität Bochum, Rheumatology and Clinical Immunology, Bochum, Germany ; [7]Royal National Orthopaedic Hospital Stanmore, Rheumatology, Stanmore, United Kingdom ; [8]INSERM UMR 1033- Université Claude Bernard-Lyon 1, Rheumatology, Lyon, France ; [9]Homerton Healthcare NHS Foundation Trust, Rheumatology, London, United Kingdom ; [10]University College London, Centre for Medical Imaging, London, United Kingdom ; [11]Cambridge University Hospitals- NHS Foundation Trust, Rheumatology, Cambridge, United Kingdom ; [12]San Carlo Hospital of Potenza, Rheumatology of Lucania, Potenza, Italy ; [13]University of Patras Medical School, Rheumatology, Patras, Greece ; [14]Sint Maartenskliniek, Rheumatology, Nijmegen, Netherlands ; [15]Paris Saint-Joseph Hospital, Rheumatology, Paris, France ; [16]Cochin Hospital AP-HP, Rheumatology, Paris, France ; [17]Vivantes Clinic Berlin Friedrichshain, Pediatrics, Berlin, Germany ; [18]Western General Hospital, Diseases Unit- NHS Lothian, Edinburgh, United Kingdom ; [19]MRC Institute of Genetics and Cancer- Western General Hospital, Centre for Genomic and Experimental Medicine, Edinburgh, United Kingdom ; [20]University of Liverpool, Women's and Children's Health, Liverpool, United Kingdom ; [21]Leeds Institute of Rheumatic and Musculoskeletal Medicine- University of Leeds, Rheumatology, Leeds, United Kingdom ; [22]Charité Universitätsmedizin Berlin, Radiology, Berlin, Germany ; [23]Ospedale Pediatrico Bambino Gesù, Rheumatology, Rome, Italy ; [24]Aarhus University Hospital, Radiology, Aarhus, Denmark ; [25]Kyorin University School of Medicine, Nephrology and Rheumatology, Tokyo, Japan ; [26]Amsterdam University Medical Center, Rheumatology, Amsterdam, Netherlands ; [27]McCaig Institute for Bone and Joint Health, Pediatrics, Calgary, Canada ; [28]Charité Universitätsmedizin Berlin, Rheumatology, Berlin, Germany ; [29]Leiden University Medical Center, Radiology, Leiden, Netherlands ; [30]Hospital Universitario Marqués de Valdecilla, Rheumatology, Santander, Spain ; [31]Leiden University Medical Center, Radiology- division of Nuclear Medicine, Leiden, Netherlands ; [32]Alrijne Ziekenhuis, Nuclear Medicine, Leiderdorp, Netherlands ; [33]Nuffield Orthopaedic Centre, Radiology, Oxford, United Kingdom ; [34]Unit for Osteoporosis and Metabolic Bone Diseases - Ghent University Hospital, Endocrinology, Ghent, Belgium ; [35]Leuven University Hospital, Rheumatology, Leuven, Belgium ; [36]Université de Franche-Comté CHU, Rheumatology, Besançon, France ; [37]Beijing Medical University Hospital, Rheumatology, Beijing, China ; [38]University Hospital Ghent, Rheumatology, Ghent, Belgium*

**Abstract Text**

**Introduction:** Adults with sterile bone inflammation are variably labelled as Synovitis, Acne, Pustulosis, Hyperostosis, Osteitis (SAPHO)-syndrome, chronic nonbacterial osteomyelitis, chronic recurrent multifocal osteomyelitis, pustulotic arthro-osteitis, or sternocostoclavicular hyperostosis. A standardized diagnostic approach for this rare disease is lacking, and clinical management is challenging due to limited available evidence.

**Objectives:** To develop consensus recommendations for the disease definition, diagnosis and management of adults with sterile bone inflammation, integrating existing evidence and multidisciplinary expert opinion.

**Methods:** The consensus initiative comprised a systematic literature review and appraisal of evidence, two Delphi surveys and digital and a two-day in-person consensus meeting with a multidisciplinary expert panel and patient representatives.

**Recommendations:** A consensus disease definition was developed, and agreement was reached among panel members and patient representatives to uniformly use the diagnostic term "chronic nonbacterial osteitis (CNO)" for adults. Considerations for the initial evaluation of suspected adult CNO patients were formulated, including laboratory investigations, bone biopsies, and imaging, the latter taking a crucial role in expediting diagnosis. Regarding management, treatment goals and outcomes were outlined with active involvement from patient representatives. A treatment algorithm for adults with symptomatic CNO was includes first-, second-, and third-line strategies, with specific attention for the heterogeneity of the CNO disease spectrum, and how individual patient factors influence medication preferences. Consensus advice for follow-up, management of adverse events and complications was devised, as well as a research agenda.

**Conclusions and future perspectives:** This first international and multidisciplinary set of recommendations offers clinical guidance on the terminology, diagnosis, and management of adults with sterile bone inflammation, i.e. CNO. This consensus initiative will contribute to the timely recognition of adult CNO, improve clinical care, and facilitate future systematic and collaborative research.

**P269**

**Raman spectroscopy sheds light on bone composition of animal models of Autosomal Recessive Osteopetrosis**

*Marco Ventura[1], Maria Lucia Schiavone[2], Federico Vernuccio[1], Morteza Behrouzitabar[1], Alejandro De la Cadena[1], Giuseppe Antonacci[3], Giulio Cerullo[1], Dario Polli[1], Cristina Sobacchi[4], Renzo Vanna[5]*
*[1]Politecnico di Milano, Physics Department, Milan, Italy ; [2]Humanitas Research Hospital IRCCS, Human Genome, Rozzano, Italy ; [3]Specto photonics srl, Milan, Italy ; [4]CNR-IRGB, Milan Unit, Rozzano, Italy ; [5]CNR, Institute for Photonics and Nanotechnologies, Milan, Italy*

**Abstract Text**

**Background**: Autosomal Recessive Osteopetrosis (ARO) is a rare genetic pathology usually diagnosed soon after birth, caused by defects in osteoclast activity or formation, and resulting in increased bone density, deformities, fractures, and secondary haematological and neurological deficits. Different ARO genetic subtypes are known, associated with different severity and prognosis. Imaging investigations are an essential diagnostic tool, able also to reveal major complications. On the other hand, Raman spectroscopy might offer the possibility to study this pathology in a non-invasive way, thus representing an attractive tool for in vivo diagnosis and disease monitoring.

**Methods**: the study was conducted on fixed, non-decalcified bone samples (femur or tibia, vertebrae and skull) from oc/oc mice, as a model of TCIRG1-dependent osteopetrosis, and wild-type littermates; and RANKL KO mice, as a model of RANKL-deficient osteopetrosis, and age-matched wild-type (Table 1). We acquired single point Raman spectra (>1000) and hyperspectral images, using a home-built Raman microspectrometer equipped with a 660nm laser.

**Results**: Hyperspectral maps of deplasticized sections of murine vertebrae showed a higher mineralized tissue area in osteopetrotic bones, but a locally low mineral-to-matrix ratio (Fig. 1A-C). Quantitative analysis on single point spectra demonstrated a significantly lower mineral-to-matrix ratio (p = 10e-30, p = 10e-58) in the bone of both oc/oc and RANKL KO mice compared to the respective WT controls. Crystallinity was significantly lower in oc/oc vs WT (p = 10e-29) without difference in carbonate substitution (p = 0.19). RANKL KO samples showed a significant increase in both crystallinity and carbonate substitution (p = 10e-4, p = 10e-19) (Fig. 1D-E).

**Conclusion**: our results indicate that Raman spectroscopy can be an adequate tool for the direct and non-invasive study of osteopetrosis, able to highlight compositional features inherent to specific subsets of ARO. Thus, our study paves the way to new research and diagnostic perspectives.

**Figure 1.** Hyperspectral Raman maps of tissue sections of **(A)** wild type, **(B)** oc/oc and **(C)** RANKL KO murine vertebra. **(D)** Box plot representing the mineral-to-matrix ratio. **(E)** the crystallinity and **(F)** the carbonate substitution in the bone of mice of the different genotype and age groups. Each data point represents a single spectrum. Each box represents the 25th to 75th percentiles [interquartile range (IQR)]. The whiskers represent the lowest and highest values within the boxes ± 1.5 × the IQR. (***) Calculated p-value <0.001.

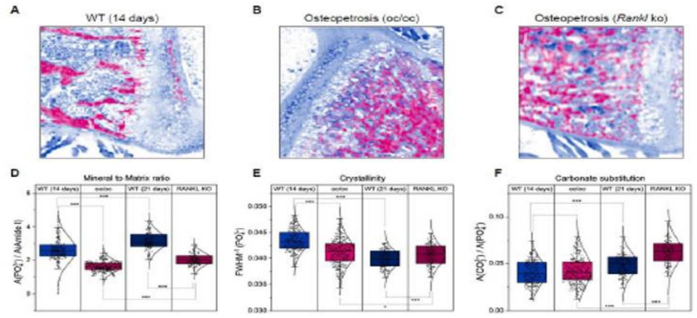

**Table 1. List of samples and measures acquired**

| Mouse model | Osteopetrosis subtype | Age | N° of mice | Bones analyzed | N° of Raman spectra |
|---|---|---|---|---|---|
| oc/oc | TCIRG1-deficient | 14-day-old | 6 | skull | 200 |
| | | | | femur | 240 |
| | | | | vertebra# | 125x125=15625# |
| WT | - | 14-day-old | 4 | skull | 120 |
| | | | | femur | 160 |
| | | | | vertebra# | 125x125=15625# |
| Rankl ko | RANKL-deficient | 21-day-old | 3 | skull | 120 |
| | | | | tibia | 120 |
| | | | | vertebra# | 125x125=15625# |
| WT | - | 21-day-old | 3 | skull | 120 |
| | | | | tibia | 120 |

#Representative images of hyperspectral Raman maps are shown in Fig. 1A-C

**P270**

**KIF5B – the culprit in an unclassified skeletal dysplasia?**

*Victoria Sarne[1,2], Anna Huber[1,2], Alexander Beribisky[1], Hannes Steinkellner[1], Richard Arnhold[3], Peter Nikkels[4], Winfried Neuhaus[5,6], Thomas Dechat[7], Franco Laccone[1]*
*[1]Medical University of Vienna, Institute of Medical Genetics, Vienna, Austria ; [2]University of Vienna, Vienna Doctoral School of Pharmaceutical- Nutritional and Sport Sciences PhaNuSpo, Vienna, Austria ; [3]Danube Hospital, Department of Pathology and Bacteriology, Vienna, Austria ; [4]Wilhelmina*

*Children's Hospital- University Medical Center Utrecht and Utrecht University, Department of Pathology, Utrecht, Netherlands ; [5]AIT – Austrian Institute of Technology GmbH, Competence Unit Molecular Diagnostics- Center Health and Bioresources, Vienna, Austria ; [6]Danube Private University, Department of Medicine, Krems, Austria ; [7]Ludwig Boltzmann Institute of Osteology at Hanusch Hospital of OEGK and AUVA Trauma Centre Meidling, 1st Medical Department, Vienna, Austria*

**Abstract Text**

**Background:** The *KIF5B* gene encodes the motor protein Kinesin Family Member 5B (KIF5B), which is ubiquitously expressed and has been implicated in a variety of cellular processes. Recently, three human patients with a specific *KIF5B* mutation (p.K91R) have been reported. One of these patients was a fetus with an unclassifiable skeletal dysplasia registered at the Danube Hospital Vienna, who is the focus of this study.

**Purpose:** The aim of this work is to investigate the relationship between the observed phenotype and the mutation by biochemical assays, as well as *in vitro* and *in vivo* studies. The p.K91R mutation resides within the ATP binding site of the motor domain of the KIF5B protein. Therefore, we hypothesize, that the resulting protein is an immobile rigor mutant with diminished or absent ATP hydrolysis function. Hence, the motor activity of recombinant wildtype and mutant KIF5B proteins was tested by isothermal titration calorimetry and enzyme linked inorganic phosphate assay. The mutant protein showed highly diminished motor activity.

**Methods and Results:** *In vitro,* the chondrogenic differentiation and formation of extracellular matrix in ATDC5 mouse pre-chondrocytes is negatively affected, when p.K91R KIF5B is overexpressed compared to wildtype and empty vector control.

To investigate the effect of the p.K91R mutation on *in vivo* skeletal development, a mouse model carrying the p.K91R mutation, which was generated in our lab, are compared to wildtype littermates regarding their stature, size and presence of skeletal elements as well as bone matrix composition and quality.

**Conclusion:** Overall, the p.K91R mutation has been shown to produce a protein with diminished motor domain activity, which leads to slowed differentiation and extracellular matrix formation in mouse pre-chondrocytes as well as a skeletal phenotype in mice comparable to that observed in humans.

**P271**

**Disruption of the osteocyte lacunocanalicular network in osteogenesis imperfecta type XIV**

*Chloe Jones[1], Stéphane Blouin[1,2], Adalbert Raimann[2,3], Gabriel Mindler[2,4], Barbara M. Contento[5], Roberta Besio[5], Alexandra Stauffer[2,4], Franco Laccone[2,3], Markus A. Hartmann[1,2], Antonella Forlino[5], Nadja Fratzl-Zelman[1,2]*
*[1]Ludwig Boltzmann Institute of Osteology, Hanusch Hospital of OEGK and AUVA Trauma Centre Meidling, Vienna, Austria ; [2]Vienna Bone and Growth Centre, n/a, Vienna, Austria ; [3]Division of Paediatric Pulmonology- Allergology and Endocrinology- Medical University of Vienna, Department of Paediatrics and Adolescent Medicine, Vienna, Austria ; [4]Orthopaedic Hospital Speising, Department of Paediatric Orthopaedics, Vienna, Austria ; [5]University of Pavia, Department of Molecular Medicine-Biochemistry Unit, Pavia, Italy*

**Abstract Text**

Autosomal recessive osteogenesis imperfecta (OI) type XIV is caused by mutations in *TMEM38B* encoding for the ubiquitously expressed endoplasmic reticulum trimeric intracellular cation channel type B (TRIC-B), causing calcium homeostasis and collagen synthesis disruptions. Affected patients present with a highly variable clinical phenotype, including bone fragility and skeletal deformities. Because intact calcium signalling is crucial for proper osteocyte signalling, we hypothesised that osteocyte function and consequently the osteocyte lacunocanalicular network (OLCN) might be altered in OI type XIV. Therefore, we characterised the OLCN in bone specimens from a paediatric patient and a *Tmem38b* conditional knock-out (*Runx2Cre*;*Tmem38b*$^{fl/fl}$;cKO) mouse (Besio et al., Matrix Biol., 2023).

We investigated tibia and fibula bone samples obtained from a 9-year-old boy following corrective surgery and tibiae from 4-week-old male *Tmem38b* cko mice (cKO: n=4, Ctrl: n=6).

The OLCN was stained with rhodamine 6G and imaged using confocal laser scanning microscopy (patient: 16 osteons, murine bone: 6 ROIs/sample). Subsequent network analysis provided the canalicular density (Can.Dn, $\mu m/\mu m^3$), mean lacunae volume (Lc.V, $\mu m^3$), and mean lacunae degree (Lc.Deg, mean number of canaliculi emanating from a single lacuna). We evaluated bone matrix mineralisation in bone specimens using quantitative backscattered electron imaging.

Can.Dn, Lc.V and Lc.Deg were decreased in the patient (tibia: -50.61%, -11.62% and -44.72% versus healthy reference), and in cKO (-17.67%, -32.47%, -35.23% versus Ctrl). In contrast, the mean calcium concentration of the matrix of the child was within normal range (22.66±0.56 wt% Ca) despite bisphosphonate treatment, but found increased in cKO (+3.7% wt% Ca, p=0.0016).

These findings demonstrate that the OLCN is disrupted in absence of TRIC-B in our patient and the osteoblast (and presumably osteocyte) specific *Tmem38b* cKO murine model. The non-hypermineralised bone matrix in the child with OI type XIV confirms previous findings but contrasts with typical OI hypermineralised murine bone and warrants further clarification.

**P272**

**A clinical case of treating a patient with calvarial doughnut lesions and bone fragility**

*<u>Anton Tyurin</u>[1], Diana Valeeva[1], Rita Khusainova[2]*
*[1]Bashkir State Medical University, Internal medicine, Ufa, Russia C.I.S. ; [2]Bashkir State Medical University- Medical Genetics Department, National Medical Research Centre for Endocrinology-Laboratory of genomic medicine, Ufa, Russia C.I.S.*

**Abstract Text**

Calvarial doughnut lesions with bone fragility (MIM: #126550) is a rare autosomal dominant skeletal disorder characterized by low bone mineral density, spinal and peripheral fractures, and specific sclerotic lesions of the cranial bones. The disease is caused by pathogenic variants c.148C>T (p.Arg50Ter) in the SGMS2 gene.

Proband is a 30-year-old woman, the first fracture of the right femur was revealed shortly after birth, and the second fracture of the same bone was sustained at the age of one year. Throughout her life, multiple fractures of the upper and lower limbs (about 20) have been registered. An increase in bone pain syndrome not related to physical activity is noted. At the age of 27, pamidronate acid therapy was resumed, at which point the severity of the pain syndrome was maximal, with a Z- criterion of the lumbar spine -2.4. During the therapy, the patient reported a decrease in the severity of the pain syndrome, and also an increase in bone mineral density at various times, with the Z- criterion ranging from 0.3 to 1.4. In addition to bisphosphonates, from November 2019 to November 2020, the patient received teriparatide in a standard dosage. After November 2021, there was a break in bisphosphonate therapy due to the coronavirus pandemic. Since the beginning of 2022, the patient has reported a recurrence of the pain syndrome, despite normal bone mineral density levels (Z=0.8). Pamidronate acid therapy was resumed with a positive therapeutic effect. The dynamics of pain syndrome indicators and bone mineral density levels during the therapy are presented in Figure 1 (red - severe pain, yellow - moderate, green - no pain).

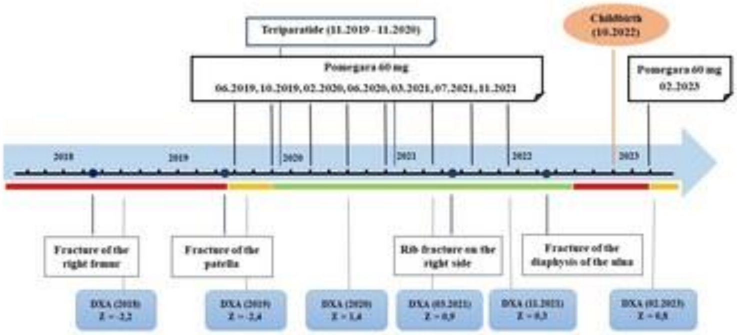

Thus, bisphosphonate therapy not only improves bone density in patients with calvarial doughnut lesions, but also reduces the intensity of pain syndrome.

**P273**

**Heterotopic ossification in Fibrodysplasia ossificans progressiva involves the activation of Activator Protein-1 signaling in response to Activins.**

*Marius Wits[1], Alexandre Deber López[2], Jose Antonio Valer Ratera[2], Francesc Ventura Pujol[2], Marie-Jose Goumans[1], Gonzalo Sanchez-Duffhues[3]*
*[1]Leiden University Medical Center, Cell and Chemical Biology, Leiden, Netherlands ; [2]Universitat de Barcelona- IDIBELL, Department de Ciències Fisiològiques, Barcelona, Spain ; [3]Spanish National Research Council CSIC/Nanomaterials and Nanotechnology Research Center CINN, Health Research Institute of the Principality of Asturias ISPA, Oviedo, Spain*

**Abstract Text**

**Background/introduction:** Patients with Fibrodysplasia Ossificans Progressiva (FOP) experience progressive heterotopic ossification (HO) of connective soft tissues, leading to severe immobility and cardiorespiratory failure. Disease development is linked to mutations in ACVR1, which encodes the Bone Morphogenetic Protein (BMP) type I receptor ALK2, with the predominant heterozygous single-point mutation c.617G>A; p.R206H. The mutant ALK2 FOP receptor signals via canonical BMP signaling involving SMADs in response to circulating Activins. Therapeutic strategies targeting ALK2 and/or Activins effectively prevent HO but also compromise the physiological functions of the Transforming Growth Factor (TGF)-β pathway. However, non-canonical signaling pathways induced by Activins in FOP, potentially contributing to HO, remain poorly understood, representing a novel druggable target in FOP.

**Purpose**: To investigate how Activins regulate non-canonical ALK2 signaling in FOP and to identify novel cellular druggable targets not compromising ALK2 and/or Activins physiological functions.

**Methods**: Bulk RNA transcriptomics and phosphoproteomics assays in human MSCs (mesenchymal stromal cells, n=4) over-expressing ALK2 R206H. *In vitro* functional differentiation assays (by histochemical staining and qPCR analysis) in isogenic control and FOP MSCs differentiated from induced pluripotent stem cells (iPSCs) (n=4). Immunohistochemical stainings, weight monitoring in FOP mice (n=15).

**Results**: In response to Activins, FOP cells display a distinct phosphoproteome compared to cells expressing wild-type ALK2, including the activation of several cellular inflammatory kinases ($p<0.05$). Activins specifically induce the expression of Activator Protein-1 family genes in vitro and in FOP mice ($p<0.001$). The repurposed AP-1 inhibitor T-5224 (20 µM) prevents chondrogenic differentiation in FOP iPSC-derived MSCs ($p<0.05$) and is well-tolerated (30 mg/kg) in FOP mice.

**Conclusion**: *In vitro* and *in vivo*, AP-1 is specifically induced in FOP bone progenitor cells, where it contributes to chondrogenic differentiation. Pharmacological blockade of AP-1 using T-5224 may represent a novel therapeutic strategy for FOP, without interfering with the physiological functions of ALK2 and/or Activins.

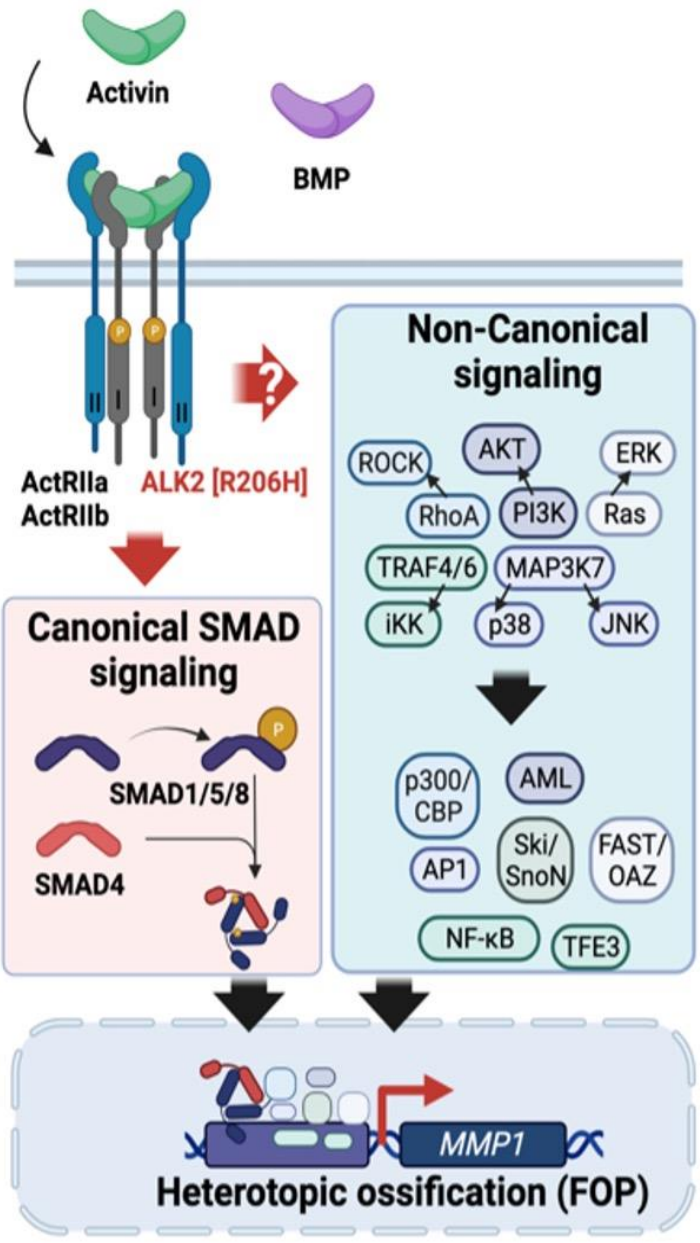

**P274**

**Cellular alterations caused by KIF5B mutants associated with bone pathologies**

*Julian Umprecht[1], Michelle Schweiger[1], Johanna Besold[1], Victoria Sarne[2,3], Silvia Spitzer[1], Thomas Dechat[1,4]*
*[1]Ludwig Boltzmann Institute of Osteology at the Hanusch Hospital of OEGK and AUVA Trauma Centre Meidling, 1st Medical Department Hanusch Hospital, Vienna, Austria ; [2]Center for Pathobiochemistry and Genetics- Medical University of Vienna, Institute of Medical Genetics, Vienna, Austria ; [3]Vienna Doctoral School of Pharmaceutical- Nutritional and Sport Sciences, n/a, Vienna, Austria ; [4]Vienna Bone and Growth Center, n/a, Vienna, Austria*

**Abstract Text**

Kinesin family member protein 5B (KIF5B) is a microtubule associated motor protein responsible for the transport of various cargos to the cellular periphery by hydrolysing ATP. In addition, KIF5B is also involved in the organization of the microtubule network within a cell and in the distribution of various cellular organelles, such as the Golgi apparatus and lysosomes. KIF5B is composed of a motor domain (MD) responsible for the movement, a stalk domain (SD) necessary for homo-dimerization and a tail which associates with a kinesin light chain and subsequently with cargo.

Recently mutations in the MD and in the SD of KIF5B have been associated with various pathologic human conditions. Interestingly, while SD mutations were mainly associated with neurodevelopmental disorders, MD mutations resulted in bone pathologies resembling skeletal dysplasias.

The aim of this study is to better understand the common and distinct disease causing mechanisms associated with the various KIF5B mutants. In addition, we aim to understand why MD mutants cause different pathologies in humans than SD mutants. Therefore, the respective mutants were overexpressed in model cell lines for bone research such as human osteosarcoma (U2OS) and murine mesenchymal stroma (D1) cells. Transfected cells were analysed by immunofluorescence microscopy using antibodies against various organelle markers (e.g. Golgin97, TOM20 and LAMP1) and against ß-tubulin.

We found that that while most of the MD mutants led to microtubule-bundling, wild type KIF5B and the SD mutants did not affect the microtubule network dramatically. However, the distribution of lysosomes and the Golgi apparatus was altered even by wild type KIF5B, suggesting that controlled KIF5B expression levels are critical. Based on our data we speculate that MD mutants act dominant negatively blocking microtubule tracks also for other proteins and mutations in the SD lead to a loss of function of KIF5B.

**P275**

**The combination of height loss and back pain which severely limits normal activities are highly predictive of pregnancy associated osteoporosis**

*Elizabeth Orhadje[1], Kathryn Berg[1], Karen Whitehead[1], Barbara Hauser[2], Stuart Ralston[1]*
*[1]University of Edinburgh, Centre for Genomic and Experimental Medicine, Western General Hospital-Edinburgh, United Kingdom ; [2]NHS Lothian, Rheumatic Diseases Unit, Western General Hospital-Edinburgh, United Kingdom*

**Abstract Text**

**Introduction:** Pregnancy associated osteoporosis (PAO) is a rare condition with a prevalence of 4/100,000 women. It usually presents with back pain due to multiple vertebral fractures, but the diagnosis is often delayed. Women with PAO report that they are often told that back pain is normal during pregnancy and given false reassurance that there is nothing to be concerned about.

**Purpose:** We aimed to identify clinical features that differentiated individuals with PAO from women of a similar age who were pregnant but had not been diagnosed with PAO.

**Methods:** Individuals with a self-reported diagnosis of PAO completed an online questionnaire about their presentation which gathered information on back pain, height loss and severe pain interfering with their normal activities, and other factors. They were invited to nominate a friend who was pregnant around the same time but did not have PAO to act as a control. The sensitivity and specificity of each feature in identifying PAO was assessed using SPSS, and cases and controls were compared using Fisher's exact tests.

**Results:** We studied 152 cases and 124 controls. The average age at the time of pregnancy was about 32 years. Back pain was present in 86.8% of cases *vs.* 44.4% of controls, height loss in 50.7% *vs.* 0% and severe pain in 83.2% *vs.* 1.6% ($p<0.0001$ for all features). The sensitivity of back pain in identifying PAO was 86.8% but specificity was low at 55.6%. Sensitivity for height loss was 50% and specificity 100%; for severe pain, sensitivity was 83.2% and specificity 98.4%. When all three features were present, sensitivity was 89.6% and specificity 100%.

**Conclusion:** The combination of back pain, height loss, and severe pain has high sensitivity and specificity for identifying people with PAO. Measurement of height at booking of pregnancy should be routinely implemented to help facilitate diagnosis of PAO.

**P276**

**Beyond Vitamin D: Unraveling Bone Health Complexities in Hypophosphatasia**

*Mani Sangar*[1], *Simran Kaur*[1], *Sanjay Kumar Bharata*[1], *Liza Das*[1], *Vandana Dhiman*[1]
*[1]Post Graduate Institute of Medical Education & Research, Endocrinology, Chandigarh, India*

**Abstract Text**

**Introduction/Purpose:** Disorders affecting the mineralization of bones disrupt the process essential for bone strength due to imbalances in calcium and phosphorus deposition or removal. Hypophosphatasia (HPP) results from TNSALP enzyme deficiency, presenting symptoms from mild dental issues to severe skeletal problems, disrupting bone mineralization due to imbalances in calcium and phosphorus.

**Methods:** The online repository, the rare metabolic bone disease registry of India (rarembd.in) serves as a comprehensive database for individuals diagnosed with rare metabolic bone diseases (MBD) undergoing assessment and treatment at a specific tertiary healthcare facility. Over 13 years (2010-2023), patient data collected from this source were examined to investigate epidemiological trends, clinical manifestations, genetic factors, and how skeletal structures are affected.

**Results:** There were a total of 6 patients with hypophosphatasia (all females) in the registry. The cohort's mean age was 47.1 ± 12.9 years (range 32-71). At diagnosis, the mean serum alkaline phosphatase was 42.8 ± 13.9IU/L (range 23-58). The mean serum calcium was 9.2 ± 0.3mg/dl, inorganic phosphate was 3.7 ± 1.2mg/dl and 25(OH)D was 46 ± 14.6ng/ml, with all but one patient having vitamin D sufficiency (>30ng/ml). Among bone turnover markers, serum P1NP was 31.4 ± 17.9ng/ml and CTX was 205.9 ± 46.4pg/ml. Osteoporosis was present most commonly at the lumbar spine (2/6), followed by the femoral neck (1/5) and none at the distal radius. TBS analysis showed a mean TBS of 1.23 ± 0.04 and partially degraded microarchitecture in all but 1 patient.

**Conclusion:** In six female hypophosphatasia patients, despite sufficient vitamin D, low alkaline phosphatase indicated enzyme deficiency. Bone turnover markers revealed increased resorption, and reduced formation, notably causing osteoporosis in the lumbar spine. The analysis emphasized compromised bone structure beyond vitamin D in hypophosphatasia.

**P277**

**Micropetrosis and heterogenous bone mineralization pattern in an adult patient with Pycnodysostosis**

*Nadja Fratzl-Zelman*[1,2]*, Stéphane Blouin*[1,2]*, Markus A. Hartmann*[1,2]*, Andreas A. Kurth*[3]*, Jochen Zwerina*[1,2]
[1]*Ludwig Boltzmann Institute of Osteology at the Hanusch Hospital of OEGK and AUVA Trauma Center Meidling, 1st Medical Department Hanusch Hospital, Vienna, Austria ;* [2]*Vienna Bone and Growth Center, n/a, Vienna, Austria ;* [3]*Center for Orthopaedics and Trauma Surgery, Marienhaus Klinikum Mainz, Mainz, Germany*

**Abstract Text**

**Background/Introduction:** Pycnodysostosis is an ultra-rare skeletal dysplasia caused by biallelic loss-of-function mutations in cathepsin K, a lysosomal protease highly expressed by osteoclasts. Defective cathepsin K leads to dysfunctional bone resorption, progressive osteosclerosis and bone fragility. Moreover, cathepsin K is also expressed by mature osteocytes. Whether the number, size and viability of osteocytes as well as the osteocyte lacunocanalicular network (OLCN) are also altered in pycnodysostosis and thereby contribute to bone fragility has not been explored yet.

**Methods:** In this study, we used light microscopy, quantitative backscattered electron imaging and confocal laser scanning microscopy to examine bone material obtained from a 57-year-old female patient who underwent surgical correction after femoral head fracture.

**Results:** High number of multinucleated osteoclasts and fringes of demineralized matrix were viewed on bone surfaces. The trabecular volume BV/TV was elevated, varying locally from 30.3% to 67.4% and the matrix was hypermineralized (average calcium content: +7.5% versus reference values, with a fivefold increase of highly mineralized areas >27wt% calcium). The cortex consisted of a compact shell of parallel ordered fibrils, reflecting vigorous periosteal bone formation and multiple osteons with concentrically ordered lamellae. Density (number/$mm^2$: 193 to 223) and mean area (20 $\mu m^2$) of osteocyte lacunae and density of canaliculi (0.05 $\mu m/\mu m^3$bone volume) were within normal range. However, numerous bone packets exhibited elevated number of mineralized lacunas (micropetrosis) resulting in a local disruption of the OLCN.

**Conclusions:** In summary, our data indicate that in pycnodysostosis not only osteoclast are dysfunctional but also osteocyte viability is decreased, leading to enhanced micropetrosis and a very heterogenous bone mineralization pattern. Thus, the function of both, osteoclasts and osteocytes might be impaired, contributing to lower bone quality. Nevertheless, the presence of an ordered and dense osteocyte network in large areas of the sample indicates that cathepsin K is not essential for the formation of the OLCN.

**P278**

**Generation and characterization of the first mouse model of Cole Carpenter Syndrome.**

*Elisa Pucci[1], Luciana Silvestri[1], Piergiorgio Patrizii[1], Antonella Stoppacciaro[2], Paolo Mené[2], Anna Teti[1], Antonio Maurizi[1]*
*[1]University of L'Aquila, Department of Biotechnological and Applied Clinical Sciences, L'Aquila, Italy ; [2]University Sapienza, Department of Clinical and Molecular Medicine, Rome, Italy*

**Abstract Text**

The Cole Carpenter Syndrome (CCS) is a neglected autosomal dominant disease affecting bone, which becomes brittle. CCS is caused by the heterozygous p.Y393C mutation in the *P4HB* gene encoding for the protein disulfide isomerase A1 (PDIA1). To date, there are no studies showing how the mutation affects the skeletal. Based on this, we generated a mouse model (CCS mouse) carrying the mouse homolog aminoacidic substitution *tyr*395*cys*. Bone phenotype, assessed by µCT in CCS mice showed marked osteopenia compared to the WT counterpart (p<0.05). In addition, the Indentation Distance was dramatically increased (+1.7fold;p=0.03) in CSS femurs compared to WT, indicating a bone fragility. At the subcellular level, Western blot and immunofluorescence analyses conducted on cellular models carrying the *P4HB*$^{Y393C}$ revealed a reduction in type I collagen expression (-50%;p=0.009) and secretion (-70%;p=0.01) along with a higher PDIA1 stabilization and Bip1 expression (+3 fold;p=0.05) compared to WT cells suggesting the presence of ER stress. PDIA1$^{Y393C}$ subcellular localization was unchanged. Similar results were obtained in primary CCS mouse osteoblasts. In line with this, in vivo serum PINP1 levels, a marker of type I collagen turnover in the bone matrix, were lower in CCS mice (-60% vs WT;p<0.01). Alteration in the type I collagen path and cellular trafficking was further confirmed by proteomic analysis in CCS bone (p<0.05). Finally, metabolic tests revealed a significant reduction of blood glucose levels in CCS male mice compared to WT (-14%;p<0.05), along with an unchanged glucose and insulin tolerance. This finding could be linked to renal defects found in CSS mice consisting of tubular vacuolization and glomerular shrinkage. Our data indicate the presence of a severe bone phenotype in *CCS* mice due to defective type 1 collagen biosynthesis, consistent with the skeletal features of the human disease, and the pathogenic involvement of organs beyond the bone.

**P279**

**Osteoporosis in patients with indolent systemic mastocytosis is influenced by age, body mass index and bone formation**

*Ngan Nguyen[1], Martina Blaschke[1,2], Annette Lamersdorf[2], Gabriele Lehmann[2], Heide Siggelkow[1,2]*
*[1]University Medical Center Göttingen, Clinic of Gastroenterology- Gastrointestinal Oncology and Endocrinology, Göttingen, Germany ; [2]MVZ Endokrinologikum, Medivision Trägergesellschaft, Göttingen, Germany*

**Abstract Text**

Indolent systemic mastocytosis (ISM) is characterized by an aggregation of mast cells in the skin or other organs. Mast cell infiltration of bone is often followed by low trauma spine fractures.

To investigate bone affection in ISM, we performed a single-center, prospective cross-sectional study and included 24 ISM patients and 15 age matched controls (DRKS00030069). General patient information, blood samples for a number of investigations, and 24-hour-urine samples were collected. Bone mineral density (BMD) and fracture history were documented. For analysis of clinical disease severity, two questionnaires for quality of life (SF-36; German pain questionnaire) and two MC-specific questionnaires (MC-QoL; MAS) were completed. The study was approved by the ethical committee.

ISM patients were characterized with Osteoporosis (n=17, T-score<-2,5 and/ or fractures), Osteopenia/Normal BMD (n=9, T-score >-2.5 and no fractures), and compared to age- and gender matched controls (n=15). ISM patients without osteoporosis were significantly younger (55± 12 years of age) and had a higher BMI (31 kg/m$^2$±5) compared to ISM patients with osteoporosis (64±11 years of age 25kg/m$^2$±5). Laboratory values including tryptase, histamine, parathyroid hormone, bone resorption markers, calcium and vitamin D metabolism and urinary values were not different between those with and without osteoporosis. However, bone formation measured by bone alkaline phosphatase (BAP) was significantly higher (p=0.046) in ISM patients with osteoporosis. The clinical disease burden determined by general and disease specific questionnaires demonstrated no differences between the two ISM patient groups.

In conclusion, ISM patients with osteoporosis are older, have a lower BMI, and higher BAP values. Our data suggest, that ISM patients get osteoporosis between 55 and 64 years of age, are at higher risk with low BMI and that BAP is the best bone turnover marker for monitoring. Further analysis of study material hopefully will give further insight in bone affection in this disease.

**P280**

**The European Registries for Rare Bone and Mineral Conditions (EuRR-Bone): Collecting Core Data Elements and Clinician and Patient Reported Outcomes**

*Mariya Cherenko[1], Ana Luisa Priego Zurita[1], Inês Alves[2], Manila Boarini[3], Maria Luisa Brandi[4], Corinna Grasemann[5], Martine Cohen-Solal[6], Wolfgang Högler[7], M Kassim Javaid[8], Agnès Linglart[9], Klaus Mohnike[10], Marina Mordenti[3], Tess M De Rooij[1], Marco Roos[11], Luca Sangiorgi[3], Rebecca Skarberg[12], Ondrej Soucek[13], S. Faisal Ahmed[14], Natasha M. Appelman-Dijkstra[1]*

*[1]Leiden University Medical Centre, Department of Medicine- Division of Endocrinology, Leiden, Netherlands ; [2]Ando Portugal, ERN BOND ePAG Representative, Evora, Portugal ; [3]IRCCS Istituto Ortopedico Rizzoli, Department of Rare Skeletal Disorders, Bologna, Italy ; [4]Fondazione FIRMO Onlus- Fondazione Italiana per la Ricerca sulle Malattie dell'Osso, Donatello Bone Clinic- Casa di Cura Villa Donatello- Sesto Fiorentino, Florence, Italy ; [5]Ruhr-University Bochum, Department of Pediatrics- St. Josef Hospital, Bochum, Germany ; [6]Inserm U1132 Bioscar and Université Paris-Cité, Center Viggo Petersen Hôpital Lariboisière, Paris, France ; [7]Johannes Kepler University Linz, Department of Paediatrics and Adolescent Medicine, Linz, Austria ; [8]University of Oxford, Nuffield Department of Orthopaedics- Rheumatology and Musculoskeletal Sciences, Oxford, United Kingdom ; [9]Le Kremlin Bicetre, AP-HP- Paris Saclay University Department of Pediatric Endocrinology And Diabetology for Children, Paris, France ; [10]Otto-Von-Guericke-Universitaet Magdeburg, kinderklinik, Magdeburg, Germany ; [11]Leiden University Medical Centre, Department of Human Genetics, Leiden, Netherlands ; [12]Osteogenesis Imperfecta Federation Europe OIFE, ERN BOND ePAG Representative, Oslo, Norway ; [13]harles University and Motol University Hospital, Department of Paediatrics Second Faculty of Medicine, Prague, Czech Republic ; [14]University of Glasgow, Developmental Endocrinology Research Group- Royal Hospital for Children, Glasgow, United Kingdom*

**Abstract Text**

**Introduction:** The European Registries for Rare Bone and Mineral Conditions (EuRR-Bone) were created in collaboration with the European Reference Network on Rare Bone Diseases (ERN BOND)

and on Rare Endocrine Conditions (Endo-ERN). The Core Registry collects a set of Core Data Elements and longitudinal patient- and clinician-reported outcomes.

**Aim:** To describe the data entered between April 2020 and December 2023.

**Methods:** EuRR-Bone clinical contributors register new and existing cases of rare bone and mineral conditions mapped according to the Orphanet classification, collecting core demographic and diagnostic data elements. Condition-specific modules (CSM's) are available for Clinician-Reported (CROMs), and Patient-Reported Outcome Measures (PROMs) are available for patients to complete through the 'patient platform'.

**Results:** To date, 21 centres from 14 European countries contributed 652 cases to the Core Registry, 444 (68%) bone and 208 (32%) mineral conditions. Ninety-nine (15%) were between 0-9 years old, 82 (13%) between 10-17 years and 471 (72%) over 18 years. The median age was 34 years (range 0-86). Six-hundred patients (92%) were under active follow-up. Of 164 patients (25%) who had expressed interest in accessing the patient platform 51 (31%) had an active account. One hundred thirty-four PROMs outcomes were completed, being EQ-5D, WHO ICF (International Classification of Function and Disability) and BPI-SF (Brief Pain Inventory Short Form). PROMs were mostly entered by clinicians (82%). Most reported condition was FD/MAS(n=176), followed by Achondroplasia(n=142), Genetic hyperparathyroidism(n=74) and Osteogenesis Imperfecta(n=61). CSM's are available for some of these conditions. Again, most entrances were for FD/MAS, followed by Achondroplasia and the ECTS/ERN Bond/EuRR-Bone Melorheostosis module.

**Conclusion**: EuRR-Bone has proved its ability to collect CROMS and PROMS in patients of all ages and has proven its long-term sustainability. This makes the platform a suitable option for studying the natural history and long-term clinical outcomes of rare bone and mineral conditions.

**P281**

### Adeno-associated virus type 8 vector encoding TNAP-D10 improves the skeletal phenotype in a mouse model of infantile HPP

*Cintia Kazuko Tokuhara[1], Flavia Amadeu de Oliveira[1], Fatma F. Mohamed[2], Sonoko Narisawa[1], Brian L. Foster[3], Koichi Miyake[4], José Luis Millán[1]*
*[1]Sanford Burnham Prebys Medical Discovery Institute, Human Genetics Program, La Jolla, United States ; [2]University of Michigan School of Dentistry, Biologic and Materials Science & Prosthodontics, Ann Harbor, United States ; [3]The Ohio State University. College of Denstistry, Division of Biosciences, Columbus, United States ; [4]Nippon Medical School, Department of Gene Therapy, Tokyo, Japan*

**Abstract Text**

Hypophosphatasia (HPP) is a rare genetic metabolic disorder, caused by loss of function in the *ALPL* gene encoding tissue-nonspecific alkaline phosphatase (TNAP), that features hypomineralization caused by the accumulation of extracellular inorganic pyrophosphate ($PP_i$), a potent mineralization inhibitor. Asfotase alfa, a mineral-targeting recombinant TNAP, a life-saving enzyme replacement therapy approved for pediatric-onset HPP requires 3 to 6 times subcutaneous injections per week. Here, we aimed to determine the efficacy of a single injection of adeno-associated virus vector serotype 8 harboring TNAP-$D_{10}$ (AAV8-TNAP-$D_{10}$) in improving the skeletal phenotype in the *Alpl*$^{-/-}$ mice, a mouse model of infantile-onset HPP. Three doses, $4x10^8$, $4x10^9$, and $4x10^{10}$ (vg/b) of AAV8-TNAP-$D_{10}$ were injected intramuscularly into 4-day-old *Alpl*$^{-/-}$ and WT littermates and the treatment efficacy was evaluated after 70 days. Biochemical analysis showed elevated serum alkaline phosphatase activity (ALP) and suppressed plasma $PP_i$ levels at $4x10^9$ and $4x10^{10}$ (vg/b) of AAV8-TNAP-$D_{10}$. The achieved serum ALP activity was higher in males than females. There was no significant difference in serum calcium and phosphorus levels in the *Alpl*$^{-/-}$ compared to WT mice. The treatment with AAV8-TNAP-$D_{10}$ improved the skeletal phenotypes in *Alpl*$^{-/-}$ mice at the doses of $4x10^9$ and $4x10^{10}$ when compared to the lowest dose of $4x10^8$ visualized by radiographic images. In addition, the lengths of the long bone of the *Alpl*$^{-/-}$ mice with the $4x10^{10}$ dose were greater than with the lowest doses. There was no ectopic calcification in the soft organs after 70 days of treatment. Our results showed that the lowest tested dose $4x10^8$ was efficient in saving the lives of *Alpl*$^{-/-}$ mice, but it did not fully prevent or correct the bone phenotype. In contrast, the highest dose was sufficient to have a complete prevention of the bone phenotype demonstrating that gene therapy can be a promising alternative to the treatment of HPP.

**ND-P022**

**Structural heart disease in patients with osteogenesis imperfecta**

*Anastasia Mocritcaia[1], Chafik Chacur[1], Eva Gonzalez[2], Marc Ramos-Jovani[3], Ana García-Alvarez[3], Helena Florez[1], Ana Monegal[1], Nuria Guañabens[1], Pilar Peris[1]*
*[1]Hospital Clinic, Rheumatology, Barcelona, Spain ; [2]Hospital Clinic, Molecular Biology- CORE Laboratory, Barcelona, Spain ; [3]Hospital Clinic, Cardiology, Barcelona, Spain*

**Abstract Text**

**Background**: Osteogenesis imperfecta (OI) is a genetic disorder characterized by connective tissue abnormalities with decreased bioavailability of collagen type I (COL1) and bone fragility. While the bone OI phenotype is well described, less is known on the effects of OI in other tissues containing COL1, such as the heart. Heart valves, chordae tendineae, annuli fibrosi, and the interventricular septum contain COL1, indicating that the presence of heart disease should be evaluated in OI.

**Objectives**: Evaluate the prevalence of structural heart disease in a cohort of adults with OI and its relationship with the severity of the disease and the COL1 genotype.

**Methods**: Retrospective study of 28 adult patients diagnosed with OI. Clinical data (type, phenotype and genetic mutation) were obtained from medical records. Laboratory, densitometric, and echocardiographic findings were also collected.

**Results**: Among the 28 patients included (aged 19-78 years old) most had type I OI (26 type I,1 type III,1 type IV), Seven (25%) exhibited echocardiographic alterations: diastolic dysfunction (1), left atrial dilatation (3), aortic root dilatation (1), apical diverticulum (1) and significant valvulopathies (4;14,3%). The former included 3 aortic insufficiencies (2 severe requiring valve replacement and 1 moderate;2/3 of these patients had a bicuspid aortic valve) and a moderate mitral insufficiency. Of the 4 subjects with valvulopathies, all had type 1 OI with several previous skeletal fractures (4-12), blue sclera, and none presented hearing loss or dentinogenesis. The 2 subjects with severe valvulopathies also presented a marked low bone mass (Z-score <-4) and >10 previous fractures. In the genetic test they showed *COL1A1* mutations affecting the glycine amino acid (p.(Gly200Asp) and p.(Gly221Arg), respectively.

**Conclusions:** Patients with OI may present cardiac involvement, especially heart valvular disease. Periodic screening with echocardiography seems essential to identify and manage potential severe heart complications in this disorder. Whether some mutations favor valvular disease needs further studies.

**ND-P023**

**Hypofosfatasia and neuropathic pain: related to intracellular depletion or extracellular accumulation of vitamin B6?**

*Zografia Zervou[1], Evert F.S. Van Velsen[1], M. Carola Zillikens[1]*
*[1]Erasmus Medical Center, Internal Medicine, Rotterdam, Netherlands*

**Abstract Text**

Hypophosphatasia (HPP) is an inherited disorder, due to mutation(s) of the ALPL gene encoding non-tissue specific alkaline phosphatase (TNSALP). A complete or partial lack of enzyme activity results in elevated serum concentrations pyridoxal 5′-phosphate (PLP), the main circulating form of vitamin B6. HPP has been associated with neuropathy, but the specific symptoms and mechanism remains inadequately understood. Here, we describe four HPP patients with neuropathic pain and the potential mechanisms, related to the vitamin B6 metabolism. A 56-year-old woman, with severe childhood HPP, PLP levels of 410nmol/L (35-110nmol/L) and chronic neuropathic pain in her lower limbs, had an impressive decrease in symptoms after starting asfotase alfa, with rapid normalization of serum PLP levels. The second patient, a 28-year-old woman with childhood onset HPP and PLP 999nmol/L, experienced burning pain in her feet for almost 3 years. The neurologist diagnosed neuropathic pain and prescribed nortriptyline which resulted in impressive pain relief. The third patient, a 22-year-old man, with childhood onset HPP and chronic neuropathic pain of his knees, feet and lower limbs, had serum PLP levels of >300nmol/L. He received opioids, NSAIDS and anti-epileptics with little effect on pain. The last patient is a 71-year-old man with adult onset of HPP, PLP 390nmol/L and neuropathic pain in hands and feet since 3 years. His symptoms improved spontaneously. Mechanistically, defective TNSALP activity could cause neuropathic pain by defective myelination due to altered metabolites, such as adenosine, which is involved in myelin synthesis. Alternatively, various cases of polyneuropathy due to vitamin B6 supplementation have been reported, but the mechanism is unknown. In conclusion, we describe 4 patients with HPP and neuropathic pain. The rapid decrease of neuropathic pain in the patient treated with asfotase alfa, concomitantly with normalization of vitamin B6 levels, suggest a potential relationship with vitamin B6 or other metabolic changes.

**ND-P024**

**Evaluation of bone compartments in men with PLS3 variants assessed by HR-pQCT and 3D-DXA.**

*Zografia Zervou[1], Rana Mabrouk[1], Evert F.S. Van Velsen[1], Melissa S.A.M. Bevers[2], Joop P. Van den Bergh[2], M. Carola Zillikens[1]*
*[1]Erasmus Medical Center, Internal Medicine, Rotterdam, Netherlands ; [2]VieCuri Medical Center, Internal Medicine, Venlo, Netherlands*

**Abstract Text**

X-linked osteoporosis due to *PLS3* genetic variants is a rare disease, clinically affecting men more than women. To date, on bone quality of these patients is limited. Our aim was to compare trabecular and cortical volumetric bone mineral density (vBMD) in adults with *PLS3* variants between data from high-resolution peripheral quantitative computed tomography (HR-pQCT) scans and 3D-DXA-based analysis (3D Shaper® software, v2.13.0). Eleven men had HR-pQCT scans at the distal radius and tibia and a DXA scan at the total hip. Trabecular and cortical vBMD from HR-pQCT were evaluated using standard analysis methods and from DXA using 3D Shaper software to allow separate evaluation of cortical and trabecular compartments. Results were compared with age- and sexmatched controls and expressed as *Z*-scores. DXA showed lower areal BMD (aBMD) at the lumbar spine (median *Z*-score:-1.7) than at the femoral neck (*Z*:-1.3) and total hip (*Z*:-1.4) (Table 1). 3D-DXA results revealed a low trabecular vBMD (*Z*:-2.0), while cortical surface BMD was less affected (*Z*:-1.1). Similar results were seen with HR-pQCT at the tibia, with low trabecular vBMD (*Z*:-2.0) and low-normal cortical vBMD (*Z*:-1.2). Results at the radius showed lower values for both trabecular (*Z*:-2.7) and cortical vBMD (*Z*:-1.8). In conclusion, both HR-pQCT and 3D-DXA showed lower trabecular than cortical vBMD in adult men with PLS3 genetic variants. This is consistent with the lower aBMD by DXA at the lumbar spine than at the femoral neck and total hip. It suggests that 3D-DXA could be an easily accessible and alternative tool for HR-pQCT to evaluate 3D cortical and trabecular vBMD in X-linked osteoporosis due to PLS3 genetic variants. Yet, bone microarchitecture cannot be directly assessed in contrast to HR-pQCT.

**ND-P025**

**Real-world experience with burosumab treatment in adults with X-linked hypophosphataemia (XLH) in the UK**

*Richard Keen[1], Robin Lachmann[2], Elaine Murphy[3], Gauri Krishna[4], Gavin Clunie[5], Jennifer Walsh[6], Schini Marian[7], Matthew Roy[8], Leigh Mathieson[9], Victoria Hayes[9], Ben Johnson[10], Daniel Stevens[11], Mark Nixon[12], Judith Bubbear[13]*
*[1]Royal National Orthopaedic Hospital, Consultant Rheumatologist, Stanmore, United Kingdom ; [2]University College Hospital, Consultant in Inherited Metabolic Disease, London, United Kingdom ; [3]University College Hospital, Consultant Physician, London, United Kingdom ; [4]University College Hospital, Medical Doctor, London, United Kingdom ; [5]Cambridge University Hospital Trust-Addenbrookes, Consultant in Rheumatology and Metabolic Bone Disease, Cambridge, United Kingdom ; [6]Medical Doctor, Sheffield Teaching Hospitals, Sheffield, United Kingdom ; [7]Sheffield Teaching Hospitals, Consultant in Metabolic Bone, Sheffield, United Kingdom ; [8]University Hospitals Bristol, Consultant Rheumatologist, Bristol, United Kingdom ; [9]Kyowa Kirin, Medical Affairs, Marlow, United Kingdom ; [10]Kyowa Kirin, Health Economics and Outcomes Research, Marlow, United Kingdom ; [11]Bionical Emas, Medical Affairs, Willington, United Kingdom ; [12]Chilli Consultancy, Analytics, Salisbury, United Kingdom ; [13]Royal National Orthopaedic Hospital, Consultant Rheumatologist/Metabolic Bone, Stanmore, United Kingdom*

**Abstract Text**

**Background/Introduction:** XLH is a rare, genetic, progressive, phosphate-wasting disorder that causes skeletal morbidities, stiffness, pain, and impaired physical function throughout life. Burosumab is a fully human monoclonal antibody that inhibits fibroblast growth factor 23, resulting in increased renal phosphate reabsorption. An early access programme (EAP) was set up to enable access to burosumab for adults with XLH in the UK, providing an opportunity to capture real-world clinical experience with burosumab treatment.

**Purpose:** To describe the baseline characteristics of participants in the EAP and determine the proportion achieving a normal serum phosphate concentration (above lower limit of normal [LLN] according to local reference ranges) and mean change in serum phosphate concentration after 6 months' burosumab treatment.

**Methods:** This retrospective, longitudinal, real-world cohort study involved adults with XLH receiving treatment with burosumab via the EAP at five UK sites. Retrospective data were collected from medical records.

**Results:** A total of 142 patients were enrolled and received at least one dose of burosumab; 136 (96%) had not previously received burosumab. The patients had a mean age of 43.4 years (SD 14.7); 64% were female and 77% were white. At baseline, mean (SD) height was 155.2 (9.3) cm and weight was 73.6 (16.9) kg. Among the burosumab-naïve patients, 5% had serum phosphate concentration >LLN (95% CI 2–10%; n=127) at baseline, increasing to 63% after 6 months' burosumab treatment (95% CI 52–74%; n=82). Their mean serum phosphate concentration was 0.61 (SD 0.16) mmol/L (n=127) at baseline and had increased by 0.28 (0.24) mmol/L after 6 months' burosumab treatment (range −0.54 to 0.95 mmol/L, n=75).

**Conclusions:** Burosumab treatment in adults with XLH in real-world practice is associated with improvements in serum phosphate concentration to within the normal range.

**Poster Sessions: Innovative concepts, new technologies, case reports**

**P282**

**3D bioreactor development for bone cell adhesion studies**

*Kai Böker*[1]*, Linda Gätjen*[2]*, Katarina Vasic*[1]*, Shahed Taheri*[1]*, Christopher Dölle*[2]*, Wolfgang Lehmann*[1]*, Arndt. F. Schilling*[1]
[1]*UMG Göttingen, Department for Trauma Surgery- Orthopaedics and Plastic Surgery, Göttingen, Germany ;* [2]*Fraunhofer IFAM Bremen, Institute for Manufacturing Technology and Advanced Materials, Bremen, Germany*

**Abstract Text**

**Background:** Current research in the field of implant ingrowth is often based on animal experiments. Along with the importance of animal welfare, new advanced in vitro culture systems that mimic the general in vivo environment are needed. In addition, a fundamental understanding of cell adhesion to metal surfaces would help to understand the interaction of the cell with the surface and thus create the possibility of tailoring cell adhesion specifically to the implant.

**Methods:** A 3D analysis system of the implant/tissue interface was established, which allows to mimic the environment near the implant. The material properties of medical steel were modified using LightPLAS coating technology. Bone cell adhesion was tested with lentiviral transduced, RFP-positive MG-63 cells in both a 2D environment and in a FlowBone setup with two different medium-flows.

**Results:** Coated surfaces demonstrated significantly reduced cell attachment, increased circularity and reduced cell area at all tested time points in 2D culture. Low flow conditions lead to 51% reduction of cells after 24h and 57% reduction after 48h compared to uncoated metal surfaces. Circularity of cells on coated surfaces was significant increased by 11% after 48h while cell area remained unaffected compared to non-coated controls for both time points. High flow conditions resulted in an even more strongly reduced cell count after 24h (77% reduction) and 48h (78% reduction). Circularity was increased by 8% and cell area was reduced by 24% after 24h, while no differences were observed after 48h (Tab. 1)

**Conclusion:** We successfully established a 3D bioreactor setup to test cell adhesion in an *in vivo* like environment. We observed a decreased cell adhesion and altered cell phenotype of osteoblasts on

LightPLAS coated surfaces. Our 3D bioreactor system provides the possibility to test cell adhesion under flow conditions, which opens the possibility to reduce animal experiments in the future.

**Table 1:** Mean and SD of relative cell count, circularity and single cell area of MG-63 cells cultured for 24 and 48 h in a 3D bioreactor system.
ns = non-significant, * $p < 0.05$, *** $p < 0.001$ vs. corresponding control.

| | | **24 h** | | **48 h** | |
|---|---|---|---|---|---|
| | | Mean | SD | Mean | SD |
| 5 ml/min | Relative cell count uncoated | 100% | 132% | 100% | 138% |
| | Relative cell count coated | 49% $^{*}$ | 89% | 44% $^{*}$ | 81% |
| | Circularity uncoated | 0,57 | 0,15 | 0,57 | 0,15 |
| | Circularity coated | 0,58 $^{ns}$ | 0,14 | 0,63 $^{***}$ | 0,14 |
| | Single cell area uncoated [$\mu m^2$] | 971,0 | 777,1 | 1174,0 | 904,6 |
| | Single cell area coated [$\mu m^2$] | 989,3 $^{ns}$ | 609,4 | 1293,0 $^{ns}$ | 850,6 |
| 10 ml/min | Relative cell count uncoated | 100% | 58% | 100% | 70% |
| | Relative cell count coated | 33% $^{***}$ | 36% | 32% $^{***}$ | 47% |
| | Circularity uncoated | 0,57 | 0,13 | 0,55 | 0,14 |
| | Circularity coated | 0,62 $^{***}$ | 0,16 | 0,56 $^{ns}$ | 0,12 |
| | Single cell area, uncoated [$\mu m^2$] | 1282,0 | 1141000 | 1355,0 | 1340,0 |
| | Single cell area coated [$\mu m^2$] | 1032,0 $^{***}$ | 754144 | 1426,0 $^{ns}$ | 938,7 |

### P283

### Design of nanocarriers for macrophage targeting to promote skeletal muscle regeneration

*Lining Xu*[1,2], *Dingding Wang*[1,2], *Quanzhong Ren*[1,2], *Linlin Fan*[1,2], *Renxian Wang*[1,2], *Yajun Liu*[2]
[1]*Beijing Research Institute of Traumatology and Orthopaedics, JST sarcopenia Research Centre, Beijing, China ;* [2]*Beijing Jishuitan Hospital- Capital Medical University, National Center for Orthopaedics, Beijing, China*

### Abstract Text

Sarcopenia is a condition characterized by decreased skeletal muscle mass, strength, and function, which can increase the risk of falls and fractures. It is one of the most common geriatric syndromes. However, there are no clinical drugs specific for sarcopenia, and treatment options are limited, so new treatment strategies need to be developed urgently. Patients with sarcopenia are in a long-term chronic and systemic inflammatory state, and their muscle inflammatory microenvironment is a complex environment composed of myofibroblasts, muscle satellite cells, extracellular matrix/vascular stromal cells, and immune cells, in which macrophages play an important role. M1 and M2 macrophages are like two ends of a balance, maintaining a delicate and precise immune internal environment balance. However, high expression of M1 disrupts the homeostasis of M1-M2, forming a promoting immune microenvironment that inhibits skeletal muscle regeneration. Therefore, there is an urgent need to develop therapeutic agents that target macrophages in the muscle inflammatory microenvironment. Gold nanoparticles (Au NPs) have been widely studied and used in preclinical and clinical applications as tools for drug delivery, photothermal therapy, diagnostics, and biomedical imaging due to their unique physicochemical properties. Au NPs have excellent macrophage targeting ability and can be used as a drug delivery platform to regulate macrophage immune function. We constructed a library of Au NPs with different physicochemical properties, proved the biosafety of Au NPs, and explored the regulatory effect of physicochemical properties on macrophage immune polarization. Functionalized Au NPs to reverse macrophage M1 polarization and promote skeletal muscle regeneration may provide a clinical technical solution for the realization of immunotherapy for sarcopenia.

**P284**

**Ultrasound-based fracture risk discrimination in patients with diabetes mellitus**

*Angela Galindo[1], Kay Raum[1], Lukas Maurer[2], Edgar Wiebe[3], Paula Hoff[3], Seyed Hossein Seyed Aghamir[1], Ziyuan Liu[1], Nadege Leprêtre[3], Carolin Dehnen[3], Oliver Palmer[4]*
*[1]Charité – Universitätsmedizin Berlin, Center for Biomedicine - Imaging- Simulation & Stimulation, Berlin, Germany ; [2]Charité – Universitätsmedizin Berlin, Medizinische Klinik für Endokrinologie, Berlin, Germany ; [3]Charité – Universitätsmedizin Berlin, Medizinische Klinik m.S. Rheumatologie und Klinische Immunologie, Berlin, Germany ; [4]Aemedes Gruppe, Endokrinologikum Berlin, Berlin, Germany*

**Abstract Text**

**Introduction:** Diabetes mellitus (DM) is a common chronic metabolic disease that increases the risk of fragility fractures, with dual-energy X-ray absorptiometry (DXA)-based bone mineral density often underestimating fracture risk in these patients. Cortical Backscatter (CortBS) ultrasound, a radiation-free technique, examines cortical viscoelastic material and microstructural properties. This study aims to assess CortBS's discrimination performance in DM patients compared to the gold standard (DXA).

**Methods:** The study (DRKS00029331, EA4/140/22) included 48 women and 38 men with DM (Type I = 38, Type II = 46, Type_other = 2), aged > 49 years, along with 77 matched individuals without DM. DXA measured bone density in the lumbar spine and both femoral sites. CortBS measurements were taken in the anteromedial tibia using a SonixTOUCH with custom software. Multivariate analysis of variance assessed the impact of DM type on CortBS and DXA. Partial least squares discriminant analyses with cross-validation were used to compare the discrimination performance for vertebral, non-vertebral, and any fragility fractures, considering gender, age, and anthropometric information (AP: weight, height, BMI).

**Results:** The DXA T-score was influenced by AP factors and age and was significantly higher in DM patients than in those without DM (Type I: +0.73 SD, Type II: +1.36 SD), but not significantly different between patients with and without fractures. The CortBS risk score was independent of AP factors, gender, and DM, and was significantly larger in patients with non-vertebral fractures. CortBS outperformed DXA for all fracture types, the difference was significant for vertebral and non-vertebral fractures (AUCDXA ≤ 0.58, AUCCortBS ≤ 0.64, Fig. 1). Irrespective of DM status, patients with fractures exhibited an elevated fracture risk in the ultrasound score, but not a decreased DXA T-score (Fig. 1).

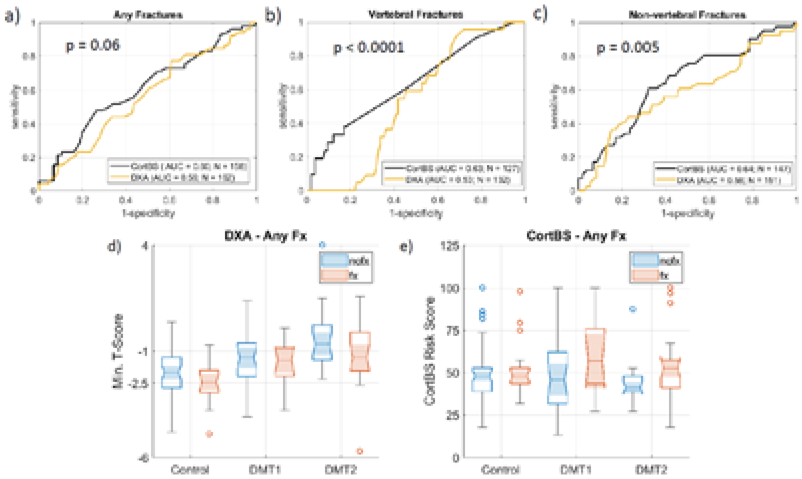

Fig. 1 ROC-AUC with 5-fold cross-validation for a) any, b) vertebral and c) non-vertebral fragility fractures for all study participants (independent of DM disease). DXA T-scores (d) show the well-known "overestimation" in patients with DM. The CortBS risk score (e) is particularly elevated in patients with DM type I and fractures.

**Discussion:** CortBS showed equivalent or better discrimination performance and more accurate risk assessment in patients with DM compared to DXA.

**P285**

**Cortical ultrasound spectroscopy discriminates fragility fractures in women and men better than bone mineral density**

*Angela Galindo[1], Gabriele Armbrecht[2], Kay Raum[1], Edgar Wiebe[3], Nadège Leprêtre[3], Ziyuan Liu[1], Seyed Hossein Seyed Aghamiry[1]*
*[1]Charité – Universitätsmedizin Berlin, Center for Biomedicine - Imaging- Simulation & Stimulation, Berlin, Germany ; [2]Charité – Universitätsmedizin Berlin, Klinik für Radiologie Röntgendiagnostik, Berlin, Germany ; [3]Charité – Universitätsmedizin Berlin, Medizinische Klinik m.S. Rheumatologie und Klinische Immunologie, Berlin, Germany*

**Abstract Text**

**Introduction:** Cortical ultrasound spectroscopy (CortBS) is a novel non-ionizing imaging technique that measures viscoelastic material properties and intracortical pore size distribution in the tibia. A preliminary study in postmenopausal women with low bone mineral density confirmed better fracture discrimination of CortBS over the gold standard (dual-energy X-ray absorptiometry DXA). This research aims to evaluate the discrimination performance of CortBS in male and female patients and young healthy volunteers and compare it with DXA.

**Methods:** The study (DRKS00025849, EA4/068/19) contained 144 women (including 55 postmenopausal women from a previous study [1]) and 33 men (age: 30 - 86 years, DXA: > 56 years). DXA bone density was taken at the lumbar spine and both femoral sites. CortBS measurements were performed in the anteromedial region of the tibia using a SonixTOUCH with custom software. Multivariate analysis of variance investigated the influence of gender, age and anthropometric (AP) information (height, weight, BMI) on CortBS and DXA. Partial least squares (PLS) discriminant analyses with cross-validation were used to compare the discrimination performance for vertebral, non-vertebral and any fragility fractures with and without gender, age and AP information.

**Results:** The DXA T-score was affected by weight and BMI and was significantly lower in patients with non-vertebral fractures ($p<0.05$). CortBS fracture risk score was significantly increasing with age ($p<0.01$) and significantly higher in patients with fractures ($p<0.005$). CortBS outperformed DXA's discrimination ability for all fracture types ($AUC_{DXA} \leq 0.60$, $AUC_{CortBS} \leq 0.72$, Fig. 1). Regardless of gender, patients with fractures showed an increased fracture risk in the ultrasound score, but not in the DXA T-score (Fig. 1).

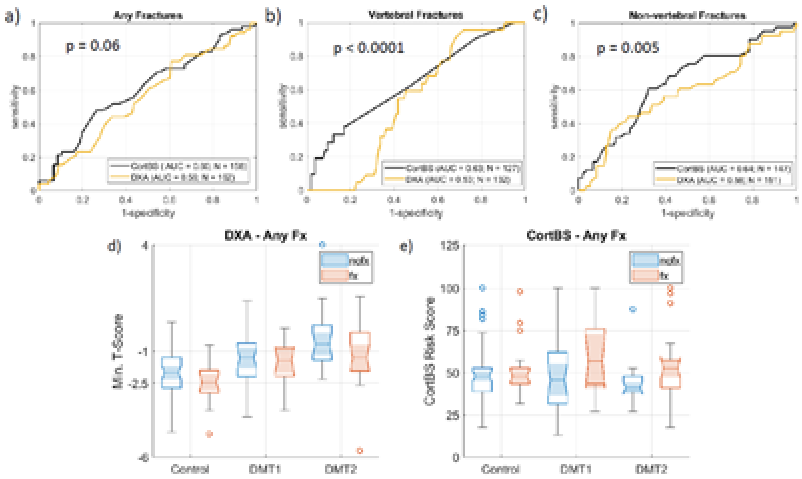

Fig. 1 ROC-AUC with 5-fold cross-validation for a) any, b) vertebral and c) non-vertebral fragility fractures for all study participants (independent of DM disease). DXA T-scores (d) show the well-known "overestimation" in patients with DM. The CortBS risk score (e) is particularly elevated in patients with DM type I and fractures.

**Discussion:** CortBS exhibits the general age- and gender-associated fracture risk and has a significantly better discrimination performance than DXA.

**Reference:** 1.Armbrecht et al. JBMR Plus, 2021, e10536.

**P286**

**New concept of motion artifacts compensation in HR-pQCT**

*Paul Jürß[1], Tobias Knopp[1], Mirco Grosser[1], Björn Busse[2], Marija Boberg[1], Felix Nikolai Schmidt[2]*
*[1]Technical University Hamburg, Institute for Biomedical Imaging, Hamburg, Germany ; [2]University Medical Center Hamburg-Eppendorf, Department of Osteology and Biomechanics, Hamburg, Germany*

**Abstract Text**

High-resolution peripheral quantitative computed tomography (HR-pQCT) is a versatile tool for clinical bone-microstructure and density measurements to detect alterations in osteoporosis and monitor treatment success. However, long scanning times of about two minutes create a high susceptibility for image artefacts due to patient motion, thus hampering the diagnostic accuracy. Today, about 20% of the scans have to be invalidated due to high motion artefacts.

Here, we examine possibilities to address the problem of motion artefacts in HR-pQCT imaging without using external markers or repeated measurements. Therefore, we established an iterative approach to estimate and compensate motion artefacts increasing the accuracy of the imaging and thus clinical parameters for monitoring. The approach involves finding the motion vector that minimizes the non-convex reprojection distance, ensuring the reconstructed image closely resembles the measured sinogram.

This approach was validated using a numerical study utilizing a non-corrupted HR-pQCT image stack. Simulated motion corrupted sinograms incorporating normally distributed noise were used for evaluation of the proposed method. Results do indicate significant improvements of the structural-similarity-index (SSIM) and visual perception, indicating the method's effectiveness in reducing motion artifacts. The method was implemented in Phyton with GPU accelerated reconstruction routines. Results exhibited a smooth objective function with a distinct minimum, facilitating efficient parameter optimization.

The versatility of this approach was demonstrated by applying it to different simulated motion displacements reflecting the potential use for multiple clinical motion cases. However, the calculation time was 2.5 hours. Yet, since results are not needed in a short curse of time, the reduction of irradiation and reduced need of repeated scan may outweigh the high calculation times. Future studies are however needed to explore the full range of motion artefacts that can be corrected. Additionally, experimental setups are needed to generate real world data with motion artefacts to proof validity for a clinical use.

**ND-P026**

**Erythropoiesis-stimulating agent and risk of osteoporotic fracture among chronic kidney disease patients: A territory-wide nested case-control study**

*Shun-Cheong Ho[1], Suhas Krishnamoorthy[1], Chor-Wing Sing[1], Ching-Lung Cheung[1]*
*[1]The University of Hong Kong, Department of Pharmacology and Pharmacy, Hong Kong, Hong Kong*

**Abstract Text**

**Background:** Erythropoietin was linked to bone metabolism in preclinical studies, but the relationship remained inconclusive in humans. Therefore, the association between erythropoiesis-stimulating agent (ESA) use and bone metabolism needs further investigation.

**Methods:** Using the electronic health record from the Hong Kong Hospital Authority, we conducted a nested case-control study among patients with chronic kidney disease (CKD) using ESA between 2005 and 2022. Incidence cases of osteoporotic fracture were matched with up to 10 fracture-free controls on age, sex, and year of fracture incidence. We estimated the odds ratio (OR) and 95% confidence interval (CI) for the association of ESA with fracture risk using conditional logistic regression, adjusted for unbalanced CKD-related risk factors after matching.

**Results:** 959 osteoporotic fracture cases were identified and matched with 9262 fracture-free control. The duration of EAS treatment was associated with an increased risk of osteoporotic fracture [OR per year of ESA treatment: 1.27, 95% CI: 1.21-1.33, p<0.001]. An increase in accumulated ESA dose was associated with increased fracture risk, but the effect was attenuated after adjustment for ESA treatment duration. Consistent findings were observed in the spine and hip fracture subtypes. Similar results were observed in subgroup analysis stratified by age, sex, CKD stages, and dialysis history, and in the sensitivity analysis with adjusted prescription duration for iron supplementation and average haemoglobin level since initiation of ESA treatment.

**Conclusion:** This study suggests a potential relationship between the use of ESA and bone metabolism among patients with CKD. The management of anaemia management in CKD patients with ESA might warrant a shorter treatment duration.
